# HeteroMRI: Robust white matter abnormality classification across multi-scanner MRI data

**DOI:** 10.1093/gigascience/giaf092

**Published:** 2025-08-21

**Authors:** Masoud Abedi, Navid Shekarchizadeh, Pierre-Louis Bazin, Nico Scherf, Julia Lier, Christa-Caroline Bergner, for the Alzheimer’s Disease Neuroimaging Initiative, Wolfgang Köhler, Toralf Kirsten

**Affiliations:** Faculty Applied Computer and Bio Sciences, Mittweida University of Applied Sciences, 09648 Mittweida, Germany; Department for Medical Data Science, Leipzig University Medical Center, 04103 Leipzig, Germany; Institute for Medical Informatics, Statistics, and Epidemiology (IMISE), Leipzig University, 04107 Leipzig, Germany; Department for Medical Data Science, Leipzig University Medical Center, 04103 Leipzig, Germany; Institute for Medical Informatics, Statistics, and Epidemiology (IMISE), Leipzig University, 04107 Leipzig, Germany; Center for Scalable Data Analytics and Artificial Intelligence (ScaDS.AI) Dresden/Leipzig, Leipzig University, 04105 Leipzig, Germany; Full Brain Picture Analytics, 2332 XB Leiden, The Netherlands; Neural Data Science and Statistical Computing, Max Planck Institute for Human Cognitive and Brain Sciences, 04103 Leipzig, Germany; Center for Scalable Data Analytics and Artificial Intelligence (ScaDS.AI) Dresden/Leipzig, Leipzig University, 04105 Leipzig, Germany; Department of Neurology, Leipzig University Medical Center, 04103 Leipzig, Germany; Myelin Research Center (MRC) Leipzig, Department of Neurology, Leipzig University Medical Center, 04103 Leipzig, Germany; Department of Neurology, Leipzig University Medical Center, 04103 Leipzig, Germany; Myelin Research Center (MRC) Leipzig, Department of Neurology, Leipzig University Medical Center, 04103 Leipzig, Germany; Department of Neurology, Leipzig University Medical Center, 04103 Leipzig, Germany; Myelin Research Center (MRC) Leipzig, Department of Neurology, Leipzig University Medical Center, 04103 Leipzig, Germany; Faculty Applied Computer and Bio Sciences, Mittweida University of Applied Sciences, 09648 Mittweida, Germany; Department for Medical Data Science, Leipzig University Medical Center, 04103 Leipzig, Germany; Institute for Medical Informatics, Statistics, and Epidemiology (IMISE), Leipzig University, 04107 Leipzig, Germany; Center for Scalable Data Analytics and Artificial Intelligence (ScaDS.AI) Dresden/Leipzig, Leipzig University, 04105 Leipzig, Germany

**Keywords:** brain MRI classification, multi-scanner MRI, multi-protocol MRI, intensity clustering, white matter abnormality, rare disease, convolutional neural network

## Abstract

**Background:**

Magnetic resonance imaging (MRI) is commonly used for analyzing white matter abnormalities in the human brain. Integrating machine learning into MRI analysis can enhance diagnostic processes. However, the application of such techniques for white matter analysis in clinical practice is often limited when MRI data are multi-scanner (i.e., heterogeneous), particularly in scenarios with limited data, as seen in rare diseases. Therefore, it is crucial to develop methods that are highly independent of the MRI scanner and acquisition protocol.

**Results:**

This study introduces HeteroMRI, a deep learning method for classifying MRIs based on white matter abnormalities. Most importantly, HeteroMRI mitigates the effects of data heterogeneity on classification performance. Herein, HeteroMRI is employed to detect brain MRIs with white matter abnormalities. This method utilizes intensity clustering of the white matter tissue to reduce the effects of the heterogeneity of MRIs. MRI data from 11 public datasets with 40 MRI protocols are included. By using 200 MRIs for training the model, the binary classifier achieves an average accuracy of 93% ± 4%. Furthermore, the method is evaluated in limited data scenarios, simulating conditions of rare diseases. By reducing the data by 64% and 75%, the model’s accuracy has a 4% and 12% decrease, respectively.

**Conclusions:**

The presented method opens new avenues for white matter abnormality-related classification of heterogeneous MRI data without additional machine learning methods to reduce MRI heterogeneity. This classification approach demonstrates a high degree of independence from the MRI scanner and protocol, while also proving to be relatively generalizable to unseen MRI protocols.

## Introduction

Magnetic resonance imaging (MRI) of the brain is widely used to diagnose neurological diseases as it provides a clear contrast between the different tissues of the brain, including white matter (WM) and gray matter (GM) [1]. Detection and assessment of WM abnormalities or lesions in demyelinating or neurodegenerative diseases are an important application of MRI in daily clinical practice [2]. An excellent contrast for visualizing WM abnormalities is provided by the fluid-attenuated inversion recovery (FLAIR) imaging technique, making the abnormalities stand out from the surrounding normal brain tissue. FLAIR is a T2-weighted imaging technique in which the signal from cerebrospinal fluid (CSF) is suppressed, which facilitates the detection of WM abnormalities as they may appear adjacent to CSF-filled spaces [3]. The MRI technique allows for the study of the pattern and volume of WM lesions, which ultimately contributes to image-based diagnosis of demyelinating and other neurological disorders [4].

In recent years, artificial intelligence (AI) has revolutionized the medical imaging domain, offering powerful tools for automating time-consuming tasks such as lesion segmentation [5]. This reduces examiner-based variability and enables health-care professionals to focus on critical aspects of diagnosis and research. In brain MRI analysis, machine learning (ML) and deep learning (DL) models have been widely applied [6] for tasks, including disease classification [7–9], WM lesion segmentation [10,11], tumor detection and grading [12, 13], stroke lesion analysis [14, 15], brain age prediction [16, 17], and brain tissue segmentation [18, 19]. Additionally, AI-based algorithms are commonly used in MRI preprocessing steps such as image registration [20], brain extraction [21, 22], denoising [23–25], intensity normalization [26, 27], bias field correction [28], and MRI interpolation [29].

A challenge in using MRI data in ML/DL models is the variability of MRIs across different sites and scanners. It is shown that scanner differences lead to significant biases in automated MS lesion volumetric analyses, even when the scanner manufacturer and acquisition protocol are consistent [30]. Acquisition protocol refers to a set of procedures and parameters (e.g., echo time [TE], repetition time [TR], and inversion time [TI]), used to acquire the images. The variabilities due to different scanners and acquisition protocols are often greater than the biological variabilities [31–36]. High-capacity classifiers, such as deep neural networks, often struggle to produce consistent outcomes when applied to multi-scanner data. This limitation is caused by the model’s tendency to overfit to non-biological variations; thus, the model fails to detect desired biological features or to generalize well across MRI data from unseen scanners [37].

A common approach to address scanner variability is using standardized MRI datasets—acquired with identical scanners and protocols—to improve model consistency [38, 39]. While standardization enhances image comparability and model performance, it requires substantial coordination and resources. Moreover, many DL models still fail to generalize to unseen scanners or protocols [40], limiting their clinical utility. Thus, methods that are robust to scanner and protocol variability are crucial.

Another approach to address scanner-related heterogeneity in MRI data is *harmonization*, which aims to remove scanner and protocol effects before analysis. Statistical techniques such as intensity normalization [26, 41] or ComBat-based batch effect correction [31–36] have shown success to some extent, but often fail to improve DL model performance in disease classification tasks [37]. More recent harmonization approaches rely on supervised [42, 43] and unsupervised [44, 45] DL methods, with hybrid models like DeepComBat also emerging [46]. While these methods aim to learn and remove scanner-specific features, they often rely on restrictive assumptions, such as availability of matched subjects across scanners [42], standardized acquisition protocols across scanners [44], necessity of multi-contrast data from the same session [45], or requiring a large amount of training data [44]. Furthermore, there is still no universal harmonization strategy, and even after harmonization, DL models must still be robustly generalizable to unseen scanners and protocols. For an extensive review of MRI harmonization methods, see [47].

On a different note, the context of rare diseases has specific challenges and limitations. Data availability is extremely limited, which severely restricts the application of ML/DL approaches to these diseases, including both predictive models, such as classifiers, and harmonization methods. For example, in the case of leukodystrophies [48], the brain MRIs are gathered from various clinical centers over a long period, making it infeasible to even create a standardized dataset. Such datasets are not only small in size but also highly heterogeneous in terms of MRI scanners and protocols. These conditions significantly hinder the applicability of conventional ML methods to rare diseases.

Several existing methods address WM abnormality analysis, such as lesion segmentation or volumetric assessments, typically using supervised deep learning models. Many of these approaches require large amounts of voxel-wise, manually annotated data for training [49–51]. Some methods specifically target the segmentation of lesions within the brain [11, 52–55], whereas this study focuses on classifying MRIs based on the WM abnormalities. Additionally, several segmentation models require the availability of multiple MRI sequences (e.g., FLAIR, T1, T2) [19, 38, 56], while the approach presented in this study is designed to operate solely on FLAIR images, enhancing its practicality and ease of deployment across heterogeneous clinical datasets.

Herein, we present HeteroMRI, an approach for classifying brain MRIs based on WM abnormalities while mitigating the heterogeneity effects of the images acquired from multiple scanners and acquisition protocols. In this article, HeteroMRI is utilized to detect brains with WM abnormalities in FLAIR images through binary classification. HeteroMRI is applicable to multi-scanner and multi-protocol datasets and demonstrates effectiveness in data-limited conditions, providing a flexible and practical solution for both research and clinical applications. The presented method employs MRI intensity clustering, a technique used in the literature for other MRI-related purposes such as brain tissue segmentation [57, 58], brain tumor segmentation [59], and inhomogeneity correction [60]. HeteroMRI is evaluated in various experimental settings to ensure its robustness. Additionally, we apply the method to limited data scenarios in order to assess the performance and applicability of the presented method for rare diseases. In future work, the method is intended to be applied to the task of classifying 2 WM diseases based on their distinct WM abnormality patterns. The presented method opens new avenues for performing WM abnormality-related analyses on heterogeneous MRI datasets and the large amount of MRI data generated daily in medical centers. The current article is structured in the following way: the second section provides an overview of the methodology used, detailing the data preprocessing steps and the architecture of the DL model employed in this study. Moving on to the third section, the experiments, the datasets used, the experimental settings, the execution of the model, and the evaluation metrics are presented. Following that, the fourth section presents the key results, while the fifth section discusses the results, highlights the limitations of the method, and introduces the possible future directions. Finally, the sixth section provides the conclusion.

## Methodology

**Figure 1:**
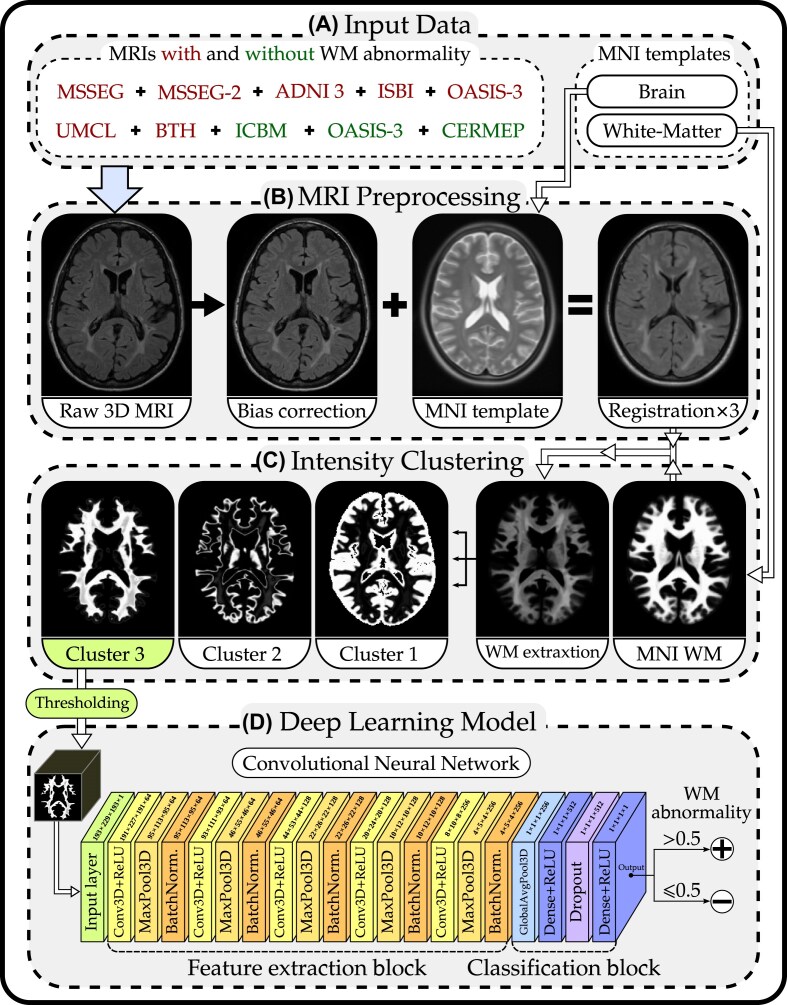
Overview of the methodology. (A) Input data: The MRI datasets used for the classification model and MNI brain template [61, 62]. The MRI data with and without WM abnormality are taken from the datasets shown in red and green, respectively. (B) MRI preprocessing: The N4 bias field correction method [28] is applied on the FLAIR MRIs (in 3D), and then the MRIs are 3 times registered (nonlinearly) to the MNI template. (C) Intensity clustering: The WM of the brain is extracted, and the WM is clustered into 3 intensity clusters using the RFCM [63] algorithm. (D) DL model: Only cluster 3 of the WM is thresholded and used for a binary classification model with the CNN architecture shown.

The WM abnormality detection approach presented in this article uses heterogeneous brain MRI data with various acquisition protocols (multi-protocol) as the input data for a convolutional neural network (CNN). The model is a binary classifier trained to detect patients with WM abnormalities in their brain MRI. The method consists of 3 main modules explained in the following subsections, namely, MRI preprocessing, intensity clustering, and DL model. An overview of the methodology is illustrated in Fig. 1.

### MRI preprocessing

For preparing the image data for the analysis, we use our brain MRI preprocessing pipeline, *FlexiMRIprep* (https://github.com/ul-mds/FlexiMRIprep), that consecutively performs all the requested preprocessing steps/algorithms on all the selected images automatically. Being the optimal MRI sequence in detecting WM abnormalities, only FLAIR images are used in the analysis in this article. All selected images have a minimum of 128, 192, and 22 voxels in their first, second, and third dimensions, respectively. All the MRIs are converted to the Neuroimaging Informatics Technology Initiative (NIfTI-1) format using the dcm2niix tool (version 1.0.20211006) [64] at this point. The preprocessing steps described below are applied identically to all MRIs from different datasets. Detailed information on the parameters used in each step is reported in the GitHub repository of HeteroMRI (https://github.com/ul-mds/HeteroMRI).

- **Bias field correction**: For correcting the bias field or inhomogeneity issues in the MRIs, we employ the commonly used N4ITK [28] bias field correction method for this purpose. For implementation, the N4BiasFieldCorrectionImageFilter class from the SimpleITK [65] (version 2.1.1.2) *Python* library with the default parameters is used.
- **Registration**: Registration enables precise spatial mapping and the comparison of anatomical structures among the MRIs. In this process, all the MRIs used for training and testing the AI model are aligned to a standard brain template. Among the available brain templates, we chose the “ICBM 2009c Nonlinear Asymmetric” template [61, 62] (referred to below as the Montreal Neurological Institute [MNI] template), which the developers created using the data from the International Consortium for Brain Mapping (ICBM) project [66]. This template was selected due to its high accuracy and the availability of the WM probability map required for our analysis approach. Since there is no dedicated FLAIR template in the MNI template, the T2-weighted template was used due to its proximity to FLAIR. For the registration, the antsRegistration tool from the Advanced Normalization Tools (ANTs) [67] (version 2.4.4) is employed. A nonlinear registration is applied 3 times (with identical parameters) on each image consecutively. This repeated registration aims to achieve a high level of alignment of the MRIs with the template. Mutual information (MI) was calculated between each registered MRI and the MNI template, after each of the 3 registration steps. As shown in Supplementary Fig. S1, the MI increased after each nonlinear registration step, with mean values improving from 0.62 (first registration) to 0.73 (second registration) and 0.80 (third registration). However, the improvement between the second and third steps was relatively small (ΔMI ≈ 0.072), indicating diminishing returns. This suggests that 3 registration steps are sufficient to achieve consistent and reliable alignment. Performing additional iterations may not justify the computational cost and could even introduce unnecessary anatomical distortions, particularly due to the nonlinear nature of the transformations. Multi-pass registration has also been used by others, for example, to address large differences in the initial positions of image pairs [68]. The registered MRIs all have a size of $193\times 229\times 193$ voxels with a voxel size of $1\times 1\times 1$ mm.

### Intensity clustering

- **WM extraction**: After the brain images are aligned with the MNI template, the WM probability map of the template is used to extract the WM volumes of each brain. Therefore, all the other brain tissues are removed. The WM extraction is performed by using the MultiplyImages tool from ANTs.
- **WM clustering**: The WM clustering is performed for 2 essential purposes: (i) to obtain a subgroup of WM volumes that includes significant signs of WM abnormalities and (ii) to reduce the negative effect of the heterogeneous MRI data coming from multiple scanners and protocols. These 2 points are elaborated on in the following.
  A clustering algorithm is used to estimate 3 intensity clusters from the WM volumes obtained in the previous step. The algorithm groups the WM volumes into 3 subgroups that share a relatively similar intensity range. We expect that one of the clusters shows more indications of WM abnormalities (if present in the brain) since the abnormalities have higher intensity values in FLAIR images. This specific cluster will be used as the training data for the classifier model. The cluster is a membership function with float values in the range of [0,1] for each voxel. As a result, the impact of the heterogeneous nature of the multi-protocol MRIs is reduced, in a way that the proposed method is robust to the many MRI protocols we used in this work. The decision to use 3 clusters was based on our empirical observations from a dataset different from those used in this study. Through testing various cluster numbers on different MRIs, we found that 3 clusters yielded consistently comparable patterns in nearly all MRIs. In other words, the shapes of the clusters in 1 MRI were generally consistent with those in another MRI. This was also later observed in the data of the current study. Therefore, the choice of 3 clusters is robust and does not depend on a specific dataset. Here, we employ a robust fuzzy C-means (RFCM) algorithm [63] for WM intensity clustering. The RFCM algorithm modifies the standard fuzzy C-means (FCM) objective function by incorporating a local spatial penalty term, leading to the computation of smoother membership functions. This modification not only improves segmentation performance but also provides a level of noise insensitivity. The RFCM algorithm is implemented using the fuzzy_cmeans function available in the *Nighres* (Neuroimaging at high resolution) *Python* package, version 1.4.0 [69]. Upon examining the 3 WM intensity clusters in MRIs with WM abnormalities, we noted that one of the clusters within each MRI, cluster 3 in Fig. 1C, consistently exhibited significant lesion-related features. Therefore, from each MRI, we should take the cluster that looks visually similar to cluster 3 in Fig. 1C, but it is not always the cluster number 3. For this purpose, we use the Dice similarity coefficient [70] to compare the 3 clusters of each MRI with a fixed reference cluster to detect the most similar one. The reference image (available in the GitHub repository of HeteroMRI) is generated by averaging the intended intensity cluster of 4 MRIs from a clinical dataset. This method detected the right cluster for all the MRIs of this study correctly (i.e., with 100% accuracy) as checked manually.
- **Thresholding**: A thresholding is applied on the selected WM intensity cluster of each MRI. All the intensity values below 0.5 are ignored in order to remove uncertain, low-confidence assignments and retain only the core voxels that are strongly associated with the cluster. The value 0.5 is chosen experimentally in the design phase of HeteroMRI by using (clinical) datasets different from those used in this study for the task of classifying 2 WM diseases. The value 0.5 resulted in the highest improvement in the classification accuracy compared to other tested thresholds. The histogram for most MRIs follows the same overall pattern: approximately 50% ± 5% of intensity values are below 0.2, and around 35% ± 5% are above 0.8. Supplementary Fig. S4 shows the WM cluster of a sample MRI before and after thresholding along with their normalized histograms (for the 99% upper percentile). Finally, the thresholded clusters from the MRIs (1 intensity cluster per MRI) are used as training data for the DL model, as described in the following section.

### Deep learning model

The objective is to train a binary classifier model that detects the brain MRIs that have WM abnormalities. Inspired by [71, 72], we configured a 3D CNN comprising a total of 20 layers, as illustrated in Fig. 1D. The network has a total of 1,795,905 parameters. The model begins with the input layer, followed by a feature extraction block, and ends with a classification block. In the feature extraction block, we employ 5 3D Convolution (Conv3D) layers with 64, 64, 128, 128, and 256 filters, respectively. Each Conv3D has a 3 × 3 × 3 kernel size and employs the rectified linear unit (ReLU) activation function. Subsequently, each Conv3D layer is succeeded by a 3D Max Pooling (MaxPool3D) layer with a stride of (2,2,2) and a pool size of (2,2,2), which downscales the 3-dimensional (3D) input by half in each dimension. Batch normalization [73] layers with default parameters follow each MaxPool3D layer.

In the classification block, a 3D Global Average Pooling (GlobalAvgPool3D) layer is followed by a dense layer with a dimensionality of 512 and with a ReLU activation function. To help prevent overfitting, a dropout layer with a 30% rate is introduced next. Finally, the output layer performs a binary classification employing a sigmoid activation function. The binary cross-entropy loss, Adam optimizer [74], and an *Early Stopping* feature (*patience* = 40) are employed in the model. In each epoch, the checkpoint feature saves the model if the validation accuracy has improved. In the case of an unchanged validation accuracy, the mode is saved if the validation loss has decreased. The *Python* implementation code of the HeteroMRI method is publicly available (https://github.com/ul-mds/HeteroMRI) .

## Experiments

Different MRI datasets are used along with multiple experimental settings with various conditions to train and evaluate the CNN model for classifying brain MRIs. In the following subsections, the datasets and the experimental settings are elaborated.

### Datasets

In this study, we utilized FLAIR images from multiple brain MRI datasets as introduced below. Incorporating a combination of MRIs with a high diversity of acquisition protocols and scanners ensures a robust evaluation of the presented methodology. All the datasets used in this study are either publicly available or are accessible upon request to the respective dataset providers. All the MRIs were visually checked by the authors to exclude images with large artifacts. As presented in Table 1, a total of 11 MRI datasets are utilized.

**Table 1.** MRI datasets used in this study. M/F: number of male/female subjects; Age: mean ± standard deviation (years)

No.	Dataset name/alias	Images^1^		Demographics		Protocols^2^	Availability	Reference
		+	–	M/F	Age			
1	ISBI	19	0	4/15	40.4±9.2	1	Public	[75]
2	UMCL	30	0	7/23	median 39 (25–64)	1	Public	[76]
3	MSSEG	52	0	15/37	45.3±10.3	4	AoR^3^	[77]
4	MSSEG-2	40	0	N/A	N/A	10	AoR^3^	[78]
5	BTH	9	0	1/8	29.4±8.9	2	Public	[79]
6	ICBM	0	5	2/3	28.2±7.5	1	AoR^3^	[80]
7	OASIS-3	14	90	50/54	68.4±9.5	4	AoR^3^	[81]
8	ADNI 3	58	0	28/30	76.5±8.6	8	AoR^3^	[82]
9	CERMEP	0	27	14/13	34.9±9.3	1	AoR^3^	[83]
10	WMH	10	0	N/A**	N/A**	5	Public	[84]
11	PPMI	0	10	3/7	52.7±13.2	4	AoR^3^	[85]
	Sum	232	132	-	-	40*	-	-

^1^Number of FLAIR images, with (**+**) and without (**–**) WM abnormality.

^2^Number of MRI protocols in the used data.

^3^Accessible on request (to the respective dataset provider).

*Sum of unique protocols in the data.

**Not available on individual level.

The details of each dataset are outlined below:

1. **ISBI**: The International Symposium on Biomedical Imaging (ISBI) in 2015 [75] conducted a multiple sclerosis (MS) lesion segmentation challenge using longitudinal MRI data. The dataset comprises imaging data from MS patients, acquired using the same scanner and protocol. We utilize 19 FLAIR images from this dataset. For each patient, the MRI taken at the latest time point is used.
2. **UMCL**: A cohort of MS patients was imaged at the University Medical Center Ljubljana (UMCL) [76]. The images were acquired using the same scanner and protocol. We use 30 3D FLAIR images from this dataset.
3. **MSSEG**: The MSSEG dataset [77] was presented for the MS lesion segmentation challenge during the MICCAI 2016 conference. The dataset contains MRIs of MS patients from 4 different sites. Each site used different MRI scanners and protocols. We utilize 52 FLAIR images (the MSSEG dataset originally contains 53 FLAIR images, but one of them was inadvertently excluded from our analysis) from this dataset.
4. **MSSEG-2**: Data were generated by participating neurologists in the framework of Observatoire Français de la Sclérose en Plaques (OFSEP), the French MS registry [86]. They collect clinical data prospectively in the European Database for MS (EDMUS) software [87]. MRIs of patients were provided as part of a care protocol. Nominative data are deleted from the MRI before transfer and storage on the Shanoir platform (Sharing NeurOImagingResources, shanoir.org). MSSEG-2 [78] presents a challenge for segmenting new MS lesions in the brain, as presented at the MICCAI 2021 conference. At the time of the current research, only the training data of the dataset is accessible. The images of the training set were acquired at 12 different sites and using 10 different scanners. All the images were acquired at 2 different time points from each patient. From this dataset, we utilize 40 3D FLAIR images from the second time point.
5. **BTH**: The brain MRI dataset of MS patients from Baghdad Teaching Hospital (BTH) [79] includes MRIs taken at 20 centers with different protocols. We used 9 FLAIR images from this dataset, which were taken using 2 different protocols: the NIfTI files in this dataset lack orientation information (qform and sform), making it impossible for the registration algorithm to identify the correct orientation of the brain. Additionally, the MRIs are 2-dimensional (2D), resulting in around 10 times fewer slices than the pixels in the first and second dimensions, and they all share the same pixel thickness across all 3 dimensions. Consequently, the brain appears unrealistically short in 3D view. Due to these dataset-specific conditions, we applied 2 additional preprocessing steps at the beginning for this dataset: (i) added correct orientation information to each file and (ii) edited slice thicknesses in the header of NIfTI files based on slice thickness information provided in the dataset’s metadata. However, the height of many images still does not appear realistic and may cause problems for the registration. Therefore, only 9 images were used.
6. **ICBM** (ICBM project [Principal Investigator John Mazziotta, M.D., University of California, Los Angeles]) is supported by the National Institute of Biomedical Imaging and BioEngineering. ICBM is the result of efforts of coinvestigators from UCLA, Montreal Neurologic Institute, University of Texas at San Antonio, and the Institute of Medicine, Juelich/Heinrich Heine University. The International Consortium for Brain Mapping (ICBM) [80] has developed a probabilistic atlas and reference system for the human brain for normal adults. The dataset includes 20 3D FLAIR images; however, only 5 were selected for this study, as our neurology specialist confirmed these to be the only ones free of WM abnormalities. The images were acquired using the same scanner and protocol.
7. **OASIS-3**: The Open Access Series of Imaging Studies (OASIS) project provides publicly available neuroimaging datasets. Among its releases, only OASIS-3 [81] includes FLAIR images, encompassing both cognitively normal individuals and those at various stages of cognitive decline. Approximately 850 subjects in OASIS-3 have FLAIR scans. For this study, we used 104 FLAIR and T2-FLAIR images acquired under 4 different MRI protocols. From a random subset of 600 images, 90 were selected by 2 neurology specialists based on the absence of WM abnormalities. The remaining 14 images were selected from the same pool to ensure representation of diverse WM lesion patterns, as confirmed through visual inspection.
8. **ADNI 3**: The Alzheimer’s Disease Neuroimaging Initiative (ADNI) was launched in 2003 as a public–private partnership, led by Principal Investigator Michael W. Weiner, MD. The primary goal of ADNI (https://adni.loni.usc.edu) has been to test whether serial MRI, positron emission tomography (PET), other biological markers, and clinical and neuropsychological assessment can be combined to measure the progression of mild cognitive impairment and early Alzheimer’s disease. The ADNI provides a rich repository of neuroimaging and clinical data [82]. From ADNI-3, which includes FLAIR scans for over 1,100 subjects, we selected 58 3D FLAIR images specifically based on the diversity of their WM lesion patterns. A neurology specialist verified that these images include a range of lesion types, including multifocal, confluent, and brainstem lesions.
9. **CERMEP**: The CERMEP-IDB-MRXFDG dataset [83] comprises MRI, computed tomography (CT), and [^18^F]FDG PET image data with the BIDS standard of healthy subjects. The dataset has 37 FLAIR images obtained using the same scanner and protocol. As reported in the original study, these images underwent visual review by 2 neurologists to confirm the absence of any apparent brain abnormalities. However, due to our strict criteria for even minor lesions, our neurologists confirmed only 27 MRIs as free of WM abnormalities for use as control data in our model.
10. **WMH**: The White Matter Hyperintensity (WMH) segmentation challenge dataset [84], introduced at the MICCAI 2017 conference, includes MRI scans from 170 subjects acquired using 5 different scanners. The MRIs have different loads of WM lesion. In this study, we used 10 FLAIR images from this dataset. From each scanner, 2 MRIs were taken (the first training and the first test MRI, as well as the first 2 test MRIs from the scanners that were used only as test data).
11. **PPMI**: The Parkinson’s Progression Markers Initiative (PPMI) [85] is a large-scale, longitudinal study that offers comprehensive imaging, clinical, and biospecimen data from individuals with Parkinson’s disease and healthy controls. For this study, we selected 10 FLAIR images without WM abnormalities, identified by a neurology specialist from approximately 220 healthy control subjects with available FLAIR scans.

From the MRI data explained above, the datasets 1 to 9 (344 images) are used for training and testing the model in the experimental settings explained below, and the datasets 10 and 11 (20 images) are used as holdout sets. Around 36% of these MRIs are 2D, based on our definition that MRIs with 70 or fewer slices are considered 2D. A comprehensive list of the MRI files is available in the GitHub repository of HeteroMRI, providing details for each image, including the subject ID from the original dataset and the acquisition protocol.

### Experimental settings

Various experimental settings have been designed for a robust evaluation of the presented classification approach. An experimental setting means the specification of the data used for training, validating, and testing the CNN model. By employing the datasets explained in “Datasets,” FLAIR images from different scanners and acquisition protocols are intentionally combined and used for training and testing the model. The images necessary for each setting are selected randomly from the MRIs available. The number of MRIs with and without WM abnormality is balanced in the training, validation, and test data of all the settings. There are 4 setting groups: $A, B, C,$ and *D*. In setting *A*, the data are selected based on datasets, while in the settings $B, C,$ and *D*, the data are incorporated based on their acquisition protocol. We assigned a protocol name to each of the MRIs based on the scanner name and model, magnetic field strength, and acquisition parameters. The protocol naming convention is explained in the HeteroMRI Github repository. The experimental settings are introduced as follows:

- **Setting *A***: In setting *A*, the goal is to evaluate HeteroMRI on a combination of MRIs from different datasets beginning from a relatively large number of data and then decreasing the data gradually. In setting *A*, there are 19 settings that are run independently. In *A*00, 244 MRIs from 9 datasets are used. The data of *each* dataset is split into training (70%), validation (10%), and test (20%) sets. In *A*01, the training data are downsized by approximately 10% while the test set retains the same images as in *A*00. The downsizing process continues up to *A*18, where the training and validation sets together include only 4 MRIs. The downsizing is performed by removing random MRIs while keeping the maximum possible number of protocols among the data. Across all settings from *A*00 to *A*18, the test set remains identical. Supplementary Table S1 shows the number of MRIs used for training, validation, and test sets from each dataset in the settings *A*00 to *A*18.
- **Setting *B***: In setting *B*, the goal is to choose the MRIs with the most diversity of protocols while having an equal number of MRIs from each protocol; therefore, the model sees the same number of MRIs per protocol. MRIs from 10 different protocols are incorporated. The test data are selected from all protocols. In *B*00, from each protocol, 5 MRIs for training, 1 MRI for validation, and 1 MRI for the test are used. In the subsequent settings, the training data are reduced. By *B*04, only 1 MRI per protocol is used for the training set. In Supplementary Table S2, the list of selected protocols and the number of MRIs used for training, validation, and test sets for *B*00 to *B*04 is reported.
- **Setting *C***: In setting *C*, the goal is to assess the generalizability of HeteroMRI to unseen MRI protocols. In this setting, MRIs from 8 different protocols are included. Only data from 3D MRIs are included in this setting due to the reason later explained later. MRIs from 6 protocols are used *only* in training and validation sets, while the other 2 protocols are *only* used in the test set. In fact, the trained model does not see any data from the protocols used in the test set during training. We consider 10 cases, in each case considering 2 different protocols for testing the model. The setting *C* begins with *C*00, which uses 64 MRIs for training and validation. This continues up to *C*06 with only 8 MRIs for training and validation. In Supplementary Table S3, the list of selected protocols and the number of MRIs used for training, validation, and test sets for *C*00 to *C*06 is reported.
- **Setting *D***: In setting *D*, the goal is to see the effect of the number of MRI protocols on the performance of the model. Beginning from *D*00 and going toward *D*03, more protocols are included in the data used for training and testing the model. At the same time, the total number of data and test set size are kept the same among the settings *D*00 to *D*03 (82 MRIs for training including validation data); therefore, it is possible to compare the results of the settings to see the effect of having more protocols in the data. In *D*00, there are MRIs from 4 protocols. In *D*01 to *D*03, there are MRIs from, respectively, 6, 8, and 10 protocols. The test data are selected from all protocols. In Supplementary Table S4, the list of selected protocols and the number of MRIs used for training, validation, and test sets for *D*00 to *D*03 is reported.

### Model execution

All the MRIs used in this study, introduced in “Datasets,” are preprocessed following the procedure elaborated in “MRI preprocessing.” Next, the intensity clustering procedure is applied to each preprocessed MRI, following the procedure introduced in “Intensity clustering.” As a result, a single intensity cluster per MRI is used for training or testing the model. Notably, the 3D intensity clusters obtained from the MRIs serve as the exclusive training data for the model. The model has no exposure to the original MRIs or any form of WM lesion annotation file. For the preprocessing and intensity clustering tasks, we used a machine with Intel Xeon Gold 6240R CPU @ 2.40 GHz and 128 GB of RAM. The preprocessing pipeline (introduced in “MRI preprocessing”) employs a parallelization approach in some of the preprocessing steps to make the procedure faster. The computation time required for preprocessing each MRI depends on multiple factors; nevertheless, the total number of voxels in the 3D MRI plays a more significant role. More specifically, based on our assessments, the number of slices of the magnetic resonance image highly affects the required computation time. Supplementary Fig. S2 shows the average time required for preprocessing and intensity clustering of 5 sample MRI dimensions.

The CNN model explained in “Deep learning model” is trained and tested on each experimental setting independently. In settings $A, B, C,$ and *D*, the required number of data is selected (and split into training, validation, and test sets) from all available data 50 independent times (referred to below as “data shuffle”). For each data shuffle, the model is trained and tested 10 times. For training and testing the CNN model, we used a computational server with an AMD Epyc 7352 CPU, 1 TB of CPU RAM, and an NVIDIA A100 GPU (40 GB GPU RAM). The average required time for training the model of each setting is shown in Supplementary Fig. S3. The inference time of the model on a single test data is a few seconds. The inference process can be efficiently performed without the necessity of a GPU.

### Evaluation metrics

To assess the performance of the classification model, we employ 5 common metrics: accuracy, sensitivity, specificity, F1 score, and precision. Moreover, the area under the receiver operating characteristic curve (AUROC) is reported for selected settings. Additionally, we calculate a cumulative metric called the Machine Learning cumulative performance score (*MLcps*) [88]. The *MLcps* combines the precomputed performance metrics into a single metric that encapsulates the core aspects of all the metrics. The value of *MLcps* is equal to the area of the polygon created by the metrics in a radar plot. We used the *MLcps Python* package version 0.0.6. The *MLcps* metric was originally designed for comparing and identifying the best-performing ML algorithm. However, herein, we utilize *MLcps* to compare the performance of the same model for the different amounts of training data. As we have a fixed number of precalculated metrics (accuracy, sensitivity, specificity, F1 score, and precision), we define *MLcps%* as


(1) \begin{eqnarray*}
{\it MLcps\%}=\frac{{\it MLcps}}{{\it MLcps}_{max}}\times 100,
\end{eqnarray*}

where *MLcps* is the area of the pentagon in the radar plot, and ${\it MLcps}_{max}$ is the area of the pentagon when all 5 metrics are 100%.

Furthermore, a permutation test was conducted to assess the statistical significance of the model’s performance. By randomly shuffling the class labels 1,000 times and retraining the model on each shuffled set of data, a distribution of accuracy values under chance conditions was obtained for all the experimental settings.

## Results

**Figure 2:**
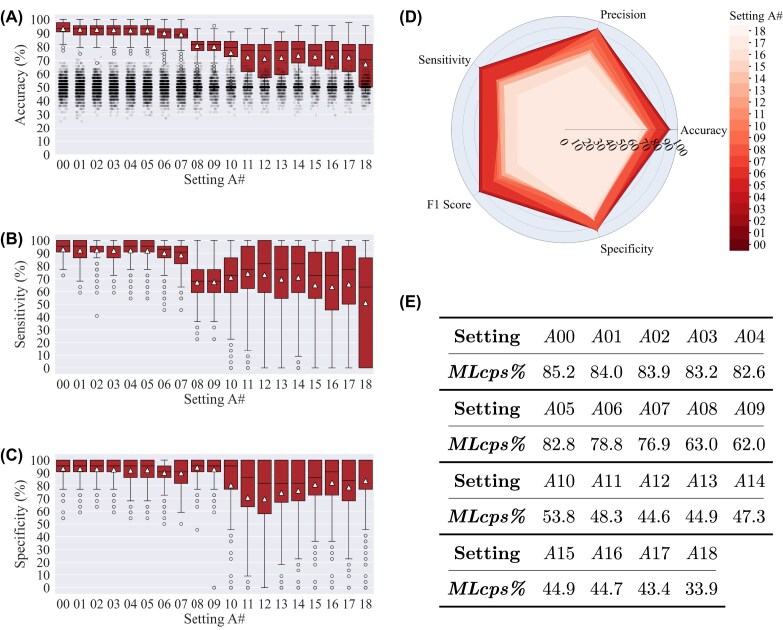
Classification results of settings *A*00 to *A*18: (A) accuracy, (B) sensitivity, (C) specificity, (D) radar plot of 5 classification metrics for different setting #s, and (E) *MLcps%* (a cumulative performance score) in percentages for each setting #. The triangle marker indicates the mean value, and the whiskers represent $1.5\times IQR$. In each setting # (i.e., *A*00, *A*01, $\ldots$, *A*18), the training set size is sequentially reduced by approximately 10% relative to the previous setting, as detailed in Supplementary Table S1. In (A), the jittered points represent the results of 1,000 permutation tests for each setting #. Average accuracy starts at 93.2% ± 4.3% for *A*00, where training includes 200 MRIs, and decreases to 89.2% ± 6.4% in *A*07, with 72 training data, after which it drops with further reductions in training data. A similar trend is observed in the *MLcps%*.

In this section, we present and analyze the results of the experiments to evaluate the performance of the HeteroMRI method. The results are reported separately for the experimental settings $A, B, C,$ and *D*. Furthermore, the insights into limited data scenarios gained through the experiments are discussed afterward. The classification metrics are provided in boxplot and radar plot formats in Figs. 2, 5, 6, and 7. In all boxplots, the triangle marker indicates the mean value, and the whiskers represent $1.5\times IQR$, where *IQR* is the interquartile range. In settings $A, B,$ and *D*, the boxplots show the distribution of 500 values for each setting #, corresponding to 50 data shuffles that each has been run 10 times. By “setting #,” we mean, for example, $A00, A01, \ldots , A18$. In setting *C*, the boxplot shows the distribution of 5,000 accuracy values for each setting # since there are 10 cases with different protocols chosen as the test set, as explained in “Experimental settings.” The results of permutation tests are plotted as jittered points in the accuracy plots in Figs. 2A, 5A, 6A, and 7A. For settings $A, B$, and *D*, there are 1,000 permutations, while for setting *C*, there are 10,000 permutations since there are 10 cases with different protocols chosen as the test set.

**Figure 3:**
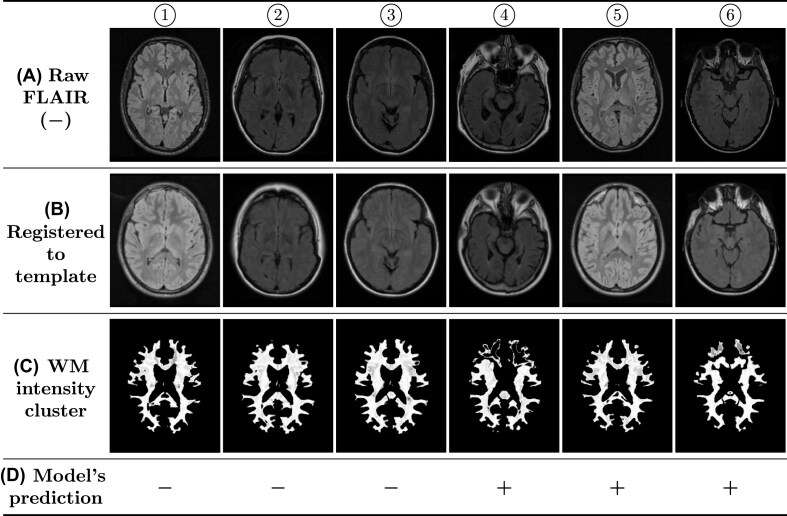
Example of 6 MRIs (① to ⑥) *without* WM abnormality. (A) Raw FLAIR images, (B) the MRI 3 times registered to the MNI template (the middle slice shown), (C) the obtained intensity clusters (thresholded) used for testing the model, and (D) the prediction of the model in setting *A*00 for the presence of WM abnormalities ($\boldsymbol {+}$: with WM abnormality, $\boldsymbol {-}$: without WM abnormality). This figure shows 3 cases of true negative and 3 cases of false positive. The WM intensity cluster slice corresponds to the registered MRI slice, but not exactly to the raw MRI slice, due to deformations from nonlinear registrations. MRI source: ①, ⑤ [83]; ②, ③, ④ [81]; and ⑥ [80].

**Figure 4:**
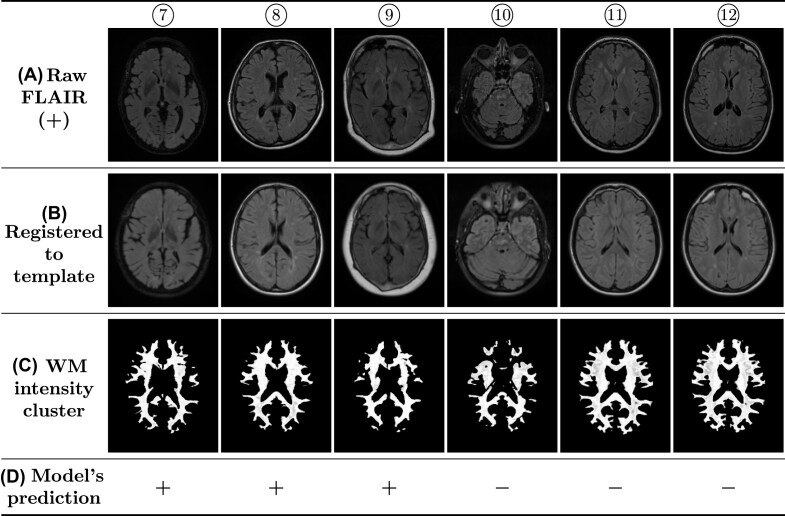
Example of 6 MRIs (⑦ to ⑫) *with* WM abnormality. (A) raw FLAIR images, (B) the MRI 3 times registered to the MNI template (the middle slice shown), (C) the obtained intensity clusters (thresholded) used for testing the model, and (D) the prediction of the model in setting *A*00 for the presence of WM abnormalities ($\boldsymbol {+}$: with WM abnormality, $\boldsymbol {-}$: without WM abnormality). This figure shows 3 cases of true positive and 3 cases of false negative. The WM intensity cluster slice corresponds to the registered MRI slice, but not exactly to the raw MRI slice, due to deformations from nonlinear registrations. MRI source: ⑦,⑩ [82]; ⑧[79]; ⑨ [81]; ⑪,⑫ [75].

**Figure 5:**
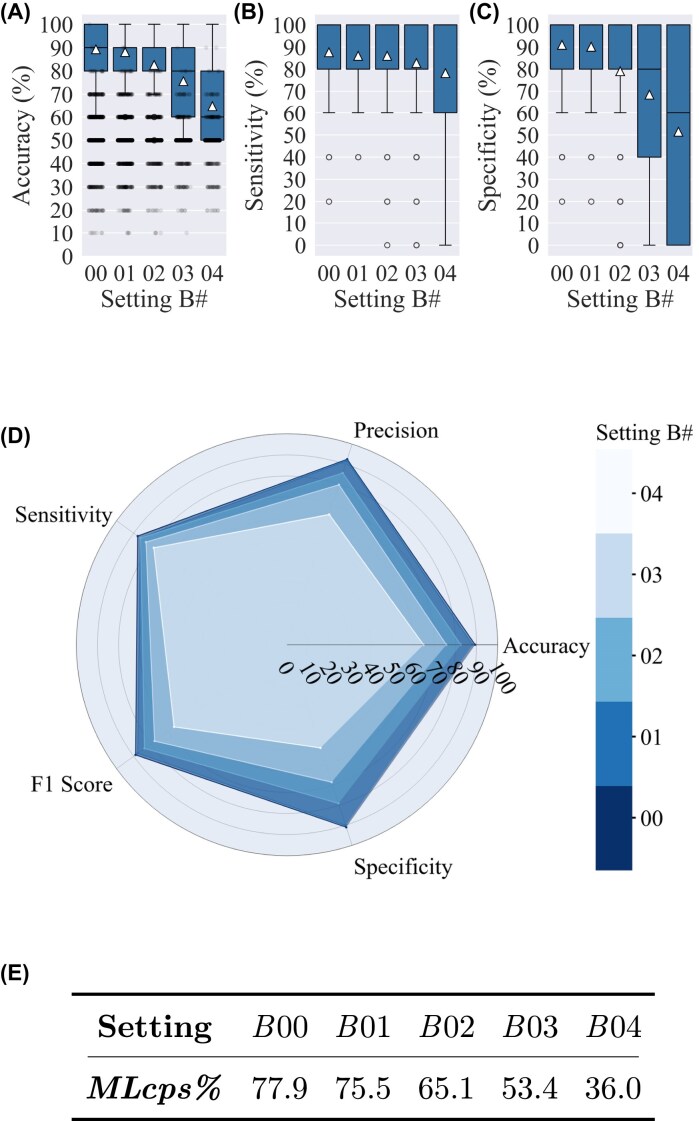
Classification results of settings *B*00 to *B*04: (A) accuracy, (B) sensitivity, (C) specificity, (D) radar plot of 5 classification metrics for different setting #s, and (E) *MLcps%* (a cumulative performance score) in percentages for each setting #. The triangle marker indicates the mean value, and the whiskers represent $1.5\times IQR$. In each setting # (i.e., *B*00, *B*01, $\ldots$, *B*04), the training set size is sequentially reduced relative to the previous setting, as detailed in Supplementary Table S2. In (A), the jittered points represent the results of 1,000 permutation tests for each setting #. In setting *B*, an equal number from 10 different MRI protocols is used for the model. Average accuracy starts at 89.3% ± 9.1% for *B*00, where training includes 60 MRIs, and decreases to 82.5% ± 14.2% in *B*02, with 40 training data, after which it decreases gradually with further reductions in training data. A similar trend is observed in the *MLcps%*.

**Figure 6:**
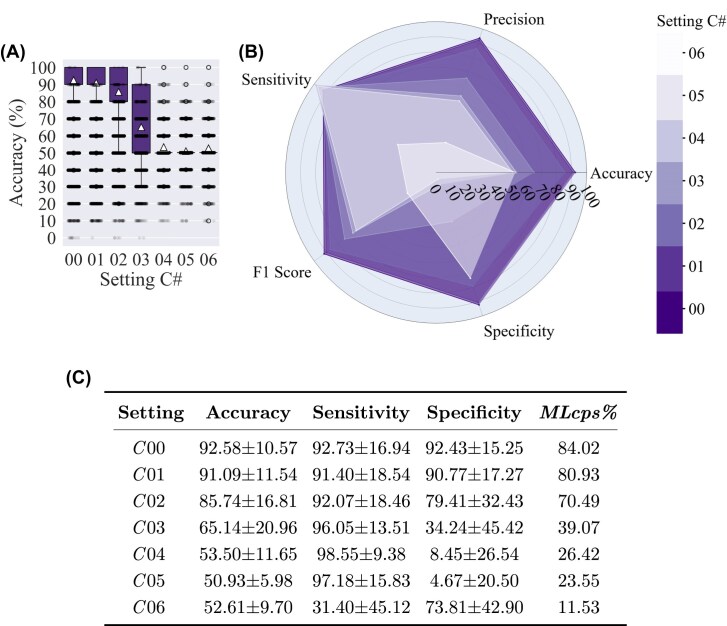
Classification results of settings *C*00 to *C*06: (A) accuracy, (B) radar plot of 5 classification metrics for different setting #s, and (C) four metric values for each setting # (in percentages, reported as mean ± standard deviation). The triangle marker indicates the mean value, and the whiskers represent $1.5\times IQR$. In each setting # (i.e.,*C*00, *C*01, $\ldots$, *C*06), the training set size is sequentially reduced relative to the previous setting, as detailed in Supplementary Table S3. In (A), the jittered points represent the results of 1,0000 permutation tests for each setting #. In setting *C*, the MRI protocols of the test set are unseen by the model during training. Average accuracy starts at 92.6% ± 10.6% for *C*00, where training includes 64 MRIs, and reduces to 85.7% ± 16.8% in *C*02, with 46 training data, after which it drops sharply with further reductions in training data. A similar trend is observed in the *MLcps%*.

**Figure 7:**
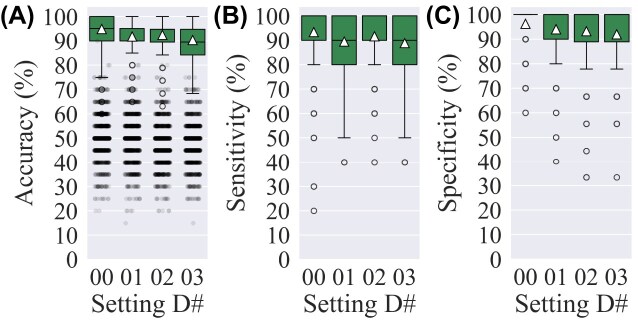
Classification results of settings *D*00 to *D*03: (A) accuracy, (B) sensitivity, and (C) specificity. The triangle marker indicates the mean value, and the whiskers represent $1.5\times IQR$. In each setting # (i.e., *D*00, *D*01, $\ldots$, *D*03), the number of MRI protocols is sequentially increased relative to the previous setting while maintaining equal training set size (82 MRIs), as detailed in Supplementary Table S4. In (A), the jittered points represent the results of 1,000 permutation tests for each setting #. Average accuracy starts at 94.8% ± 6.8% for *D*00, where training data include 4 different MRI protocols, and ends at 90.3% ± 6.3% for *D*03, with 10 MRI protocols in training data.

The radar plots illustrate the metric values for all the setting #s simultaneously, allowing us to perceive the effect of reducing training data size on each metric. In addition, the radar plots are utilized for calculating the *MLcps* values using Eq. (1). The *MLcps%* values for the settings $A, B,$ and *C* are reported in Figs. 2E, 5E, and 6C. The radar plots are plotted using the mean metric values; therefore, no standard deviation is reported for the *MLcps%* values. Evaluation of the presented approach across various experimental settings provides several key insights, offering a detailed understanding of its performance and challenges. The results of each experimental setting are presented below.

### Setting *A*

As shown in Fig. 2, setting *A*00, in which the highest number of MRIs (174 training + 26 validation + 44 test) was included, demonstrates an average accuracy of 93.2% ± 4.3% in the classification of MRIs. The training and testing data in this setting include, in total, 32 different MRI protocols. This underscores the model’s adaptability and robustness in handling a diverse range of imaging protocols. Notably, the effect of reducing the training data on the model’s performance is inspected here. In *A*07, where the training data (including validation data) are reduced to 36% of *A*00, the accuracy is 89.2% ± 6.4%. Beginning from setting *A*08, where the training data are 25% of *A*00, the average accuracy and sensitivity have a sharp decrease. With a much further decrease in the training data (e.g., *A*18 with only 2 MRIs as training), the accuracy and sensitivity gradually decrease to low values as expected, while specificity tends to remain relatively high. In relatively limited data settings (particularly from *A*11 to *A*18), the boxplots indicate that accuracy values have significant fluctuations across the range.

Sample raw MRIs, the registered MRIs, the obtained WM intensity clusters, and the model’s label predictions are provided here for a better insight into the data used for testing the model. Figures 3 and 4 depict samples of MRIs without and with WM abnormality, respectively. Below each raw MRI, the 3-times registered MRI and the obtained WM intensity cluster (thresholded) are illustrated. Additionally, the model’s prediction for the presence of WM abnormalities in setting *A*00 is reported. Each figure includes 3 true predictions and 3 false predictions. It is important to note that the shown WM intensity cluster and the registered MRI slice show the same location of the brain (the middle slice). However, these 2 slices do not directly correspond to the raw MRI slice. This is because the nonlinear registration process results in the deformation of the brain. As a result, finding the exact corresponding slices in the raw MRI and the registered one is impractical. Here, the middle slice of the registered MRI is illustrated. The raw MRI slices shown here are selected based on their visual similarity to the corresponding registered slices. The slices of the thresholded WM cluster are presented here solely to illustrate the input provided to the DL model for label prediction.

By investigating the possible reasons for the false predictions in setting *A*00, certain aspects became apparent. In some MRIs, the registration process has not been successful in correctly aligning the brain to the brain template. In such cases, the brain regions are not located in the correct locations after the 3-times registration in the MRI preprocessing phase. Therefore, in the WM extraction step, wrong parts of the brain are extracted as WM. This mostly results in a false prediction by the model, especially if the MRI is, in fact, free of WM abnormalities. Examples of incorrect registration are MRIs ④, ⑤, ⑥, ⑩, and ⑫ shown in Figs. 3 and 4. These registration errors are identified by comparing the slices of the registered MRIs with the corresponding slice from the MNI template slice shown in Fig. 1B. In both cases, the middle slice of the MRI is displayed. In an accurate registration, the brain regions in the registered slice align approximately with those in the template. We noticed that some of the MRIs with incorrect registration are 2D MRIs (such as MRIs ④ and ⑫). The low number of slices in 2D MRIs appears to be a contributing factor to registration problems in some cases. Considering that MRI registration is usually a challenging problem, other factors are also likely to contribute to incorrect registrations; however, they have not been investigated in this study. We refrained from excluding the MRIs with erroneous registration from the study, as our goal was to evaluate HeteroMRI as a fully automatic method without user interference.

### Setting *B*

The setting *B*, which uses an equal number of MRIs from each MRI protocol, is designed to make the prediction task more challenging for the model. Setting *B*00 has an average accuracy of 89.3% ± 9.1%, as shown in Fig. 5. In terms of the amount of training data, setting *A*07 is the closest match to *B*00. While *B*00 includes 50 training and 10 validation data, *A*07 has a comparable setup with 52 training and 20 validation data. However, setting *B* is a more challenging scenario than setting *A* because the model sees an equal number of MRIs from each MRI protocol without being biased by a higher number of images from some protocols. Despite this challenge, the accuracy of *B*00 is almost equal to that of *A*07, which is 89.2% ± 6.4%. This shows the high independence of the presented MRI classification approach on the acquisition protocol of the FLAIR images. With a further decrease in the training data, in settings *B*01 to *B*04, all metrics show a gradual decrease in value.

### Setting *C*

The evaluation of setting *C* is of more importance since it is very close to the real-world use of such a model, as it evaluates the generalizability of HeteroMRI to MRIs from unseen protocols. In this setting, the MRI protocols of the test set are not present in the training data. It resembles a situation in which a clinical center has heterogeneous MRI data and wants to train a classification model with them. Then the model is supposed to classify new MRIs brought by new patients from other centers, acquired most probably with MRI protocols different from those in the training data.

It is noteworthy that in setting *C*, only 3D MRIs (with 192 or more slices) are included in training and test data, as discussed in the next paragraph. Setting *C*00, as reported in Fig. 6, shows an average accuracy of 92.6% ± 10.6% with 64 MRIs used for training (including validation), which proves the generalizability of the trained model to unseen MRI scanners and protocols. By reducing the data to 46, in *C*02, the model shows an accuracy of 85.7% ± 16.8%. By further decreasing the data to 28 or less, the model’s accuracy drops to the chance level (around 50%) in *C*04 to *C*06.

Initially, we used both 3D and 2D MRIs for setting *C*. However, the model showed a lack of robustness when its generalizability to different protocols was evaluated (i.e., by varying the protocols considered as test data). As we suspected the 2D MRIs (with 70 or fewer slices) as a source of the model’s poor performance, we redesigned the setting *C* to include *only* 3D MRIs. As a result, the performance significantly improved, as reported in the results for setting *C*. It is important to emphasize that the test data were not fixed for the 2 versions of setting *C*, as the model was tested with multiple data shuffles for each setting, with the data being split again in each shuffle. However, from a certain perspective, the decision to exclude 2D MRIs could be interpreted as a form of overfitting to the data characteristics. Additionally, to further investigate the role of 2D MRIs, we also designed a separate setting with *only* 2D MRIs. In this case, the model showed notably poor robustness.

### Setting *D*

Setting *D*, in which the number of MRI protocols among the data was increased in each setting # while maintaining the same data size (82 MRIs for training), shows the slight negative effect of having higher numbers of protocols, as observed by the overall decrease in accuracy, sensitivity, and specificity (Fig. 7). In *D*00 with 4 protocols, the accuracy is 94.80% ± 6.79% while in *D*03 with 10 protocols, the model classifies the MRIs with an accuracy of 90.31% ± 6.36%.

### Limited data scenarios

A more detailed examination of the impact of reducing training data is presented in Table 2. By comparing settings $A, B$, and *C*, a rough correlation can be concluded between the number of MRIs in the training data and the performance of the model for the classification task of this study, regardless of the experimental setting. When the training data (including validation) consist of 60 to 72 MRIs (as in *A*07 and *B*00), an accuracy of 89% is expected. By having 40 to 46 MRIs in the training set (as in $A09, B02,$ and *C*02), the accuracy falls roughly within the range of 80% to 86%. Further reducing the training data to the 20 to 36 range (as in $A12, B03,$ and *C*03) is associated with a rough accuracy of 76% or less and an F1 score of 78% or less. In this last scenario, the model’s performance cannot be considered fully reliable, as the results show a high degree of variability across different runs.

**Table 2.** Average performance results of selected experimental settings (in percentages, reported as mean ± standard deviation). The table shows the effect of reducing training data on the model’s performance in settings $A, B,$ and *C*. For these settings, 3 cases are reported, respectively: (i) with the highest number of training data, (ii) with the borderline number of training data after which the performance drops, and (iii) with the number of training data that results in relatively low performance. Setting *D* shows the effect of increasing the number of MRI protocols for the same number of MRI data. AUROC: the area under the receiver operating characteristic curve.

Setting	Data size^1^		Protocols^2^		Accuracy	Sensitivity	Specificity	F1 score	AUROC
	Train$^{\dagger }$	Test	Train$^{\dagger }$	Test					
*A*00	200	44	31	14	93.24±4.29	93.05±6.34	93.44±7.38	93.24±4.25	96.76±3.09
*A*07	72	44	17±1	14	89.16±6.39	88.41±11.17	89.91±9.48	88.87±7.00	94.89±4.18
*A*09	42	44	9±1	14	80.17±6.69	67.59±13.29	92.74±14.78	76.83±8.55	91.18±4.30
*A*12	20	44	6±1	14	71.41±13.76	73.14±30.01	69.67±34.80	68.35±23.32	91.91±3.71
*B*00	60	10	10	10	89.32±9.14	87.64±14.31	91.00±14.25	88.88±9.75	95.11±7.24
*B*02	40	10	10	10	82.54±14.17	86.00±15.46	79.08±30.20	83.65±12.09	91.98±8.68
*B*03	30	10	10	10	75.64±16.36	82.76±19.25	68.52±38.97	77.71±14.63	90.26±8.69
*C*00	64	10	8	2	92.58±10.57	92.73±16.94	92.43±15.25	91.91±13.25	99.07±1.06
*C*02	46	10	8	2	85.74±16.81	92.07±18.46	79.41±32.43	86.80±16.27	97.43±2.12
*C*03	36	10	8	2	65.14±20.96	96.05±13.51	34.24±45.42	75.09±14.84	93.34±3.33
*D*00	82	20	4	4	94.80±6.79	93.38±11.91	96.22±7.69	94.38±8.27	99.38±1.86
*D*03	82	20	10	10	90.30±6.33	88.82±11.42	91.95±10.28	90.36±6.85	96.11±5.03

^1^Number of MRIs used in the training and test sets.

^2^Number of MRI protocols present in the training and test data.

$^{\dagger }$ Including the validation data.

### Comparison with related methods

To further evaluate the performance of HeteroMRI, we applied it on a hold-out set of data and compared the performance with 3 lesion segmentation methods: DeepWMH [53], WHITE-Net [54], and the Lesion Prediction Algorithm from the Lesion Segmentation Tool (LST-LPA) [55]. These methods are not designed for direct binary classification of MRIs. To enable a comparison, we postprocessed their segmentation outputs by binarizing the masks through 3D connected components analysis (using the ConnectedComponentImageFilter class from the SimpleITK [65] *Python* library): if at least 1 WM lesion consisting of *5* or more 3D-connected voxels was detected, the corresponding MRI scan was labeled as positive. The comparison was conducted using datasets 10 and 11, introduced in “Datasets.” The test set includes 20 MRIs in total, with balanced labels and images acquired from 9 different MRI protocols. These datasets serve as holdout sets for HeteroMRI and were not used during training or testing in any of the experimental settings. For HeteroMRI, we employed the 500 models trained in setting *A*00. The final prediction label for each test scan was determined by majority vote across these models. The DeepWMH, WHITE-Net, and LST-LPA are pretrained, ready-to-use models that do not require retraining. All of the methods require only a FLAIR image per subject. Table 3 reports the accuracy, sensitivity, and specificity for all methods. Based on the results, HeteroMRI outperforms DeepWMH, WHITE-Net, and LST-LPA in accuracy and specificity, while the other methods have a higher sensitivity.

**Table 3.** Performance of HeteroMRI (setting *A*00) as a binary classifier for detecting brain MRIs with WM abnormalities in a hold-out set, in comparison to 3 other related methods

Method	Accuracy	Sensitivity	Specificity
**HeteroMRI** (This work)	**70%**	90%	**50%**
**DeepWMH** [53]	55%	**100%**	10%
**WHITE-Net** [54]	55%	**100%**	10%
**LST-LPA** [55]	60%	**100%**	20%

## Discussion

This study presented HeteroMRI, a DL framework designed for robust binary classification of WM abnormalities across heterogeneous FLAIR MRI datasets. The results show that HeteroMRI is capable of performing the classification while being highly robust to scanner and protocol variabilities. The method is also adaptable to standardized MRI datasets acquired using a uniform scanner and protocol. The experimental results across settings *A*–*D* and the limited-data scenarios offer several insights into the method’s robustness, generalizability, and limitations under varying data constraints.

In setting *A*, where training data included all available protocols, HeteroMRI achieved high classification performance. Even by reducing the training data to 72 MRIs, the accuracy remained at 89±6%. This suggests that the model learns effectively from the heterogeneous data, with only a gradual decline in performance as data scarcity increases. In setting *B*, where each protocol was equally represented, HeteroMRI maintained comparable accuracy, confirming that the model is highly independent of protocols. In setting *C*, which tested generalization to unseen MRI protocols, HeteroMRI continued to perform robustly, provided the training data included a sufficient number of high-resolution (3D) MRIs. This emphasizes the importance of data quality in achieving protocol-invariant performance. Finally, setting *D* showed the impact of increasing protocol diversity while keeping data volume fixed. A gradual decrease in accuracy was observed with a growing number of MRI protocols, decreasing from 94.8% in D00 (4 protocols) to 90.3% in D03 (10 protocols).

In comparison of the performance on a hold-out set with 3 segmentation-based methods—DeepWMH [53], WHITE-Net [54], and LST-LPA [55]— HeteroMRI outperformed all 3 methods in terms of both accuracy and specificity, showing a more reliable ability to avoid false positives despite the fact that DeepWMH and WHITE-Net claim to be robust to scanner and protocol variability. This comparison was conducted on a highly heterogeneous hold-out set comprising 20 MRIs from 9 different acquisition protocols. Moreover, this comparison result is obtained in a condition in favor of the segmentation methods since we applied a threshold requiring a minimum of five 3D-connected voxels for determining the presence of a lesion in the segmentation outputs. This conservative criterion helps filter out small, potentially false predictions and helps segmentation-based models by reducing false positives. Nevertheless, the segmentation-based models demonstrated higher sensitivity, which can be attributed to their design: these methods are tailored to detect even subtle or small lesion patterns, making them more prone to false positives in borderline cases. These findings generally support HeteroMRI as a method that is highly independent of scanner and protocol variability, generalizes relatively well to unseen protocols, and retains performance under data-limited conditions to some extent.

Despite the promising results of the presented method, it has several limitations to be considered. HeteroMRI faces a challenge with the registration problem with certain MRIs, leading to a false prediction. Registration problems are a well-known issue in MRI analysis. For addressing this challenge with HeteroMRI, a more elaborate registration strategy may reduce the misalignments, or an automatic method may be developed to warn about significant deformations during registration, which can be a sign of wrong registration. These improvements are future work directions for further improving the method. Notably, 2D MRIs were identified as one of the factors that can contribute to registration issues, strongly suggesting the use of 3D MRIs with HeteroMRI. Further factors that cause an incorrect registration were not investigated in this study. Another limitation of HeteroMRI is the high GPU memory requirement, which is not easily available in every computing server. For each specific use case, one can evaluate the method’s performance by downscaling the dimension of input images (and therefore reducing the dimensionality of the CNN) to decrease the required GPU memory. Additionally, the design of HeteroMRI can be upgraded to a multi-channel format, enabling the integration of multiple MRI sequences per subject for enhanced analysis. Moreover, the performance of the method could be studied in case of unbalanced training data. The current classification task of HeteroMRI may be less interesting to apply in clinical practice compared to, for example, segmentation or volumetric measurement methods. However, this study is supposed to present the HeteroMRI method and evaluate its performance on heterogeneous data. In future work, the methodology is planned to be employed for the disease-specific classification of MRIs based on WM abnormalities by learning from subtle lesion patterns.

## Conclusion

In this study, we introduced HeteroMRI, a novel approach for robust classification of brain MRIs based on WM abnormalities, specifically designed to handle heterogeneous MRI data acquired from diverse scanners and acquisition protocols. HeteroMRI achieved high accuracy in detecting MRIs with WM abnormalities, even in scenarios with relatively limited data. Furthermore, the method proved to be relatively more generalizable to unseen MRI protocols compared to segmentation-based methods. There is room for enhancing HeteroMRI’s performance by improving the registration accuracy or preventing wrong predictions by detecting the erroneous registrations automatically. Our future research will focus on applying this approach to differentiate between unspecific and disease-associated WM lesions, as well as to classify rare demyelinating diseases against their differential diagnoses.

## Availability of Supporting Source Code and Requirements

Project name: HeteroMRI v1.0

Project homepage: https://github.com/ul-mds/HeteroMRI

Operating system(s): Linux-based OS (Ubuntu recommended)

Programming language: Python

Other requirements: TensorFlow v2.x, NVIDIA GPU with CUDA support

License: GNU GPL version 3

RRID:SCR_027298

## Abbreviations

AI: artificial intelligence; ANTs: Advanced Normalization Tools; AUROC: area under the receiver operating characteristic curve; BTH: Baghdad Teaching Hospital; CNN: convolutional neural network; CSF: cerebrospinal fluid; CT: computed tomography; DL: deep learning; FCM: fuzzy C-means; FLAIR: fluid-attenuated inversion recovery; GM: gray matter; ICBM: International Consortium for Brain Mapping; MI: mutual information; ML: machine learning; *MLcps*: machine learning cumulative performance score; MNI: Montreal Neurological Institute; MRI: magnetic resonance imaging; MS: multiple sclerosis; NifTI: Neuroimaging Informatics Technology Initiative; PET: positron emission tomography; ReLU: rectified linear unit; RFCM: robust fuzzy C-means; TE: echo time; TI: inversion time; TR: repetition time; WM: white matter; 2D: 2-dimensional; 3D: 3-dimensional.

## Additional Files

**Supplementary Table S1**. Number of MRIs used for training (Tr), validation (V), and test (T) sets from each *dataset* in experimental settings *A*00 to *A*18.

**Supplementary Table S2**. Number of MRIs used for training (Tr), validation (V), and test (T) sets from each MRI acquisition *protocol* in experimental settings *B*00 to *B*04.

**Supplementary Table S3**. Number of MRIs used for training (Tr), validation (V), and test (T) sets from each MRI acquisition *protocol* in experimental settings *C*00 to *C*06. The two protocols in boldface are used only as test data. These two protocols are changed in 10 different cases.

**Supplementary Table S4**. Number of MRIs used for training (Tr), validation (V), and test (T) sets from each MRI acquisition *protocol* in experimental settings *D*00 to *D*03.

**Supplementary Fig. S1**. Mutual information metric between each MRI and the MNI template after each registration. The × marker indicates the mean value, and the whiskers represent 1.5 × *IQR*.

**Supplementary Fig. S2**. Average required time for preprocessing and intensity clustering of five sample MRI dimensions.

**Supplementary Fig. S3**. Average required time for training the CNN model of each experimental setting.

**Supplementary Fig. S4**. The WM intensity cluster of a sample MRI (a) before and (b) after applying a threshold value of 0.5. The normalized histograms of their 99% upper percentile are shown in (c) and (d), respectively.

giaf092_HeteroMRI_Supp_Material

giaf092_Authors_Response_To_Reviewer_Comments_Original_Submission

giaf092_Authors_Response_To_Reviewer_Comments_Revision_1

giaf092_Authors_Response_To_Reviewer_Comments_Revision_2

giaf092_GIGA-D-24-00230_Original_Submission

giaf092_GIGA-D-24-00230_Revision_1

giaf092_GIGA-D-24-00230_Revision_2

giaf092_GIGA-D-24-00230_Revision_3

giaf092_Reviewer_1_Report_Original_Submission Pravesh Parekh -- 8/17/2024

giaf092_Reviewer_1_Report_Revision_1 Pravesh Parekh -- 12/24/2024

giaf092_Reviewer_1_Report_Revision_2 Pravesh Parekh -- 7/2/2025

giaf092_Reviewer_2_Report_Original_Submission Chiara Marzi -- 8/30/2024

giaf092_Reviewer_2_Report_Revision_1 Chiara Marzi -- 12/16/2024

## Data Availability

All the datasets used in this study are publicly available or are accessible upon request to the respective dataset providers as referenced in Table 1. Snapshots of our code and other data further supporting this work are archived in Software Heritage [89]. In addition, machine learning algorithms have been deposited in the DOME Registry [90].
