# Supplementary material for: HeteroMRI: Robust white matter abnormality classification across multi-scanner MRI data
Source: Gigascience. 2025 Aug 21;14:giaf092. doi: 10.1093/gigascience/giaf092 (PMC12371411; doi:10.1093/gigascience/giaf092)
Supplement: giaf092_HeteroMRI_Supp_Material [file giaf092_heteromri_supp_material.pdf]

Supplementary Material

This file contains the supplementary material for the following article:

Title: HeteroMRI: Robust white matter abnormality classification across multi-scanner MRI data
Authors: Masoud Abedi, Navid Shekarchizadeh, Christa-Caroline Bergner, Pierre-Louis Bazin, Nico Scherf, Julia Lier, Wolfgang Köhler, and Toralf Kirsten
Published in: GigaScience, 2025.
DOI: https://doi.org/10.1093/gigascience/giaf092

Supplementary Table S1: Number of MRIs used for training (Tr), validation (V), and test (T) sets from each *dataset* in experimental settings $A00$ to $A18$

**Dataset**		**Set**	**Setting $A\#$**																		
**Label***	**Name**		**00**	**01**	**02**	**03**	**04**	**05**	**06**	**07**	**08**	**09**	**10**	**11**	**12**	**13**	**14**	**15**	**16**	**17**	**18**
+	ISBI	Tr	8	7	6	5	4	3	2	1	0	0	0	0	0	0	0	0	0	0	0
		V	1	1	1	1	1	1	1	1	0	0	0	0	0	0	0	0	0	0	0
		T	2	2	2	2	2	2	2	2	2	2	2	2	2	2	2	2	2	2	2
	UMCL	Tr	8	7	6	5	4	3	2	1	0	0	0	0	0	0	0	0	0	0	0
		V	1	1	1	1	1	1	1	1	0	0	0	0	0	0	0	0	0	0	0
		T	2	2	2	2	2	2	2	2	2	2	2	2	2	2	2	2	2	2	2
	MSSEG	Tr	8	7	6	5	4	3	2	1	0	0	0	0	0	0	0	0	0	0	0
		V	1	1	1	1	1	1	1	1	0	0	0	0	0	0	0	0	0	0	0
		T	2	2	2	2	2	2	2	2	2	2	2	2	2	2	2	2	2	2	2
	MSSEG-2	Tr	8	7	6	5	4	3	2	1	0	0	0	0	0	0	0	0	0	0	0
		V	1	1	1	1	1	1	1	1	0	0	0	0	0	0	0	0	0	0	0
		T	2	2	2	2	2	2	2	2	2	2	2	2	2	2	2	2	2	2	2
	BTH	Tr	6	5	4	3	2	1	0	0	0	0	0	0	0	0	0	0	0	0	0
		V	1	1	1	1	1	1	0	0	0	0	0	0	0	0	0	0	0	0	0
		T	2	2	2	2	2	2	2	2	2	2	2	2	2	2	2	2	2	2	2
	OASIS-3	Tr	10	9	8	7	6	5	4	3	2	1	0	0	0	0	0	0	0	0	0
		V	2	2	2	1	1	1	1	1	1	1	0	0	0	0	0	0	0	0	0
		T	2	2	2	2	2	2	2	2	2	2	2	2	2	2	2	2	2	2	2
	ADNI 3	Tr	39	35	31	28	26	23	21	19	17	14	12	9	7	6	5	4	3	2	1
		V	6	6	6	6	6	6	5	5	5	5	4	4	3	3	3	2	2	1	1
		T	10	10	10	10	10	10	10	10	10	10	10	10	10	10	10	10	10	10	10
−	ICBM	Tr	3	2	1	0	0	0	0	0	0	0	0	0	0	0	0	0	0	0	0
		V	1	1	1	0	0	0	0	0	0	0	0	0	0	0	0	0	0	0	0
		T	1	1	1	1	1	1	1	1	1	1	1	1	1	1	1	1	1	1	1
	OASIS-3	Tr	65	58	51	45	39	32	26	21	15	12	10	8	7	6	5	4	3	2	1
		V	9	9	9	9	9	9	8	8	5	5	3	3	3	3	3	2	2	1	1
		T	16	16	16	16	16	16	16	16	16	16	16	16	16	16	16	16	16	16	16
	CERMEP	Tr	19	17	15	13	11	9	7	5	4	3	2	1	0	0	0	0	0	0	0
		V	3	3	3	3	3	3	2	2	1	1	1	1	0	0	0	0	0	0	0
		T	5	5	5	5	5	5	5	5	5	5	5	5	5	5	5	5	5	5	5
+	Total	Tr	87	77	67	58	50	41	33	26	19	15	12	9	7	6	5	4	3	2	1
		V	13	13	13	12	12	12	10	10	6	6	4	4	3	3	3	2	2	1	1
		T	22	22	22	22	22	22	22	22	22	22	22	22	22	22	22	22	22	22	22
−	Total	Tr	87	77	67	58	50	41	33	26	19	15	12	9	7	6	5	4	3	2	1
		V	13	13	13	12	12	12	10	10	6	6	4	4	3	3	3	2	2	1	1
		T	22	22	22	22	22	22	22	22	22	22	22	22	22	22	22	22	22	22	22
**Total**			244	224	204	184	168	150	130	116	94	86	76	70	64	62	60	56	54	50	48

* With (+) and without (−) WM abnormality

Supplementary Table S2: Number of MRIs used for training (Tr), validation (V), and test (T) sets from each MRI acquisition *protocol* in experimental settings $B00$ to $B04$

**Protocol**		**Set**	**Setting**				
**Label***	**Name**		$B00$	$B01$	$B02$	$B03$	$B04$
+	Sie_Tri_30_Prot1	Tr	5	4	3	2	1
		V	1	1	1	1	1
		T	1	1	1	1	1
	Phi_Ing_30_NA	Tr	5	4	3	2	1
		V	1	1	1	1	1
		T	1	1	1	1	1
	Phi_NA_30_Prot1	Tr	5	4	3	2	1
		V	1	1	1	1	1
		T	1	1	1	1	1
	Sie_Aer_15_Prot1	Tr	5	4	3	2	1
		V	1	1	1	1	1
		T	1	1	1	1	1
	GeE_Dis_30_Prot3	Tr	5	4	3	2	1
		V	1	1	1	1	1
		T	1	1	1	1	1
−	Sie_Bio_30_Prot1	Tr	5	4	3	2	1
		V	1	1	1	1	1
		T	1	1	1	1	1
	Sie_Son_15_Prot1	Tr	5	4	3	2	1
		V	1	1	1	1	1
		T	1	1	1	1	1
	Sie_MaV_30_Prot2	Tr	5	4	3	2	1
		V	1	1	1	1	1
		T	1	1	1	1	1
	Sie_MaV_30_Prot1	Tr	5	4	3	2	1
		V	1	1	1	1	1
		T	1	1	1	1	1
	Sie_TrT_30_Prot2	Tr	5	4	3	2	1
		V	1	1	1	1	1
		T	1	1	1	1	1
+	Total	Tr	25	20	15	10	5
		V	5	5	5	5	5
		T	5	5	5	5	5
−	Total	Tr	25	20	15	10	5
		V	5	5	5	5	5
		T	5	5	5	5	5
**Total**			70	60	50	40	30

* With (+) and without (−) WM abnormality

Supplementary Table S3: Number of MRIs used for training (Tr), validation (V), and test (T) sets from each MRI acquisition *protocol* in experimental settings $C00$ to $C06$. The two protocols in boldface are used only as test data. These two protocols are changed in 10 different cases.

**Protocol**		**Set**	**Setting**						
**Label***	**Name**		$C00$	$C01$	$C02$	$C03$	$C04$	$C05$	$C06$
+	Phi_Ing_30_NA	Tr	7	6	5	4	3	2	1
		V	1	1	1	1	1	1	1
		T	0	0	0	0	0	0	0
	GeE_Dis_30_Prot3	Tr	7	6	5	4	3	2	1
		V	1	1	1	1	1	1	1
		T	0	0	0	0	0	0	0
	Sie_Pri_30_Prot1	Tr	6	5	4	3	2	1	0
		V	2	2	2	1	1	1	0
		T	0	0	0	0	0	0	0
	Sie_Ver_30_Prot1	Tr	6	5	4	3	2	1	0
		V	2	1	1	1	1	1	0
		T	0	0	0	0	0	0	0
	**Phi_Ing_30_Prot2**	Tr	0	0	0	0	0	0	0
		V	0	0	0	0	0	0	0
		T	5	5	5	5	5	5	5
−	Sie_MaV_30_Prot1	Tr	6	5	4	3	2	1	0
		V	1	1	1	1	1	1	0
		T	0	0	0	0	0	0	0
	Sie_Son_15_Prot1	Tr	20	17	14	11	8	5	2
		V	5	4	4	3	3	3	2
		T	0	0	0	0	0	0	0
	**Sie_TrT_30_Prot1**	Tr	0	0	0	0	0	0	0
		V	0	0	0	0	0	0	0
		T	5	5	5	5	5	5	5
+	Total	Tr	26	22	18	14	10	6	2
		V	6	5	5	4	4	4	2
		T	5	5	5	5	5	5	5
−	Total	Tr	26	22	18	14	10	6	2
		V	6	5	5	4	4	4	2
		T	5	5	5	5	5	5	5
**Total**			74	64	56	46	38	30	18

* With (+) and without (−) WM abnormality

Supplementary Table S4: Number of MRIs used for training (Tr), validation (V), and test (T) sets from each MRI acquisition *protocol* in experimental settings $D00$ to $D03$

**Protocol**		**Set**	**Setting**			
**Label***	**Name**		$D00$	$D01$	$D02$	$D03$
+	Sie_Tri_30_Prot1	Tr	21	12	9	8
		V	3	2	2	1
		T	6	3	2	2
	Phi_Ing_30_NA	Tr	15	12	9	8
		V	2	2	1	1
		T	4	3	3	2
	Phi_NA_30_Prot1	Tr	0	12	9	7
		V	0	1	1	1
		T	0	4	3	2
	Sie_Aer_15_Prot1	Tr	0	0	9	7
		V	0	0	1	1
		T	0	0	2	2
	GeE_Dis_30_Prot3	Tr	0	0	0	6
		V	0	0	0	1
		T	0	0	0	2
−	Sie_Bio_30_Prot1	Tr	21	124	11	9
		V	3	2	2	1
		T	6	3	2	3
	Sie_Son_15_Prot1	Tr	15	12	10	8
		V	2	2	1	1
		T	4	3	4	3
	Sie_MaV_30_Prot2	Tr	0	12	10	9
		V	0	1	1	1
		T	0	4	3	2
	Sie_MaV_30_Prot1	Tr	0	0	5	5
		V	0	0	1	1
		T	0	0	1	1
	Sie_TrT_30_Prot2	Tr	0	0	0	5
		V	0	0	0	1
		T	0	0	0	1
+	Total	Tr	36	36	36	36
		V	5	5	5	5
		T	10	10	10	10
−	Total	Tr	36	36	36	36
		V	5	5	5	5
		T	10	10	10	10
**Total**			102	102	102	102

* With (+) and without (−) WM abnormality

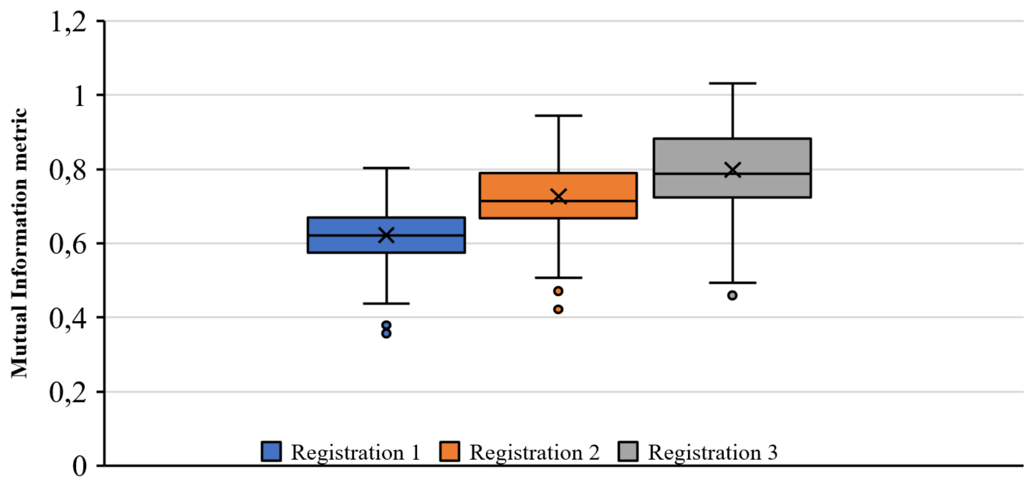

Supplementary Figure S1: Mutual information metric between each MRI and the MNI template after each registration. The × marker indicates the mean value, and the whiskers represent $1.5 \times IQR$.

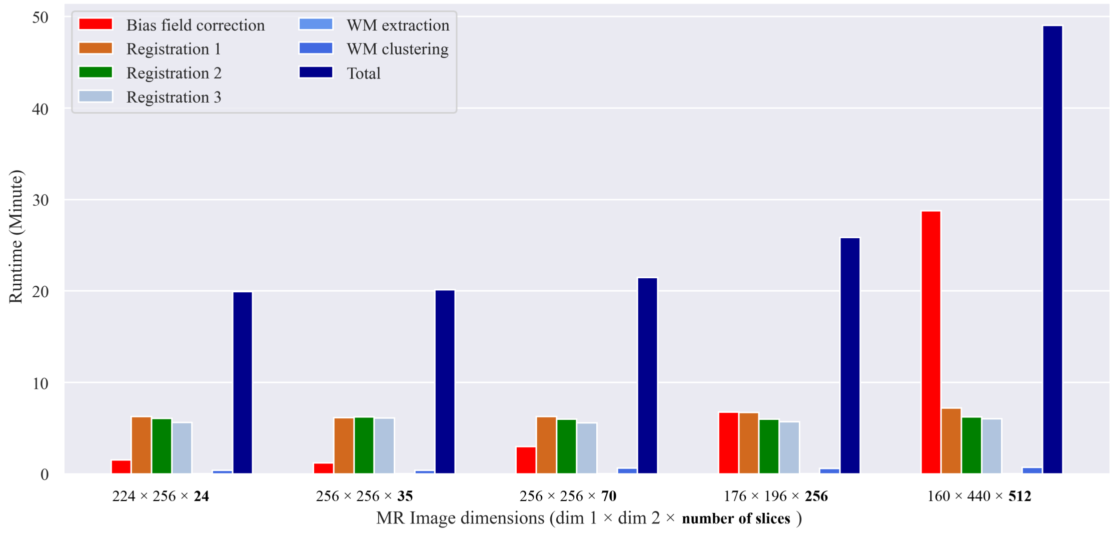

Supplementary Figure S2: Average required time for preprocessing and intensity clustering of five sample MRI dimensions.

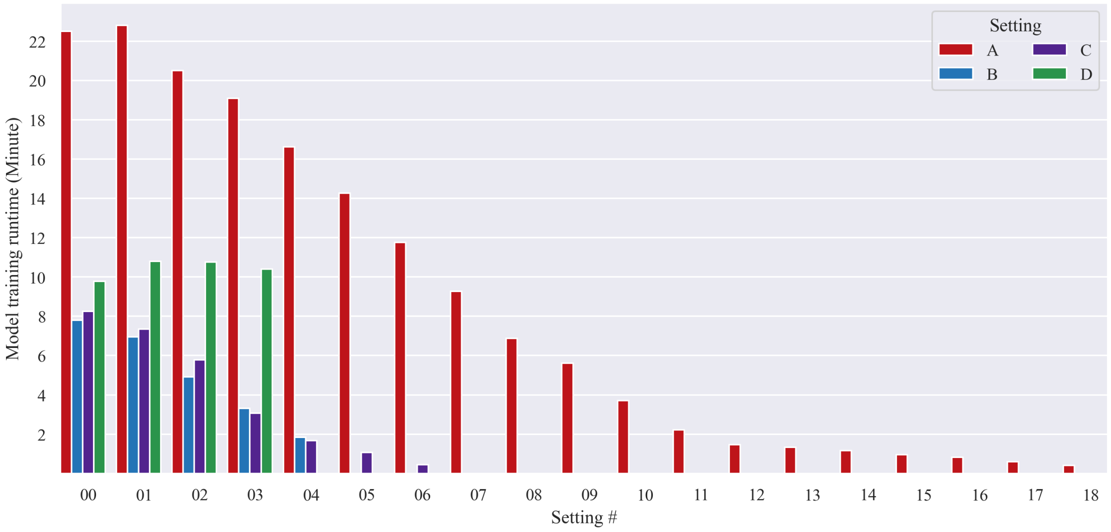

Supplementary Figure S3: Average required time for training the CNN model of each experimental setting.

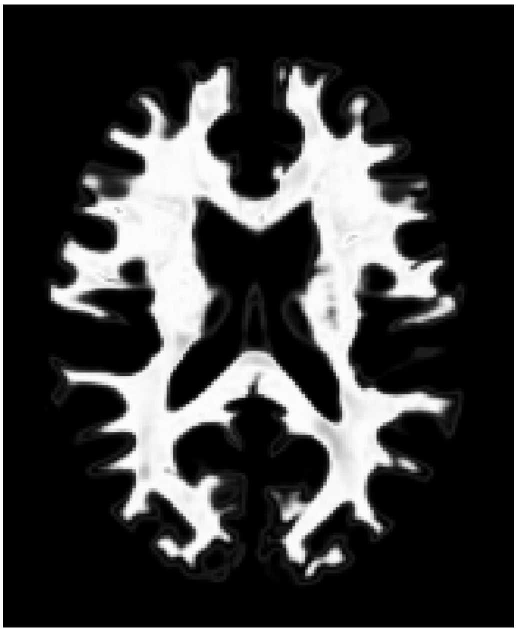

(a)

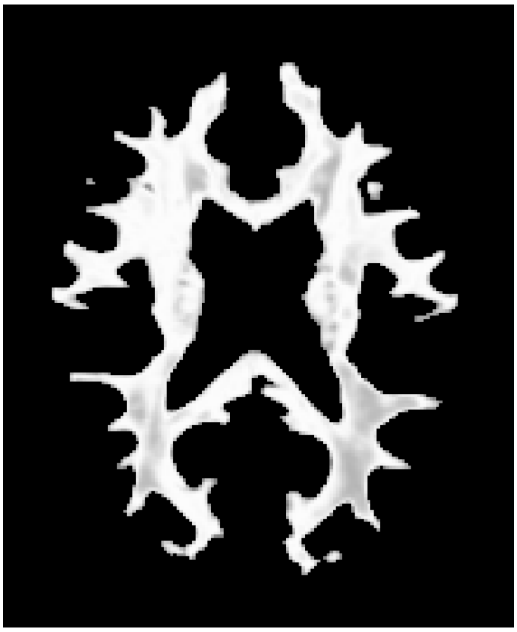

(b)

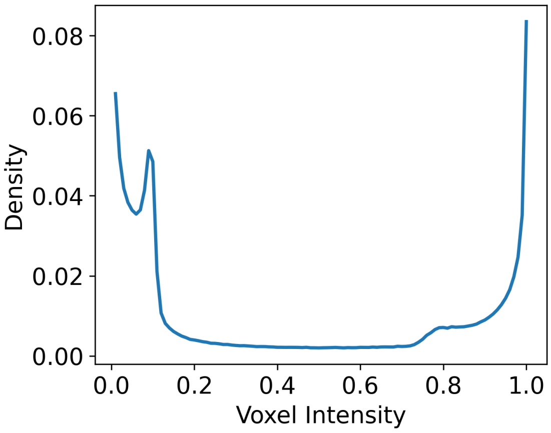

(c)

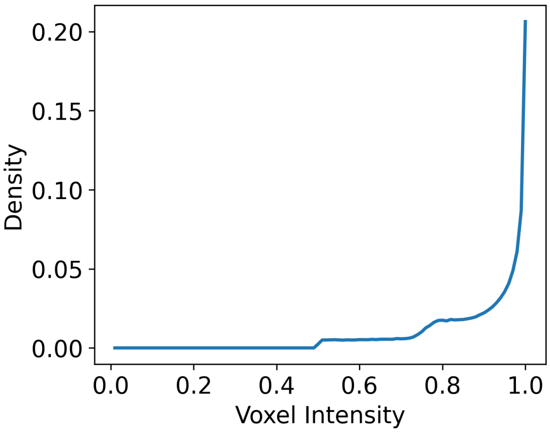

(d)

Supplementary Figure S4: The WM intensity cluster of a sample MRI (a) before and (b) after applying a threshold value of 0.5. The normalized histograms of their 99% upper percentile are shown in (c) and (d), respectively.
