# Supplementary material for: HeteroMRI: Robust white matter abnormality classification across multi-scanner MRI data
Source: Gigascience. 2025 Aug 21;14:giaf092. doi: 10.1093/gigascience/giaf092 (PMC12371411; doi:10.1093/gigascience/giaf092)
Supplement: giaf092_GIGA-D-24-00230_Original_Submission [file giaf092_giga-d-24-00230_original_submission.pdf]

GigaScience

HeteroMRI: A method for classification of multi-scanner and multi-protocol brain magnetic resonance images with deep learning

--Manuscript Draft--

**Manuscript Number:**	GIGA-D-24-00230
**Full Title:**	HeteroMRI: A method for classification of multi-scanner and multi-protocol brain magnetic resonance images with deep learning
**Article Type:**	Research
**Funding Information:**	Bundesministerium für Bildung und Forschung (100602109) — Not applicable<br>Bundesministerium für Gesundheit (ZMVI1-2520DAT94) — Mr. Masoud Abedi<br>Bundesministerium für Bildung und Forschung (ScaDS.AI) — Dr. Navid Shekarchizadeh
**Abstract:**	Background<br>A common application of Magnetic Resonance Imaging (MRI) is the analysis of white matter abnormalities in the human brain. Integrating machine learning techniques in MRI data analysis can enhance diagnostic processes. However, utilizing machine learning techniques is restricted when the available MRI data is multi-scanner and multi-protocol (i.e. heterogeneous). This issue makes introducing such methods to clinical practice improbable. Therefore, it is crucial to develop methods that are ideally independent of the MRI scanner and protocol.<br>Results<br>This study introduces HeteroMRI, a deep-learning method for analyzing MRI datasets incorporating various MRI protocols and scanners. This method utilizes intensity clustering of the white matter tissue to minimize the effects of the heterogeneity of MRIs. Herein, the presented method is employed for developing a binary classifier that identifies brain MRIs with white matter abnormalities. MRI data from nine public datasets with 32 MRI protocols is included. By using 200 MRIs for the model, the binary classifier achieves an average accuracy of 96%. Furthermore, the method is evaluated with limited data, simulating scenarios of rare diseases where only limited data is available. By reducing the data by 64% and 75%, the model's accuracy has a 4% and 15% decrease, respectively.<br>Conclusions<br>The presented method opens up new avenues for the analysis of multi-protocol and multi-scanner MRI data. This approach demonstrates a high degree of independence from the MRI scanner and protocol, achieving superior performance even in limited data scenarios without needing machine learning techniques to minimize MRI heterogeneity.
**Corresponding Author:**	Navid Shekarchizadeh<br>Leipzig University: Universitat Leipzig<br>Leipzig, GERMANY
**Corresponding Author Secondary Information:**	
**Corresponding Author's Institution:**	Leipzig University: Universitat Leipzig
**Corresponding Author's Secondary Institution:**	
**First Author:**	Masoud Abedi
**First Author Secondary Information:**	
**Order of Authors:**	Masoud Abedi<br>Navid Shekarchizadeh<br>Pierre-Louis Bazin

Powered by Editorial Manager® and ProduXion Manager® from Aries Systems Corporation

	Nico Scherf
	Julia Lier
	Christa-Caroline Bergner
	Wolfgang Köhler
	Toralf Kirsten
**Order of Authors Secondary Information:**	
**Additional Information:**	
**Question**	**Response**
Are you submitting this manuscript to a special series or article collection?	No
**Experimental design and statistics**<br><br>Full details of the experimental design and statistical methods used should be given in the Methods section, as detailed in our Minimum Standards Reporting Checklist. Information essential to interpreting the data presented should be made available in the figure legends.<br><br>Have you included all the information requested in your manuscript?	Yes
**Resources**<br><br>A description of all resources used, including antibodies, cell lines, animals and software tools, with enough information to allow them to be uniquely identified, should be included in the Methods section. Authors are strongly encouraged to cite Research Resource Identifiers (RRIDs) for antibodies, model organisms and tools, where possible.<br><br>Have you included the information requested as detailed in our Minimum Standards Reporting Checklist?	Yes
**Availability of data and materials**<br><br>All datasets and code on which the conclusions of the paper rely must be	Yes

either included in your submission or deposited in publicly available repositories (where available and ethically appropriate), referencing such data using a unique identifier in the references and in the “Availability of Data and Materials” section of your manuscript.<br><br>Have you have met the above requirement as detailed in our Minimum Standards Reporting Checklist?	

Powered by Editorial Manager® and ProduXion Manager® from Aries Systems Corporation

Manuscript (everything included) Click here to access/download;Manuscript;MRI_Classification_Manuscript.p

HeteroMRI: A method for classification of multi-scanner and multi-protocol brain magnetic resonance images with deep learning

Masoud Abedi [1,2,3,†], Navid Shekarchizadeh [2,3,4,†,§], Pierre-Louis Bazin [5], Nico Scherf [4,6], Julia Lier [7,8], Christa-Caroline Bergner [7,8], Wolfgang Köhler [7,8], for the Alzheimer's Disease Neuroimaging Initiative [*], and Toralf Kirsten [1,2,3,4]

[1] Faculty Applied Computer and Bio Sciences, Mittweida University of Applied Sciences, Mittweida, Germany

[2] Department for Medical Data Science, Leipzig University Medical Center, Leipzig, Germany

[3] Institute for Medical Informatics, Statistics, and Epidemiology (IMISE), Leipzig University, Leipzig, Germany

[4] Center for Scalable Data Analytics and Artificial Intelligence (ScaDS.AI) Dresden/Leipzig, Leipzig University, Leipzig, Germany

[5] Full brain picture Analytics, Leiden, The Netherlands

[6] Neural Data Science and Statistical Computing, Max Planck Institute for Human Cognitive and Brain Sciences, Leipzig, Germany

[7] Department of Neurology, Leipzig University Medical Center, Leipzig, Germany

[8] Myelin Research Center (MRC) Leipzig, Department of Neurology, Leipzig University Medical Center, Leipzig, Germany

[§] Correspondence address. Navid Shekarchizadeh, ScaDS.AI, Humboldtstraße 25, 04105 Leipzig, Germany. E-mail: navid.shekarchizadeh@uni-leipzig.de

[†] Equal contribution

Abstract

Background

A common application of Magnetic Resonance Imaging (MRI) is the analysis of white matter abnormalities in the human brain. Integrating machine learning techniques in MRI data analysis can enhance diagnostic processes. However, utilizing machine learning techniques is restricted when the available MRI data is multi-scanner and multi-protocol (i.e. heterogeneous). This issue makes introducing such methods to clinical practice improbable. Therefore, it is crucial to develop methods that are ideally independent of the MRI scanner and protocol.

Results

[*] A part of the data used in preparation of this article were obtained from the Alzheimer's Disease Neuroimaging Initiative (ADNI) database (adni.loni.usc.edu). As such, the investigators within the ADNI contributed to the design and implementation of ADNI and/or provided data but did not participate in analysis or writing of this report. A complete listing of ADNI investigators can be found at: http://adni.loni.usc.edu/wp-content/uploads/how_to_apply/ADNI_Acknowledgement_List.pdf

This study introduces *HeteroMRI*, a deep-learning method for analyzing MRI datasets incorporating various MRI protocols and scanners. This method utilizes intensity clustering of the white matter tissue to minimize the effects of the heterogeneity of MRIs. Herein, the presented method is employed for developing a binary classifier that identifies brain MRIs with white matter abnormalities. MRI data from nine public datasets with 32 MRI protocols is included. By using 200 MRIs for the model, the binary classifier achieves an average accuracy of 96%. Furthermore, the method is evaluated with limited data, simulating scenarios of rare diseases where only limited data is available. By reducing the data by 64% and 75%, the model's accuracy has a 4% and 15% decrease, respectively.

Conclusions

The presented method opens up new avenues for the analysis of multi-protocol and multi-scanner MRI data. This approach demonstrates a high degree of independence from the MRI scanner and protocol, achieving superior performance even in limited data scenarios without needing machine learning techniques to minimize MRI heterogeneity.

Keywords— Brain MRI classification, Multi-scanner MRI, Multi-protocol MRI, Intensity clustering, White matter abnormality, Rare disease, Deep learning, Convolutional neural network

1 Introduction

Magnetic Resonance Imaging (MRI) is widely used in clinics and hospitals to diagnose and follow up neurological diseases by generating images of the central nervous system including the brain. MRI provides a clear contrast between the different tissues of the brain, including White Matter (WM) and Gray Matter (GM) [1]. Detection and assessment of WM abnormalities in demyelinating or neurodegenerative diseases are an important application of MRI in daily clinical practice [2]. An excellent contrast for visualizing WM abnormalities is provided by the FLAIR (Fluid-Attenuated Inversion Recovery) imaging technique making the abnormalities stand out from the surrounding normal brain tissue. FLAIR is a T2-weighted imaging technique in which the signal from cerebrospinal fluid (CSF) is suppressed. By reducing the interference of CSF, the detection of WM abnormalities becomes easier, as these abnormalities may appear adjacent to CSF-filled spaces [3]. MRI provides a three-dimensional (3D) view of the brain and other anatomical structures, making it possible to accurately assess and measure the volume of WM lesions. Volume measurements of the lesions help clinicians to track the disease progression and the effectiveness of the treatments. Moreover, the pattern and volume of lesions are used in research studies to investigate the behavior of different diseases which ultimately contribute to image-based diagnosis of demyelinating disorders [4].

In recent years, Artificial Intelligence (AI) has revolutionized the medical imaging domain, bringing substantial benefits to the analysis of such data [5, 6]. Automating certain aspects of the image analysis process reduces the need for repetitive and time-consuming tasks such as lesion segmentation. This allows healthcare specialists to allocate their expertise to more critical aspects of clinical practice and research. Moreover, manual analysis such as segmentation of MRI data, especially when dealing with complex struc-

tures like demyelinating lesions, is resource-intensive and prone to examiner-based variability. AI allows for rapid and consistent analysis across a large amount of images. A large and growing body of literature has investigated the utilization of Machine Learning (ML) [7, 8] and Deep Learning (DL) [9, 10] models in analyzing medical images.

In brain MRI analysis, studies have focused on developing different AI models using ML techniques, e.g. for disease classification [11, 12, 13], WM lesion segmentation [14, 15], tumor detection and grading [16, 17], stroke lesion detection and segmentation [18, 19], brain age prediction [20, 21], tracking disease progression in neurodegenerative disorders using longitudinal MRI data [22, 23], and automated segmentation of brain tissues, such as GM, WM, and CSF, for quantitative analysis and volumetric measurements [24, 25]. Before analysing brain MRIs, certain preprocessing steps are commonly performed to optimize the image data for further analysis and interpretation. AI models and mathematical algorithms are generally used for preprocessing steps including image registration [26], brain extraction or skull stripping [27, 28], image denoising [29, 30, 31], intensity normalization [32, 33], bias field (also called inhomogeneity) correction [34], and MRI interpolation [35]. For protecting the privacy and anonymity of the individuals whose brain images are being analyzed, defacing [36] algorithms are employed.

In many types of AI methods mentioned above for MRI data analysis, including lesion segmentation, it is highly recommended that all the images are *standardized* [37, 38, 39]. Here, "standardized" means the images have been acquired with the same acquisition protocol, with the same magnetic field strength, and preferably with the same MR scanner model. Acquisition protocol refers to a set of procedures and parameters, e.g. Echo Time (TE), Repetition Time (TR), and Inversion Time (TI), used to acquire the images. Using a standardized dataset ensures that the images have similar image quality, similar intensity range for each tissue, and similar spatial characteristics. Moreover, the contrast among the brain tissues would be uniform across the dataset [40, 37]. Furthermore, this consistency allows the model to effectively recognize and learn the disease patterns and features across the images, leading to more accurate and reliable results.

The necessity for a standardized dataset poses a significant limitation in utilizing routinely available MRIs for analysis. Numerous MRIs are produced daily at hospitals or clinics. However, since the images are usually not standardized across centers or even within one, their use for an AI-based analysis is limited, restricting the clinical applicability of AI methods. Moreover, generating a standardized dataset imposes additional costs and requires a well-structured organization at the medical center(s).

The variability of MRIs across different sites was studied in Ref. [40]. In this study, a multiple sclerosis (MS) patient underwent imaging at seven different sites, all following a similar acquisition protocol. Notably, clear differences in volumetric measurements were observed, showing significant variations in both manual and automatic WM lesion segmentations across sites. This variability persisted even among the sites that had scanners from the same manufacturer and model. Furthermore, also rescanning the patient on the same scanner on the same day showed variations in the lesion volume measurements. Such systematic differences are also referred to as batch effect in the literature [41].

Literature concerning techniques for removing scanner and protocol effects from heterogeneous MR images is limited. Such methods are referred to as *harmonization*. Harmonization involves considerable complexities and challenges due to the limited understanding of scanner effects, and the absence of standardized criteria for assessing scanner effects and evaluating the harmonization process [42]. The proposed MRI harmonization methods in the literature are either based on statistics or learning (AI). The methods based on statistics alter the intensity distribution in the image either via normalizing the intensity [32, 43, 44, 45] or adjusting the batch effect [46, 47, 48]. Nonetheless, these techniques are usually restricted to harmonizing the entire image and lack the capacity for harmonization at the brain tissue level. The intensity normalization methods can be considered more as data preprocessing techniques (for scaling the intensity distribution of the images before training the AI models) rather than an MRI harmonization method because if the heterogeneity is too severe, these statistical models will not be able to harmonize the variations caused by the different protocols. The harmonization methods based on learning have the goal of learning the scanner and protocol effects and removing them from the multi-center images. The learning-based MRI harmonization models include approaches that are built upon supervised ML [49, 50], supervised DL [42, 51, 52, 53], and unsupervised DL [54, 55] methods. Supervised ML techniques are commonly employed to predict harmonized images by training regression models incorporating manually selected features. On the contrary, DL techniques automatically capture relevant features for the harmonization task. Recently, a novel class of models has been proposed, which integrates both statistical and DL approaches [56]. For an extensive review of MRI harmonization methods, refer to Ref. [41].

Supervised DL methods often rely on training data derived from human patients at different sites, forming what is known as a "matched" dataset. This approach, as exemplified in Ref. [42], involves harmonizing MRIs taken of the same individuals across different locations. This method is categorized as supervised DL because it allows the model to observe variations in brain MRIs from the same individual across different scanners. A noteworthy aspect of the method in Ref. [42] is the use of a relatively small dataset comprising MRIs from 18 individuals. However, a significant limitation arises in its exclusive applicability to matched datasets. Essentially, this method is not suitable for datasets containing MRIs from different patients across diverse medical centers.

While unsupervised DL models do not rely on a matched dataset, such models usually have other limitations. For instance, the method proposed in Ref. [54] harmonizes the images taken by different scanners by removing the non-biological site differences. However, its applicability is restricted to datasets with uniform image protocols, as the acquisition protocols were standardized across all scanners. Another unsupervised DL model, as presented in Ref. [55], addresses the harmonization of multi-contrast multi-site MRIs. Their method necessitates multi-contrast images of the same subject within a single imaging session, with training data acquired from 10 scanners following a standardized protocol on each.

Despite the methods that have been proposed for MRI harmonization, there is no universal or standardized procedure for effectively harmonizing multi-site and multi-protocol MRI data. Notably, as mentioned

in Ref. [37], there have been no studies conducted to evaluate the impact of a change in MR acquisition parameters (including TE or TR) on the assessment of brain WM lesions, even using the same MR scanner and sequence. The harmonization of MRI data continues to be a complex and unsolved issue, given the variations that come with the nature of MRI technology.

The application of AI in the context of rare diseases has specific challenges and limitations. Data availability for rare diseases is extremely limited, which severely restricts the development of AI models for such diseases. In the case of brain MRI data for neurological rare diseases like leukodystrophies [57]), the images are gathered from various clinical centers over a long period, making it infeasible to create a standardized dataset. Datasets created by collecting MRIs from multiple centers are not only small in size but also highly heterogeneous in terms of MRI scanners and protocols. These conditions significantly hinder the applicability of AI methods for analyzing the data. In addition, the existing literature lacks MRI harmonization methods that can handle such high levels of heterogeneity in a small dataset.

In this paper, to circumvent the complex MRI harmonization, we present HeteroMRI, an approach for analyzing multi-protocol and multi-scanner MRI data. The method utilizes MRI intensity clustering, a technique used in the literature for other MRI-related purposes such as brain tissue segmentation [58, 59], brain tumor segmentation [60] and inhomogeneity correction [61]. Herein, the presented approach is evaluated for detecting the brains with WM abnormalities through binary classification. This innovative approach enables the analysis of datasets that include MRI data from various scanners and acquisition protocols, providing a flexible and practical solution for research and clinical applications. The method is evaluated in various experimental settings to ensure its robustness. Additionally, we apply the method to limited data scenarios in order to assess the performance and applicability of the presented method for rare diseases. The presented method opens new avenues for analyzing heterogeneous MRI datasets and the large amount of MRI data generated daily in medical centers.

The current paper is structured in the following way: Sec. 2 provides an overview of the methodology used, detailing the data preprocessing steps and the architecture of the DL model employed in this study. Moving on to Sec. 3, the experiments, explaining the used datasets, experimental settings, and the execution of the model are presented. Following that, in Secs. 4, the key results of this study are presented and discussed. Finally, Sec. 5 summarizes the main contributions of our research and future work.

2 Methodology

The WM abnormality detection approach presented in this paper uses heterogeneous brain MRI data with various acquisition protocols (multi-protocol) as the data for a Convolutional Neural Network (CNN). The model is a binary classifier trained to detect patients with WM abnormalities in their brain MRI. The method consists of three main modules explained in the following subsections, namely MRI preprocessing, Intensity clustering, and DL model. An overview of the methodology is illustrated in Fig. 1.

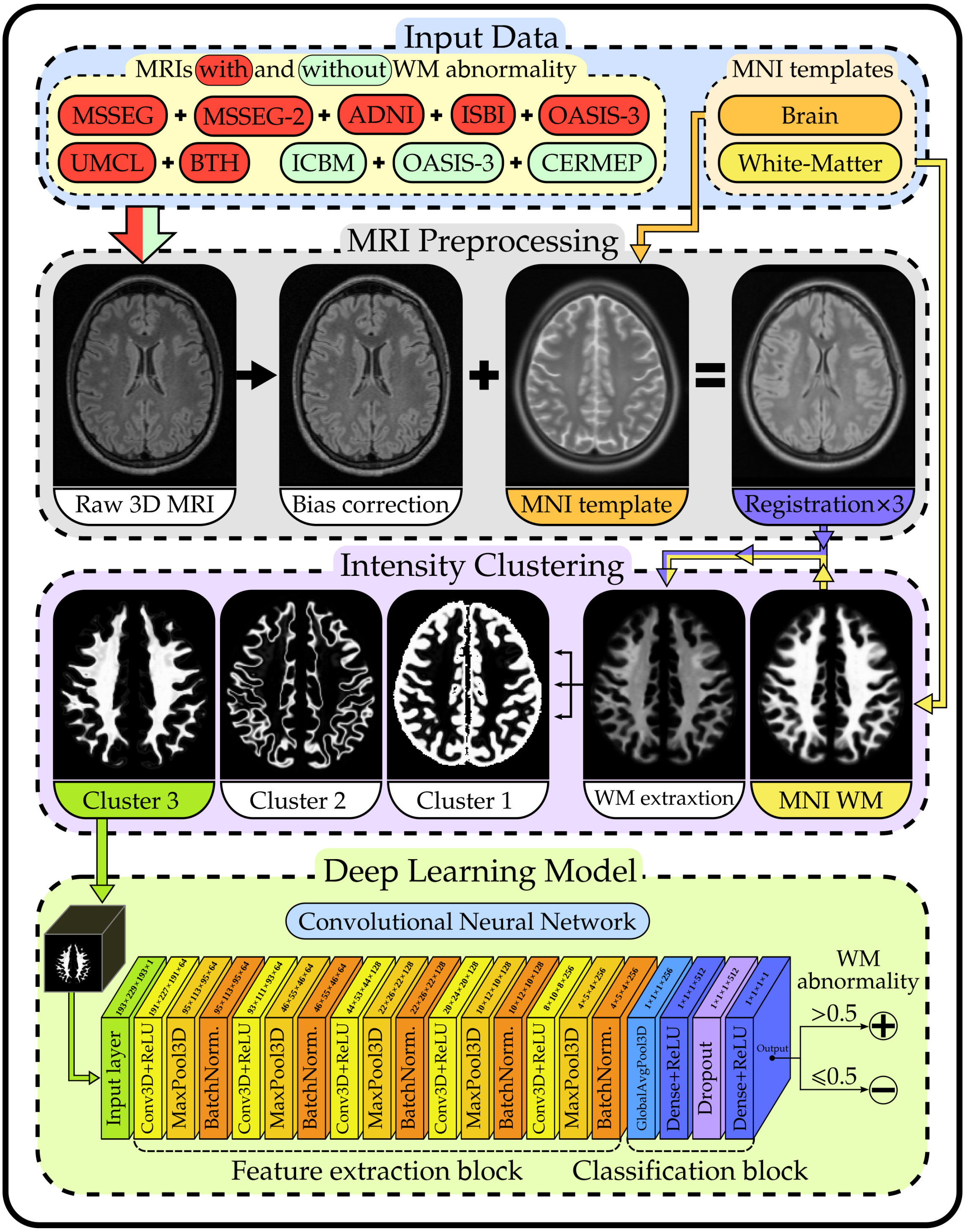

Figure 1: Overview of the methodology: Input data (top): The MRI datasets used for the classification model and MNI brain template [62, 63]. The MRI data with and without WM abnormality are taken from the datasets shown in red and green, respectively. MRI preprocessing (upper middle): The N4 bias field correction method [34] is applied on the FLAIR MRIs (in 3D) and then the MRIs are three times registered (nonlinearly) to the MNI template. Intensity clustering (lower middle): The WM of the brain is extracted and the WM is clustered into three intensity clusters using RFCM [64] algorithm. DL model (bottom): Only cluster 3 of the WM is used for a binary classification model with the CNN architecture shown.

2.1 MRI preprocessing

For preparing the image data for the analysis, we use our brain MRI preprocessing pipeline, *FlexiMRIprep*[1], that consecutively performs all the requested preprocessing steps/algorithms on all the selected images automatically. Being the optimal MRI sequence in detecting WM abnormalities, only FLAIR images are used in the analysis in this paper. All selected images have a minimum of 128 and 192 voxels in their first and second dimensions, respectively. All the MRIs are converted to NIfTI-1 format using the `dcm2niix` tool (version 1.0.20211006) [65] at this point. The preprocessing steps are described below. Detailed information on the parameters used in each step is reported in the GitHub repository of HeteroMRI[2].

- **Bias field correction**: For correcting the bias field or inhomogeneity issues in the MRIs, we employ the commonly used N4ITK [34] bias field correction method for this purpose. For implementation, the `N4BiasFieldCorrectionImageFilter` class from the `SimpleITK` [66] (version 2.1.1.2) *Python* library with the default parameters is used.

- **Registration**: Registration enables precise spatial mapping and the comparison of anatomical structures among the MRIs. In this process, all the MRIs used for training and testing the AI model are aligned to a standard brain template. Among the available brain templates, we choose the "ICBM 2009c Nonlinear Asymmetric" template [62, 63] (referred to below as the MNI template) since we need a highly accurate template that also provides the WM probability map which is required in our analysis approach. This template is created using the data from the ICBM (International Consortium for Brain Mapping) project [67]. Since there is no dedicated FLAIR template in the MNI template, the T2-weighted template was used due to its proximity to FLAIR. For the registration, the `antsRegistration` tool from the Advanced Normalization Tools (ANTs) [68] (version 2.4.4.post12-g8cc4f8a) is employed. A nonlinear registration is applied three times (with identical parameters) on each image consecutively. This repeated registration aims to achieve the most precise alignment of the MRIs with the template.

2.2 Intensity clustering

- **WM extraction**: After having the brain images aligned with the MNI template, the WM probability map of the template is used to extract the WM volumes of each brain. Therefore, all the other brain tissues are removed. The WM extraction is performed by using the `MultiplyImages` tool from ANTs.

- **WM clustering**: The WM clustering is performed for two essential purposes: 1) Calculating and obtaining a sub-group of WM volumes that includes significant signs of WM abnormalities, and 2) Minimizing the negative effect of the heterogeneous MRI data coming from multiple scanners and protocols. These two points are elaborated on in the following.

  A clustering algorithm is used to estimate a certain number of intensity clusters from the WM volumes obtained in the previous step. The algorithm groups the WM volumes into subgroups that

[1] https://github.com/ul-mds/FlexiMRIprep
[2] https://github.com/ul-mds/HeteroMRI

share a relatively similar intensity range. We expect that one of the clusters shows more indications of WM abnormalities (if present in the brain) since the abnormalities have higher intensity values in FLAIR images. Such a cluster will be used as the training data for the classifier model. The cluster is a membership function with float values in the range of [0,1] for each voxel. As a result, the impact of the heterogeneous nature of the multi-protocol MRIs is minimized.

Here, we employ a Robust Fuzzy C-Means (RFCM) algorithm [64] for WM intensity clustering. The RFCM algorithm modifies the standard FCM objective function by incorporating a local spatial penalty term, leading to the computation of smoother membership functions. This modification not only improves segmentation performance but also provides a level of noise insensitivity. The RFCM algorithm is implemented using the `fuzzy_cmeans` function available in the *Nighres* (Neuroimaging at high resolution) *Python* package, version 1.4.0 [69]. The choice of using three clusters is based on our empirical observations. We tested various cluster numbers on different MRIs and found that three clusters yielded consistently similar patterns across almost all MRIs. In other words, the three clusters in one MRI relatively match the shapes of the three clusters in another MRI.

Upon examining the three WM intensity clusters in MRIs with WM abnormalities, we noted that one of the clusters within each MRI consistently exhibited significant lesion-related features. In the MRI data that we analyzed, usually the third cluster and sometimes (in less than 10% of the cases) the second cluster was the one including the abnormalities. For this study, we manually identified the intended cluster for each MRI by choosing the cluster that looks visually similar to Cluster 3 in Fig. 1. However, the intended cluster could also be automatically detected through the training of a DL model. Finally, all the selected clusters from all the MRIs (one intensity cluster per MRI) serve as the training data for the DL model, as described in the following section.

2.3 Deep learning model

The objective is to train a binary classifier model that detects the brain MRIs that have WM abnormalities. Inspired by [70, 71], we configured a 3D CNN comprising a total of 20 layers, as illustrated in Fig. 1 (at the bottom). The network has a total of 1,795,905 parameters. The model begins with the input layer, followed by a feature extraction block, and ends with a classification block.

In the feature extraction block, we employ five 3D Convolution (Conv3D) layers with 64, 64, 128, 128, and 256 filters, respectively. Each Conv3D has a 3x3x3 kernel size and employs the Rectified Linear Unit (ReLU) activation function. Subsequently, each Conv3D layer is succeeded by a 3D Max Pooling (MaxPool3D) layer with a stride of (2,2,2) and a pool size of (2,2,2) which downscales the 3D input by half in each dimension. Batch Normalization [72] layers with default parameters follow each MaxPool3D layer.

In the classification block, a 3D Global Average Pooling (GlobalAvgPool3D) layer is followed by a Dense

layer with a dimensionality of 512 and with a ReLU activation function. To help prevent overfitting, a Dropout layer with a 30% rate is introduced next. Finally, the output layer performs a binary classification employing a Sigmoid activation function. The binary cross-entropy loss, Adam optimizer [73], and an *Early Stopping* feature (*patience*=40) are employed in the model. In each epoch, the checkpoint feature saves the model if the validation accuracy has improved. In the case of an unchanged validation accuracy, the mode is saved if the validation loss has decreased. The *Python* implementation code of the HeteroMRI method is publicly available[3].

3 Experiments

Different MRI datasets are used along with multiple experimental settings with various conditions to train and evaluate the CNN model for classifying brain MRIs. In the following subsections, the datasets and the experimental settings are elaborated.

3.1 Datasets

In this study, we utilized FLAIR images from multiple brain MRI datasets as introduced below. Incorporating a combination of MRIs with a high diversity of acquisition protocols and scanners ensures a robust evaluation of the presented methodology. All the datasets used in this study are either publicly available or are accessible upon request to the respective dataset providers. As presented in Table 1, a total of nine MRI datasets are utilized.

Table 1: List of MRI datasets used in this study

Dataset name/alias	Images[1]		Protocols[2]	Availability	Reference
	+	−			
ISBI	19	0	1	Public	[74]
UMCL	30	0	1	Public	[75]
MSSEG	52	0	4	AoR[3]	[76]
MSSEG-2	40	0	10	AoR[3]	[77]
BTH	9	0	2	Public	[78]
ICBM	0	5	1	AoR[3]	[79]
OASIS-3	14	90	4	AoR[3]	[80]
ADNI	58	0	8	AoR[3]	[81]
CERMEP	0	27	1	AoR[3]	[82]
Sum	222	122	32	-	-

[1] Number of FLAIR images, with (+) and without (−) WM abnormality
[2] Number of MRI protocols in the used data
[3] Accessible on Request (to the respective dataset provider)

The details of each dataset are outlined below:

- **ISBI**: The International Symposium on Biomedical Imaging (ISBI) in 2015 [74] conducted an MS lesion segmentation challenge using longitudinal MRI data. The dataset comprises imaging data from MS patients, acquired using the same scanner and protocol. We utilize 19 FLAIR images from this dataset. For each patient, the MRI taken at the latest time point is used.

[3] https://github.com/ul-mds/HeteroMRI

- **UMCL**: A cohort of MS patients was imaged at the University Medical Center Ljubljana (UMCL) [75]. The images were acquired using the same scanner and protocol. We use 30 3D FLAIR images from this dataset.
- **MSSEG**: The MSSEG dataset [76] was presented for the MS lesion segmentation challenge during the MICCAI 2016 conference. The dataset contains MRIs of MS patients from four different sites. Each site used different MRI scanners and protocols. We utilize 52 FLAIR images from this dataset.
- **MSSEG-2**[4]: MSSEG-2 [77] is a challenge for the segmentation of new MS lesions in the brain conducted in the MICCAI 2021 conference. At the time of the current research, only the training data of the dataset is accessible. The images of the training set were acquired at 12 different sites and using 10 different scanners. All the images were acquired at two different time points from each patient. From this dataset, we utilize 40 3D FLAIR images from the second time point.
- **BTH**: The brain MRI dataset of MS patients from Baghdad Teaching Hospital (BTH) [78] includes MRIs taken at 20 centers with different protocols. We used nine[5] FLAIR images from this dataset, which were taken using two different protocols.
- **ICBM**[6]: The International Consortium for Brain Mapping (ICBM) [79] has developed a probabilistic atlas and reference system for the human brain for normal adults. The dataset includes 20 3D FLAIR images; however, only five were selected for this study, as our neurology specialist confirmed these to be the only ones free of white matter abnormalities. The images were acquired using the same scanner and protocol.
- **OASIS-3**: The Open Access Series of Imaging Studies (OASIS) is a project with the goal of providing open access to neuroimaging datasets of the brain. Among the OASIS datasets, FLAIR images are exclusively available in OASIS-3 [80]. OASIS-3 encompasses both cognitively normal adults and individuals at different stages of cognitive decline. Our model utilizes a total of 104 FLAIR and T2-FLAIR images from this dataset, acquired using four different protocols. Out of these images, 90 were meticulously selected by two neurology specialists from a pool of 600, confirming the absence of white matter abnormalities in the brain. The other 14 images included different patterns of WM abnormalities.
- **ADNI**: The Alzheimer's Disease Neuroimaging Initiative (ADNI) database[7] provides an extensive collection of neuroimaging and clinical data [81]. In our model, we included 58 3D FLAIR images

[4]Data were generated by participating neurologists in the framework of Observatoire Français de la Sclérose en Plaques (OFSEP), the French MS registry [83]. They collect clinical data prospectively in the European Database for MS (EDMUS) software [84]. MRI of patients were provided as part of a care protocol. Nominative data are deleted from MRI before transfer and storage on the Shanoir platform (Sharing NeurOImagingResources, shanoir.org).

[5]The NIfTI files in this dataset lack orientation information (qform and sform), making it impossible for the registration algorithm to identify the correct orientation of the brain. Additionally, the MRIs are 2D, resulting in around 10 times fewer layers than the pixels in the first and second dimensions, and they all share the same pixel thickness across all three dimensions. Consequently, the brain appears unrealistically short in 3D view. Due to these dataset-specific conditions, we applied two additional preprocessing steps at the beginning for this dataset: 1) added correct orientation information to each file, and 2) edited layer thicknesses in the header of NIfTI files based on layer thickness information provided in the dataset's metadata. However, the height of many images still does not appear realistic and may cause problems for the registration. Therefore only nine images were used

[6]The ICBM project (Principal Investigator John Mazziotta, M.D., University of California, Los Angeles) is supported by the National Institute of Biomedical Imaging and BioEngineering. ICBM is the result of efforts of co-investigators from UCLA, Montreal Neurologic Institute, University of Texas at San Antonio, and the Institute of Medicine, Juelich/Heinrich Heine University - Germany.

[7]The ADNI was launched in 2003 as a public-private partnership, led by Principal Investigator Michael W. Weiner, MD. The primary goal of ADNI (`https://adni.loni.usc.edu`) has been to test whether serial MRI, positron emission tomography (PET), other biological markers, and clinical and neuropsychological assessment can be combined to measure the progression of Mild Cognitive Impairment (MCI) and early Alzheimer's Disease (AD).

from this dataset, all of which contained WM abnormalities. These images were carefully selected to encompass various patterns of lesions, including multifocal and confluent lesions, as well as those located in the brain stem, as confirmed by a neurology specialist.

- **CERMEP**: The CERMEP-IDB-MRXFDG dataset [82] comprises MRI, CT, and [^{18}F]FDG PET image data with BIDS standard of healthy subjects. The dataset has 37 FLAIR images obtained using the same scanner and protocol. As reported in the original study, these images underwent visual review by two neurologists to confirm the absence of any apparent brain abnormalities. However, due to our strict criteria for even minor lesions, our neurologists confirmed only 27 MRIs as free of WM abnormalities for use as control data in our model.

From the MRI datasets explained above, a total of 344 images are used in this study for training and testing the model. A comprehensive list of the MRI files is available in the GitHub repository of HeteroMRI providing details for each image, including the subject ID from the original dataset and the acquisition protocol.

3.2 Experimental settings

Various experimental settings, have been designed for a robust evaluation of the presented classification approach. An experimental setting means the specification of the data used for training, validating, and testing the CNN model. By employing the datasets explained in Sec. 3.1, MRIs with different protocols are intentionally combined and used for training and testing the model. The number of MRIs with and without WM abnormality is balanced in the training data of all the settings. The same holds for the test data. In total, there are four groups of settings, namely A, B, C, and D. In setting A, the data are selected based on datasets while in the settings B, C, and D, the data are incorporated based on their acquisition protocol. We assigned a protocol name to each of the MRIs based on the scanner name and model, magnetic field strength, and acquisition parameters. The protocol naming convention is explained in the HeteroMRI Github repository. The experimental settings are introduced below:

- **Setting $\boldsymbol{A}$**: In setting A, there are 19 settings that are run independently. In $A00$, 244 MRIs from nine datasets are used. The data of each dataset is split into training (70%), validation (10%), and test (20%) sets. In $A01$, the training data is downsized by approximately 10% while the test set remains the same as in $A00$. The downsizing process continues up to $A18$, where the training and validation sets together include only four MRIs. Across all settings from $A00$ to $A18$, the test set remains identical. Supplementary Table S1 shows the number of MRIs used for training, validation, and test sets from each dataset in the settings $A00$ to $A18$.

- **Setting $\boldsymbol{B}$**: In setting B, the goal is to choose the MRIs with the most diversity of protocols while having an equal number of MRI from each protocol. Therefore, an equal number of MRIs is incorporated from 10 different protocols. The test data is selected from all protocols. In $B00$, from each protocol, five MRIs for training, one MRI for validation, and one MRI for the test are used. In the next subsequent settings, the training data is reduced. By $B04$ only one MRI per protocol is used for the training set. In Supplementary Table S2, the list of selected protocols and the number of MRIs used for training, validation, and test sets for $B00$ to $B04$ is reported.

- **Setting *C***: In this setting, MRIs from eight different protocols are included. Only data from 3D MRIs are included in this setting due to the reason later explained in Sec. 4.3. MRIs from six protocols are used *only* in training and validation sets while the other two protocols are *only* used in the test set. In fact, the trained model does not see any data from the protocols used in the test set during training. We consider 10 cases, in each case considering two different protocols for testing the model. The setting C begins with $C00$ which uses 64 MRIs for training and validation. This continues up to $C06$ with only eight MRIs for training and validation. In Table S3, the list of selected protocols and the number of MRIs used for training, validation, and test sets for $C00$ to $C06$ is reported.
- **Setting *D***: In setting D, the goal is to see the effect of the number of MRI protocols on the performance of the model. Beginning from $D00$ and going toward $D03$, more protocols are included in the data used for training and testing the model. At the same time, the total number of data and test set size are kept the same among the settings $D00$ to $D03$ (82 MRIs for training including validation data), therefore it is possible to compare the results of the settings to see the effect of having more protocols in the data. In $D00$, there are MRIs from four protocols. In $D01$ to $D03$, there are MRIs from, respectively, six, eight, and 10 protocols. The test data is selected from all protocols. In Table S4, the list of selected protocols and the number of MRIs used for training, validation, and test sets for $D00$ to $D03$ is reported.

3.3 Model execution

All the MRIs used in this study, introduced in Sec. 3.1, are preprocessed following the procedure elaborated in Sec. 2.1. Next, the intensity clustering procedure is applied to each preprocessed MRI, following the procedure introduced in Sec. 2.2. As a result, a single intensity cluster per MRI is used for training or testing the model. Notably, the 3D intensity clusters obtained from the MRIs serve as the exclusive training data for the model. The model has no exposure to the original MRIs or any form of WM lesion annotation file. For the preprocessing and intensity clustering tasks, we used a machine with Intel(R) Xeon(R) Gold 6240R CPU @ 2.40GHz and 128 GB of RAM. The preprocessing pipeline (introduced in Sec. 2.1) employs a parallelization approach in some of the preprocessing steps to make the procedure faster. The computation time required for preprocessing each MRI depends on multiple factors; nevertheless, the total number of voxels in the 3D MRI plays a more significant role. More specifically, based on our assessments, the number of layers of the MR image highly affects the required computation time. The Supplementary Fig. S1, shows the average time required for preprocessing and intensity clustering of five sample MRI dimensions.

The CNN model explained in Sec.2.3 is trained and tested on each experimental setting independently. In settings A, B, and D, the required number of data is selected (and split into training, validation, and test sets) from all available data 20 independent times (referred to below as "data shuffle"). For each data shuffle, the model is trained and tested 10 times. In setting C, 5 data shuffles are selected, and for each shuffle, the model is trained and tested 10 times. For training and testing the CNN model, we used a computational server with AMD Epyc 7352 CPU, 1 TB of CPU RAM, and NVIDIA A100 GPU (40 GB

GPU RAM). The average required time for training the model of each setting is shown in Supplementary Fig. S2. The inference time of the model on a single test data is a few seconds. The inference process can be efficiently performed without the necessity of a GPU.

4 Results and discussion

In this section, we present and analyze the results of the experiments to evaluate the performance of the classification model. The results are reported separately for the experimental setting A, B, C, and D. Furthermore, the insights on limited data scenarios gained through the experiments are discussed afterward. The classification metrics are provided in box plot and radar plot format in Figs. 2, 5, 6, and 7. In settings A, B, and D, the box plots show the distribution of 200 values for each setting # corresponding to 20 data shuffles that each has been run 10 times. Setting # means for example $A00, A01, \ldots, A18$. The higher the setting #, the less training data is used. In setting C, the box plots show the distribution of 10 values for each setting #. Each of these 10 values is the average of 50 values (five shuffles, 10 runs each) corresponding to the 10 cases with different protocols chosen as the test set, as explained in Sec. 3.2.

The radar plots illustrate the metric values for all the setting #s simultaneously, allowing us to perceive the effect of reducing training data size on each metric. In addition, the radar plots are utilized for calculating the Machine Learning Cumulative Performance Score ($MLcps$) [85]. $MLcps$ is a *Python* package for classification problems that combines various pre-computed performance metrics into a single metric that encapsulates the core aspects of all the metrics. The value of $MLcps$ is equal to the area of the polygon created by the metrics in the radar plot. We used the $MLcps$ *Python* package version 0.0.6. The $MLcps$ metric was originally designed for comparing and identifying the best-performing ML algorithm. However, herein, we utilize $MLcps$ to compare the performance of the same model for the different amounts of training data. As we have a fixed number of pre-calculated metrics (accuracy, sensitivity, specificity, F1 score, and precision), we define $MLcps\%$ as

$$MLcps\% = \frac{MLcps}{MLcps_{max}} \times 100, \tag{1}$$

where $MLcps$ is the area of the pentagon in the radar plot of each setting and $MLcps_{max}$ is the area of the pentagon when all the five metrics are 100%. The $MLcps\%$ values for the settings A, B, and C are reported in the Figs. 2, 5, and 6 in part (e).

Evaluation of the presented approach across various experimental settings provides several key insights, offering a detailed understanding of its performance and challenges. The results of each experimental setting are presented below.

4.1 Setting *A*

As shown in Fig. 2, setting $A00$, in which the highest number of MRIs (174 training+26 validation+44 test) were included, demonstrates an average accuracy of 96% in the classification of MRIs. The training and testing data in this setting includes in total 32 different MRI protocols. This underscores the model's

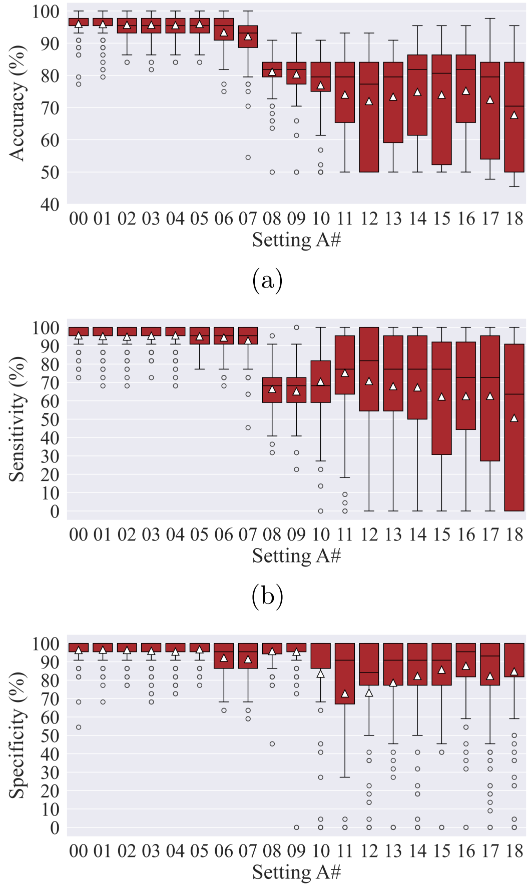

(a) (b) (c)

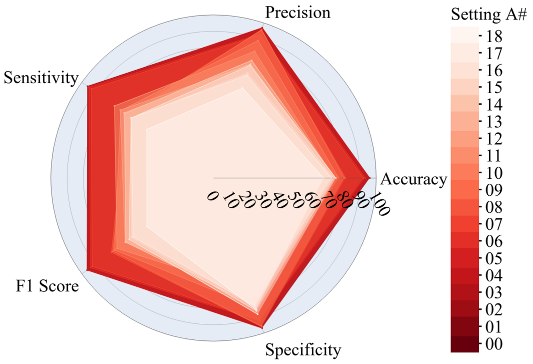

(d)

**Setting**	$A00$	$A01$	$A02$	$A03$	$A04$
$\boldsymbol{MLcps\%}$	91	91	91	90	90
**Setting**	$A05$	$A06$	$A07$	$A08$	$A09$
$\boldsymbol{MLcps\%}$	91	85	83	64	63
**Setting**	$A10$	$A11$	$A12$	$A13$	$A14$
$\boldsymbol{MLcps\%}$	56	51	45	47	49
**Setting**	$A15$	$A16$	$A17$	$A18$	
$\boldsymbol{MLcps\%}$	45	48	43	34	

(e)

Figure 2: Classification results of settings $A00$ to $A18$: (a) accuracy, (b) sensitivity, (c) specificity, (d) radar plot of five classification metrics for different setting #s, and (e) $MLcps\%$ (a cumulative performance score) in % for each setting #. In each setting # (i.e. $A00$, $A01$, . . ., $A18$) the training set size is sequentially reduced by approximately 10% relative to the previous setting, as detailed in Supplementary Table S 1. Average accuracy starts at 96% for $A00$, where training includes 200 MRIs, and remains above 92% until $A07$, with 72 training data, after which it drops with further reductions in training data. A similar trend is observed in the $MLcps\%$.

adaptability and robustness in handling a diverse range of imaging protocols. Notably, the effect of reducing the training data on the model's performance is inspected here. In $A07$, where the training data (including validation data) is reduced to 36% of $A00$, the accuracy remains above 92%. Beginning from setting $A08$, where the training data is 25% of $A00$, the average accuracy and sensitivity have a sharp decrease. With much further decreasing the training data (e.g. $A18$ with only two MRIs as training) the accuracy and sensitivity gradually decrease to low values as expected while specificity tends to remain relatively high.

Sample MRIs and the WM intensity cluster obtained from each of them are provided here for a better insight into the data used for testing the model and the obtained result. Figs. 3 and 4 depict samples of MRIs without and with WM abnormality, respectively. Below each original MRI, the obtained WM intensity cluster is illustrated. Additionally, the model's prediction for the presence of WM abnormalities in setting $A00$ is reported. Each figure includes three true predictions and three false predictions. It is important to note that the shown WM intensity cluster slice does not directly correspond to the original MRI slice. This is because the non-linear registration process during the MRI preprocessing results in significant deformation of the brain structure and the MRI's number of layers is changed. In addition, the

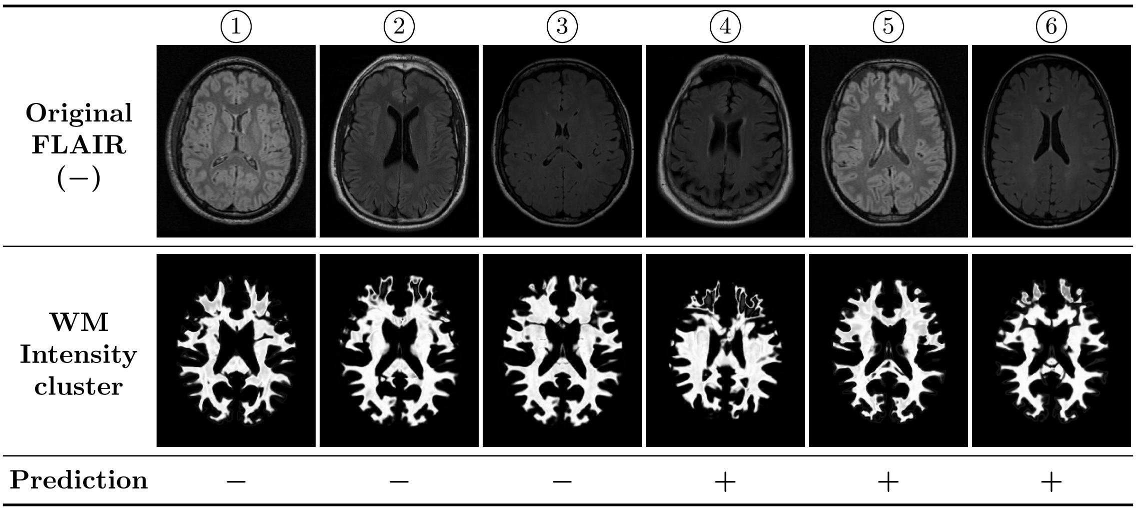

Figure 3: Example of six MRIs (① to ⑥) *without* WM abnormality. Top: original FLAIR images, Middle: the obtained intensity clusters used for testing the model, and Bottom: the prediction of the model in setting $A00$ for the presence of WM abnormalities ($+$: with WM abnormality, $-$: without WM abnormality). This figure shows three cases of true negative and three cases of false positive. The WM intensity cluster slice does not exactly correspond to the original MRI slice due to deformations from non-linear registration and only one of the three WM parts being present in this cluster. MRIs source: ①,⑤:[82], ②,④:[80], ③,⑥:[79].

WM of the brain is clustered into three parts, and the illustrated WM intensity cluster is only one of the three parts. As a result, finding exactly the corresponding slices in the original MRI and the WM intensity cluster is impractical. These sample slices of the WM cluster are presented here solely to illustrate the input provided to the DL model for label prediction.

By investigating the possible reasons for the false predictions in setting $A00$, certain aspects became apparent. In some MRIs, the registration process has not been successful in correctly aligning the brain to the brain template. In such cases, the brain regions are not located in the correct locations after the three times of registration (discussed in Sec. 2.1). Therefore, in the WM extraction step, wrong parts of the brain are extracted as WM. This mostly results in a false prediction by the model, especially if the MRI is, in fact, free of WM abnormalities. The examples of wrong registration are MRIs ④, ⑤, ⑥, and ⑩ shown in Figs. 3 and 4. We noticed that the registration problem happened more with 2D MRIs (such as MRI ④) although it is not the only reason. Around 35% of MRIs used in this study were 2D. The nonlinear registration algorithm did not succeed in registering some of the 2D MRIs due to their low resolution in the 3rd dimension, i.e. having thick layers.

Another factor causing a false negative prediction is seen in MRI ⑫. By inspecting the three intensity clusters of this MRI, we noticed that the trace of the WM abnormalities is present in a different intensity cluster than the one used by the model for its prediction. As shown in Fig.4, the WM intensity cluster of MRI ⑫ lacks the regions relevant to WM abnormalities (small dark areas). Consequently, the model has classified this MRI as negative. However, this source of false prediction occurs rarely among the MRIs.

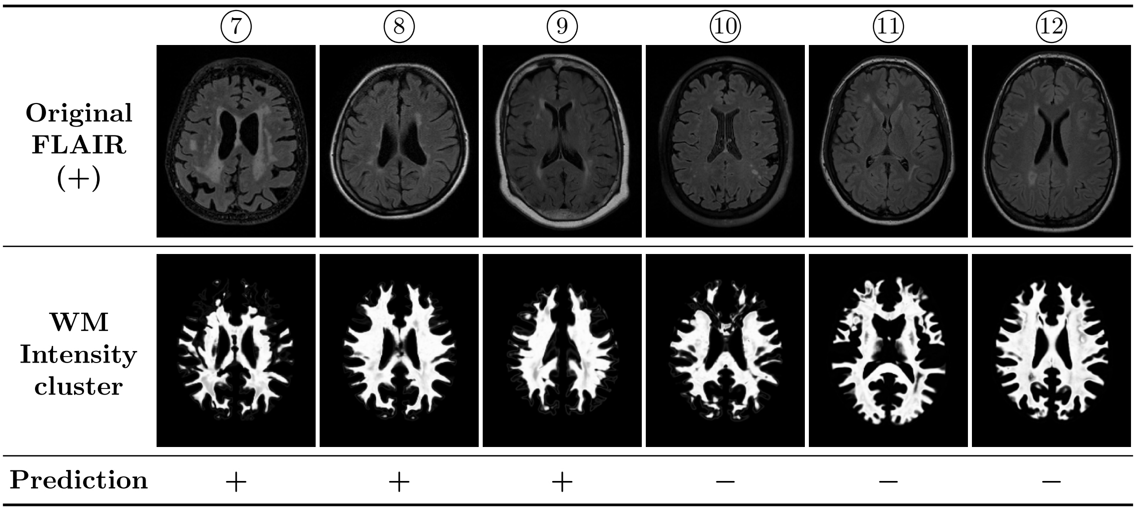

Figure 4: Example of six MRIs (⑦ to ⑫) *with* WM abnormality. Top: original FLAIR images, Middle: the obtained intensity clusters used for testing the model, and Bottom: the prediction of the model in setting $A00$ for the presence of WM abnormalities (+: with WM abnormality, −: without WM abnormality). This figure shows three cases of true positive and three cases of false negative. The WM intensity cluster slice does not exactly correspond to the original MRI slice due to deformations from non-linear registration and only one of the three WM parts being present in this cluster. MRIs source: ⑦,⑩:[81], ⑧:[78], ⑨:[80], ⑪,⑫:[74].

4.2 Setting *B*

The setting B which uses an equal number of MRIs from each MRI protocol is designed to make the prediction task more challenging for the model. Setting $B00$ has an average accuracy of 88.6%, as shown in Fig. 5. In terms of the amount of training data, setting $A07$ is the closest match to $B00$. While $B00$ includes 50 training and 10 validation data, $A07$ has a comparable setup with 52 training and 20 validation data. However, setting B is a more challenging scenario than setting A because it includes an equal number of MRIs from each MRI protocol in the training data. Despite this challenge, the accuracy of $B00$ is only 3.6% less than that of $A07$. This shows the high independency of the presented MRI classification approach on the acquisition protocol of the FLAIR image. With further decrease in the training data, in settings $B01$ to $B04$, all metrics show a gradual decrease in value.

4.3 Setting *C*

The evaluation of setting C is of more importance since it is very close to the real-world use of such a model. In this setting, the MRI protocols of the test set are not present in the training data. It resembles a situation in which a clinical center has heterogeneous MRI data and wants to train a classification model with them. Then the model is supposed to classify new MRIs brought by new patients from other centers, acquired most probably with MRI protocols different from those in the training data.

It is noteworthy that in setting C only 3D MRIs (with 192 or more layers) are included in training and test data, as discussed in the next paragraph. Setting $C00$, as reported in Fig. 6, shows an average accuracy of 94.9% with 64 MRIs used for training (including validation), despite being tested with MRI protocols that were unseen during training. By reducing the data to 46, in $C02$, the model shows an accu-

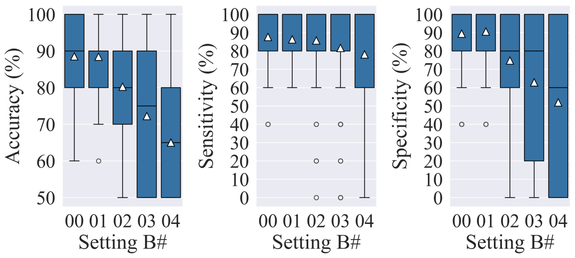

(a) (b) (c)

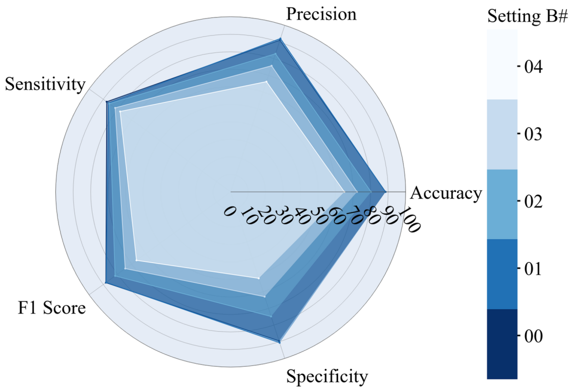

(d)

**Setting**	$B00$	$B01$	$B02$	$B03$	$B04$
$\boldsymbol{MLcps}\%$	76	76	61	48	37

(e)

Figure 5: Classification results of settings $B00$ to $B04$: (a) accuracy, (b) sensitivity, (c) specificity, (d) radar plot of five classification metrics for different setting #s, and (e) $MLcps\%$ (a cumulative performance score) in % for each setting #. In each setting # (i.e. $B00$, $B01$, . . ., $B04$) the training set size is sequentially reduced relative to the previous setting, as detailed in Supplementary Table S 2. In setting B, an equal number from 10 different MRI protocols is used for the model. Average accuracy starts at 88.5% for $B00$, where training includes 60 MRIs, and remains above 80% until $B02$, with 40 training data, after which it decreases gradually with further reductions in training data. A similar trend is observed in the $MLcps\%$.

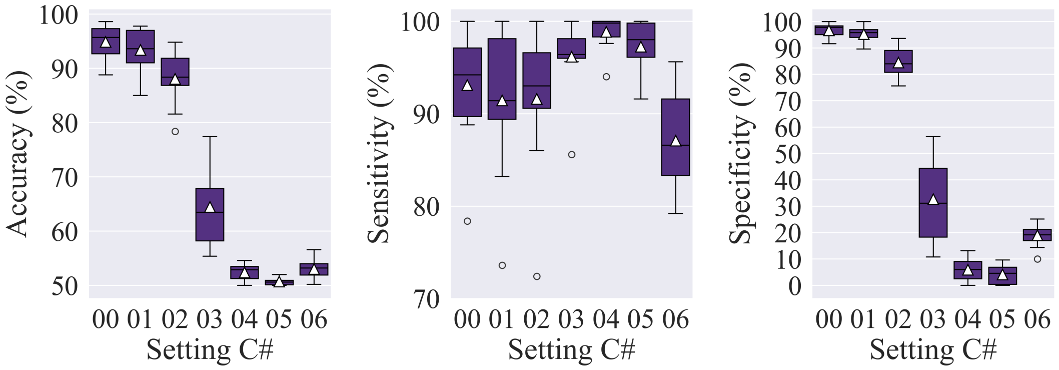

(a) Accuracy (b) Sensitivity (c) Specificity

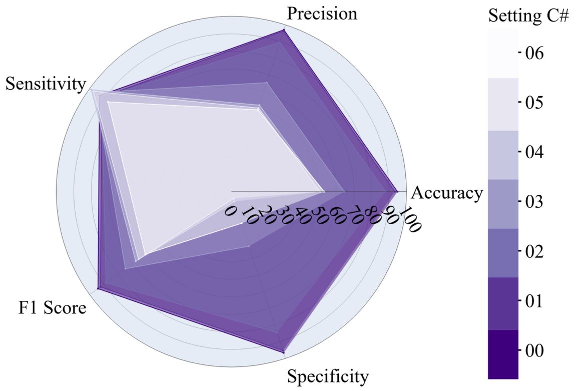

(d) Radar plot

**Setting**	$C00$	$C01$	$C02$	$C03$	$C04$
$\boldsymbol{MLcps}$%	89	86	75	38	25
**Setting**	$C05$	$C06$			
$\boldsymbol{MLcps}$%	24	23			

(e) $MLcps$% values (in %)

Figure 6: Classification results of settings $C00$ to $C06$: (a) accuracy, (b) sensitivity, (c) specificity, (d) radar plot of five classification metrics for different setting #s, and (e) $MLcps$% (a cumulative performance score) in % for each setting #. In each setting # (i.e. $C00$, $C01$, ..., $C06$) the training set size is sequentially reduced relative to the previous setting, as detailed in Supplementary Table S 3. In setting C, the MRI protocols of the test set are unseen by the model during training. Average accuracy starts at 94.9% for $C00$, where training includes 64 MRIs, and remains above 88% until $C02$, with 46 training data, after which it drops sharply with further reductions in training data. A similar trend is observed in the $MLcps$%.

racy of 88.1%. By further decreasing the data to 36, the model's performance drops to 64.4% accuracy. In a nutshell, by training the model with only 46 3D MRIs, it is able to classify MRIs acquired with protocols unseen during training with 88.1% accuracy.

Initially, we used the same data as setting B for setting C. However, the model showed a lack of robustness when the MRI protocols used as test data were changed. To investigate possible sources of this issue, we first used *only* 2D MRIs (with 70 or fewer layers) for the model, but it was not robust. Subsequently, we trained and tested the model by excluding 2D MRIs entirely. Remarkably, the performance notably improved, as reported in setting C results, proving the adverse effect of 2D MRIs.

4.4 Setting D

Setting D, in which the number of protocols was increased in each setting # while maintaining the same data size (82 MRIs for training), shows the slight negative effect of having higher numbers of protocols, as observed by the overall decrease in accuracy, sensitivity, and specificity (Fig.7). In the setting with the

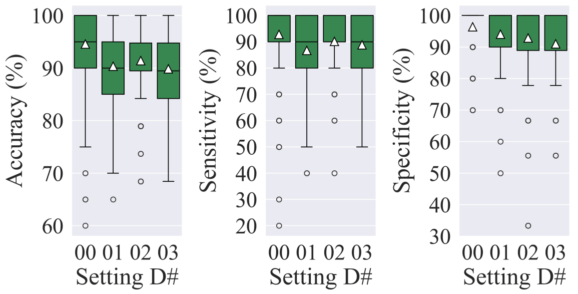

(a) Accuracy (b) Sensitivity (c) Specificity

Figure 7: Classification results of settings $D00$ to $D03$: (a) accuracy, (b) sensitivity, and (c) specificity. In each setting # (i.e. $D00$, $D01$, …, $D03$) the number of MRI protocols is sequentially increased relative to the previous setting while maintaining equal training set size (82 MRIs), as detailed in Supplementary Table S 4. Average accuracy starts at 94.6% for $D00$, where training data includes four different MRI protocols, and ends at 89.8% for $D03$, with 10 MRI protocols in training data.

most diverse protocols, setting $D03$, the model classifies the MRIs with an accuracy of 89.80%.

4.5 Insights on limited data scenarios

A comparison of settings A, B, and C reveals a rough correlation between the number of MRIs in the training data and the performance of the model. A more detailed examination of the impact of reducing training data is presented in Table 2. Regardless of the experimental setting, when the training data (including validation) consists of 40 to 46 MRIs, the accuracy falls within the range of 80% to 88%. Further reducing the training data to the 20 to 36 range is associated with an accuracy of 72% or less and an F1 score of 74% or less. These findings highlight the method's robustness and its sensitivity to the quantity of training data.

5 Conclusion

In this study, we introduce HeteroMRI, a novel approach based on intensity clustering for analyzing MRI datasets that include multiple MRI protocols and scanners to address the challenge of analyzing heterogeneous MRI data. Specifically, we apply this approach to train a binary classification model on FLAIR images, designed to detect the brains that contain WM abnormalities. Notably, our method excels without requiring learning-based MRI harmonization efforts. Our extensive evaluation across diverse experimental settings has shown both the strengths and challenges of the proposed approach.

The results demonstrate the method's robustness in handling a broad spectrum of imaging protocols and scanners in FLAIR MRI data. In other words, the method shows high independence from the scanner and protocol of the MRI data. Importantly, the presented approach proves effective even when the model is tested using MRIs from protocols not present in the training data. Notably, the method also proves to be effective in scenarios with relatively limited data, where only around 50 MRIs are available, considering the classification task studied here. However, despite the promising results of the presented method, it

Table 2: Average performance results of selected experimental settings (in %). The table shows the effect of reducing training data on the model's performance in settings A, B, and C. For these settings, three cases are reported respectively: 1) with the highest number of training data, 2) with the borderline number of training data after which the performance drops, and 3) with the number of training data that results in relatively low performance. Setting D shows the effect of increasing the number of MRI protocols for the same number of MRI data. AUROC: the area under the receiver operating characteristic curve.

**Setting**	**Data size**[1]		**Protocols**[2]		**Accuracy**	**Sensitivity**	**Specificity**	**F1 score**	**AUROC**
	**Train**[†]	**Test**	**Train**[†]	**Test**					
$A00$	200	44	31	14	96.03	95.68	96.39	95.85	97.69
$A07$	72	44	17±1	14	92.17	92.91	91.43	92.23	96.57
$A12$	20	44	6±1	14	72.02	70.82	73.23	67.18	94.03
$B00$	60	10	10	10	88.55	87.60	89.50	87.94	95.64
$B02$	40	10	10	10	80.25	85.70	74.80	81.63	92.37
$B04$	20	10	10	10	65.00	78.10	51.90	66.51	85.02
$C00$	64	10	8	2	94.88	93.08	96.68	94.12	99.50
$C02$	46	10	8	2	88.06	91.60	84.52	88.44	98.10
$C03$	36	10	8	2	64.44	96.16	32.72	74.80	93.18
$D00$	82	20	4	4	94.58	92.75	96.40	93.98	99.29
$D03$	82	20	10	10	89.84	88.80	91.00	90.06	94.72

[1] Number of MRIs used in the training and test sets
[2] Number of MRI protocols present in the training and test data
[†] Including the validation data

is important to note its limitations. Firstly, the current approach was tested with FLAIR images, and evaluation with other MRI sequences relevant to WM abnormalities, such as T2, has not yet been conducted. Secondly, the registration problem with certain MRIs leads to a false prediction by the model, which presents a challenge that needs to be addressed. While a more elaborate registration strategy may reduce the misalignments, this aspect is not the primary focus of the current study. Notably, 2D MRIs have been identified as one of the factors contributing to registration issues, strongly suggesting the use of 3D MRIs for the model. Thirdly, in this study, the intended intensity cluster was chosen manually among the three clusters. This process could potentially be automated by training a classifier model for this task.

This approach paves the way for AI-driven analysis of the abundant MRI data available at medical centers, even when these datasets include data from different protocols and scanners. Additionally, the method is also adaptable to standardized MRI datasets acquired using uniform scanner and protocol settings. Looking ahead, our future research will focus on applying this approach to differentiate between unspecific and disease-associated lesions, as well as to classify rare demyelinating diseases against their differential diagnoses.

6 Availability of Supporting Source Code and Requirements

Project name: HeteroMRI v1.0

Project home page: https://github.com/ul-mds/HeteroMRI

Operating system(s): Linux-based OS (Ubuntu recommended)

Programming language: Python

Other requirements: TensorFlow v2.x, NVIDIA GPU with CUDA support

License: GNU GPL version 3

7 Data Availability

All the datasets used in this study are either publicly available or are accessible upon request to the respective dataset providers as referenced in Table 1.

8 Abbreviations

AI: Artificial Intelligence; ANTs: Advanced Normalization Tools; AUROC: Area Under the Receiver Operating Characteristic Curve; BTH: Baghdad Teaching Hospital; CSF: CerebroSpinal Fluid; CT: Computed Tomography; CNN: Convolutional Neural Network; DL: Deep Learning; TE: Echo Time; FLAIR: Fluid-Attenuated Inversion Recovery; FCM: Fuzzy C-Means; GM: Gray Matter; ICBM: International Consortium for Brain Mapping; TI: Inversion Time; ML: Machine Learning; $MLcps$: Machine Learning Cumulative Performance Score; MRI: Magnetic Resonance Imaging; MNI: Montreal Neurological Institute; MS: Multiple Sclerosis; NifTI: Neuroimaging Informatics Technology Initiative; ReLU: Rectified Linear Unit; TR: Repetition Time; RFCM: Robust Fuzzy C-Means; 3D: three-dimensional; WM: White Matter.

9 Competing Interests

The authors declare that they have no competing interests

10 Authors' Contributions

M.A. (Methodology, Formal Analysis, Software) N.SH. (Conceptualization, Methodology, Writing – Original Draft) P.L.B. (Methodology) N.SC. (Supervision) J.L. (Supervision, Data Curation) C.C.B. (Supervision) W.K. (Supervision, Data Curation, Project Investigation, Funding Acquisition) T.K. (Supervision, Project Investigation, Funding Acquisition) All authors contributed to reviewing and editing the manuscript.

11 Funding

The authors acknowledge the financial support by the Federal Ministry of Health of Germany in project LeukoExpert (grant no. ZMVI1-2520DAT94), the State Ministry for Education and Research of Germany in the project Tag-White (grant no. 100602109), and the Federal Ministry of Education and Research of Germany and by Sächsische Staatsministerium für Wissenschaft, Kultur und Tourismus in the programme Center of Excellence for AI-research "Center for Scalable Data Analytics and Artificial Intelligence Dresden/Leipzig", project identification number: ScaDS.AI.

12 Acknowledgments

The authors sincerely thank Dr. Sina Sadeghi for his insightful comments on the manuscript. The authors are grateful to the Center for Information Services and High-Performance Computing [Zentrum für Informationsdienste und Hochleistungsrechnen (ZIH)] at TU Dresden for providing its facilities for high throughput calculations. Data were provided in part by OASIS Longitudinal Multimodal Neuroimaging: Principal Investigators: T. Benzinger, D. Marcus, J. Morris; NIH P30 AG066444, P50 AG00561, P30 NS09857781, P01 AG026276, P01 AG003991, R01 AG043434, UL1 TR000448, R01 EB009352. AV-45 doses were provided by Avid Radiopharmaceuticals, a wholly owned subsidiary of Eli Lilly. Part of the Data collection and sharing for the Alzheimer's Disease Neuroimaging Initiative (ADNI) is funded by the National Institute on Aging (National Institutes of Health Grant U19 AG024904). The grantee organization is the Northern California Institute for Research and Education. In the past, ADNI has also received funding from the National Institute of Biomedical Imaging and Bioengineering, the Canadian Institutes of Health Research, and private sector contributions through the Foundation for the National Institutes of Health (FNIH) including generous contributions from the following: AbbVie, Alzheimer's Association; Alzheimer's Drug Discovery Foundation; Araclon Biotech; BioClinica, Inc.; Biogen; Bristol-Myers Squibb Company; CereSpir, Inc.; Cogstate; Eisai Inc.; Elan Pharmaceuticals, Inc.; Eli Lilly and Company; EuroImmun; F. Hoffmann-La Roche Ltd and its affiliated company Genentech, Inc.; Fujirebio; GE Healthcare; IXICO Ltd.; Janssen Alzheimer Immunotherapy Research & Development, LLC.; Johnson & Johnson Pharmaceutical Research &Development LLC.; Lumosity; Lundbeck; Merck & Co., Inc.; Meso Scale Diagnostics, LLC.; NeuroRx Research; Neurotrack Technologies; Novartis Pharmaceuticals Corporation; Pfizer Inc.; Piramal Imaging; Servier; Takeda Pharmaceutical Company; and Transition Therapeutics. Part of the data collection and sharing for this project was provided by the International Consortium for Brain Mapping (ICBM; Principal Investigator: John Mazziotta, MD, PhD). ICBM funding was provided by the National Institute of Biomedical Imaging and BioEngineering. ICBM data are disseminated by the Laboratory of Neuro Imaging at the University of Southern California. Part of the data was provided in collaboration with The Observatoire Français de la Sclérose en Plaques (OFSEP), which is supported by a grant provided by the French State and handled by the "Agence Nationale de la Recherche," within the framework of the "Investments for the Future" program, under the reference ANR-10-COHO-002, by the Eugène Devic EDMUS Foundation against multiple sclerosis and by the ARSEP Foundation.

13 Additional Files

Supplementary Table S1. The experimental setting A and details of the MRI data used.

Supplementary Table S2. The experimental setting B and details of the MRI data used.

Supplementary Table S3. The experimental setting C and details of the MRI data used.

Supplementary Table S4. The experimental setting D and details of the MRI data used.

Supplementary Fig. S1. Average required time for preprocessing and intensity clustering of five sample MRI dimensions.

Supplementary Fig. S2. Average required time for training the CNN model of each experimental setting.

References

[1] T. Yousaf, G. Dervenoulas, & M. Politis (2018) *Chapter two - advances in MRI methodology*, M. Politis (Ed.) *Imaging in Movement Disorders: Imaging Methodology and Applications in Parkinson's Disease*, vol. 141 of *International Review of Neurobiology*, pp. 31–76, Academic Press

[2] F. Agosta, S. Galantucci, & M. Filippi (2017) *Advanced magnetic resonance imaging of neurodegenerative diseases*, Neurological Sciences, **38(1)**:pp. 41–51

[3] M. Filippi, P. Preziosa, B. L. Banwell, F. Barkhof, O. Ciccarelli, N. De Stefano, J. J. G. Geurts, F. Paul, D. S. Reich, A. T. Toosy, A. Traboulsee, M. P. Wattjes, T. A. Yousry, A. Gass, C. Lubetzki, B. G. Weinshenker, & M. A. Rocca (2019) *Assessment of lesions on magnetic resonance imaging in multiple sclerosis: practical guidelines*, Brain, **142(7)**:pp. 1858–1875

[4] C. Domínguez-Fernández, J. Egiguren-Ortiz, J. Razquin, M. Gómez-Galán, L. De las Heras-García, E. Paredes-Rodríguez, E. Astigarraga, C. Miguélez, & G. Barreda-Gómez (2023) *Review of technological challenges in personalised medicine and early diagnosis of neurodegenerative disorders*, International Journal of Molecular Sciences, **24(4)**

[5] H.-P. Chan, R. K. Samala, L. M. Hadjiiski, & C. Zhou (2020) *Deep learning in medical image analysis*, G. Lee & H. Fujita (Eds.) *Deep Learning in Medical Image Analysis : Challenges and Applications*, pp. 3–21, Springer International Publishing, Cham

[6] A. S. Panayides, A. Amini, N. D. Filipovic, A. Sharma, S. A. Tsaftaris, A. Young, D. Foran, N. Do, S. Golemati, T. Kurc, K. Huang, K. S. Nikita, B. P. Veasey, M. Zervakis, J. H. Saltz, & C. S. Pattichis (2020) *AI in medical imaging informatics: Current challenges and future directions*, IEEE Journal of Biomedical and Health Informatics, **24(7)**:pp. 1837–1857

[7] B. J. Erickson, P. Korfiatis, Z. Akkus, & T. L. Kline (2017) *Machine learning for medical imaging*, RadioGraphics, **37(2)**:pp. 505–515, pMID: 28212054

[8] M. L. Giger (2018) *Machine learning in medical imaging*, Journal of the American College of Radiology, **15(3, Part B)**:pp. 512–520, data Science: Big Data Machine Learning and Artificial Intelligence

[9] S. Suganyadevi, V. Seethalakshmi, & K. Balasamy (2022) *A review on deep learning in medical image analysis*, International Journal of Multimedia Information Retrieval, **11(1)**:pp. 19–38

[10] M. I. Razzak, S. Naz, & A. Zaib (2018) *Deep learning for medical image processing: Overview, challenges and the future*, N. Dey, A. S. Ashour, & S. Borra (Eds.) *Classification in BioApps: Automation of Decision Making*, pp. 323–350, Springer International Publishing, Cham

[11] N. Garg, M. S. Choudhry, & R. M. Bodade (2023) *A review on Alzheimer's disease classification from normal controls and mild cognitive impairment using structural MR images*, Journal of Neuroscience Methods, **384**:p. 109745

[12] J. Wen, E. Thibeau-Sutre, M. Diaz-Melo, J. Samper-González, A. Routier, S. Bottani, D. Dormont, S. Durrleman, N. Burgos, & O. Colliot (2020) *Convolutional neural networks for classification of Alzheimer's disease: Overview and reproducible evaluation*, Medical Image Analysis, **63**:p. 101694

[13] A. Kursad Poyraz, S. Dogan, E. Akbal, & T. Tuncer (2022) *Automated brain disease classification using exemplar deep features*, Biomedical Signal Processing and Control, **73**:p. 103448

[14] D. García-Lorenzo, S. Francis, S. Narayanan, D. L. Arnold, & D. Louis Collins (2013) *Review of automatic segmentation methods of multiple sclerosis white matter lesions on conventional magnetic resonance imaging*, Medical Image Analysis, **17(1)**:pp. 1–18

[15] O. Cetin, V. Seymen, & U. Sakoglu (2020) *Multiple sclerosis lesion detection in multimodal MRI using simple clustering-based segmentation and classification*, Informatics in Medicine Unlocked, **20**:p. 100409

[16] J. Amin, M. Sharif, M. Yasmin, & S. L. Fernandes (2020) *A distinctive approach in brain tumor detection and classification using MRI*, Pattern Recognition Letters, **139**:pp. 118–127

[17] M. A. Naser & M. J. Deen (2020) *Brain tumor segmentation and grading of lower-grade glioma using deep learning in MRI images*, Computers in Biology and Medicine, **121**:p. 103758

[18] S. Zhang, S. Xu, L. Tan, H. Wang, & J. Meng (2021) *Stroke lesion detection and analysis in MRI images based on deep learning*, Journal of Healthcare Engineering, **2021**:p. 5524769

[19] Y. Kabir, M. Dojat, B. Scherrer, F. Forbes, & C. Garbay (2007) *Multimodal MRI segmentation of ischemic stroke lesions*, *2007 29th Annual International Conference of the IEEE Engineering in Medicine and Biology Society*, pp. 1595–1598

[20] H. Peng, W. Gong, C. F. Beckmann, A. Vedaldi, & S. M. Smith (2021) *Accurate brain age prediction with lightweight deep neural networks*, Medical Image Analysis, **68**:p. 101871

[21] H. Sajedi & N. Pardakhti (2019) *Age prediction based on brain MRI image: A survey*, Journal of Medical Systems, **43(8)**:p. 279

[22] W. M. van Oostveen & E. C. M. de Lange (2021) *Imaging techniques in Alzheimer's disease: A review of applications in early diagnosis and longitudinal monitoring*, International Journal of Molecular Sciences, **22(4)**

[23] R. Zivadinov, J. Sepcic, D. Nasuelli, R. D. Masi, L. M. Bragadin, M. A. Tommasi, S. Zambito-Marsala, R. Moretti, A. Bratina, M. Ukmar, R. S. Pozzi-Mucelli, A. Grop, G. Cazzato, & M. Zorzon (2001) *A longitudinal study of brain atrophy and cognitive disturbances in the early phase of relapsing-remitting multiple sclerosis*, Journal of Neurology, Neurosurgery & Psychiatry, **70(6)**:pp. 773–780

[24] J. West, J. B. M. Warntjes, & P. Lundberg (2012) *Novel whole brain segmentation and volume estimation using quantitative MRI*, European Radiology, **22(5)**:pp. 998–1007

[25] S. Valverde, A. Oliver, E. Roura, S. González-Villà, D. Pareto, J. C. Vilanova, L. Ramió-Torrentà, Àlex Rovira, & X. Lladó (2017) *Automated tissue segmentation of MR brain images in the presence of white matter lesions*, Medical Image Analysis, **35**:pp. 446–457

[26] N. Andrade, F. A. Faria, & F. A. M. Cappabianco (2018) *A practical review on medical image registration: From rigid to deep learning based approaches*, *2018 31st SIBGRAPI Conference on Graphics, Patterns and Images (SIBGRAPI)*, pp. 463–470

[27] J. Kleesiek, G. Urban, A. Hubert, D. Schwarz, K. Maier-Hein, M. Bendszus, & A. Biller (2016) *Deep MRI brain extraction: A 3D convolutional neural network for skull stripping*, NeuroImage, **129**:pp. 460–469

[28] P. Kalavathi & V. B. S. Prasath (2016) *Methods on skull stripping of MRI head scan images—a review*, Journal of Digital Imaging, **29(3)**:pp. 365–379

[29] A. Kaur & G. Dong (2023) *A complete review on image denoising techniques for medical images*, Neural Processing Letters, **55(6)**:pp. 7807–7850

[30] J. Mohan, V. Krishnaveni, & Y. Guo (2014) *A survey on the magnetic resonance image denoising methods*, Biomedical Signal Processing and Control, **9**:pp. 56–69

[31] J. V. Manjón, J. Carbonell-Caballero, J. J. Lull, G. García-Martí, L. Martí-Bonmatí, & M. Robles (2008) *MRI denoising using non-local means*, Medical Image Analysis, **12(4)**:pp. 514–523

[32] M. Shah, Y. Xiao, N. Subbanna, S. Francis, D. L. Arnold, D. L. Collins, & T. Arbel (2011) *Evaluating intensity normalization on MRIs of human brain with multiple sclerosis*, Medical Image Analysis, **15(2)**:pp. 267–282

[33] C. Loizou, M. Pantziaris, I. Seimenis, & C. Pattichis (2009) *Brain MR image normalization in texture analysis of multiple sclerosis*, *2009 9th International Conference on Information Technology and Applications in Biomedicine*, pp. 1–5

[34] N. J. Tustison, B. B. Avants, P. A. Cook, Y. Zheng, A. Egan, P. A. Yushkevich, & J. C. Gee (2010) *N4ITK: Improved N3 bias correction*, IEEE Transactions on Medical Imaging, **29(6)**:pp. 1310–1320

[35] M. Joliot & B. Mazoyer (1993) *Three-dimensional segmentation and interpolation of magnetic resonance brain images*, IEEE Transactions on Medical Imaging, **12(2)**:pp. 269–277

[36] A. Bischoff-Grethe, I. B. Ozyurt, E. Busa, B. T. Quinn, C. Fennema-Notestine, C. P. Clark, S. Morris, M. W. Bondi, T. L. Jernigan, A. M. Dale, G. G. Brown, & B. Fischl (2007) *A technique for the deidentification of structural brain MR images*, Human Brain Mapping, **28(9)**:pp. 892–903

[37] N. De Stefano, M. Battaglini, D. Pareto, R. Cortese, J. Zhang, N. Oesingmann, F. Prados, M. A. Rocca, P. Valsasina, H. Vrenken, C. A. Gandini Wheeler-Kingshott, M. Filippi, F. Barkhof, & Àlex Rovira (2022) *MAGNIMS recommendations for harmonization of MRI data in MS multicenter studies*, NeuroImage: Clinical, **34**:p. 102972

[38] A. J. Hasse (2022) *Quantitative Magnetic Resonance Imaging of Multiple Sclerosis*, Ph.D. thesis, The University of Chicago

[39] G. Mårtensson, D. Ferreira, T. Granberg, L. Cavallin, K. Oppedal, A. Padovani, I. Rektorova, L. Bonanni, M. Pardini, M. G. Kramberger, J.-P. Taylor, J. Hort, J. Snædal, J. Kulisevsky, F. Blanc, A. Antonini, P. Mecocci, B. Vellas, M. Tsolaki, I. Kłoszewska, H. Soininen, S. Lovestone, A. Simmons, D. Aarsland, & E. Westman (2020) *The reliability of a deep learning model in clinical out-of-distribution MRI data: A multicohort study*, Med Image Anal, **66**:p. 101714

[40] R. Shinohara, J. Oh, G. Nair, P. Calabresi, C. Davatzikos, J. Doshi, R. Henry, G. Kim, K. Linn, N. Papinutto, D. Pelletier, D. Pham, D. Reich, W. Rooney, S. Roy, W. Stern, S. Tummala, F. Yousuf, A. Zhu, N. Sicotte, R. Bakshi, & the NAIMS Cooperative (2017) *Volumetric analysis from a harmonized multisite brain MRI study of a single subject with multiple sclerosis*, American Journal of Neuroradiology, **38(8)**:pp. 1501–1509

[41] F. Hu, A. A. Chen, H. Horng, V. Bashyam, C. Davatzikos, A. Alexander-Bloch, M. Li, H. Shou, T. D. Satterthwaite, M. Yu, & R. T. Shinohara (2023) *Image harmonization: A review of statistical and deep learning methods for removing batch effects and evaluation metrics for effective harmonization*, NeuroImage, **274**:p. 120125

[42] M. E. Torbati, D. S. Minhas, C. M. Laymon, P. Maillard, J. D. Wilson, C.-L. Chen, C. M. Crainiceanu, C. S. DeCarli, S. J. Hwang, & D. L. Tudorascu (2023) *MISPEL: A supervised deep learning harmonization method for multi-scanner neuroimaging data*, Medical Image Analysis, **89**:p. 102926

[43] L. G. Nyúl & J. K. Udupa (1999) *On standardizing the MR image intensity scale*, Magnetic Resonance in Medicine, **42(6)**:pp. 1072–1081

[44] R. T. Shinohara, E. M. Sweeney, J. Goldsmith, N. Shiee, F. J. Mateen, P. A. Calabresi, S. Jarso, D. L. Pham, D. S. Reich, & C. M. Crainiceanu (2014) *Statistical normalization techniques for magnetic resonance imaging*, NeuroImage: Clinical, **6**:pp. 9–19

[45] J. Wrobel, M. Martin, R. Bakshi, P. Calabresi, M. Elliot, D. Roalf, R. Gur, R. Gur, R. Henry, G. Nair, J. Oh, N. Papinutto, D. Pelletier, D. Reich, W. Rooney, T. Satterthwaite, W. Stern, K. Prabhakaran, N. Sicotte, R. Shinohara, & J. Goldsmith (2020) *Intensity warping for multisite MRI harmonization*, NeuroImage, **223**:p. 117242

[46] J.-P. Fortin, E. M. Sweeney, J. Muschelli, C. M. Crainiceanu, & R. T. Shinohara (2016) *Removing inter-subject technical variability in magnetic resonance imaging studies*, NeuroImage, **132**:pp. 198–212

[47] J.-P. Fortin, D. Parker, B. Tunç, T. Watanabe, M. A. Elliott, K. Ruparel, D. R. Roalf, T. D. Satterthwaite, R. C. Gur, R. E. Gur, R. T. Schultz, R. Verma, & R. T. Shinohara (2017) *Harmonization of multi-site diffusion tensor imaging data*, NeuroImage, **161**:pp. 149–170

[48] J.-P. Fortin, N. Cullen, Y. I. Sheline, W. D. Taylor, I. Aselcioglu, P. A. Cook, P. Adams, C. Cooper, M. Fava, P. J. McGrath, M. McInnis, M. L. Phillips, M. H. Trivedi, M. M. Weissman, & R. T. Shinohara (2018) *Harmonization of cortical thickness measurements across scanners and sites*, NeuroImage, **167**:pp. 104–120

[49] R. Garcia-Dias, C. Scarpazza, L. Baecker, S. Vieira, W. H. Pinaya, A. Corvin, A. Redolfi, B. Nelson, B. Crespo-Facorro, C. McDonald, D. Tordesillas-Gutiérrez, D. Cannon, D. Mothersill, D. Hernaus,

D. Morris, E. Setien-Suero, G. Donohoe, G. Frisoni, G. Tronchin, J. Sato, M. Marcelis, M. Kempton, N. E. van Haren, O. Gruber, P. McGorry, P. Amminger, P. McGuire, Q. Gong, R. S. Kahn, R. Ayesa-Arriola, T. van Amelsvoort, V. Ortiz-García de la Foz, V. Calhoun, W. Cahn, & A. Mechelli (2020) *Neuroharmony: A new tool for harmonizing volumetric MRI data from unseen scanners*, NeuroImage, **220**:p. 117127

[50] A. Jog, A. Carass, S. Roy, D. L. Pham, & J. L. Prince (2017) *Random forest regression for magnetic resonance image synthesis*, Medical Image Analysis, **35**:pp. 475–488

[51] B. E. Dewey, C. Zhao, J. C. Reinhold, A. Carass, K. C. Fitzgerald, E. S. Sotirchos, S. Saidha, J. Oh, D. L. Pham, P. A. Calabresi, P. C. van Zijl, & J. L. Prince (2019) *Deepharmony: A deep learning approach to contrast harmonization across scanner changes*, Magnetic Resonance Imaging, **64**:pp. 160–170, artificial Intelligence in MRI

[52] B. E. Dewey, L. Zuo, A. Carass, Y. He, Y. Liu, E. M. Mowry, S. Newsome, J. Oh, P. A. Calabresi, & J. L. Prince (2020) *A disentangled latent space for cross-site MRI harmonization*, A. L. Martel, P. Abolmaesumi, D. Stoyanov, D. Mateus, M. A. Zuluaga, S. K. Zhou, D. Racoceanu, & L. Joskowicz (Eds.) *Medical Image Computing and Computer Assisted Intervention – MICCAI 2020*, pp. 720–729, Springer International Publishing, Cham

[53] N. K. Dinsdale, M. Jenkinson, & A. I. Namburete (2021) *Deep learning-based unlearning of dataset bias for MRI harmonisation and confound removal*, NeuroImage, **228**:p. 117689

[54] S. Liu & P.-T. Yap (2024) *Learning multi-site harmonization of magnetic resonance images without traveling human phantoms*, Communications Engineering, **3(1)**:p. 6

[55] L. Zuo, B. E. Dewey, Y. Liu, Y. He, S. D. Newsome, E. M. Mowry, S. M. Resnick, J. L. Prince, & A. Carass (2021) *Unsupervised MR harmonization by learning disentangled representations using information bottleneck theory*, NeuroImage, **243**:p. 118569

[56] F. Hu, A. Lucas, A. A. Chen, K. Coleman, H. Horng, R. W. Ng, N. J. Tustison, K. A. Davis, H. Shou, M. Li, R. T. Shinohara, & T. A. D. N. Initiative (2023) *Deepcombat: A statistically motivated, hyperparameter-robust, deep learning approach to harmonization of neuroimaging data*, bioRxiv

[57] L. L. Resende, A. R. B. de Paiva, F. Kok, C. da Costa Leite, & L. T. Lucato (2019) *Adult leukodystrophies: a step-by-step diagnostic approach*, Radiographics, **39(1)**:pp. 153–168

[58] X. Tu, J. Gao, C. Zhu, J.-Z. Cheng, Z. Ma, X. Dai, & M. Xie (2016) *MR image segmentation and bias field estimation based on coherent local intensity clustering with total variation regularization*, Medical & Biological Engineering & Computing, **54(12)**:pp. 1807–1818

[59] D. Kumar, R. K. Agrawal, & P. Kumar (2022) *Bias-corrected intuitionistic fuzzy c-means with spatial neighborhood information approach for human brain MRI image segmentation*, IEEE Transactions on Fuzzy Systems, **30(3)**:pp. 687–700

[60] A. Khosravanian, M. Rahmanimanesh, P. Keshavarzi, & S. Mozaffari (2021) *Fast level set method for glioma brain tumor segmentation based on superpixel fuzzy clustering and lattice boltzmann method*, Computer Methods and Programs in Biomedicine, **198**:p. 105809

[61] L. Szilágyi, S. M. Szilágyi, B. Benyó, & Z. Benyó (2011) *Intensity inhomogeneity compensation and segmentation of MR brain images using hybrid c-means clustering models*, Biomedical Signal Processing and Control, **6(1)**:pp. 3–12, biomedical signal processing(Extended selected papers from the 7th IFAC Symposium on Modelling and Control in Biomedical Systems(MCBMS'09))

[62] V. Fonov, A. Evans, R. McKinstry, C. Almli, & D. Collins (2009) *Unbiased nonlinear average age-appropriate brain templates from birth to adulthood*, NeuroImage, **47**:p. S102, organization for Human Brain Mapping 2009 Annual Meeting

[63] V. Fonov, A. C. Evans, K. Botteron, C. R. Almli, R. C. McKinstry, & D. L. Collins (2011) *Unbiased average age-appropriate atlases for pediatric studies*, NeuroImage, **54(1)**:pp. 313–327

[64] D. L. Pham (2001) *Spatial models for fuzzy clustering*, Computer Vision and Image Understanding, **84(2)**:pp. 285–297

[65] X. Li, P. S. Morgan, J. Ashburner, J. Smith, & C. Rorden (2016) *The first step for neuroimaging data analysis: DICOM to NIfTI conversion*, Journal of Neuroscience Methods, **264**:pp. 47–56

[66] B. Lowekamp, D. Chen, L. Ibanez, & D. Blezek (2013) *The design of SimpleITK*, Frontiers in Neuroinformatics, **7**:p. 45

[67] J. C. Mazziotta, A. W. Toga, A. Evans, P. Fox, & J. Lancaster (1995) *A probabilistic atlas of the human brain: Theory and rationale for its development: The International Consortium for Brain Mapping (ICBM)*, NeuroImage, **2(2, Part A)**:pp. 89–101

[68] B. B. Avants, N. Tustison, G. Song, et al. (2009) *Advanced normalization tools (ANTs)*, Insight j, **2(365)**:pp. 1–35

[69] J. M. Huntenburg, C. J. Steele, & P.-L. Bazin (2018) *Nighres: processing tools for high-resolution neuroimaging*, GigaScience, **7(7)**:p. giy082

[70] H. Zunair, A. Rahman, N. Mohammed, & J. P. Cohen (2020) *Uniformizing techniques to process CT scans with 3D CNNs for tuberculosis prediction*, I. Rekik, E. Adeli, S. H. Park, & M. d. C. Valdés Hernández (Eds.) *Predictive Intelligence in Medicine*, pp. 156–168, Springer International Publishing, Cham

[71] D. Maturana & S. Scherer (2015) *Voxnet: A 3D convolutional neural network for real-time object recognition*, *2015 IEEE/RSJ International Conference on Intelligent Robots and Systems (IROS)*, pp. 922–928

[72] S. Ioffe & C. Szegedy (2015) *Batch normalization: Accelerating deep network training by reducing internal covariate shift*, F. Bach & D. Blei (Eds.) *Proceedings of the 32nd International Conference on Machine Learning*, vol. 37 of *Proceedings of Machine Learning Research*, pp. 448–456, PMLR, Lille, France

[73] D. P. Kingma & J. Ba (2017) *Adam: A method for stochastic optimization*, arXiv preprint arXiv:1412.6980

[74] A. Carass, S. Roy, A. Jog, J. L. Cuzzocreo, E. Magrath, A. Gherman, J. Button, J. Nguyen, F. Prados, C. H. Sudre, M. Jorge Cardoso, N. Cawley, O. Ciccarelli, C. A. Wheeler-Kingshott, S. Ourselin, L. Catanese, H. Deshpande, P. Maurel, O. Commowick, C. Barillot, X. Tomas-Fernandez, S. K.

Warfield, S. Vaidya, A. Chunduru, R. Muthuganapathy, G. Krishnamurthi, A. Jesson, T. Arbel, O. Maier, H. Handels, L. O. Iheme, D. Unay, S. Jain, D. M. Sima, D. Smeets, M. Ghafoorian, B. Platel, A. Birenbaum, H. Greenspan, P.-L. Bazin, P. A. Calabresi, C. M. Crainiceanu, L. M. Ellingsen, D. S. Reich, J. L. Prince, & D. L. Pham (2017) *Longitudinal multiple sclerosis lesion segmentation: Resource and challenge*, NeuroImage, **148**:pp. 77–102

[75] Ž. Lesjak, A. Galimzianova, A. Koren, M. Lukin, F. Pernuš, B. Likar, & Ž. Špiclin (2018) *A novel public MR image dataset of multiple sclerosis patients with lesion segmentations based on multi-rater consensus*, Neuroinformatics, **16**:pp. 51–63

[76] O. Commowick, A. Istace, M. Kain, B. Laurent, F. Leray, M. Simon, S. C. Pop, P. Girard, R. Améli, J.-C. Ferré, A. Kerbrat, T. Tourdias, F. Cervenansky, T. Glatard, J. Beaumont, S. Doyle, F. Forbes, J. Knight, A. Khademi, A. Mahbod, C. Wang, R. McKinley, F. Wagner, J. Muschelli, E. Sweeney, E. Roura, X. Lladó, M. M. Santos, W. P. Santos, A. G. Silva-Filho, X. Tomas-Fernandez, H. Urien, I. Bloch, S. Valverde, M. Cabezas, F. J. Vera-Olmos, N. Malpica, C. Guttmann, S. Vukusic, G. Edan, M. Dojat, M. Styner, S. K. Warfield, F. Cotton, & C. Barillot (2018) *Objective evaluation of multiple sclerosis lesion segmentation using a data management and processing infrastructure*, Scientific Reports, **8(1)**:p. 13650

[77] O. Commowick, F. Cervenansky, F. Cotton, & M. Dojat (Eds.) (2021) *MSSEG-2 challenge proceedings: Multiple sclerosis new lesions segmentation challenge using a data management and processing infrastructure*, Strasbourg, France

[78] A. M. Muslim, S. Mashohor, G. A. Gawwam, R. Mahmud, M. binti Hanafi, O. Alnuaimi, R. Josephine, & A. D. Almutairi (2022) *Brain MRI dataset of multiple sclerosis with consensus manual lesion segmentation and patient meta information*, Data in Brief, **42**:p. 108139

[79] R. Kötter, J. Mazziotta, A. Toga, A. Evans, P. Fox, J. Lancaster, K. Zilles, R. Woods, T. Paus, G. Simpson, B. Pike, C. Holmes, L. Collins, P. Thompson, D. MacDonald, M. Iacoboni, T. Schormann, K. Amunts, N. Palomero-Gallagher, S. Geyer, L. Parsons, K. Narr, N. Kabani, G. L. Goualher, D. Boomsma, T. Cannon, R. Kawashima, & B. Mazoyer (2001) *A probabilistic atlas and reference system for the human brain: International Consortium for Brain Mapping (ICBM)*, Philosophical Transactions of the Royal Society of London. Series B: Biological Sciences, **356(1412)**:pp. 1293–1322

[80] P. J. LaMontagne, T. L. Benzinger, J. C. Morris, S. Keefe, R. Hornbeck, C. Xiong, E. Grant, J. Hassenstab, K. Moulder, A. G. Vlassenko, M. E. Raichle, C. Cruchaga, & D. Marcus (2019) *OASIS-3: Longitudinal neuroimaging, clinical, and cognitive dataset for normal aging and alzheimer disease*, medRxiv

[81] C. R. Jack Jr., M. A. Bernstein, N. C. Fox, P. Thompson, G. Alexander, D. Harvey, B. Borowski, P. J. Britson, J. L. Whitwell, C. Ward, A. M. Dale, J. P. Felmlee, J. L. Gunter, D. L. Hill, R. Killiany, N. Schuff, S. Fox-Bosetti, C. Lin, C. Studholme, C. S. DeCarli, G. Krueger, H. A. Ward, G. J. Metzger, K. T. Scott, R. Mallozzi, D. Blezek, J. Levy, J. P. Debbins, A. S. Fleisher, M. Albert, R. Green, G. Bartzokis, G. Glover, J. Mugler, & M. W. Weiner (2008) *The Alzheimer's disease neuroimaging initiative (ADNI): MRI methods*, Journal of Magnetic Resonance Imaging, **27(4)**:pp. 685–691

[82] I. Mérida, J. Jung, S. Bouvard, D. Le Bars, S. Lancelot, F. Lavenne, C. Bouillot, J. Redouté, A. Hammers, & N. Costes (2021) *CERMEP-IDB-MRXFDG: a database of 37 normal adult human brain [^{18}F]FDG PET, T1 and FLAIR MRI, and CT images available for research*, EJNMMI Research, **11(1)**:p. 91

[83] S. Vukusic, R. Casey, F. Rollot, B. Brochet, J. Pelletier, D.-A. Laplaud, J. D. Sèze, F. Cotton, T. Moreau, B. Stankoff, B. Fontaine, F. Guillemin, M. Debouverie, & M. Clanet (2020) *Observatoire Français de la Sclérose en Plaques (OFSEP): A unique multimodal nationwide MS registry in France*, Multiple Sclerosis Journal, **26(1)**:pp. 118–122, pMID: 30541380

[84] C. Confavreux, D. A. Compston, O. R. Hommes, W. I. McDonald, & A. J. Thompson (1992) *EDMUS, a European database for multiple sclerosis.*, Journal of Neurology, Neurosurgery & Psychiatry, **55(8)**:pp. 671–676

[85] A. Akshay, M. Abedi, N. Shekarchizadeh, F. C. Burkhard, M. Katoch, A. Bigger-Allen, R. M. Adam, K. Monastyrskaya, & A. Hashemi Gheinani (2023) *MLcps: machine learning cumulative performance score for classification problems*, GigaScience, **12**:p. giad108

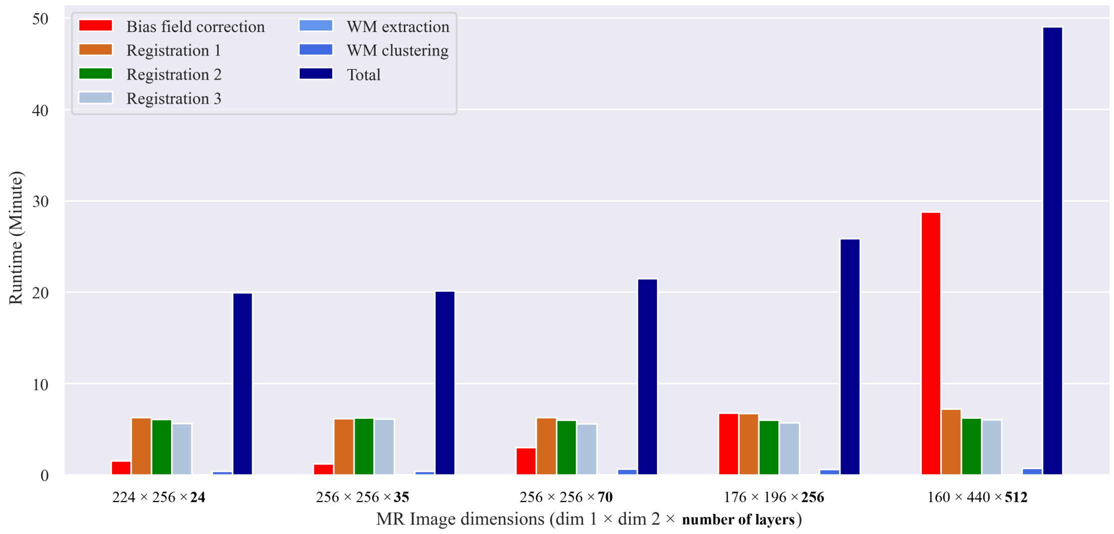

Supplementary Figure S 1: Average required time for preprocessing and intensity clustering of five sample MRI dimensions.

Supplementary Table S 1: Number of MRIs used for training (Tr), validation (V), and test (T) sets from each *dataset* in experimental settings $A00$ to $A18$

**Dataset**		**Set**	**Setting $A\#$**																		
**Label***	**Name**		**00**	**01**	**02**	**03**	**04**	**05**	**06**	**07**	**08**	**09**	**10**	**11**	**12**	**13**	**14**	**15**	**16**	**17**	**18**
+	ISBI	Tr	8	7	6	5	4	3	2	1	0	0	0	0	0	0	0	0	0	0	0
		V	1	1	1	1	1	1	1	1	0	0	0	0	0	0	0	0	0	0	0
		T	2	2	2	2	2	2	2	2	2	2	2	2	2	2	2	2	2	2	2
	UMCL	Tr	8	7	6	5	4	3	2	1	0	0	0	0	0	0	0	0	0	0	0
		V	1	1	1	1	1	1	1	1	0	0	0	0	0	0	0	0	0	0	0
		T	2	2	2	2	2	2	2	2	2	2	2	2	2	2	2	2	2	2	2
	MSSEG	Tr	8	7	6	5	4	3	2	1	0	0	0	0	0	0	0	0	0	0	0
		V	1	1	1	1	1	1	1	1	0	0	0	0	0	0	0	0	0	0	0
		T	2	2	2	2	2	2	2	2	2	2	2	2	2	2	2	2	2	2	2
	MSSEG-2	Tr	8	7	6	5	4	3	2	1	0	0	0	0	0	0	0	0	0	0	0
		V	1	1	1	1	1	1	1	1	0	0	0	0	0	0	0	0	0	0	0
		T	2	2	2	2	2	2	2	2	2	2	2	2	2	2	2	2	2	2	2
	BTH	Tr	6	5	4	3	2	1	0	0	0	0	0	0	0	0	0	0	0	0	0
		V	1	1	1	1	1	1	0	0	0	0	0	0	0	0	0	0	0	0	0
		T	2	2	2	2	2	2	2	2	2	2	2	2	2	2	2	2	2	2	2
	OASIS-3	Tr	10	9	8	7	6	5	4	3	2	1	0	0	0	0	0	0	0	0	0
		V	2	2	2	1	1	1	1	1	1	1	0	0	0	0	0	0	0	0	0
		T	2	2	2	2	2	2	2	2	2	2	2	2	2	2	2	2	2	2	2
	ADNI	Tr	39	35	31	28	26	23	21	19	17	14	12	9	7	6	5	4	3	2	1
		V	6	6	6	6	6	6	5	5	5	5	4	4	3	3	3	2	2	1	1
		T	10	10	10	10	10	10	10	10	10	10	10	10	10	10	10	10	10	10	10
−	ICBM	Tr	3	2	1	0	0	0	0	0	0	0	0	0	0	0	0	0	0	0	0
		V	1	1	1	0	0	0	0	0	0	0	0	0	0	0	0	0	0	0	0
		T	1	1	1	1	1	1	1	1	1	1	1	1	1	1	1	1	1	1	1
	OASIS-3	Tr	65	58	51	45	39	32	26	21	15	12	10	8	7	6	5	4	3	2	1
		V	9	9	9	9	9	9	8	8	5	5	3	3	3	3	3	2	2	1	1
		T	16	16	16	16	16	16	16	16	16	16	16	16	16	16	16	16	16	16	16
	CERMEP	Tr	19	17	15	13	11	9	7	5	4	3	2	1	0	0	0	0	0	0	0
		V	3	3	3	3	3	3	2	2	1	1	1	1	0	0	0	0	0	0	0
		T	5	5	5	5	5	5	5	5	5	5	5	5	5	5	5	5	5	5	5
+	Total	Tr	87	77	67	58	50	41	33	26	19	15	12	9	7	6	5	4	3	2	1
		V	13	13	13	12	12	12	10	10	6	6	4	4	3	3	3	2	2	1	1
		T	22	22	22	22	22	22	22	22	22	22	22	22	22	22	22	22	22	22	22
−	Total	Tr	87	77	67	58	50	41	33	26	19	15	12	9	7	6	5	4	3	2	1
		V	13	13	13	12	12	12	10	10	6	6	4	4	3	3	3	2	2	1	1
		T	22	22	22	22	22	22	22	22	22	22	22	22	22	22	22	22	22	22	22
**Total**			244	224	204	184	168	150	130	116	94	86	76	70	64	62	60	56	54	50	48

* With (+) and without (−) WM abnormality

Supplementary Table S 2: Number of MRIs used for training (Tr), validation (V), and test (T) sets from each MRI acquisition *protocol* in experimental settings $B00$ to $B04$

**Protocol**		**Set**	**Setting**				
**Label***	**Name**		$B00$	$B01$	$B02$	$B03$	$B04$
+	Sie_Tri_30_Prot1	Tr	5	4	3	2	1
		V	1	1	1	1	1
		T	1	1	1	1	1
	Phi_Ing_30_NA	Tr	5	4	3	2	1
		V	1	1	1	1	1
		T	1	1	1	1	1
	Phi_NA_30_Prot1	Tr	5	4	3	2	1
		V	1	1	1	1	1
		T	1	1	1	1	1
	Sie_Aer_15_Prot1	Tr	5	4	3	2	1
		V	1	1	1	1	1
		T	1	1	1	1	1
	GeE_Dis_30_Prot3	Tr	5	4	3	2	1
		V	1	1	1	1	1
		T	1	1	1	1	1
−	Sie_Bio_30_Prot1	Tr	5	4	3	2	1
		V	1	1	1	1	1
		T	1	1	1	1	1
	Sie_Son_15_Prot1	Tr	5	4	3	2	1
		V	1	1	1	1	1
		T	1	1	1	1	1
	Sie_MaV_30_Prot2	Tr	5	4	3	2	1
		V	1	1	1	1	1
		T	1	1	1	1	1
	Sie_MaV_30_Prot1	Tr	5	4	3	2	1
		V	1	1	1	1	1
		T	1	1	1	1	1
	Sie_TrT_30_Prot2	Tr	5	4	3	2	1
		V	1	1	1	1	1
		T	1	1	1	1	1
+	Total	Tr	25	20	15	10	5
		V	5	5	5	5	5
		T	5	5	5	5	5
−	Total	Tr	25	20	15	10	5
		V	5	5	5	5	5
		T	5	5	5	5	5
**Total**			70	60	50	40	30

* With (+) and without (−) WM abnormality

Supplementary Table S 3: Number of MRIs used for training (Tr), validation (V), and test (T) sets from each MRI acquisition *protocol* in experimental settings $C00$ to $C06$. The protocols highlighted in gray are used only as test data. The protocols chosen as test data are changed in 10 different cases.

**Protocol**		**Set**	**Setting**						
**Label***	**Name**		$C00$	$C01$	$C02$	$C03$	$C04$	$C05$	$C06$
+	Phi_Ing_30_NA	Tr	7	6	5	4	3	2	1
		V	1	1	1	1	1	1	1
		T	0	0	0	0	0	0	0
	GeE_Dis_30_Prot3	Tr	7	6	5	4	3	2	1
		V	1	1	1	1	1	1	1
		T	0	0	0	0	0	0	0
	Sie_Pri_30_Prot1	Tr	6	5	4	3	2	1	0
		V	2	2	2	1	1	1	0
		T	0	0	0	0	0	0	0
	Sie_Ver_30_Prot1	Tr	6	5	4	3	2	1	0
		V	2	1	1	1	1	1	0
		T	0	0	0	0	0	0	0
	Phi_Ing_30_Prot2	Tr	0	0	0	0	0	0	0
		V	0	0	0	0	0	0	0
		T	5	5	5	5	5	5	5
−	Sie_MaV_30_Prot1	Tr	6	5	4	3	2	1	0
		V	1	1	1	1	1	1	0
		T	0	0	0	0	0	0	0
	Sie_Son_15_Prot1	Tr	20	17	14	11	8	5	2
		V	5	4	4	3	3	3	2
		T	0	0	0	0	0	0	0
	Sie_TrT_30_Prot1	Tr	0	0	0	0	0	0	0
		V	0	0	0	0	0	0	0
		T	5	5	5	5	5	5	5
+	Total	Tr	26	22	18	14	10	6	2
		V	6	5	5	4	4	4	2
		T	5	5	5	5	5	5	5
−	Total	Tr	26	22	18	14	10	6	2
		V	6	5	5	4	4	4	2
		T	5	5	5	5	5	5	5
**Total**			74	64	56	46	38	30	18

* With (+) and without (−) WM abnormality

Supplementary Table S 4: Number of MRIs used for training (Tr), validation (V), and test (T) sets from each MRI acquisition *protocol* in experimental settings $D00$ to $D03$

**Protocol**		**Set**	**Setting**			
**Label***	**Name**		$D00$	$D01$	$D02$	$D03$
+	Sie_Tri_30_Prot1	Tr	21	12	9	8
		V	3	2	2	1
		T	6	3	2	2
	Phi_Ing_30_NA	Tr	15	12	9	8
		V	2	2	1	1
		T	4	3	3	2
	Phi_NA_30_Prot1	Tr	0	12	9	7
		V	0	1	1	1
		T	0	4	3	2
	Sie_Aer_15_Prot1	Tr	0	0	9	7
		V	0	0	1	1
		T	0	0	2	2
	GeE_Dis_30_Prot3	Tr	0	0	0	6
		V	0	0	0	1
		T	0	0	0	2
−	Sie_Bio_30_Prot1	Tr	21	124	11	9
		V	3	2	2	1
		T	6	3	2	3
	Sie_Son_15_Prot1	Tr	15	12	10	8
		V	2	2	1	1
		T	4	3	4	3
	Sie_MaV_30_Prot2	Tr	0	12	10	9
		V	0	1	1	1
		T	0	4	3	2
	Sie_MaV_30_Prot1	Tr	0	0	5	5
		V	0	0	1	1
		T	0	0	1	1
	Sie_TrT_30_Prot2	Tr	0	0	0	5
		V	0	0	0	1
		T	0	0	0	1
+	Total	Tr	36	36	36	36
		V	5	5	5	5
		T	10	10	10	10
−	Total	Tr	36	36	36	36
		V	5	5	5	5
		T	10	10	10	10
**Total**			102	102	102	102

* With (+) and without (−) WM abnormality

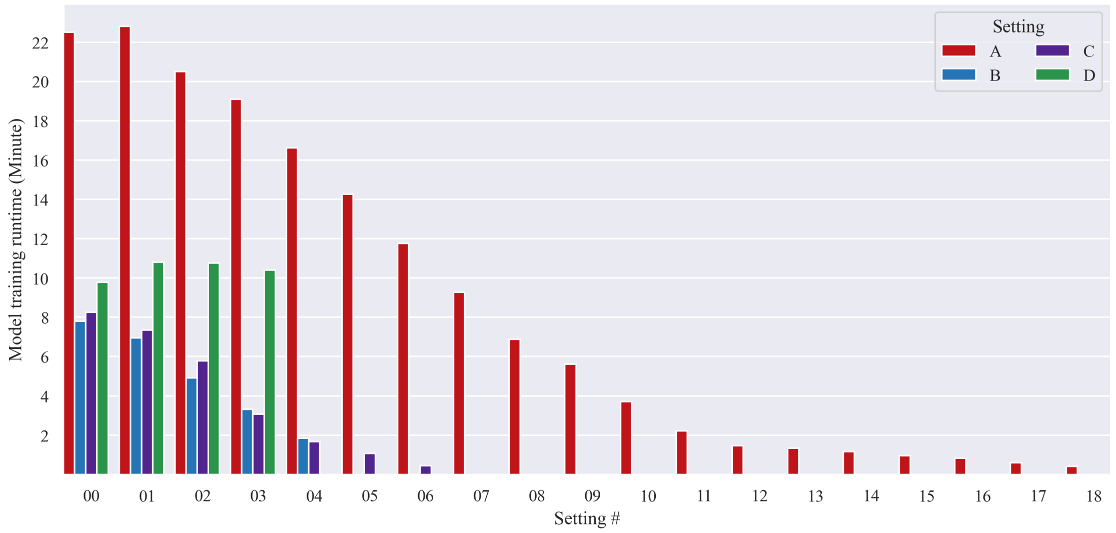

Supplementary Figure S 2: Average required time for training the CNN model of each experimental setting
