# Supplementary material for: HeteroMRI: Robust white matter abnormality classification across multi-scanner MRI data
Source: Gigascience. 2025 Aug 21;14:giaf092. doi: 10.1093/gigascience/giaf092 (PMC12371411; doi:10.1093/gigascience/giaf092)
Supplement: giaf092_GIGA-D-24-00230_Revision_1 [file giaf092_giga-d-24-00230_revision_1.pdf]

GigaScience

HeteroMRI: Robust white matter abnormality classification across multi-scanner MRI data

--Manuscript Draft--

**Manuscript Number:**	GIGA-D-24-00230R1
**Full Title:**	HeteroMRI: Robust white matter abnormality classification across multi-scanner MRI data
**Article Type:**	Research
**Funding Information:**	Bundesministerium für Gesundheit (ZMVI1-2520DAT94) — Mr. Masoud Abedi<br>Bundesministerium für Bildung und Forschung (ScaDS.AI) — Dr. Navid Shekarchizadeh<br>Bundesministerium für Bildung und Forschung (100602109) — Not applicable
**Abstract:**	Background<br>Magnetic Resonance Imaging (MRI) is commonly used for analyzing white matter abnormalities in the human brain. Integrating machine learning into MRI analysis can enhance diagnostic processes. However, the application of such techniques for white matter analysis in clinical practice is often limited when MRI data is multi-scanner (i.e., heterogeneous), particularly in scenarios with limited data, as seen in rare diseases. Therefore, it is crucial to develop methods that are highly independent of the MRI scanner and acquisition protocol.<br>Results<br>This study introduces HeteroMRI, a deep-learning method for classifying MRIs based on white matter abnormalities. Most importantly, HeteroMRI mitigates the effects of data heterogeneity on classification performance. Herein, HeteroMRI is employed to detect brain MRIs with white matter abnormalities. This method utilizes intensity clustering of the white matter tissue to minimize the effects of the heterogeneity of MRIs. MRI data from nine public datasets with 32 MRI protocols is included. By using 200 MRIs for training the model, the binary classifier achieves an average accuracy of 96%. Furthermore, the method is evaluated in limited data scenarios, simulating conditions of rare diseases. By reducing the data by 64% and 75%, the model's accuracy has a 4% and 15% decrease, respectively.<br>Conclusions<br>The presented method opens new avenues for white matter abnormality-related classification of heterogeneous MRI data without additional machine learning methods to minimize MRI heterogeneity. This classification approach demonstrates a high degree of independence from the MRI scanner and protocol, while also proving to be generalizable to unseen MRI protocols.
**Corresponding Author:**	Navid Shekarchizadeh<br>Leipzig University: Universitat Leipzig<br>Leipzig, GERMANY
**Corresponding Author Secondary Information:**	
**Corresponding Author's Institution:**	Leipzig University: Universitat Leipzig
**Corresponding Author's Secondary Institution:**	
**First Author:**	Masoud Abedi
**First Author Secondary Information:**	
**Order of Authors:**	Masoud Abedi<br>Navid Shekarchizadeh<br>Pierre-Louis Bazin

Powered by Editorial Manager® and ProduXion Manager® from Aries Systems Corporation

	Nico Scherf
	Julia Lier
	Christa-Caroline Bergner
	Wolfgang Köhler
	Toralf Kirsten
**Order of Authors Secondary Information:**	

Response to Reviewers:

Dear Dr. Nicole Nogoy,

We are pleased to have the opportunity to revise our manuscript now entitled “HeteroMRI: Robust white matter abnormality classification across multi-scanner MRI data”. In the revised manuscript, we have carefully considered reviewers’ comments and suggestions. The reviewers’ comments were very helpful overall, and we are appreciative of such constructive feedback on our original submission. We reply to each comment in point-by-point fashion. Please find the responses to the concerns raised by both reviewers below. In addition to the revised manuscript, we also provide a version of the manuscript with all the changes marked with respect to the previous version.

At the end, we would like to thank you and the reviewers for their time and thoughtful comments and efforts towards improving our manuscript.

Sincerely,
Navid Shekarchizadeh
On behalf of the authors

##
Reviewer 1
##
Reviewer #1: The manuscript by Abedi et al have proposed a new deep learning method to predict if a given FLAIR image has white matter abnormalities. The CNN-based method looks interesting and seemingly has quite high performance (up to 96%). However, I have a few concerns: there is no comparison analysis presented, the study design prevents any independent assessment, there is no permutation testing, and very limited clinical utility if the method merely gives a binary decision.

Dear Dr. Pravesh Parekh,
We would like to highly thank you for your detailed assessment of our work and your constructive comments. Thanks to your comments and suggestions, the paper has highly improved through the changes that we made. Please find below a point-to-point answer to your comments. Apart from the changes made based on the comments from the reviewers, we also added the following paragraph (and the relevant Supp. Fig.) as we have missed to include it in the previous version of the paper:

“A thresholding is applied on the selected WM intensity cluster of each MRI. All the intensity values below 0.5 are ignored in order to remove uncertain, low-confidence assignments and retain only the core voxels that are strongly associated with the cluster. The value 0.5 is chosen experimentally in the design phase of HeteroMRI by using datasets different from those used in this study for a task of classifying two WM diseases. The value 0.5 resulted in the highest improvement in the classification accuracy compared to other tested thresholds. The histogram for most MRIs follows the same overall pattern: approximately 50∓5% of values are below 0.2, and around 33∓5% are above 0.8. Supplementary Fig. S4 shows the WM cluster of a sample MRI before and after thresholding along with their normalized histograms (for the 99% upper percentile). Finally, the thresholded clusters from the MRIs (one intensity cluster per MRI) are used as training data for the DL model, as described in the following section.”

Powered by Editorial Manager® and ProduXion Manager® from Aries Systems Corporation

Detailed notes below:
Title
1. The title of the paper mentions "classification of multi-scanner and multi-protocol brain magnetic resonance images with deep learning" - however, from this title, it appears that the paper is about differentiating between multiple scanners and protocols. Put another way, the title doesn't specify the classification task at all. It eithers ends up giving an incorrect impression or fails to answer the question "classification of what"? Suggest updating the title to reflect the content better.

Response: Thank you for your insightful comment regarding the title. The title is changed to “HeteroMRI: Robust white matter abnormality classification across multi-scanner MRI data”. Hopefully, it addresses the points that you mentioned.

Abstract
2. "This study introduces HeteroMRI, a deep-learning method for analyzing MRI datasets incorporating various MRI protocols and scanners" - this seems a bit misleading as HeteroMRI is neither a harmonization method nor is it a general purpose analyses method; this should be rephrased to clarify that HeteroMRI only gives binary predictions of WM abnormalities only using FLAIR images.

Response: The sentence is modified as “This study introduces \textit{HeteroMRI}, a deep-learning method for classifying brain MRIs based on white matter abnormalities. Most importantly, HeteroMRI mitigates the effects of data heterogeneity on classification performance. Herein, the presented method is employed to develop a binary classifier that identifies brain MRIs with white matter abnormalities.”

Introduction
3. Trivial point: page 2, line 52, for consistency, better to introduce the abbreviation FLAIR in the bracket, rather than the other way around.

Response: Thank you for the suggestion. It is changed accordingly.

4. Page 3, line 79, the authors mention that "Before analysing brain MRIs, certain preprocessing steps are commonly performed to optimize the image data for further analysis and interpretation"; at the end of the paragraph, the authors mention defacing which is incongruous to the rest of the paragraph - protecting the privacy of individuals using defacing methods is not a step performed for optimizing the images for any analyses or interpretation - it is a separate issue. I suggest removing that line as it doesn't connect with the rest of the text. It is also not something that the authors themselves re-visit at any point in the manuscript (or something they address).

Response: The sentence about defacing is deleted.

5. Page 3, lines 79-83, the authors mention a range of methods that are commonly employed in (pre-)processing of MR data - however, they do not mention image normalization (i.e., transformation from native space to standard space; although one could argue that it is part of the registration step; note that this is different from intensity normalization), image segmentation, and smoothing (although one could argue that it is related to interpolation and denoising).

Response: Thank you for reminding the point. That is right. There might be further MRI preprocessing procedures that we have not included herein. For the sake of avoiding a long introduction, no change is made in the text in this regard.

6. Page 3, line 86, reference 38: perhaps I missed it while looking into the reference, but I did not see a recommendation that images should be acquired "with the same acquisition protocol, with the same magnetic field strength, and preferably with the same MR scanner model" (or "standardized") - in fact, the reference proposes a harmonization method.

Response: Thank you for your question. In fact the recommendation is not directly mentioned in reference 38 however the following statement is mentioned as a limitation

of their work: “There are limitations to this study. In addition to needing all three tissue types (GM, WM, CSF) in each slice for the fit, this study was also performed on a single 3T Philips Ingenia scanner with no changes to protocol (altering TR or TE, slice thickness, etc.).”
We decided to remove this reference from our paper to avoid confusion for the reader.

7. Page 3, line 86, references 37 - 39: I fail to see how the generalization that "it is highly recommended that all the images are standardized" follows from these, where the authors mention that standardized implies same acquisition protocol, same magnetic field strength, and preferably the same MR scanner model.

Response: Please see the response to comment 11

8. Generally, it is true that if the training data and the test data are very different (where differences could originate from different scanner make/models, field strengths, acquisition protocols, etc.), a ML/DL model may fail to generalize. However, that does not imply that "standardization" is the way to go. In other words, while creating a standardized dataset may improve performance in a test scenario, it would never work in a clinical setting (which is what the goal of the paper is).

Response: Yes, completely agreed. As you mentioned correctly, it is the goal of the current paper to perform the analysis with the available heterogenous data since creating a standardized dataset in clinical setting is impractical and even by doing it, one should still make sure if the model is generalizable to the new data in the clinic. We added an essence of your comment to the introduction.

9. "Using a standardized dataset ensures that the images have similar image quality, similar intensity range for each tissue, and similar spatial characteristics." - this is merely a theoretical guarantee; it doesn't really work in practice.

Response: Thank you for spotting the inaccuracy with the sentence. It is revised as follows:
“Using a standardized dataset results in consistent image quality, comparable intensity range for each tissue type, and uniform spatial characteristics, although some variability may still exist.”

10. "Moreover, the contrast among the brain tissues would be uniform across the dataset [40, 37]" - this is not necessarily true; also, doesn't seem to be implied from these citations.

Response: The sentence was removed.

11. The overall issue with this paragraph (page 3, lines 85-94) is that the authors are contextualizing everything as generally true while picking very specific nuanced notes from the field of multiple sclerosis.

Response: Following your comments regarding this paragraph, we rephrased the whole paragraph by changing the way the problem is explained for the reader. As different changes have been made in the introduction, please see the introduction of the revision of the paper for the changes.

12. Page 4, line 114, "The methods based on statistics alter the intensity distribution in the image…" is not entirely accurate - methods like ComBat (references 46-48) are generally meant to be used on features derived from images and not directly the image intensity itself.

Response: Thank you for the correction. The related sentences are revised as follows:
“The proposed MRI harmonization methods in the literature are generally divided into statistical approaches or ML/DL techniques. One group of statistical methods adjusts the intensity distribution, by normalizing the intensity~\cite{SHAH2011267, Nyul1999, SHINOHARA20149, WROBEL2020117242} or by harmonizing values measured from the images (such as cortical thickness) by adjusting the so-called batch effect across different scanners~\cite{FORTIN2016198, FORTIN2017149, FORTIN2018104, Marzi2024, Pomponio2020, RADUA2020116956}”

Powered by Editorial Manager® and ProduXion Manager® from Aries Systems Corporation

13. "In this paper, to circumvent the complex MRI harmonization, we present HeteroMRI, an approach for analyzing multi-protocol and multi-scanner MRI data." - well, HeteroMRI has 1,795,905 parameters and therefore is not simple in anyway. Additionally, this doesn't circumvent harmonization in anyway - perhaps I am missing what the authors really wanted to say here.

Response: Thank you for your detecting the ambiguity in this sentence. What we meant here is that HeteroMRI does not try to harmonize the images itself by modifying them for example through a trained AI model. However, it is true that it reduces the scanner effect through the intensity clustering. We modified the sentence as follows:
"Herein, we present HeteroMRI, an approach for the classification of brain MRIs based on WM abnormalities while mitigating the heterogeneity effects of the images acquired from multiple scanners and acquisition protocols. In this paper, HeteroMRI is utilized to detect brains with WM abnormalities in FLAIR images through binary classification. In future work, the method is intended to be applied to the task of classifying two WM diseases based on their distinct WM abnormality patterns."

14. Overall, I found the introduction section to be very hard to follow. The introduction starts with a brief remark about WM abnormalities and then goes on to talk extensively about other topics (MR preprocessing, use of ML/DL methods, and an extensive discussion about harmonization). However, it is only towards the end of the introduction that the authors return to WM abnormalities and even then, the goals are a bit unclear - is HeteroMRI a way to harmonize the data or is it a way to detect WM abnormalities which is robust to scanner effects? Throughout the introduction, the authors heavily cite literature from MS and other very specific neurological applications but continue to phrase the introduction very generically. In fact, even towards the end, when the authors state "we present HeteroMRI, an approach for analyzing multi-protocol and multi-scanner MRI data" - even at this point it is unclear what this analysis method is supposed to do - it is only a few sentences
later, "Herein, the presented approach is evaluated for detecting the brains with WM abnormalities through binary classification." that it becomes clear that the method is supposed to identify WM abnormalities. This ambiguity is also applicable to the title where the title states the goal being classification but doesn't specify classification of what. I strongly encourage the authors to edit the introduction to have a stronger focus.

Response: Thank you for your comprehensive comment regarding the introduction. We modified the introduction accordingly in order to improve the flow of the text and remove the ambiguities by adding details on the goal of the paper. Please see the introduction for all the changes.

15. Page 5, line 171: "The presented method opens new avenues for analyzing heterogeneous MRI datasets and the large amount of MRI data generated daily in medical centers" - I don't quite see how. HeteroMRI can classify WM abnormalities as yes and no; it is not a general-purpose analyses tool nor is it a harmonization method. Therefore, "the presented method opens new avenues for detecting WM abnormalities in heterogeneous MRI datasets".
Methodology

Response: We appreciate your thoughtful review. It is true that in this paper HeteroMRI is used for detecting the MRIs that have white matter abnormality, however, this paper is meant to serve as publishing the method which is then to be used for other clinical classification tasks in future work, for example for classifying two different white matter-related diseases which have more or less different white matter abnormality patterns in the brain. We have addressed your comment by modifying the sentence as follows:
"The presented method opens new avenues for performing WM abnormality-related analyses on heterogeneous MRI datasets and the large amount of MRI data generated daily in medical centers."
Also, another sentence is added in this regard to clarify our future work and the potential applications of HeteroMRI. Please see the response to your comment 13 for the added sentence.

16. "The WM abnormality detection approach presented in this paper uses

heterogeneous brain MRI data with various acquisition protocols (multi-protocol) as the data for a Convolutional Neural Network (CNN). The model is a binary classifier trained to detect patients with WM abnormalities in their brain MRI." - this is the first place where the authors have very clearly stated what they do and their goal! This clarity, if added back to introduction, will be very useful to the readers.

Response: Thank you for bringing this to our attention. We modify the introduction accordingly to make the goal of the paper more clear.

17. Page 7, line 189, what about the number of voxels in the third dimension?

Response: The information is added to the text as follows:
“All selected images have a minimum of 128, 192, and 22 voxels in their first, second, and third dimensions, respectively.”

18. Page 7, line 192: "Detailed information on the parameters used in each step is reported in the GitHub repository of HeteroMRI" - on the GitHub page [https://github.com/ul-mds/HeteroMRI/tree/main], I do not see any details of what FlexiMRIprep settings / configuration was used for which dataset.

Response: Thank you very much for spotting the missing information also on the GitHub page. The info is added to the repository as follows:
“The following parameters are used in the antsRegistration tool:
--dimensionality 3, --interpolation Linear, --transform SyN[gradientStep=0.1], --metric MI[metricWeight=1, numberOfBins=32] (Mutual Information), --initial-moving-transform [initializationFeature=1] --convergence 500x400x70x30, --shrink-factors 8x4x2x1, --smoothing-sigmas 3x2x1x0vox, --use-histogram-matching 0, --winsorize-image-intensities [0.005,0.995]
and for the rest of the parameters the default values are used.

In fuzzy_cmeans function, the parameter values of `clusters=3, max_iterations=200`, and the default values for all the other parameters are used.
”

19. Does this imply that different processing strategies / settings were used for each dataset? If yes, why? What was differently done for which datasets? Please include these details in the manuscript.

Response: Thank you for the insightful question. All the MRIs undergo the same preprocessing steps. We modified the sentence in the paper as follows:
“The preprocessing steps described below are applied identically to all MRIs from different datasets.”

20. Page 7, line 202: "This template is created using the data from the ICBM (International Consortium for Brain Mapping) project" - does this mean you created your own template? I think not, but please clarify / edit the sentence.

Response: Following your advice, we have changed the text as follow:
“Among the available brain templates, we choose the ICBM 2009c Nonlinear Asymmetric template~\cite{FONOV2009S102, FONOV2011313} (referred to below as the MNI template), which the developers created using the data from the International Consortium for Brain Mapping (ICBM) project~\cite{MAZZIOTTA199589}. This template was selected due to its high accuracy and the availability of the WM probability map required for our analysis approach.”

21. Why was the registration step performed three times - the authors claim that this leads to "most precise alignment" - how was this established/judged?

Response: Thank you for your point regarding the registration. We modified the text accordingly to mention how the number 3 is determined and I agree that it the word “most” is not the best word to be used here. The modified text is as follows:

“A nonlinear registration is applied three times (with identical parameters) on each

Powered by Editorial Manager® and ProduXion Manager® from Aries Systems Corporation

image consecutively. This repeated registration aims to achieve a high level of alignment of the MRIs with the template. The selection of three iterations was determined experimentally to maintain high alignment accuracy while avoiding the higher computational cost of additional nonlinear registrations. Multi-pass registration has also been used by others, for example, to address large differences in the initial positions of image pairs~\cite{Yang2013}."

22. At the end of registration + normalization step, what was the final voxel size and image size?

Response: Thank you for bringing this to our attention. We added the following sentence:
"The registered MRIs all have a size of 193 × 229 × 193 voxels with a voxel size of 1 × 1 × 1 mm."

23. Since some of the dataset had 2D images (many in OASIS, ISBI, and BTH), were they all normalized to some fixed voxel size and overall size?

Response: Yes that is right. All the MRIs were registered to the same template therefore they have an identical image size and voxel size at the end. This is due to the dimension of the input layer of the CNN network which requires all the training data to have a fixed size.

24. From Fig. 1, it appears that all input images were 193 229 193 - that seems like a massive up-sampling for images which only had 22 or so slices. How did the authors arrive at this resolution?

Response: Thank you for the question. That is right. While the 2D MRIs were upscaled to match the fixed 193 229 193 size, many other 3D MRIs were downscaled to this size. This image size is the size of the chosen MNI template. In fact, we did not make any changes to the template, and by registering all the MRIs to the template, they will have the size of the template. Please also see the response to comment 23 in this regard.

25. The WM extraction step is not clear at all - once the FLAIR images are aligned with the MNI template, the voxels still represent some (bias corrected and) interpolated version of the arbitrary MR signal from the raw data. The MNI WM probability map, on the other hand, has values between 0 and 1 showing the probability of that voxel being white matter. A simple multiplication of these two doesn't make sense, given that there will be arbitrary differences between subjects simply because of the scanner intensity scaling differences. The actual voxel intensity per se doesn't have a lot of meaning to it.

Response: Thank you for your question in this regard. What you explained about the mathematics done on the MRI is completely correct. We simply multiply the original WM probability map by the registered MRI. The goal is solely to keep any voxel that "might" be white matter and ignore the rest of the voxels. Of course, the MRIs coming from different scanners still have different orders of voxel values but this is not of importance in this step since the normalization of these numbers is done in the next preprocessing step which is the white matter intensity clustering. We explain further on this step in the response to the comment 27.

Of course, this is true that the brain of each person has a different shape and even a 3-time registration does not make all the brains completely similar. Moreover, multiplying the WM map to it tries to keep the probable WM voxels while ignoring the rest of the voxels. For sure, this approach has some extent of error in extracting the WM which can be considered as a drawback. However, this sacrification of accuracy is with the goal of making use of heterogeneous MRI data, especially in cases of data scarcity such as rare diseases where providing a standardized dataset is impractical.

26. Was the WM probability map binarized?

Response: No, we did not make any changes to the WM probability map.

Powered by Editorial Manager® and ProduXion Manager® from Aries Systems Corporation

27. Since there doesn't seem to be any intensity normalization / correction / rescaling, how does the method account for extremely different intensities between different MRI vendors? For example, for data that comes from a Philips scanner, the intensity values would be very high (provided Philips precise scaling is turned on when converting images from DICOM to NIfTI). This would imply intensity values of tens of thousands while the ones from Siemens would be in a few hundreds (typically). Bias correction would not (completely) account for this (since it is not performed across images but within images).

Response: In fact, the intensity normalization is performed in the intensity clustering section. In this step, the extracted WM is divided into three clusters. The resultant 3 intensity clusters have normalized values between 0 and 1. The clusters are membership files (like probability maps) therefore the sum of values of a voxel in the 3 clusters is equal to 1.
In other words, the intensity clustering step not only calculates the 3 clusters but also normalizes the values. Although this normalization is not performed across images, it serves as a last resort in cases of data scarcity where enough data for training an AI model for harmonization of MRI across all the images is not available.

28. Page 7, line 215: "Calculating and obtaining a …", it is just one operation being performed.

Response: Thank you for your attention. The first verb is removed.

29. Visually, it appears that cluster 3 is merely an eroded version of WM segmentation - did the authors try comparing cluster 3 with an eroded version of WM segmentation from standard neuroimaging tools?

Response: Very interesting question. Following your comment, we checked if cluster 3 is merely similar to the eroded version of the WM segmentation. We performed the following steps on one FLAIR MRI: 1-Skullstripping, 2-FAST segmentation, 3-Eroding the segmented WM
And compared it with cluster 3. It came out that they are only similar to a limited extent but are generally different.

30. "We tested various cluster numbers on different MRIs…" - were these the same dataset that were eventually used for training and test?

Response: Thank you for your question. We tested it on a different dataset (a clinical dataset of a different disease) than the ones used for training and testing since this was done in the initial method design stage in which we were not using the final data of the current paper yet. However, it is noteworthy that coming up with the number of clusters does not really depend on the dataset used since after the intensity clustering of the MRIs of the current paper (with 3 clusters) we noticed merely similar-looking clusters in nearly all data. This shows the independency of the selection of the number of clusters from the dataset.

In the light of your question, we clarified the text in the paper as follows:
“The decision to use three clusters was based on our empirical observations from a dataset different from those used in this study. Through testing various cluster numbers on different MRIs, we found that three clusters yielded consistently comparable patterns in nearly all MRIs. In other words, the shapes of the clusters in one MRI were generally consistent with those in another MRI. This was also later observed in the data of the current study. Therefore, the choice of three clusters is robust and does not depend on a specific dataset.”

31. "For this study, we manually identified the intended cluster for each MRI by choosing the cluster that looks visually similar to Cluster 3 in Fig. 1." - that doesn't sound like a robust strategy and requires manual intervention. This needs to be addressed.

Response: Thank you for pointing out this point. Please see the response to the next comment.

32. I don't think a DL model to identify the right cluster is necessary - at least a visual examination of the three clusters in Fig. 1 indicates that some combination of standard image processing tools and the right evaluation metrics (like Dice coefficient) might solve the problem. Since the authors have already done the manual identification, it should be easy to evaluate any solution for robustly identifying the correct cluster image.

Response: We thank you very much for this brilliant suggestion. Following your suggestion, we used Dice coefficient and tested it on all the 344 MRIs of this study and Dice score could find the correct WM cluster with 100% accuracy. As the reference image for calculating Dice, we used the average of the intended intensity cluster of four MRIs from our clinal dataset which are not among the data of this study. We updated the code and readme on GitHub and the text in paper accordingly. In fact, we change the manual cluster selection to an automatic one using the Dice score. The related paragraph in the paper is changed as following:

“Upon examining the three WM intensity clusters in MRIs with WM abnormalities, we noted that one of the clusters (Cluster 3 in Fig. 1) within each MRI consistently exhibited significant lesion-related features. Therefore, from each MRI, we should take the cluster that looks visually similar to Cluster 3 in Fig.~\ref{fig:steps} but it is not always the cluster number 3. For this purpose, we use the Dice similarity coefficient\cite{Dice} to compare the three clusters of each MRI with a fixed reference cluster to detect the most similar one. The reference image (available in the GitHub repository of HeteroMRI) is generated by averaging the intended intensity cluster of four MRIs from a clinical dataset. This method detected the right cluster for all the MRIs of this study correctly (i.e. with 100\% accuracy) as checked manually.”

33. For the entire DL design - what was the rationale for implementing this specific configuration? In other words, how did the authors arrive at this precise configuration? Some aspects seem standard while others represent an arbitrary choice - was any of this optimized? If yes, using which dataset?

Response: Good question! We began with a CNN architecture recommended on the Keras website(https://keras.io/examples/vision/3D_image_classification/). The code is an example of 3D image classification of CT scans. We used one of our clinical MRI datasets (Leukodystrophy) and we trained the CNN to classify the MRIs (Leukodystrophy versus its differential diagnoses) with the HeteroMRI approach (this will be another publication based on HeterMRI). As the accuracy was not high enough, we tried adding more layers to the network, which helped increase the accuracy. The added layers are the following:

```
x = layers.Conv3D(filters=128, kernel_size=3, activation="relu")(x)
x = layers.MaxPool3D(pool_size=2)(x)
x = layers.BatchNormalization()(x)
```

Considering the GPU memory limitation, which prevents testing the effect of adding more layers, we used this CNN network (with the three new layers added) for the current paper. We observed an even higher accuracy as the classification task of the current paper is easier than differentiating two white-matter diseases. No further optimization of the network was performed due to the costly computations.

34. Table 1: would be additionally helpful (to the readers) to include a hyperlink to the datasets or the page where the dataset can be requested.

Response: Thank you for the suggestion. It is a nice idea however we have noticed that sometimes the webpage address of the datasets changes over time so we refer only to the publication which the reader can use to find out the data access procedure.

35. The MSSEG paper mentions 53 images - why was one image dropped in the current study?

Response: Thank you for noticing this point. Actually, we made an error during the

data preparation and creation of the MRI list and one image of the MSSEG dataset is missing from the list. The missing image is “Patient_10_Center_08_test” which is the last image of the dataset (by sorting the images alphabetically) which has probably been deleted from our MRI list inadvertently. Therefore it is not included in the experiments. We noticed this issue after all the costly computations were finished so we could not help it.

36. ICBM dataset - only five out of 20 did not have WM abnormalities; can't the remaining 15 be used as positive cases (just like in OASIS-3)? Why were they removed?

Response: Good question! Yes, that is right, we could have added them as cases. However, there was already more than enough cases available with a high diversity of scanners, protocols, and WM lesion patterns. Furthermore, if we wanted to increase the number of cases in an experiment (e.g. A00), we did not have more controls to add to keep the labels balanced. The number of controls used in A00 setting was the most number of controls that we were able to provide considering our resources and time limits.

37. For OASIS-3, from a pool of 600 images, only 90 were identified without WM abnormalities; then, how are there only 14 cases with WM abnormalities? Either some detail is missing here or one of the numbers is incorrect - from the document on GitHub, it looks like 90 and 14 are correct - that means there was a massive pool of images with WM abnormalities, but they were not used in the study? Why not?

Response: Thank you for the question. We added the 14 images from OASIS-3 during the design of Setting A since in A00 only 14 more MRIs were necessary to complete this setting by considering the balanced number of cases and controls. However, we simply did not put more images of this dataset in the MRI list in favor of data diversity as the scanner and protocol diversity was already so high among the data. In summary, we had no specific intention or goal for not including more images (cases) of this dataset.

38. ADNI: First, please specify if you examined across ADNI1, ADNI2, ADNI-GO, and ADNI-3? Second, what was the total number of available images? Third, if only 58 images with WM abnormalities were identified, shouldn't there be a good number of control images from ADNI? Why were they not used?

Response: Thank you for noticing the missing information and the question regarding ADNI dataset. The MRIs used in this study are taken randomly from the downloaded pool of FLAIR images from ADNI 3. We changed ADNI to ADNI 3 throughout the paper text. Regarding the second question: By filtering ADNI 3 data by the %FLAIR% in the “Image Description” field, we would get 2401 images from 1147 unique patients. We got the 58 cases from the 235 first patients of the patients' list (by sorting Subject ID ascendingly) by trying to include different WM lesion patterns. Regarding the third question: As the neurologists checked the ADNI images, very limited number of brains were free of WM abnormalities while in OASIS dataset, there was around 15 MRIs per 100 patients. Therefore, it was decided to consider only OASIS (among the two) for finding controls. This decision was made considering the time-consuming task of checking all the slices of hundreds of MRIs. This difference between ADNI and OASIS might be due to the age of the subjects, which may be higher in ADNI, causing most of the brains to have WM abnormalities. However, we did not check this fact specifically.

39. CERMAP: The remaining 10 subjects who were identified as having minor lesions - why were they not used as cases?

Response: Please see the answer to comment 36. The situation is similar.

40. Coming back to BTH: the dataset has 60 2D FLAIR images - however, only 9 were used in the present study. The authors mention a problem with the 2D images; however, many images in the OASIS dataset had similar number of slices: specifically, 8 images with 24 slices and 86 with 35 slices. What was the strategy used for processing the OASIS images and couldn't the same be used for BTH?

Powered by Editorial Manager® and ProduXion Manager® from Aries Systems Corporation

Response: Thank you for noticing this point. This is true that the 2D MRIs of BTH and OASIS datasets have merely a similar number of slices. The difference is about the slice thickness. In OASIS the slices have realistic thicknesses so when you open the raw file, it looks like a normal brain but with low resolution in the coronal and sagittal planes. However, in BTH, all slices have a 1 mm thickness. Therefore, in the raw file, the brain has a much lower height than a normal brain in the coronal and sagittal planes. So it is not possible to directly register the BTH images to the MNI template, unlike the OASIS images. Even increasing the thickness of the slices of BTH images based on the provided metadata file does not result in normal-looking brains for all patients. That is why we chose only images of 9 patients that looked “quite” normal after increasing the slice thickness. Despite the problems with BTH dataset, we preferred to keep it in the study in favor of having higher heterogeneity (protocol diversity) in the study data.

41.   Overall comments for the data section: the rationale for what was included and not, is not specified / is unclear. Please provide more details. There seems to be a large number of cases/controls that could have been included but were seemingly not.

Response: We hope the current version of the paper and the answers to your comments addresses your concerns.

42.   What about the age and sex distribution of the samples from each of the site? These are important variables to consider when evaluating whether there are systematic differences in sites or between cases and controls within a site.

Response: Thank you for your question. We used only public datasets and not all datasets share the demographic data of the subjects. Therefore we cannot consider such variables in the study, unfortunately.

43.   There is no mention of quality checks performed on the images prior to their inclusion in the analysis - this is critical, especially given that the authors later remark about registration / segmentation issues - please provide documentation on what were the QA/QC procedures employed. If these were not done, there needs to be a very good reason why not.

Response: In this regard, our criteria was to include FLAIR images that do not have severe noise or artifacts. Since all the MRIs of this study are from public datasets, apparently the MRIs have been checked once by the dataset providers, therefore we expect no MRI with severe noise or artifact to be present among the data. Furthermore, we created a list (“MRIs_list.csv” on GitHub) of all MRIs with their 3D dimension and voxel size to make sure that there are no images with dimensions less than the minimum values reported in the paper. Regarding registration, It is not completely clear to us which factors result in the registration problem. Otherwise, we would have excluded the MRIs with such factors in the data quality control phase. The only factor we found that sometimes led to incorrect registration was the low number of slices in the 2D MRI. However, one might argue that we could have removed the MRIs with the wrong registration from the study. We refrained from removing such cases since we prefer HeteroMRI to be working without human interference. The goal is to develop a tool (especially for the future applications of HeteroMRI, i.e. other white-matter-related classification tasks) to be used by clinicians therefore we avoid the necessity of technical interference.

44.   For each of the settings: as a first sentence, can you please state the goal of the experiment/setting? It would be helpful for the readers to get oriented in the first sentence itself of the goal of the experiment.

Response: Thank you for your nice suggestion. Following your comment, we added the first sentence for the settings that missed such a sentence, as following:
Setting A:
“In setting A, the goal is to evaluate HeteroMRI on a combination of MRIs from different datasets beginning from a relatively large number of data and then decreasing the data gradually.”
Setting B:

Powered by Editorial Manager® and ProduXion Manager® from Aries Systems Corporation

	"In setting B, the goal is to choose the MRIs with the most diversity of protocols while having an equal number of MRIs from each protocol therefore the model sees the same number of MRIs per protocol."<br>Setting C:<br>"In setting C, the goal is to assess the generalizability of HeteroMRI to unseen MRI protocols."<br><br><br>45. I found supplementary table S1 hard to follow; it was easier to use the Excel sheet from the GitHub repository of the authors - perhaps the first part of the table (where the dataset breakdown is not present) could be additionally included in the supplementary materials?<br><br>Response: We agree with you that understanding the Excel sheet is easier. However, fitting more parts of the table in the supplementary table is challenging as the space is limited in an A4 page. We would expect the interested reader to see the GitHub repository.<br><br>46. Page 11, line 229: "The number of MRIs with and without WM abnormality is balanced in the training data of all the settings." - wasn't this the case for validation and test sets as well (and not just for training)?<br><br>Response: That is right. Thank you for spotting the missing point. The mentioned sentence is combined with the next sentence and it is modified as follows:<br>"The number of MRIs with and without WM abnormality is balanced in the training, validation, and test data of all the settings."<br><br>47. What was the rationale for enforcing equal number of cases and controls in training, validation, and test sets? Was it merely convenience to ensure that training was not biased by class imbalance?<br><br>Response: Thank you for the question. Yes, we always balanced the cases and controls to avoid any bias due to class imbalance, so that the model learned equally from both classes. It was also important because we wanted to test the ability of the model to handle the heterogeneity of the MRI data coming from different scanners and protocols without the additional complexities introduced by class imbalance.<br><br>48. The authors had 222 positive cases and 122 negative cases; in setting A, 100 positive cases were removed - on what basis? And which ones?<br><br>Response: Good question! In each setting and in each shuffle, the code (https://github.com/ul-mds/HeteroMRI/blob/main/select_id_settings_class.py) selects the necessary number of MRIs from the MRI list randomly. The selection is either based on name of dataset or name of the protocol depending on the experimental setting design. For example, in setting A00, 244 MRIs are selected randomly (in this case all 122 controls are taken and 122 random MRIs from the 222 pool of cases are taken). In setting A01 (and further), as the data needs to be decreased, the necessary number of MRIs is removed from the data used for A00 randomly, only with the condition that the highest number of protocols is maintained among the data used in the setting.<br>We add the following sentences to the paper to clarify the point that you mentioned:<br>"The number of images necessary for each setting is selected randomly from the MRIs available."<br>and<br>" The downsizing is performed by removing random MRIs while keeping the maximum possible number of protocols among the data."<br><br>49. Even if the authors insisted on balanced training and validation split, couldn't these 100 be used for external holdout testing?<br><br>Response: Thank you for your suggestion. The 100 MRIs are all from cases and considering them as a test set does not allow calculating and evaluating all performance metrics. Due to limited number of available controls, we did not have extra control MRIs to combine them with the 100 cases to consider as a holdout test

Powered by Editorial Manager® and ProduXion Manager® from Aries Systems Corporation

set.

50. Setting B: there are total 32 protocols; it is unclear how only 10 protocols were sampled. What was the goal of setting B?

Response: Good questions regarding the setting B.
Selection of 10 protocols:
In the setting B, the goal was to include the data with the following conditions: 1) to include as many protocols as possible, 2) to include an equal number of images (as many as possible) from each protocol, and 3) to include an equal number of protocols for both cases and controls. Based on these conditions, we selected a total of 10 protocols (5 for cases and 5 for controls), each with at least 7 MRI scans available in our MRI list. This resulted in having 70 MRIs (10*7) in setting B00. It is noteworthy that, in each data shuffle, the 7 MRIs per protocol are selected randomly among the available data of that protcol. If we chose more than 10 protocols, e.g. 12, then we could take only 5 MRIs per protocol (since not all of the 12 protocols had more than 5 images in the MRI list). In this case, B00 would have 60 MRIs (12*5), however, we prefer to see the effect of data size on the performance of the model beginning from a higher number and then reduce to smaller numbers in B01, B02, etc.
The goal of setting B:
We designed setting B for having the highest diversity of protocols and at the same time trying to remove any other factor that might affect the model's learning. In setting A, the number of MRIs per protocol is not the same and this may have an effect as the model sees different number of images from each protocol. In setting B, it is the other way around.

51. Overall, the experimental design section was hard to follow; perhaps responding to and adding clarification based on my previous comments might improve the readability of this section.

Response: We hope the current version of the paper and the answers to your comments addresses your concerns and has improved the readability of this section.

52. Generally speaking, the equal number of cases and controls situation is merely an idealised scenario and does not reflect practical clinical setting.

Response: We appreciate your close evaluation of our proposed method. It is true that in clinical settings the cases and controls are not equal. However, the positive point about the proposed approach is that it can ignore the scanner effect to a high degree therefore it is possible to collect data (e.g. from different scanners of the hospital) to provide the necessary training data for the model. Moreover, as we are evaluating the approach in this paper, we are staying with the balanced scenario to avoid other sources of complexity in the model and we may test the model for unbalanced scenarios in future work.

Results and Discussion
53. For Figures 3 and 4, please include a slice from the registered MRI that corresponds to the slice presented in the WM intensity cluster - that will make it easier to compare the image used for learning/prediction vs. the image from which the cluster was derived.

Response: Thank you for your insightful suggestion. Following your comment, we added the middle slice of the registered MRI in Figs. 3 and 4. Also, the WM cluster is now showing the middle slice so it matches the registered MRI. The WM cluster shown is now thresholded as it is the input to the CNN model. As explained in the beginning of this letter, we previously missed to include the thresholding step in the paper.

54. It is a bit hard to follow how the authors are concluding that MRIs 4, 5, 6, and 10 have incorrect registration - the cluster images still capture white matter. Perhaps including additional images on what went wrong would help underscore this point?

Response: Your helpful suggestion in the previous comment also helps solving this issue! Since we are displaying the middle slice of the registered MRI, it is possible to

Powered by Editorial Manager® and ProduXion Manager® from Aries Systems Corporation

	compare these slices to the middle slice of the MNI template shown in Fig. 1. If one sees different sections of the brain compared to the template, the registration has been incorrect. For example, in MRIs 4 and 6 in Fig. 3, we are seeing the eyes in the middle slice of the registered MRI while in the MNI template we do not see the eyes in this slice number. This is an apparent sign of erroneous registration. The following text is added to the paper in this regard:<br>“ These registration errors are identified by comparing the slices of the registered MRIs with the corresponding slice from the MNI template slice shown in Fig. 1. In both cases, the middle slice of the MRI is displayed. In an accurate registration, the brain regions in the registered slice align approximately with those in the template.”<br><br>55. It seems a bit remarkable that even in A18 or B04, the average accuracy is ~67% and 65% - since the datasets have been (artificially) balanced between cases and control images, in extreme paucity of data (such as 1 + 1 in A18), one wouldn't expect any meaningful learning to happen - and therefore, the accuracy should fall to chance level: 50%.<br><br>Response: Thank you for your interesting question. We would also expect the chance level to happen for such settings. We think a much higher number of shuffles would result in an average accuracy of ~50%, however, due to the high computational cost we refrained from doing that. Moreover, the more important for us was to find the mere border (data size) after which the model’s performance falls drastically (e.g. below 70%).<br>Regarding the reason for the 67% average accuracy in A18, it is noteworthy that the test set contained 44 MRIs (a rather large number) and we see in Fig. 2(a) how widely the values fluctuated among the 200 runs (20 shuffles*10 runs each). This shows that the average 67% is not a completely reliable number and more shuffles are necessary which is not the focus of the current study. On the other hand, the accumulative score (MLcps%) for A18 is only 34% which shows the low learning of this setting. The same discussion holds true for B04.<br><br>56. In fact, in B04, the accuracy looks to be ~65% which is ~222/(222+122) i.e., the original cases and control proportion, considering majority class. Can the authors comment on this?<br><br>Response: Thank you for noticing this interesting point. However, the number of data used in setting B is different from 222+122, as we are taking MRIs only from certain protocols. Therefore, we believe this is a coincidence rather than a result of the original class proportion.<br><br>57. Page 18, lines 487 - 491, the authors have inadvertently ended up overfitting to the test set - fitting a model and examining the performance on the test, followed by a decision to change the model, refit, and report performance on the same test set. This is problematic; at the bare minimum, the authors should include (in the supplement), the results from the original situation of using 2D images and acknowledge the fact that these results are obtained after examining the prediction performance on the test set and then re-doing the modelling (overfitted to test).<br><br>Response: We understand and appreciate your concern regarding possible refitting of the model on the same test data. The point is that the test set of the two versions of the setting C were not the same data. By making the decision to exclude 2D MRIs, we completely redesigned setting C. Not only the test data is not the same, but also the number of data in the training, validation, and test were ch...
**Additional Information:**	
**Question**	**Response**
Are you submitting this manuscript to a special series or article collection?	No
**Experimental design and statistics**	Yes

Powered by Editorial Manager® and ProduXion Manager® from Aries Systems Corporation

Full details of the experimental design and statistical methods used should be given in the Methods section, as detailed in our Minimum Standards Reporting Checklist. Information essential to interpreting the data presented should be made available in the figure legends.<br><br>Have you included all the information requested in your manuscript?	
**Resources**<br><br>A description of all resources used, including antibodies, cell lines, animals and software tools, with enough information to allow them to be uniquely identified, should be included in the Methods section. Authors are strongly encouraged to cite Research Resource Identifiers (RRIDs) for antibodies, model organisms and tools, where possible.<br><br>Have you included the information requested as detailed in our Minimum Standards Reporting Checklist?	Yes
**Availability of data and materials**<br><br>All datasets and code on which the conclusions of the paper rely must be either included in your submission or deposited in publicly available repositories (where available and ethically appropriate), referencing such data using a unique identifier in the references and in the "Availability of Data and Materials" section of your manuscript.<br><br>Have you have met the above requirement as detailed in our Minimum Standards Reporting Checklist?	Yes

Revised Manuscript
Click here to access/download;Manuscript;HeteroMRI_Manuscript_rev1.pdf

HeteroMRI: Robust white matter abnormality classification across multi-scanner MRI data

Masoud Abedi[1,2,3,†], Navid Shekarchizadeh[2,3,4,†,§], Pierre-Louis Bazin[5], Nico Scherf[4,6], Julia Lier[7,8], Christa-Caroline Bergner[7,8], for the Alzheimer's Disease Neuroimaging Initiative* , Wolfgang Köhler[7,8,‡], and Toralf Kirsten[1,2,3,4,‡]

[1]Faculty Applied Computer and Bio Sciences, Mittweida University of Applied Sciences, Mittweida, Germany
[2]Department for Medical Data Science, Leipzig University Medical Center, Leipzig, Germany
[3]Institute for Medical Informatics, Statistics, and Epidemiology (IMISE), Leipzig University, Leipzig, Germany
[4]Center for Scalable Data Analytics and Artificial Intelligence (ScaDS.AI) Dresden/Leipzig, Leipzig University, Leipzig, Germany
[5]Full brain picture Analytics, Leiden, The Netherlands
[6]Neural Data Science and Statistical Computing, Max Planck Institute for Human Cognitive and Brain Sciences, Leipzig, Germany
[7]Department of Neurology, Leipzig University Medical Center, Leipzig, Germany
[8]Myelin Research Center (MRC) Leipzig, Department of Neurology, Leipzig University Medical Center, Leipzig, Germany
[§]Correspondence address. Navid Shekarchizadeh, ScaDS.AI, Humboldtstraße 25, 04105 Leipzig, Germany. E-mail: navid.shekarchizadeh@uni-leipzig.de
[†]Equal contribution
[‡]Joint senior authorship

Abstract

Background

Magnetic Resonance Imaging (MRI) is commonly used for analyzing white matter abnormalities in the human brain. Integrating machine learning into MRI analysis can enhance diagnostic processes. However, the application of such techniques for white matter analysis in clinical practice is often limited when MRI data is multi-scanner (i.e., heterogeneous), particularly in scenarios with limited data, as seen in rare diseases. Therefore, it is crucial to develop methods that are highly independent of the MRI scanner and acquisition protocol.

Results

This study introduces *HeteroMRI*, a deep-learning method for classifying MRIs based on white

*A part of the data used in preparation of this article were obtained from the Alzheimer's Disease Neuroimaging Initiative (ADNI) database (adni.loni.usc.edu). As such, the investigators within the ADNI contributed to the design and implementation of ADNI and/or provided data but did not participate in analysis or writing of this report. A complete listing of ADNI investigators can be found at: http://adni.loni.usc.edu/wp-content/uploads/how_to_apply/ADNI_Acknowledgement_List.pdf

matter abnormalities. Most importantly, HeteroMRI mitigates the effects of data heterogeneity on classification performance. Herein, HeteroMRI is employed to detect brain MRIs with white matter abnormalities. This method utilizes intensity clustering of the white matter tissue to minimize the effects of the heterogeneity of MRIs. MRI data from nine public datasets with 32 MRI protocols is included. By using 200 MRIs for training the model, the binary classifier achieves an average accuracy of 96%. Furthermore, the method is evaluated in limited data scenarios, simulating conditions of rare diseases. By reducing the data by 64% and 75%, the model's accuracy has a 4% and 15% decrease, respectively.

Conclusions

The presented method opens new avenues for white matter abnormality-related classification of heterogeneous MRI data without additional machine learning methods to minimize MRI heterogeneity. This classification approach demonstrates a high degree of independence from the MRI scanner and protocol, while also proving to be generalizable to unseen MRI protocols.

Keywords— Brain MRI classification, Multi-scanner MRI, Multi-protocol MRI, Intensity clustering, White matter abnormality, Rare disease, Convolutional neural network

1 Introduction

Magnetic Resonance Imaging (MRI) is widely used in clinics and hospitals to diagnose and follow up neurological diseases by generating images of the central nervous system including the brain. MRI provides a clear contrast between the different tissues of the brain, including white matter (WM) and gray matter (GM) [1]. Detection and assessment of WM abnormalities in demyelinating or neurodegenerative diseases are an important application of MRI in daily clinical practice [2]. An excellent contrast for visualizing WM abnormalities is provided by the Fluid-Attenuated Inversion Recovery (FLAIR) imaging technique making the abnormalities stand out from the surrounding normal brain tissue. FLAIR is a T2-weighted imaging technique in which the signal from cerebrospinal fluid (CSF) is suppressed. By reducing the interference of CSF, the detection of WM abnormalities becomes easier, as these abnormalities may appear adjacent to CSF-filled spaces [3]. MRI provides a three-dimensional (3D) view of the brain and other anatomical structures, making it possible to accurately assess and measure the volume of WM lesions. Volume measurements of the lesions help clinicians to track the disease progression and the effectiveness of the treatments. Moreover, the pattern and volume of lesions are used in research studies to investigate the behavior of different diseases which ultimately contribute to image-based diagnosis of demyelinating disorders [4].

In recent years, Artificial Intelligence (AI) has revolutionized the medical imaging domain, bringing substantial benefits to the analysis of such data [5, 6]. Automating certain aspects of the image analysis process reduces the need for repetitive and time-consuming tasks such as lesion segmentation. This allows healthcare specialists to allocate their expertise to more critical aspects of clinical practice and research. Moreover, manual analysis such as segmentation of MRI data, especially when dealing with complex structures like demyelinating lesions, is resource-intensive and prone to examiner-based variability. AI allows

for rapid and consistent analysis across a large amount of images. A large and growing body of literature has investigated the utilization of Machine Learning (ML) [7, 8] and Deep Learning (DL) [9, 10] models in analyzing medical images.

In brain MRI analysis, studies have focused on developing different models using ML techniques, e.g. for disease classification [11, 12, 13], WM lesion segmentation [14, 15], tumor detection and grading [16, 17], stroke lesion detection and segmentation [18, 19], brain age prediction [20, 21], tracking disease progression in neurodegenerative disorders using longitudinal MRI data [22, 23], and automated segmentation of brain tissues, such as GM, WM, and CSF, for quantitative analysis and volumetric measurements [24, 25]. Before analyzing brain MRIs, certain preprocessing steps are commonly performed to optimize the image data for further analysis and interpretation. AI models and mathematical algorithms are generally used for preprocessing steps including image registration [26], brain extraction or skull stripping [27, 28], image denoising [29, 30, 31], intensity normalization [32, 33], bias field (also called inhomogeneity) correction [34], and MRI interpolation [35].

A challenge in using MRI data in ML/DL models is the variability of MRIs across different sites and scanners. It is shown that scanner differences leads to significant biases in automated MS lesion volumetric analyses, even when the scanner manufacturer and acquisition protocol are consistent [36]. The variabilities due to different scanners and acquisition protocols are often greater than the biological variabilities [37, 38, 39, 40, 41, 42]. High-capacity classifiers, such as deep neural networks, often struggle to produce consistent outcomes when applied to multi-scanner data. This limitation is caused by the model's tendency to overfit to non-bioligical variations, thus the model fails to detect desired biological features or to generalize well across MRI data from unseen scanners [43].

One common strategy to address the above-mentioned challenges is to provide *standardized* images for automated MRI data analysis, especially for WM lesion segmentation models, e.g. as in [44]. By "standardized", we refer to images that have been acquired with the same acquisition protocol and the same MR scanner model. Acquisition protocol refers to a set of procedures and parameters, e.g. Echo Time (TE), Repetition Time (TR), and Inversion Time (TI), used to acquire the images. For example, in the UK Biobank[45], all the MRI data are acquired following highly standardized procedures to be used in future studies. Using a standardized dataset results in consistent image quality, comparable intensity range for each tissue type, and uniform spatial characteristics, although some variability may still exist. This consistency may assist ML models in effectively recognizing and learning disease patterns and features across images, leading to more accurate and reliable results. However, generating a standardized dataset imposes additional costs and requires a well-structured organization at the medical center(s). In addition, even by providing standardized data for training a model, many DL models fail to generalize to images that are acquired with different scanners or protocols than the ones included in the training set [46]. These conditions pose a significant limitation in the applicability of DL models in clinical settings since the images are usually not standardized across centers or even across scanners within one center. Therefore, analysis methods that are highly independent of the MRI scanner and acquisition protocol are preferred.

Another strategy for handling the MRI data heterogeneity is to develop techniques for removing scanner and protocol effects from the available MRIs. Such methods are referred to as *harmonization*. Literature concerning harmonization techniques is relatively limited. Harmonization involves considerable complexities and challenges due to the limited understanding of scanner effects, and the absence of standard criteria for assessing scanner effects and evaluating the harmonization process [47]. The proposed MRI harmonization methods in the literature are generally divided into statistical approaches or ML/DL techniques. One group of statistical methods adjusts the intensity distribution, by normalizing the intensity [32, 48, 49, 50] or by harmonizing values measured from the images (such as cortical thickness) by adjusting the so-called batch effect across different scanners [39, 38, 37, 40, 41, 42]. Nonetheless, these statistical harmonization techniques are effective only in specific applications. For instance, a study has shown that ComBat-based harmonization methods [38, 37] fail to make any noticeable improvement in the performance of DL models in disease classification tasks [43].

The ML/DL harmonization methods aim at learning the scanner and protocol effects and removing them from the multi-center MRIs. Such methods include approaches that are built upon supervised ML [51, 52], supervised DL [47, 53, 54, 55], and unsupervised DL [56, 57] methods. Supervised ML techniques are commonly employed to predict harmonized images by training regression models incorporating manually selected features. On the contrary, DL techniques automatically capture relevant features for the harmonization task. Recently, a novel class of models has been proposed, which integrates both statistical and DL approaches [58]. For an extensive review of MRI harmonization methods, refer to Ref. [59].

There are significant limitations in the application of the ML/DL harmonization methods. As an example, a supervised method [47] is trained using a relatively small dataset of MRIs from 18 individuals, demonstrating its ability to work effectively with limited training data. However, the applicability of the method is limited to datasets that include the MRIs of similar patients from different scanners. Meanwhile, unsupervised methods usually have other limitations. For instance, the method proposed in Ref. [56] harmonizes the images taken by different scanners by removing the non-biological site differences. However, its applicability is restricted to datasets with the acquisition protocols standardized across all scanners. Another unsupervised method [57] necessitates multi-contrast images of the same subject within a single imaging session, with training data acquired from 10 scanners following a standardized protocol on each. In summary, there is no universal or standardized procedure for effectively harmonizing heterogeneous MRI data. The harmonization of MRI data remains a complex and unsolved challenge, given the variations that come with the nature of MRI technology. As mentioned in Ref. [60], no studies have so far evaluated the impact of changes in MR acquisition parameters (such as TE or TR) on the assessment of brain WM lesions, even when using the same MR scanner and sequence. Moreover, even assuming successful harmonization of the MRI data for use in training DL models, these models must also achieve robust generalizability to new MRI data to ensure their applicability in clinical settings.

On a different note, the context of rare diseases has specific challenges and limitations. Data availabil-

ity is extremely limited, which severely restricts the application of ML/DL approaches to these diseases, including both predictive models, such as classifiers, and harmonization methods. For example, in the case of leukodystrophies [61]), the brain MRIs are gathered from various clinical centers over a long period, making it infeasible to even create a standardized dataset. Such datasets are not only small in size but also highly heterogeneous in terms of MRI scanners and protocols. These conditions significantly hinder the applicability of conventional ML methods to rare diseases.

Herein, we present HeteroMRI, an approach for classifying brain MRIs based on WM abnormalities while mitigating the heterogeneity effects of the images acquired from multiple scanners and acquisition protocols. In this paper, HeteroMRI is utilized to detect brains with WM abnormalities in FLAIR images through binary classification. HeteroMRI is applicable to multi-scanner and multi-protocol datasets and demonstrates effectiveness in data-limited conditions, providing a flexible and practical solution for both research and clinical applications. The presented method employs MRI intensity clustering, a technique used in the literature for other MRI-related purposes such as brain tissue segmentation [62, 63], brain tumor segmentation [64] and inhomogeneity correction [65]. HeteroMRI is evaluated in various experimental settings to ensure its robustness. Additionally, we apply the method to limited data scenarios in order to assess the performance and applicability of the presented method for rare diseases. In future work, the method is intended to be applied to the task of classifying two WM diseases based on their distinct WM abnormality patterns. The presented method opens new avenues for performing WM abnormality-related analyses on heterogeneous MRI datasets and the large amount of MRI data generated daily in medical centers.

The current paper is structured in the following way: Sec. 2 provides an overview of the methodology used, detailing the data preprocessing steps and the architecture of the DL model employed in this study. Moving on to Sec. 3, the experiments, the datasets used, the experimental settings, the execution of the model, and the evaluation metrics are presented. Following that, Sec. 4 presents the key results, while Sec. 5 discusses the results, highlights the limitations of the method, and introduces the possible future directions. Finally, Sec. 6 provides the conclusion.

2 Methodology

The WM abnormality detection approach presented in this paper uses heterogeneous brain MRI data with various acquisition protocols (multi-protocol) as the input data for a Convolutional Neural Network (CNN). The model is a binary classifier trained to detect patients with WM abnormalities in their brain MRI. The method consists of three main modules explained in the following subsections, namely MRI preprocessing, Intensity clustering, and DL model. An overview of the methodology is illustrated in Fig. 1.

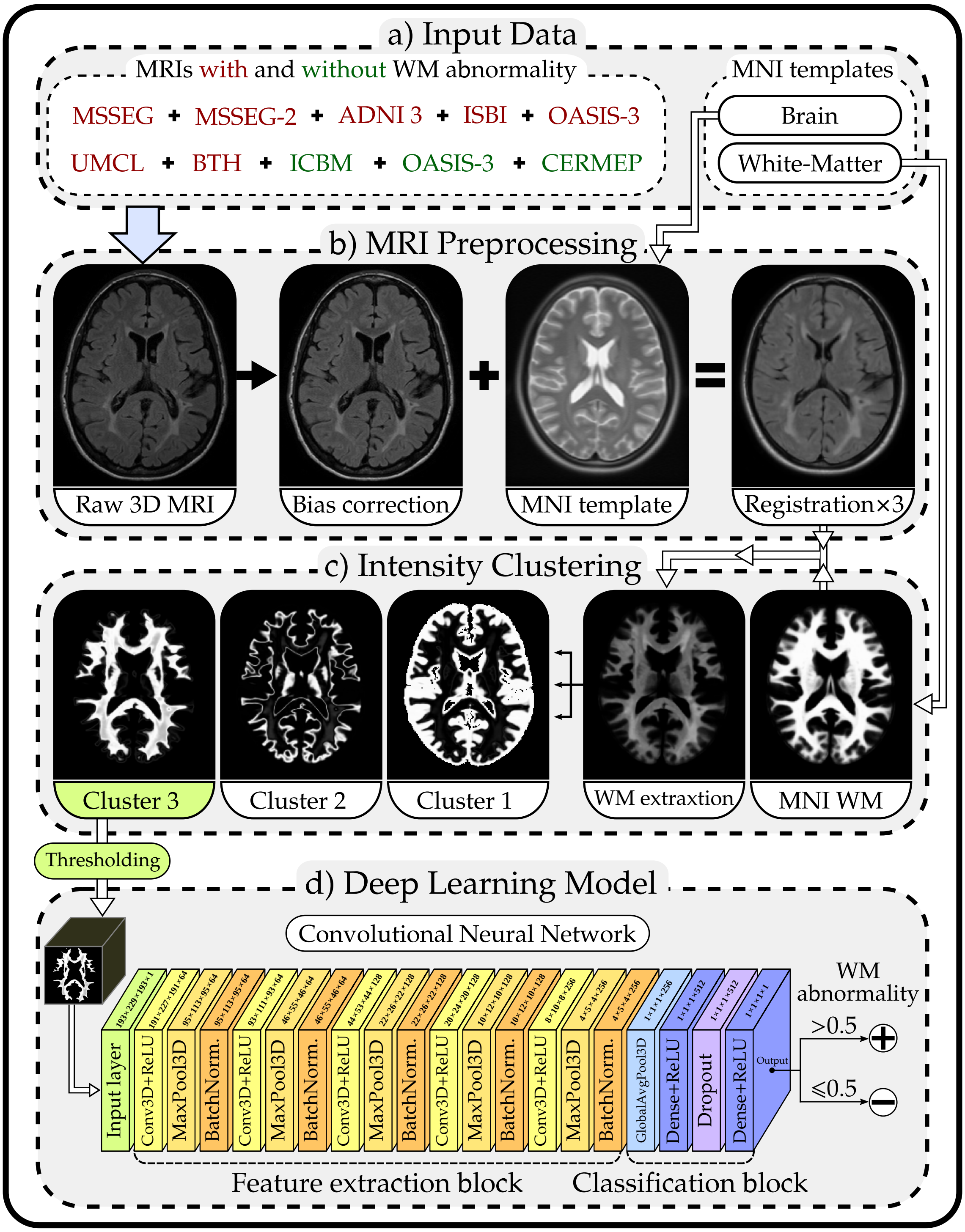

Figure 1: Overview of the methodology: a) Input data: The MRI datasets used for the classification model and MNI brain template [66, 67]. The MRI data with and without WM abnormality are taken from the datasets shown in red and green, respectively. b) MRI preprocessing: The N4 bias field correction method [34] is applied on the FLAIR MRIs (in 3D) and then the MRIs are three times registered (nonlinearly) to the MNI template. c) Intensity clustering: The WM of the brain is extracted and the WM is clustered into three intensity clusters using RFCM [68] algorithm. d) DL model: Only Cluster 3 of the WM is thresholded and used for a binary classification model with the CNN architecture shown.

2.1 MRI preprocessing

For preparing the image data for the analysis, we use our brain MRI preprocessing pipeline, *FlexiMRIprep*[1], that consecutively performs all the requested preprocessing steps/algorithms on all the selected images automatically. Being the optimal MRI sequence in detecting WM abnormalities, only FLAIR images are used in the analysis in this paper. All selected images have a minimum of 128, 192, and 22 voxels in their first, second, and third dimensions, respectively. All the MRIs are converted to NIfTI-1 format using the `dcm2niix` tool (version 1.0.20211006) [69] at this point. The preprocessing steps described below are applied identically to all MRIs from different datasets. Detailed information on the parameters used in each step is reported in the GitHub repository of HeteroMRI[2].

- **Bias field correction**: For correcting the bias field or inhomogeneity issues in the MRIs, we employ the commonly used N4ITK [34] bias field correction method for this purpose. For implementation, the `N4BiasFieldCorrectionImageFilter` class from the `SimpleITK` [70] (version 2.1.1.2) *Python* library with the default parameters is used.
- **Registration**: Registration enables precise spatial mapping and the comparison of anatomical structures among the MRIs. In this process, all the MRIs used for training and testing the AI model are aligned to a standard brain template. Among the available brain templates, we choose the "ICBM 2009c Nonlinear Asymmetric" template [66, 67] (referred to below as the MNI template), which the developers created using the data from the International Consortium for Brain Mapping (ICBM) project [71]. This template was selected due to its high accuracy and the availability of the WM probability map required for our analysis approach. Since there is no dedicated FLAIR template in the MNI template, the T2-weighted template was used due to its proximity to FLAIR. For the registration, the `antsRegistration` tool from the Advanced Normalization Tools (ANTs) [72] (version 2.4.4.post12-g8cc4f8a) is employed. A nonlinear registration is applied three times (with identical parameters) on each image consecutively. This repeated registration aims to achieve a high level of alignment of the MRIs with the template. The selection of three iterations was determined experimentally to maintain high alignment accuracy while avoiding the higher computational cost of additional nonlinear registrations. Multi-pass registration has also been used by others, for example, to address large differences in the initial positions of image pairs [73]. The registered MRIs all have a size of $193 \times 229 \times 193$ voxels with a voxel size of $1 \times 1 \times 1$ mm.

2.2 Intensity clustering

- **WM extraction**: After the brain images are aligned with the MNI template, the WM probability map of the template is used to extract the WM volumes of each brain. Therefore, all the other brain tissues are removed. The WM extraction is performed by using the `MultiplyImages` tool from ANTs.
- **WM clustering**: The WM clustering is performed for two essential purposes: 1) to obtain a subgroup of WM volumes that includes significant signs of WM abnormalities, and 2) to minimize the

[1] https://github.com/ul-mds/FlexiMRIprep
[2] https://github.com/ul-mds/HeteroMRI

negative effect of the heterogeneous MRI data coming from multiple scanners and protocols. These two points are elaborated on in the following.

A clustering algorithm is used to estimate three intensity clusters from the WM volumes obtained in the previous step. The algorithm groups the WM volumes into three subgroups that share a relatively similar intensity range. We expect that one of the clusters shows more indications of WM abnormalities (if present in the brain) since the abnormalities have higher intensity values in FLAIR images. This specific cluster will be used as the training data for the classifier model. The cluster is a membership function with float values in the range of [0,1] for each voxel. As a result, the impact of the heterogeneous nature of the multi-protocol MRIs is minimized. The decision to use three clusters was based on our empirical observations from a dataset different from those used in this study. Through testing various cluster numbers on different MRIs, we found that three clusters yielded consistently comparable patterns in nearly all MRIs. In other words, the shapes of the clusters in one MRI were generally consistent with those in another MRI. This was also later observed in the data of the current study. Therefore, the choice of three clusters is robust and does not depend on a specific dataset.

Here, we employ a Robust Fuzzy C-Means (RFCM) algorithm [68] for WM intensity clustering. The RFCM algorithm modifies the standard FCM objective function by incorporating a local spatial penalty term, leading to the computation of smoother membership functions. This modification not only improves segmentation performance but also provides a level of noise insensitivity. The RFCM algorithm is implemented using the `fuzzy_cmeans` function available in the *Nighres* (Neuroimaging at high resolution) *Python* package, version 1.4.0 [74].

Upon examining the three WM intensity clusters in MRIs with WM abnormalities, we noted that one of the clusters within each MRI, Cluster 3 in Fig. 1(c), consistently exhibited significant lesion-related features. Therefore, from each MRI, we should take the cluster that looks visually similar to Cluster 3 in Fig. 1(c) but it is not always the cluster number 3. For this purpose, we use the Dice similarity coefficient[75] to compare the three clusters of each MRI with a fixed reference cluster to detect the most similar one. The reference image (available in the GitHub repository of HeteroMRI) is generated by averaging the intended intensity cluster of four MRIs from a clinical dataset. This method detected the right cluster for all the MRIs of this study correctly (i.e. with 100% accuracy) as checked manually.

- **Thresholding**: A thresholding is applied on the selected WM intensity cluster of each MRI. All the intensity values below 0.5 are ignored in order to remove uncertain, low-confidence assignments and retain only the core voxels that are strongly associated with the cluster. The value 0.5 is chosen experimentally in the design phase of HeteroMRI by using (clinical) datasets different from those used in this study for the task of classifying two WM diseases. The value 0.5 resulted in the highest improvement in the classification accuracy compared to other tested thresholds. The histogram for

most MRIs follows the same overall pattern: approximately $50 \pm 5\%$ of intensity values are below 0.2, and around $35 \pm 5\%$ are above 0.8. Supplementary Fig. S4 shows the WM cluster of a sample MRI before and after thresholding along with their normalized histograms (for the 99% upper percentile). Finally, the thresholded clusters from the MRIs (one intensity cluster per MRI) are used as training data for the DL model, as described in the following section.

2.3 Deep learning model

The objective is to train a binary classifier model that detects the brain MRIs that have WM abnormalities. Inspired by [76, 77], we configured a 3D CNN comprising a total of 20 layers, as illustrated in Fig. 1(d). The network has a total of 1,795,905 parameters. The model begins with the input layer, followed by a feature extraction block, and ends with a classification block.

In the feature extraction block, we employ five 3D Convolution (Conv3D) layers with 64, 64, 128, 128, and 256 filters, respectively. Each Conv3D has a 3x3x3 kernel size and employs the Rectified Linear Unit (ReLU) activation function. Subsequently, each Conv3D layer is succeeded by a 3D Max Pooling (MaxPool3D) layer with a stride of (2,2,2) and a pool size of (2,2,2) which downscales the 3D input by half in each dimension. Batch Normalization [78] layers with default parameters follow each MaxPool3D layer.

In the classification block, a 3D Global Average Pooling (GlobalAvgPool3D) layer is followed by a Dense layer with a dimensionality of 512 and with a ReLU activation function. To help prevent overfitting, a Dropout layer with a 30% rate is introduced next. Finally, the output layer performs a binary classification employing a Sigmoid activation function. The binary cross-entropy loss, Adam optimizer [79], and an *Early Stopping* feature (*patience*=40) are employed in the model. In each epoch, the checkpoint feature saves the model if the validation accuracy has improved. In the case of an unchanged validation accuracy, the mode is saved if the validation loss has decreased. The *Python* implementation code of the HeteroMRI method is publicly available[3].

3 Experiments

Different MRI datasets are used along with multiple experimental settings with various conditions to train and evaluate the CNN model for classifying brain MRIs. In the following subsections, the datasets and the experimental settings are elaborated.

3.1 Datasets

In this study, we utilized FLAIR images from multiple brain MRI datasets as introduced below. Incorporating a combination of MRIs with a high diversity of acquisition protocols and scanners ensures a robust evaluation of the presented methodology. All the datasets used in this study are either publicly available

[3] https://github.com/ul-mds/HeteroMRI

or are accessible upon request to the respective dataset providers. As presented in Table 1, a total of nine MRI datasets are utilized.

Table 1: List of MRI datasets used in this study

**Dataset name/alias**	**Images**[1]		**Protocols**[2]	**Availability**	**Reference**
	+	−			
ISBI	19	0	1	Public	[80]
UMCL	30	0	1	Public	[81]
MSSEG	52	0	4	AoR[3]	[82]
MSSEG-2	40	0	10	AoR[3]	[83]
BTH	9	0	2	Public	[84]
ICBM	0	5	1	AoR[3]	[85]
OASIS-3	14	90	4	AoR[3]	[86]
ADNI 3	58	0	8	AoR[3]	[87]
CERMEP	0	27	1	AoR[3]	[88]
Sum	222	122	32	-	-

[1] Number of FLAIR images, with (+) and without (−) WM abnormality
[2] Number of MRI protocols in the used data
[3] Accessible on Request (to the respective dataset provider)

The details of each dataset are outlined below:

- **ISBI**: The International Symposium on Biomedical Imaging (ISBI) in 2015 [80] conducted an MS lesion segmentation challenge using longitudinal MRI data. The dataset comprises imaging data from MS patients, acquired using the same scanner and protocol. We utilize 19 FLAIR images from this dataset. For each patient, the MRI taken at the latest time point is used.
- **UMCL**: A cohort of MS patients was imaged at the University Medical Center Ljubljana (UMCL) [81]. The images were acquired using the same scanner and protocol. We use 30 3D FLAIR images from this dataset.
- **MSSEG**: The MSSEG dataset [82] was presented for the MS lesion segmentation challenge during the MICCAI 2016 conference. The dataset contains MRIs of MS patients from four different sites. Each site used different MRI scanners and protocols. We utilize 52 FLAIR images from this dataset.
- **MSSEG-2**[4]: MSSEG-2 [83] is a challenge for the segmentation of new MS lesions in the brain conducted in the MICCAI 2021 conference. At the time of the current research, only the training data of the dataset is accessible. The images of the training set were acquired at 12 different sites and using 10 different scanners. All the images were acquired at two different time points from each patient. From this dataset, we utilize 40 3D FLAIR images from the second time point.
- **BTH**: The brain MRI dataset of MS patients from Baghdad Teaching Hospital (BTH) [84] includes MRIs taken at 20 centers with different protocols. We used nine[5] FLAIR images from this dataset,

[4]Data were generated by participating neurologists in the framework of Observatoire Français de la Sclérose en Plaques (OFSEP), the French MS registry [89]. They collect clinical data prospectively in the European Database for MS (EDMUS) software [90]. MRI of patients were provided as part of a care protocol. Nominative data are deleted from MRI before transfer and storage on the Shanoir platform (Sharing NeurOImagingResources, shanoir.org).

[5]The NIfTI files in this dataset lack orientation information (qform and sform), making it impossible for the registration algorithm to identify the correct orientation of the brain. Additionally, the MRIs are 2D, resulting in around 10 times fewer slices than the pixels in the first and second dimensions, and they all share the same pixel thickness across all three dimensions. Consequently, the brain appears unrealistically short in 3D view. Due to these dataset-specific conditions, we applied two additional preprocessing steps at the beginning for this dataset: 1) added correct orientation information to each file, and 2) edited slice thicknesses in the header of NIfTI files based on slice thickness information provided in the dataset's metadata. However, the height of many images still does not appear realistic and may cause problems for the registration. Therefore only nine images were used

which were taken using two different protocols.

- **ICBM**[6]: The International Consortium for Brain Mapping (ICBM) [85] has developed a probabilistic atlas and reference system for the human brain for normal adults. The dataset includes 20 3D FLAIR images; however, only five were selected for this study, as our neurology specialist confirmed these to be the only ones free of WM abnormalities. The images were acquired using the same scanner and protocol.
- **OASIS-3**: The Open Access Series of Imaging Studies (OASIS) is a project with the goal of providing open access to neuroimaging datasets of the brain. Among the OASIS datasets, FLAIR images are exclusively available in OASIS-3 [86]. OASIS-3 encompasses both cognitively normal adults and individuals at different stages of cognitive decline. Our model utilizes a total of 104 FLAIR and T2-FLAIR images from this dataset, acquired using four different protocols. Out of these images, 90 were meticulously selected by two neurology specialists from a pool of 600, confirming the absence of WM abnormalities in the brain. The other 14 images included different patterns of WM abnormalities.
- **ADNI 3**: The Alzheimer's Disease Neuroimaging Initiative (ADNI) database[7] provides an extensive collection of neuroimaging and clinical data [87]. In our model, we included 58 3D FLAIR images from the ADNI 3 dataset, all of which contained WM abnormalities. These images were carefully selected to encompass various patterns of lesions, including multifocal and confluent lesions, as well as those located in the brain stem, as confirmed by a neurology specialist.
- **CERMEP**: The CERMEP-IDB-MRXFDG dataset [88] comprises MRI, CT, and [^{18}F]FDG PET image data with BIDS standard of healthy subjects. The dataset has 37 FLAIR images obtained using the same scanner and protocol. As reported in the original study, these images underwent visual review by two neurologists to confirm the absence of any apparent brain abnormalities. However, due to our strict criteria for even minor lesions, our neurologists confirmed only 27 MRIs as free of WM abnormalities for use as control data in our model.

From the MRI datasets explained above, a total of 344 images are used in this study for training and testing the model. Around 35% of these MRIs are 2D, based on our definition that MRIs with 70 or fewer slices are considered 2D. A comprehensive list of the MRI files is available in the GitHub repository of HeteroMRI providing details for each image, including the subject ID from the original dataset and the acquisition protocol.

3.2 Experimental settings

Various experimental settings have been designed for a robust evaluation of the presented classification approach. An experimental setting means the specification of the data used for training, validating, and testing the CNN model. By employing the datasets explained in Sec. 3.1, FLAIR images from different scanners and acquisition protocols are intentionally combined and used for training and testing the model.

[6]The ICBM project (Principal Investigator John Mazziotta, M.D., University of California, Los Angeles) is supported by the National Institute of Biomedical Imaging and BioEngineering. ICBM is the result of efforts of co-investigators from UCLA, Montreal Neurologic Institute, University of Texas at San Antonio, and the Institute of Medicine, Juelich/Heinrich Heine University - Germany.

[7]The ADNI was launched in 2003 as a public-private partnership, led by Principal Investigator Michael W. Weiner, MD. The primary goal of ADNI (https://adni.loni.usc.edu) has been to test whether serial MRI, positron emission tomography (PET), other biological markers, and clinical and neuropsychological assessment can be combined to measure the progression of Mild Cognitive Impairment (MCI) and early Alzheimer's Disease (AD).

The images necessary for each setting are selected randomly from the MRIs available. The number of MRIs with and without WM abnormality is balanced in the training, validation, and test data of all the settings. There are four setting groups, namely A, B, C, and D. In setting A, the data are selected based on datasets while in the settings B, C, and D, the data are incorporated based on their acquisition protocol. We assigned a protocol name to each of the MRIs based on the scanner name and model, magnetic field strength, and acquisition parameters. The protocol naming convention is explained in the HeteroMRI Github repository. The experimental settings are introduced below:

- **Setting *A***: In setting A, the goal is to evaluate HeteroMRI on a combination of MRIs from different datasets beginning from a relatively large number of data and then decreasing the data gradually. In setting A, there are 19 settings that are run independently. In $A00$, 244 MRIs from nine datasets are used. The data of *each* dataset is split into training (70%), validation (10%), and test (20%) sets. In $A01$, the training data is downsized by approximately 10% while the test set remains the same images as in $A00$. The downsizing process continues up to $A18$, where the training and validation sets together include only four MRIs. The downsizing is performed by removing random MRIs while keeping the maximum possible number of protocols among the data. Across all settings from $A00$ to $A18$, the test set remains identical. Supplementary Table S1 shows the number of MRIs used for training, validation, and test sets from each dataset in the settings $A00$ to $A18$.
- **Setting *B***: In setting B, the goal is to choose the MRIs with the most diversity of protocols while having an equal number of MRIs from each protocol therefore the model sees the same number of MRIs per protocol. MRIs from 10 different protocols are incorporated. The test data is selected from all protocols. In $B00$, from each protocol, five MRIs for training, one MRI for validation, and one MRI for the test are used. In the next subsequent settings, the training data is reduced. By $B04$ only one MRI per protocol is used for the training set. In Supplementary Table S2, the list of selected protocols and the number of MRIs used for training, validation, and test sets for $B00$ to $B04$ is reported.
- **Setting *C***: In setting C, the goal is to assess the generalizability of HeteroMRI to unseen MRI protocols. In this setting, MRIs from eight different protocols are included. Only data from 3D MRIs are included in this setting due to the reason later explained in Sec. 4.3. MRIs from six protocols are used *only* in training and validation sets while the other two protocols are *only* used in the test set. In fact, the trained model does not see any data from the protocols used in the test set during training. We consider 10 cases, in each case considering two different protocols for testing the model. The setting C begins with $C00$ which uses 64 MRIs for training and validation. This continues up to $C06$ with only eight MRIs for training and validation. In Table S3, the list of selected protocols and the number of MRIs used for training, validation, and test sets for $C00$ to $C06$ is reported.
- **Setting *D***: In setting D, the goal is to see the effect of the number of MRI protocols on the performance of the model. Beginning from $D00$ and going toward $D03$, more protocols are included in the data used for training and testing the model. At the same time, the total number of data and test set size are kept the same among the settings $D00$ to $D03$ (82 MRIs for training including validation data), therefore it is possible to compare the results of the settings to see the effect of having more protocols in the data. In $D00$, there are MRIs from four protocols. In $D01$ to $D03$,

there are MRIs from, respectively, six, eight, and 10 protocols. The test data is selected from all protocols. In Table S4, the list of selected protocols and the number of MRIs used for training, validation, and test sets for $D00$ to $D03$ is reported.

3.3 Model execution

All the MRIs used in this study, introduced in Sec. 3.1, are preprocessed following the procedure elaborated in Sec. 2.1. Next, the intensity clustering procedure is applied to each preprocessed MRI, following the procedure introduced in Sec. 2.2. As a result, a single intensity cluster per MRI is used for training or testing the model. Notably, the 3D intensity clusters obtained from the MRIs serve as the exclusive training data for the model. The model has no exposure to the original MRIs or any form of WM lesion annotation file. For the preprocessing and intensity clustering tasks, we used a machine with Intel(R) Xeon(R) Gold 6240R CPU @ 2.40GHz and 128 GB of RAM. The preprocessing pipeline (introduced in Sec. 2.1) employs a parallelization approach in some of the preprocessing steps to make the procedure faster. The computation time required for preprocessing each MRI depends on multiple factors; nevertheless, the total number of voxels in the 3D MRI plays a more significant role. More specifically, based on our assessments, the number of slices of the MR image highly affects the required computation time. The Supplementary Fig. S1, shows the average time required for preprocessing and intensity clustering of five sample MRI dimensions.

The CNN model explained in Sec.2.3 is trained and tested on each experimental setting independently. In settings A, B, and D, the required number of data is selected (and split into training, validation, and test sets) from all available data 20 independent times (referred to below as "data shuffle"). For each data shuffle, the model is trained and tested 10 times. In setting C, 5 data shuffles are selected, and for each shuffle, the model is trained and tested 10 times. For training and testing the CNN model, we used a computational server with AMD Epyc 7352 CPU, 1 TB of CPU RAM, and NVIDIA A100 GPU (40 GB GPU RAM). The average required time for training the model of each setting is shown in Supplementary Fig. S2. The inference time of the model on a single test data is a few seconds. The inference process can be efficiently performed without the necessity of a GPU.

3.4 Evaluation metrics

To assess the performance of the classification model, we employ five common metrics: accuracy, sensitivity, specificity, F1 score, and precision. Moreover, the area under the receiver operating characteristic curve (AUROC) is reported for selected settings. Additionally, we calculate a cumulative metric called Machine Learning cumulative performance score (*MLcps*) [91]. The *MLcps* combines the pre-computed performance metrics into a single metric that encapsulates the core aspects of all the metrics. The value of *MLcps* is equal to the area of the polygon created by the metrics in a radar plot. We used the *MLcps Python* package version 0.0.6. The *MLcps* metric was originally designed for comparing and identifying the best-performing ML algorithm. However, herein, we utilize *MLcps* to compare the performance of the same model for the different amounts of training data. As we have a fixed number of pre-calculated metrics

(accuracy, sensitivity, specificity, F1 score, and precision), we define $MLcps\%$ as

$$MLcps\% = \frac{MLcps}{MLcps_{max}} \times 100, \tag{1}$$

where $MLcps$ is the area of the pentagon in the radar plot and $MLcps_{max}$ is the area of the pentagon when all the five metrics are 100%.

4 Results

In this section, we present and analyze the results of the experiments to evaluate the performance of the HeteroMRI method. The results are reported separately for the experimental setting A, B, C, and D. Furthermore, the insights on limited data scenarios gained through the experiments are discussed afterward. The classification metrics are provided in box plot and radar plot formats in Figs. 2, 5, 6, and 7. In all box plots, the triangle marker indicates the mean value, and the whiskers represent $1.5 \times IQR$, where IQR is the interquartile range. In settings A, B, and D, the box plots show the distribution of 200 values for each setting # corresponding to 20 data shuffles that each has been run 10 times. By "setting #", we mean for example $A00, A01, \ldots, A18$. The higher the setting #, the less training data is used. In setting C, the box plots show the distribution of 10 values for each setting #. Each of these 10 values is the average of 50 values (five shuffles, 10 runs each) corresponding to the 10 cases with different protocols chosen as the test set, as explained in Sec. 3.2.

The radar plots illustrate the metric values for all the setting #s simultaneously, allowing us to perceive the effect of reducing training data size on each metric. In addition, the radar plots are utilized for calculating the $MLcps$ values using Eq. (1). The $MLcps\%$ values for the settings A, B, and C are reported in the Figs. 2, 5, and 6 in part (e). Evaluation of the presented approach across various experimental settings provides several key insights, offering a detailed understanding of its performance and challenges. The results of each experimental setting are presented below.

4.1 Setting A

As shown in Fig. 2, setting $A00$, in which the highest number of MRIs (174 training+26 validation+44 test) were included, demonstrates an average accuracy of 96% in the classification of MRIs. The training and testing data in this setting includes, in total, 32 different MRI protocols. This underscores the model's adaptability and robustness in handling a diverse range of imaging protocols. Notably, the effect of reducing the training data on the model's performance is inspected here. In $A07$, where the training data (including validation data) is reduced to 36% of $A00$, the accuracy remains above 92%. Beginning from setting $A08$, where the training data is 25% of $A00$, the average accuracy and sensitivity have a sharp decrease. With much further decreasing the training data (e.g. $A18$ with only two MRIs as training) the accuracy and sensitivity gradually decrease to low values as expected while specificity tends to remain relatively high. In relatively limited data settings (particularly from $A11$ to $A18$), the boxplots indicate that accuracy values have significant fluctuations across the range. This shows that, with additional repetitions

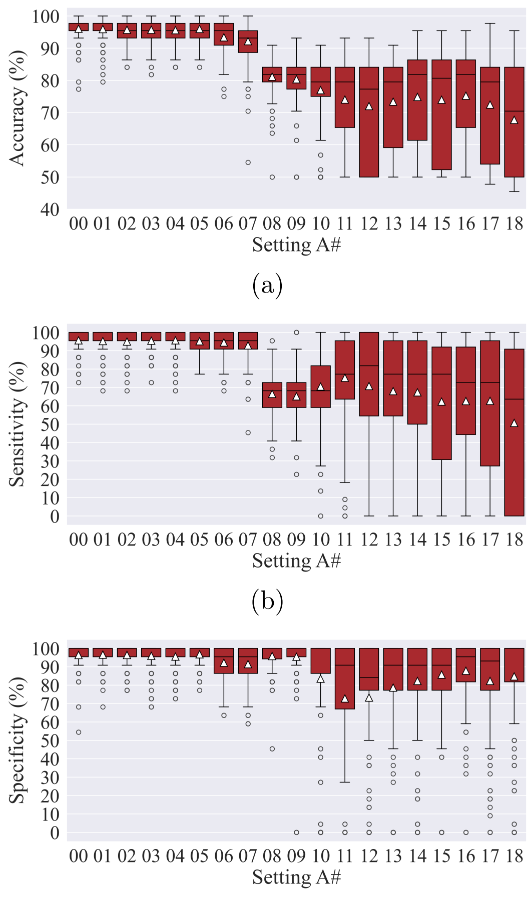

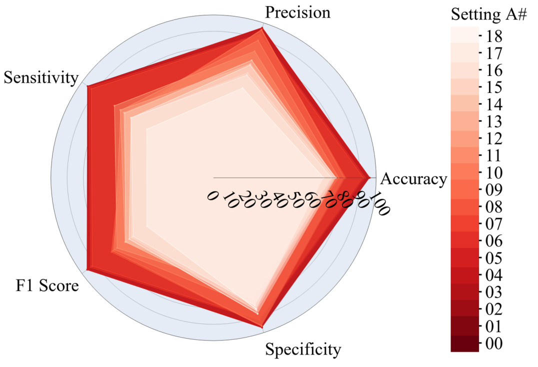

Setting	$A00$	$A01$	$A02$	$A03$	$A04$
MLcps%	91	91	91	90	90
**Setting**	$A05$	$A06$	$A07$	$A08$	$A09$
MLcps%	91	85	83	64	63
**Setting**	$A10$	$A11$	$A12$	$A13$	$A14$
MLcps%	56	51	45	47	49
**Setting**	$A15$	$A16$	$A17$	$A18$	
MLcps%	45	48	43	34	

(e)

Figure 2: Classification results of settings $A00$ to $A18$: (a) accuracy, (b) sensitivity, (c) specificity, (d) radar plot of five classification metrics for different setting #s, and (e) *MLcps%* (a cumulative performance score) in % for each setting #. The triangle marker indicates the mean value and the whiskers represent $1.5 \times IQR$. In each setting # (i.e. $A00$, $A01$, ..., $A18$) the training set size is sequentially reduced by approximately 10% relative to the previous setting, as detailed in Supplementary Table S 1. Average accuracy starts at 96% for $A00$, where training includes 200 MRIs, and remains above 92% until $A07$, with 72 training data, after which it drops with further reductions in training data. A similar trend is observed in the *MLcps%*.

of such settings using different data shuffles, the average accuracy would likely converge toward the chance level (50%) due to insufficient training data for the CNN to effectively learn. However, investigating this phenomenon is beyond the scope of this study.

We conducted a permutation test to assess the statistical significance of the model's performance. By randomly shuffling the class labels 7,000 times and retraining the model (for setting $A00$) on each shuffled dataset, a distribution of accuracy values under chance conditions was obtained. All of the 7,000 permuted accuracy values were smaller than the model's original accuracy for setting $A00$. This results in a p-value of 0.0001, showing a statistically significant and meaningful distinction between the two classes, confirming that the model captures real patterns in the data. Supplementary Fig. S3 shows the normalized histogram of the permutations compared to the model's original accuracy.

Sample raw MRIs, the registered MRIs, the obtained WM intensity clusters, and the model's label predictions are provided here for a better insight into the data used for testing the model. Figs. 3 and 4 depict

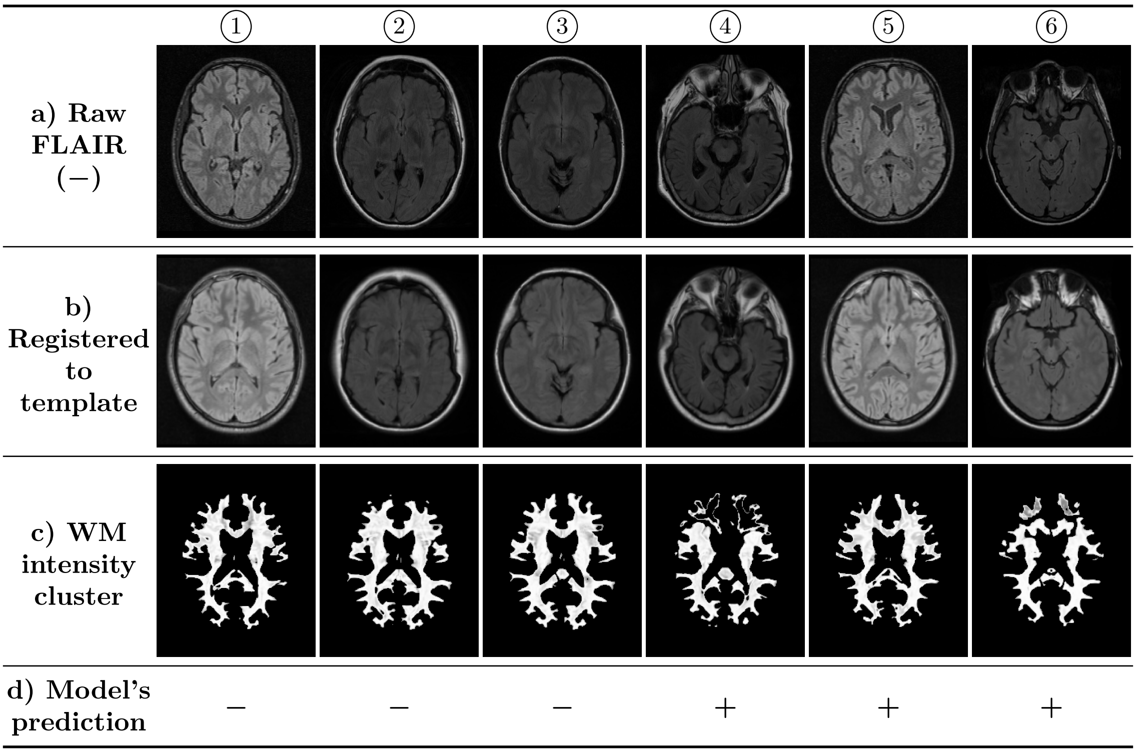

Figure 3: Example of six MRIs (① to ⑥) *without* WM abnormality. a) Raw FLAIR images, b) the MRI three times registered to the MNI template (the middle slice shown), c) the obtained intensity clusters (thresholded) used for testing the model, and d) the prediction of the model in setting $A00$ for the presence of WM abnormalities (+: with WM abnormality, −: without WM abnormality). This figure shows three cases of true negative and three cases of false positive. The WM intensity cluster slice corresponds to the registered MRI slice but not exactly to the raw MRI slice due to deformations from non-linear registrations. MRIs source: ①, ⑤:[88], ②, ③, ④:[86], ⑥:[85].

samples of MRIs without and with WM abnormality, respectively. Below each raw MRI, the three-times registered MRI, and the obtained WM intensity cluster (thresholded) are illustrated. Additionally, the model's prediction for the presence of WM abnormalities in setting $A00$ is reported. Each figure includes three true predictions and three false predictions. It is important to note that the shown WM intensity cluster and the registered MRI slice show the same location of the brain (the middle slice). However, these two slices do not directly correspond to the raw MRI slice. This is because the non-linear registration process results in the deformation of the brain. As a result, finding the exact corresponding slices in the raw MRI and the registered one is impractical. Here, the middle slice of the registered MRI is illustrated. The raw MRI slices shown here are selected based on their visual similarity to the corresponding registered slices. The slices of the thresholded WM cluster are presented here solely to illustrate the input provided to the DL model for label prediction.

By investigating the possible reasons for the false predictions in setting $A00$, certain aspects became apparent. In some MRIs, the registration process has not been successful in correctly aligning the brain to the brain template. In such cases, the brain regions are not located in the correct locations after the three-times registration in the MRI preprocessing phase. Therefore, in the WM extraction step, wrong parts of the brain are extracted as WM. This mostly results in a false prediction by the model, especially if the MRI is, in fact, free of WM abnormalities. Examples of incorrect registration are MRIs ④, ⑤,

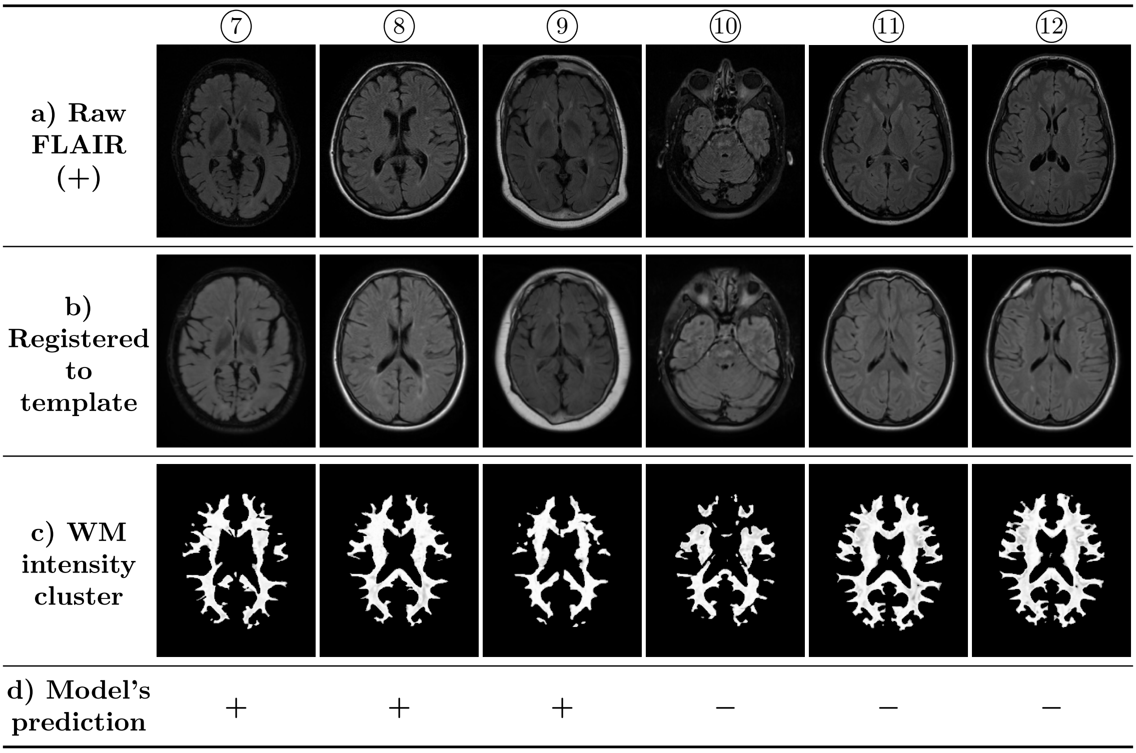

Figure 4: Example of six MRIs (⑦ to ⑫) *with* WM abnormality. a) raw FLAIR images, b) the MRI three times registered to the MNI template (the middle slice shown), c) the obtained intensity clusters (thresholded) used for testing the model, and d) the prediction of the model in setting $A00$ for the presence of WM abnormalities (+: with WM abnormality, −: without WM abnormality). This figure shows three cases of true positive and three cases of false negative. The WM intensity cluster slice corresponds to the registered MRI slice but not exactly to the raw MRI slice due to deformations from non-linear registrations. MRIs source: ⑦,⑩:[87], ⑧:[84], ⑨:[86], ⑪,⑫:[80].

⑥, ⑩, and ⑫ shown in Figs. 3 and 4. These registration errors are identified by comparing the slices of the registered MRIs with the corresponding slice from the MNI template slice shown in Fig. 1(b). In both cases, the middle slice of the MRI is displayed. In an accurate registration, the brain regions in the registered slice align approximately with those in the template. We noticed that some of the MRIs with incorrect registration are 2D MRIs (such as MRIs ④ and ⑫). The low number of slices in 2D MRIs appears to be a contributing factor to registration problems in some cases. Considering that MRI registration is usually a challenging problem, other factors are also likely to contribute to incorrect registrations; however, they have not been investigated in this study. We refrained from excluding the MRIs with erroneous registration from the study, as our goal was to evaluate HeteroMRI as a fully automatic method without user interference.

4.2 Setting B

The setting B which uses an equal number of MRIs from each MRI protocol is designed to make the prediction task more challenging for the model. Setting $B00$ has an average accuracy of 88.6%, as shown in Fig. 5. In terms of the amount of training data, setting $A07$ is the closest match to $B00$. While $B00$ includes 50 training and 10 validation data, $A07$ has a comparable setup with 52 training and 20 validation data. However, setting B is a more challenging scenario than setting A because the model sees an equal number of MRIs from each MRI protocol without being biased by higher number of images of some protocols. Despite this challenge, the accuracy of $B00$ is only 3.6% less than that of $A07$. This shows the high independence of the presented MRI classification approach on the acquisition protocol of the FLAIR images. With further decrease in the training data, in settings $B01$ to $B04$, all metrics show a gradual decrease in value. We expect the accuracy to likely tend toward the chance level by repeating more data shuffles in very limited data scenarios such as $B03$ and $B04$.

4.3 Setting C

The evaluation of setting C is of more importance since it is very close to the real-world use of such a model as it evaluates the generalizability of HeteroMRI to MRIs from unseen protocols. In this setting, the MRI protocols of the test set are not present in the training data. It resembles a situation in which a clinical center has heterogeneous MRI data and wants to train a classification model with them. Then the model is supposed to classify new MRIs brought by new patients from other centers, acquired most probably with MRI protocols different from those in the training data.

It is noteworthy that in setting C only 3D MRIs (with 192 or more slices) are included in training and test data, as discussed in the next paragraph. Setting $C00$, as reported in Fig. 6, shows an average accuracy of 94.9% with 64 MRIs used for training (including validation), which proves the generalizability of the trained model to unseen MRI scanners and protocols. By reducing the data to 46, in $C02$, the model shows an accuracy of 88.1%. By further decreasing the data to 36, the model's performance drops to 64.4% accuracy in $C03$, which is expected to tend toward 50% with more data shuffles.

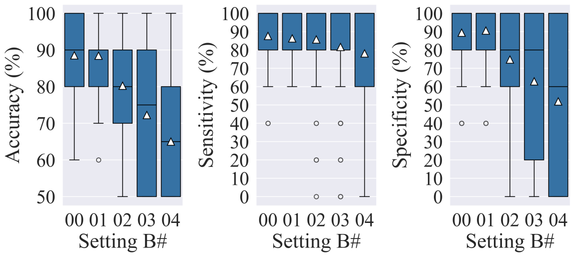

(a) (b) (c)

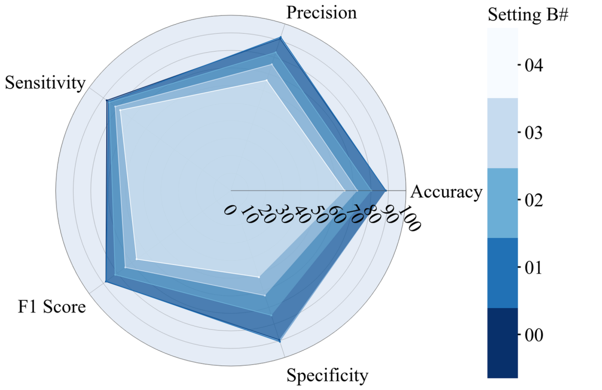

(d)

**Setting**	$B00$	$B01$	$B02$	$B03$	$B04$
MLcps%	76	76	61	48	37

(e)

Figure 5: Classification results of settings $B00$ to $B04$: (a) accuracy, (b) sensitivity, (c) specificity, (d) radar plot of five classification metrics for different setting #s, and (e) $MLcps\%$ (a cumulative performance score) in % for each setting #. The triangle marker indicates the mean value and the whiskers represent $1.5 \times IQR$. In each setting # (i.e. $B00$, $B01$, …, $B04$) the training set size is sequentially reduced relative to the previous setting, as detailed in Supplementary Table S 2. In setting B, an equal number from 10 different MRI protocols is used for the model. Average accuracy starts at 88.5% for $B00$, where training includes 60 MRIs, and remains above 80% until $B02$, with 40 training data, after which it decreases gradually with further reductions in training data. A similar trend is observed in the $MLcps\%$.

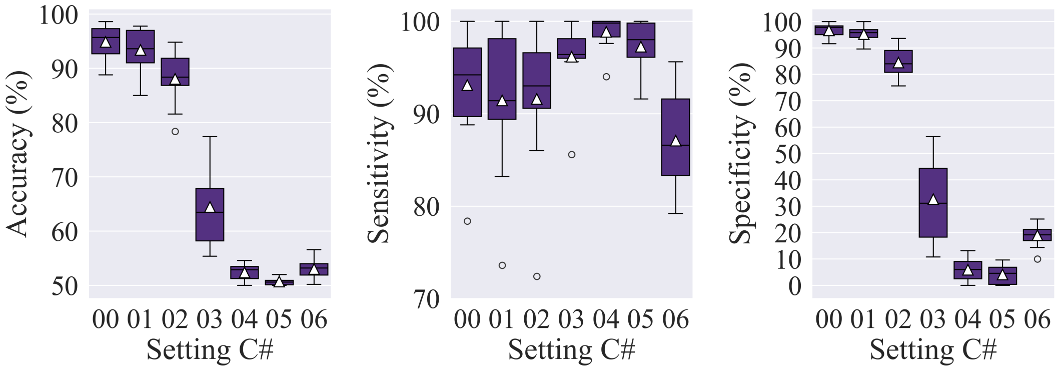

(a) Accuracy (b) Sensitivity (c) Specificity

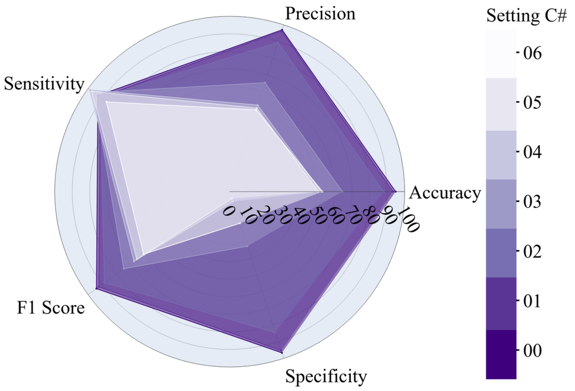

(d) Radar plot

**Setting**	$C00$	$C01$	$C02$	$C03$	$C04$
MLcps%	89	86	75	38	25
**Setting**	$C05$	$C06$			
MLcps%	24	23			

(e) *MLcps%* values (in %)

Figure 6: Classification results of settings $C00$ to $C06$: (a) accuracy, (b) sensitivity, (c) specificity, (d) radar plot of five classification metrics for different setting #s, and (e) *MLcps%* (a cumulative performance score) in % for each setting #. The triangle marker indicates the mean value and the whiskers represent $1.5 \times IQR$. In each setting # (i.e. $C00$, $C01$, ..., $C06$) the training set size is sequentially reduced relative to the previous setting, as detailed in Supplementary Table S 3. In setting C, the MRI protocols of the test set are unseen by the model during training. Average accuracy starts at 94.9% for $C00$, where training includes 64 MRIs, and remains above 88% until $C02$, with 46 training data, after which it drops sharply with further reductions in training data. A similar trend is observed in the *MLcps%*.

Initially, we used both 3D and 2D MRIs for setting C. However, the model showed a lack of robustness when its generalizability to different protocols was evaluated (i.e. by varying the protocols considered as test data). As we suspected the 2D MRIs (with 70 or fewer slices) as a source of the model's poor performance, we redesigned the setting C to include *only* 3D MRIs. As a result, the performance significantly improved, as reported in the results for setting C. It is important to emphasize that the test data were not fixed for the two versions of setting C, as the model was tested with multiple data shuffles for each setting, with the data being split again in each shuffle. This ensures that this was not a case of refitting on the same test data. Additionally, to verify the adverse effect of 2D MRIs, we designed a separate setting with *only* 2D MRIs. In this case, the model showed very poor robustness.

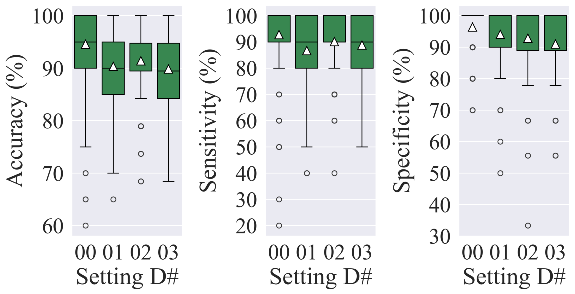

(a) Accuracy (b) Sensitivity (c) Specificity

Figure 7: Classification results of settings $D00$ to $D03$: (a) accuracy, (b) sensitivity, and (c) specificity. The triangle marker indicates the mean value and the whiskers represent $1.5 \times IQR$. In each setting # (i.e. $D00$, $D01$, ..., $D03$) the number of MRI protocols is sequentially increased relative to the previous setting while maintaining equal training set size (82 MRIs), as detailed in Supplementary Table S 4. Average accuracy starts at 94.6% for $D00$, where training data includes four different MRI protocols, and ends at 89.8% for $D03$, with 10 MRI protocols in training data.

4.4 Setting *D*

Setting D, in which the number of protocols was increased in each setting # while maintaining the same data size (82 MRIs for training), shows the slight negative effect of having higher numbers of protocols, as observed by the overall decrease in accuracy, sensitivity, and specificity (Fig.7). In the setting with the most diverse protocols, setting $D03$, the model classifies the MRIs with an accuracy of 89.80%.

4.5 Limited data scenarios

A more detailed examination of the impact of reducing training data is presented in Table 2. By comparing settings A, B, and C a rough correlation can be concluded between the number of MRIs in the training data and the performance of the model for the classification task of this study, regardless of the experimental setting. When the training data (including validation) consists of at least 72 MRIs (as in $A07$), an accuracy of 92% is expected. By having 40 to 46 MRIs in the training set (as in $A09, B02$, and $C02$), the accuracy falls within the range of 80% to 88%. Further reducing the training data to the 20 to 36 range (as in $A12, B03$, and $C03$) is associated with an accuracy of 72% or less and an F1 score of 74% or less. In this last scenario, the result is not fully reliable as it may tend toward chance level by increasing the number of data shuffles.

5 Discussion

In this study, we introduce HeteroMRI, a novel approach for robust classification of brain MRIs based on WM abnormalities, specifically for cases where the data consists of heterogeneous MRIs acquired with different scanners and acquisition protocols. In this paper, we apply this approach to train a binary classification model on FLAIR images to detect MRIs containing WM abnormalities. The results demonstrate that the proposed method shows a robust performance by achieving up to 96% accuracy in detecting MRIs with WM abnormalities across various experimental settings. The method effectively handles the high

Table 2: Average performance results of selected experimental settings (in %). The table shows the effect of reducing training data on the model's performance in settings A, B, and C. For these settings, three cases are reported respectively: 1) with the highest number of training data, 2) with the borderline number of training data after which the performance drops, and 3) with the number of training data that results in relatively low performance. Setting D shows the effect of increasing the number of MRI protocols for the same number of MRI data. AUROC: the area under the receiver operating characteristic curve.

**Setting**	**Data size**[1]		**Protocols**[2]		**Accuracy**	**Sensitivity**	**Specificity**	**F1 score**	**AUROC**
	**Train**†	**Test**	**Train**†	**Test**					
$A00$	200	44	31	14	96.03	95.68	96.39	95.85	97.69
$A07$	72	44	17±1	14	92.17	92.91	91.43	92.23	96.57
$A09$	42	44	9±1	14	80.33	65.18	95.48	76.40	92.31
$A12$	20	44	6±1	14	72.02	70.82	73.23	67.18	94.03
$B00$	60	10	10	10	88.55	87.60	89.50	87.94	95.64
$B02$	40	10	10	10	80.25	85.70	74.80	81.63	92.37
$B03$	30	10	10	10	72.25	81.60	62.90	74.48	90.14
$C00$	64	10	8	2	94.88	93.08	96.68	94.12	99.50
$C02$	46	10	8	2	88.06	91.60	84.52	88.44	98.10
$C03$	36	10	8	2	64.44	96.16	32.72	74.80	93.18
$D00$	82	20	4	4	94.58	92.75	96.40	93.98	99.29
$D03$	82	20	10	10	89.84	88.80	91.00	90.06	94.72

[1] Number of MRIs used in the training and test sets
[2] Number of MRI protocols present in the training and test data
† Including the validation data

diversity of MRI scanners and acquisition protocols present in the data. In addition, HeteroMRI mitigates the adverse effect of data heterogeneity through MRI preprocessing steps, primarily the WM intensity clustering step, without relying on additional ML methods.

Notably, the approach performs reliably even in scenarios with limited data, showing the potential of HeteroMRI for application in the classification of MRIs in WM-related rare diseases. For the binary classification task of detecting MRIs with WM abnormalities, providing 72 and 42 MRIs results in a 92% and 80% accuracy, respectively. Moreover, HeteroMRI shows generalizability to unseen MRI scanners and protocols which makes the method stand out compared to many other ML/DL methods that fail in this aspect. Furthermore, by not relying on manual lesion annotations, HeteroMRI reduces the demand for expert human resources, facilitating its usability in practical settings compared to other methods.

Despite the promising results of the presented method, its limitations should be considered. HeteroMRI faces a challenge with the registration problem with certain MRIs leads to a false prediction. For addressing this challenge, a more elaborate registration strategy may reduce the misalignments which is a future work direction for further improving the method. Notably, 2D MRIs were identified as one of the factors that can contribute to registration issues, strongly suggesting the use of 3D MRIs with HeteroMRI. Further factors that cause an incorrect registration were not investigated in this study. Another limitation of HeteroMRI is the high GPU memory requirement which is not easily available in every computing server. For each

specific use case, one can evaluate the method's performance by downscaling the dimension of input images to decrease the required GPU memory. In this study, HeteroMRI was not directly compared with other methods due to the reason that we do not know of any method that performs binary classification for detecting MRIs with WM abnormality, even with standardized data. On the other hand, comparing our method with widely available lesion segmentation methods is not practical as they usually require lesion annotation masks. In addition, a binary classification of MRIs based on the lesion segmentation result is controversial as a threshold is necessary as a minimum value for considering the segmented lesion volume as a WM abnormality in the brain. Furthermore, HeteroMRI was evaluated with only FLAIR images being the superior MRI image for analyzing WM abnormalities. However, the method can be evaluated with other MRI sequences, such as T1 and T2, for instance, to study brain conditions related to other brain tissues. Additionally, the design of HeteroMRI can be upgraded to a multi-channel format, enabling the integration of multiple MRI sequences per subject for enhanced analysis.

6 Conclusion

In this study, we introduced HeteroMRI, a novel approach for robust classification of brain MRIs based on white matter abnormalities, specifically designed to handle heterogeneous MRI data acquired from diverse scanners and acquisition protocols. The method is also adaptable to standardized MRI datasets acquired using a uniform scanner and protocol. HeteroMRI achieved high accuracy in detecting MRIs with WM abnormalities, even in scenarios with limited data. Furthermore, the method proved to be generalizable to unseen MRI protocols, highlighting its robustness. There is room for enhancing HeteroMRI's performance by improving the registration accuracy. Our future research will focus on applying this approach to differentiate between unspecific and disease-associated WM lesions, as well as to classify rare demyelinating diseases against their differential diagnoses.

7 Availability of Supporting Source Code and Requirements

Project name: HeteroMRI v1.0

Project home page: https://github.com/ul-mds/HeteroMRI

Operating system(s): Linux-based OS (Ubuntu recommended)

Programming language: Python

Other requirements: TensorFlow v2.x, NVIDIA GPU with CUDA support

License: GNU GPL version 3

8 Data Availability

All the datasets used in this study are either publicly available or are accessible upon request to the respective dataset providers as referenced in Table 1.

9 Abbreviations

AI: Artificial Intelligence; ANTs: Advanced Normalization Tools; AUROC: Area Under the Receiver Operating Characteristic Curve; BTH: Baghdad Teaching Hospital; CSF: CerebroSpinal Fluid; CT: Computed Tomography; CNN: Convolutional Neural Network; DL: Deep Learning; TE: Echo Time; FLAIR: Fluid-Attenuated Inversion Recovery; FCM: Fuzzy C-Means; GM: Gray Matter; ICBM: International Consortium for Brain Mapping; TI: Inversion Time; ML: Machine Learning; $MLcps$: Machine Learning Cumulative Performance Score; MRI: Magnetic Resonance Imaging; MNI: Montreal Neurological Institute; MS: Multiple Sclerosis; NifTI: Neuroimaging Informatics Technology Initiative; ReLU: Rectified Linear Unit; TR: Repetition Time; RFCM: Robust Fuzzy C-Means; 3D: three-dimensional; WM: White Matter.

10 Competing Interests

The authors declare that they have no competing interests

11 Authors' Contributions

M.A. (Methodology, Formal Analysis, Software), N.SH. (Conceptualization, Methodology, Writing – Original Draft), P.L.B. (Methodology), N.SC. (Supervision), J.L. (Supervision, Data Curation), C.C.B. (Supervision), W.K. (Supervision, Data Curation, Project Investigation, Funding Acquisition), T.K. (Supervision, Project Investigation, Funding Acquisition). All authors contributed to reviewing and editing the manuscript.

12 Funding

The authors acknowledge the financial support by the Federal Ministry of Health of Germany in project LeukoExpert (grant no. ZMVI1-2520DAT94), the State Ministry for Education and Research of Germany in the project Tag-White (grant no. 100602109), and the Federal Ministry of Education and Research of Germany and by Sächsische Staatsministerium für Wissenschaft, Kultur und Tourismus in the programme Center of Excellence for AI-research "Center for Scalable Data Analytics and Artificial Intelligence Dresden/Leipzig", project identification number: ScaDS.AI. Supported by the Open Access Publication Fund of Leipzig University.

13 Acknowledgments

The authors sincerely thank Dr. Sina Sadeghi for his insightful comments on the manuscript. The authors are grateful to the Center for Information Services and High-Performance Computing [Zentrum für Informationsdienste und Hochleistungsrechnen (ZIH)] at TU Dresden for providing its facilities for high throughput calculations. Data were provided in part by OASIS Longitudinal Multimodal Neuroimaging: Principal Investigators: T. Benzinger, D. Marcus, J. Morris; NIH P30 AG066444, P50 AG00561, P30

NS09857781, P01 AG026276, P01 AG003991, R01 AG043434, UL1 TR000448, R01 EB009352. AV-45 doses were provided by Avid Radiopharmaceuticals, a wholly owned subsidiary of Eli Lilly. Part of the Data collection and sharing for the Alzheimer's Disease Neuroimaging Initiative (ADNI) is funded by the National Institute on Aging (National Institutes of Health Grant U19 AG024904). The grantee organization is the Northern California Institute for Research and Education. In the past, ADNI has also received funding from the National Institute of Biomedical Imaging and Bioengineering, the Canadian Institutes of Health Research, and private sector contributions through the Foundation for the National Institutes of Health (FNIH) including generous contributions from the following: AbbVie, Alzheimer's Association; Alzheimer's Drug Discovery Foundation; Araclon Biotech; BioClinica, Inc.; Biogen; Bristol-Myers Squibb Company; CereSpir, Inc.; Cogstate; Eisai Inc.; Elan Pharmaceuticals, Inc.; Eli Lilly and Company; EuroImmun; F. Hoffmann-La Roche Ltd and its affiliated company Genentech, Inc.; Fujirebio; GE Healthcare; IXICO Ltd.; Janssen Alzheimer Immunotherapy Research & Development, LLC.; Johnson & Johnson Pharmaceutical Research &Development LLC.; Lumosity; Lundbeck; Merck & Co., Inc.; Meso Scale Diagnostics, LLC.; NeuroRx Research; Neurotrack Technologies; Novartis Pharmaceuticals Corporation; Pfizer Inc.; Piramal Imaging; Servier; Takeda Pharmaceutical Company; and Transition Therapeutics. Part of the data collection and sharing for this project was provided by the International Consortium for Brain Mapping (ICBM; Principal Investigator: John Mazziotta, MD, PhD). ICBM funding was provided by the National Institute of Biomedical Imaging and BioEngineering. ICBM data are disseminated by the Laboratory of Neuro Imaging at the University of Southern California. Part of the data was provided in collaboration with The Observatoire Français de la Sclérose en Plaques (OFSEP), which is supported by a grant provided by the French State and handled by the "Agence Nationale de la Recherche," within the framework of the "Investments for the Future" program, under the reference ANR-10-COHO-002, by the Eugène Devic EDMUS Foundation against multiple sclerosis and by the ARSEP Foundation.

14 Additional Files

Supplementary Table S1. The experimental setting A and details of the MRI data used.

Supplementary Table S2. The experimental setting B and details of the MRI data used.

Supplementary Table S3. The experimental setting C and details of the MRI data used.

Supplementary Table S4. The experimental setting D and details of the MRI data used.

Supplementary Fig. S1. Average required time for preprocessing and intensity clustering of five sample MRI dimensions.

Supplementary Fig. S2. Average required time for training the CNN model of each experimental setting.

Supplementary Fig. S3. Normalized histogram of accuracy values from 7,000 permutations of shuffled labels (orange) and from the 20 data shuffles of the original labels (blue). Dashed lines indicate the mean accuracy for each group, highlighting the significant difference between chance-level accuracy and the model's performance with true labels.

Supplementary Fig. S4. The WM intensity cluster of a sample MRI (a) before and (b) after applying

a threshold value of 0.5. The normalized histograms of their 99% upper percentile are shown in (c) and (d), respectively.

References

[1] T. Yousaf, G. Dervenoulas, & M. Politis (2018) *Chapter two - advances in MRI methodology*, M. Politis (Ed.) *Imaging in Movement Disorders: Imaging Methodology and Applications in Parkinson's Disease*, vol. 141 of *International Review of Neurobiology*, pp. 31–76, Academic Press

[2] F. Agosta, S. Galantucci, & M. Filippi (2017) *Advanced magnetic resonance imaging of neurodegenerative diseases*, Neurological Sciences, **38(1)**:pp. 41–51

[3] M. Filippi, P. Preziosa, B. L. Banwell, F. Barkhof, O. Ciccarelli, N. De Stefano, J. J. G. Geurts, F. Paul, D. S. Reich, A. T. Toosy, A. Traboulsee, M. P. Wattjes, T. A. Yousry, A. Gass, C. Lubetzki, B. G. Weinshenker, & M. A. Rocca (2019) *Assessment of lesions on magnetic resonance imaging in multiple sclerosis: practical guidelines*, Brain, **142(7)**:pp. 1858–1875

[4] C. Domínguez-Fernández, J. Egiguren-Ortiz, J. Razquin, M. Gómez-Galán, L. De las Heras-García, E. Paredes-Rodríguez, E. Astigarraga, C. Miguélez, & G. Barreda-Gómez (2023) *Review of technological challenges in personalised medicine and early diagnosis of neurodegenerative disorders*, International Journal of Molecular Sciences, **24(4)**

[5] H.-P. Chan, R. K. Samala, L. M. Hadjiiski, & C. Zhou (2020) *Deep learning in medical image analysis*, G. Lee & H. Fujita (Eds.) *Deep Learning in Medical Image Analysis : Challenges and Applications*, pp. 3–21, Springer International Publishing, Cham

[6] A. S. Panayides, A. Amini, N. D. Filipovic, A. Sharma, S. A. Tsaftaris, A. Young, D. Foran, N. Do, S. Golemati, T. Kurc, K. Huang, K. S. Nikita, B. P. Veasey, M. Zervakis, J. H. Saltz, & C. S. Pattichis (2020) *AI in medical imaging informatics: Current challenges and future directions*, IEEE Journal of Biomedical and Health Informatics, **24(7)**:pp. 1837–1857

[7] B. J. Erickson, P. Korfiatis, Z. Akkus, & T. L. Kline (2017) *Machine learning for medical imaging*, RadioGraphics, **37(2)**:pp. 505–515, pMID: 28212054

[8] M. L. Giger (2018) *Machine learning in medical imaging*, Journal of the American College of Radiology, **15(3, Part B)**:pp. 512–520, data Science: Big Data Machine Learning and Artificial Intelligence

[9] S. Suganyadevi, V. Seethalakshmi, & K. Balasamy (2022) *A review on deep learning in medical image analysis*, International Journal of Multimedia Information Retrieval, **11(1)**:pp. 19–38

[10] M. I. Razzak, S. Naz, & A. Zaib (2018) *Deep learning for medical image processing: Overview, challenges and the future*, N. Dey, A. S. Ashour, & S. Borra (Eds.) *Classification in BioApps: Automation of Decision Making*, pp. 323–350, Springer International Publishing, Cham

[11] N. Garg, M. S. Choudhry, & R. M. Bodade (2023) *A review on Alzheimer's disease classification from normal controls and mild cognitive impairment using structural MR images*, Journal of Neuroscience Methods, **384**:p. 109745

[12] J. Wen, E. Thibeau-Sutre, M. Diaz-Melo, J. Samper-González, A. Routier, S. Bottani, D. Dormont, S. Durrleman, N. Burgos, & O. Colliot (2020) *Convolutional neural networks for classification of Alzheimer's disease: Overview and reproducible evaluation*, Medical Image Analysis, **63**:p. 101694

[13] A. Kursad Poyraz, S. Dogan, E. Akbal, & T. Tuncer (2022) *Automated brain disease classification using exemplar deep features*, Biomedical Signal Processing and Control, **73**:p. 103448

[14] D. García-Lorenzo, S. Francis, S. Narayanan, D. L. Arnold, & D. Louis Collins (2013) *Review of automatic segmentation methods of multiple sclerosis white matter lesions on conventional magnetic resonance imaging*, Medical Image Analysis, **17(1)**:pp. 1–18

[15] O. Cetin, V. Seymen, & U. Sakoglu (2020) *Multiple sclerosis lesion detection in multimodal MRI using simple clustering-based segmentation and classification*, Informatics in Medicine Unlocked, **20**:p. 100409

[16] J. Amin, M. Sharif, M. Yasmin, & S. L. Fernandes (2020) *A distinctive approach in brain tumor detection and classification using MRI*, Pattern Recognition Letters, **139**:pp. 118–127

[17] M. A. Naser & M. J. Deen (2020) *Brain tumor segmentation and grading of lower-grade glioma using deep learning in MRI images*, Computers in Biology and Medicine, **121**:p. 103758

[18] S. Zhang, S. Xu, L. Tan, H. Wang, & J. Meng (2021) *Stroke lesion detection and analysis in MRI images based on deep learning*, Journal of Healthcare Engineering, **2021**:p. 5524769

[19] Y. Kabir, M. Dojat, B. Scherrer, F. Forbes, & C. Garbay (2007) *Multimodal MRI segmentation of ischemic stroke lesions*, *2007 29th Annual International Conference of the IEEE Engineering in Medicine and Biology Society*, pp. 1595–1598

[20] H. Peng, W. Gong, C. F. Beckmann, A. Vedaldi, & S. M. Smith (2021) *Accurate brain age prediction with lightweight deep neural networks*, Medical Image Analysis, **68**:p. 101871

[21] H. Sajedi & N. Pardakhti (2019) *Age prediction based on brain MRI image: A survey*, Journal of Medical Systems, **43(8)**:p. 279

[22] W. M. van Oostveen & E. C. M. de Lange (2021) *Imaging techniques in Alzheimer's disease: A review of applications in early diagnosis and longitudinal monitoring*, International Journal of Molecular Sciences, **22(4)**

[23] R. Zivadinov, J. Sepcic, D. Nasuelli, R. D. Masi, L. M. Bragadin, M. A. Tommasi, S. Zambito-Marsala, R. Moretti, A. Bratina, M. Ukmar, R. S. Pozzi-Mucelli, A. Grop, G. Cazzato, & M. Zorzon (2001) *A longitudinal study of brain atrophy and cognitive disturbances in the early phase of relapsing-remitting multiple sclerosis*, Journal of Neurology, Neurosurgery & Psychiatry, **70(6)**:pp. 773–780

[24] J. West, J. B. M. Warntjes, & P. Lundberg (2012) *Novel whole brain segmentation and volume estimation using quantitative MRI*, European Radiology, **22(5)**:pp. 998–1007

[25] S. Valverde, A. Oliver, E. Roura, S. González-Villà, D. Pareto, J. C. Vilanova, L. Ramió-Torrentà, Àlex Rovira, & X. Lladó (2017) *Automated tissue segmentation of MR brain images in the presence of white matter lesions*, Medical Image Analysis, **35**:pp. 446–457

[26] N. Andrade, F. A. Faria, & F. A. M. Cappabianco (2018) *A practical review on medical image registration: From rigid to deep learning based approaches*, *2018 31st SIBGRAPI Conference on Graphics, Patterns and Images (SIBGRAPI)*, pp. 463–470

[27] J. Kleesiek, G. Urban, A. Hubert, D. Schwarz, K. Maier-Hein, M. Bendszus, & A. Biller (2016) *Deep MRI brain extraction: A 3D convolutional neural network for skull stripping*, NeuroImage, **129**:pp. 460–469

[28] P. Kalavathi & V. B. S. Prasath (2016) *Methods on skull stripping of MRI head scan images—a review*, Journal of Digital Imaging, **29(3)**:pp. 365–379

[29] A. Kaur & G. Dong (2023) *A complete review on image denoising techniques for medical images*, Neural Processing Letters, **55(6)**:pp. 7807–7850

[30] J. Mohan, V. Krishnaveni, & Y. Guo (2014) *A survey on the magnetic resonance image denoising methods*, Biomedical Signal Processing and Control, **9**:pp. 56–69

[31] J. V. Manjón, J. Carbonell-Caballero, J. J. Lull, G. García-Martí, L. Martí-Bonmatí, & M. Robles (2008) *MRI denoising using non-local means*, Medical Image Analysis, **12(4)**:pp. 514–523

[32] M. Shah, Y. Xiao, N. Subbanna, S. Francis, D. L. Arnold, D. L. Collins, & T. Arbel (2011) *Evaluating intensity normalization on MRIs of human brain with multiple sclerosis*, Medical Image Analysis, **15(2)**:pp. 267–282

[33] C. Loizou, M. Pantziaris, I. Seimenis, & C. Pattichis (2009) *Brain MR image normalization in texture analysis of multiple sclerosis*, *2009 9th International Conference on Information Technology and Applications in Biomedicine*, pp. 1–5

[34] N. J. Tustison, B. B. Avants, P. A. Cook, Y. Zheng, A. Egan, P. A. Yushkevich, & J. C. Gee (2010) *N4ITK: Improved N3 bias correction*, IEEE Transactions on Medical Imaging, **29(6)**:pp. 1310–1320

[35] M. Joliot & B. Mazoyer (1993) *Three-dimensional segmentation and interpolation of magnetic resonance brain images*, IEEE Transactions on Medical Imaging, **12(2)**:pp. 269–277

[36] R. Shinohara, J. Oh, G. Nair, P. Calabresi, C. Davatzikos, J. Doshi, R. Henry, G. Kim, K. Linn, N. Papinutto, D. Pelletier, D. Pham, D. Reich, W. Rooney, S. Roy, W. Stern, S. Tummala, F. Yousuf, A. Zhu, N. Sicotte, R. Bakshi, & the NAIMS Cooperative (2017) *Volumetric analysis from a harmonized multisite brain MRI study of a single subject with multiple sclerosis*, American Journal of Neuroradiology, **38(8)**:pp. 1501–1509

[37] J.-P. Fortin, N. Cullen, Y. I. Sheline, W. D. Taylor, I. Aselcioglu, P. A. Cook, P. Adams, C. Cooper, M. Fava, P. J. McGrath, M. McInnis, M. L. Phillips, M. H. Trivedi, M. M. Weissman, & R. T. Shinohara (2018) *Harmonization of cortical thickness measurements across scanners and sites*, NeuroImage, **167**:pp. 104–120

[38] J.-P. Fortin, D. Parker, B. Tunç, T. Watanabe, M. A. Elliott, K. Ruparel, D. R. Roalf, T. D. Satterthwaite, R. C. Gur, R. E. Gur, R. T. Schultz, R. Verma, & R. T. Shinohara (2017) *Harmonization of multi-site diffusion tensor imaging data*, NeuroImage, **161**:pp. 149–170

[39] J.-P. Fortin, E. M. Sweeney, J. Muschelli, C. M. Crainiceanu, & R. T. Shinohara (2016) *Removing inter-subject technical variability in magnetic resonance imaging studies*, NeuroImage, **132**:pp. 198–212

[40] C. Marzi, M. Giannelli, A. Barucci, C. Tessa, M. Mascalchi, & S. Diciotti (2024) *Efficacy of MRI data harmonization in the age of machine learning: a multicenter study across 36 datasets*, Scientific Data, **11(1)**:p. 115

[41] R. Pomponio, G. Erus, M. Habes, J. Doshi, D. Srinivasan, E. Mamourian, V. Bashyam, I. M. Nasrallah, T. D. Satterthwaite, Y. Fan, L. J. Launer, C. L. Masters, P. Maruff, C. Zhuo, H. Völzke, S. C. Johnson, J. Fripp, N. Koutsouleris, D. H. Wolf, R. Gur, R. Gur, J. Morris, M. S. Albert, H. J. Grabe, S. M. Resnick, R. N. Bryan, D. A. Wolk, R. T. Shinohara, H. Shou, & C. Davatzikos (2020) *Harmonization of large MRI datasets for the analysis of brain imaging patterns throughout the lifespan*, NeuroImage, **208**:p. 116450

[42] J. Radua, E. Vieta, R. Shinohara, P. Kochunov, Y. Quidé, M. J. Green, C. S. Weickert, T. Weickert, J. Bruggemann, T. Kircher, I. Nenadić, M. J. Cairns, M. Seal, U. Schall, F. Henskens, J. M. Fullerton, B. Mowry, C. Pantelis, R. Lenroot, V. Cropley, C. Loughland, R. Scott, D. Wolf, T. D. Satterthwaite, Y. Tan, K. Sim, F. Piras, G. Spalletta, N. Banaj, E. Pomarol-Clotet, A. Solanes, A. Albajes-Eizagirre, E. J. Canales-Rodríguez, S. Sarro, A. Di Giorgio, A. Bertolino, M. Stäblein, V. Oertel, C. Knöchel, S. Borgwardt, S. du Plessis, J.-Y. Yun, J. S. Kwon, U. Dannlowski, T. Hahn, D. Grotegerd, C. Alloza, C. Arango, J. Janssen, C. Díaz-Caneja, W. Jiang, V. Calhoun, S. Ehrlich, K. Yang, N. G. Cascella, Y. Takayanagi, A. Sawa, A. Tomyshev, I. Lebedeva, V. Kaleda, M. Kirschner, C. Hoschl, D. Tomecek, A. Skoch, T. van Amelsvoort, G. Bakker, A. James, A. Preda, A. Weideman, D. J. Stein, F. Howells, A. Uhlmann, H. Temmingh, C. López-Jaramillo, A. Díaz-Zuluaga, L. Fortea, E. Martinez-Heras, E. Solana, S. Llufriu, N. Jahanshad, P. Thompson, J. Turner, T. van Erp, D. Glahn, G. Pearlson, E. Hong, A. Krug, V. Carr, P. Tooney, G. Cooper, P. Rasser, P. Michie, S. Catts, R. Gur, R. Gur, F. Yang, F. Fan, J. Chen, H. Guo, S. Tan, Z. Wang, H. Xiang, F. Piras, F. Assogna, R. Salvador, P. McKenna, A. Bonvino, M. King, S. Kaiser, D. Nguyen, & J. Pineda-Zapata (2020) *Increased power by harmonizing structural MRI site differences with the ComBat batch adjustment method in ENIGMA*, NeuroImage, **218**:p. 116956

[43] R. Kushol, P. Parnianpour, A. H. Wilman, S. Kalra, & Y.-H. Yang (2023) *Effects of MRI scanner manufacturers in classification tasks with deep learning models*, Scientific Reports, **13(1)**:p. 16791

[44] A. P. De Rosa, M. Benedetto, S. Tagliaferri, F. Bardozzo, A. D'Ambrosio, A. Bisecco, A. Gallo, M. Cirillo, R. Tagliaferri, & F. Esposito (2024) *Consensus of algorithms for lesion segmentation in brain MRI studies of multiple sclerosis*, Scientific Reports, **14(1)**:p. 21348

[45] T. J. Littlejohns, J. Holliday, L. M. Gibson, S. Garratt, N. Oesingmann, F. Alfaro-Almagro, J. D. Bell, C. Boultwood, R. Collins, M. C. Conroy, N. Crabtree, N. Doherty, A. F. Frangi, N. C. Harvey, P. Leeson, K. L. Miller, S. Neubauer, S. E. Petersen, J. Sellors, S. Sheard, S. M. Smith, C. L. M. Sudlow, P. M. Matthews, & N. E. Allen (2020) *The UK Biobank imaging enhancement of 100,000 participants: rationale, data collection, management and future directions*, Nature Communications, **11(1)**:p. 2624

[46] G. Mårtensson, D. Ferreira, T. Granberg, L. Cavallin, K. Oppedal, A. Padovani, I. Rektorova, L. Bonanni, M. Pardini, M. G. Kramberger, J.-P. Taylor, J. Hort, J. Snædal, J. Kulisevsky, F. Blanc, A. Antonini, P. Mecocci, B. Vellas, M. Tsolaki, I. Kłoszewska, H. Soininen, S. Lovestone, A. Simmons, D. Aarsland, & E. Westman (2020) *The reliability of a deep learning model in clinical out-of-distribution MRI data: A multicohort study*, Med Image Anal, **66**:p. 101714

[47] M. E. Torbati, D. S. Minhas, C. M. Laymon, P. Maillard, J. D. Wilson, C.-L. Chen, C. M. Crainiceanu, C. S. DeCarli, S. J. Hwang, & D. L. Tudorascu (2023) *MISPEL: A supervised deep learning harmonization method for multi-scanner neuroimaging data*, Medical Image Analysis, **89**:p. 102926

[48] L. G. Nyúl & J. K. Udupa (1999) *On standardizing the MR image intensity scale*, Magnetic Resonance in Medicine, **42(6)**:pp. 1072–1081

[49] R. T. Shinohara, E. M. Sweeney, J. Goldsmith, N. Shiee, F. J. Mateen, P. A. Calabresi, S. Jarso, D. L. Pham, D. S. Reich, & C. M. Crainiceanu (2014) *Statistical normalization techniques for magnetic resonance imaging*, NeuroImage: Clinical, **6**:pp. 9–19

[50] J. Wrobel, M. Martin, R. Bakshi, P. Calabresi, M. Elliot, D. Roalf, R. Gur, R. Gur, R. Henry, G. Nair, J. Oh, N. Papinutto, D. Pelletier, D. Reich, W. Rooney, T. Satterthwaite, W. Stern, K. Prabhakaran, N. Sicotte, R. Shinohara, & J. Goldsmith (2020) *Intensity warping for multisite MRI harmonization*, NeuroImage, **223**:p. 117242

[51] R. Garcia-Dias, C. Scarpazza, L. Baecker, S. Vieira, W. H. Pinaya, A. Corvin, A. Redolfi, B. Nelson, B. Crespo-Facorro, C. McDonald, D. Tordesillas-Gutiérrez, D. Cannon, D. Mothersill, D. Hernaus, D. Morris, E. Setien-Suero, G. Donohoe, G. Frisoni, G. Tronchin, J. Sato, M. Marcelis, M. Kempton, N. E. van Haren, O. Gruber, P. McGorry, P. Amminger, P. McGuire, Q. Gong, R. S. Kahn, R. Ayesa-Arriola, T. van Amelsvoort, V. Ortiz-García de la Foz, V. Calhoun, W. Cahn, & A. Mechelli (2020) *Neuroharmony: A new tool for harmonizing volumetric MRI data from unseen scanners*, NeuroImage, **220**:p. 117127

[52] A. Jog, A. Carass, S. Roy, D. L. Pham, & J. L. Prince (2017) *Random forest regression for magnetic resonance image synthesis*, Medical Image Analysis, **35**:pp. 475–488

[53] B. E. Dewey, C. Zhao, J. C. Reinhold, A. Carass, K. C. Fitzgerald, E. S. Sotirchos, S. Saidha, J. Oh, D. L. Pham, P. A. Calabresi, P. C. van Zijl, & J. L. Prince (2019) *Deepharmony: A deep learning approach to contrast harmonization across scanner changes*, Magnetic Resonance Imaging, **64**:pp. 160–170, artificial Intelligence in MRI

[54] B. E. Dewey, L. Zuo, A. Carass, Y. He, Y. Liu, E. M. Mowry, S. Newsome, J. Oh, P. A. Calabresi, & J. L. Prince (2020) *A disentangled latent space for cross-site MRI harmonization*, A. L. Martel, P. Abolmaesumi, D. Stoyanov, D. Mateus, M. A. Zuluaga, S. K. Zhou, D. Racoceanu, & L. Joskowicz (Eds.) *Medical Image Computing and Computer Assisted Intervention – MICCAI 2020*, pp. 720–729, Springer International Publishing, Cham

[55] N. K. Dinsdale, M. Jenkinson, & A. I. Namburete (2021) *Deep learning-based unlearning of dataset bias for MRI harmonisation and confound removal*, NeuroImage, **228**:p. 117689

[56] S. Liu & P.-T. Yap (2024) *Learning multi-site harmonization of magnetic resonance images without traveling human phantoms*, Communications Engineering, **3(1)**:p. 6

[57] L. Zuo, B. E. Dewey, Y. Liu, Y. He, S. D. Newsome, E. M. Mowry, S. M. Resnick, J. L. Prince, & A. Carass (2021) *Unsupervised MR harmonization by learning disentangled representations using information bottleneck theory*, NeuroImage, **243**:p. 118569

[58] F. Hu, A. Lucas, A. A. Chen, K. Coleman, H. Horng, R. W. Ng, N. J. Tustison, K. A. Davis, H. Shou, M. Li, R. T. Shinohara, & T. A. D. N. Initiative (2023) *Deepcombat: A statistically motivated, hyperparameter-robust, deep learning approach to harmonization of neuroimaging data*, bioRxiv

[59] F. Hu, A. A. Chen, H. Horng, V. Bashyam, C. Davatzikos, A. Alexander-Bloch, M. Li, H. Shou, T. D. Satterthwaite, M. Yu, & R. T. Shinohara (2023) *Image harmonization: A review of statistical and deep learning methods for removing batch effects and evaluation metrics for effective harmonization*, NeuroImage, **274**:p. 120125

[60] N. De Stefano, M. Battaglini, D. Pareto, R. Cortese, J. Zhang, N. Oesingmann, F. Prados, M. A. Rocca, P. Valsasina, H. Vrenken, C. A. Gandini Wheeler-Kingshott, M. Filippi, F. Barkhof, & Àlex Rovira (2022) *MAGNIMS recommendations for harmonization of MRI data in MS multicenter studies*, NeuroImage: Clinical, **34**:p. 102972

[61] L. L. Resende, A. R. B. de Paiva, F. Kok, C. da Costa Leite, & L. T. Lucato (2019) *Adult leukodystrophies: a step-by-step diagnostic approach*, Radiographics, **39(1)**:pp. 153–168

[62] X. Tu, J. Gao, C. Zhu, J.-Z. Cheng, Z. Ma, X. Dai, & M. Xie (2016) *MR image segmentation and bias field estimation based on coherent local intensity clustering with total variation regularization*, Medical & Biological Engineering & Computing, **54(12)**:pp. 1807–1818

[63] D. Kumar, R. K. Agrawal, & P. Kumar (2022) *Bias-corrected intuitionistic fuzzy c-means with spatial neighborhood information approach for human brain MRI image segmentation*, IEEE Transactions on Fuzzy Systems, **30(3)**:pp. 687–700

[64] A. Khosravanian, M. Rahmanimanesh, P. Keshavarzi, & S. Mozaffari (2021) *Fast level set method for glioma brain tumor segmentation based on superpixel fuzzy clustering and lattice boltzmann method*, Computer Methods and Programs in Biomedicine, **198**:p. 105809

[65] L. Szilágyi, S. M. Szilágyi, B. Benyó, & Z. Benyó (2011) *Intensity inhomogeneity compensation and segmentation of MR brain images using hybrid c-means clustering models*, Biomedical Signal Processing and Control, **6(1)**:pp. 3–12, biomedical signal processing(Extended selected papers from the 7th IFAC Symposium on Modelling and Control in Biomedical Systems(MCBMS'09))

[66] V. Fonov, A. Evans, R. McKinstry, C. Almli, & D. Collins (2009) *Unbiased nonlinear average age-appropriate brain templates from birth to adulthood*, NeuroImage, **47**:p. S102, organization for Human Brain Mapping 2009 Annual Meeting

[67] V. Fonov, A. C. Evans, K. Botteron, C. R. Almli, R. C. McKinstry, & D. L. Collins (2011) *Unbiased average age-appropriate atlases for pediatric studies*, NeuroImage, **54(1)**:pp. 313–327

[68] D. L. Pham (2001) *Spatial models for fuzzy clustering*, Computer Vision and Image Understanding, **84(2)**:pp. 285–297

[69] X. Li, P. S. Morgan, J. Ashburner, J. Smith, & C. Rorden (2016) *The first step for neuroimaging data analysis: DICOM to NIfTI conversion*, Journal of Neuroscience Methods, **264**:pp. 47–56

[70] B. Lowekamp, D. Chen, L. Ibanez, & D. Blezek (2013) *The design of SimpleITK*, Frontiers in Neuroinformatics, **7**:p. 45

[71] J. C. Mazziotta, A. W. Toga, A. Evans, P. Fox, & J. Lancaster (1995) *A probabilistic atlas of the human brain: Theory and rationale for its development: The International Consortium for Brain Mapping (ICBM)*, NeuroImage, **2(2, Part A)**:pp. 89–101

[72] B. B. Avants, N. Tustison, G. Song, et al. (2009) *Advanced normalization tools (ANTs)*, Insight j, **2(365)**:pp. 1–35

[73] S. Yang, Z. Yang, K. Fischer, K. Zhong, J. Stadler, F. Godenschweger, J. Steiner, H.-J. Heinze, H.-G. Bernstein, B. Bogerts, C. Mawrin, D. Reutens, O. Speck, & M. Walter (2013) *Integration of ultra-high field MRI and histology for connectome based research of brain disorders*, Frontiers in Neuroanatomy, **7**

[74] J. M. Huntenburg, C. J. Steele, & P.-L. Bazin (2018) *Nighres: processing tools for high-resolution neuroimaging*, GigaScience, **7(7)**:p. giy082

[75] L. R. Dice (1945) *Measures of the amount of ecologic association between species*, Ecology, **26(3)**:pp. 297–302

[76] H. Zunair, A. Rahman, N. Mohammed, & J. P. Cohen (2020) *Uniformizing techniques to process CT scans with 3D CNNs for tuberculosis prediction*, I. Rekik, E. Adeli, S. H. Park, & M. d. C. Valdés Hernández (Eds.) *Predictive Intelligence in Medicine*, pp. 156–168, Springer International Publishing, Cham

[77] D. Maturana & S. Scherer (2015) *VoxNet: A 3D convolutional neural network for real-time object recognition*, *2015 IEEE/RSJ International Conference on Intelligent Robots and Systems (IROS)*, pp. 922–928

[78] S. Ioffe & C. Szegedy (2015) *Batch normalization: Accelerating deep network training by reducing internal covariate shift*, F. Bach & D. Blei (Eds.) *Proceedings of the 32nd International Conference on Machine Learning*, vol. 37 of *Proceedings of Machine Learning Research*, pp. 448–456, PMLR, Lille, France

[79] D. P. Kingma & J. Ba (2017) *Adam: A method for stochastic optimization*, arXiv preprint arXiv:1412.6980

[80] A. Carass, S. Roy, A. Jog, J. L. Cuzzocreo, E. Magrath, A. Gherman, J. Button, J. Nguyen, F. Prados, C. H. Sudre, M. Jorge Cardoso, N. Cawley, O. Ciccarelli, C. A. Wheeler-Kingshott, S. Ourselin, L. Catanese, H. Deshpande, P. Maurel, O. Commowick, C. Barillot, X. Tomas-Fernandez, S. K. Warfield, S. Vaidya, A. Chunduru, R. Muthuganapathy, G. Krishnamurthi, A. Jesson, T. Arbel, O. Maier, H. Handels, L. O. Iheme, D. Unay, S. Jain, D. M. Sima, D. Smeets, M. Ghafoorian, B. Platel, A. Birenbaum, H. Greenspan, P.-L. Bazin, P. A. Calabresi, C. M. Crainiceanu, L. M. Ellingsen, D. S. Reich, J. L. Prince, & D. L. Pham (2017) *Longitudinal multiple sclerosis lesion segmentation: Resource and challenge*, NeuroImage, **148**:pp. 77–102

[81] Ž. Lesjak, A. Galimzianova, A. Koren, M. Lukin, F. Pernuš, B. Likar, & Ž. Špiclin (2018) *A novel public MR image dataset of multiple sclerosis patients with lesion segmentations based on multi-rater consensus*, Neuroinformatics, **16**:pp. 51–63

[82] O. Commowick, A. Istace, M. Kain, B. Laurent, F. Leray, M. Simon, S. C. Pop, P. Girard, R. Améli, J.-C. Ferré, A. Kerbrat, T. Tourdias, F. Cervenansky, T. Glatard, J. Beaumont, S. Doyle, F. Forbes,

J. Knight, A. Khademi, A. Mahbod, C. Wang, R. McKinley, F. Wagner, J. Muschelli, E. Sweeney, E. Roura, X. Lladó, M. M. Santos, W. P. Santos, A. G. Silva-Filho, X. Tomas-Fernandez, H. Urien, I. Bloch, S. Valverde, M. Cabezas, F. J. Vera-Olmos, N. Malpica, C. Guttmann, S. Vukusic, G. Edan, M. Dojat, M. Styner, S. K. Warfield, F. Cotton, & C. Barillot (2018) *Objective evaluation of multiple sclerosis lesion segmentation using a data management and processing infrastructure*, Scientific Reports, **8(1)**:p. 13650

[83] O. Commowick, F. Cervenansky, F. Cotton, & M. Dojat (Eds.) (2021) *MSSEG-2 challenge proceedings: Multiple sclerosis new lesions segmentation challenge using a data management and processing infrastructure*, Strasbourg, France

[84] A. M. Muslim, S. Mashohor, G. A. Gawwam, R. Mahmud, M. binti Hanafi, O. Alnuaimi, R. Josephine, & A. D. Almutairi (2022) *Brain MRI dataset of multiple sclerosis with consensus manual lesion segmentation and patient meta information*, Data in Brief, **42**:p. 108139

[85] R. Kötter, J. Mazziotta, A. Toga, A. Evans, P. Fox, J. Lancaster, K. Zilles, R. Woods, T. Paus, G. Simpson, B. Pike, C. Holmes, L. Collins, P. Thompson, D. MacDonald, M. Iacoboni, T. Schormann, K. Amunts, N. Palomero-Gallagher, S. Geyer, L. Parsons, K. Narr, N. Kabani, G. L. Goualher, D. Boomsma, T. Cannon, R. Kawashima, & B. Mazoyer (2001) *A probabilistic atlas and reference system for the human brain: International Consortium for Brain Mapping (ICBM)*, Philosophical Transactions of the Royal Society of London. Series B: Biological Sciences, **356(1412)**:pp. 1293–1322

[86] P. J. LaMontagne, T. L. Benzinger, J. C. Morris, S. Keefe, R. Hornbeck, C. Xiong, E. Grant, J. Hassenstab, K. Moulder, A. G. Vlassenko, M. E. Raichle, C. Cruchaga, & D. Marcus (2019) *OASIS-3: Longitudinal neuroimaging, clinical, and cognitive dataset for normal aging and alzheimer disease*, medRxiv

[87] C. R. Jack Jr., M. A. Bernstein, N. C. Fox, P. Thompson, G. Alexander, D. Harvey, B. Borowski, P. J. Britson, J. L. Whitwell, C. Ward, A. M. Dale, J. P. Felmlee, J. L. Gunter, D. L. Hill, R. Killiany, N. Schuff, S. Fox-Bosetti, C. Lin, C. Studholme, C. S. DeCarli, G. Krueger, H. A. Ward, G. J. Metzger, K. T. Scott, R. Mallozzi, D. Blezek, J. Levy, J. P. Debbins, A. S. Fleisher, M. Albert, R. Green, G. Bartzokis, G. Glover, J. Mugler, & M. W. Weiner (2008) *The Alzheimer's disease neuroimaging initiative (ADNI): MRI methods*, Journal of Magnetic Resonance Imaging, **27(4)**:pp. 685–691

[88] I. Mérida, J. Jung, S. Bouvard, D. Le Bars, S. Lancelot, F. Lavenne, C. Bouillot, J. Redouté, A. Hammers, & N. Costes (2021) *CERMEP-IDB-MRXFDG: a database of 37 normal adult human brain [^{18}F]FDG PET, T1 and FLAIR MRI, and CT images available for research*, EJNMMI Research, **11(1)**:p. 91

[89] S. Vukusic, R. Casey, F. Rollot, B. Brochet, J. Pelletier, D.-A. Laplaud, J. D. Sèze, F. Cotton, T. Moreau, B. Stankoff, B. Fontaine, F. Guillemin, M. Debouverie, & M. Clanet (2020) *Observatoire Français de la Sclérose en Plaques (OFSEP): A unique multimodal nationwide MS registry in France*, Multiple Sclerosis Journal, **26(1)**:pp. 118–122, pMID: 30541380

[90] C. Confavreux, D. A. Compston, O. R. Hommes, W. I. McDonald, & A. J. Thompson (1992) *EDMUS, a European database for multiple sclerosis.*, Journal of Neurology, Neurosurgery & Psychiatry, **55(8)**:pp. 671–676

[91] A. Akshay, M. Abedi, N. Shekarchizadeh, F. C. Burkhard, M. Katoch, A. Bigger-Allen, R. M. Adam, K. Monastyrskaya, & A. Hashemi Gheinani (2023) *MLcps: machine learning cumulative performance score for classification problems*, GigaScience, **12**:p. giad108

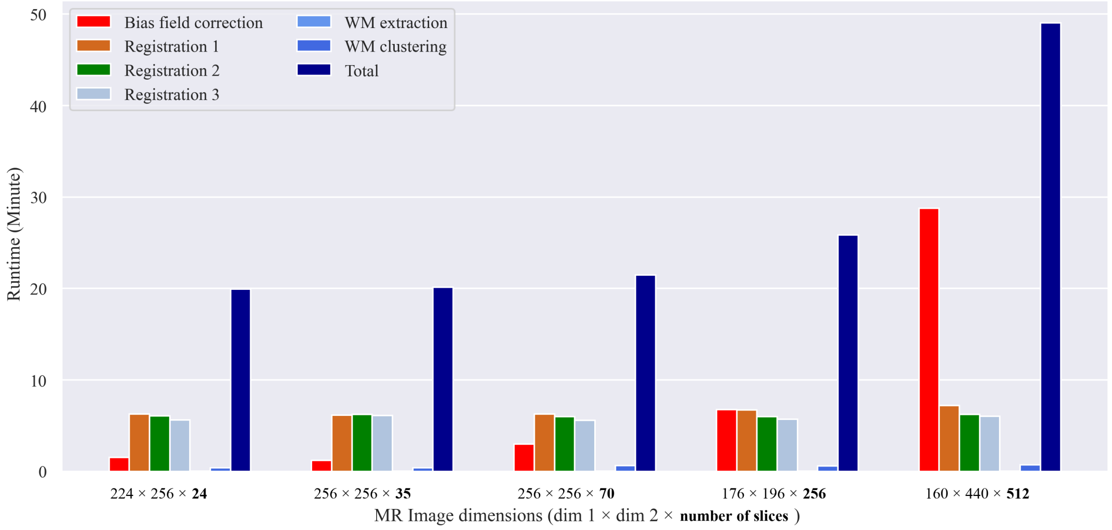

Supplementary Figure S 1: Average required time for preprocessing and intensity clustering of five sample MRI dimensions.

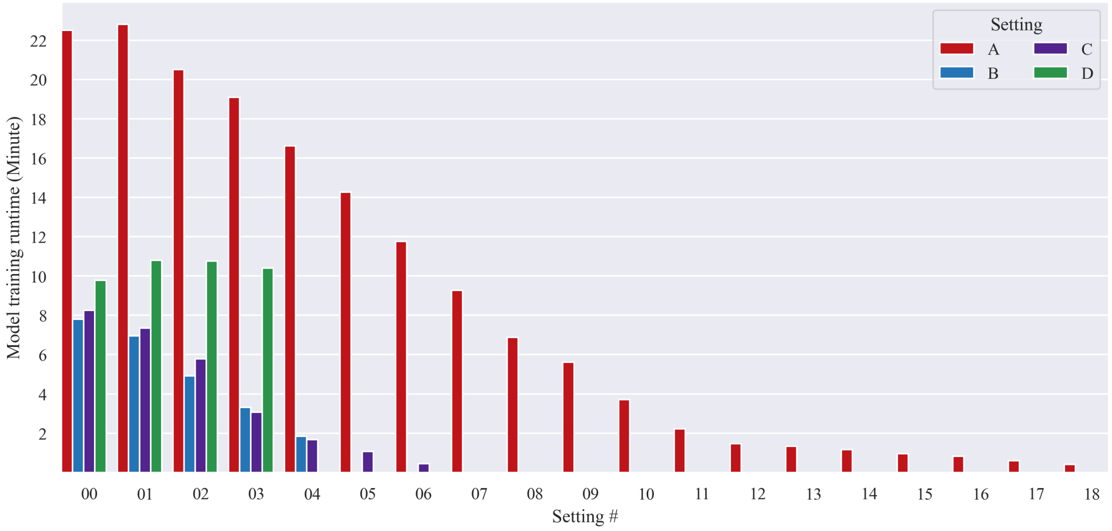

Supplementary Figure S 2: Average required time for training the CNN model of each experimental setting.

Supplementary Table S 1: Number of MRIs used for training (Tr), validation (V), and test (T) sets from each *dataset* in experimental settings $A00$ to $A18$

**Dataset**		**Set**	**Setting $A\#$**																		
**Label***	**Name**		**00**	**01**	**02**	**03**	**04**	**05**	**06**	**07**	**08**	**09**	**10**	**11**	**12**	**13**	**14**	**15**	**16**	**17**	**18**
+	ISBI	Tr	8	7	6	5	4	3	2	1	0	0	0	0	0	0	0	0	0	0	0
		V	1	1	1	1	1	1	1	1	0	0	0	0	0	0	0	0	0	0	0
		T	2	2	2	2	2	2	2	2	2	2	2	2	2	2	2	2	2	2	2
	UMCL	Tr	8	7	6	5	4	3	2	1	0	0	0	0	0	0	0	0	0	0	0
		V	1	1	1	1	1	1	1	1	0	0	0	0	0	0	0	0	0	0	0
		T	2	2	2	2	2	2	2	2	2	2	2	2	2	2	2	2	2	2	2
	MSSEG	Tr	8	7	6	5	4	3	2	1	0	0	0	0	0	0	0	0	0	0	0
		V	1	1	1	1	1	1	1	1	0	0	0	0	0	0	0	0	0	0	0
		T	2	2	2	2	2	2	2	2	2	2	2	2	2	2	2	2	2	2	2
	MSSEG-2	Tr	8	7	6	5	4	3	2	1	0	0	0	0	0	0	0	0	0	0	0
		V	1	1	1	1	1	1	1	1	0	0	0	0	0	0	0	0	0	0	0
		T	2	2	2	2	2	2	2	2	2	2	2	2	2	2	2	2	2	2	2
	BTH	Tr	6	5	4	3	2	1	0	0	0	0	0	0	0	0	0	0	0	0	0
		V	1	1	1	1	1	1	0	0	0	0	0	0	0	0	0	0	0	0	0
		T	2	2	2	2	2	2	2	2	2	2	2	2	2	2	2	2	2	2	2
	OASIS-3	Tr	10	9	8	7	6	5	4	3	2	1	0	0	0	0	0	0	0	0	0
		V	2	2	2	1	1	1	1	1	1	1	0	0	0	0	0	0	0	0	0
		T	2	2	2	2	2	2	2	2	2	2	2	2	2	2	2	2	2	2	2
	ADNI 3	Tr	39	35	31	28	26	23	21	19	17	14	12	9	7	6	5	4	3	2	1
		V	6	6	6	6	6	6	5	5	5	5	4	4	3	3	3	2	2	1	1
		T	10	10	10	10	10	10	10	10	10	10	10	10	10	10	10	10	10	10	10
−	ICBM	Tr	3	2	1	0	0	0	0	0	0	0	0	0	0	0	0	0	0	0	0
		V	1	1	1	0	0	0	0	0	0	0	0	0	0	0	0	0	0	0	0
		T	1	1	1	1	1	1	1	1	1	1	1	1	1	1	1	1	1	1	1
	OASIS-3	Tr	65	58	51	45	39	32	26	21	15	12	10	8	7	6	5	4	3	2	1
		V	9	9	9	9	9	9	8	8	5	5	3	3	3	3	3	2	2	1	1
		T	16	16	16	16	16	16	16	16	16	16	16	16	16	16	16	16	16	16	16
	CERMEP	Tr	19	17	15	13	11	9	7	5	4	3	2	1	0	0	0	0	0	0	0
		V	3	3	3	3	3	3	2	2	1	1	1	1	0	0	0	0	0	0	0
		T	5	5	5	5	5	5	5	5	5	5	5	5	5	5	5	5	5	5	5
+	Total	Tr	87	77	67	58	50	41	33	26	19	15	12	9	7	6	5	4	3	2	1
		V	13	13	13	12	12	12	10	10	6	6	4	4	3	3	3	2	2	1	1
		T	22	22	22	22	22	22	22	22	22	22	22	22	22	22	22	22	22	22	22
−	Total	Tr	87	77	67	58	50	41	33	26	19	15	12	9	7	6	5	4	3	2	1
		V	13	13	13	12	12	12	10	10	6	6	4	4	3	3	3	2	2	1	1
		T	22	22	22	22	22	22	22	22	22	22	22	22	22	22	22	22	22	22	22
**Total**			244	224	204	184	168	150	130	116	94	86	76	70	64	62	60	56	54	50	48

* With (+) and without (−) WM abnormality

Supplementary Table S 2: Number of MRIs used for training (Tr), validation (V), and test (T) sets from each MRI acquisition *protocol* in experimental settings $B00$ to $B04$

**Protocol**		**Set**	**Setting**				
**Label***	**Name**		$B00$	$B01$	$B02$	$B03$	$B04$
+	Sie_Tri_30_Prot1	Tr	5	4	3	2	1
		V	1	1	1	1	1
		T	1	1	1	1	1
	Phi_Ing_30_NA	Tr	5	4	3	2	1
		V	1	1	1	1	1
		T	1	1	1	1	1
	Phi_NA_30_Prot1	Tr	5	4	3	2	1
		V	1	1	1	1	1
		T	1	1	1	1	1
	Sie_Aer_15_Prot1	Tr	5	4	3	2	1
		V	1	1	1	1	1
		T	1	1	1	1	1
	GeE_Dis_30_Prot3	Tr	5	4	3	2	1
		V	1	1	1	1	1
		T	1	1	1	1	1
−	Sie_Bio_30_Prot1	Tr	5	4	3	2	1
		V	1	1	1	1	1
		T	1	1	1	1	1
	Sie_Son_15_Prot1	Tr	5	4	3	2	1
		V	1	1	1	1	1
		T	1	1	1	1	1
	Sie_MaV_30_Prot2	Tr	5	4	3	2	1
		V	1	1	1	1	1
		T	1	1	1	1	1
	Sie_MaV_30_Prot1	Tr	5	4	3	2	1
		V	1	1	1	1	1
		T	1	1	1	1	1
	Sie_TrT_30_Prot2	Tr	5	4	3	2	1
		V	1	1	1	1	1
		T	1	1	1	1	1
+	Total	Tr	25	20	15	10	5
		V	5	5	5	5	5
		T	5	5	5	5	5
−	Total	Tr	25	20	15	10	5
		V	5	5	5	5	5
		T	5	5	5	5	5
**Total**			70	60	50	40	30

* With (+) and without (−) WM abnormality

Supplementary Table S 3: Number of MRIs used for training (Tr), validation (V), and test (T) sets from each MRI acquisition *protocol* in experimental settings $C00$ to $C06$. The two protocols in boldface are used only as test data. These two protocols are changed in 10 different cases.

Protocol		Set	Setting						
**Label***	**Name**		$C00$	$C01$	$C02$	$C03$	$C04$	$C05$	$C06$
+	Phi_Ing_30_NA	Tr	7	6	5	4	3	2	1
		V	1	1	1	1	1	1	1
		T	0	0	0	0	0	0	0
	GeE_Dis_30_Prot3	Tr	7	6	5	4	3	2	1
		V	1	1	1	1	1	1	1
		T	0	0	0	0	0	0	0
	Sie_Pri_30_Prot1	Tr	6	5	4	3	2	1	0
		V	2	2	2	1	1	1	0
		T	0	0	0	0	0	0	0
	Sie_Ver_30_Prot1	Tr	6	5	4	3	2	1	0
		V	2	1	1	1	1	1	0
		T	0	0	0	0	0	0	0
	**Phi_Ing_30_Prot2**	Tr	0	0	0	0	0	0	0
		V	0	0	0	0	0	0	0
		T	5	5	5	5	5	5	5
−	Sie_MaV_30_Prot1	Tr	6	5	4	3	2	1	0
		V	1	1	1	1	1	1	0
		T	0	0	0	0	0	0	0
	Sie_Son_15_Prot1	Tr	20	17	14	11	8	5	2
		V	5	4	4	3	3	3	2
		T	0	0	0	0	0	0	0
	**Sie_TrT_30_Prot1**	Tr	0	0	0	0	0	0	0
		V	0	0	0	0	0	0	0
		T	5	5	5	5	5	5	5
+	Total	Tr	26	22	18	14	10	6	2
		V	6	5	5	4	4	4	2
		T	5	5	5	5	5	5	5
−	Total	Tr	26	22	18	14	10	6	2
		V	6	5	5	4	4	4	2
		T	5	5	5	5	5	5	5
**Total**			74	64	56	46	38	30	18

* With (+) and without (−) WM abnormality

Supplementary Table S 4: Number of MRIs used for training (Tr), validation (V), and test (T) sets from each MRI acquisition *protocol* in experimental settings $D00$ to $D03$

**Protocol**		**Set**	**Setting**			
**Label***	**Name**		$D00$	$D01$	$D02$	$D03$
+	Sie_Tri_30_Prot1	Tr	21	12	9	8
		V	3	2	2	1
		T	6	3	2	2
	Phi_Ing_30_NA	Tr	15	12	9	8
		V	2	2	1	1
		T	4	3	3	2
	Phi_NA_30_Prot1	Tr	0	12	9	7
		V	0	1	1	1
		T	0	4	3	2
	Sie_Aer_15_Prot1	Tr	0	0	9	7
		V	0	0	1	1
		T	0	0	2	2
	GeE_Dis_30_Prot3	Tr	0	0	0	6
		V	0	0	0	1
		T	0	0	0	2
−	Sie_Bio_30_Prot1	Tr	21	124	11	9
		V	3	2	2	1
		T	6	3	2	3
	Sie_Son_15_Prot1	Tr	15	12	10	8
		V	2	2	1	1
		T	4	3	4	3
	Sie_MaV_30_Prot2	Tr	0	12	10	9
		V	0	1	1	1
		T	0	4	3	2
	Sie_MaV_30_Prot1	Tr	0	0	5	5
		V	0	0	1	1
		T	0	0	1	1
	Sie_TrT_30_Prot2	Tr	0	0	0	5
		V	0	0	0	1
		T	0	0	0	1
+	Total	Tr	36	36	36	36
		V	5	5	5	5
		T	10	10	10	10
−	Total	Tr	36	36	36	36
		V	5	5	5	5
		T	10	10	10	10
**Total**			102	102	102	102

* With (+) and without (−) WM abnormality

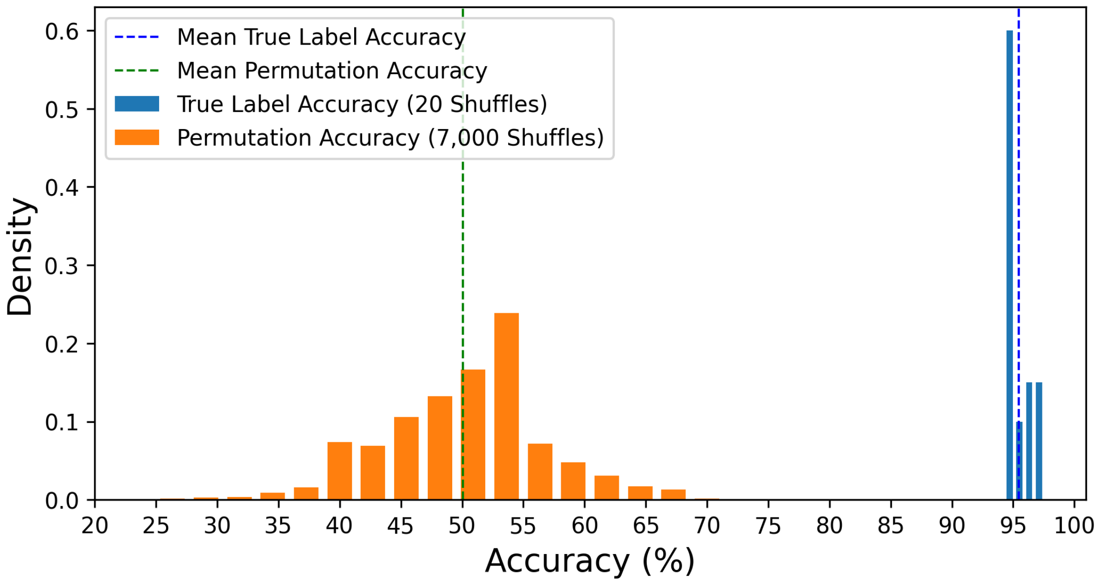

Supplementary Figure S 3: Normalized histogram of accuracy values from 7,000 permutations of shuffled labels (orange) and from the 20 data shuffles of the original labels (blue). Dashed lines indicate the mean accuracy for each group, highlighting the significant difference between chance-level accuracy and the model's performance with true labels.

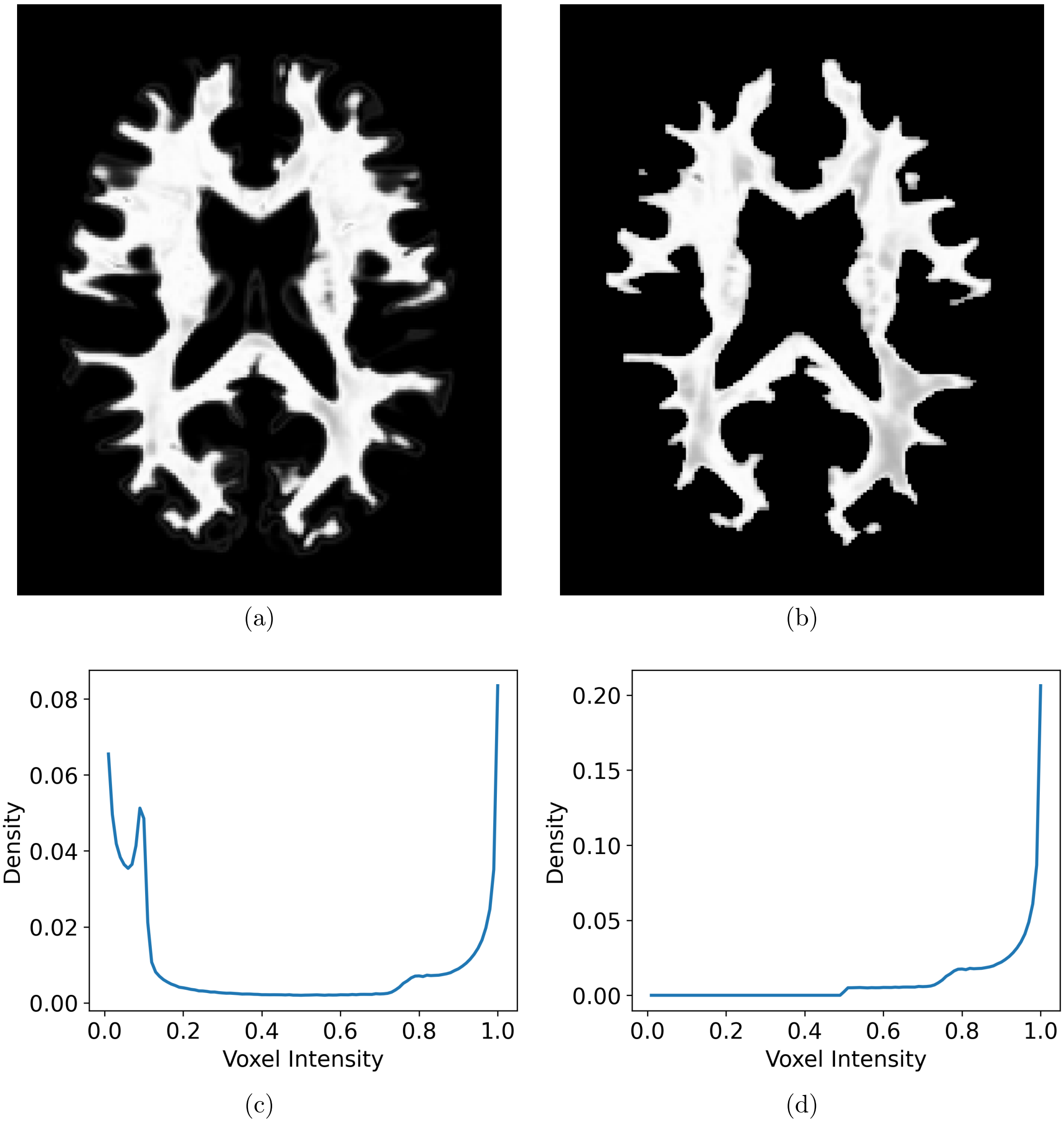

Supplementary Figure S 4: The WM intensity cluster of a sample MRI (a) before and (b) after applying a threshold value of 0.5. The normalized histograms of their 99% upper percentile are shown in (c) and (d), respectively.

Manuscript_changes_highlighted

Click here to access/download;Manuscript;HeteroMRI_rev1_changes.pdf

HeteroMRI: Robust white matter abnormality classification across multi-scanner MRI data

Masoud Abedi [1,2,3,†], Navid Shekarchizadeh [2,3,4,†,§], Pierre-Louis Bazin [5], Nico Scherf [4,6], Julia Lier [7,8], Christa-Caroline Bergner[7,8], for the Alzheimer's Disease Neuroimaging Initiative[*] , Wolfgang Köhler[7,8,‡], and Toralf Kirsten [1,2,3,4,‡]

[1]Faculty Applied Computer and Bio Sciences, Mittweida University of Applied Sciences, Mittweida, Germany

[2]Department for Medical Data Science, Leipzig University Medical Center, Leipzig, Germany

[3]Institute for Medical Informatics, Statistics, and Epidemiology (IMISE), Leipzig University, Leipzig, Germany

[4]Center for Scalable Data Analytics and Artificial Intelligence (ScaDS.AI) Dresden/Leipzig, Leipzig University, Leipzig, Germany

[5]Full brain picture Analytics, Leiden, The Netherlands

[6]Neural Data Science and Statistical Computing, Max Planck Institute for Human Cognitive and Brain Sciences, Leipzig, Germany

[7]Department of Neurology, Leipzig University Medical Center, Leipzig, Germany

[8]Myelin Research Center (MRC) Leipzig, Department of Neurology, Leipzig University Medical Center, Leipzig, Germany

[§]Correspondence address. Navid Shekarchizadeh, ScaDS.AI, Humboldtstraße 25, 04105 Leipzig, Germany. E-mail: navid.shekarchizadeh@uni-leipzig.de

[†]Equal contribution

[‡]Joint senior authorship

Abstract

Background

~~A common application of~~ Magnetic Resonance Imaging (MRI) is ~~the analysis of~~ commonly used for analyzing white matter abnormalities in the human brain. Integrating machine learning ~~techniques in MRI data~~ into MRI analysis can enhance diagnostic processes. However, ~~utilizing machine learning techniques is restricted when the available~~ the application of such techniques for white matter analysis in clinical practice is often limited when MRI data is multi-scanner ~~and multi-protocol (i.e.heterogeneous). This issue makes introducing such methods to clinical practice improbable.~~ (i.e., heterogeneous), particularly in scenarios with limited data, as seen in rare diseases. Therefore, it is crucial to develop methods that are ~~ideally~~ highly independent of the MRI scanner and acquisition protocol.

[*]A part of the data used in preparation of this article were obtained from the Alzheimer's Disease Neuroimaging Initiative (ADNI) database (adni.loni.usc.edu). As such, the investigators within the ADNI contributed to the design and implementation of ADNI and/or provided data but did not participate in analysis or writing of this report. A complete listing of ADNI investigators can be found at: http://adni.loni.usc.edu/wp-content/uploads/how_to_apply/ADNI_Acknowledgement_List.pdf

Results

This study introduces *HeteroMRI*, a deep-learning method for ~~analyzing MRI datasets incorporating various MRI protocols and scanners.~~ classifying MRIs based on white matter abnormalities. Most importantly, HeteroMRI mitigates the effects of data heterogeneity on classification performance. Herein, HeteroMRI is employed to detect brain MRIs with white matter abnormalities. This method utilizes intensity clustering of the white matter tissue to minimize the effects of the heterogeneity of MRIs. ~~Herein, the presented method is employed for developing a binary classifier that identifies brain MRIs with white matter abnormalities.~~ MRI data from nine public datasets with 32 MRI protocols is included. By using 200 MRIs for training the model, the binary classifier achieves an average accuracy of 96%. Furthermore, the method is evaluated ~~with limited data , simulating scenarios~~ in limited data scenarios, simulating conditions of rare diseases~~where only limited data is available~~. By reducing the data by 64% and 75%, the model's accuracy has a 4% and 15% decrease, respectively.

Conclusions

The presented method opens ~~up~~ new avenues for ~~the analysis of multi-protocol and multi-scanner MRI data . This~~ white matter abnormality-related classification of heterogeneous MRI data without additional machine learning methods to minimize MRI heterogeneity. This classification approach demonstrates a high degree of independence from the MRI scanner and protocol, ~~achieving superior performance even in limited data scenarios without needing machine learning techniques to minimize MRI heterogeneity~~while also proving to be generalizable to unseen MRI protocols.

Keywords — Brain MRI classification, Multi-scanner MRI, Multi-protocol MRI, Intensity clustering, White matter abnormality, Rare disease, Convolutional neural network

1 Introduction

Magnetic Resonance Imaging (MRI) is widely used in clinics and hospitals to diagnose and follow up neurological diseases by generating images of the central nervous system including the brain. MRI provides a clear contrast between the different tissues of the brain, including ~~White Matter~~ white matter (WM) and ~~Gray Matter~~ gray matter (GM) [1]. Detection and assessment of WM abnormalities in demyelinating or neurodegenerative diseases are an important application of MRI in daily clinical practice [2]. An excellent contrast for visualizing WM abnormalities is provided by the ~~FLAIR (~~Fluid-Attenuated Inversion Recovery (FLAIR) imaging technique making the abnormalities stand out from the surrounding normal brain tissue. FLAIR is a T2-weighted imaging technique in which the signal from cerebrospinal fluid (CSF) is suppressed. By reducing the interference of CSF, the detection of WM abnormalities becomes easier, as these abnormalities may appear adjacent to CSF-filled spaces [3]. MRI provides a three-dimensional (3D) view of the brain and other anatomical structures, making it possible to accurately assess and measure the volume of WM lesions. Volume measurements of the lesions help clinicians to track the disease progression and the effectiveness of the treatments. Moreover, the pattern and volume of lesions are used in research studies to investigate the behavior of different diseases which ultimately contribute to image-based diag-

nosis of demyelinating disorders [4].

In recent years, Artificial Intelligence (AI) has revolutionized the medical imaging domain, bringing substantial benefits to the analysis of such data [5, 6]. Automating certain aspects of the image analysis process reduces the need for repetitive and time-consuming tasks such as lesion segmentation. This allows healthcare specialists to allocate their expertise to more critical aspects of clinical practice and research. Moreover, manual analysis such as segmentation of MRI data, especially when dealing with complex structures like demyelinating lesions, is resource-intensive and prone to examiner-based variability. AI allows for rapid and consistent analysis across a large amount of images. A large and growing body of literature has investigated the utilization of Machine Learning (ML) [7, 8] and Deep Learning (DL) [9, 10] models in analyzing medical images.

In brain MRI analysis, studies have focused on developing different ~~AI~~ models using ML techniques, e.g. for disease classification [11, 12, 13], WM lesion segmentation [14, 15], tumor detection and grading [16, 17], stroke lesion detection and segmentation [18, 19], brain age prediction [20, 21], tracking disease progression in neurodegenerative disorders using longitudinal MRI data [22, 23], and automated segmentation of brain tissues, such as GM, WM, and CSF, for quantitative analysis and volumetric measurements [24, 25]. Before ~~analysing~~ analyzing brain MRIs, certain preprocessing steps are commonly performed to optimize the image data for further analysis and interpretation. AI models and mathematical algorithms are generally used for preprocessing steps including image registration [26], brain extraction or skull stripping [27, 28], image denoising [29, 30, 31], intensity normalization [32, 33], bias field (also called inhomogeneity) correction [34], and MRI interpolation [35].~~For protecting the privacy and anonymity of the individuals whose brain images are being analyzed, defacing [36] algorithms are employed.~~

~~In many types of AI methods mentioned above for~~ A challenge in using MRI data in ML/DL models is the variability of MRIs across different sites and scanners. It is shown that scanner differences leads to significant biases in automated MS lesion volumetric analyses, even when the scanner manufacturer and acquisition protocol are consistent [37]. The variabilities due to different scanners and acquisition protocols are often greater than the biological variabilities [38, 39, 40, ?, ?, ?]. High-capacity classifiers, such as deep neural networks, often struggle to produce consistent outcomes when applied to multi-scanner data. This limitation is caused by the model's tendency to overfit to non-bioligical variations, thus the model fails to detect desired biological features or to generalize well across MRI data from unseen scanners [?].

One common strategy to address the above-mentioned challenges is to provide *standardized* images for automated MRI data analysis, ~~including lesion segmentation , it is highly recommended that all the images are *standardized* [41, 42, 43]. Here,~~ especially for WM lesion segmentation models, e.g. as in [?]. By "standardized"~~means the images~~, we refer to images that have been acquired with the same acquisition protocol ~~, with the same magnetic field strength, and preferably with~~ and the same MR scanner model. Acquisition protocol refers to a set of procedures and parameters, e.g. Echo Time (TE), Repetition Time (TR), and Inversion Time (TI), used to acquire the images. For example, in the UK Biobank[?], all

the MRI data are acquired following highly standardized procedures to be used in future studies. Using a standardized dataset ~~ensures that the images have similar~~ results in consistent image quality, ~~similar~~ comparable intensity range for each tissue type, and uniform spatial characteristics, ~~and similar spatial characteristics. Moreover, the contrast among the brain tissues would be uniform across the dataset [37, 41] . Furthermore, this consistency allows the model to effectively recognize and learn the~~ although some variability may still exist. This consistency may assist ML models in effectively recognizing and learning disease patterns and features across ~~the~~ images, leading to more accurate and reliable results.

~~The necessity for a standardized dataset poses a significant limitation in utilizing routinely available MRIs for analysis. Numerous MRIs are produced daily at hospitals or clinics. However, since the images are usually not standardized across centers or even within one, their use for an AI-based analysis is limited, restricting the clinical applicability of AI methods. Moreover,~~ However, generating a standardized dataset imposes additional costs and requires a well-structured organization at the medical center(s).

~~The variability of MRIs across different sites was studied in Ref. [37]. In this study, a multiple sclerosis (MS) patient underwent imaging at seven different sites, all following a similar acquisition protocol. Notably, clear differences in volumetric measurements were observed, showing significant variations in both manual and automatic WM lesion segmentations across sites. This variability persisted even among the sites that had scanners from the same manufacturer and model. Furthermore, also rescanning the patient on the same scanner on the same day showed variations in the lesion volume measurements. Such systematic differences are also referred to as batch effect in the literature [44]~~In addition, even by providing standardized data for training a model, many DL models fail to generalize to images that are acquired with different scanners or protocols than the ones included in the training set [43]. These conditions pose a significant limitation in the applicability of DL models in clinical settings since the images are usually not standardized across centers or even across scanners within one center. Therefore, analysis methods that are highly independent of the MRI scanner and acquisition protocol are preferred.

~~Literature concerning~~ Another strategy for handling the MRI data heterogeneity is to develop techniques for removing scanner and protocol effects from ~~heterogeneous MR images is limited~~the available MRIs. Such methods are referred to as *harmonization*. Literature concerning harmonization techniques is relatively limited. Harmonization involves considerable complexities and challenges due to the limited understanding of scanner effects, and the absence of ~~standardized~~ standard criteria for assessing scanner effects and evaluating the harmonization process [45]. The proposed MRI harmonization methods in the literature are ~~either based on statistics or learning (AI). The methods based on statistics alter~~ generally divided into statistical approaches or ML/DL techniques. One group of statistical methods adjusts the intensity distribution~~in the image either via~~ , by normalizing the intensity [32, 46, 47, 48] or ~~adjusting the batch effect [40, 39, 38]~~by harmonizing values measured from the images (such as cortical thickness) by adjusting the so-called batch effect across different scanners [40, 39, 38, ?, ?, ?]. Nonetheless, these ~~techniques are usually restricted to harmonizing the entire image and lack the capacity for harmonization at the brain tissue level. The intensity normalization methodscan be considered more as data preprocessing techniques (for scaling the intensity distribution of the images before training the AI models) rather than an MRI harmonization method because if the heterogeneity is too severe, these statistical models will~~

~~not be able to harmonize the variations caused by the different protocols. The harmonization methods based on learning have the goal of learning the~~ statistical harmonization techniques are effective only in specific applications. For instance, a study has shown that ComBat-based harmonization methods [39, 38] fail to make any noticeable improvement in the performance of DL models in disease classification tasks [?].

The ML/DL harmonization methods aim at learning the scanner and protocol effects and removing them from the multi-center ~~images. The learning-based MRI harmonization models~~ MRIs. Such methods include approaches that are built upon supervised ML [49, 50], supervised DL [45, 51, 52, 53], and unsupervised DL [54, 55] methods. Supervised ML techniques are commonly employed to predict harmonized images by training regression models incorporating manually selected features. On the contrary, DL techniques automatically capture relevant features for the harmonization task. Recently, a novel class of models has been proposed, which integrates both statistical and DL approaches [56]. For an extensive review of MRI harmonization methods, refer to Ref. [44].

~~Supervised DL methods often rely on training data derived from human patients at different sites, forming what is known as a "matched" dataset. This approach, as exemplified in Ref. [45], involves harmonizing MRIs taken of the same individuals across different locations. This method is categorized as supervised DL because it allows the model to observe variations in brain MRIs from the same individual across different scanners. A noteworthy aspect of the methodin Ref.~~ There are significant limitations in the application of the ML/DL harmonization methods. As an example, a supervised method [45] is ~~the use of~~ trained using a relatively small dataset ~~comprising~~ of MRIs from 18 individuals~~. However, a significant limitation arises in its exclusive applicability to matched datasets. Essentially, this method is not suitable for datasets containing MRIs from different patients across diverse medical centers.~~

~~While unsupervised DL models do not rely on a matched dataset, such models~~ , demonstrating its ability to work effectively with limited training data. However, the applicability of the method is limited to datasets that include the MRIs of similar patients from different scanners. Meanwhile, unsupervised methods usually have other limitations. For instance, the method proposed in Ref. [54] harmonizes the images taken by different scanners by removing the non-biological site differences. However, its applicability is restricted to datasets with ~~uniform image protocols, as~~ the acquisition protocols ~~were~~ standardized across all scanners. Another unsupervised ~~DL model, as presented in Ref. [55] , addresses the harmonization of multi-contrast multi-site MRIs. Their method~~ method [55] necessitates multi-contrast images of the same subject within a single imaging session, with training data acquired from 10 scanners following a standardized protocol on each.

~~Despite the methods that have been proposed for MRI harmonization~~In summary, there is no universal or standardized procedure for effectively harmonizing ~~multi-site and multi-protocol~~ heterogeneous MRI data. ~~Notably, as~~ The harmonization of MRI data remains a complex and unsolved challenge, given the variations that come with the nature of MRI technology. As mentioned in Ref. [41], ~~there have been no studies conducted to evaluate~~ no studies have so far evaluated the impact of ~~a change~~ changes in MR acquisition parameters (~~including~~ such as TE or TR) on the assessment of brain WM lesions, even when using the same MR scanner and sequence. ~~The harmonization of MRI data continues to be a complex and~~

~~unsolved issue, given the variations that come with the nature of MRI technology~~Moreover, even assuming successful harmonization of the MRI data for use in training DL models, these models must also achieve robust generalizability to new MRI data to ensure their applicability in clinical settings.

~~The application of AI in~~

On a different note, the context of rare diseases has specific challenges and limitations. Data availability ~~for rare diseases~~ is extremely limited, which severely restricts the ~~development of AI modelsfor such diseases. In~~ application of ML/DL approaches to these diseases, including both predictive models, such as classifiers, and harmonization methods. For example, in the case of ~~brain MRI data for neurological rare diseases like~~ leukodystrophies [57]), the ~~images~~ brain MRIs are gathered from various clinical centers over a long period, making it infeasible to even create a standardized dataset. ~~Datasets created by collecting MRIs from multiple centers~~ Such datasets are not only small in size but also highly heterogeneous in terms of MRI scanners and protocols. These conditions significantly hinder the applicability of ~~AI methods for analyzing the data. In addition, the existing literature lacks MRI harmonization methods that can handle such high levels of heterogeneity in a small dataset.~~ conventional ML methods to rare diseases.

Herein, we present HeteroMRI, an approach for classifying brain MRIs based on WM abnormalities while mitigating the heterogeneity effects of the images acquired from multiple scanners and acquisition protocols. In this paper, ~~to circumvent the complex MRI harmonization, we present HeteroMRI , an approach for analyzing multi-protocol and~~ HeteroMRI is utilized to detect brains with WM abnormalities in FLAIR images through binary classification. HeteroMRI is applicable to multi-scanner ~~MRI data. The method utilizes~~ and multi-protocol datasets and demonstrates effectiveness in data-limited conditions, providing a flexible and practical solution for both research and clinical applications. The presented method employs MRI intensity clustering, a technique used in the literature for other MRI-related purposes such as brain tissue segmentation [58, 59], brain tumor segmentation [60] and inhomogeneity correction [61]. ~~Herein, the presented approach is evaluated for detecting the brains with WM abnormalities through binary classification. This innovative approach enables the analysis of datasets that include MRI data from various scanners and acquisition protocols, providing a flexible and practical solution for research and clinical applications. The method is evaluated~~ HeteroMRI is evaluated in various experimental settings to ensure its robustness. Additionally, we apply the method to limited data scenarios in order to assess the performance and applicability of the presented method for rare diseases. In future work, the method is intended to be applied to the task of classifying two WM diseases based on their distinct WM abnormality patterns. The presented method opens new avenues for ~~analyzing~~ performing WM abnormality-related analyses on heterogeneous MRI datasets and the large amount of MRI data generated daily in medical centers.

The current paper is structured in the following way: Sec. 2 provides an overview of the methodology used, detailing the data preprocessing steps and the architecture of the DL model employed in this study. Moving on to Sec. 3, the experiments, ~~explaining the used datasets ,~~ the datasets used, the experimental settings, ~~and~~ the execution of the model, and the evaluation metrics are presented. Following that, ~~in~~

~~Secs~~Sec. 4 ~~,~~ presents the key results~~of this study are presented and discussed~~, while Sec. 5 discusses the results, highlights the limitations of the method, and introduces the possible future directions. Finally, Sec. 6 ~~summarizes the main contributions of our research and future work~~provides the conclusion.

2 Methodology

The WM abnormality detection approach presented in this paper uses heterogeneous brain MRI data with various acquisition protocols (multi-protocol) as the input data for a Convolutional Neural Network (CNN). The model is a binary classifier trained to detect patients with WM abnormalities in their brain MRI. The method consists of three main modules explained in the following subsections, namely MRI preprocessing, Intensity clustering, and DL model. An overview of the methodology is illustrated in Fig. 1.

2.1 MRI preprocessing

For preparing the image data for the analysis, we use our brain MRI preprocessing pipeline, *FlexiMRIprep*[1], that consecutively performs all the requested preprocessing steps/algorithms on all the selected images automatically. Being the optimal MRI sequence in detecting WM abnormalities, only FLAIR images are used in the analysis in this paper. All selected images have a minimum of 128~~and~~ , 192, and 22 voxels in their first~~and second~~, second, and third dimensions, respectively. All the MRIs are converted to NIfTI-1 format using the `dcm2niix` tool (version 1.0.20211006) [65] at this point. The preprocessing steps ~~are described below~~ described below are applied identically to all MRIs from different datasets. Detailed information on the parameters used in each step is reported in the GitHub repository of HeteroMRI[2].

- **Bias field correction**: For correcting the bias field or inhomogeneity issues in the MRIs, we employ the commonly used N4ITK [34] bias field correction method for this purpose. For implementation, the `N4BiasFieldCorrectionImageFilter` class from the `SimpleITK` [66] (version 2.1.1.2) *Python* library with the default parameters is used.
- **Registration**: Registration enables precise spatial mapping and the comparison of anatomical structures among the MRIs. In this process, all the MRIs used for training and testing the AI model are aligned to a standard brain template. Among the available brain templates, we choose the "ICBM 2009c Nonlinear Asymmetric" template [62, 63] (referred to below as the MNI template)~~since we need a highly accurate template that also provides the WM probability map which is required in our analysis approach. This template is~~ , which the developers created using the data from the ~~ICBM (~~International Consortium for Brain Mapping (ICBM) project [67]. This template was selected due to its high accuracy and the availability of the WM probability map required for our analysis approach. Since there is no dedicated FLAIR template in the MNI template, the T2-weighted template was used due to its proximity to FLAIR. For the registration, the `antsRegistration` tool from the Advanced Normalization Tools (ANTs) [68] (version 2.4.4.post12-g8cc4f8a) is employed. A nonlinear registration is applied three times (with identical parameters) on each image consecutively. This repeated registration aims to achieve ~~the most precise~~ a high level of alignment

[1] https://github.com/ul-mds/FlexiMRIprep
[2] https://github.com/ul-mds/HeteroMRI

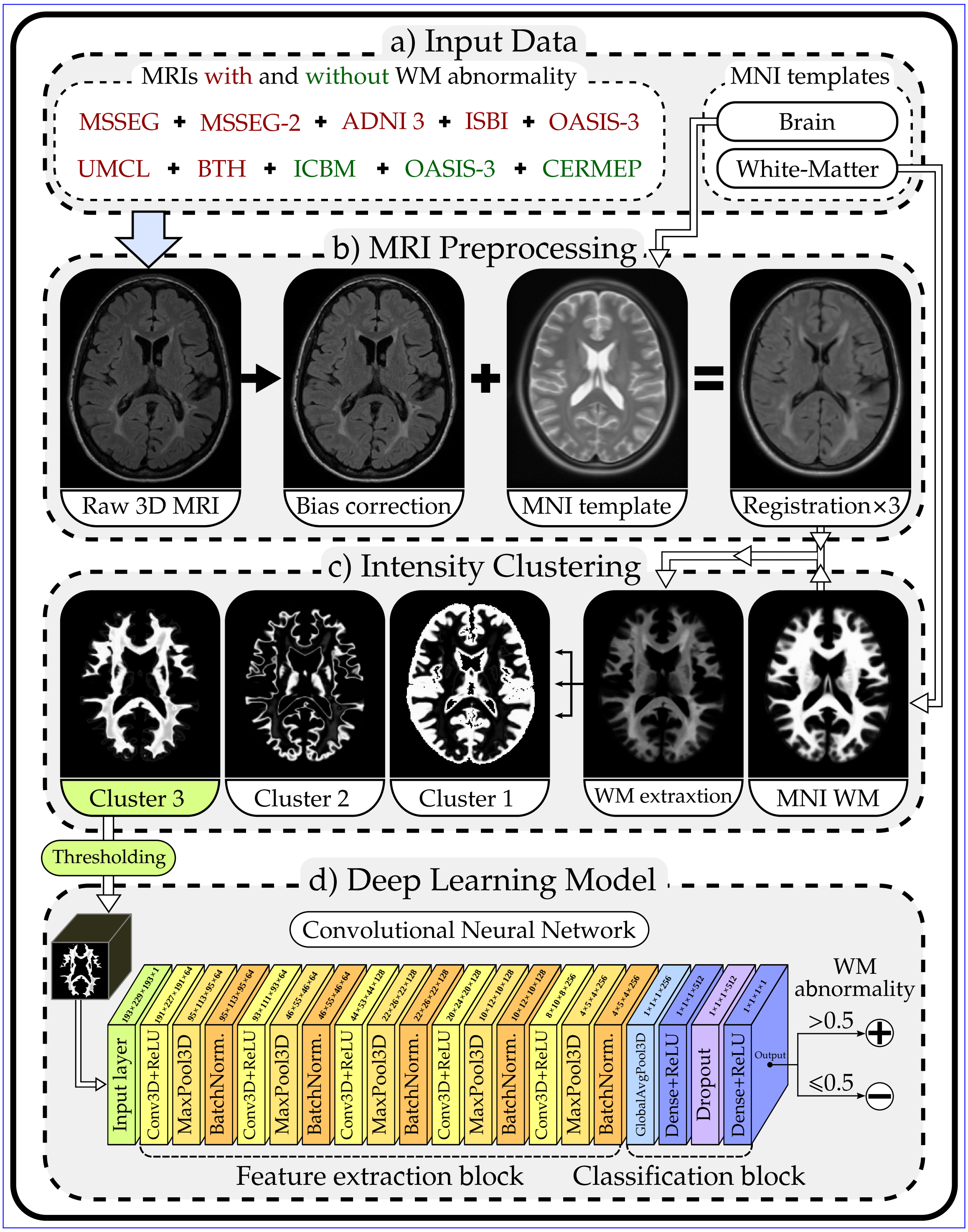

Figure 1: Overview of the methodology: a) Input data(top): The MRI datasets used for the classification model and MNI brain template [62, 63]. The MRI data with and without WM abnormality are taken from the datasets shown in red and green, respectively. b) MRI preprocessing(upper middle): The N4 bias field correction method [34] is applied on the FLAIR MRIs (in 3D) and then the MRIs are three times registered (nonlinearly) to the MNI template. c) Intensity clustering(lower middle): The WM of the brain is extracted and the WM is clustered into three intensity clusters using RFCM [64] algorithm. d) DL model(bottom): Only cluster Cluster 3 of the WM is thresholded and used for a binary classification model with the CNN architecture shown.

of the MRIs with the template. The selection of three iterations was determined experimentally to maintain high alignment accuracy while avoiding the higher computational cost of additional nonlinear registrations. Multi-pass registration has also been used by others, for example, to address large differences in the initial positions of image pairs [?]. The registered MRIs all have a size of 193 × 229 × 193 voxels with a voxel size of 1 × 1 × 1 mm.

2.2 Intensity clustering

- **WM extraction**: After ~~having~~ the brain images are aligned with the MNI template, the WM probability map of the template is used to extract the WM volumes of each brain. Therefore, all the other brain tissues are removed. The WM extraction is performed by using the `MultiplyImages` tool from ANTs.
- **WM clustering**: The WM clustering is performed for two essential purposes: 1) ~~Calculating and obtaining~~ to obtain a sub-group of WM volumes that includes significant signs of WM abnormalities, and 2) ~~Minimizing~~ to minimize the negative effect of the heterogeneous MRI data coming from multiple scanners and protocols. These two points are elaborated on in the following.

A clustering algorithm is used to estimate ~~a certain number of~~ three intensity clusters from the WM volumes obtained in the previous step. The algorithm groups the WM volumes into three subgroups that share a relatively similar intensity range. We expect that one of the clusters shows more indications of WM abnormalities (if present in the brain) since the abnormalities have higher intensity values in FLAIR images. ~~Such a~~ This specific cluster will be used as the training data for the classifier model. The cluster is a membership function with float values in the range of [0,1] for each voxel. As a result, the impact of the heterogeneous nature of the multi-protocol MRIs is minimized. The decision to use three clusters was based on our empirical observations from a dataset different from those used in this study. Through testing various cluster numbers on different MRIs, we found that three clusters yielded consistently comparable patterns in nearly all MRIs. In other words, the shapes of the clusters in one MRI were generally consistent with those in another MRI. This was also later observed in the data of the current study. Therefore, the choice of three clusters is robust and does not depend on a specific dataset.

Here, we employ a Robust Fuzzy C-Means (RFCM) algorithm [64] for WM intensity clustering. The RFCM algorithm modifies the standard FCM objective function by incorporating a local spatial penalty term, leading to the computation of smoother membership functions. This modification not only improves segmentation performance but also provides a level of noise insensitivity. The RFCM algorithm is implemented using the `fuzzy_cmeans` function available in the *Nighres* (Neuroimaging at high resolution) *Python* package, version 1.4.0 [69].~~The choice of using three clusters is based on our empirical observations. We tested various cluster numbers on different MRIs and found that three clusters yielded consistently similar patterns across almost all MRIs. In other words, the three clusters in one MRI relatively match the shapes of the three clusters in another MRI.~~

Upon examining the three WM intensity clusters in MRIs with WM abnormalities, we noted that one of the clusters within each MRI, Cluster 3 in Fig. 1(c), consistently exhibited significant lesion-related features. In the MRIdata that we analyzed, usually the third cluster and sometimes (in less than 10% of the cases) Therefore, from each MRI, we should take the cluster that looks visually similar to Cluster 3 in Fig. 1(c) but it is not always the cluster number 3. For this purpose, we use the Dice similarity coefficient[?] to compare the three clusters of each MRI with a fixed reference cluster to detect the most similar one. The reference image (available in the second cluster was the one including the abnormalities. For this study , we manuallyidentified the intended cluster for each MRIby choosing the cluster that looks visually similar to Cluster 3 in Fig. 1. However, the intended cluster could also be automatically detected through the training of a DL modelGitHub repository of HeteroMRI) is generated by averaging the intended intensity cluster of four MRIs from a clinical dataset. This method detected the right cluster for all the MRIs of this study correctly (i.e. with 100% accuracy) as checked manually.

- **Thresholding**: A thresholding is applied on the selected WM intensity cluster of each MRI. All the intensity values below 0.5 are ignored in order to remove uncertain, low-confidence assignments and retain only the core voxels that are strongly associated with the cluster. The value 0.5 is chosen experimentally in the design phase of HeteroMRI by using (clinical) datasets different from those used in this study for the task of classifying two WM diseases. The value 0.5 resulted in the highest improvement in the classification accuracy compared to other tested thresholds. The histogram for most MRIs follows the same overall pattern: approximately $50 \pm 5\%$ of intensity values are below 0.2, and around $35 \pm 5\%$ are above 0.8. Supplementary Fig. S4 shows the WM cluster of a sample MRI before and after thresholding along with their normalized histograms (for the 99% upper percentile). Finally, all the selected clusters from all the thresholded clusters from the MRIs (one intensity cluster per MRI) serve as the are used as training data for the DL model, as described in the following section.

2.3 Deep learning model

The objective is to train a binary classifier model that detects the brain MRIs that have WM abnormalities. Inspired by [70, 71], we configured a 3D CNN comprising a total of 20 layers, as illustrated in Fig. 1(at the bottomd). The network has a total of 1,795,905 parameters. The model begins with the input layer, followed by a feature extraction block, and ends with a classification block.

In the feature extraction block, we employ five 3D Convolution (Conv3D) layers with 64, 64, 128, 128, and 256 filters, respectively. Each Conv3D has a 3x3x3 kernel size and employs the Rectified Linear Unit (ReLU) activation function. Subsequently, each Conv3D layer is succeeded by a 3D Max Pooling (MaxPool3D) layer with a stride of (2,2,2) and a pool size of (2,2,2) which downscales the 3D input by half in each dimension. Batch Normalization [72] layers with default parameters follow each MaxPool3D layer.

In the classification block, a 3D Global Average Pooling (GlobalAvgPool3D) layer is followed by a Dense layer with a dimensionality of 512 and with a ReLU activation function. To help prevent overfitting, a Dropout layer with a 30% rate is introduced next. Finally, the output layer performs a binary classification employing a Sigmoid activation function. The binary cross-entropy loss, Adam optimizer [73], and an *Early Stopping* feature (*patience*=40) are employed in the model. In each epoch, the checkpoint feature saves the model if the validation accuracy has improved. In the case of an unchanged validation accuracy, the mode is saved if the validation loss has decreased. The *Python* implementation code of the HeteroMRI method is publicly available[3].

3 Experiments

Different MRI datasets are used along with multiple experimental settings with various conditions to train and evaluate the CNN model for classifying brain MRIs. In the following subsections, the datasets and the experimental settings are elaborated.

3.1 Datasets

In this study, we utilized FLAIR images from multiple brain MRI datasets as introduced below. Incorporating a combination of MRIs with a high diversity of acquisition protocols and scanners ensures a robust evaluation of the presented methodology. All the datasets used in this study are either publicly available or are accessible upon request to the respective dataset providers. As presented in Table 1, a total of nine MRI datasets are utilized.

Table 1: List of MRI datasets used in this study

Dataset name/alias	Images[1]		Protocols[2]	Availability	Reference
	+	−			
ISBI	19	0	1	Public	[74]
UMCL	30	0	1	Public	[75]
MSSEG	52	0	4	AoR[3]	[76]
MSSEG-2	40	0	10	AoR[3]	[77]
BTH	9	0	2	Public	[78]
ICBM	0	5	1	AoR[3]	[79]
OASIS-3	14	90	4	AoR[3]	[80]
ADNI 3	58	0	8	AoR[3]	[81]
CERMEP	0	27	1	AoR[3]	[82]
Sum	222	122	32	-	-

[1] Number of FLAIR images, with (+) and without (−) WM abnormality
[2] Number of MRI protocols in the used data
[3] Accessible on Request (to the respective dataset provider)

The details of each dataset are outlined below:

- **ISBI**: The International Symposium on Biomedical Imaging (ISBI) in 2015 [74] conducted an MS lesion segmentation challenge using longitudinal MRI data. The dataset comprises imaging data

[3]https://github.com/ul-mds/HeteroMRI

from MS patients, acquired using the same scanner and protocol. We utilize 19 FLAIR images from this dataset. For each patient, the MRI taken at the latest time point is used.

- **UMCL**: A cohort of MS patients was imaged at the University Medical Center Ljubljana (UMCL) [75]. The images were acquired using the same scanner and protocol. We use 30 3D FLAIR images from this dataset.
- **MSSEG**: The MSSEG dataset [76] was presented for the MS lesion segmentation challenge during the MICCAI 2016 conference. The dataset contains MRIs of MS patients from four different sites. Each site used different MRI scanners and protocols. We utilize 52 FLAIR images from this dataset.
- **MSSEG-2**[4]: MSSEG-2 [77] is a challenge for the segmentation of new MS lesions in the brain conducted in the MICCAI 2021 conference. At the time of the current research, only the training data of the dataset is accessible. The images of the training set were acquired at 12 different sites and using 10 different scanners. All the images were acquired at two different time points from each patient. From this dataset, we utilize 40 3D FLAIR images from the second time point.
- **BTH**: The brain MRI dataset of MS patients from Baghdad Teaching Hospital (BTH) [78] includes MRIs taken at 20 centers with different protocols. We used nine[5] FLAIR images from this dataset, which were taken using two different protocols.
- **ICBM**[6]: The International Consortium for Brain Mapping (ICBM) [79] has developed a probabilistic atlas and reference system for the human brain for normal adults. The dataset includes 20 3D FLAIR images; however, only five were selected for this study, as our neurology specialist confirmed these to be the only ones free of ~~white matter~~ WM abnormalities. The images were acquired using the same scanner and protocol.
- **OASIS-3**: The Open Access Series of Imaging Studies (OASIS) is a project with the goal of providing open access to neuroimaging datasets of the brain. Among the OASIS datasets, FLAIR images are exclusively available in OASIS-3 [80]. OASIS-3 encompasses both cognitively normal adults and individuals at different stages of cognitive decline. Our model utilizes a total of 104 FLAIR and T2-FLAIR images from this dataset, acquired using four different protocols. Out of these images, 90 were meticulously selected by two neurology specialists from a pool of 600, confirming the absence of ~~white matter~~ WM abnormalities in the brain. The other 14 images included different patterns of WM abnormalities.
- **ADNI 3**: The Alzheimer's Disease Neuroimaging Initiative (ADNI) database[7] provides an extensive

[4]Data were generated by participating neurologists in the framework of Observatoire Français de la Sclérose en Plaques (OFSEP), the French MS registry [83]. They collect clinical data prospectively in the European Database for MS (EDMUS) software [84]. MRI of patients were provided as part of a care protocol. Nominative data are deleted from MRI before transfer and storage on the Shanoir platform (Sharing NeurOImagingResources, shanoir.org).

[5]The NIfTI files in this dataset lack orientation information (qform and sform), making it impossible for the registration algorithm to identify the correct orientation of the brain. Additionally, the MRIs are 2D, resulting in around 10 times fewer ~~layers~~ slices than the pixels in the first and second dimensions, and they all share the same pixel thickness across all three dimensions. Consequently, the brain appears unrealistically short in 3D view. Due to these dataset-specific conditions, we applied two additional preprocessing steps at the beginning for this dataset: 1) added correct orientation information to each file, and 2) edited ~~layer~~ slice thicknesses in the header of NIfTI files based on ~~layer~~ slice thickness information provided in the dataset's metadata. However, the height of many images still does not appear realistic and may cause problems for the registration. Therefore only nine images were used

[6]The ICBM project (Principal Investigator John Mazziotta, M.D., University of California, Los Angeles) is supported by the National Institute of Biomedical Imaging and BioEngineering. ICBM is the result of efforts of co-investigators from UCLA, Montreal Neurologic Institute, University of Texas at San Antonio, and the Institute of Medicine, Juelich/Heinrich Heine University - Germany.

[7]The ADNI was launched in 2003 as a public-private partnership, led by Principal Investigator Michael W.

collection of neuroimaging and clinical data [81]. In our model, we included 58 3D FLAIR images from ~~this~~ the ADNI 3 dataset, all of which contained WM abnormalities. These images were carefully selected to encompass various patterns of lesions, including multifocal and confluent lesions, as well as those located in the brain stem, as confirmed by a neurology specialist.

- **CERMEP**: The CERMEP-IDB-MRXFDG dataset [82] comprises MRI, CT, and [^{18}F]FDG PET image data with BIDS standard of healthy subjects. The dataset has 37 FLAIR images obtained using the same scanner and protocol. As reported in the original study, these images underwent visual review by two neurologists to confirm the absence of any apparent brain abnormalities. However, due to our strict criteria for even minor lesions, our neurologists confirmed only 27 MRIs as free of WM abnormalities for use as control data in our model.

From the MRI datasets explained above, a total of 344 images are used in this study for training and testing the model. Around 35% of these MRIs are 2D, based on our definition that MRIs with 70 or fewer slices are considered 2D. A comprehensive list of the MRI files is available in the GitHub repository of HeteroMRI providing details for each image, including the subject ID from the original dataset and the acquisition protocol.

3.2 Experimental settings

Various experimental settings ~~,~~ have been designed for a robust evaluation of the presented classification approach. An experimental setting means the specification of the data used for training, validating, and testing the CNN model. By employing the datasets explained in Sec. 3.1, ~~MRIs with different~~ FLAIR images from different scanners and acquisition protocols are intentionally combined and used for training and testing the model. The images necessary for each setting are selected randomly from the MRIs available. The number of MRIs with and without WM abnormality is balanced in the training, validation, and test data of all the settings. ~~The same holds for the test data. In total, there are four groups of settings~~There are four setting groups, namely A, B, C, and D. In setting A, the data are selected based on datasets while in the settings B, C, and D, the data are incorporated based on their acquisition protocol. We assigned a protocol name to each of the MRIs based on the scanner name and model, magnetic field strength, and acquisition parameters. The protocol naming convention is explained in the HeteroMRI Github repository. The experimental settings are introduced below:

- **Setting *A***: In setting A, the goal is to evaluate HeteroMRI on a combination of MRIs from different datasets beginning from a relatively large number of data and then decreasing the data gradually. In setting A, there are 19 settings that are run independently. In $A00$, 244 MRIs from nine datasets are used. The data of ~~each~~ *each* dataset is split into training (70%), validation (10%), and test (20%) sets. In $A01$, the training data is downsized by approximately 10% while the test set remains the same images as in $A00$. The downsizing process continues up to $A18$, where the training and validation sets together include only four MRIs. The downsizing is performed by removing random MRIs while keeping the maximum possible number of protocols among the data. Across all settings

Weiner, MD. The primary goal of ADNI (`https://adni.loni.usc.edu`) has been to test whether serial MRI, positron emission tomography (PET), other biological markers, and clinical and neuropsychological assessment can be combined to measure the progression of Mild Cognitive Impairment (MCI) and early Alzheimer's Disease (AD).

from $A00$ to $A18$, the test set remains identical. Supplementary Table S1 shows the number of MRIs used for training, validation, and test sets from each dataset in the settings $A00$ to $A18$.

- **Setting B**: In setting B, the goal is to choose the MRIs with the most diversity of protocols while having an equal number of ~~MRI~~ MRIs from each protocol ~~. Therefore, an equal~~ therefore the model sees the same number of MRIs ~~is incorporated~~ per protocol. MRIs from 10 different protocols are incorporated. The test data is selected from all protocols. In $B00$, from each protocol, five MRIs for training, one MRI for validation, and one MRI for the test are used. In the next subsequent settings, the training data is reduced. By $B04$ only one MRI per protocol is used for the training set. In Supplementary Table S2, the list of selected protocols and the number of MRIs used for training, validation, and test sets for $B00$ to $B04$ is reported.

- **Setting C**: In setting C, the goal is to assess the generalizability of HeteroMRI to unseen MRI protocols. In this setting, MRIs from eight different protocols are included. Only data from 3D MRIs are included in this setting due to the reason later explained in Sec. 4.3. MRIs from six protocols are used *only* in training and validation sets while the other two protocols are *only* used in the test set. In fact, the trained model does not see any data from the protocols used in the test set during training. We consider 10 cases, in each case considering two different protocols for testing the model. The setting C begins with $C00$ which uses 64 MRIs for training and validation. This continues up to $C06$ with only eight MRIs for training and validation. In Table S3, the list of selected protocols and the number of MRIs used for training, validation, and test sets for $C00$ to $C06$ is reported.

- **Setting D**: In setting D, the goal is to see the effect of the number of MRI protocols on the performance of the model. Beginning from $D00$ and going toward $D03$, more protocols are included in the data used for training and testing the model. At the same time, the total number of data and test set size are kept the same among the settings $D00$ to $D03$ (82 MRIs for training including validation data), therefore it is possible to compare the results of the settings to see the effect of having more protocols in the data. In $D00$, there are MRIs from four protocols. In $D01$ to $D03$, there are MRIs from, respectively, six, eight, and 10 protocols. The test data is selected from all protocols. In Table S4, the list of selected protocols and the number of MRIs used for training, validation, and test sets for $D00$ to $D03$ is reported.

3.3 Model execution

All the MRIs used in this study, introduced in Sec. 3.1, are preprocessed following the procedure elaborated in Sec. 2.1. Next, the intensity clustering procedure is applied to each preprocessed MRI, following the procedure introduced in Sec. 2.2. As a result, a single intensity cluster per MRI is used for training or testing the model. Notably, the 3D intensity clusters obtained from the MRIs serve as the exclusive training data for the model. The model has no exposure to the original MRIs or any form of WM lesion annotation file. For the preprocessing and intensity clustering tasks, we used a machine with Intel(R) Xeon(R) Gold 6240R CPU @ 2.40GHz and 128 GB of RAM. The preprocessing pipeline (introduced in Sec. 2.1) employs a parallelization approach in some of the preprocessing steps to make the procedure faster. The computation time required for preprocessing each MRI depends on multiple factors; nevertheless, the total number of voxels in the 3D MRI plays a more significant role. More specifically, based on

our assessments, the number of ~~layers~~ slices of the MR image highly affects the required computation time. The Supplementary Fig. S1, shows the average time required for preprocessing and intensity clustering of five sample MRI dimensions.

The CNN model explained in Sec.2.3 is trained and tested on each experimental setting independently. In settings A, B, and D, the required number of data is selected (and split into training, validation, and test sets) from all available data 20 independent times (referred to below as "data shuffle"). For each data shuffle, the model is trained and tested 10 times. In setting C, 5 data shuffles are selected, and for each shuffle, the model is trained and tested 10 times. For training and testing the CNN model, we used a computational server with AMD Epyc 7352 CPU, 1 TB of CPU RAM, and NVIDIA A100 GPU (40 GB GPU RAM). The average required time for training the model of each setting is shown in Supplementary Fig. S2. The inference time of the model on a single test data is a few seconds. The inference process can be efficiently performed without the necessity of a GPU.

3.4 Evaluation metrics

To assess the performance of the classification model, we employ five common metrics: accuracy, sensitivity, specificity, F1 score, and precision. Moreover, the area under the receiver operating characteristic curve (AUROC) is reported for selected settings. Additionally, we calculate a cumulative metric called Machine Learning cumulative performance score (*MLcps*) [85]. The *MLcps* combines the pre-computed performance metrics into a single metric that encapsulates the core aspects of all the metrics. The value of *MLcps* is equal to the area of the polygon created by the metrics in a radar plot. We used the *MLcps* *Python* package version 0.0.6. The *MLcps* metric was originally designed for comparing and identifying the best-performing ML algorithm. However, herein, we utilize *MLcps* to compare the performance of the same model for the different amounts of training data. As we have a fixed number of pre-calculated metrics (accuracy, sensitivity, specificity, F1 score, and precision), we define *MLcps%* as

$$MLcps\% = \frac{MLcps}{MLcps_{max}} \times 100, \tag{1}$$

where $MLcps$ is the area of the pentagon in the radar plot and $MLcps_{max}$ is the area of the pentagon when all the five metrics are 100%.

4 Results~~and discussion~~

In this section, we present and analyze the results of the experiments to evaluate the performance of the ~~classification model~~HeteroMRI method. The results are reported separately for the experimental setting A, B, C, and D. Furthermore, the insights on limited data scenarios gained through the experiments are discussed afterward. The classification metrics are provided in box plot and radar plot ~~format~~ formats in Figs. 2, 5, 6, and 7. In all box plots, the triangle marker indicates the mean value, and the whiskers represent $1.5 \times IQR$, where IQR is the interquartile range. In settings A, B, and D, the box plots show the distribution of 200 values for each setting # corresponding to 20 data shuffles that each has been run

10 times. Setting By "setting #means ", we mean for example $A00, A01, \ldots, A18$. The higher the setting #, the less training data is used. In setting C, the box plots show the distribution of 10 values for each setting #. Each of these 10 values is the average of 50 values (five shuffles, 10 runs each) corresponding to the 10 cases with different protocols chosen as the test set, as explained in Sec. 3.2.

The radar plots illustrate the metric values for all the setting #s simultaneously, allowing us to perceive the effect of reducing training data size on each metric. In addition, the radar plots are utilized for calculating the Machine Learning Cumulative Performance Score ($MLcps$) [85]. $MLcps$ is a *Python* package for classification problems that combines various pre-computed performance metrics into a single metric that encapsulates the core aspects of all the metrics. The value of $MLcps$ is equal to the area of the polygon created by the metrics in the radar plot. We used the $MLcps$ $MLcps$ values using Eq. (1). The *Python*$MLcps\%$ package version 0.0.6. The $MLcps$ metric was originally designed for comparing and identifying the best-performing ML algorithm. However, herein, we utilize $MLcps$ to compare the performance of the same model for the different amounts of training data. As we have a fixed number of pre-calculated metrics (accuracy, sensitivity, specificity, F1 score, and precision), we define $MLcps\%$ as

$$MLcps\% = \frac{MLcps}{MLcps_{max}} \times 100,$$

where $MLcps$ is the area of the pentagon in the radar plot of each setting and $MLcps_{max}$ is the area of the pentagon when all the five metrics are 100%. The $MLcps\%$ values for the settings A, B, and C are reported in the Figs. 2, 5, and 6 in part (e).

Evaluation of the presented approach across various experimental settings provides several key insights, offering a detailed understanding of its performance and challenges. The results of each experimental setting are presented below.

4.1 Setting A

As shown in Fig. 2, setting $A00$, in which the highest number of MRIs (174 training+26 validation+44 test) were included, demonstrates an average accuracy of 96% in the classification of MRIs. The training and testing data in this setting includesin total , in total, 32 different MRI protocols. This underscores the model's adaptability and robustness in handling a diverse range of imaging protocols. Notably, the effect of reducing the training data on the model's performance is inspected here. In $A07$, where the training data (including validation data) is reduced to 36% of $A00$, the accuracy remains above 92%. Beginning from setting $A08$, where the training data is 25% of $A00$, the average accuracy and sensitivity have a sharp decrease. With much further decreasing the training data (e.g. $A18$ with only two MRIs as training) the accuracy and sensitivity gradually decrease to low values as expected while specificity tends to remain relatively high. In relatively limited data settings (particularly from $A11$ to $A18$), the boxplots indicate that accuracy values have significant fluctuations across the range. This shows that, with additional repetitions of such settings using different data shuffles, the average accuracy would likely converge toward the chance level (50%) due to insufficient training data for the CNN to effectively learn. However, investigating this

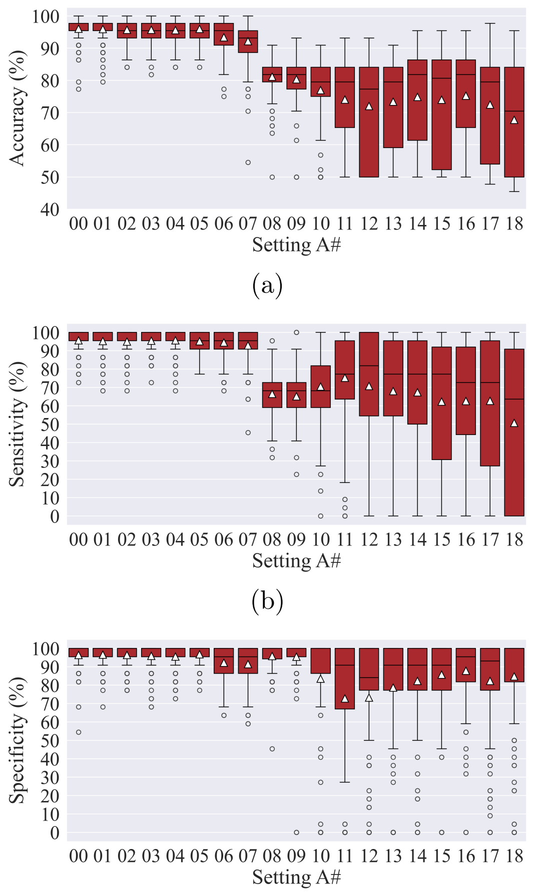

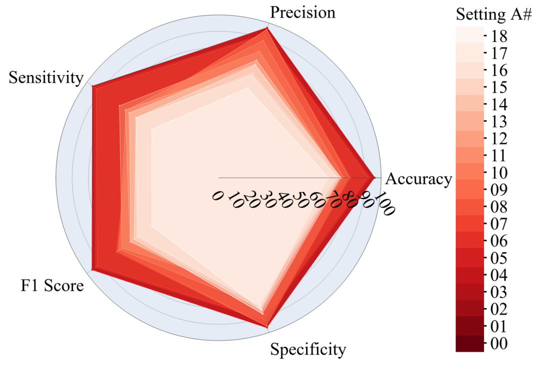

Setting	$A00$	$A01$	$A02$	$A03$	$A04$
MLcps%	91	91	91	90	90
**Setting**	$A05$	$A06$	$A07$	$A08$	$A09$
MLcps%	91	85	83	64	63
**Setting**	$A10$	$A11$	$A12$	$A13$	$A14$
MLcps%	56	51	45	47	49
**Setting**	$A15$	$A16$	$A17$	$A18$	
MLcps%	45	48	43	34	

(e)

Figure 2: Classification results of settings $A00$ to $A18$: (a) accuracy, (b) sensitivity, (c) specificity, (d) radar plot of five classification metrics for different setting #s, and (e) ~~*MLcps%*~~ *MLcps%* (a cumulative performance score) in % for each setting #. The triangle marker indicates the mean value and the whiskers represent $1.5 \times IQR$. In each setting # (i.e. $A00$, $A01$, …, $A18$) the training set size is sequentially reduced by approximately 10% relative to the previous setting, as detailed in Supplementary Table S 1. Average accuracy starts at 96% for $A00$, where training includes 200 MRIs, and remains above 92% until $A07$, with 72 training data, after which it drops with further reductions in training data. A similar trend is observed in the ~~*MLcps%*~~*MLcps%*.

phenomenon is beyond the scope of this study.

~~Sample MRIs and the WM intensity cluster obtained from each of them~~

We conducted a permutation test to assess the statistical significance of the model's performance. By randomly shuffling the class labels 7,000 times and retraining the model (for setting $A00$) on each shuffled dataset, a distribution of accuracy values under chance conditions was obtained. All of the 7,000 permuted accuracy values were smaller than the model's original accuracy for setting $A00$. This results in a p-value of 0.0001, showing a statistically significant and meaningful distinction between the two classes, confirming that the model captures real patterns in the data. Supplementary Fig. S3 shows the normalized histogram of the permutations compared to the model's original accuracy.

Sample raw MRIs, the registered MRIs, the obtained WM intensity clusters, and the model's label predictions are provided here for a better insight into the data used for testing the model~~and the obtained result~~. Figs. 3 and 4 depict samples of MRIs without and with WM abnormality, respectively. Below each

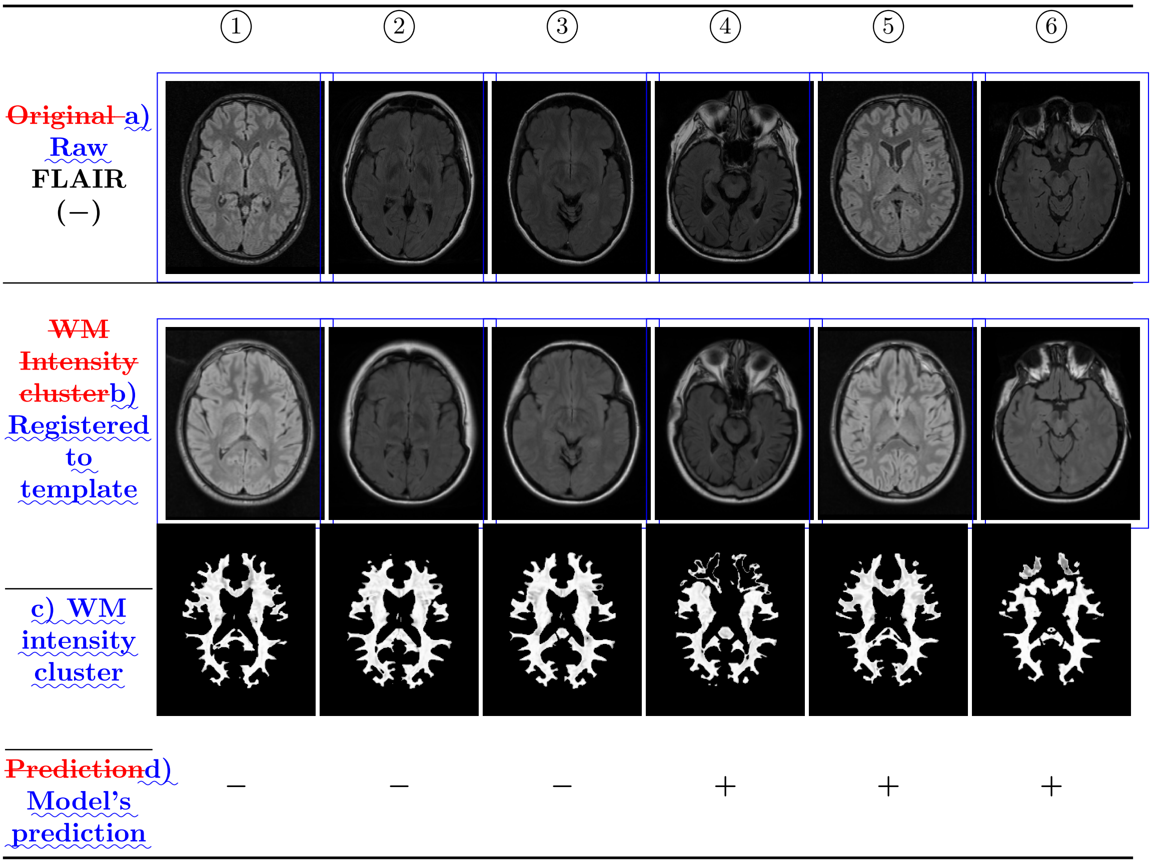

Figure 3: Example of six MRIs (① to ⑥) *without* WM abnormality. ~~Top: original~~ a) Raw FLAIR images, ~~Middle:~~ b) the MRI three times registered to the MNI template (the middle slice shown), c) the obtained intensity clusters (thresholded) used for testing the model, and ~~Bottom:~~ d) the prediction of the model in setting $A00$ for the presence of WM abnormalities (+: with WM abnormality, −: without WM abnormality). This figure shows three cases of true negative and three cases of false positive. The WM intensity cluster slice ~~does~~ corresponds to the registered MRI slice but not exactly ~~correspond~~ to the ~~original~~ raw MRI slice due to deformations from non-linear ~~registration and only one of the three WM parts being present in this cluster~~registrations. MRIs source: ①, ⑤:[82], ②, ~~④~~ ③ ~~:[80]~~, ~~③~~ ④:[80], ⑥:[79].

~~original~~ raw MRI, the three-times registered MRI, and the obtained WM intensity cluster ~~is~~ (thresholded) are illustrated. Additionally, the model's prediction for the presence of WM abnormalities in setting $A00$ is reported. Each figure includes three true predictions and three false predictions. It is important to note that the shown WM intensity cluster ~~slice does~~ and the registered MRI slice show the same location of the brain (the middle slice). However, these two slices do not directly correspond to the ~~original~~ raw MRI slice. This is because the non-linear registration process ~~during the MRI preprocessing results in significant~~ results in the deformation of the brain~~structure and the MRI's number of layers is changed. In addition, the WM of the brain is clustered into three parts, and the illustrated WM intensity cluster is only one of the three parts.~~. As a result, finding ~~exactly the~~ the exact corresponding slices in the ~~original~~ raw MRI and the ~~WM intensity cluster~~ registered one is impractical. ~~These sample slices of the~~ Here, the middle slice of the registered MRI is illustrated. The raw MRI slices shown here are selected based on their visual similarity to the corresponding registered slices. The slices of the thresholded WM cluster are presented here solely to illustrate the input provided to the DL model for label prediction.

By investigating the possible reasons for the false predictions in setting $A00$, certain aspects became

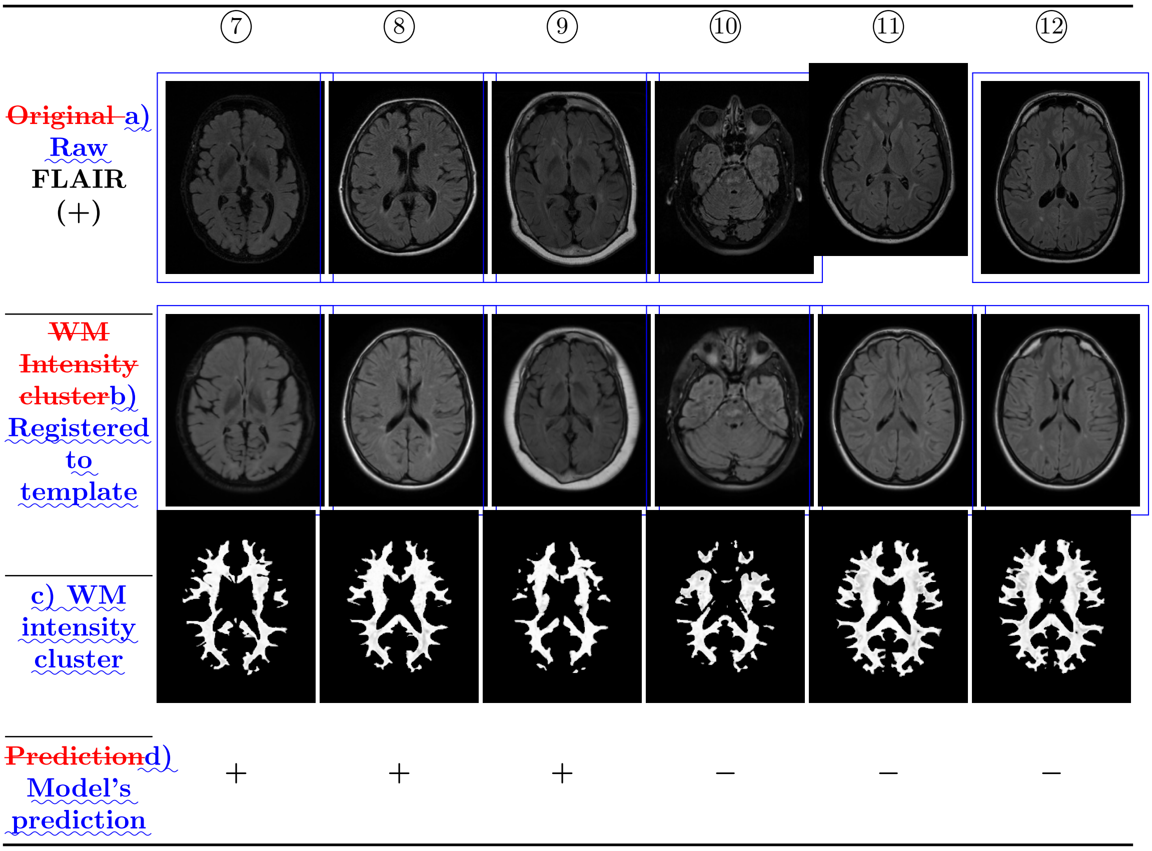

Figure 4: Example of six MRIs (⑦ to ⑫) *with* WM abnormality. ~~Top: original~~ a) raw FLAIR images, ~~Middle:~~ b) the MRI three times registered to the MNI template (the middle slice shown), c) the obtained intensity clusters (thresholded) used for testing the model, and ~~Bottom:~~ d) the prediction of the model in setting $A00$ for the presence of WM abnormalities (+: with WM abnormality, −: without WM abnormality). This figure shows three cases of true positive and three cases of false negative. The WM intensity cluster slice ~~does~~ corresponds to the registered MRI slice but not exactly ~~correspond~~ to the ~~original~~ raw MRI slice due to deformations from non-linear ~~registration and only one of the three WM parts being present in this cluster~~registrations. MRIs source: ⑦,⑩:[81], ⑧:[78], ⑨:[80], ⑪,⑫:[74].

apparent. In some MRIs, the registration process has not been successful in correctly aligning the brain to the brain template. In such cases, the brain regions are not located in the correct locations after the ~~three times of registration (discussed in Sec. 2.1).~~ three-times registration in the MRI preprocessing phase. Therefore, in the WM extraction step, wrong parts of the brain are extracted as WM. This mostly results in a false prediction by the model, especially if the MRI is, in fact, free of WM abnormalities. ~~The examples of wrong~~ Examples of incorrect registration are MRIs ④, ⑤, ⑥, ~~and~~ ⑩, and ⑫ shown in Figs. 3 and 4. These registration errors are identified by comparing the slices of the registered MRIs with the corresponding slice from the MNI template slice shown in Fig. 1(b). In both cases, the middle slice of the MRI is displayed. In an accurate registration, the brain regions in the registered slice align approximately with those in the template. We noticed that ~~the registration problem happened more with~~ some of the MRIs with incorrect registration are 2D MRIs (such as ~~MRI~~ MRIs ④ ~~) although it is not the only reason. Around 35% of MRIs used in this study were 2D. The nonlinear registration algorithm did not succeed in registering some of the 2D MRIs due to their low resolution in the 3rd dimension, i.e. having thick layers.~~

~~Another factor causing a false negative prediction is seen in MRI~~ and ⑫~~. By inspecting the three intensity clusters of this MRI, we noticed that the trace of the WM abnormalities is present in a different intensity cluster than the one used by the model for its prediction. As shown in Fig.4, the WM intensity cluster of MRI ⑫ lacks the regions relevant to WM abnormalities (small dark areas). Consequently, the model has classified this MRI as negative. However, this source of false prediction occurs rarely among the MRIs~~). The low number of slices in 2D MRIs appears to be a contributing factor to registration problems in some cases. Considering that MRI registration is usually a challenging problem, other factors are also likely to contribute to incorrect registrations; however, they have not been investigated in this study. We refrained from excluding the MRIs with erroneous registration from the study, as our goal was to evaluate HeteroMRI as a fully automatic method without user interference.

4.2 Setting *B*

The setting B which uses an equal number of MRIs from each MRI protocol is designed to make the prediction task more challenging for the model. Setting $B00$ has an average accuracy of 88.6%, as shown in Fig. 5. In terms of the amount of training data, setting $A07$ is the closest match to $B00$. While $B00$ includes 50 training and 10 validation data, $A07$ has a comparable setup with 52 training and 20 validation data. However, setting B is a more challenging scenario than setting A because ~~it includes~~ the model sees an equal number of MRIs from each MRI protocol ~~in the training data~~without being biased by higher number of images of some protocols. Despite this challenge, the accuracy of $B00$ is only 3.6% less than that of $A07$. This shows the high ~~independency~~ independence of the presented MRI classification approach on the acquisition protocol of the FLAIR ~~image~~images. With further decrease in the training data, in settings $B01$ to $B04$, all metrics show a gradual decrease in value. We expect the accuracy to likely tend toward the chance level by repeating more data shuffles in very limited data scenarios such as $B03$ and $B04$.

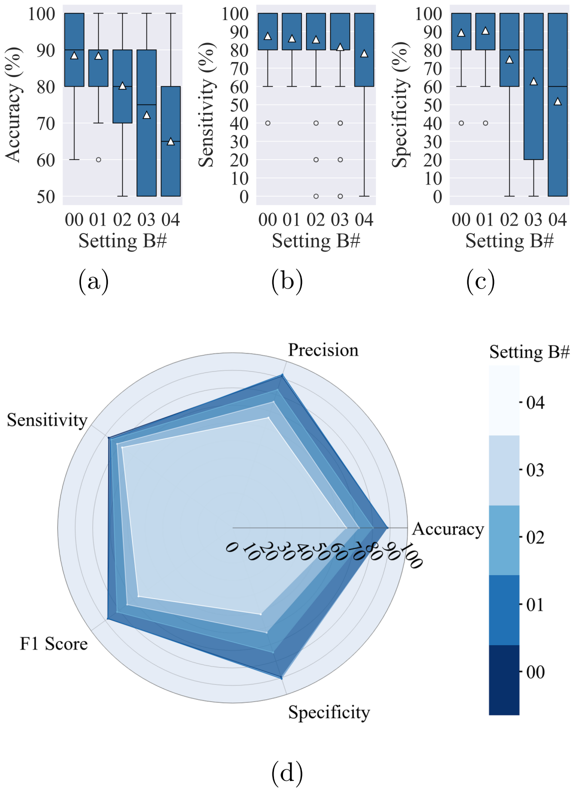

**Setting**	$B00$	$B01$	$B02$	$B03$	$B04$
MLcps%	76	76	61	48	37

(e)

Figure 5: Classification results of settings $B00$ to $B04$: (a) accuracy, (b) sensitivity, (c) specificity, (d) radar plot of five classification metrics for different setting #s, and (e) ~~*MLcps%*~~ *MLcps%* (a cumulative performance score) in % for each setting #. The triangle marker indicates the mean value and the whiskers represent $1.5 \times IQR$. In each setting # (i.e. $B00$, $B01$, ..., $B04$) the training set size is sequentially reduced relative to the previous setting, as detailed in Supplementary Table S 2. In setting B, an equal number from 10 different MRI protocols is used for the model. Average accuracy starts at 88.5% for $B00$, where training includes 60 MRIs, and remains above 80% until $B02$, with 40 training data, after which it decreases gradually with further reductions in training data. A similar trend is observed in the ~~*MLcps%*~~ *MLcps%*.

4.3 Setting C

The evaluation of setting C is of more importance since it is very close to the real-world use of such a model as it evaluates the generalizability of HeteroMRI to MRIs from unseen protocols. In this setting, the MRI protocols of the test set are not present in the training data. It resembles a situation in which a clinical center has heterogeneous MRI data and wants to train a classification model with them. Then the model is supposed to classify new MRIs brought by new patients from other centers, acquired most probably with MRI protocols different from those in the training data.

It is noteworthy that in setting C only 3D MRIs (with 192 or more ~~layers~~slices) are included in training and test data, as discussed in the next paragraph. Setting $C00$, as reported in Fig. 6, shows an average accuracy of 94.9% with 64 MRIs used for training (including validation), ~~despite being tested with MRI protocols that were unseen during training~~which proves the generalizability of the trained model to unseen MRI scanners and protocols. By reducing the data to 46, in $C02$, the model shows an accuracy of 88.1%. By further decreasing the data to 36, the model's performance drops to 64.4% accuracy ~~. In a nutshell, by training the model with only 46 3D MRIs, it is able to classify MRIs acquired with protocols unseen during training with 88.1% accuracy~~in $C03$, which is expected to tend toward 50% with more data shuffles.

Initially, we used ~~the same data as setting *B*~~ both 3D and 2D MRIs for setting C. However, the model showed a lack of robustness when ~~the MRI protocols used~~ its generalizability to different protocols was evaluated (i.e. by varying the protocols considered as test data~~were changed. To investigate possible sources of this issue, we first used *only*~~). As we suspected the 2D MRIs (with 70 or fewer ~~layers) for the model~~slices) as a source of the model's poor performance, we redesigned the setting C to include *only* 3D MRIs. As a result, ~~but it was not robust. Subsequently, we trained and tested the model by excluding 2D MRIsentirely. Remarkably,~~ the performance ~~notably~~ significantly improved, as reported in the results for setting C~~results, proving the~~ . It is important to emphasize that the test data were not fixed for the two versions of setting C, as the model was tested with multiple data shuffles for each setting, with the data being split again in each shuffle. This ensures that this was not a case of refitting on the same test data. Additionally, to verify the adverse effect of 2D MRIs~~.~~ , we designed a separate setting with *only* 2D MRIs. In this case, the model showed very poor robustness.

4.4 Setting D

Setting D, in which the number of protocols was increased in each setting # while maintaining the same data size (82 MRIs for training), shows the slight negative effect of having higher numbers of protocols, as observed by the overall decrease in accuracy, sensitivity, and specificity (Fig.7). In the setting with the most diverse protocols, setting $D03$, the model classifies the MRIs with an accuracy of 89.80%.

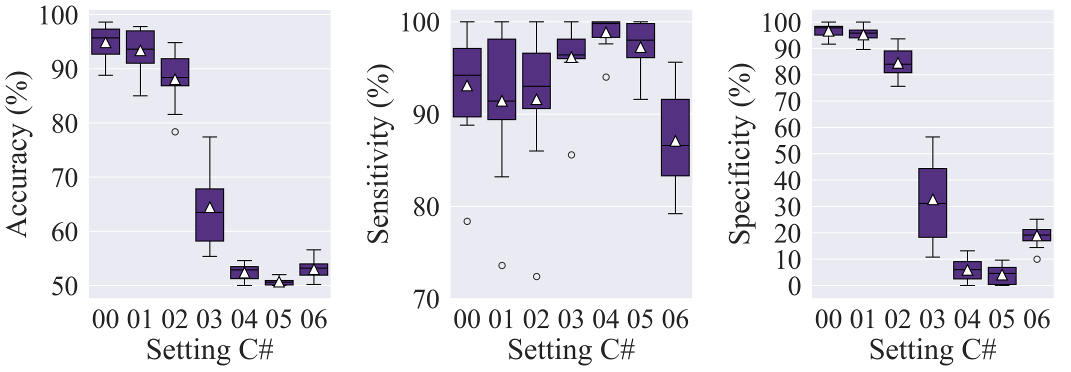

(a) Accuracy (b) Sensitivity (c) Specificity

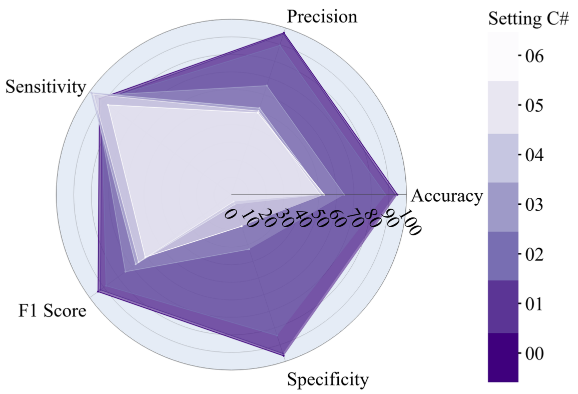

(d) Radar plot

**Setting**	$C00$	$C01$	$C02$	$C03$	$C04$
MLcps%	89	86	75	38	25
**Setting**	$C05$	$C06$			
MLcps%	24	23			

(e) ~~*MLcps%*~~ *MLcps%* values (in %)

Figure 6: Classification results of settings $C00$ to $C06$: (a) accuracy, (b) sensitivity, (c) specificity, (d) radar plot of five classification metrics for different setting #s, and (e) ~~*MLcps%*~~ *MLcps%* (a cumulative performance score) in % for each setting #. The triangle marker indicates the mean value and the whiskers represent $1.5 \times IQR$. In each setting # (i.e. $C00$, $C01$, ..., $C06$) the training set size is sequentially reduced relative to the previous setting, as detailed in Supplementary Table S 3. In setting C, the MRI protocols of the test set are unseen by the model during training. Average accuracy starts at 94.9% for $C00$, where training includes 64 MRIs, and remains above 88% until $C02$, with 46 training data, after which it drops sharply with further reductions in training data. A similar trend is observed in the ~~*MLcps%*~~*MLcps%*.

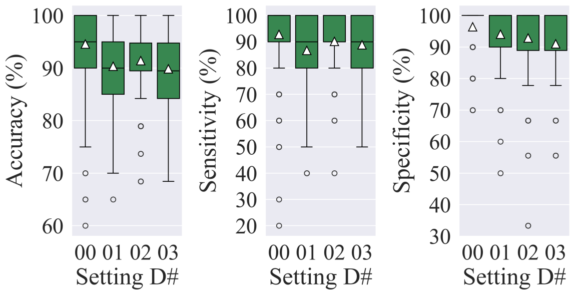

(a) Accuracy (b) Sensitivity (c) Specificity

Figure 7: Classification results of settings $D00$ to $D03$: (a) accuracy, (b) sensitivity, and (c) specificity. The triangle marker indicates the mean value and the whiskers represent $1.5 \times IQR$. In each setting # (i.e. $D00$, $D01$, ..., $D03$) the number of MRI protocols is sequentially increased relative to the previous setting while maintaining equal training set size (82 MRIs), as detailed in Supplementary Table S 4. Average accuracy starts at 94.6% for $D00$, where training data includes four different MRI protocols, and ends at 89.8% for $D03$, with 10 MRI protocols in training data.

4.5 ~~Insights on limited~~ Limited data scenarios

A ~~comparison of~~ more detailed examination of the impact of reducing training data is presented in Table 2. By comparing settings A, B, and C ~~reveals~~ a rough correlation can be concluded between the number of MRIs in the training data and the performance of the model ~~. A more detailed examination of the impact of reducing training data is presented in Table 2. Regardless~~ for the classification task of this study, regardless of the experimental setting~~, when~~ . When the training data (including validation) consists of at least 72 MRIs (as in $A07$), an accuracy of 92% is expected. By having 40 to 46 MRIs in the training set (as in $A09$, $B02$, and $C02$), the accuracy falls within the range of 80% to 88%. Further reducing the training data to the 20 to 36 range (as in $A12$, $B03$, and $C03$) is associated with an accuracy of 72% or less and an F1 score of 74% or less. ~~These findings highlight the method's robustness and its sensitivity to the quantity of training data~~ In this last scenario, the result is not fully reliable as it may tend toward chance level by increasing the number of data shuffles.

Table 2: Average performance results of selected experimental settings (in %). The table shows the effect of reducing training data on the model's performance in settings A, B, and C. For these settings, three cases are reported respectively: 1) with the highest number of training data, 2) with the borderline number of training data after which the performance drops, and 3) with the number of training data that results in relatively low performance. Setting D shows the effect of increasing the number of MRI protocols for the same number of MRI data. AUROC: the area under the receiver operating characteristic curve.

Setting	Data size[1]		Protocols[2]		Accuracy	Sensitivity	Specificity	F1 score	AUROC
	Train[†]	Test	Train[†]	Test					
$A00$	200	44	31	14	96.03	95.68	96.39	95.85	97.69
$A07$	72	44	17±1	14	92.17	92.91	91.43	92.23	96.57
$A09$	42	44	9±1	14	80.33	65.18	95.48	76.40	92.31
$A12$	20	44	6±1	14	72.02	70.82	73.23	67.18	94.03
$B00$	60	10	10	10	88.55	87.60	89.50	87.94	95.64
$B02$	40	10	10	10	80.25	85.70	74.80	81.63	92.37
B~~04~~03	~~20~~ 30	10	10	10	~~65.00~~ 72.25	~~78.10~~ 81.60	~~51.90~~ 62.90	~~66.51~~ 74.48	~~85.02~~90.14
$C00$	64	10	8	2	94.88	93.08	96.68	94.12	99.50
$C02$	46	10	8	2	88.06	91.60	84.52	88.44	98.10
$C03$	36	10	8	2	64.44	96.16	32.72	74.80	93.18
$D00$	82	20	4	4	94.58	92.75	96.40	93.98	99.29
$D03$	82	20	10	10	89.84	88.80	91.00	90.06	94.72

[1] Number of MRIs used in the training and test sets
[2] Number of MRI protocols present in the training and test data
[†] Including the validation data

5 ~~Conclusion~~Discussion

In this study, we introduce HeteroMRI, a novel approach ~~based on intensity clustering for analyzing MRI datasets that include multiple MRI protocols and scanners to address the challenge of analyzing~~

~~heterogeneous MRI data. Specifically~~for robust classification of brain MRIs based on WM abnormalities, specifically for cases where the data consists of heterogeneous MRIs acquired with different scanners and acquisition protocols. In this paper, we apply this approach to train a binary classification model on FLAIR images ~~, designed to detect the brains that contain~~ to detect MRIs containing WM abnormalities. ~~Notably, our method excels without requiring learning-based MRI harmonization efforts. Our extensive evaluation across diverse experimental settingshas shown both the strengths and challenges of the proposed approach.~~

~~The results demonstrate the method 's robustness in handling a broad spectrum of imaging protocols and scanners in FLAIR MRI~~ The results demonstrate that the proposed method shows a robust performance by achieving up to 96% accuracy in detecting MRIs with WM abnormalities across various experimental settings. The method effectively handles the high diversity of MRI scanners and acquisition protocols present in the data. In ~~other words,~~ addition, HeteroMRI mitigates the adverse effect of data heterogeneity through MRI preprocessing steps, primarily the WM intensity clustering step, without relying on additional ML methods.

Notably, the approach performs reliably even in scenarios with limited data, showing the potential of HeteroMRI for application in the classification of MRIs in WM-related rare diseases. For the binary classification task of detecting MRIs with WM abnormalities, providing 72 and 42 MRIs results in a 92% and 80% accuracy, respectively. Moreover, HeteroMRI shows generalizability to unseen MRI scanners and protocols which makes the method stand out compared to many other ML/DL methods that fail in this aspect. Furthermore, by not relying on manual lesion annotations, HeteroMRI reduces the demand for expert human resources, facilitating its usability in practical settings compared to other methods.

Despite the ~~method shows high independence from the scanner and protocol of the MRI data . Importantly, the presented approach proves effective even when the model is tested using MRIs from protocols not present in the training data. Notably, the method also proves to be effective in scenarios with relatively limited data, where only around 50 MRIs are available, considering the classification task studied here. However, despite the~~ promising results of the presented method, ~~it is important to note its limitations . Firstly, the current approach was tested with FLAIR images, and evaluation with other MRI sequences relevant to WM abnormalities, such as T2, has not yet been conducted. Secondly,~~ its limitations should be considered. HeteroMRI faces a challenge with the registration problem with certain MRIs leads to a false prediction~~by the model, which presents a challenge that needs to be addressed. While a~~. For addressing this challenge, a more elaborate registration strategy may reduce the misalignments ~~, this aspect is not the primary focus of the current study~~which is a future work direction for further improving the method. Notably, 2D MRIs ~~have been~~ were identified as one of the factors ~~contributing~~ that can contribute to registration issues, strongly suggesting the use of 3D MRIs ~~for the model. Thirdly, in~~ with HeteroMRI. Further factors that cause an incorrect registration were not investigated in this study. Another limitation of HeteroMRI is the high GPU memory requirement which is not easily available in every computing server. For each specific use case, one can evaluate the method's performance by downscaling the dimension of input images to decrease the required GPU memory. In this study, ~~the intended intensity cluster was chosen~~

~~manually among the three clusters. This process could potentially be automated by training a classifier model for this task.~~ HeteroMRI was not directly compared with other methods due to the reason that we do not know of any method that performs binary classification for detecting MRIs with WM abnormality, even with standardized data. On the other hand, comparing our method with widely available lesion segmentation methods is not practical as they usually require lesion annotation masks. In addition, a binary classification of MRIs based on the lesion segmentation result is controversial as a threshold is necessary as a minimum value for considering the segmented lesion volume as a WM abnormality in the brain. Furthermore, HeteroMRI was evaluated with only FLAIR images being the superior MRI image for analyzing WM abnormalities. However, the method can be evaluated with other MRI sequences, such as T1 and T2, for instance, to study brain conditions related to other brain tissues. Additionally, the design of HeteroMRI can be upgraded to a multi-channel format, enabling the integration of multiple MRI sequences per subject for enhanced analysis.

~~This approach paves the way for AI-driven analysis of the abundant MRI data available at medical centers, even when these datasets include data from different protocols and scanners . Additionally, the~~

6 Conclusion

In this study, we introduced HeteroMRI, a novel approach for robust classification of brain MRIs based on white matter abnormalities, specifically designed to handle heterogeneous MRI data acquired from diverse scanners and acquisition protocols. The method is also adaptable to standardized MRI datasets acquired using a uniform scanner and protocol~~settings. Looking ahead, our~~ . HeteroMRI achieved high accuracy in detecting MRIs with WM abnormalities, even in scenarios with limited data. Furthermore, the method proved to be generalizable to unseen MRI protocols, highlighting its robustness. There is room for enhancing HeteroMRI's performance by improving the registration accuracy. Our future research will focus on applying this approach to differentiate between unspecific and disease-associated WM lesions, as well as to classify rare demyelinating diseases against their differential diagnoses.

7 Availability of Supporting Source Code and Requirements

Project name: HeteroMRI v1.0

Project home page: https://github.com/ul-mds/HeteroMRI

Operating system(s): Linux-based OS (Ubuntu recommended)

Programming language: Python

Other requirements: TensorFlow v2.x, NVIDIA GPU with CUDA support

License: GNU GPL version 3

8 Data Availability

All the datasets used in this study are either publicly available or are accessible upon request to the respective dataset providers as referenced in Table 1.

9 Abbreviations

AI: Artificial Intelligence; ANTs: Advanced Normalization Tools; AUROC: Area Under the Receiver Operating Characteristic Curve; BTH: Baghdad Teaching Hospital; CSF: CerebroSpinal Fluid; CT: Computed Tomography; CNN: Convolutional Neural Network; DL: Deep Learning; TE: Echo Time; FLAIR: Fluid-Attenuated Inversion Recovery; FCM: Fuzzy C-Means; GM: Gray Matter; ICBM: International Consortium for Brain Mapping; TI: Inversion Time; ML: Machine Learning; $MLcps$: Machine Learning Cumulative Performance Score; MRI: Magnetic Resonance Imaging; MNI: Montreal Neurological Institute; MS: Multiple Sclerosis; NifTI: Neuroimaging Informatics Technology Initiative; ReLU: Rectified Linear Unit; TR: Repetition Time; RFCM: Robust Fuzzy C-Means; 3D: three-dimensional; WM: White Matter.

10 Competing Interests

The authors declare that they have no competing interests

11 Authors' Contributions

M.A. (Methodology, Formal Analysis, Software), N.SH. (Conceptualization, Methodology, Writing – Original Draft), P.L.B. (Methodology), N.SC. (Supervision), J.L. (Supervision, Data Curation), C.C.B. (Supervision), W.K. (Supervision, Data Curation, Project Investigation, Funding Acquisition), T.K. (Supervision, Project Investigation, Funding Acquisition). All authors contributed to reviewing and editing the manuscript.

12 Funding

The authors acknowledge the financial support by the Federal Ministry of Health of Germany in project LeukoExpert (grant no. ZMVI1-2520DAT94), the State Ministry for Education and Research of Germany in the project Tag-White (grant no. 100602109), and the Federal Ministry of Education and Research of Germany and by Sächsische Staatsministerium für Wissenschaft, Kultur und Tourismus in the programme Center of Excellence for AI-research "Center for Scalable Data Analytics and Artificial Intelligence Dresden/Leipzig", project identification number: ScaDS.AI. Supported by the Open Access Publication Fund of Leipzig University.

13 Acknowledgments

The authors sincerely thank Dr. Sina Sadeghi for his insightful comments on the manuscript. The authors are grateful to the Center for Information Services and High-Performance Computing [Zentrum für Informationsdienste und Hochleistungsrechnen (ZIH)] at TU Dresden for providing its facilities for high throughput calculations. Data were provided in part by OASIS Longitudinal Multimodal Neuroimaging: Principal Investigators: T. Benzinger, D. Marcus, J. Morris; NIH P30 AG066444, P50 AG00561, P30 NS09857781, P01 AG026276, P01 AG003991, R01 AG043434, UL1 TR000448, R01 EB009352. AV-45 doses were provided by Avid Radiopharmaceuticals, a wholly owned subsidiary of Eli Lilly. Part of the Data collection and sharing for the Alzheimer's Disease Neuroimaging Initiative (ADNI) is funded by the National Institute on Aging (National Institutes of Health Grant U19 AG024904). The grantee organization is the Northern California Institute for Research and Education. In the past, ADNI has also received funding from the National Institute of Biomedical Imaging and Bioengineering, the Canadian Institutes of Health Research, and private sector contributions through the Foundation for the National Institutes of Health (FNIH) including generous contributions from the following: AbbVie, Alzheimer's Association; Alzheimer's Drug Discovery Foundation; Araclon Biotech; BioClinica, Inc.; Biogen; Bristol-Myers Squibb Company; CereSpir, Inc.; Cogstate; Eisai Inc.; Elan Pharmaceuticals, Inc.; Eli Lilly and Company; EuroImmun; F. Hoffmann-La Roche Ltd and its affiliated company Genentech, Inc.; Fujirebio; GE Healthcare; IXICO Ltd.; Janssen Alzheimer Immunotherapy Research & Development, LLC.; Johnson & Johnson Pharmaceutical Research &Development LLC.; Lumosity; Lundbeck; Merck & Co., Inc.; Meso Scale Diagnostics, LLC.; NeuroRx Research; Neurotrack Technologies; Novartis Pharmaceuticals Corporation; Pfizer Inc.; Piramal Imaging; Servier; Takeda Pharmaceutical Company; and Transition Therapeutics. Part of the data collection and sharing for this project was provided by the International Consortium for Brain Mapping (ICBM; Principal Investigator: John Mazziotta, MD, PhD). ICBM funding was provided by the National Institute of Biomedical Imaging and BioEngineering. ICBM data are disseminated by the Laboratory of Neuro Imaging at the University of Southern California. Part of the data was provided in collaboration with The Observatoire Français de la Sclérose en Plaques (OFSEP), which is supported by a grant provided by the French State and handled by the "Agence Nationale de la Recherche," within the framework of the "Investments for the Future" program, under the reference ANR-10-COHO-002, by the Eugène Devic EDMUS Foundation against multiple sclerosis and by the ARSEP Foundation.

14 Additional Files

Supplementary Table S1. The experimental setting A and details of the MRI data used.

Supplementary Table S2. The experimental setting B and details of the MRI data used.

Supplementary Table S3. The experimental setting C and details of the MRI data used.

Supplementary Table S4. The experimental setting D and details of the MRI data used.

Supplementary Fig. S1. Average required time for preprocessing and intensity clustering of five sample MRI dimensions.

Supplementary Fig. S2. Average required time for training the CNN model of each experimental set-

ting.

Supplementary Fig. S3. Normalized histogram of accuracy values from 7,000 permutations of shuffled labels (orange) and from the 20 data shuffles of the original labels (blue). Dashed lines indicate the mean accuracy for each group, highlighting the significant difference between chance-level accuracy and the model's performance with true labels.

Supplementary Fig. S4. The WM intensity cluster of a sample MRI (a) before and (b) after applying a threshold value of 0.5. The normalized histograms of their 99% upper percentile are shown in (c) and (d), respectively.

References

[1] T. Yousaf, G. Dervenoulas, & M. Politis (2018) *Chapter two - advances in MRI methodology*, M. Politis (Ed.) *Imaging in Movement Disorders: Imaging Methodology and Applications in Parkinson's Disease*, vol. 141 of *International Review of Neurobiology*, pp. 31–76, Academic Press

[2] F. Agosta, S. Galantucci, & M. Filippi (2017) *Advanced magnetic resonance imaging of neurodegenerative diseases*, Neurological Sciences, **38(1)**:pp. 41–51

[3] M. Filippi, P. Preziosa, B. L. Banwell, F. Barkhof, O. Ciccarelli, N. De Stefano, J. J. G. Geurts, F. Paul, D. S. Reich, A. T. Toosy, A. Traboulsee, M. P. Wattjes, T. A. Yousry, A. Gass, C. Lubetzki, B. G. Weinshenker, & M. A. Rocca (2019) *Assessment of lesions on magnetic resonance imaging in multiple sclerosis: practical guidelines*, Brain, **142(7)**:pp. 1858–1875

[4] C. Domínguez-Fernández, J. Egiguren-Ortiz, J. Razquin, M. Gómez-Galán, L. De las Heras-García, E. Paredes-Rodríguez, E. Astigarraga, C. Miguélez, & G. Barreda-Gómez (2023) *Review of technological challenges in personalised medicine and early diagnosis of neurodegenerative disorders*, International Journal of Molecular Sciences, **24(4)**

[5] H.-P. Chan, R. K. Samala, L. M. Hadjiiski, & C. Zhou (2020) *Deep learning in medical image analysis*, G. Lee & H. Fujita (Eds.) *Deep Learning in Medical Image Analysis : Challenges and Applications*, pp. 3–21, Springer International Publishing, Cham

[6] A. S. Panayides, A. Amini, N. D. Filipovic, A. Sharma, S. A. Tsaftaris, A. Young, D. Foran, N. Do, S. Golemati, T. Kurc, K. Huang, K. S. Nikita, B. P. Veasey, M. Zervakis, J. H. Saltz, & C. S. Pattichis (2020) *AI in medical imaging informatics: Current challenges and future directions*, IEEE Journal of Biomedical and Health Informatics, **24(7)**:pp. 1837–1857

[7] B. J. Erickson, P. Korfiatis, Z. Akkus, & T. L. Kline (2017) *Machine learning for medical imaging*, RadioGraphics, **37(2)**:pp. 505–515, pMID: 28212054

[8] M. L. Giger (2018) *Machine learning in medical imaging*, Journal of the American College of Radiology, **15(3, Part B)**:pp. 512–520, data Science: Big Data Machine Learning and Artificial Intelligence

[9] S. Suganyadevi, V. Seethalakshmi, & K. Balasamy (2022) *A review on deep learning in medical image analysis*, International Journal of Multimedia Information Retrieval, **11(1)**:pp. 19–38

[10] M. I. Razzak, S. Naz, & A. Zaib (2018) *Deep learning for medical image processing: Overview, challenges and the future*, N. Dey, A. S. Ashour, & S. Borra (Eds.) *Classification in BioApps: Automation of Decision Making*, pp. 323–350, Springer International Publishing, Cham

[11] N. Garg, M. S. Choudhry, & R. M. Bodade (2023) *A review on Alzheimer's disease classification from normal controls and mild cognitive impairment using structural MR images*, Journal of Neuroscience Methods, **384**:p. 109745

[12] J. Wen, E. Thibeau-Sutre, M. Diaz-Melo, J. Samper-González, A. Routier, S. Bottani, D. Dormont, S. Durrleman, N. Burgos, & O. Colliot (2020) *Convolutional neural networks for classification of Alzheimer's disease: Overview and reproducible evaluation*, Medical Image Analysis, **63**:p. 101694

[13] A. Kursad Poyraz, S. Dogan, E. Akbal, & T. Tuncer (2022) *Automated brain disease classification using exemplar deep features*, Biomedical Signal Processing and Control, **73**:p. 103448

[14] D. García-Lorenzo, S. Francis, S. Narayanan, D. L. Arnold, & D. Louis Collins (2013) *Review of automatic segmentation methods of multiple sclerosis white matter lesions on conventional magnetic resonance imaging*, Medical Image Analysis, **17(1)**:pp. 1–18

[15] O. Cetin, V. Seymen, & U. Sakoglu (2020) *Multiple sclerosis lesion detection in multimodal MRI using simple clustering-based segmentation and classification*, Informatics in Medicine Unlocked, **20**:p. 100409

[16] J. Amin, M. Sharif, M. Yasmin, & S. L. Fernandes (2020) *A distinctive approach in brain tumor detection and classification using MRI*, Pattern Recognition Letters, **139**:pp. 118–127

[17] M. A. Naser & M. J. Deen (2020) *Brain tumor segmentation and grading of lower-grade glioma using deep learning in MRI images*, Computers in Biology and Medicine, **121**:p. 103758

[18] S. Zhang, S. Xu, L. Tan, H. Wang, & J. Meng (2021) *Stroke lesion detection and analysis in MRI images based on deep learning*, Journal of Healthcare Engineering, **2021**:p. 5524769

[19] Y. Kabir, M. Dojat, B. Scherrer, F. Forbes, & C. Garbay (2007) *Multimodal MRI segmentation of ischemic stroke lesions*, *2007 29th Annual International Conference of the IEEE Engineering in Medicine and Biology Society*, pp. 1595–1598

[20] H. Peng, W. Gong, C. F. Beckmann, A. Vedaldi, & S. M. Smith (2021) *Accurate brain age prediction with lightweight deep neural networks*, Medical Image Analysis, **68**:p. 101871

[21] H. Sajedi & N. Pardakhti (2019) *Age prediction based on brain MRI image: A survey*, Journal of Medical Systems, **43(8)**:p. 279

[22] W. M. van Oostveen & E. C. M. de Lange (2021) *Imaging techniques in Alzheimer's disease: A review of applications in early diagnosis and longitudinal monitoring*, International Journal of Molecular Sciences, **22(4)**

[23] R. Zivadinov, J. Sepcic, D. Nasuelli, R. D. Masi, L. M. Bragadin, M. A. Tommasi, S. Zambito-Marsala, R. Moretti, A. Bratina, M. Ukmar, R. S. Pozzi-Mucelli, A. Grop, G. Cazzato, & M. Zorzon (2001) *A longitudinal study of brain atrophy and cognitive disturbances in the early phase of relapsing-remitting multiple sclerosis*, Journal of Neurology, Neurosurgery & Psychiatry, **70(6)**:pp. 773–780

[24] J. West, J. B. M. Warntjes, & P. Lundberg (2012) *Novel whole brain segmentation and volume estimation using quantitative MRI*, European Radiology, **22(5)**:pp. 998–1007

[25] S. Valverde, A. Oliver, E. Roura, S. González-Villà, D. Pareto, J. C. Vilanova, L. Ramió-Torrentà, Àlex Rovira, & X. Lladó (2017) *Automated tissue segmentation of MR brain images in the presence of white matter lesions*, Medical Image Analysis, **35**:pp. 446–457

[26] N. Andrade, F. A. Faria, & F. A. M. Cappabianco (2018) *A practical review on medical image registration: From rigid to deep learning based approaches*, *2018 31st SIBGRAPI Conference on Graphics, Patterns and Images (SIBGRAPI)*, pp. 463–470

[27] J. Kleesiek, G. Urban, A. Hubert, D. Schwarz, K. Maier-Hein, M. Bendszus, & A. Biller (2016) *Deep MRI brain extraction: A 3D convolutional neural network for skull stripping*, NeuroImage, **129**:pp. 460–469

[28] P. Kalavathi & V. B. S. Prasath (2016) *Methods on skull stripping of MRI head scan images—a review*, Journal of Digital Imaging, **29(3)**:pp. 365–379

[29] A. Kaur & G. Dong (2023) *A complete review on image denoising techniques for medical images*, Neural Processing Letters, **55(6)**:pp. 7807–7850

[30] J. Mohan, V. Krishnaveni, & Y. Guo (2014) *A survey on the magnetic resonance image denoising methods*, Biomedical Signal Processing and Control, **9**:pp. 56–69

[31] J. V. Manjón, J. Carbonell-Caballero, J. J. Lull, G. García-Martí, L. Martí-Bonmatí, & M. Robles (2008) *MRI denoising using non-local means*, Medical Image Analysis, **12(4)**:pp. 514–523

[32] M. Shah, Y. Xiao, N. Subbanna, S. Francis, D. L. Arnold, D. L. Collins, & T. Arbel (2011) *Evaluating intensity normalization on MRIs of human brain with multiple sclerosis*, Medical Image Analysis, **15(2)**:pp. 267–282

[33] C. Loizou, M. Pantziaris, I. Seimenis, & C. Pattichis (2009) *Brain MR image normalization in texture analysis of multiple sclerosis*, *2009 9th International Conference on Information Technology and Applications in Biomedicine*, pp. 1–5

[34] N. J. Tustison, B. B. Avants, P. A. Cook, Y. Zheng, A. Egan, P. A. Yushkevich, & J. C. Gee (2010) *N4ITK: Improved N3 bias correction*, IEEE Transactions on Medical Imaging, **29(6)**:pp. 1310–1320

[35] M. Joliot & B. Mazoyer (1993) *Three-dimensional segmentation and interpolation of magnetic resonance brain images*, IEEE Transactions on Medical Imaging, **12(2)**:pp. 269–277

[36] A. Bischoff-Grethe, I. B. Ozyurt, E. Busa, B. T. Quinn, C. Fennema-Notestine, C. P. Clark, S. Morris, M. W. Bondi, T. L. Jernigan, A. M. Dale, G. G. Brown, & B. Fischl (2007) *A technique for the deidentification of structural brain MR images*, Human Brain Mapping, **28(9)**:pp. 892–903

[37] R. Shinohara, J. Oh, G. Nair, P. Calabresi, C. Davatzikos, J. Doshi, R. Henry, G. Kim, K. Linn, N. Papinutto, D. Pelletier, D. Pham, D. Reich, W. Rooney, S. Roy, W. Stern, S. Tummala, F. Yousuf, A. Zhu, N. Sicotte, R. Bakshi, & the NAIMS Cooperative (2017) *Volumetric analysis from a harmonized multisite brain MRI study of a single subject with multiple sclerosis*, American Journal of Neuroradiology, **38(8)**:pp. 1501–1509

[38] J.-P. Fortin, N. Cullen, Y. I. Sheline, W. D. Taylor, I. Aselcioglu, P. A. Cook, P. Adams, C. Cooper, M. Fava, P. J. McGrath, M. McInnis, M. L. Phillips, M. H. Trivedi, M. M. Weissman, & R. T. Shinohara (2018) *Harmonization of cortical thickness measurements across scanners and sites*, NeuroImage, **167**:pp. 104–120

[39] J.-P. Fortin, D. Parker, B. Tunç, T. Watanabe, M. A. Elliott, K. Ruparel, D. R. Roalf, T. D. Satterthwaite, R. C. Gur, R. E. Gur, R. T. Schultz, R. Verma, & R. T. Shinohara (2017) *Harmonization of multi-site diffusion tensor imaging data*, NeuroImage, **161**:pp. 149–170

[40] J.-P. Fortin, E. M. Sweeney, J. Muschelli, C. M. Crainiceanu, & R. T. Shinohara (2016) *Removing inter-subject technical variability in magnetic resonance imaging studies*, NeuroImage, **132**:pp. 198–212

[41] N. De Stefano, M. Battaglini, D. Pareto, R. Cortese, J. Zhang, N. Oesingmann, F. Prados, M. A. Rocca, P. Valsasina, H. Vrenken, C. A. Gandini Wheeler-Kingshott, M. Filippi, F. Barkhof, & Àlex Rovira (2022) *MAGNIMS recommendations for harmonization of MRI data in MS multicenter studies*, NeuroImage: Clinical, **34**:p. 102972

[42] A. J. Hasse (2022) *Quantitative Magnetic Resonance Imaging of Multiple Sclerosis*, Ph.D. thesis, The University of Chicago

[43] G. Mårtensson, D. Ferreira, T. Granberg, L. Cavallin, K. Oppedal, A. Padovani, I. Rektorova, L. Bonanni, M. Pardini, M. G. Kramberger, J.-P. Taylor, J. Hort, J. Snædal, J. Kulisevsky, F. Blanc, A. Antonini, P. Mecocci, B. Vellas, M. Tsolaki, I. Kłoszewska, H. Soininen, S. Lovestone, A. Simmons, D. Aarsland, & E. Westman (2020) *The reliability of a deep learning model in clinical out-of-distribution MRI data: A multicohort study*, Med Image Anal, **66**:p. 101714

[44] F. Hu, A. A. Chen, H. Horng, V. Bashyam, C. Davatzikos, A. Alexander-Bloch, M. Li, H. Shou, T. D. Satterthwaite, M. Yu, & R. T. Shinohara (2023) *Image harmonization: A review of statistical and deep learning methods for removing batch effects and evaluation metrics for effective harmonization*, NeuroImage, **274**:p. 120125

[45] M. E. Torbati, D. S. Minhas, C. M. Laymon, P. Maillard, J. D. Wilson, C.-L. Chen, C. M. Crainiceanu, C. S. DeCarli, S. J. Hwang, & D. L. Tudorascu (2023) *MISPEL: A supervised deep learning harmonization method for multi-scanner neuroimaging data*, Medical Image Analysis, **89**:p. 102926

[46] L. G. Nyúl & J. K. Udupa (1999) *On standardizing the MR image intensity scale*, Magnetic Resonance in Medicine, **42(6)**:pp. 1072–1081

[47] R. T. Shinohara, E. M. Sweeney, J. Goldsmith, N. Shiee, F. J. Mateen, P. A. Calabresi, S. Jarso, D. L. Pham, D. S. Reich, & C. M. Crainiceanu (2014) *Statistical normalization techniques for magnetic resonance imaging*, NeuroImage: Clinical, **6**:pp. 9–19

[48] J. Wrobel, M. Martin, R. Bakshi, P. Calabresi, M. Elliot, D. Roalf, R. Gur, R. Gur, R. Henry, G. Nair, J. Oh, N. Papinutto, D. Pelletier, D. Reich, W. Rooney, T. Satterthwaite, W. Stern, K. Prabhakaran, N. Sicotte, R. Shinohara, & J. Goldsmith (2020) *Intensity warping for multisite MRI harmonization*, NeuroImage, **223**:p. 117242

[49] R. Garcia-Dias, C. Scarpazza, L. Baecker, S. Vieira, W. H. Pinaya, A. Corvin, A. Redolfi, B. Nelson, B. Crespo-Facorro, C. McDonald, D. Tordesillas-Gutiérrez, D. Cannon, D. Mothersill, D. Hernaus,

D. Morris, E. Setien-Suero, G. Donohoe, G. Frisoni, G. Tronchin, J. Sato, M. Marcelis, M. Kempton, N. E. van Haren, O. Gruber, P. McGorry, P. Amminger, P. McGuire, Q. Gong, R. S. Kahn, R. Ayesa-Arriola, T. van Amelsvoort, V. Ortiz-García de la Foz, V. Calhoun, W. Cahn, & A. Mechelli (2020) *Neuroharmony: A new tool for harmonizing volumetric MRI data from unseen scanners*, NeuroImage, **220**:p. 117127

[50] A. Jog, A. Carass, S. Roy, D. L. Pham, & J. L. Prince (2017) *Random forest regression for magnetic resonance image synthesis*, Medical Image Analysis, **35**:pp. 475–488

[51] B. E. Dewey, C. Zhao, J. C. Reinhold, A. Carass, K. C. Fitzgerald, E. S. Sotirchos, S. Saidha, J. Oh, D. L. Pham, P. A. Calabresi, P. C. van Zijl, & J. L. Prince (2019) *Deepharmony: A deep learning approach to contrast harmonization across scanner changes*, Magnetic Resonance Imaging, **64**:pp. 160–170, artificial Intelligence in MRI

[52] B. E. Dewey, L. Zuo, A. Carass, Y. He, Y. Liu, E. M. Mowry, S. Newsome, J. Oh, P. A. Calabresi, & J. L. Prince (2020) *A disentangled latent space for cross-site MRI harmonization*, A. L. Martel, P. Abolmaesumi, D. Stoyanov, D. Mateus, M. A. Zuluaga, S. K. Zhou, D. Racoceanu, & L. Joskowicz (Eds.) *Medical Image Computing and Computer Assisted Intervention – MICCAI 2020*, pp. 720–729, Springer International Publishing, Cham

[53] N. K. Dinsdale, M. Jenkinson, & A. I. Namburete (2021) *Deep learning-based unlearning of dataset bias for MRI harmonisation and confound removal*, NeuroImage, **228**:p. 117689

[54] S. Liu & P.-T. Yap (2024) *Learning multi-site harmonization of magnetic resonance images without traveling human phantoms*, Communications Engineering, **3(1)**:p. 6

[55] L. Zuo, B. E. Dewey, Y. Liu, Y. He, S. D. Newsome, E. M. Mowry, S. M. Resnick, J. L. Prince, & A. Carass (2021) *Unsupervised MR harmonization by learning disentangled representations using information bottleneck theory*, NeuroImage, **243**:p. 118569

[56] F. Hu, A. Lucas, A. A. Chen, K. Coleman, H. Horng, R. W. Ng, N. J. Tustison, K. A. Davis, H. Shou, M. Li, R. T. Shinohara, & T. A. D. N. Initiative (2023) *Deepcombat: A statistically motivated, hyperparameter-robust, deep learning approach to harmonization of neuroimaging data*, bioRxiv

[57] L. L. Resende, A. R. B. de Paiva, F. Kok, C. da Costa Leite, & L. T. Lucato (2019) *Adult leukodystrophies: a step-by-step diagnostic approach*, Radiographics, **39(1)**:pp. 153–168

[58] X. Tu, J. Gao, C. Zhu, J.-Z. Cheng, Z. Ma, X. Dai, & M. Xie (2016) *MR image segmentation and bias field estimation based on coherent local intensity clustering with total variation regularization*, Medical & Biological Engineering & Computing, **54(12)**:pp. 1807–1818

[59] D. Kumar, R. K. Agrawal, & P. Kumar (2022) *Bias-corrected intuitionistic fuzzy c-means with spatial neighborhood information approach for human brain MRI image segmentation*, IEEE Transactions on Fuzzy Systems, **30(3)**:pp. 687–700

[60] A. Khosravanian, M. Rahmanimanesh, P. Keshavarzi, & S. Mozaffari (2021) *Fast level set method for glioma brain tumor segmentation based on superpixel fuzzy clustering and lattice boltzmann method*, Computer Methods and Programs in Biomedicine, **198**:p. 105809

[61] L. Szilágyi, S. M. Szilágyi, B. Benyó, & Z. Benyó (2011) *Intensity inhomogeneity compensation and segmentation of MR brain images using hybrid c-means clustering models*, Biomedical Signal Processing and Control, **6(1)**:pp. 3–12, biomedical signal processing(Extended selected papers from the 7th IFAC Symposium on Modelling and Control in Biomedical Systems(MCBMS'09))

[62] V. Fonov, A. Evans, R. McKinstry, C. Almli, & D. Collins (2009) *Unbiased nonlinear average age-appropriate brain templates from birth to adulthood*, NeuroImage, **47**:p. S102, organization for Human Brain Mapping 2009 Annual Meeting

[63] V. Fonov, A. C. Evans, K. Botteron, C. R. Almli, R. C. McKinstry, & D. L. Collins (2011) *Unbiased average age-appropriate atlases for pediatric studies*, NeuroImage, **54(1)**:pp. 313–327

[64] D. L. Pham (2001) *Spatial models for fuzzy clustering*, Computer Vision and Image Understanding, **84(2)**:pp. 285–297

[65] X. Li, P. S. Morgan, J. Ashburner, J. Smith, & C. Rorden (2016) *The first step for neuroimaging data analysis: DICOM to NIfTI conversion*, Journal of Neuroscience Methods, **264**:pp. 47–56

[66] B. Lowekamp, D. Chen, L. Ibanez, & D. Blezek (2013) *The design of SimpleITK*, Frontiers in Neuroinformatics, **7**:p. 45

[67] J. C. Mazziotta, A. W. Toga, A. Evans, P. Fox, & J. Lancaster (1995) *A probabilistic atlas of the human brain: Theory and rationale for its development: The International Consortium for Brain Mapping (ICBM)*, NeuroImage, **2(2, Part A)**:pp. 89–101

[68] B. B. Avants, N. Tustison, G. Song, et al. (2009) *Advanced normalization tools (ANTs)*, Insight j, **2(365)**:pp. 1–35

[69] J. M. Huntenburg, C. J. Steele, & P.-L. Bazin (2018) *Nighres: processing tools for high-resolution neuroimaging*, GigaScience, **7(7)**:p. giy082

[70] H. Zunair, A. Rahman, N. Mohammed, & J. P. Cohen (2020) *Uniformizing techniques to process CT scans with 3D CNNs for tuberculosis prediction*, I. Rekik, E. Adeli, S. H. Park, & M. d. C. Valdés Hernández (Eds.) *Predictive Intelligence in Medicine*, pp. 156–168, Springer International Publishing, Cham

[71] D. Maturana & S. Scherer (2015) *Voxnet: A 3D convolutional neural network for real-time object recognition*, *2015 IEEE/RSJ International Conference on Intelligent Robots and Systems (IROS)*, pp. 922–928

[72] S. Ioffe & C. Szegedy (2015) *Batch normalization: Accelerating deep network training by reducing internal covariate shift*, F. Bach & D. Blei (Eds.) *Proceedings of the 32nd International Conference on Machine Learning*, vol. 37 of *Proceedings of Machine Learning Research*, pp. 448–456, PMLR, Lille, France

[73] D. P. Kingma & J. Ba (2017) *Adam: A method for stochastic optimization*, arXiv preprint arXiv:1412.6980

[74] A. Carass, S. Roy, A. Jog, J. L. Cuzzocreo, E. Magrath, A. Gherman, J. Button, J. Nguyen, F. Prados, C. H. Sudre, M. Jorge Cardoso, N. Cawley, O. Ciccarelli, C. A. Wheeler-Kingshott, S. Ourselin, L. Catanese, H. Deshpande, P. Maurel, O. Commowick, C. Barillot, X. Tomas-Fernandez, S. K.

Warfield, S. Vaidya, A. Chunduru, R. Muthuganapathy, G. Krishnamurthi, A. Jesson, T. Arbel, O. Maier, H. Handels, L. O. Iheme, D. Unay, S. Jain, D. M. Sima, D. Smeets, M. Ghafoorian, B. Platel, A. Birenbaum, H. Greenspan, P.-L. Bazin, P. A. Calabresi, C. M. Crainiceanu, L. M. Ellingsen, D. S. Reich, J. L. Prince, & D. L. Pham (2017) *Longitudinal multiple sclerosis lesion segmentation: Resource and challenge*, NeuroImage, **148**:pp. 77–102

[75] Ž. Lesjak, A. Galimzianova, A. Koren, M. Lukin, F. Pernuš, B. Likar, & Ž. Špiclin (2018) *A novel public MR image dataset of multiple sclerosis patients with lesion segmentations based on multi-rater consensus*, Neuroinformatics, **16**:pp. 51–63

[76] O. Commowick, A. Istace, M. Kain, B. Laurent, F. Leray, M. Simon, S. C. Pop, P. Girard, R. Améli, J.-C. Ferré, A. Kerbrat, T. Tourdias, F. Cervenansky, T. Glatard, J. Beaumont, S. Doyle, F. Forbes, J. Knight, A. Khademi, A. Mahbod, C. Wang, R. McKinley, F. Wagner, J. Muschelli, E. Sweeney, E. Roura, X. Lladó, M. M. Santos, W. P. Santos, A. G. Silva-Filho, X. Tomas-Fernandez, H. Urien, I. Bloch, S. Valverde, M. Cabezas, F. J. Vera-Olmos, N. Malpica, C. Guttmann, S. Vukusic, G. Edan, M. Dojat, M. Styner, S. K. Warfield, F. Cotton, & C. Barillot (2018) *Objective evaluation of multiple sclerosis lesion segmentation using a data management and processing infrastructure*, Scientific Reports, **8(1)**:p. 13650

[77] O. Commowick, F. Cervenansky, F. Cotton, & M. Dojat (Eds.) (2021) *MSSEG-2 challenge proceedings: Multiple sclerosis new lesions segmentation challenge using a data management and processing infrastructure*, Strasbourg, France

[78] A. M. Muslim, S. Mashohor, G. A. Gawwam, R. Mahmud, M. binti Hanafi, O. Alnuaimi, R. Josephine, & A. D. Almutairi (2022) *Brain MRI dataset of multiple sclerosis with consensus manual lesion segmentation and patient meta information*, Data in Brief, **42**:p. 108139

[79] R. Kötter, J. Mazziotta, A. Toga, A. Evans, P. Fox, J. Lancaster, K. Zilles, R. Woods, T. Paus, G. Simpson, B. Pike, C. Holmes, L. Collins, P. Thompson, D. MacDonald, M. Iacoboni, T. Schormann, K. Amunts, N. Palomero-Gallagher, S. Geyer, L. Parsons, K. Narr, N. Kabani, G. L. Goualher, D. Boomsma, T. Cannon, R. Kawashima, & B. Mazoyer (2001) *A probabilistic atlas and reference system for the human brain: International Consortium for Brain Mapping (ICBM)*, Philosophical Transactions of the Royal Society of London. Series B: Biological Sciences, **356(1412)**:pp. 1293–1322

[80] P. J. LaMontagne, T. L. Benzinger, J. C. Morris, S. Keefe, R. Hornbeck, C. Xiong, E. Grant, J. Hassenstab, K. Moulder, A. G. Vlassenko, M. E. Raichle, C. Cruchaga, & D. Marcus (2019) *OASIS-3: Longitudinal neuroimaging, clinical, and cognitive dataset for normal aging and alzheimer disease*, medRxiv

[81] C. R. Jack Jr., M. A. Bernstein, N. C. Fox, P. Thompson, G. Alexander, D. Harvey, B. Borowski, P. J. Britson, J. L. Whitwell, C. Ward, A. M. Dale, J. P. Felmlee, J. L. Gunter, D. L. Hill, R. Killiany, N. Schuff, S. Fox-Bosetti, C. Lin, C. Studholme, C. S. DeCarli, G. Krueger, H. A. Ward, G. J. Metzger, K. T. Scott, R. Mallozzi, D. Blezek, J. Levy, J. P. Debbins, A. S. Fleisher, M. Albert, R. Green, G. Bartzokis, G. Glover, J. Mugler, & M. W. Weiner (2008) *The Alzheimer's disease neuroimaging initiative (ADNI): MRI methods*, Journal of Magnetic Resonance Imaging, **27(4)**:pp. 685–691

[82] I. Mérida, J. Jung, S. Bouvard, D. Le Bars, S. Lancelot, F. Lavenne, C. Bouillot, J. Redouté, A. Hammers, & N. Costes (2021) *CERMEP-IDB-MRXFDG: a database of 37 normal adult human brain [^{18}F]FDG PET, T1 and FLAIR MRI, and CT images available for research*, EJNMMI Research, **11(1)**:p. 91

[83] S. Vukusic, R. Casey, F. Rollot, B. Brochet, J. Pelletier, D.-A. Laplaud, J. D. Sèze, F. Cotton, T. Moreau, B. Stankoff, B. Fontaine, F. Guillemin, M. Debouverie, & M. Clanet (2020) *Observatoire Français de la Sclérose en Plaques (OFSEP): A unique multimodal nationwide MS registry in France*, Multiple Sclerosis Journal, **26(1)**:pp. 118–122, pMID: 30541380

[84] C. Confavreux, D. A. Compston, O. R. Hommes, W. I. McDonald, & A. J. Thompson (1992) *EDMUS, a European database for multiple sclerosis.*, Journal of Neurology, Neurosurgery & Psychiatry, **55(8)**:pp. 671–676

[85] A. Akshay, M. Abedi, N. Shekarchizadeh, F. C. Burkhard, M. Katoch, A. Bigger-Allen, R. M. Adam, K. Monastyrskaya, & A. Hashemi Gheinani (2023) *MLcps: machine learning cumulative performance score for classification problems*, GigaScience, **12**:p. giad108

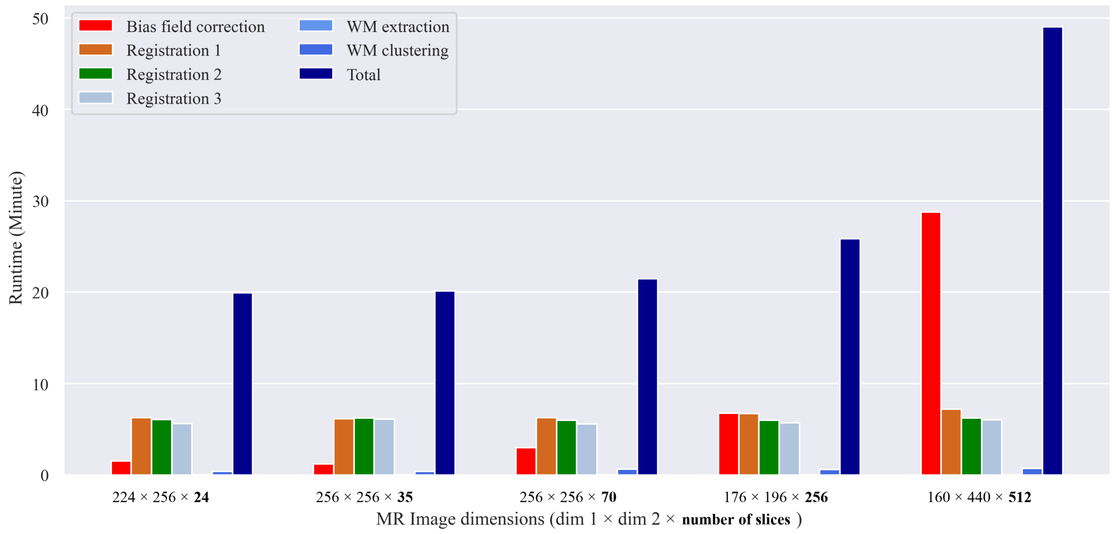

Supplementary Figure S 1: Average required time for preprocessing and intensity clustering of five sample MRI dimensions.

Supplementary Table S 1: Number of MRIs used for training (Tr), validation (V), and test (T) sets from each *dataset* in experimental settings *A*00 to *A*18

Dataset		Set	Setting *A*#																		
**Label***	**Name**		**00**	**01**	**02**	**03**	**04**	**05**	**06**	**07**	**08**	**09**	**10**	**11**	**12**	**13**	**14**	**15**	**16**	**17**	**18**
+	ISBI	Tr	8	7	6	5	4	3	2	1	0	0	0	0	0	0	0	0	0	0	0
		V	1	1	1	1	1	1	1	1	0	0	0	0	0	0	0	0	0	0	0
		T	2	2	2	2	2	2	2	2	2	2	2	2	2	2	2	2	2	2	2
	UMCL	Tr	8	7	6	5	4	3	2	1	0	0	0	0	0	0	0	0	0	0	0
		V	1	1	1	1	1	1	1	1	0	0	0	0	0	0	0	0	0	0	0
		T	2	2	2	2	2	2	2	2	2	2	2	2	2	2	2	2	2	2	2
	MSSEG	Tr	8	7	6	5	4	3	2	1	0	0	0	0	0	0	0	0	0	0	0
		V	1	1	1	1	1	1	1	1	0	0	0	0	0	0	0	0	0	0	0
		T	2	2	2	2	2	2	2	2	2	2	2	2	2	2	2	2	2	2	2
	MSSEG-2	Tr	8	7	6	5	4	3	2	1	0	0	0	0	0	0	0	0	0	0	0
		V	1	1	1	1	1	1	1	1	0	0	0	0	0	0	0	0	0	0	0
		T	2	2	2	2	2	2	2	2	2	2	2	2	2	2	2	2	2	2	2
	BTH	Tr	6	5	4	3	2	1	0	0	0	0	0	0	0	0	0	0	0	0	0
		V	1	1	1	1	1	1	0	0	0	0	0	0	0	0	0	0	0	0	0
		T	2	2	2	2	2	2	2	2	2	2	2	2	2	2	2	2	2	2	2
	OASIS-3	Tr	10	9	8	7	6	5	4	3	2	1	0	0	0	0	0	0	0	0	0
		V	2	2	2	1	1	1	1	1	1	1	0	0	0	0	0	0	0	0	0
		T	2	2	2	2	2	2	2	2	2	2	2	2	2	2	2	2	2	2	2
	ADNI [3]	Tr	39	35	31	28	26	23	21	19	17	14	12	9	7	6	5	4	3	2	1
		V	6	6	6	6	6	6	5	5	5	5	4	4	3	3	3	2	2	1	1
		T	10	10	10	10	10	10	10	10	10	10	10	10	10	10	10	10	10	10	10
−	ICBM	Tr	3	2	1	0	0	0	0	0	0	0	0	0	0	0	0	0	0	0	0
		V	1	1	1	0	0	0	0	0	0	0	0	0	0	0	0	0	0	0	0
		T	1	1	1	1	1	1	1	1	1	1	1	1	1	1	1	1	1	1	1
	OASIS-3	Tr	65	58	51	45	39	32	26	21	15	12	10	8	7	6	5	4	3	2	1
		V	9	9	9	9	9	9	8	8	5	5	3	3	3	3	3	2	2	1	1
		T	16	16	16	16	16	16	16	16	16	16	16	16	16	16	16	16	16	16	16
	CERMEP	Tr	19	17	15	13	11	9	7	5	4	3	2	1	0	0	0	0	0	0	0
		V	3	3	3	3	3	3	2	2	1	1	1	1	0	0	0	0	0	0	0
		T	5	5	5	5	5	5	5	5	5	5	5	5	5	5	5	5	5	5	5
+	Total	Tr	87	77	67	58	50	41	33	26	19	15	12	9	7	6	5	4	3	2	1
		V	13	13	13	12	12	12	10	10	6	6	4	4	3	3	3	2	2	1	1
		T	22	22	22	22	22	22	22	22	22	22	22	22	22	22	22	22	22	22	22
−	Total	Tr	87	77	67	58	50	41	33	26	19	15	12	9	7	6	5	4	3	2	1
		V	13	13	13	12	12	12	10	10	6	6	4	4	3	3	3	2	2	1	1
		T	22	22	22	22	22	22	22	22	22	22	22	22	22	22	22	22	22	22	22
**Total**			244	224	204	184	168	150	130	116	94	86	76	70	64	62	60	56	54	50	48

* With (+) and without (−) WM abnormality

Supplementary Table S 2: Number of MRIs used for training (Tr), validation (V), and test (T) sets from each MRI acquisition *protocol* in experimental settings $B00$ to $B04$

**Protocol**		**Set**	**Setting**				
**Label***	**Name**		$B00$	$B01$	$B02$	$B03$	$B04$
+	Sie_Tri_30_Prot1	Tr	5	4	3	2	1
		V	1	1	1	1	1
		T	1	1	1	1	1
	Phi_Ing_30_NA	Tr	5	4	3	2	1
		V	1	1	1	1	1
		T	1	1	1	1	1
	Phi_NA_30_Prot1	Tr	5	4	3	2	1
		V	1	1	1	1	1
		T	1	1	1	1	1
	Sie_Aer_15_Prot1	Tr	5	4	3	2	1
		V	1	1	1	1	1
		T	1	1	1	1	1
	GeE_Dis_30_Prot3	Tr	5	4	3	2	1
		V	1	1	1	1	1
		T	1	1	1	1	1
−	Sie_Bio_30_Prot1	Tr	5	4	3	2	1
		V	1	1	1	1	1
		T	1	1	1	1	1
	Sie_Son_15_Prot1	Tr	5	4	3	2	1
		V	1	1	1	1	1
		T	1	1	1	1	1
	Sie_MaV_30_Prot2	Tr	5	4	3	2	1
		V	1	1	1	1	1
		T	1	1	1	1	1
	Sie_MaV_30_Prot1	Tr	5	4	3	2	1
		V	1	1	1	1	1
		T	1	1	1	1	1
	Sie_TrT_30_Prot2	Tr	5	4	3	2	1
		V	1	1	1	1	1
		T	1	1	1	1	1
+	Total	Tr	25	20	15	10	5
		V	5	5	5	5	5
		T	5	5	5	5	5
−	Total	Tr	25	20	15	10	5
		V	5	5	5	5	5
		T	5	5	5	5	5
**Total**			70	60	50	40	30

* With (+) and without (−) WM abnormality

Supplementary Table S 3: Number of MRIs used for training (Tr), validation (V), and test (T) sets from each MRI acquisition *protocol* in experimental settings $C00$ to $C06$. The two protocols ~~highlighted~~ in ~~gray~~ boldface are used only as test data. ~~The~~ These two protocols ~~chosen as test data~~ are changed in 10 different cases.

Protocol		Set	Setting						
**Label***	**Name**		$C00$	$C01$	$C02$	$C03$	$C04$	$C05$	$C06$
+	Phi_Ing_30_NA	Tr	7	6	5	4	3	2	1
		V	1	1	1	1	1	1	1
		T	0	0	0	0	0	0	0
	GeE_Dis_30_Prot3	Tr	7	6	5	4	3	2	1
		V	1	1	1	1	1	1	1
		T	0	0	0	0	0	0	0
	Sie_Pri_30_Prot1	Tr	6	5	4	3	2	1	0
		V	2	2	2	1	1	1	0
		T	0	0	0	0	0	0	0
	Sie_Ver_30_Prot1	Tr	6	5	4	3	2	1	0
		V	2	1	1	1	1	1	0
		T	0	0	0	0	0	0	0
	~~Phi_Ing_30_Prot2~~**Phi_Ing_30_Prot2**	Tr	0	0	0	0	0	0	0
		V	0	0	0	0	0	0	0
		T	5	5	5	5	5	5	5
−	Sie_MaV_30_Prot1	Tr	6	5	4	3	2	1	0
		V	1	1	1	1	1	1	0
		T	0	0	0	0	0	0	0
	Sie_Son_15_Prot1	Tr	20	17	14	11	8	5	2
		V	5	4	4	3	3	3	2
		T	0	0	0	0	0	0	0
	~~Sie_TrT_30_Prot1~~**Sie_TrT_30_Prot1**	Tr	0	0	0	0	0	0	0
		V	0	0	0	0	0	0	0
		T	5	5	5	5	5	5	5
+	Total	Tr	26	22	18	14	10	6	2
		V	6	5	5	4	4	4	2
		T	5	5	5	5	5	5	5
−	Total	Tr	26	22	18	14	10	6	2
		V	6	5	5	4	4	4	2
		T	5	5	5	5	5	5	5
**Total**			74	64	56	46	38	30	18

* With (+) and without (−) WM abnormality

Supplementary Table S 4: Number of MRIs used for training (Tr), validation (V), and test (T) sets from each MRI acquisition *protocol* in experimental settings $D00$ to $D03$

**Protocol**		**Set**	**Setting**			
**Label***	**Name**		$D00$	$D01$	$D02$	$D03$
+	Sie_Tri_30_Prot1	Tr	21	12	9	8
		V	3	2	2	1
		T	6	3	2	2
	Phi_Ing_30_NA	Tr	15	12	9	8
		V	2	2	1	1
		T	4	3	3	2
	Phi_NA_30_Prot1	Tr	0	12	9	7
		V	0	1	1	1
		T	0	4	3	2
	Sie_Aer_15_Prot1	Tr	0	0	9	7
		V	0	0	1	1
		T	0	0	2	2
	GeE_Dis_30_Prot3	Tr	0	0	0	6
		V	0	0	0	1
		T	0	0	0	2
−	Sie_Bio_30_Prot1	Tr	21	124	11	9
		V	3	2	2	1
		T	6	3	2	3
	Sie_Son_15_Prot1	Tr	15	12	10	8
		V	2	2	1	1
		T	4	3	4	3
	Sie_MaV_30_Prot2	Tr	0	12	10	9
		V	0	1	1	1
		T	0	4	3	2
	Sie_MaV_30_Prot1	Tr	0	0	5	5
		V	0	0	1	1
		T	0	0	1	1
	Sie_TrT_30_Prot2	Tr	0	0	0	5
		V	0	0	0	1
		T	0	0	0	1
+	Total	Tr	36	36	36	36
		V	5	5	5	5
		T	10	10	10	10
−	Total	Tr	36	36	36	36
		V	5	5	5	5
		T	10	10	10	10
**Total**			102	102	102	102

* With (+) and without (−) WM abnormality

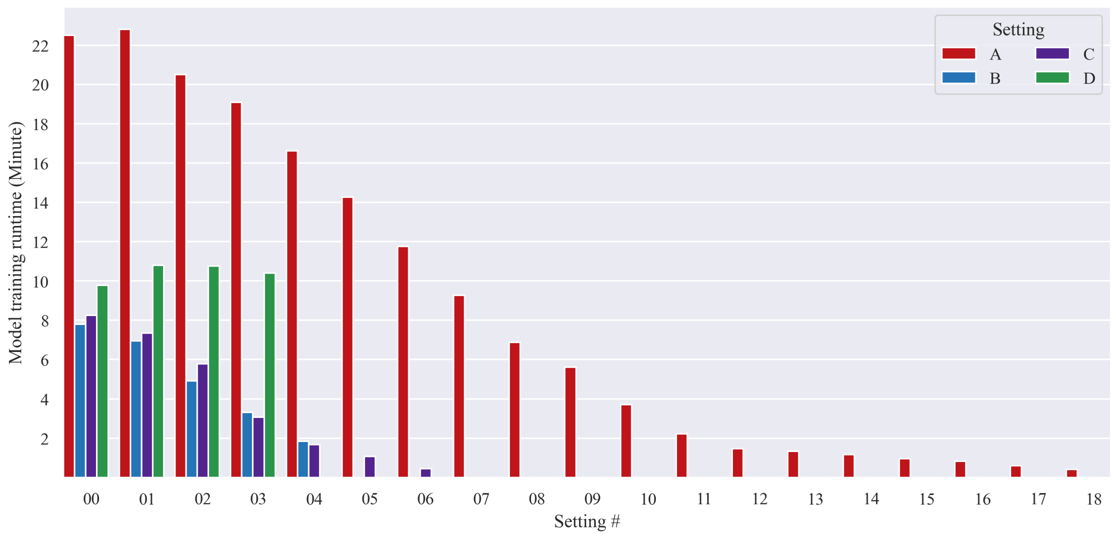

Supplementary Figure S 2: Average required time for training the CNN model of each experimental setting.

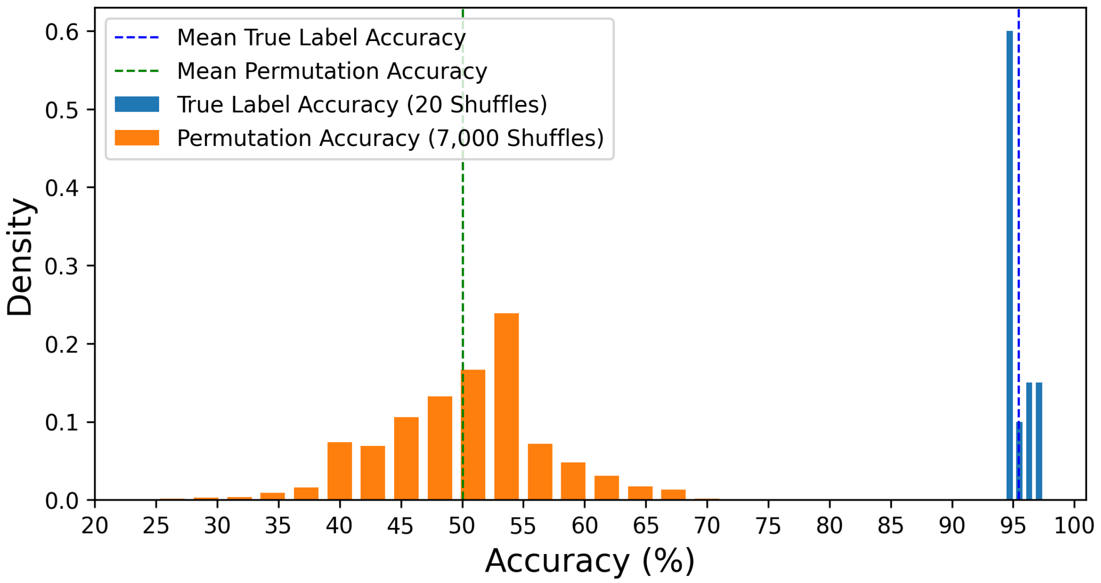

Supplementary Figure S 3: Normalized histogram of accuracy values from 7,000 permutations of shuffled labels (orange) and from the 20 data shuffles of the original labels (blue). Dashed lines indicate the mean accuracy for each group, highlighting the significant difference between chance-level accuracy and the model's performance with true labels.

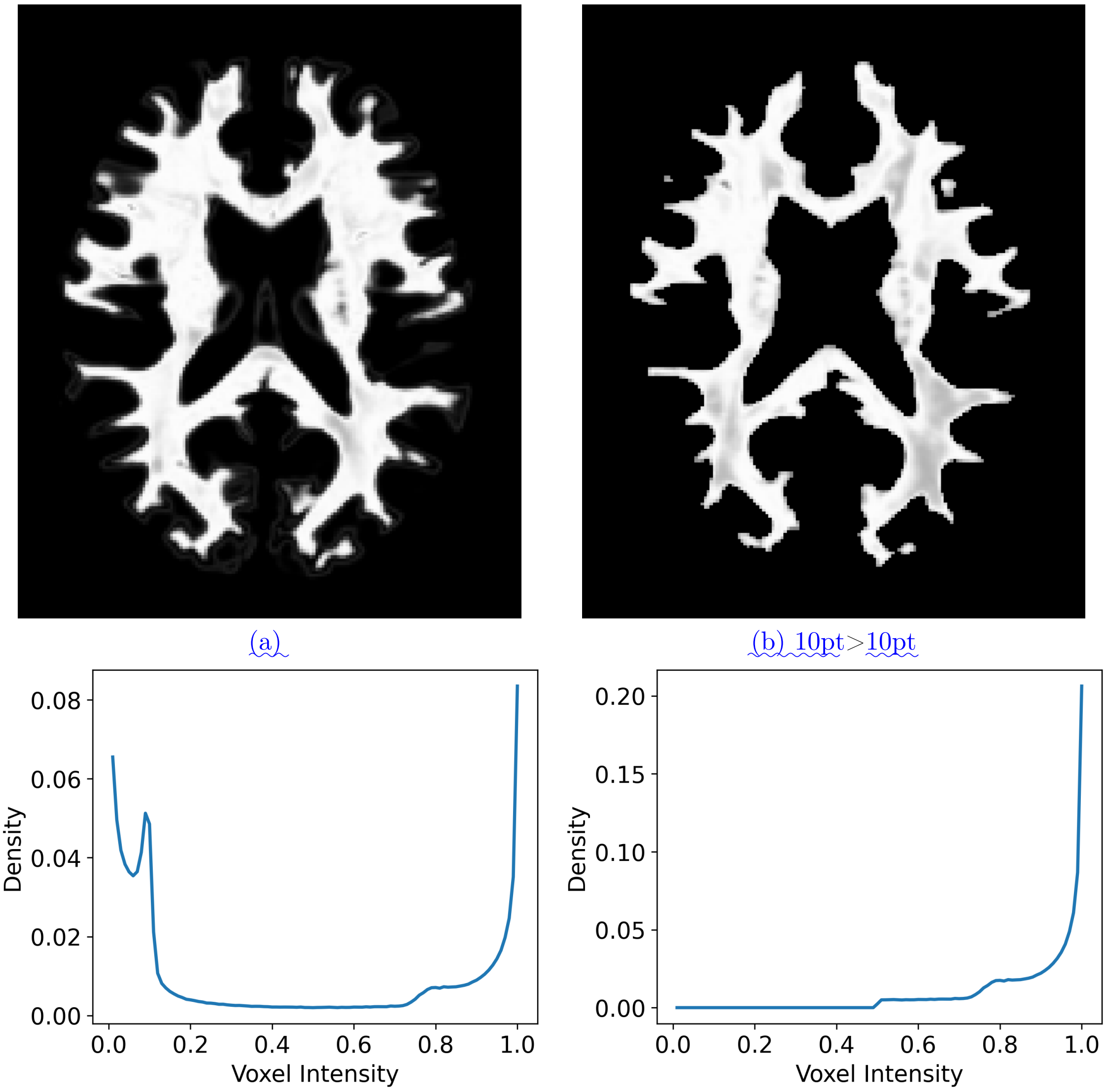

(a) (b) 10pt>10pt

(c) (d) 10pt>10pt

Supplementary Figure S 4: The WM intensity cluster of a sample MRI (a) before and (b) after applying a threshold value of 0.5. The normalized histograms of their 99% upper percentile are shown in (c) and (d), respectively.
