# Supplementary material for: HeteroMRI: Robust white matter abnormality classification across multi-scanner MRI data
Source: Gigascience. 2025 Aug 21;14:giaf092. doi: 10.1093/gigascience/giaf092 (PMC12371411; doi:10.1093/gigascience/giaf092)
Supplement: giaf092_GIGA-D-24-00230_Revision_3 [file giaf092_giga-d-24-00230_revision_3.pdf]

GigaScience

HeteroMRI: Robust white matter abnormality classification across multi-scanner MRI data

--Manuscript Draft--

**Manuscript Number:**	GIGA-D-24-00230R3
**Full Title:**	HeteroMRI: Robust white matter abnormality classification across multi-scanner MRI data
**Article Type:**	Research
**Funding Information:**	Bundesministerium für Gesundheit (ZMVI1-2520DAT94) — Mr. Masoud Abedi<br>Bundesministerium für Bildung und Forschung (ScaDS.AI) — Dr. Navid Shekarchizadeh<br>Bundesministerium für Bildung und Forschung (100602109) — Not applicable
**Abstract:**	Background<br>Magnetic Resonance Imaging (MRI) is commonly used for analyzing white matter abnormalities in the human brain. Integrating machine learning into MRI analysis can enhance diagnostic processes. However, the application of such techniques for white matter analysis in clinical practice is often limited when MRI data is multi-scanner (i.e., heterogeneous), particularly in scenarios with limited data, as seen in rare diseases. Therefore, it is crucial to develop methods that are highly independent of the MRI scanner and acquisition protocol.<br>Results<br>This study introduces HeteroMRI, a deep-learning method for classifying MRIs based on white matter abnormalities. Most importantly, HeteroMRI mitigates the effects of data heterogeneity on classification performance. Herein, HeteroMRI is employed to detect brain MRIs with white matter abnormalities. This method utilizes intensity clustering of the white matter tissue to reduce the effects of the heterogeneity of MRIs. MRI data from 11 public datasets with 40 MRI protocols are included. By using 200 MRIs for training the model, the binary classifier achieves an average accuracy of 93±4%. Furthermore, the method is evaluated in limited data scenarios, simulating conditions of rare diseases. By reducing the data by 64% and 75%, the model's accuracy has a 4% and 12% decrease, respectively.<br>Conclusions<br>The presented method opens new avenues for white matter abnormality-related classification of heterogeneous MRI data without additional machine learning methods to reduce MRI heterogeneity. This classification approach demonstrates a high degree of independence from the MRI scanner and protocol, while also proving to be relatively generalizable to unseen MRI protocols.
**Corresponding Author:**	Navid Shekarchizadeh<br>Leipzig University: Universitat Leipzig<br>Leipzig, GERMANY
**Corresponding Author Secondary Information:**	
**Corresponding Author's Institution:**	Leipzig University: Universitat Leipzig
**Corresponding Author's Secondary Institution:**	
**First Author:**	Masoud Abedi
**First Author Secondary Information:**	
**Order of Authors:**	Masoud Abedi<br>Navid Shekarchizadeh<br>Pierre-Louis Bazin

Powered by Editorial Manager® and ProduXion Manager® from Aries Systems Corporation

	Nico Scherf
	Julia Lier
	Christa-Caroline Bergner
	Wolfgang Köhler
	Toralf Kirsten
**Order of Authors Secondary Information:**	
**Response to Reviewers:**	Dear Dr. Nicole Nogoy,<br><br>We are pleased to hear that our manuscript entitled “HeteroMRI: Robust white matter abnormality classification across multi-scanner MRI data” has been accepted for publication in the GigaScience journal. In the revised manuscript, we have performed the necessary amendments requested by you in the marked-up PDF file sent by you. Moreover, minor character- or word-level corrections have been made in the paper. Additionally, one of the dataset providers requested that an extra piece of text be added to the acknowledgments section. The respected reviewer has not suggested any further changes in his report. In addition to the revised manuscript and all the LaTeX files, we also provide a version of the manuscript with all changes highlighted compared to the previous version.<br><br>At the end, we would like to thank you and the reviewer for their time and thoughtful comments, and efforts towards improving our manuscript. We would remain available for any further information/corrections from our side.<br><br><br>Sincerely,<br>Navid Shekarchizadeh<br>On behalf of the authors
**Additional Information:**	
**Question**	**Response**
Are you submitting this manuscript to a special series or article collection?	No
**Experimental design and statistics**<br><br><br>Full details of the experimental design and statistical methods used should be given in the Methods section, as detailed in our Minimum Standards Reporting Checklist. Information essential to interpreting the data presented should be made available in the figure legends.<br><br><br>Have you included all the information requested in your manuscript?	Yes
**Resources**<br><br><br>A description of all resources used, including antibodies, cell lines, animals and software tools, with enough	Yes

Powered by Editorial Manager® and ProduXion Manager® from Aries Systems Corporation

information to allow them to be uniquely identified, should be included in the Methods section. Authors are strongly encouraged to cite Research Resource Identifiers (RRIDs) for antibodies, model organisms and tools, where possible.<br><br>Have you included the information requested as detailed in our Minimum Standards Reporting Checklist?	
**Availability of data and materials**<br><br>All datasets and code on which the conclusions of the paper rely must be either included in your submission or deposited in publicly available repositories (where available and ethically appropriate), referencing such data using a unique identifier in the references and in the “Availability of Data and Materials” section of your manuscript.<br><br>Have you have met the above requirement as detailed in our Minimum Standards Reporting Checklist?	Yes

Powered by Editorial Manager® and ProduXion Manager® from Aries Systems Corporation

LaTeX - Main document

Click here to access/download;LaTeX - Main Document (TeX file);main.tex

HeteroMRI: Robust white matter abnormality classification across multi-scanner MRI data

Masoud Abedi [1,2,3,†], Navid Shekarchizadeh [2,3,4,†,ğ], Pierre-Louis Bazin [5], Nico Scherf [4,6], Julia Lier [7,8], Christa-Caroline Bergner[7,8], for the Alzheimers Disease Neuroimaging Initiative[*] , Wolfgang Köhler[7,8,‡], and Toralf Kirsten [1,2,3,4,‡]

[1]Faculty Applied Computer and Bio Sciences, Mittweida University of Applied Sciences, Mittweida, Germany

[2]Department for Medical Data Science, Leipzig University Medical Center, Leipzig, Germany

[3]Institute for Medical Informatics, Statistics, and Epidemiology (IMISE), Leipzig University, Leipzig, Germany

[4]Center for Scalable Data Analytics and Artificial Intelligence (ScaDS.AI) Dresden/Leipzig, Leipzig University, Leipzig, Germany

[5]Full brain picture Analytics, Leiden, The Netherlands

[6]Neural Data Science and Statistical Computing, Max Planck Institute for Human Cognitive and Brain Sciences, Leipzig, Germany

[7]Department of Neurology, Leipzig University Medical Center, Leipzig, Germany

[8]Myelin Research Center (MRC) Leipzig, Department of Neurology, Leipzig University Medical Center, Leipzig, Germany

[§]Correspondence address. Navid Shekarchizadeh, ScaDS.AI, HumboldtstraSSe 25, 04105 Leipzig, Germany. E-mail: navid.shekarchizadeh@uni-leipzig.de

[†]Equal contribution

[‡]Joint senior authorship

Abstract

Background

Magnetic Resonance Imaging (MRI) is commonly used for analyzing white matter abnormalities in the human brain. Integrating machine learning into MRI analysis can enhance diagnostic processes. However, the application of such techniques for white matter analysis in clinical practice is often limited when MRI data is multi-scanner (i.e., heterogeneous), particularly in scenarios with limited data, as seen in rare diseases. Therefore, it is crucial to develop methods that are highly independent of the MRI scanner and acquisition protocol.

Results

This study introduces *HeteroMRI*, a deep-learning method for classifying MRIs based on white

[*]A part of the data used in preparation of this article were obtained from the Alzheimers Disease Neuroimaging Initiative (ADNI) database (adni.loni.usc.edu). As such, the investigators within the ADNI contributed to the design and implementation of ADNI and/or provided data but did not participate in analysis or writing of this report. A complete listing of ADNI investigators can be found at: http://adni.loni.usc.edu/wp-content/uploads/how_to_apply/ADNI_Acknowledgement_List.pdf

matter abnormalities. Most importantly, HeteroMRI mitigates the effects of data heterogeneity on classification performance. Herein, HeteroMRI is employed to detect brain MRIs with white matter abnormalities. This method utilizes intensity clustering of the white matter tissue to reduce the effects of the heterogeneity of MRIs. MRI data from 11 public datasets with 40 MRI protocols are included. By using 200 MRIs for training the model, the binary classifier achieves an average accuracy of 93±4%. Furthermore, the method is evaluated in limited data scenarios, simulating conditions of rare diseases. By reducing the data by 64% and 75%, the model's accuracy has a 4% and 12% decrease, respectively.

Conclusions

The presented method opens new avenues for white matter abnormality-related classification of heterogeneous MRI data without additional machine learning methods to reduce MRI heterogeneity. This classification approach demonstrates a high degree of independence from the MRI scanner and protocol, while also proving to be relatively generalizable to unseen MRI protocols.

Keywords— Brain MRI classification, Multi-scanner MRI, Multi-protocol MRI, Intensity clustering, White matter abnormality, Rare disease, Convolutional neural network

1 Introduction

Magnetic Resonance Imaging (MRI) of the brain is widely used to diagnose neurological diseases as it provides a clear contrast between the different tissues of the brain, including white matter (WM) and gray matter (GM) [1]. Detection and assessment of WM abnormalities or lesions in demyelinating or neurodegenerative diseases are an important application of MRI in daily clinical practice [2]. An excellent contrast for visualizing WM abnormalities is provided by the Fluid-Attenuated Inversion Recovery (FLAIR) imaging technique, making the abnormalities stand out from the surrounding normal brain tissue. FLAIR is a T2-weighted imaging technique in which the signal from cerebrospinal fluid (CSF) is suppressed, which facilitates the detection of WM abnormalities as they may appear adjacent to CSF-filled spaces [3]. The MRI technique allows for the study of the pattern and volume of WM lesions, which ultimately contributes to image-based diagnosis of demyelinating and other neurological disorders [4].

In recent years, Artificial Intelligence (AI) has revolutionized the medical imaging domain, offering powerful tools for automating time-consuming tasks such as lesion segmentation [5]. This reduces examiner-based variability and enables healthcare professionals to focus on critical aspects of diagnosis and research. In brain MRI analysis, Machine Learning (ML) and Deep Learning (DL) models have been widely applied [6] for tasks including disease classification [7, 8, 9], WM lesion segmentation [10, 11], tumor detection and grading [12, 13], stroke lesion analysis [14, 15], brain age prediction [16, 17], and brain tissue segmentation [18, 19]. Additionally, AI-based algorithms are commonly used in MRI preprocessing steps such as image registration [20], brain extraction [21, 22], denoising [23, 24, 25], intensity normalization [26, 27], bias field correction [28], and MRI interpolation [29].

A challenge in using MRI data in ML/DL models is the variability of MRIs across different sites and scanners. It is shown that scanner differences lead to significant biases in automated MS lesion volumetric analyses, even when the scanner manufacturer and acquisition protocol are consistent [30]. Acquisition protocol refers to a set of procedures and parameters, e.g., Echo Time (TE), Repetition Time (TR), and Inversion Time (TI), used to acquire the images. The variabilities due to different scanners and acquisition protocols are often greater than the biological variabilities [31, 32, 33, 34, 35, 36]. High-capacity classifiers, such as deep neural networks, often struggle to produce consistent outcomes when applied to multi-scanner data. This limitation is caused by the model's tendency to overfit to non-biological variations; thus, the model fails to detect desired biological features or to generalize well across MRI data from unseen scanners [37].

A common approach to address scanner variability is using standardized MRI datasets—acquired with identical scanners and protocols—to improve model consistency [38, 39]. While standardization enhances image comparability and model performance, it requires substantial coordination and resources. Moreover, many DL models still fail to generalize to unseen scanners or protocols [40], limiting their clinical utility. Thus, methods that are robust to scanner and protocol variability are crucial.

Another approach to address scanner-related heterogeneity in MRI data is *harmonization*, which aims to remove scanner and protocol effects before analysis. Statistical techniques such as intensity normalization [41, 26] or ComBat-based batch effect correction [33, 32, 31, 34, 35, 36] have shown success to some extent, but often fail to improve DL model performance in disease classification tasks [37]. More recent harmonization approaches rely on supervised [42, 43] and unsupervised [44, 45] DL methods, with hybrid models like DeepComBat also emerging [46]. While these methods aim to learn and remove scanner-specific features, they often rely on restrictive assumptions, such as availability of matched subjects across scanners [42], standardized acquisition protocols across scanners [44], necessity of multi-contrast data from the same session [45], or requiring a large amount of training data [44]. Furthermore, there is still no universal harmonization strategy, and even after harmonization, DL models must still be robustly generalizable to unseen scanners and protocols. For an extensive review of MRI harmonization methods, see [47].

On a different note, the context of rare diseases has specific challenges and limitations. Data availability is extremely limited, which severely restricts the application of ML/DL approaches to these diseases, including both predictive models, such as classifiers, and harmonization methods. For example, in the case of leukodystrophies [48], the brain MRIs are gathered from various clinical centers over a long period, making it infeasible to even create a standardized dataset. Such datasets are not only small in size but also highly heterogeneous in terms of MRI scanners and protocols. These conditions significantly hinder the applicability of conventional ML methods to rare diseases.

Several existing methods address WM abnormality analysis, such as lesion segmentation or volumetric assessments, typically using supervised deep learning models. Many of these approaches require large amounts of voxel-wise, manually annotated data for training [49, 50, 51]. Some methods specifically target

the segmentation of lesions within the brain [52, 11, 53, 54, 55], whereas this study focuses on classifying MRIs based on the WM abnormalities. Additionally, several segmentation models require the availability of multiple MRI sequences (e.g., FLAIR, T1, T2) [56, 38, 19], while the approach presented in this study is designed to operate solely on FLAIR images, enhancing its practicality and ease of deployment across heterogeneous clinical datasets.

Herein, we present HeteroMRI, an approach for classifying brain MRIs based on WM abnormalities while mitigating the heterogeneity effects of the images acquired from multiple scanners and acquisition protocols. In this paper, HeteroMRI is utilized to detect brains with WM abnormalities in FLAIR images through binary classification. HeteroMRI is applicable to multi-scanner and multi-protocol datasets and demonstrates effectiveness in data-limited conditions, providing a flexible and practical solution for both research and clinical applications. The presented method employs MRI intensity clustering, a technique used in the literature for other MRI-related purposes such as brain tissue segmentation [57, 58], brain tumor segmentation [59], and inhomogeneity correction [60]. HeteroMRI is evaluated in various experimental settings to ensure its robustness. Additionally, we apply the method to limited data scenarios in order to assess the performance and applicability of the presented method for rare diseases. In future work, the method is intended to be applied to the task of classifying two WM diseases based on their distinct WM abnormality patterns. The presented method opens new avenues for performing WM abnormality-related analyses on heterogeneous MRI datasets and the large amount of MRI data generated daily in medical centers.

The current paper is structured in the following way: Sec. 2 provides an overview of the methodology used, detailing the data preprocessing steps and the architecture of the DL model employed in this study. Moving on to Sec. 3, the experiments, the datasets used, the experimental settings, the execution of the model, and the evaluation metrics are presented. Following that, Sec. 4 presents the key results, while Sec. 5 discusses the results, highlights the limitations of the method, and introduces the possible future directions. Finally, Sec. 6 provides the conclusion.

2 Methodology

The WM abnormality detection approach presented in this paper uses heterogeneous brain MRI data with various acquisition protocols (multi-protocol) as the input data for a Convolutional Neural Network (CNN). The model is a binary classifier trained to detect patients with WM abnormalities in their brain MRI. The method consists of three main modules explained in the following subsections, namely, MRI preprocessing, Intensity clustering, and DL model. An overview of the methodology is illustrated in Fig. 1.

2.1 MRI preprocessing

For preparing the image data for the analysis, we use our brain MRI preprocessing pipeline, *FlexiMRIprep*[1], that consecutively performs all the requested preprocessing steps/algorithms on all the selected images

[1] https://github.com/ul-mds/FlexiMRIprep

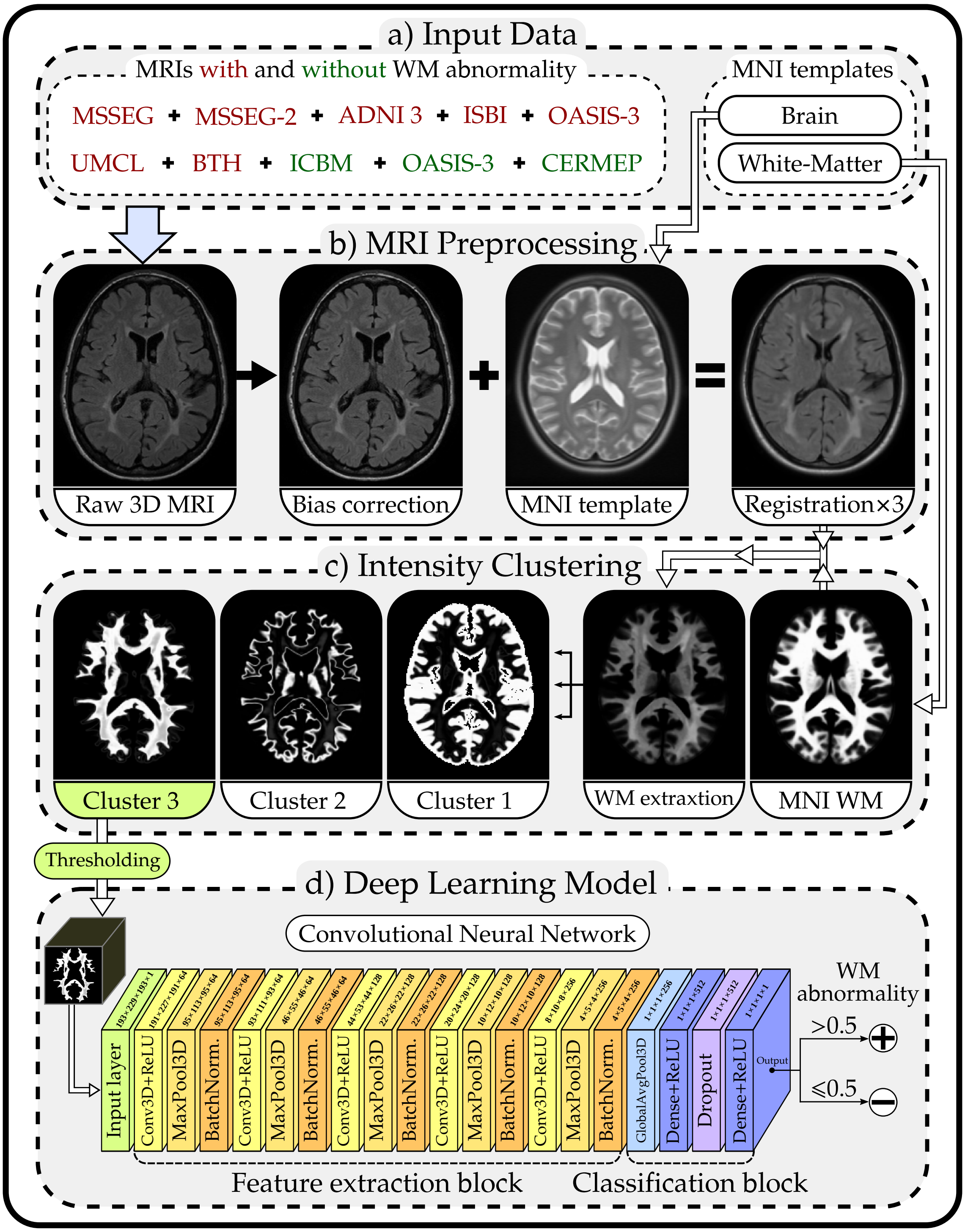

Figure 1: Overview of the methodology: a) Input data: The MRI datasets used for the classification model and MNI brain template [61, 62]. The MRI data with and without WM abnormality are taken from the datasets shown in red and green, respectively. b) MRI preprocessing: The N4 bias field correction method [28] is applied on the FLAIR MRIs (in 3D), and then the MRIs are three times registered (nonlinearly) to the MNI template. c) Intensity clustering: The WM of the brain is extracted, and the WM is clustered into three intensity clusters using RFCM [63] algorithm. d) DL model: Only Cluster 3 of the WM is thresholded and used for a binary classification model with the CNN architecture shown.

automatically. Being the optimal MRI sequence in detecting WM abnormalities, only FLAIR images are used in the analysis in this paper. All selected images have a minimum of 128, 192, and 22 voxels in their first, second, and third dimensions, respectively. All the MRIs are converted to the Neuroimaging Informatics Technology Initiative (NIfTI-1) format using the `dcm2niix` tool (version 1.0.20211006) [64] at this point. The preprocessing steps described below are applied identically to all MRIs from different datasets. Detailed information on the parameters used in each step is reported in the GitHub repository of HeteroMRI[2].

- **Bias field correction**: For correcting the bias field or inhomogeneity issues in the MRIs, we employ the commonly used N4ITK [28] bias field correction method for this purpose. For implementation, the `N4BiasFieldCorrectionImageFilter` class from the `SimpleITK` [65] (version 2.1.1.2) *Python* library with the default parameters is used.
- **Registration**: Registration enables precise spatial mapping and the comparison of anatomical structures among the MRIs. In this process, all the MRIs used for training and testing the AI model are aligned to a standard brain template. Among the available brain templates, we choose the "ICBM 2009c Nonlinear Asymmetric" template [61, 62] (referred to below as the MNI template), which the developers created using the data from the International Consortium for Brain Mapping (ICBM) project [66]. This template was selected due to its high accuracy and the availability of the WM probability map required for our analysis approach. Since there is no dedicated FLAIR template in the MNI template, the T2-weighted template was used due to its proximity to FLAIR. For the registration, the `antsRegistration` tool from the Advanced Normalization Tools (ANTs) [67] (version 2.4.4) is employed. A nonlinear registration is applied three times (with identical parameters) on each image consecutively. This repeated registration aims to achieve a high level of alignment of the MRIs with the template. Mutual information (MI) was calculated between each registered MRI and the MNI template, after each of the three registration steps. As shown in Supplementary Fig. S1, the MI increased after each nonlinear registration step, with mean values improving from 0.62 (1st registration), to 0.73 (2nd registration) and 0.80 (3rd registration). However, the improvement between the second and third steps was relatively small (ΔMI $\approx$ 0.072), indicating diminishing returns. This suggests that three registration steps are sufficient to achieve consistent and reliable alignment. Performing additional iterations may not justify the computational cost and could even introduce unnecessary anatomical distortions, particularly due to the nonlinear nature of the transformations. Multi-pass registration has also been used by others, for example, to address large differences in the initial positions of image pairs [68]. The registered MRIs all have a size of $193 \times 229 \times 193$ voxels with a voxel size of $1 \times 1 \times 1$ mm.

2.2 Intensity clustering

- **WM extraction**: After the brain images are aligned with the MNI template, the WM probability map of the template is used to extract the WM volumes of each brain. Therefore, all the other

[2] `https://github.com/ul-mds/HeteroMRI`

brain tissues are removed. The WM extraction is performed by using the `MultiplyImages` tool from ANTs.

- **WM clustering**: The WM clustering is performed for two essential purposes: 1) to obtain a subgroup of WM volumes that includes significant signs of WM abnormalities, and 2) to reduce the negative effect of the heterogeneous MRI data coming from multiple scanners and protocols. These two points are elaborated on in the following.

A clustering algorithm is used to estimate three intensity clusters from the WM volumes obtained in the previous step. The algorithm groups the WM volumes into three subgroups that share a relatively similar intensity range. We expect that one of the clusters shows more indications of WM abnormalities (if present in the brain) since the abnormalities have higher intensity values in FLAIR images. This specific cluster will be used as the training data for the classifier model. The cluster is a membership function with float values in the range of [0,1] for each voxel. As a result, the impact of the heterogeneous nature of the multi-protocol MRIs is reduced, in a way that the proposed method is robust to the many MRI protocols we used in this work. The decision to use three clusters was based on our empirical observations from a dataset different from those used in this study. Through testing various cluster numbers on different MRIs, we found that three clusters yielded consistently comparable patterns in nearly all MRIs. In other words, the shapes of the clusters in one MRI were generally consistent with those in another MRI. This was also later observed in the data of the current study. Therefore, the choice of three clusters is robust and does not depend on a specific dataset.

Here, we employ a Robust Fuzzy C-Means (RFCM) algorithm [63] for WM intensity clustering. The RFCM algorithm modifies the standard FCM objective function by incorporating a local spatial penalty term, leading to the computation of smoother membership functions. This modification not only improves segmentation performance but also provides a level of noise insensitivity. The RFCM algorithm is implemented using the `fuzzy_cmeans` function available in the *Nighres* (Neuroimaging at high resolution) *Python* package, version 1.4.0 [69].

Upon examining the three WM intensity clusters in MRIs with WM abnormalities, we noted that one of the clusters within each MRI, Cluster 3 in Fig. 1(c), consistently exhibited significant lesion-related features. Therefore, from each MRI, we should take the cluster that looks visually similar to Cluster 3 in Fig. 1(c), but it is not always the cluster number 3. For this purpose, we use the Dice similarity coefficient[70] to compare the three clusters of each MRI with a fixed reference cluster to detect the most similar one. The reference image (available in the GitHub repository of HeteroMRI) is generated by averaging the intended intensity cluster of four MRIs from a clinical dataset. This method detected the right cluster for all the MRIs of this study correctly (i.e., with 100% accuracy) as checked manually.

- **Thresholding**: A thresholding is applied on the selected WM intensity cluster of each MRI. All the intensity values below 0.5 are ignored in order to remove uncertain, low-confidence assignments

and retain only the core voxels that are strongly associated with the cluster. The value 0.5 is chosen experimentally in the design phase of HeteroMRI by using (clinical) datasets different from those used in this study for the task of classifying two WM diseases. The value 0.5 resulted in the highest improvement in the classification accuracy compared to other tested thresholds. The histogram for most MRIs follows the same overall pattern: approximately $50 \pm 5\%$ of intensity values are below 0.2, and around $35 \pm 5\%$ are above 0.8. Supplementary Fig. S4 shows the WM cluster of a sample MRI before and after thresholding along with their normalized histograms (for the 99% upper percentile). Finally, the thresholded clusters from the MRIs (one intensity cluster per MRI) are used as training data for the DL model, as described in the following section.

2.3 Deep learning model

The objective is to train a binary classifier model that detects the brain MRIs that have WM abnormalities. Inspired by [71, 72], we configured a 3D CNN comprising a total of 20 layers, as illustrated in Fig. 1(d). The network has a total of 1,795,905 parameters. The model begins with the input layer, followed by a feature extraction block, and ends with a classification block.

In the feature extraction block, we employ five 3D Convolution (Conv3D) layers with 64, 64, 128, 128, and 256 filters, respectively. Each Conv3D has a 3x3x3 kernel size and employs the Rectified Linear Unit (ReLU) activation function. Subsequently, each Conv3D layer is succeeded by a 3D Max Pooling (MaxPool3D) layer with a stride of (2,2,2) and a pool size of (2,2,2), which downscales the 3D input by half in each dimension. Batch Normalization [73] layers with default parameters follow each MaxPool3D layer.

In the classification block, a 3D Global Average Pooling (GlobalAvgPool3D) layer is followed by a Dense layer with a dimensionality of 512 and with a ReLU activation function. To help prevent overfitting, a Dropout layer with a 30% rate is introduced next. Finally, the output layer performs a binary classification employing a Sigmoid activation function. The binary cross-entropy loss, Adam optimizer [74], and an *Early Stopping* feature (*patience*=40) are employed in the model. In each epoch, the checkpoint feature saves the model if the validation accuracy has improved. In the case of an unchanged validation accuracy, the mode is saved if the validation loss has decreased. The *Python* implementation code of the HeteroMRI method is publicly available[3].

3 Experiments

Different MRI datasets are used along with multiple experimental settings with various conditions to train and evaluate the CNN model for classifying brain MRIs. In the following subsections, the datasets and the experimental settings are elaborated.

[3] https://github.com/ul-mds/HeteroMRI

3.1 Datasets

In this study, we utilized FLAIR images from multiple brain MRI datasets as introduced below. Incorporating a combination of MRIs with a high diversity of acquisition protocols and scanners ensures a robust evaluation of the presented methodology. All the datasets used in this study are either publicly available or are accessible upon request to the respective dataset providers. All the MRIs were visually checked by the authors to exclude images with large artefacts. As presented in Table 1, a total of 11 MRI datasets are utilized.

Table 1: MRI datasets used in this study. M/F: number of male/female subjects; Age: mean±standard deviation (years).

No.	Dataset name/alias	Images[1]		Demographics		Protocols[2]	Availability	Reference
		+	−	M/F	Age			
1	ISBI	19	0	4/15	40.4±9.2	1	Public	[75]
2	UMCL	30	0	7/23	median 39 (2564)	1	Public	[76]
3	MSSEG	52	0	15/37	45.3±10.3	4	AoR[3]	[77]
4	MSSEG-2	40	0	N/A	N/A	10	AoR[3]	[78]
5	BTH	9	0	1/8	29.4±8.9	2	Public	[79]
6	ICBM	0	5	2/3	28.2±7.5	1	AoR[3]	[80]
7	OASIS-3	14	90	50/54	68.4±9.5	4	AoR[3]	[81]
8	ADNI 3	58	0	28/30	76.5±8.6	8	AoR[3]	[82]
9	CERMEP	0	27	14/13	34.9±9.3	1	AoR[3]	[83]
10	WMH	10	0	N/A**	N/A**	5	Public	[84]
11	PPMI	0	10	3/7	52.7±13.2	4	AoR[3]	[85]
	Sum	232	132	-	-	40*	-	-

[1] Number of FLAIR images, with (+) and without (−) WM abnormality
[2] Number of MRI protocols in the used data
[3] Accessible on Request (to the respective dataset provider)
* Sum of unique protocols in the data
** Not available on individual level

The details of each dataset are outlined below:

1. **ISBI**: The International Symposium on Biomedical Imaging (ISBI) in 2015 [75] conducted an MS lesion segmentation challenge using longitudinal MRI data. The dataset comprises imaging data from MS patients, acquired using the same scanner and protocol. We utilize 19 FLAIR images from this dataset. For each patient, the MRI taken at the latest time point is used.

2. **UMCL**: A cohort of MS patients was imaged at the University Medical Center Ljubljana (UMCL) [76]. The images were acquired using the same scanner and protocol. We use 30 3D FLAIR images from this dataset.

3. **MSSEG**: The MSSEG dataset [77] was presented for the MS lesion segmentation challenge during the MICCAI 2016 conference. The dataset contains MRIs of MS patients from four different sites. Each site used different MRI scanners and protocols. We utilize 52 FLAIR images[4] from this dataset.

4. **MSSEG-2**[5]: MSSEG-2 [78] is a challenge for the segmentation of new MS lesions in the brain conducted in the MICCAI 2021 conference. At the time of the current research, only the training

[4] The MSSEG dataset originally contains 53 FLAIR images, but one of them was inadvertently excluded from our analysis.

[5] Data were generated by participating neurologists in the framework of Observatoire Français de la Sclérose en Plaques (OFSEP), the French MS registry [86]. They collect clinical data prospectively in the European Database for MS (EDMUS) software [87]. MRI of patients were provided as part of a care protocol. Nominative data are deleted from MRI before transfer and storage on the Shanoir platform (Sharing NeurOImagingResources, shanoir.org).

data of the dataset is accessible. The images of the training set were acquired at 12 different sites and using 10 different scanners. All the images were acquired at two different time points from each patient. From this dataset, we utilize 40 3D FLAIR images from the second time point.

5. **BTH**: The brain MRI dataset of MS patients from Baghdad Teaching Hospital (BTH) [79] includes MRIs taken at 20 centers with different protocols. We used nine[6] FLAIR images from this dataset, which were taken using two different protocols.

6. **ICBM**[7]: The International Consortium for Brain Mapping (ICBM) [80] has developed a probabilistic atlas and reference system for the human brain for normal adults. The dataset includes 20 3D FLAIR images; however, only five were selected for this study, as our neurology specialist confirmed these to be the only ones free of WM abnormalities. The images were acquired using the same scanner and protocol.

7. **OASIS-3**: The Open Access Series of Imaging Studies (OASIS) project provides publicly available neuroimaging datasets. Among its releases, only OASIS-3 [81] includes FLAIR images, encompassing both cognitively normal individuals and those at various stages of cognitive decline. Approximately 850 subjects in OASIS-3 have FLAIR scans. For this study, we used 104 FLAIR and T2-FLAIR images acquired under four different MRI protocols. From a random subset of 600 images, 90 were selected by two neurology specialists based on the absence of WM abnormalities. The remaining 14 images were selected from the same pool to ensure representation of diverse WM lesion patterns, as confirmed through visual inspection.

8. **ADNI 3**: The Alzheimer's Disease Neuroimaging Initiative[8] (ADNI) provides a rich repository of neuroimaging and clinical data [82]. From ADNI-3, which includes FLAIR scans for over 1,100 subjects, we selected 58 3D FLAIR images specifically based on the diversity of their WM lesion patterns. A neurology specialist verified that these images include a range of lesion types, including multifocal, confluent, and brainstem lesions.

9. **CERMEP**: The CERMEP-IDB-MRXFDG dataset [83] comprises MRI, CT, and [^{18}F]FDG PET image data with BIDS standard of healthy subjects. The dataset has 37 FLAIR images obtained using the same scanner and protocol. As reported in the original study, these images underwent visual review by two neurologists to confirm the absence of any apparent brain abnormalities. However, due to our strict criteria for even minor lesions, our neurologists confirmed only 27 MRIs as free of WM abnormalities for use as control data in our model.

[6]The NIfTI files in this dataset lack orientation information (qform and sform), making it impossible for the registration algorithm to identify the correct orientation of the brain. Additionally, the MRIs are 2D, resulting in around 10 times fewer slices than the pixels in the first and second dimensions, and they all share the same pixel thickness across all three dimensions. Consequently, the brain appears unrealistically short in 3D view. Due to these dataset-specific conditions, we applied two additional preprocessing steps at the beginning for this dataset: 1) added correct orientation information to each file, and 2) edited slice thicknesses in the header of NIfTI files based on slice thickness information provided in the dataset's metadata. However, the height of many images still does not appear realistic and may cause problems for the registration. Therefore, only nine images were used

[7]The ICBM project (Principal Investigator John Mazziotta, M.D., University of California, Los Angeles) is supported by the National Institute of Biomedical Imaging and BioEngineering. ICBM is the result of efforts of co-investigators from UCLA, Montreal Neurologic Institute, University of Texas at San Antonio, and the Institute of Medicine, Juelich/Heinrich Heine University - Germany.

[8]The ADNI was launched in 2003 as a public-private partnership, led by Principal Investigator Michael W. Weiner, MD. The primary goal of ADNI (`https://adni.loni.usc.edu`) has been to test whether serial MRI, positron emission tomography (PET), other biological markers, and clinical and neuropsychological assessment can be combined to measure the progression of Mild Cognitive Impairment (MCI) and early Alzheimer's Disease (AD).

10. **WMH**: The White Matter Hyperintensity (WMH) segmentation challenge dataset [84], introduced at the MICCAI 2017 conference, includes MRI scans from 170 subjects acquired using five different scanners. The MRIs have different loads of WM lesion. In this paper, we used 10 FLAIR images from this dataset. From each scanner, two MRIs were taken (the first training and the first test MRI, and the first two test MRIs from the scanners that were used only as test data).

11. **PPMI**: The Parkinsons Progression Markers Initiative (PPMI) [85] is a large-scale, longitudinal study that offers comprehensive imaging, clinical, and biospecimen data from individuals with Parkinsons disease and healthy controls. For this study, we selected 10 FLAIR images without WM abnormalities, identified by a neurology specialist from approximately 220 healthy control subjects with available FLAIR scans.

From the MRI data explained above, the datasets one to nine (344 images) are used for training and testing the model in the experimental settings explained below, and the datasets 10 and 11 (20 images) are used as holdout sets. Around 36% of these MRIs are 2D, based on our definition that MRIs with 70 or fewer slices are considered 2D. A comprehensive list of the MRI files is available in the GitHub repository of HeteroMRI, providing details for each image, including the subject ID from the original dataset and the acquisition protocol.

3.2 Experimental settings

Various experimental settings have been designed for a robust evaluation of the presented classification approach. An experimental setting means the specification of the data used for training, validating, and testing the CNN model. By employing the datasets explained in Sec. 3.1, FLAIR images from different scanners and acquisition protocols are intentionally combined and used for training and testing the model. The images necessary for each setting are selected randomly from the MRIs available. The number of MRIs with and without WM abnormality is balanced in the training, validation, and test data of all the settings. There are four setting groups, namely A, B, C, and D. In setting A, the data are selected based on datasets, while in the settings B, C, and D, the data are incorporated based on their acquisition protocol. We assigned a protocol name to each of the MRIs based on the scanner name and model, magnetic field strength, and acquisition parameters. The protocol naming convention is explained in the HeteroMRI Github repository. The experimental settings are introduced below:

- **Setting *A***: In setting A, the goal is to evaluate HeteroMRI on a combination of MRIs from different datasets beginning from a relatively large number of data and then decreasing the data gradually. In setting A, there are 19 settings that are run independently. In $A00$, 244 MRIs from nine datasets are used. The data of *each* dataset is split into training (70%), validation (10%), and test (20%) sets. In $A01$, the training data is downsized by approximately 10% while the test set remains the same images as in $A00$. The downsizing process continues up to $A18$, where the training and validation sets together include only four MRIs. The downsizing is performed by removing random MRIs while keeping the maximum possible number of protocols among the data. Across all settings from $A00$ to $A18$, the test set remains identical. Supplementary Table S1 shows the number of MRIs used for training, validation, and test sets from each dataset in the settings $A00$ to $A18$.

- **Setting *B***: In setting B, the goal is to choose the MRIs with the most diversity of protocols while having an equal number of MRIs from each protocol; therefore, the model sees the same number of MRIs per protocol. MRIs from 10 different protocols are incorporated. The test data is selected from all protocols. In $B00$, from each protocol, five MRIs for training, one MRI for validation, and one MRI for the test are used. In the subsequent settings, the training data is reduced. By $B04$, only one MRI per protocol is used for the training set. In Supplementary Table S2, the list of selected protocols and the number of MRIs used for training, validation, and test sets for $B00$ to $B04$ is reported.

- **Setting *C***: In setting C, the goal is to assess the generalizability of HeteroMRI to unseen MRI protocols. In this setting, MRIs from eight different protocols are included. Only data from 3D MRIs are included in this setting due to the reason later explained in Sec. 4.3. MRIs from six protocols are used *only* in training and validation sets, while the other two protocols are *only* used in the test set. In fact, the trained model does not see any data from the protocols used in the test set during training. We consider 10 cases, in each case considering two different protocols for testing the model. The setting C begins with $C00$, which uses 64 MRIs for training and validation. This continues up to $C06$ with only eight MRIs for training and validation. In Table S3, the list of selected protocols and the number of MRIs used for training, validation, and test sets for $C00$ to $C06$ is reported.

- **Setting *D***: In setting D, the goal is to see the effect of the number of MRI protocols on the performance of the model. Beginning from $D00$ and going toward $D03$, more protocols are included in the data used for training and testing the model. At the same time, the total number of data and test set size are kept the same among the settings $D00$ to $D03$ (82 MRIs for training including validation data), therefore it is possible to compare the results of the settings to see the effect of having more protocols in the data. In $D00$, there are MRIs from four protocols. In $D01$ to $D03$, there are MRIs from, respectively, six, eight, and 10 protocols. The test data is selected from all protocols. In Table S4, the list of selected protocols and the number of MRIs used for training, validation, and test sets for $D00$ to $D03$ is reported.

3.3 Model execution

All the MRIs used in this study, introduced in Sec. 3.1, are preprocessed following the procedure elaborated in Sec. 2.1. Next, the intensity clustering procedure is applied to each preprocessed MRI, following the procedure introduced in Sec. 2.2. As a result, a single intensity cluster per MRI is used for training or testing the model. Notably, the 3D intensity clusters obtained from the MRIs serve as the exclusive training data for the model. The model has no exposure to the original MRIs or any form of WM lesion annotation file. For the preprocessing and intensity clustering tasks, we used a machine with Intel(R) Xeon(R) Gold 6240R CPU @ 2.40GHz and 128 GB of RAM. The preprocessing pipeline (introduced in Sec. 2.1) employs a parallelization approach in some of the preprocessing steps to make the procedure faster. The computation time required for preprocessing each MRI depends on multiple factors; nevertheless, the total number of voxels in the 3D MRI plays a more significant role. More specifically, based on our assessments, the number of slices of the MR image highly affects the required computation time. The Supplementary Fig. S2 shows the average time required for preprocessing and intensity clustering of five

sample MRI dimensions.

The CNN model explained in Sec.2.3 is trained and tested on each experimental setting independently. In settings A, B, C, and D, the required number of data is selected (and split into training, validation, and test sets) from all available data 50 independent times (referred to below as "data shuffle"). For each data shuffle, the model is trained and tested 10 times. For training and testing the CNN model, we used a computational server with an AMD Epyc 7352 CPU, 1 TB of CPU RAM, and an NVIDIA A100 GPU (40 GB GPU RAM). The average required time for training the model of each setting is shown in Supplementary Fig. S3. The inference time of the model on a single test data is a few seconds. The inference process can be efficiently performed without the necessity of a GPU.

3.4 Evaluation metrics

To assess the performance of the classification model, we employ five common metrics: accuracy, sensitivity, specificity, F1 score, and precision. Moreover, the area under the receiver operating characteristic curve (AUROC) is reported for selected settings. Additionally, we calculate a cumulative metric called Machine Learning cumulative performance score (*MLcps*) [88]. The *MLcps* combines the pre-computed performance metrics into a single metric that encapsulates the core aspects of all the metrics. The value of *MLcps* is equal to the area of the polygon created by the metrics in a radar plot. We used the *MLcps Python* package version 0.0.6. The *MLcps* metric was originally designed for comparing and identifying the best-performing ML algorithm. However, herein, we utilize *MLcps* to compare the performance of the same model for the different amounts of training data. As we have a fixed number of pre-calculated metrics (accuracy, sensitivity, specificity, F1 score, and precision), we define *MLcps%* as

$$MLcps\% = \frac{MLcps}{MLcps_{max}} \times 100, \tag{1}$$

where *MLcps* is the area of the pentagon in the radar plot and $MLcps_{max}$ is the area of the pentagon when all the five metrics are 100%.

Furthermore, a permutation test was conducted to assess the statistical significance of the models performance. By randomly shuffling the class labels 1,000 times and retraining the model on each shuffled set of data, a distribution of accuracy values under chance conditions was obtained for all the experimental settings.

4 Results

In this section, we present and analyze the results of the experiments to evaluate the performance of the HeteroMRI method. The results are reported separately for the experimental settings A, B, C, and D. Furthermore, the insights into limited data scenarios gained through the experiments are discussed afterwards. The classification metrics are provided in box plot and radar plot formats in Figs. 2, 5, 6, and 7. In all box plots, the triangle marker indicates the mean value, and the whiskers represent $1.5 \times IQR$, where IQR is the interquartile range. In settings A, B, and D, the box plots show the distribution of 500 values

for each setting # corresponding to 50 data shuffles that each has been run 10 times. By "setting #", we mean for example $A00, A01, \ldots, A18$. In setting C, the box plot shows the distribution of 5,000 accuracy values for each setting # since there are 10 cases with different protocols chosen as the test set, as explained in Sec. 3.2. The results of permutation tests are plotted as jittered points in the accuracy plots in Figs. 2, 5, 6, and 7 in part (a). For settings A, B, and D, there are 1,000 permutations, while for setting C, there are 10,000 permutations since there are 10 cases with different protocols chosen as the test set.

The radar plots illustrate the metric values for all the setting #s simultaneously, allowing us to perceive the effect of reducing training data size on each metric. In addition, the radar plots are utilized for calculating the *MLcps* values using Eq. (1). The *MLcps%* values for the settings A, B, and C are reported in the Figs. 2(e), 5(e), and 6(c). The radar plots are plotted using the mean metric values; therefore, no standard deviation is reported for the *MLcps%* values. Evaluation of the presented approach across various experimental settings provides several key insights, offering a detailed understanding of its performance and challenges. The results of each experimental setting are presented below.

4.1 Setting *A*

As shown in Fig. 2, setting $A00$, in which the highest number of MRIs (174 training+26 validation+44 test) were included, demonstrates an average accuracy of 93.2±4.3% in the classification of MRIs. The training and testing data in this setting include, in total, 32 different MRI protocols. This underscores the model's adaptability and robustness in handling a diverse range of imaging protocols. Notably, the effect of reducing the training data on the model's performance is inspected here. In $A07$, where the training data (including validation data) is reduced to 36% of $A00$, the accuracy is 89.2±6.4%. Beginning from setting $A08$, where the training data is 25% of $A00$, the average accuracy and sensitivity have a sharp decrease. With much further decreasing the training data (e.g., $A18$ with only two MRIs as training), the accuracy and sensitivity gradually decrease to low values as expected, while specificity tends to remain relatively high. In relatively limited data settings (particularly from $A11$ to $A18$), the boxplots indicate that accuracy values have significant fluctuations across the range.

Sample raw MRIs, the registered MRIs, the obtained WM intensity clusters, and the model's label predictions are provided here for a better insight into the data used for testing the model. Figs. 3 and 4 depict samples of MRIs without and with WM abnormality, respectively. Below each raw MRI, the three-times registered MRI, and the obtained WM intensity cluster (thresholded) are illustrated. Additionally, the model's prediction for the presence of WM abnormalities in setting $A00$ is reported. Each figure includes three true predictions and three false predictions. It is important to note that the shown WM intensity cluster and the registered MRI slice show the same location of the brain (the middle slice). However, these two slices do not directly correspond to the raw MRI slice. This is because the non-linear registration process results in the deformation of the brain. As a result, finding the exact corresponding slices in the raw MRI and the registered one is impractical. Here, the middle slice of the registered MRI is illustrated. The raw MRI slices shown here are selected based on their visual similarity to the corresponding registered

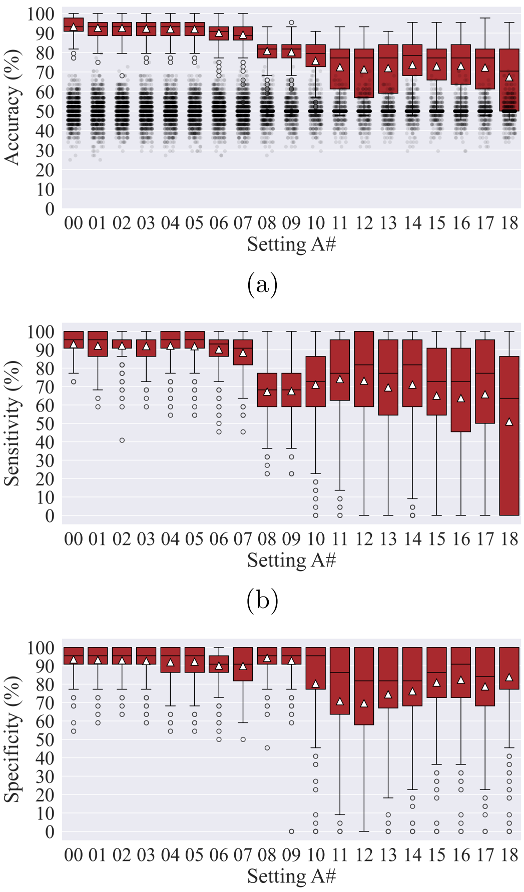

(a)

(b)

(c)

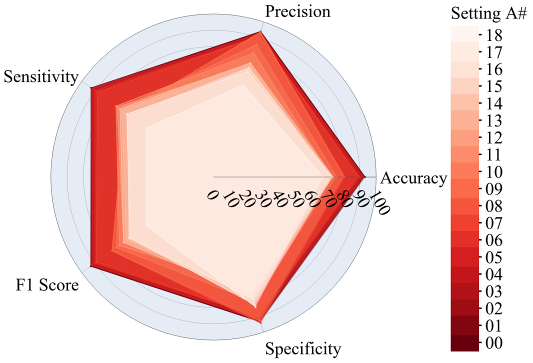

(d)

**Setting**	*A*00	*A*01	*A*02	*A*03	*A*04
MLcps%	85.2	84.0	83.9	83.2	82.6
**Setting**	*A*05	*A*06	*A*07	*A*08	*A*09
MLcps%	82.8	78.8	76.9	63.0	62.0
**Setting**	*A*10	*A*11	*A*12	*A*13	*A*14
MLcps%	53.8	48.3	44.6	44.9	47.3
**Setting**	*A*15	*A*16	*A*17	*A*18	
MLcps%	44.9	44.7	43.4	33.9	

(e)

Figure 2: Classification results of settings *A*00 to *A*18: (a) accuracy, (b) sensitivity, (c) specificity, (d) radar plot of five classification metrics for different setting #s, and (e) *MLcps%* (a cumulative performance score) in % for each setting #. The triangle marker indicates the mean value, and the whiskers represent $1.5 \times IQR$. In each setting # (i.e. *A*00, *A*01, ..., *A*18), the training set size is sequentially reduced by approximately 10% relative to the previous setting, as detailed in Supplementary Table S1. In (a), the jittered points represent the results of 1,000 permutation tests for each setting #. Average accuracy starts at 93.2±4.3% for *A*00, where training includes 200 MRIs, and decreases to 89.2±6.4% in *A*07, with 72 training data, after which it drops with further reductions in training data. A similar trend is observed in the *MLcps%*.

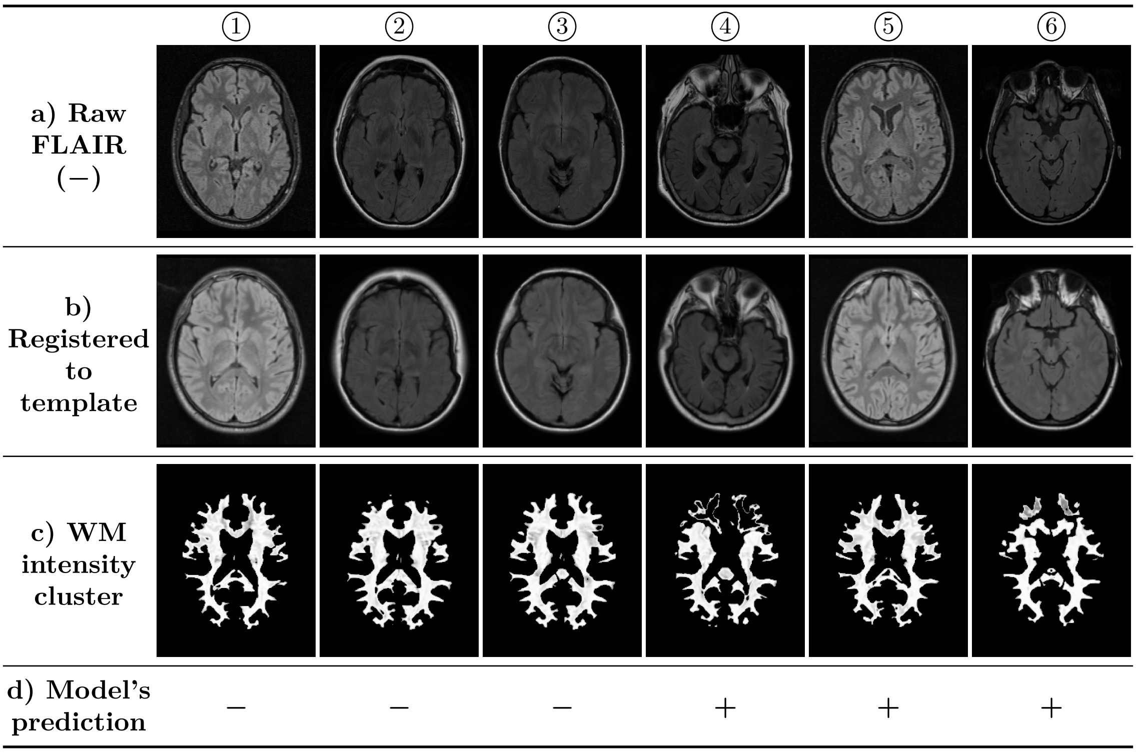

Figure 3: Example of six MRIs (① to ⑥) *without* WM abnormality. a) Raw FLAIR images, b) the MRI three times registered to the MNI template (the middle slice shown), c) the obtained intensity clusters (thresholded) used for testing the model, and d) the prediction of the model in setting $A00$ for the presence of WM abnormalities (+: with WM abnormality, −: without WM abnormality). This figure shows three cases of true negative and three cases of false positive. The WM intensity cluster slice corresponds to the registered MRI slice, but not exactly to the raw MRI slice due to deformations from non-linear registrations. MRIs source: ①, ⑤:[83], ②, ③, ④:[81], ⑥:[80].

slices. The slices of the thresholded WM cluster are presented here solely to illustrate the input provided to the DL model for label prediction.

By investigating the possible reasons for the false predictions in setting $A00$, certain aspects became apparent. In some MRIs, the registration process has not been successful in correctly aligning the brain to the brain template. In such cases, the brain regions are not located in the correct locations after the three-times registration in the MRI preprocessing phase. Therefore, in the WM extraction step, wrong parts of the brain are extracted as WM. This mostly results in a false prediction by the model, especially if the MRI is, in fact, free of WM abnormalities. Examples of incorrect registration are MRIs ④, ⑤, ⑥, ⑩, and ⑫ shown in Figs. 3 and 4. These registration errors are identified by comparing the slices of the registered MRIs with the corresponding slice from the MNI template slice shown in Fig. 1(b). In both cases, the middle slice of the MRI is displayed. In an accurate registration, the brain regions in the registered slice align approximately with those in the template. We noticed that some of the MRIs with incorrect registration are 2D MRIs (such as MRIs ④ and ⑫). The low number of slices in 2D MRIs appears to be a contributing factor to registration problems in some cases. Considering that MRI registration is usually a challenging problem, other factors are also likely to contribute to incorrect registrations; however, they have not been investigated in this study. We refrained from excluding the MRIs with erroneous registration from the study, as our goal was to evaluate HeteroMRI as a fully automatic

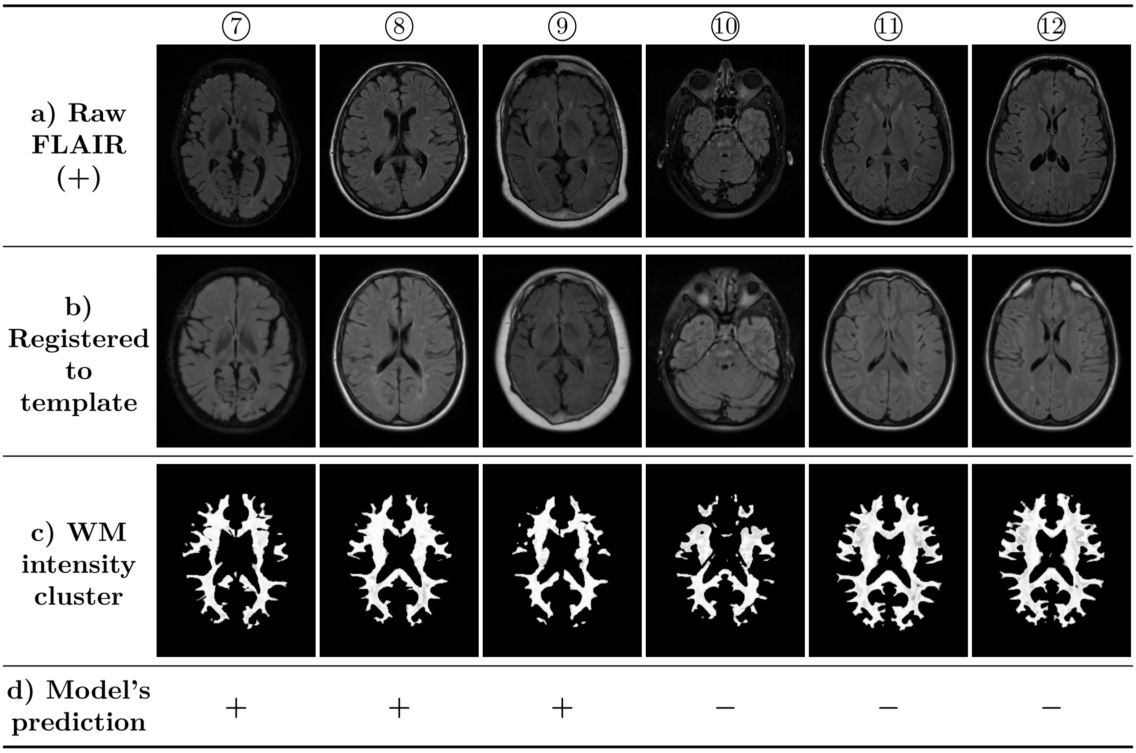

Figure 4: Example of six MRIs (⑦ to ⑫) *with* WM abnormality. a) raw FLAIR images, b) the MRI three times registered to the MNI template (the middle slice shown), c) the obtained intensity clusters (thresholded) used for testing the model, and d) the prediction of the model in setting $A00$ for the presence of WM abnormalities (+: with WM abnormality, −: without WM abnormality). This figure shows three cases of true positive and three cases of false negative. The WM intensity cluster slice corresponds to the registered MRI slice, but not exactly to the raw MRI slice due to deformations from non-linear registrations. MRIs source: ⑦,⑩:[82], ⑧:[79], ⑨:[81], ⑪,⑫:[75].

method without user interference.

4.2 Setting B

The setting B, which uses an equal number of MRIs from each MRI protocol, is designed to make the prediction task more challenging for the model. Setting $B00$ has an average accuracy of 89.3±9.1%, as shown in Fig. 5. In terms of the amount of training data, setting $A07$ is the closest match to $B00$. While $B00$ includes 50 training and 10 validation data, $A07$ has a comparable setup with 52 training and 20 validation data. However, setting B is a more challenging scenario than setting A because the model sees an equal number of MRIs from each MRI protocol without being biased by a higher number of images from some protocols. Despite this challenge, the accuracy of $B00$ is almost equal to that of $A07$, which is 89.2±6.4%. This shows the high independence of the presented MRI classification approach on the acquisition protocol of the FLAIR images. With further decrease in the training data, in settings $B01$ to $B04$, all metrics show a gradual decrease in value.

4.3 Setting C

The evaluation of setting C is of more importance since it is very close to the real-world use of such a model, as it evaluates the generalizability of HeteroMRI to MRIs from unseen protocols. In this setting, the MRI protocols of the test set are not present in the training data. It resembles a situation in which a clinical center has heterogeneous MRI data and wants to train a classification model with them. Then the model is supposed to classify new MRIs brought by new patients from other centers, acquired most probably with MRI protocols different from those in the training data.

It is noteworthy that in setting C, only 3D MRIs (with 192 or more slices) are included in training and test data, as discussed in the next paragraph. Setting $C00$, as reported in Fig. 6, shows an average accuracy of 92.6±10.6% with 64 MRIs used for training (including validation), which proves the generalizability of the trained model to unseen MRI scanners and protocols. By reducing the data to 46, in $C02$, the model shows an accuracy of 85.7±16.8%. By further decreasing the data to 28 or less, the model's accuracy drops to the chance level (around 50%) in $C04$ to $C06$.

Initially, we used both 3D and 2D MRIs for setting C. However, the model showed a lack of robustness when its generalizability to different protocols was evaluated (i.e., by varying the protocols considered as test data). As we suspected the 2D MRIs (with 70 or fewer slices) as a source of the model's poor performance, we redesigned the setting C to include *only* 3D MRIs. As a result, the performance significantly improved, as reported in the results for setting C. It is important to emphasize that the test data were not fixed for the two versions of setting C, as the model was tested with multiple data shuffles for each setting, with the data being split again in each shuffle. However, from a certain perspective, the decision to exclude 2D MRIs could be interpreted as a form of overfitting to the data characteristics. Additionally, to further investigate the role of 2D MRIs, we also designed a separate setting with *only* 2D MRIs. In this

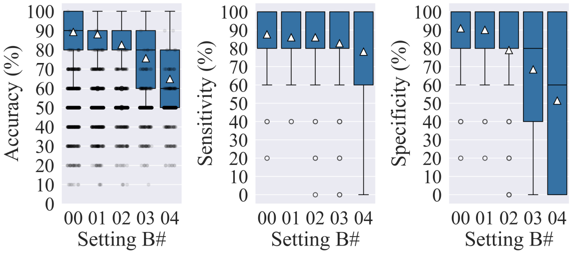

(a) (b) (c)

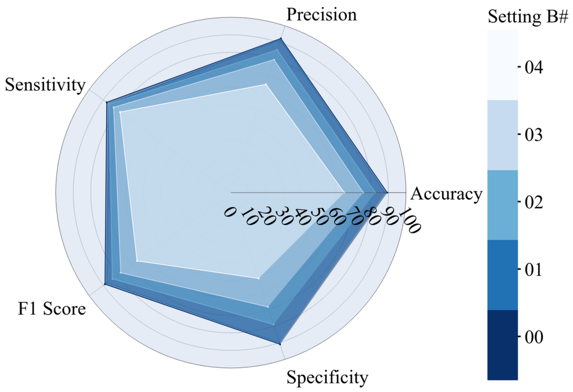

(d)

**Setting**	$B00$	$B01$	$B02$	$B03$	$B04$
MLcps%	77.9	75.5	65.1	53.4	36.0

(e)

Figure 5: Classification results of settings $B00$ to $B04$: (a) accuracy, (b) sensitivity, (c) specificity, (d) radar plot of five classification metrics for different setting #s, and (e) *MLcps%* (a cumulative performance score) in % for each setting #. The triangle marker indicates the mean value, and the whiskers represent $1.5 \times IQR$. In each setting # (i.e. $B00$, $B01$, $\ldots$, $B04$), the training set size is sequentially reduced relative to the previous setting, as detailed in Supplementary Table S2. In (a), the jittered points represent the results of 1,000 permutation tests for each setting #. In setting B, an equal number from 10 different MRI protocols is used for the model. Average accuracy starts at 89.3±9.1% for $B00$, where training includes 60 MRIs, and decreases to 82.5±14.2% in $B02$, with 40 training data, after which it decreases gradually with further reductions in training data. A similar trend is observed in the *MLcps%*.

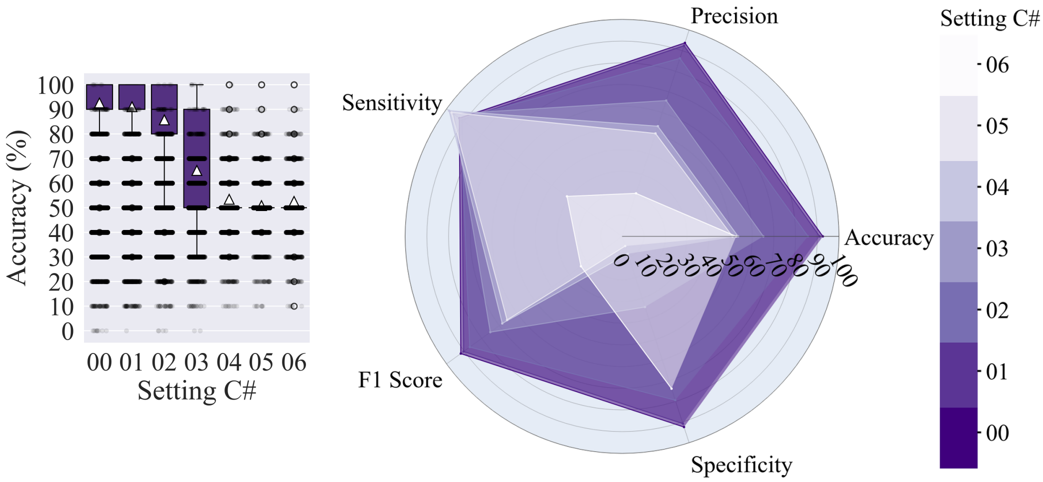

(a) (b)

Setting	Accuracy	Sensitivity	Specificity	*MLcps%*
$C00$	92.58±10.57	92.73±16.94	92.43±15.25	84.02
$C01$	91.09±11.54	91.40±18.54	90.77±17.27	80.93
$C02$	85.74±16.81	92.07±18.46	79.41±32.43	70.49
$C03$	65.14±20.96	96.05±13.51	34.24±45.42	39.07
$C04$	53.50±11.65	98.55±9.38	8.45±26.54	26.42
$C05$	50.93±5.98	97.18±15.83	4.67±20.50	23.55
$C06$	52.61±9.70	31.40±45.12	73.81±42.90	11.53

(c)

Figure 6: Classification results of settings $C00$ to $C06$: (a) accuracy, (b) radar plot of five classification metrics for different setting #s, and (c) four metric values for each setting # (in %, reported as mean±standard deviation). The triangle marker indicates the mean value, and the whiskers represent $1.5 \times IQR$. In each setting # (i.e. $C00$, $C01$, ..., $C06$), the training set size is sequentially reduced relative to the previous setting, as detailed in Supplementary Table S3. In (a), the jittered points represent the results of 1,0000 permutation tests for each setting #. In setting C, the MRI protocols of the test set are unseen by the model during training. Average accuracy starts at 92.6±10.6% for $C00$, where training includes 64 MRIs, and reduces to 85.7±16.8% in $C02$, with 46 training data, after which it drops sharply with further reductions in training data. A similar trend is observed in the *MLcps%*.

case, the model showed notably poor robustness.

4.4 Setting *D*

Setting D, in which the number of MRI protocols among the data was increased in each setting # while maintaining the same data size (82 MRIs for training), shows the slight negative effect of having higher numbers of protocols, as observed by the overall decrease in accuracy, sensitivity, and specificity (Fig.7). In $D00$ with 4 protocols, the accuracy is 94.80±6.79% while in $D03$ with 10 protocols, the model classifies the MRIs with an accuracy of 90.31±6.36%.

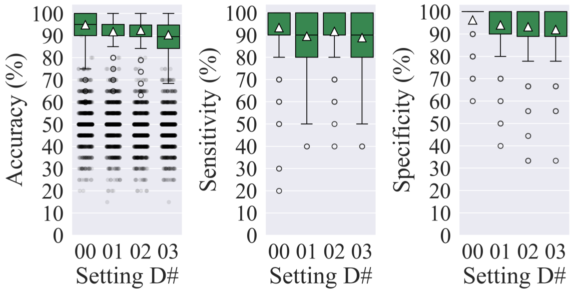

(a) (b) (c)

Figure 7: Classification results of settings $D00$ to $D03$: (a) accuracy, (b) sensitivity, and (c) specificity. The triangle marker indicates the mean value, and the whiskers represent $1.5 \times IQR$. In each setting # (i.e. $D00$, $D01$, . . ., $D03$), the number of MRI protocols is sequentially increased relative to the previous setting while maintaining equal training set size (82 MRIs), as detailed in Supplementary Table S4. In (a), the jittered points represent the results of 1,000 permutation tests for each setting #. Average accuracy starts at 94.8±6.8% for $D00$, where training data includes four different MRI protocols, and ends at 90.3±6.3% for $D03$, with 10 MRI protocols in training data.

4.5 Limited data scenarios

A more detailed examination of the impact of reducing training data is presented in Table 2. By comparing settings A, B, and C, a rough correlation can be concluded between the number of MRIs in the training data and the performance of the model for the classification task of this study, regardless of the experimental setting. When the training data (including validation) consists of 60 to 72 MRIs (as in $A07$ and $B00$), an accuracy of 89% is expected. By having 40 to 46 MRIs in the training set (as in $A09, B02$, and $C02$), the accuracy falls roughly within the range of 80% to 86%. Further reducing the training data to the 20 to 36 range (as in $A12, B03$, and $C03$) is associated with a rough accuracy of 76% or less and an F1 score of 78% or less. In this last scenario, the model's performance cannot be considered fully reliable, as the results show a high degree of variability across different runs.

4.6 Comparison with related methods

To further evaluate the performance of HeteroMRI, we applied it on a hold-out set of data and compared the performance with three lesion segmentation methods: DeepWMH [53], WHITE-Net [54], and the Lesion Prediction Algorithm from the Lesion Segmentation Tool (LST-LPA) [55]. These methods are not designed for direct binary classification of MRIs. To enable a comparison, we post-processed their segmentation outputs by binarizing the masks through 3D connected components analysis (using the `ConnectedComponentImageFilter` class from `SimpleITK` [65] *Python* library): if at least one WM lesion consisting of *five* or more 3D-connected voxels was detected, the corresponding MRI scan was labeled as positive. The comparison was conducted using datasets 10 and 11, introduced in Sec. 3.1. The test set includes 20 MRIs in total, with balanced labels and images acquired from nine different MRI protocols. These datasets serve as holdout sets for HeteroMRI and were not used during training or testing in any of the experimental settings. For HeteroMRI, we employed the 500 models trained in setting $A00$. The final prediction label for each test scan was determined by majority vote across these models. The DeepWMH,

Table 2: Average performance results of selected experimental settings (in %, reported as mean±standard deviation). The table shows the effect of reducing training data on the model's performance in settings A, B, and C. For these settings, three cases are reported respectively: 1) with the highest number of training data, 2) with the borderline number of training data after which the performance drops, and 3) with the number of training data that results in relatively low performance. Setting D shows the effect of increasing the number of MRI protocols for the same number of MRI data. AUROC: the area under the receiver operating characteristic curve.

Setting	Data size[1]		Protocols[2]		Accuracy	Sensitivity	Specificity	F1 score	AUROC
	Train†	Test	Train†	Test					
A00	200	44	31	14	93.24±4.29	93.05±6.34	93.44±7.38	93.24±4.25	96.76±3.09
A07	72	44	17±1	14	89.16±6.39	88.41±11.17	89.91±9.48	88.87±7.00	94.89±4.18
A09	42	44	9±1	14	80.17±6.69	67.59±13.29	92.74±14.78	76.83±8.55	91.18±4.30
A12	20	44	6±1	14	71.41±13.76	73.14±30.01	69.67±34.80	68.35±23.32	91.91±3.71
B00	60	10	10	10	89.32±9.14	87.64±14.31	91.00±14.25	88.88±9.75	95.11±7.24
B02	40	10	10	10	82.54±14.17	86.00±15.46	79.08±30.20	83.65±12.09	91.98±8.68
B03	30	10	10	10	75.64±16.36	82.76±19.25	68.52±38.97	77.71±14.63	90.26±8.69
C00	64	10	8	2	92.58±10.57	92.73±16.94	92.43±15.25	91.91±13.25	99.07±1.06
C02	46	10	8	2	85.74±16.81	92.07±18.46	79.41±32.43	86.80±16.27	97.43±2.12
C03	36	10	8	2	65.14±20.96	96.05±13.51	34.24±45.42	75.09±14.84	93.34±3.33
D00	82	20	4	4	94.80±6.79	93.38±11.91	96.22±7.69	94.38±8.27	99.38±1.86
D03	82	20	10	10	90.30±6.33	88.82±11.42	91.95±10.28	90.36±6.85	96.11±5.03

[1] Number of MRIs used in the training and test sets
[2] Number of MRI protocols present in the training and test data
† Including the validation data

WHITE-Net, and LST-LPA are pre-trained, ready-to-use models that do not require retraining. All of the methods require only a FLAIR image per subject. Table 3 reports the accuracy, sensitivity, and specificity for all methods. Based on the results, HeteroMRI outperforms DeepWMH, WHITE-Net, and LST-LPA in accuracy and specificity, while the other methods have a higher sensitivity.

Table 3: Performance of HeteroMRI (setting A00) as a binary classifier for detecting brain MRIs with WM abnormalities in a hold-out set, in comparison to three other related methods.

Method	Accuracy	Sensitivity	Specificity
**HeteroMRI** (This work)	**70%**	90%	**50%**
**DeepWMH** [53]	55%	**100%**	10%
**WHITE-Net** [54]	55%	**100%**	10%
**LST-LPA** [55]	60%	**100%**	20%

5 Discussion

This study presented HeteroMRI, a DL framework designed for robust binary classification of WM abnormalities across heterogeneous FLAIR MRI datasets. The results show that HeteroMRI is capable of performing the classification while being highly robust to scanner and protocol variabilities. The method

is also adaptable to standardized MRI datasets acquired using a uniform scanner and protocol. The experimental results across settings AD and the limited-data scenarios offer several insights into the method's robustness, generalizability, and its limitations under varying data constraints.

In Setting A, where training data included all available protocols, HeteroMRI achieved high classification performance. Even by reducing the training data to 72 MRIs, the accuracy remained at 89±6%. This suggests that the model learns effectively from the heterogeneous data, with only a gradual decline in performance as data scarcity increases. In Setting B, where each protocol was equally represented, HeteroMRI maintained comparable accuracy, confirming that the model is highly independent of protocols. In Setting C, which tested generalization to unseen MRI protocols, HeteroMRI continued to perform robustly, provided the training data included a sufficient number of high-resolution (3D) MRIs. This emphasizes the importance of data quality in achieving protocol-invariant performance. Finally, setting D showed the impact of increasing protocol diversity while keeping data volume fixed. A gradual decrease in accuracy was observed with a growing number of MRI protocols, decreasing from 94.8% in D00 (4 protocols) to 90.3% in D03 (10 protocols).

In comparison of the performance on a hold-out set with three segmentation-based methods: DeepWMH [53], WHITE-Net [54], and LST-LPA [55], HeteroMRI outperformed all three methods in terms of both accuracy and specificity, showing a more reliable ability to avoid false positives despite the fact that DeepWMH and WHITE-Net claim to be robust to scanner and protocol variability. This comparison was conducted on a highly heterogeneous hold-out set comprising 20 MRIs from nine different acquisition protocols. Moreover, this comparison result is obtained in a condition in favor of the segmentation methods since we applied a threshold requiring a minimum of five 3D-connected voxels for determining the presence of a lesion in the segmentation outputs. This conservative criterion helps filter out small, potentially false predictions and helps segmentation-based models by reducing false positives. Nevertheless, the segmentation-based models demonstrated higher sensitivity, which can be attributed to their design: these methods are tailored to detect even subtle or small lesion patterns, making them more prone to false positives in borderline cases. These findings generally support HeteroMRI as a method that is highly independent of scanner and protocol variability, generalizes relatively well to unseen protocols, and retains performance under data-limited conditions to some extent.

Despite the promising results of the presented method, it has several limitations to be considered. HeteroMRI faces a challenge with the registration problem with certain MRIs, leading to a false prediction. Registration problems are a well-known issue in MRI analysis. For addressing this challenge with HeteroMRI, a more elaborate registration strategy may reduce the misalignments, or an automatic method may be developed to warn about significant deformations during registration, which can be a sign of wrong registration. These improvements are future work directions for further improving the method. Notably, 2D MRIs were identified as one of the factors that can contribute to registration issues, strongly suggesting the use of 3D MRIs with HeteroMRI. Further factors that cause an incorrect registration were not investigated in this study. Another limitation of HeteroMRI is the high GPU memory requirement, which

is not easily available in every computing server. For each specific use case, one can evaluate the method's performance by downscaling the dimension of input images (and therefore reducing the dimensionality of the CNN) to decrease the required GPU memory. Additionally, the design of HeteroMRI can be upgraded to a multi-channel format, enabling the integration of multiple MRI sequences per subject for enhanced analysis. Moreover, the performance of the method could be studied in case of unbalanced training data. The current classification task of HeteroMRI may be less interesting to apply in clinical practice compared to, for example, segmentation or volumetric measurement methods. However, this paper is supposed to present the HeteroMRI method and evaluate its performance on heterogeneous data. In future work, the methodology is planned to be employed for the disease-specific classification of MRIs based on WM abnormalities by learning from subtle lesion patterns.

6 Conclusion

In this study, we introduced HeteroMRI, a novel approach for robust classification of brain MRIs based on WM abnormalities, specifically designed to handle heterogeneous MRI data acquired from diverse scanners and acquisition protocols. HeteroMRI achieved high accuracy in detecting MRIs with WM abnormalities, even in scenarios with relatively limited data. Furthermore, the method proved to be relatively more generalizable to unseen MRI protocols compared to segmentation-based methods. There is room for enhancing HeteroMRI's performance by improving the registration accuracy or preventing wrong predictions by detecting the erroneous registrations automatically. Our future research will focus on applying this approach to differentiate between unspecific and disease-associated WM lesions, as well as to classify rare demyelinating diseases against their differential diagnoses.

7 Availability of Supporting Source Code and Requirements

Project name: HeteroMRI v1.0

Project home page: https://github.com/ul-mds/HeteroMRI

Operating system(s): Linux-based OS (Ubuntu recommended)

Programming language: Python

Other requirements: TensorFlow v2.x, NVIDIA GPU with CUDA support

License: GNU GPL version 3

8 Data Availability

All the datasets used in this study are either publicly available or are accessible upon request to the respective dataset providers as referenced in Table 1. Snapshots of our code and other data further supporting this work are archived in Software Heritage [89]. In addition, machine learning algorithms have been deposited in the DOME Registry [90].

9 Abbreviations

AI: Artificial Intelligence; ANTs: Advanced Normalization Tools; AUROC: Area Under the Receiver Operating Characteristic Curve; BTH: Baghdad Teaching Hospital; CSF: CerebroSpinal Fluid; CT: Computed Tomography; CNN: Convolutional Neural Network; DL: Deep Learning; TE: Echo Time; FLAIR: Fluid-Attenuated Inversion Recovery; FCM: Fuzzy C-Means; GM: Gray Matter; ICBM: International Consortium for Brain Mapping; TI: Inversion Time; ML: Machine Learning; $MLcps$: Machine Learning Cumulative Performance Score; MRI: Magnetic Resonance Imaging; MNI: Montreal Neurological Institute; MS: Multiple Sclerosis; NifTI: Neuroimaging Informatics Technology Initiative; ReLU: Rectified Linear Unit; TR: Repetition Time; RFCM: Robust Fuzzy C-Means; 3D: three-dimensional; WM: White Matter.

10 Competing Interests

The authors declare that they have no competing interests

11 Authors' Contributions

M.A. (Methodology, Formal Analysis, Software), N.SH. (Conceptualization, Methodology, Writing Original Draft), P.L.B. (Methodology), N.SC. (Supervision), J.L. (Supervision, Data Curation), C.C.B. (Supervision), W.K. (Supervision, Data Curation, Project Investigation, Funding Acquisition), T.K. (Supervision, Project Investigation, Funding Acquisition). All authors contributed to reviewing and editing the manuscript.

12 Funding

The authors acknowledge the financial support by the Federal Ministry of Health of Germany in project LeukoExpert (grant no. ZMVI1-2520DAT94), the State Ministry for Education and Research of Germany in the project Tag-White (grant no. 100602109), and the Federal Ministry of Research, Technology and Space of Germany and by Sächsische Staatsministerium für Wissenschaft, Kultur und Tourismus in the programme Center of Excellence for AI-research "Center for Scalable Data Analytics and Artificial Intelligence Dresden/Leipzig", project identification number: ScaDS.AI. Supported by the Open Access Publication Fund of Leipzig University.

13 Acknowledgments

The authors sincerely thank Dr. Sina Sadeghi for his insightful comments on the manuscript. The authors are grateful to the Center for Information Services and High-Performance Computing [Zentrum für Informationsdienste und Hochleistungsrechnen (ZIH)] at TU Dresden for providing its facilities for high-throughput calculations. Data were provided in part by OASIS Longitudinal Multimodal Neuroimaging: Principal Investigators: T. Benzinger, D. Marcus, J. Morris; NIH P30 AG066444, P50 AG00561, P30

NS09857781, P01 AG026276, P01 AG003991, R01 AG043434, UL1 TR000448, R01 EB009352. AV-45 doses were provided by Avid Radiopharmaceuticals, a wholly owned subsidiary of Eli Lilly. Part of the Data collection and sharing for the Alzheimer's Disease Neuroimaging Initiative (ADNI) is funded by the National Institute on Aging (National Institutes of Health Grant U19 AG024904). The grantee organization is the Northern California Institute for Research and Education. In the past, ADNI has also received funding from the National Institute of Biomedical Imaging and Bioengineering, the Canadian Institutes of Health Research, and private sector contributions through the Foundation for the National Institutes of Health (FNIH) including generous contributions from the following: AbbVie, Alzheimers Association; Alzheimers Drug Discovery Foundation; Araclon Biotech; BioClinica, Inc.; Biogen; Bristol-Myers Squibb Company; CereSpir, Inc.; Cogstate; Eisai Inc.; Elan Pharmaceuticals, Inc.; Eli Lilly and Company; EuroImmun; F. Hoffmann-La Roche Ltd and its affiliated company Genentech, Inc.; Fujirebio; GE Healthcare; IXICO Ltd.; Janssen Alzheimer Immunotherapy Research & Development, LLC.; Johnson & Johnson Pharmaceutical Research &Development LLC.; Lumosity; Lundbeck; Merck & Co., Inc.; Meso Scale Diagnostics, LLC.; NeuroRx Research; Neurotrack Technologies; Novartis Pharmaceuticals Corporation; Pfizer Inc.; Piramal Imaging; Servier; Takeda Pharmaceutical Company; and Transition Therapeutics. Part of the data collection and sharing for this project was provided by the International Consortium for Brain Mapping (ICBM; Principal Investigator: John Mazziotta, MD, PhD). ICBM funding was provided by the National Institute of Biomedical Imaging and BioEngineering. ICBM data are disseminated by the Laboratory of Neuro Imaging at the University of Southern California. Part of the data was provided in collaboration with The Observatoire Français de la Sclérose en Plaques (OFSEP), which is supported by a grant provided by the French State and handled by the "Agence Nationale de la Recherche," within the framework of the "Investments for the Future" program, under the reference ANR-10-COHO-002, by the Eugène Devic EDMUS Foundation against multiple sclerosis and by the ARSEP Foundation. Part of the data used in the preparation of this article was obtained on 2025-04-05 from the Parkinsons Progression Markers Initiative (PPMI) database (www.ppmi-info.org/access-dataspecimens/download-data), RRID:SCR_006431. For up-to-date information on the study, visit www.ppmi-info.org. PPMI—a public-private partnership—is funded by the Michael J. Fox Foundation for Parkinsons Research, and funding partners, including 4D Pharma, Abbvie, AcureX, Allergan, Amathus Therapeutics, Aligning Science Across Parkinson's, AskBio, Avid Radiopharmaceuticals, BIAL, BioArctic, Biogen, Biohaven, BioLegend, BlueRock Therapeutics, Bristol-Myers Squibb, Calico Labs, Capsida Biotherapeutics, Celgene, Cerevel Therapeutics, Coave Therapeutics, DaCapo Brainscience, Denali, Edmond J. Safra Foundation, Eli Lilly, Gain Therapeutics, GE HealthCare, Genentech, GSK, Golub Capital, Handl Therapeutics, Insitro, Jazz Pharmaceuticals, Johnson & Johnson Innovative Medicine, Lundbeck, Merck, Meso Scale Discovery, Mission Therapeutics, Neurocrine Biosciences, Neuron23, Neuropore, Pfizer, Piramal, Prevail Therapeutics, Roche, Sanofi, Servier, Sun Pharma Advanced Research Company, Takeda, Teva, UCB, Vanqua Bio, Verily, Voyager Therapeutics, the Weston Family Foundation and Yumanity Therapeutics.

14 Additional Files

Supplementary Table S1. The experimental setting A and details of the MRI data used.

Supplementary Table S2. The experimental setting B and details of the MRI data used.

Supplementary Table S3. The experimental setting C and details of the MRI data used.

Supplementary Table S4. The experimental setting D and details of the MRI data used.

Supplementary Fig. S1. Mutual information metric between each MRI and the MNI template after each registration.

Supplementary Fig. S2. Average required time for preprocessing and intensity clustering of five sample MRI dimensions.

Supplementary Fig. S3. Average required time for training the CNN model of each experimental setting.

Supplementary Fig. S4. The WM intensity cluster of a sample MRI (a) before and (b) after applying a threshold value of 0.5. The normalized histograms of their 99% upper percentile are shown in (c) and (d), respectively.

References

[1] T. Yousaf, G. Dervenoulas, & M. Politis (2018) *Chapter two - advances in MRI methodology*, M. Politis (Ed.) *Imaging in Movement Disorders: Imaging Methodology and Applications in Parkinson's Disease*, vol. 141 of *International Review of Neurobiology*, pp. 31–76, Academic Press

[2] F. Agosta, S. Galantucci, & M. Filippi (2017) *Advanced magnetic resonance imaging of neurodegenerative diseases*, Neurological Sciences, **38(1)**:pp. 41–51

[3] M. Filippi, P. Preziosa, B. L. Banwell, F. Barkhof, O. Ciccarelli, N. De Stefano, J. J. G. Geurts, F. Paul, D. S. Reich, A. T. Toosy, A. Traboulsee, M. P. Wattjes, T. A. Yousry, A. Gass, C. Lubetzki, B. G. Weinshenker, & M. A. Rocca (2019) *Assessment of lesions on magnetic resonance imaging in multiple sclerosis: practical guidelines*, Brain, **142(7)**:pp. 1858–1875

[4] C. Domínguez-Fernández, J. Egiguren-Ortiz, J. Razquin, M. Gómez-Galán, L. De las Heras-García, E. Paredes-Rodríguez, E. Astigarraga, C. Miguélez, & G. Barreda-Gómez (2023) *Review of technological challenges in personalised medicine and early diagnosis of neurodegenerative disorders*, International Journal of Molecular Sciences, **24(4)**

[5] A. S. Panayides, A. Amini, N. D. Filipovic, A. Sharma, S. A. Tsaftaris, A. Young, D. Foran, N. Do, S. Golemati, T. Kurc, K. Huang, K. S. Nikita, B. P. Veasey, M. Zervakis, J. H. Saltz, & C. S. Pattichis (2020) *AI in medical imaging informatics: Current challenges and future directions*, IEEE Journal of Biomedical and Health Informatics, **24(7)**:pp. 1837–1857

[6] M. L. Giger (2018) *Machine learning in medical imaging*, Journal of the American College of Radiology, **15(3, Part B)**:pp. 512–520, data Science: Big Data Machine Learning and Artificial Intelligence

[7] N. Garg, M. S. Choudhry, & R. M. Bodade (2023) *A review on Alzheimers disease classification from normal controls and mild cognitive impairment using structural MR images*, Journal of Neuroscience Methods, **384**:p. 109745

[8] J. Wen, E. Thibeau-Sutre, M. Diaz-Melo, J. Samper-González, A. Routier, S. Bottani, D. Dormont, S. Durrleman, N. Burgos, & O. Colliot (2020) *Convolutional neural networks for classification of Alzheimer's disease: Overview and reproducible evaluation*, Medical Image Analysis, **63**:p. 101694

[9] A. Kursad Poyraz, S. Dogan, E. Akbal, & T. Tuncer (2022) *Automated brain disease classification using exemplar deep features*, Biomedical Signal Processing and Control, **73**:p. 103448

[10] D. García-Lorenzo, S. Francis, S. Narayanan, D. L. Arnold, & D. Louis Collins (2013) *Review of automatic segmentation methods of multiple sclerosis white matter lesions on conventional magnetic resonance imaging*, Medical Image Analysis, **17(1)**:pp. 1–18

[11] O. Cetin, V. Seymen, & U. Sakoglu (2020) *Multiple sclerosis lesion detection in multimodal MRI using simple clustering-based segmentation and classification*, Informatics in Medicine Unlocked, **20**:p. 100409

[12] J. Amin, M. Sharif, M. Yasmin, & S. L. Fernandes (2020) *A distinctive approach in brain tumor detection and classification using MRI*, Pattern Recognition Letters, **139**:pp. 118–127

[13] M. A. Naser & M. J. Deen (2020) *Brain tumor segmentation and grading of lower-grade glioma using deep learning in MRI images*, Computers in Biology and Medicine, **121**:p. 103758

[14] S. Zhang, S. Xu, L. Tan, H. Wang, & J. Meng (2021) *Stroke lesion detection and analysis in MRI images based on deep learning*, Journal of Healthcare Engineering, **2021**:p. 5524769

[15] Y. Kabir, M. Dojat, B. Scherrer, F. Forbes, & C. Garbay (2007) *Multimodal MRI segmentation of ischemic stroke lesions*, *2007 29th Annual International Conference of the IEEE Engineering in Medicine and Biology Society*, pp. 1595–1598

[16] H. Peng, W. Gong, C. F. Beckmann, A. Vedaldi, & S. M. Smith (2021) *Accurate brain age prediction with lightweight deep neural networks*, Medical Image Analysis, **68**:p. 101871

[17] H. Sajedi & N. Pardakhti (2019) *Age prediction based on brain MRI image: A survey*, Journal of Medical Systems, **43(8)**:p. 279

[18] J. West, J. B. M. Warntjes, & P. Lundberg (2012) *Novel whole brain segmentation and volume estimation using quantitative MRI*, European Radiology, **22(5)**:pp. 998–1007

[19] S. Valverde, A. Oliver, E. Roura, S. González-Villà, D. Pareto, J. C. Vilanova, L. Ramió-Torrentà, Àlex Rovira, & X. Lladó (2017) *Automated tissue segmentation of MR brain images in the presence of white matter lesions*, Medical Image Analysis, **35**:pp. 446–457

[20] N. Andrade, F. A. Faria, & F. A. M. Cappabianco (2018) *A practical review on medical image registration: From rigid to deep learning based approaches*, *2018 31st SIBGRAPI Conference on Graphics, Patterns and Images (SIBGRAPI)*, pp. 463–470

[21] J. Kleesiek, G. Urban, A. Hubert, D. Schwarz, K. Maier-Hein, M. Bendszus, & A. Biller (2016) *Deep MRI brain extraction: A 3D convolutional neural network for skull stripping*, NeuroImage, **129**:pp. 460–469

[22] P. Kalavathi & V. B. S. Prasath (2016) *Methods on skull stripping of MRI head scan images—a review*, Journal of Digital Imaging, **29(3)**:pp. 365–379

[23] A. Kaur & G. Dong (2023) *A complete review on image denoising techniques for medical images*, Neural Processing Letters, **55(6)**:pp. 7807–7850

[24] J. Mohan, V. Krishnaveni, & Y. Guo (2014) *A survey on the magnetic resonance image denoising methods*, Biomedical Signal Processing and Control, **9**:pp. 56–69

[25] J. V. Manjón, J. Carbonell-Caballero, J. J. Lull, G. García-Martí, L. Martí-Bonmatí, & M. Robles (2008) *MRI denoising using non-local means*, Medical Image Analysis, **12(4)**:pp. 514–523

[26] M. Shah, Y. Xiao, N. Subbanna, S. Francis, D. L. Arnold, D. L. Collins, & T. Arbel (2011) *Evaluating intensity normalization on MRIs of human brain with multiple sclerosis*, Medical Image Analysis, **15(2)**:pp. 267–282

[27] C. Loizou, M. Pantziaris, I. Seimenis, & C. Pattichis (2009) *Brain MR image normalization in texture analysis of multiple sclerosis*, *2009 9th International Conference on Information Technology and Applications in Biomedicine*, pp. 1–5

[28] N. J. Tustison, B. B. Avants, P. A. Cook, Y. Zheng, A. Egan, P. A. Yushkevich, & J. C. Gee (2010) *N4ITK: Improved N3 bias correction*, IEEE Transactions on Medical Imaging, **29(6)**:pp. 1310–1320

[29] M. Joliot & B. Mazoyer (1993) *Three-dimensional segmentation and interpolation of magnetic resonance brain images*, IEEE Transactions on Medical Imaging, **12(2)**:pp. 269–277

[30] R. Shinohara, J. Oh, G. Nair, P. Calabresi, C. Davatzikos, J. Doshi, R. Henry, G. Kim, K. Linn, N. Papinutto, D. Pelletier, D. Pham, D. Reich, W. Rooney, S. Roy, W. Stern, S. Tummala, F. Yousuf, A. Zhu, N. Sicotte, R. Bakshi, & the NAIMS Cooperative (2017) *Volumetric analysis from a harmonized multisite brain MRI study of a single subject with multiple sclerosis*, American Journal of Neuroradiology, **38(8)**:pp. 1501–1509

[31] J.-P. Fortin, N. Cullen, Y. I. Sheline, W. D. Taylor, I. Aselcioglu, P. A. Cook, P. Adams, C. Cooper, M. Fava, P. J. McGrath, M. McInnis, M. L. Phillips, M. H. Trivedi, M. M. Weissman, & R. T. Shinohara (2018) *Harmonization of cortical thickness measurements across scanners and sites*, NeuroImage, **167**:pp. 104–120

[32] J.-P. Fortin, D. Parker, B. Tunç, T. Watanabe, M. A. Elliott, K. Ruparel, D. R. Roalf, T. D. Satterthwaite, R. C. Gur, R. E. Gur, R. T. Schultz, R. Verma, & R. T. Shinohara (2017) *Harmonization of multi-site diffusion tensor imaging data*, NeuroImage, **161**:pp. 149–170

[33] J.-P. Fortin, E. M. Sweeney, J. Muschelli, C. M. Crainiceanu, & R. T. Shinohara (2016) *Removing inter-subject technical variability in magnetic resonance imaging studies*, NeuroImage, **132**:pp. 198–212

[34] C. Marzi, M. Giannelli, A. Barucci, C. Tessa, M. Mascalchi, & S. Diciotti (2024) *Efficacy of MRI data harmonization in the age of machine learning: a multicenter study across 36 datasets*, Scientific Data, **11(1)**:p. 115

[35] R. Pomponio, G. Erus, M. Habes, J. Doshi, D. Srinivasan, E. Mamourian, V. Bashyam, I. M. Nasrallah, T. D. Satterthwaite, Y. Fan, L. J. Launer, C. L. Masters, P. Maruff, C. Zhuo, H. Völzke, S. C.

Johnson, J. Fripp, N. Koutsouleris, D. H. Wolf, R. Gur, R. Gur, J. Morris, M. S. Albert, H. J. Grabe, S. M. Resnick, R. N. Bryan, D. A. Wolk, R. T. Shinohara, H. Shou, & C. Davatzikos (2020) *Harmonization of large MRI datasets for the analysis of brain imaging patterns throughout the lifespan*, NeuroImage, **208**:p. 116450

[36] J. Radua, E. Vieta, R. Shinohara, P. Kochunov, Y. Quidé, M. J. Green, C. S. Weickert, T. Weickert, J. Bruggemann, T. Kircher, I. Nenadi, M. J. Cairns, M. Seal, U. Schall, F. Henskens, J. M. Fullerton, B. Mowry, C. Pantelis, R. Lenroot, V. Cropley, C. Loughland, R. Scott, D. Wolf, T. D. Satterthwaite, Y. Tan, K. Sim, F. Piras, G. Spalletta, N. Banaj, E. Pomarol-Clotet, A. Solanes, A. Albajes-Eizagirre, E. J. Canales-Rodríguez, S. Sarro, A. Di Giorgio, A. Bertolino, M. Stäblein, V. Oertel, C. Knöchel, S. Borgwardt, S. du Plessis, J.-Y. Yun, J. S. Kwon, U. Dannlowski, T. Hahn, D. Grotegerd, C. Alloza, C. Arango, J. Janssen, C. Díaz-Caneja, W. Jiang, V. Calhoun, S. Ehrlich, K. Yang, N. G. Cascella, Y. Takayanagi, A. Sawa, A. Tomyshev, I. Lebedeva, V. Kaleda, M. Kirschner, C. Hoschl, D. Tomecek, A. Skoch, T. van Amelsvoort, G. Bakker, A. James, A. Preda, A. Weideman, D. J. Stein, F. Howells, A. Uhlmann, H. Temmingh, C. López-Jaramillo, A. Díaz-Zuluaga, L. Fortea, E. Martinez-Heras, E. Solana, S. Llufriu, N. Jahanshad, P. Thompson, J. Turner, T. van Erp, D. Glahn, G. Pearlson, E. Hong, A. Krug, V. Carr, P. Tooney, G. Cooper, P. Rasser, P. Michie, S. Catts, R. Gur, R. Gur, F. Yang, F. Fan, J. Chen, H. Guo, S. Tan, Z. Wang, H. Xiang, F. Piras, F. Assogna, R. Salvador, P. McKenna, A. Bonvino, M. King, S. Kaiser, D. Nguyen, & J. Pineda-Zapata (2020) *Increased power by harmonizing structural MRI site differences with the ComBat batch adjustment method in ENIGMA*, NeuroImage, **218**:p. 116956

[37] R. Kushol, P. Parnianpour, A. H. Wilman, S. Kalra, & Y.-H. Yang (2023) *Effects of MRI scanner manufacturers in classification tasks with deep learning models*, Scientific Reports, **13(1)**:p. 16791

[38] A. P. De Rosa, M. Benedetto, S. Tagliaferri, F. Bardozzo, A. D'Ambrosio, A. Bisecco, A. Gallo, M. Cirillo, R. Tagliaferri, & F. Esposito (2024) *Consensus of algorithms for lesion segmentation in brain MRI studies of multiple sclerosis*, Scientific Reports, **14(1)**:p. 21348

[39] T. J. Littlejohns, J. Holliday, L. M. Gibson, S. Garratt, N. Oesingmann, F. Alfaro-Almagro, J. D. Bell, C. Boultwood, R. Collins, M. C. Conroy, N. Crabtree, N. Doherty, A. F. Frangi, N. C. Harvey, P. Leeson, K. L. Miller, S. Neubauer, S. E. Petersen, J. Sellors, S. Sheard, S. M. Smith, C. L. M. Sudlow, P. M. Matthews, & N. E. Allen (2020) *The UK Biobank imaging enhancement of 100,000 participants: rationale, data collection, management and future directions*, Nature Communications, **11(1)**:p. 2624

[40] G. Mårtensson, D. Ferreira, T. Granberg, L. Cavallin, K. Oppedal, A. Padovani, I. Rektorova, L. Bonanni, M. Pardini, M. G. Kramberger, J.-P. Taylor, J. Hort, J. Snædal, J. Kulisevsky, F. Blanc, A. Antonini, P. Mecocci, B. Vellas, M. Tsolaki, I. Kłoszewska, H. Soininen, S. Lovestone, A. Simmons, D. Aarsland, & E. Westman (2020) *The reliability of a deep learning model in clinical out-of-distribution MRI data: A multicohort study*, Med Image Anal, **66**:p. 101714

[41] L. G. Nyúl & J. K. Udupa (1999) *On standardizing the MR image intensity scale*, Magnetic Resonance in Medicine, **42(6)**:pp. 1072–1081

[42] M. E. Torbati, D. S. Minhas, C. M. Laymon, P. Maillard, J. D. Wilson, C.-L. Chen, C. M. Crainiceanu, C. S. DeCarli, S. J. Hwang, & D. L. Tudorascu (2023) *MISPEL: A supervised deep learning harmonization method for multi-scanner neuroimaging data*, Medical Image Analysis, **89**:p. 102926

[43] B. E. Dewey, L. Zuo, A. Carass, Y. He, Y. Liu, E. M. Mowry, S. Newsome, J. Oh, P. A. Calabresi, & J. L. Prince (2020) *A disentangled latent space for cross-site MRI harmonization*, A. L. Martel, P. Abolmaesumi, D. Stoyanov, D. Mateus, M. A. Zuluaga, S. K. Zhou, D. Racoceanu, & L. Joskowicz (Eds.) *Medical Image Computing and Computer Assisted Intervention – MICCAI 2020*, pp. 720–729, Springer International Publishing, Cham

[44] S. Liu & P.-T. Yap (2024) *Learning multi-site harmonization of magnetic resonance images without traveling human phantoms*, Communications Engineering, **3(1)**:p. 6

[45] L. Zuo, B. E. Dewey, Y. Liu, Y. He, S. D. Newsome, E. M. Mowry, S. M. Resnick, J. L. Prince, & A. Carass (2021) *Unsupervised MR harmonization by learning disentangled representations using information bottleneck theory*, NeuroImage, **243**:p. 118569

[46] F. Hu, A. Lucas, A. A. Chen, K. Coleman, H. Horng, R. W. Ng, N. J. Tustison, K. A. Davis, H. Shou, M. Li, R. T. Shinohara, & T. A. D. N. Initiative (2023) *Deepcombat: A statistically motivated, hyperparameter-robust, deep learning approach to harmonization of neuroimaging data*, bioRxiv

[47] F. Hu, A. A. Chen, H. Horng, V. Bashyam, C. Davatzikos, A. Alexander-Bloch, M. Li, H. Shou, T. D. Satterthwaite, M. Yu, & R. T. Shinohara (2023) *Image harmonization: A review of statistical and deep learning methods for removing batch effects and evaluation metrics for effective harmonization*, NeuroImage, **274**:p. 120125

[48] L. L. Resende, A. R. B. de Paiva, F. Kok, C. da Costa Leite, & L. T. Lucato (2019) *Adult leukodystrophies: a step-by-step diagnostic approach*, Radiographics, **39(1)**:pp. 153–168

[49] W. Zhu, H. Huang, Y. Zhou, F. Shi, H. Shen, R. Chen, R. Hua, W. Wang, S. Xu, & X. Luo (2022) *Automatic segmentation of white matter hyperintensities in routine clinical brain mri by 2D VB-Net: A large-scale study*, Frontiers in Aging Neuroscience, **Volume 14 - 2022**

[50] S. Mu, W. Lu, G. Yu, L. Zheng, & J. Qiu (2024) *Deep learning-based grading of white matter hyperintensities enables identification of potential markers in multi-sequence mri data*, Computer Methods and Programs in Biomedicine, **243**:p. 107904

[51] M. Kuwabara, F. Ikawa, S. Nakazawa, S. Koshino, D. Ishii, H. Kondo, T. Hara, Y. Maeda, R. Sato, T. Kaneko, S. Maeyama, Y. Shimahara, & N. Horie (2024) *Artificial intelligence for volumetric measurement of cerebral white matter hyperintensities on thick-slice fluid-attenuated inversion recovery (FLAIR) magnetic resonance images from multiple centers*, Scientific Reports, **14(1)**:p. 10104

[52] R. Guerrero, C. Qin, O. Oktay, C. Bowles, L. Chen, R. Joules, R. Wolz, M. Valdés-Hernández, D. Dickie, J. Wardlaw, & D. Rueckert (2018) *White matter hyperintensity and stroke lesion segmentation and differentiation using convolutional neural networks*, NeuroImage: Clinical, **17**:pp. 918–934

[53] C. Liu, Z. Zhuo, L. Qu, Y. Jin, T. Hua, J. Xu, G. Tan, Y. Li, Y. Duan, T. Wang, Z. Zhang, Y. Zhang, R. Chen, P. Yu, P. Zhang, Y. Shi, J. Zhang, D. Tian, R. Li, X. Zhang, F. Shi, Y. Wang, J. Jiang, A. Carass, Y. Liu, & C. Ye (2024) *DeepWMH: A deep learning tool for accurate white matter hyper-*

intensity segmentation without requiring manual annotations for training, Science Bulletin, **69(7)**:pp. 872–875

[54] C. Cathala, F. Kherif, J.-P. Thiran, A. Bussy, & B. Draganski (2025) *WHITE-Net : White matter hyperintensities tissue extraction using deep learning network*, medRxiv

[55] P. Schmidt (2017) *Bayesian inference for structured additive regression models for large-scale problems with applications to medical imaging*, Ph.D. thesis

[56] P. Tran, U. Thoprakarn, E. Gourieux, C. L. dos Santos, E. Cavedo, N. Guizard, F. Cotton, P. Krolak-Salmon, C. Delmaire, D. Heidelberg, N. Pyatigorskaya, S. Ströer, D. Dormont, J.-B. Martini, & M. Chupin (2022) *Automatic segmentation of white matter hyperintensities: validation and comparison with state-of-the-art methods on both multiple sclerosis and elderly subjects*, NeuroImage: Clinical, **33**:p. 102940

[57] X. Tu, J. Gao, C. Zhu, J.-Z. Cheng, Z. Ma, X. Dai, & M. Xie (2016) *MR image segmentation and bias field estimation based on coherent local intensity clustering with total variation regularization*, Medical & Biological Engineering & Computing, **54(12)**:pp. 1807–1818

[58] D. Kumar, R. K. Agrawal, & P. Kumar (2022) *Bias-corrected intuitionistic fuzzy c-means with spatial neighborhood information approach for human brain MRI image segmentation*, IEEE Transactions on Fuzzy Systems, **30(3)**:pp. 687–700

[59] A. Khosravanian, M. Rahmanimanesh, P. Keshavarzi, & S. Mozaffari (2021) *Fast level set method for glioma brain tumor segmentation based on superpixel fuzzy clustering and lattice boltzmann method*, Computer Methods and Programs in Biomedicine, **198**:p. 105809

[60] L. Szilágyi, S. M. Szilágyi, B. Benyó, & Z. Benyó (2011) *Intensity inhomogeneity compensation and segmentation of MR brain images using hybrid c-means clustering models*, Biomedical Signal Processing and Control, **6(1)**:pp. 3–12, biomedical signal processing(Extended selected papers from the 7th IFAC Symposium on Modelling and Control in Biomedical Systems(MCBMS'09))

[61] V. Fonov, A. Evans, R. McKinstry, C. Almli, & D. Collins (2009) *Unbiased nonlinear average age-appropriate brain templates from birth to adulthood*, NeuroImage, **47**:p. S102, organization for Human Brain Mapping 2009 Annual Meeting

[62] V. Fonov, A. C. Evans, K. Botteron, C. R. Almli, R. C. McKinstry, & D. L. Collins (2011) *Unbiased average age-appropriate atlases for pediatric studies*, NeuroImage, **54(1)**:pp. 313–327

[63] D. L. Pham (2001) *Spatial models for fuzzy clustering*, Computer Vision and Image Understanding, **84(2)**:pp. 285–297

[64] X. Li, P. S. Morgan, J. Ashburner, J. Smith, & C. Rorden (2016) *The first step for neuroimaging data analysis: DICOM to NIfTI conversion*, Journal of Neuroscience Methods, **264**:pp. 47–56

[65] B. Lowekamp, D. Chen, L. Ibanez, & D. Blezek (2013) *The design of SimpleITK*, Frontiers in Neuroinformatics, **7**:p. 45

[66] J. C. Mazziotta, A. W. Toga, A. Evans, P. Fox, & J. Lancaster (1995) *A probabilistic atlas of the human brain: Theory and rationale for its development: The International Consortium for Brain Mapping (ICBM)*, NeuroImage, **2(2, Part A)**:pp. 89–101

[67] B. B. Avants, N. Tustison, G. Song, et al. (2009) *Advanced normalization tools (ANTs)*, Insight j, **2(365)**:pp. 1–35

[68] S. Yang, Z. Yang, K. Fischer, K. Zhong, J. Stadler, F. Godenschweger, J. Steiner, H.-J. Heinze, H.-G. Bernstein, B. Bogerts, C. Mawrin, D. Reutens, O. Speck, & M. Walter (2013) *Integration of ultra-high field MRI and histology for connectome based research of brain disorders*, Frontiers in Neuroanatomy, **7**

[69] J. M. Huntenburg, C. J. Steele, & P.-L. Bazin (2018) *Nighres: processing tools for high-resolution neuroimaging*, GigaScience, **7(7)**:p. giy082

[70] L. R. Dice (1945) *Measures of the amount of ecologic association between species*, Ecology, **26(3)**:pp. 297–302

[71] H. Zunair, A. Rahman, N. Mohammed, & J. P. Cohen (2020) *Uniformizing techniques to process CT scans with 3D CNNs for tuberculosis prediction*, I. Rekik, E. Adeli, S. H. Park, & M. d. C. Valdés Hernández (Eds.) *Predictive Intelligence in Medicine*, pp. 156–168, Springer International Publishing, Cham

[72] D. Maturana & S. Scherer (2015) *VoxNet: A 3D convolutional neural network for real-time object recognition*, *2015 IEEE/RSJ International Conference on Intelligent Robots and Systems (IROS)*, pp. 922–928

[73] S. Ioffe & C. Szegedy (2015) *Batch normalization: Accelerating deep network training by reducing internal covariate shift*, F. Bach & D. Blei (Eds.) *Proceedings of the 32nd International Conference on Machine Learning*, vol. 37 of *Proceedings of Machine Learning Research*, pp. 448–456, PMLR, Lille, France

[74] D. P. Kingma & J. Ba (2017) *Adam: A method for stochastic optimization*, arXiv preprint arXiv:1412.6980

[75] A. Carass, S. Roy, A. Jog, J. L. Cuzzocreo, E. Magrath, A. Gherman, J. Button, J. Nguyen, F. Prados, C. H. Sudre, M. Jorge Cardoso, N. Cawley, O. Ciccarelli, C. A. Wheeler-Kingshott, S. Ourselin, L. Catanese, H. Deshpande, P. Maurel, O. Commowick, C. Barillot, X. Tomas-Fernandez, S. K. Warfield, S. Vaidya, A. Chunduru, R. Muthuganapathy, G. Krishnamurthi, A. Jesson, T. Arbel, O. Maier, H. Handels, L. O. Iheme, D. Unay, S. Jain, D. M. Sima, D. Smeets, M. Ghafoorian, B. Platel, A. Birenbaum, H. Greenspan, P.-L. Bazin, P. A. Calabresi, C. M. Crainiceanu, L. M. Ellingsen, D. S. Reich, J. L. Prince, & D. L. Pham (2017) *Longitudinal multiple sclerosis lesion segmentation: Resource and challenge*, NeuroImage, **148**:pp. 77–102

[76] Ž. Lesjak, A. Galimzianova, A. Koren, M. Lukin, F. Pernuš, B. Likar, & Ž. Špiclin (2018) *A novel public MR image dataset of multiple sclerosis patients with lesion segmentations based on multi-rater consensus*, Neuroinformatics, **16**:pp. 51–63

[77] O. Commowick, A. Istace, M. Kain, B. Laurent, F. Leray, M. Simon, S. C. Pop, P. Girard, R. Améli, J.-C. Ferré, A. Kerbrat, T. Tourdias, F. Cervenansky, T. Glatard, J. Beaumont, S. Doyle, F. Forbes, J. Knight, A. Khademi, A. Mahbod, C. Wang, R. McKinley, F. Wagner, J. Muschelli, E. Sweeney, E. Roura, X. Lladó, M. M. Santos, W. P. Santos, A. G. Silva-Filho, X. Tomas-Fernandez, H. Urien,

I. Bloch, S. Valverde, M. Cabezas, F. J. Vera-Olmos, N. Malpica, C. Guttmann, S. Vukusic, G. Edan, M. Dojat, M. Styner, S. K. Warfield, F. Cotton, & C. Barillot (2018) *Objective evaluation of multiple sclerosis lesion segmentation using a data management and processing infrastructure*, Scientific Reports, **8(1)**:p. 13650

[78] O. Commowick, F. Cervenansky, F. Cotton, & M. Dojat (Eds.) (2021) *MSSEG-2 challenge proceedings: Multiple sclerosis new lesions segmentation challenge using a data management and processing infrastructure*, Strasbourg, France

[79] A. M. Muslim, S. Mashohor, G. A. Gawwam, R. Mahmud, M. binti Hanafi, O. Alnuaimi, R. Josephine, & A. D. Almutairi (2022) *Brain MRI dataset of multiple sclerosis with consensus manual lesion segmentation and patient meta information*, Data in Brief, **42**:p. 108139

[80] R. Kötter, J. Mazziotta, A. Toga, A. Evans, P. Fox, J. Lancaster, K. Zilles, R. Woods, T. Paus, G. Simpson, B. Pike, C. Holmes, L. Collins, P. Thompson, D. MacDonald, M. Iacoboni, T. Schormann, K. Amunts, N. Palomero-Gallagher, S. Geyer, L. Parsons, K. Narr, N. Kabani, G. L. Goualher, D. Boomsma, T. Cannon, R. Kawashima, & B. Mazoyer (2001) *A probabilistic atlas and reference system for the human brain: International Consortium for Brain Mapping (ICBM)*, Philosophical Transactions of the Royal Society of London. Series B: Biological Sciences, **356(1412)**:pp. 1293–1322

[81] P. J. LaMontagne, T. L. Benzinger, J. C. Morris, S. Keefe, R. Hornbeck, C. Xiong, E. Grant, J. Hassenstab, K. Moulder, A. G. Vlassenko, M. E. Raichle, C. Cruchaga, & D. Marcus (2019) *OASIS-3: Longitudinal neuroimaging, clinical, and cognitive dataset for normal aging and alzheimer disease*, medRxiv

[82] C. R. Jack Jr., M. A. Bernstein, N. C. Fox, P. Thompson, G. Alexander, D. Harvey, B. Borowski, P. J. Britson, J. L. Whitwell, C. Ward, A. M. Dale, J. P. Felmlee, J. L. Gunter, D. L. Hill, R. Killiany, N. Schuff, S. Fox-Bosetti, C. Lin, C. Studholme, C. S. DeCarli, G. Krueger, H. A. Ward, G. J. Metzger, K. T. Scott, R. Mallozzi, D. Blezek, J. Levy, J. P. Debbins, A. S. Fleisher, M. Albert, R. Green, G. Bartzokis, G. Glover, J. Mugler, & M. W. Weiner (2008) *The Alzheimer's disease neuroimaging initiative (ADNI): MRI methods*, Journal of Magnetic Resonance Imaging, **27(4)**:pp. 685–691

[83] I. Mérida, J. Jung, S. Bouvard, D. Le Bars, S. Lancelot, F. Lavenne, C. Bouillot, J. Redouté, A. Hammers, & N. Costes (2021) *CERMEP-IDB-MRXFDG: a database of 37 normal adult human brain [^{18}F]FDG PET, T1 and FLAIR MRI, and CT images available for research*, EJNMMI Research, **11(1)**:p. 91

[84] H. J. Kuijf, J. M. Biesbroek, J. De Bresser, R. Heinen, S. Andermatt, M. Bento, M. Berseth, M. Belyaev, M. J. Cardoso, A. Casamitjana, D. L. Collins, M. Dadar, A. Georgiou, M. Ghafoorian, D. Jin, A. Khademi, J. Knight, H. Li, X. Lladó, M. Luna, Q. Mahmood, R. McKinley, A. Mehrtash, S. Ourselin, B.-Y. Park, H. Park, S. H. Park, S. Pezold, E. Puybareau, L. Rittner, C. H. Sudre, S. Valverde, V. Vilaplana, R. Wiest, Y. Xu, Z. Xu, G. Zeng, J. Zhang, G. Zheng, C. Chen, W. van der Flier, F. Barkhof, M. A. Viergever, & G. J. Biessels (2019) *Standardized assessment of automatic segmentation of white matter hyperintensities and results of the wmh segmentation challenge*, IEEE Transactions on Medical Imaging, **38(11)**:pp. 2556–2568

[85] K. Marek, D. Jennings, S. Lasch, A. Siderowf, C. Tanner, T. Simuni, C. Coffey, K. Kieburtz, E. Flagg, S. Chowdhury, W. Poewe, B. Mollenhauer, P.-E. Klinik, T. Sherer, M. Frasier, C. Meunier, A. Rudolph, C. Casaceli, J. Seibyl, S. Mendick, N. Schuff, Y. Zhang, A. Toga, K. Crawford, A. Ansbach, P. De Blasio, M. Piovella, J. Trojanowski, L. Shaw, A. Singleton, K. Hawkins, J. Eberling, D. Brooks, D. Russell, L. Leary, S. Factor, B. Sommerfeld, P. Hogarth, E. Pighetti, K. Williams, D. Standaert, S. Guthrie, R. Hauser, H. Delgado, J. Jankovic, C. Hunter, M. Stern, B. Tran, J. Leverenz, M. Baca, S. Frank, C.-A. Thomas, I. Richard, C. Deeley, L. Rees, F. Sprenger, E. Lang, H. Shill, S. Obradov, H. Fernandez, A. Winters, D. Berg, K. Gauss, D. Galasko, D. Fontaine, Z. Mari, M. Gerstenhaber, D. Brooks, S. Malloy, P. Barone, K. Longo, T. Comery, B. Ravina, I. Grachev, K. Gallagher, M. Collins, K. L. Widnell, S. Ostrowizki, P. Fontoura, T. Ho, J. Luthman, M. van der Brug, A. D. Reith, & P. Taylor (2011) *The parkinson progression marker initiative (ppmi)*, Progress in Neurobiology, **95(4)**:pp. 629–635, biological Markers for Neurodegenerative Diseases

[86] S. Vukusic, R. Casey, F. Rollot, B. Brochet, J. Pelletier, D.-A. Laplaud, J. D. Sèze, F. Cotton, T. Moreau, B. Stankoff, B. Fontaine, F. Guillemin, M. Debouverie, & M. Clanet (2020) *Observatoire Français de la Sclérose en Plaques (OFSEP): A unique multimodal nationwide MS registry in France*, Multiple Sclerosis Journal, **26(1)**:pp. 118–122, pMID: 30541380

[87] C. Confavreux, D. A. Compston, O. R. Hommes, W. I. McDonald, & A. J. Thompson (1992) *EDMUS, a European database for multiple sclerosis.*, Journal of Neurology, Neurosurgery & Psychiatry, **55(8)**:pp. 671–676

[88] A. Akshay, M. Abedi, N. Shekarchizadeh, F. C. Burkhard, M. Katoch, A. Bigger-Allen, R. M. Adam, K. Monastyrskaya, & A. Hashemi Gheinani (2023) *MLcps: machine learning cumulative performance score for classification problems*, GigaScience, **12**:p. giad108

[89] M. Abedi, N. Shekarchizadeh, P.-L. Bazin, N. Scherf, J. Lier, C.-C. Bergner, W. Köhler, & T. Kirsten (2025), *HeteroMRI: Robust white matter abnormality classification across multi-scanner mri data (version 1)*, [Computer software], Software Heritage, https://archive.softwareheritage.org/browse/snapshot/defd0e13d67d336ca96031dd434ea4779bc1ec2a/directory/

[90] M. Abedi, N. Shekarchizadeh, P.-L. Bazin, N. Scherf, J. Lier, C.-C. Bergner, W. Köhler, & T. Kirsten (2025), *HeteroMRI: Robust white matter abnormality classification across multi-scanner mri data*, [DOME-ML Annotations], DOME-ML Registry, https://registry.dome-ml.org/review/zj8thzbsa7

Supplementary Table S1: Number of MRIs used for training (Tr), validation (V), and test (T) sets from each *dataset* in experimental settings $A00$ to $A18$

**Dataset**		**Set**	**Setting $A\#$**																		
**Label***	**Name**		**00**	**01**	**02**	**03**	**04**	**05**	**06**	**07**	**08**	**09**	**10**	**11**	**12**	**13**	**14**	**15**	**16**	**17**	**18**
+	ISBI	Tr	8	7	6	5	4	3	2	1	0	0	0	0	0	0	0	0	0	0	0
		V	1	1	1	1	1	1	1	1	0	0	0	0	0	0	0	0	0	0	0
		T	2	2	2	2	2	2	2	2	2	2	2	2	2	2	2	2	2	2	2
	UMCL	Tr	8	7	6	5	4	3	2	1	0	0	0	0	0	0	0	0	0	0	0
		V	1	1	1	1	1	1	1	1	0	0	0	0	0	0	0	0	0	0	0
		T	2	2	2	2	2	2	2	2	2	2	2	2	2	2	2	2	2	2	2
	MSSEG	Tr	8	7	6	5	4	3	2	1	0	0	0	0	0	0	0	0	0	0	0
		V	1	1	1	1	1	1	1	1	0	0	0	0	0	0	0	0	0	0	0
		T	2	2	2	2	2	2	2	2	2	2	2	2	2	2	2	2	2	2	2
	MSSEG-2	Tr	8	7	6	5	4	3	2	1	0	0	0	0	0	0	0	0	0	0	0
		V	1	1	1	1	1	1	1	1	0	0	0	0	0	0	0	0	0	0	0
		T	2	2	2	2	2	2	2	2	2	2	2	2	2	2	2	2	2	2	2
	BTH	Tr	6	5	4	3	2	1	0	0	0	0	0	0	0	0	0	0	0	0	0
		V	1	1	1	1	1	1	0	0	0	0	0	0	0	0	0	0	0	0	0
		T	2	2	2	2	2	2	2	2	2	2	2	2	2	2	2	2	2	2	2
	OASIS-3	Tr	10	9	8	7	6	5	4	3	2	1	0	0	0	0	0	0	0	0	0
		V	2	2	2	1	1	1	1	1	1	1	0	0	0	0	0	0	0	0	0
		T	2	2	2	2	2	2	2	2	2	2	2	2	2	2	2	2	2	2	2
	ADNI 3	Tr	39	35	31	28	26	23	21	19	17	14	12	9	7	6	5	4	3	2	1
		V	6	6	6	6	6	6	5	5	5	5	4	4	3	3	3	2	2	1	1
		T	10	10	10	10	10	10	10	10	10	10	10	10	10	10	10	10	10	10	10
−	ICBM	Tr	3	2	1	0	0	0	0	0	0	0	0	0	0	0	0	0	0	0	0
		V	1	1	1	0	0	0	0	0	0	0	0	0	0	0	0	0	0	0	0
		T	1	1	1	1	1	1	1	1	1	1	1	1	1	1	1	1	1	1	1
	OASIS-3	Tr	65	58	51	45	39	32	26	21	15	12	10	8	7	6	5	4	3	2	1
		V	9	9	9	9	9	9	8	8	5	5	3	3	3	3	3	2	2	1	1
		T	16	16	16	16	16	16	16	16	16	16	16	16	16	16	16	16	16	16	16
	CERMEP	Tr	19	17	15	13	11	9	7	5	4	3	2	1	0	0	0	0	0	0	0
		V	3	3	3	3	3	3	2	2	1	1	1	1	0	0	0	0	0	0	0
		T	5	5	5	5	5	5	5	5	5	5	5	5	5	5	5	5	5	5	5
+	Total	Tr	87	77	67	58	50	41	33	26	19	15	12	9	7	6	5	4	3	2	1
		V	13	13	13	12	12	12	10	10	6	6	4	4	3	3	3	2	2	1	1
		T	22	22	22	22	22	22	22	22	22	22	22	22	22	22	22	22	22	22	22
−	Total	Tr	87	77	67	58	50	41	33	26	19	15	12	9	7	6	5	4	3	2	1
		V	13	13	13	12	12	12	10	10	6	6	4	4	3	3	3	2	2	1	1
		T	22	22	22	22	22	22	22	22	22	22	22	22	22	22	22	22	22	22	22
**Total**			244	224	204	184	168	150	130	116	94	86	76	70	64	62	60	56	54	50	48

* With (+) and without (−) WM abnormality

Supplementary Table S2: Number of MRIs used for training (Tr), validation (V), and test (T) sets from each MRI acquisition *protocol* in experimental settings $B00$ to $B04$

**Protocol**		**Set**	**Setting**				
**Label***	**Name**		$B00$	$B01$	$B02$	$B03$	$B04$
+	Sie_Tri_30_Prot1	Tr	5	4	3	2	1
		V	1	1	1	1	1
		T	1	1	1	1	1
	Phi_Ing_30_NA	Tr	5	4	3	2	1
		V	1	1	1	1	1
		T	1	1	1	1	1
	Phi_NA_30_Prot1	Tr	5	4	3	2	1
		V	1	1	1	1	1
		T	1	1	1	1	1
	Sie_Aer_15_Prot1	Tr	5	4	3	2	1
		V	1	1	1	1	1
		T	1	1	1	1	1
	GeE_Dis_30_Prot3	Tr	5	4	3	2	1
		V	1	1	1	1	1
		T	1	1	1	1	1
−	Sie_Bio_30_Prot1	Tr	5	4	3	2	1
		V	1	1	1	1	1
		T	1	1	1	1	1
	Sie_Son_15_Prot1	Tr	5	4	3	2	1
		V	1	1	1	1	1
		T	1	1	1	1	1
	Sie_MaV_30_Prot2	Tr	5	4	3	2	1
		V	1	1	1	1	1
		T	1	1	1	1	1
	Sie_MaV_30_Prot1	Tr	5	4	3	2	1
		V	1	1	1	1	1
		T	1	1	1	1	1
	Sie_TrT_30_Prot2	Tr	5	4	3	2	1
		V	1	1	1	1	1
		T	1	1	1	1	1
+	Total	Tr	25	20	15	10	5
		V	5	5	5	5	5
		T	5	5	5	5	5
−	Total	Tr	25	20	15	10	5
		V	5	5	5	5	5
		T	5	5	5	5	5
**Total**			70	60	50	40	30

* With (+) and without (−) WM abnormality

Supplementary Table S3: Number of MRIs used for training (Tr), validation (V), and test (T) sets from each MRI acquisition *protocol* in experimental settings $C00$ to $C06$. The two protocols in boldface are used only as test data. These two protocols are changed in 10 different cases.

Protocol		Set	Setting						
**Label***	**Name**		$C00$	$C01$	$C02$	$C03$	$C04$	$C05$	$C06$
+	Phi_Ing_30_NA	Tr	7	6	5	4	3	2	1
		V	1	1	1	1	1	1	1
		T	0	0	0	0	0	0	0
	GeE_Dis_30_Prot3	Tr	7	6	5	4	3	2	1
		V	1	1	1	1	1	1	1
		T	0	0	0	0	0	0	0
	Sie_Pri_30_Prot1	Tr	6	5	4	3	2	1	0
		V	2	2	2	1	1	1	0
		T	0	0	0	0	0	0	0
	Sie_Ver_30_Prot1	Tr	6	5	4	3	2	1	0
		V	2	1	1	1	1	1	0
		T	0	0	0	0	0	0	0
	**Phi_Ing_30_Prot2**	Tr	0	0	0	0	0	0	0
		V	0	0	0	0	0	0	0
		T	5	5	5	5	5	5	5
−	Sie_MaV_30_Prot1	Tr	6	5	4	3	2	1	0
		V	1	1	1	1	1	1	0
		T	0	0	0	0	0	0	0
	Sie_Son_15_Prot1	Tr	20	17	14	11	8	5	2
		V	5	4	4	3	3	3	2
		T	0	0	0	0	0	0	0
	**Sie_TrT_30_Prot1**	Tr	0	0	0	0	0	0	0
		V	0	0	0	0	0	0	0
		T	5	5	5	5	5	5	5
+	Total	Tr	26	22	18	14	10	6	2
		V	6	5	5	4	4	4	2
		T	5	5	5	5	5	5	5
−	Total	Tr	26	22	18	14	10	6	2
		V	6	5	5	4	4	4	2
		T	5	5	5	5	5	5	5
**Total**			74	64	56	46	38	30	18

* With (+) and without (−) WM abnormality

Supplementary Table S4: Number of MRIs used for training (Tr), validation (V), and test (T) sets from each MRI acquisition *protocol* in experimental settings $D00$ to $D03$

Protocol		Set	Setting			
**Label***	**Name**		$D00$	$D01$	$D02$	$D03$
+	Sie_Tri_30_Prot1	Tr	21	12	9	8
+	Sie_Tri_30_Prot1	V	3	2	2	1
+	Sie_Tri_30_Prot1	T	6	3	2	2
+	Phi_Ing_30_NA	Tr	15	12	9	8
+	Phi_Ing_30_NA	V	2	2	1	1
+	Phi_Ing_30_NA	T	4	3	3	2
+	Phi_NA_30_Prot1	Tr	0	12	9	7
+	Phi_NA_30_Prot1	V	0	1	1	1
+	Phi_NA_30_Prot1	T	0	4	3	2
+	Sie_Aer_15_Prot1	Tr	0	0	9	7
+	Sie_Aer_15_Prot1	V	0	0	1	1
+	Sie_Aer_15_Prot1	T	0	0	2	2
+	GeE_Dis_30_Prot3	Tr	0	0	0	6
+	GeE_Dis_30_Prot3	V	0	0	0	1
+	GeE_Dis_30_Prot3	T	0	0	0	2
−	Sie_Bio_30_Prot1	Tr	21	124	11	9
−	Sie_Bio_30_Prot1	V	3	2	2	1
−	Sie_Bio_30_Prot1	T	6	3	2	3
−	Sie_Son_15_Prot1	Tr	15	12	10	8
−	Sie_Son_15_Prot1	V	2	2	1	1
−	Sie_Son_15_Prot1	T	4	3	4	3
−	Sie_MaV_30_Prot2	Tr	0	12	10	9
−	Sie_MaV_30_Prot2	V	0	1	1	1
−	Sie_MaV_30_Prot2	T	0	4	3	2
−	Sie_MaV_30_Prot1	Tr	0	0	5	5
−	Sie_MaV_30_Prot1	V	0	0	1	1
−	Sie_MaV_30_Prot1	T	0	0	1	1
−	Sie_TrT_30_Prot2	Tr	0	0	0	5
−	Sie_TrT_30_Prot2	V	0	0	0	1
−	Sie_TrT_30_Prot2	T	0	0	0	1
+	Total	Tr	36	36	36	36
+	Total	V	5	5	5	5
+	Total	T	10	10	10	10
−	Total	Tr	36	36	36	36
−	Total	V	5	5	5	5
−	Total	T	10	10	10	10
**Total**			102	102	102	102

* With (+) and without (−) WM abnormality

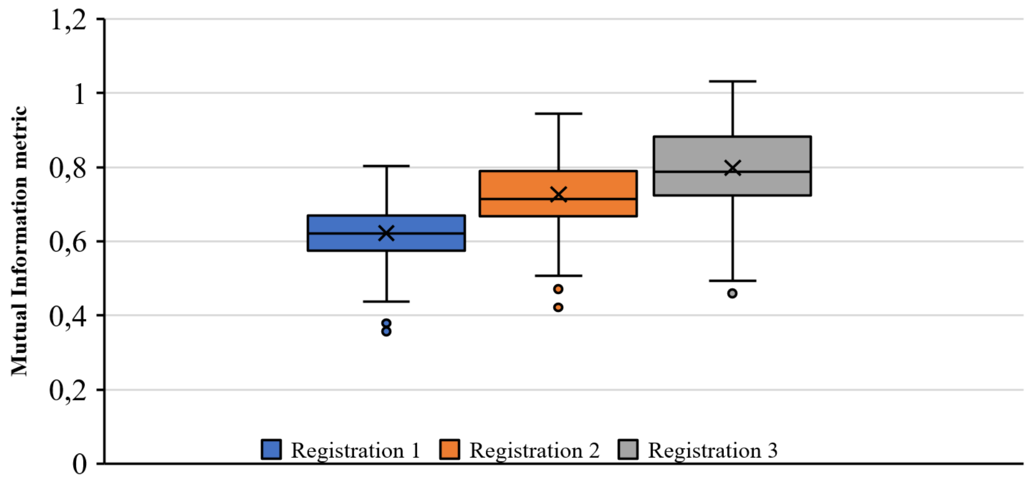

Supplementary Figure S1: Mutual information metric between each MRI and the MNI template after each registration. The × marker indicates the mean value, and the whiskers represent 1.5 × IQR.

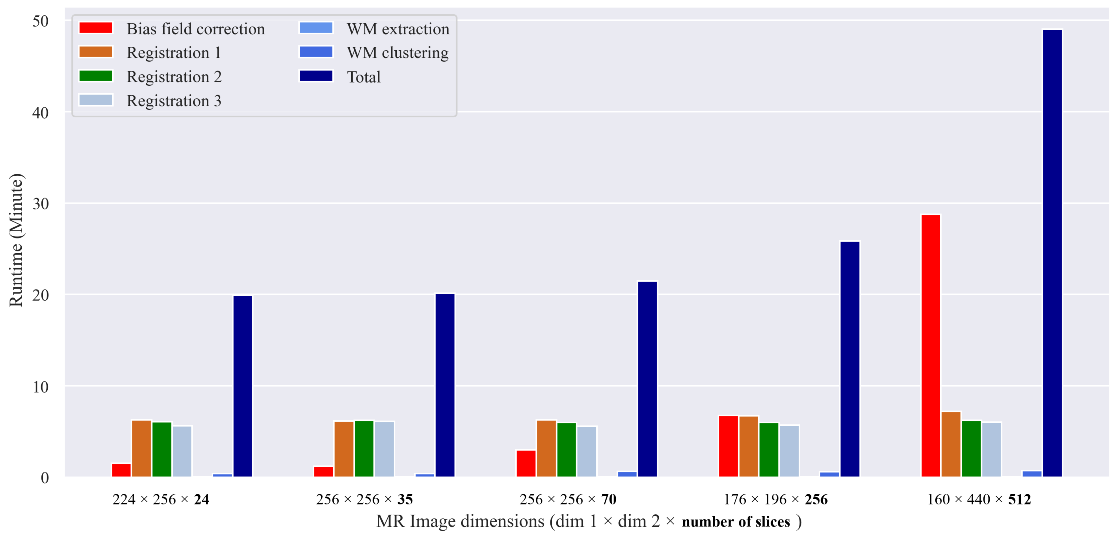

Supplementary Figure S2: Average required time for preprocessing and intensity clustering of five sample MRI dimensions.

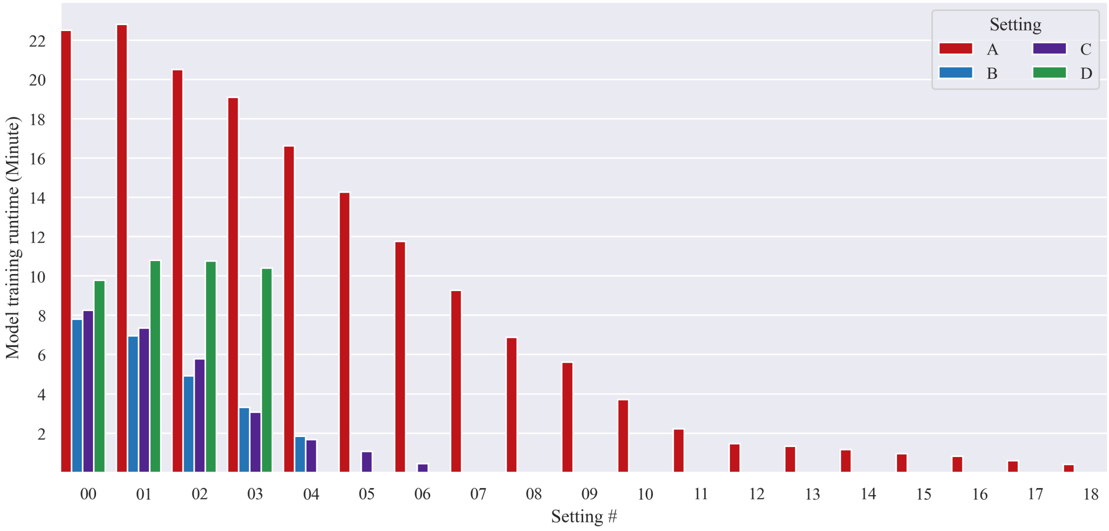

Supplementary Figure S3: Average required time for training the CNN model of each experimental setting.

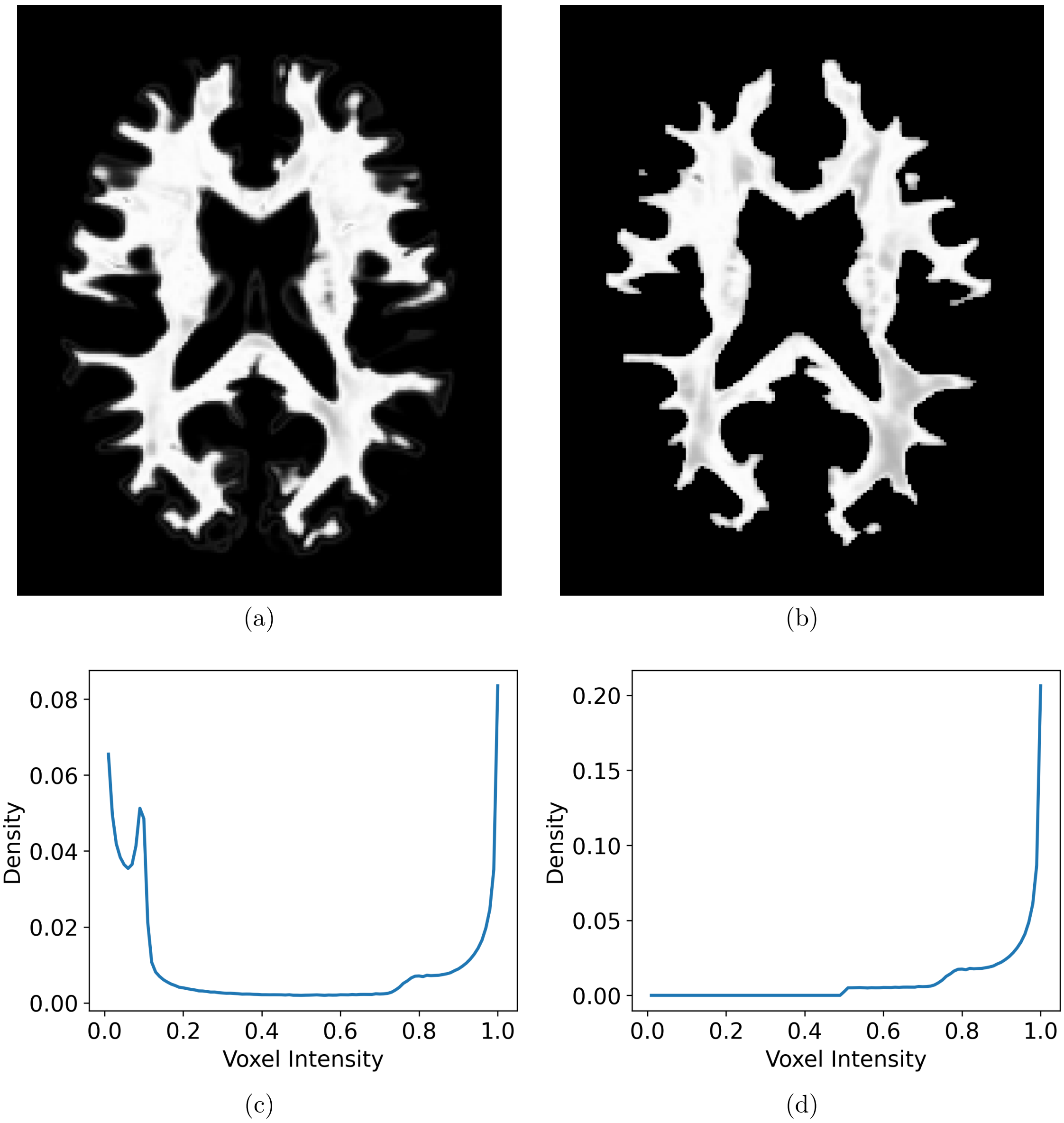

Supplementary Figure S4: The WM intensity cluster of a sample MRI (a) before and (b) after applying a threshold value of 0.5. The normalized histograms of their 99% upper percentile are shown in (c) and (d), respectively.

Manuscript

Click here to access/download;Manuscript;HeteroMRI_Manuscript_rev3.pdf

HeteroMRI: Robust white matter abnormality classification across multi-scanner MRI data

Masoud Abedi [1,2,3,†], Navid Shekarchizadeh [2,3,4,†,§], Pierre-Louis Bazin [5], Nico Scherf [4,6], Julia Lier [7,8], Christa-Caroline Bergner [7,8], for the Alzheimer's Disease Neuroimaging Initiative* , Wolfgang Köhler [7,8,‡], and Toralf Kirsten [1,2,3,4,‡]

[1]Faculty Applied Computer and Bio Sciences, Mittweida University of Applied Sciences, Mittweida, Germany

[2]Department for Medical Data Science, Leipzig University Medical Center, Leipzig, Germany

[3]Institute for Medical Informatics, Statistics, and Epidemiology (IMISE), Leipzig University, Leipzig, Germany

[4]Center for Scalable Data Analytics and Artificial Intelligence (ScaDS.AI) Dresden/Leipzig, Leipzig University, Leipzig, Germany

[5]Full brain picture Analytics, Leiden, The Netherlands

[6]Neural Data Science and Statistical Computing, Max Planck Institute for Human Cognitive and Brain Sciences, Leipzig, Germany

[7]Department of Neurology, Leipzig University Medical Center, Leipzig, Germany

[8]Myelin Research Center (MRC) Leipzig, Department of Neurology, Leipzig University Medical Center, Leipzig, Germany

[§]Correspondence address. Navid Shekarchizadeh, ScaDS.AI, Humboldtstraße 25, 04105 Leipzig, Germany. E-mail: navid.shekarchizadeh@uni-leipzig.de

[†]Equal contribution

[‡]Joint senior authorship

Abstract

Background

Magnetic Resonance Imaging (MRI) is commonly used for analyzing white matter abnormalities in the human brain. Integrating machine learning into MRI analysis can enhance diagnostic processes. However, the application of such techniques for white matter analysis in clinical practice is often limited when MRI data is multi-scanner (i.e., heterogeneous), particularly in scenarios with limited data, as seen in rare diseases. Therefore, it is crucial to develop methods that are highly independent of the MRI scanner and acquisition protocol.

Results

This study introduces *HeteroMRI*, a deep-learning method for classifying MRIs based on white

*A part of the data used in preparation of this article were obtained from the Alzheimer's Disease Neuroimaging Initiative (ADNI) database (adni.loni.usc.edu). As such, the investigators within the ADNI contributed to the design and implementation of ADNI and/or provided data but did not participate in analysis or writing of this report. A complete listing of ADNI investigators can be found at: http://adni.loni.usc.edu/wp-content/uploads/how_to_apply/ADNI_Acknowledgement_List.pdf

matter abnormalities. Most importantly, HeteroMRI mitigates the effects of data heterogeneity on classification performance. Herein, HeteroMRI is employed to detect brain MRIs with white matter abnormalities. This method utilizes intensity clustering of the white matter tissue to reduce the effects of the heterogeneity of MRIs. MRI data from 11 public datasets with 40 MRI protocols are included. By using 200 MRIs for training the model, the binary classifier achieves an average accuracy of 93±4%. Furthermore, the method is evaluated in limited data scenarios, simulating conditions of rare diseases. By reducing the data by 64% and 75%, the model's accuracy has a 4% and 12% decrease, respectively.

Conclusions

The presented method opens new avenues for white matter abnormality-related classification of heterogeneous MRI data without additional machine learning methods to reduce MRI heterogeneity. This classification approach demonstrates a high degree of independence from the MRI scanner and protocol, while also proving to be relatively generalizable to unseen MRI protocols.

Keywords— Brain MRI classification, Multi-scanner MRI, Multi-protocol MRI, Intensity clustering, White matter abnormality, Rare disease, Convolutional neural network

1 Introduction

Magnetic Resonance Imaging (MRI) of the brain is widely used to diagnose neurological diseases as it provides a clear contrast between the different tissues of the brain, including white matter (WM) and gray matter (GM) [1]. Detection and assessment of WM abnormalities or lesions in demyelinating or neurodegenerative diseases are an important application of MRI in daily clinical practice [2]. An excellent contrast for visualizing WM abnormalities is provided by the Fluid-Attenuated Inversion Recovery (FLAIR) imaging technique, making the abnormalities stand out from the surrounding normal brain tissue. FLAIR is a T2-weighted imaging technique in which the signal from cerebrospinal fluid (CSF) is suppressed, which facilitates the detection of WM abnormalities as they may appear adjacent to CSF-filled spaces [3]. The MRI technique allows for the study of the pattern and volume of WM lesions, which ultimately contributes to image-based diagnosis of demyelinating and other neurological disorders [4].

In recent years, Artificial Intelligence (AI) has revolutionized the medical imaging domain, offering powerful tools for automating time-consuming tasks such as lesion segmentation [5]. This reduces examiner-based variability and enables healthcare professionals to focus on critical aspects of diagnosis and research. In brain MRI analysis, Machine Learning (ML) and Deep Learning (DL) models have been widely applied [6] for tasks including disease classification [7, 8, 9], WM lesion segmentation [10, 11], tumor detection and grading [12, 13], stroke lesion analysis [14, 15], brain age prediction [16, 17], and brain tissue segmentation [18, 19]. Additionally, AI-based algorithms are commonly used in MRI preprocessing steps such as image registration [20], brain extraction [21, 22], denoising [23, 24, 25], intensity normalization [26, 27], bias field correction [28], and MRI interpolation [29].

A challenge in using MRI data in ML/DL models is the variability of MRIs across different sites and scanners. It is shown that scanner differences lead to significant biases in automated MS lesion volumetric analyses, even when the scanner manufacturer and acquisition protocol are consistent [30]. Acquisition protocol refers to a set of procedures and parameters, e.g., Echo Time (TE), Repetition Time (TR), and Inversion Time (TI), used to acquire the images. The variabilities due to different scanners and acquisition protocols are often greater than the biological variabilities [31, 32, 33, 34, 35, 36]. High-capacity classifiers, such as deep neural networks, often struggle to produce consistent outcomes when applied to multi-scanner data. This limitation is caused by the model's tendency to overfit to non-biological variations; thus, the model fails to detect desired biological features or to generalize well across MRI data from unseen scanners [37].

A common approach to address scanner variability is using standardized MRI datasets—acquired with identical scanners and protocols—to improve model consistency [38, 39]. While standardization enhances image comparability and model performance, it requires substantial coordination and resources. Moreover, many DL models still fail to generalize to unseen scanners or protocols [40], limiting their clinical utility. Thus, methods that are robust to scanner and protocol variability are crucial.

Another approach to address scanner-related heterogeneity in MRI data is *harmonization*, which aims to remove scanner and protocol effects before analysis. Statistical techniques such as intensity normalization [41, 26] or ComBat-based batch effect correction [33, 32, 31, 34, 35, 36] have shown success to some extent, but often fail to improve DL model performance in disease classification tasks [37]. More recent harmonization approaches rely on supervised [42, 43] and unsupervised [44, 45] DL methods, with hybrid models like DeepComBat also emerging [46]. While these methods aim to learn and remove scanner-specific features, they often rely on restrictive assumptions, such as availability of matched subjects across scanners [42], standardized acquisition protocols across scanners [44], necessity of multi-contrast data from the same session [45], or requiring a large amount of training data [44]. Furthermore, there is still no universal harmonization strategy, and even after harmonization, DL models must still be robustly generalizable to unseen scanners and protocols. For an extensive review of MRI harmonization methods, see [47].

On a different note, the context of rare diseases has specific challenges and limitations. Data availability is extremely limited, which severely restricts the application of ML/DL approaches to these diseases, including both predictive models, such as classifiers, and harmonization methods. For example, in the case of leukodystrophies [48], the brain MRIs are gathered from various clinical centers over a long period, making it infeasible to even create a standardized dataset. Such datasets are not only small in size but also highly heterogeneous in terms of MRI scanners and protocols. These conditions significantly hinder the applicability of conventional ML methods to rare diseases.

Several existing methods address WM abnormality analysis, such as lesion segmentation or volumetric assessments, typically using supervised deep learning models. Many of these approaches require large amounts of voxel-wise, manually annotated data for training [49, 50, 51]. Some methods specifically target

the segmentation of lesions within the brain [52, 11, 53, 54, 55], whereas this study focuses on classifying MRIs based on the WM abnormalities. Additionally, several segmentation models require the availability of multiple MRI sequences (e.g., FLAIR, T1, T2) [56, 38, 19], while the approach presented in this study is designed to operate solely on FLAIR images, enhancing its practicality and ease of deployment across heterogeneous clinical datasets.

Herein, we present HeteroMRI, an approach for classifying brain MRIs based on WM abnormalities while mitigating the heterogeneity effects of the images acquired from multiple scanners and acquisition protocols. In this paper, HeteroMRI is utilized to detect brains with WM abnormalities in FLAIR images through binary classification. HeteroMRI is applicable to multi-scanner and multi-protocol datasets and demonstrates effectiveness in data-limited conditions, providing a flexible and practical solution for both research and clinical applications. The presented method employs MRI intensity clustering, a technique used in the literature for other MRI-related purposes such as brain tissue segmentation [57, 58], brain tumor segmentation [59], and inhomogeneity correction [60]. HeteroMRI is evaluated in various experimental settings to ensure its robustness. Additionally, we apply the method to limited data scenarios in order to assess the performance and applicability of the presented method for rare diseases. In future work, the method is intended to be applied to the task of classifying two WM diseases based on their distinct WM abnormality patterns. The presented method opens new avenues for performing WM abnormality-related analyses on heterogeneous MRI datasets and the large amount of MRI data generated daily in medical centers.

The current paper is structured in the following way: Sec. 2 provides an overview of the methodology used, detailing the data preprocessing steps and the architecture of the DL model employed in this study. Moving on to Sec. 3, the experiments, the datasets used, the experimental settings, the execution of the model, and the evaluation metrics are presented. Following that, Sec. 4 presents the key results, while Sec. 5 discusses the results, highlights the limitations of the method, and introduces the possible future directions. Finally, Sec. 6 provides the conclusion.

2 Methodology

The WM abnormality detection approach presented in this paper uses heterogeneous brain MRI data with various acquisition protocols (multi-protocol) as the input data for a Convolutional Neural Network (CNN). The model is a binary classifier trained to detect patients with WM abnormalities in their brain MRI. The method consists of three main modules explained in the following subsections, namely, MRI preprocessing, Intensity clustering, and DL model. An overview of the methodology is illustrated in Fig. 1.

2.1 MRI preprocessing

For preparing the image data for the analysis, we use our brain MRI preprocessing pipeline, *FlexiMRIprep*[1], that consecutively performs all the requested preprocessing steps/algorithms on all the selected images

[1] https://github.com/ul-mds/FlexiMRIprep

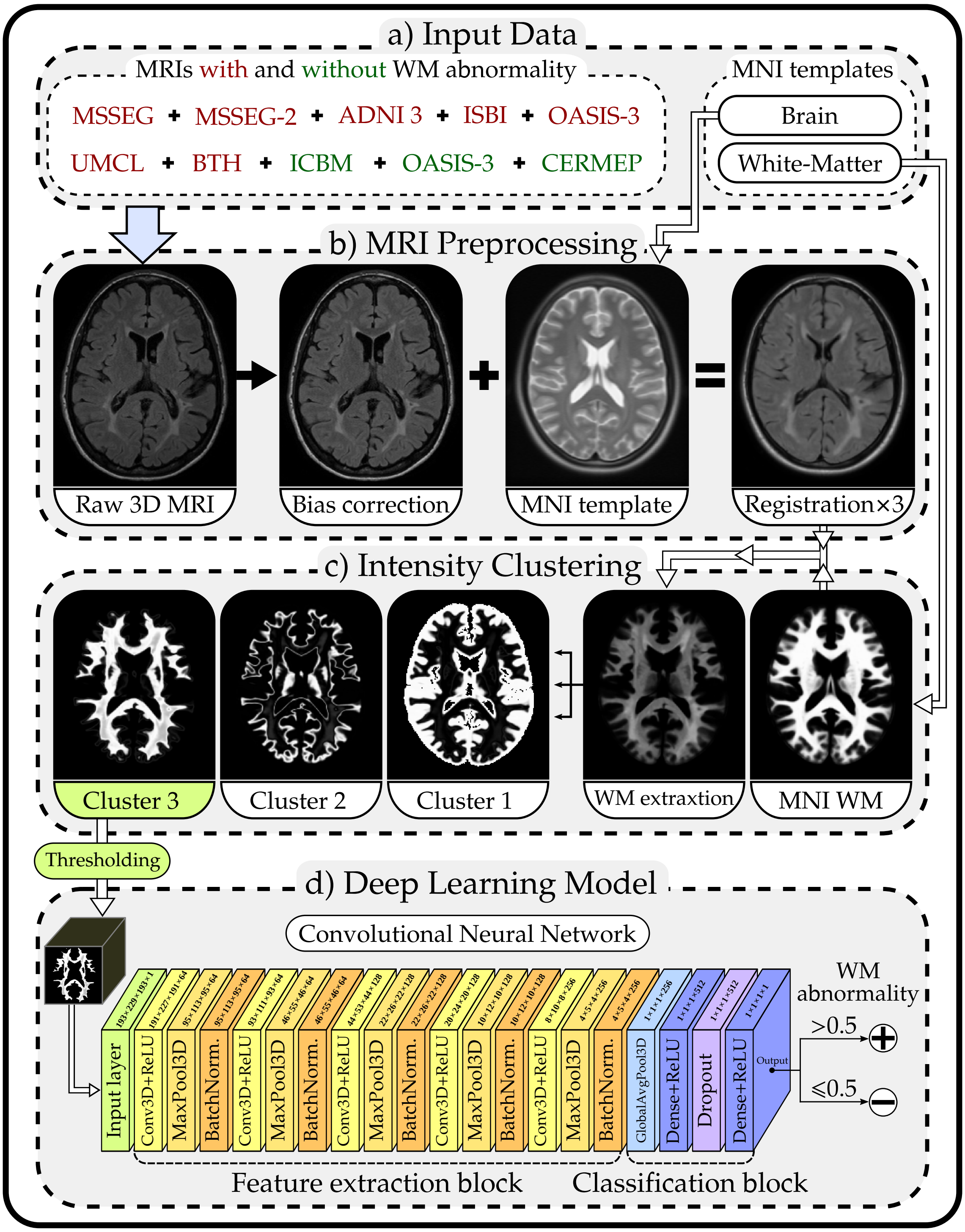

Figure 1: Overview of the methodology: a) Input data: The MRI datasets used for the classification model and MNI brain template [61, 62]. The MRI data with and without WM abnormality are taken from the datasets shown in red and green, respectively. b) MRI preprocessing: The N4 bias field correction method [28] is applied on the FLAIR MRIs (in 3D), and then the MRIs are three times registered (nonlinearly) to the MNI template. c) Intensity clustering: The WM of the brain is extracted, and the WM is clustered into three intensity clusters using RFCM [63] algorithm. d) DL model: Only Cluster 3 of the WM is thresholded and used for a binary classification model with the CNN architecture shown.

automatically. Being the optimal MRI sequence in detecting WM abnormalities, only FLAIR images are used in the analysis in this paper. All selected images have a minimum of 128, 192, and 22 voxels in their first, second, and third dimensions, respectively. All the MRIs are converted to the Neuroimaging Informatics Technology Initiative (NIfTI-1) format using the `dcm2niix` tool (version 1.0.20211006) [64] at this point. The preprocessing steps described below are applied identically to all MRIs from different datasets. Detailed information on the parameters used in each step is reported in the GitHub repository of HeteroMRI[2].

- **Bias field correction**: For correcting the bias field or inhomogeneity issues in the MRIs, we employ the commonly used N4ITK [28] bias field correction method for this purpose. For implementation, the `N4BiasFieldCorrectionImageFilter` class from the `SimpleITK` [65] (version 2.1.1.2) *Python* library with the default parameters is used.

- **Registration**: Registration enables precise spatial mapping and the comparison of anatomical structures among the MRIs. In this process, all the MRIs used for training and testing the AI model are aligned to a standard brain template. Among the available brain templates, we choose the "ICBM 2009c Nonlinear Asymmetric" template [61, 62] (referred to below as the MNI template), which the developers created using the data from the International Consortium for Brain Mapping (ICBM) project [66]. This template was selected due to its high accuracy and the availability of the WM probability map required for our analysis approach. Since there is no dedicated FLAIR template in the MNI template, the T2-weighted template was used due to its proximity to FLAIR. For the registration, the `antsRegistration` tool from the Advanced Normalization Tools (ANTs) [67] (version 2.4.4) is employed. A nonlinear registration is applied three times (with identical parameters) on each image consecutively. This repeated registration aims to achieve a high level of alignment of the MRIs with the template. Mutual information (MI) was calculated between each registered MRI and the MNI template, after each of the three registration steps. As shown in Supplementary Fig. S1, the MI increased after each nonlinear registration step, with mean values improving from 0.62 (1st registration), to 0.73 (2nd registration) and 0.80 (3rd registration). However, the improvement between the second and third steps was relatively small (ΔMI ≈ 0.072), indicating diminishing returns. This suggests that three registration steps are sufficient to achieve consistent and reliable alignment. Performing additional iterations may not justify the computational cost and could even introduce unnecessary anatomical distortions, particularly due to the nonlinear nature of the transformations. Multi-pass registration has also been used by others, for example, to address large differences in the initial positions of image pairs [68]. The registered MRIs all have a size of $193 \times 229 \times 193$ voxels with a voxel size of $1 \times 1 \times 1$ mm.

2.2 Intensity clustering

- **WM extraction**: After the brain images are aligned with the MNI template, the WM probability map of the template is used to extract the WM volumes of each brain. Therefore, all the other

[2] `https://github.com/ul-mds/HeteroMRI`

brain tissues are removed. The WM extraction is performed by using the `MultiplyImages` tool from ANTs.

- **WM clustering**: The WM clustering is performed for two essential purposes: 1) to obtain a subgroup of WM volumes that includes significant signs of WM abnormalities, and 2) to reduce the negative effect of the heterogeneous MRI data coming from multiple scanners and protocols. These two points are elaborated on in the following.

A clustering algorithm is used to estimate three intensity clusters from the WM volumes obtained in the previous step. The algorithm groups the WM volumes into three subgroups that share a relatively similar intensity range. We expect that one of the clusters shows more indications of WM abnormalities (if present in the brain) since the abnormalities have higher intensity values in FLAIR images. This specific cluster will be used as the training data for the classifier model. The cluster is a membership function with float values in the range of [0,1] for each voxel. As a result, the impact of the heterogeneous nature of the multi-protocol MRIs is reduced, in a way that the proposed method is robust to the many MRI protocols we used in this work. The decision to use three clusters was based on our empirical observations from a dataset different from those used in this study. Through testing various cluster numbers on different MRIs, we found that three clusters yielded consistently comparable patterns in nearly all MRIs. In other words, the shapes of the clusters in one MRI were generally consistent with those in another MRI. This was also later observed in the data of the current study. Therefore, the choice of three clusters is robust and does not depend on a specific dataset.

Here, we employ a Robust Fuzzy C-Means (RFCM) algorithm [63] for WM intensity clustering. The RFCM algorithm modifies the standard FCM objective function by incorporating a local spatial penalty term, leading to the computation of smoother membership functions. This modification not only improves segmentation performance but also provides a level of noise insensitivity. The RFCM algorithm is implemented using the `fuzzy_cmeans` function available in the *Nighres* (Neuroimaging at high resolution) *Python* package, version 1.4.0 [69].

Upon examining the three WM intensity clusters in MRIs with WM abnormalities, we noted that one of the clusters within each MRI, Cluster 3 in Fig. 1(c), consistently exhibited significant lesion-related features. Therefore, from each MRI, we should take the cluster that looks visually similar to Cluster 3 in Fig. 1(c), but it is not always the cluster number 3. For this purpose, we use the Dice similarity coefficient[70] to compare the three clusters of each MRI with a fixed reference cluster to detect the most similar one. The reference image (available in the GitHub repository of HeteroMRI) is generated by averaging the intended intensity cluster of four MRIs from a clinical dataset. This method detected the right cluster for all the MRIs of this study correctly (i.e., with 100% accuracy) as checked manually.

- **Thresholding**: A thresholding is applied on the selected WM intensity cluster of each MRI. All the intensity values below 0.5 are ignored in order to remove uncertain, low-confidence assignments

and retain only the core voxels that are strongly associated with the cluster. The value 0.5 is chosen experimentally in the design phase of HeteroMRI by using (clinical) datasets different from those used in this study for the task of classifying two WM diseases. The value 0.5 resulted in the highest improvement in the classification accuracy compared to other tested thresholds. The histogram for most MRIs follows the same overall pattern: approximately $50 \pm 5\%$ of intensity values are below 0.2, and around $35 \pm 5\%$ are above 0.8. Supplementary Fig. S4 shows the WM cluster of a sample MRI before and after thresholding along with their normalized histograms (for the 99% upper percentile). Finally, the thresholded clusters from the MRIs (one intensity cluster per MRI) are used as training data for the DL model, as described in the following section.

2.3 Deep learning model

The objective is to train a binary classifier model that detects the brain MRIs that have WM abnormalities. Inspired by [71, 72], we configured a 3D CNN comprising a total of 20 layers, as illustrated in Fig. 1(d). The network has a total of 1,795,905 parameters. The model begins with the input layer, followed by a feature extraction block, and ends with a classification block.

In the feature extraction block, we employ five 3D Convolution (Conv3D) layers with 64, 64, 128, 128, and 256 filters, respectively. Each Conv3D has a 3x3x3 kernel size and employs the Rectified Linear Unit (ReLU) activation function. Subsequently, each Conv3D layer is succeeded by a 3D Max Pooling (MaxPool3D) layer with a stride of (2,2,2) and a pool size of (2,2,2), which downscales the 3D input by half in each dimension. Batch Normalization [73] layers with default parameters follow each MaxPool3D layer.

In the classification block, a 3D Global Average Pooling (GlobalAvgPool3D) layer is followed by a Dense layer with a dimensionality of 512 and with a ReLU activation function. To help prevent overfitting, a Dropout layer with a 30% rate is introduced next. Finally, the output layer performs a binary classification employing a Sigmoid activation function. The binary cross-entropy loss, Adam optimizer [74], and an *Early Stopping* feature (*patience*=40) are employed in the model. In each epoch, the checkpoint feature saves the model if the validation accuracy has improved. In the case of an unchanged validation accuracy, the mode is saved if the validation loss has decreased. The *Python* implementation code of the HeteroMRI method is publicly available[3].

3 Experiments

Different MRI datasets are used along with multiple experimental settings with various conditions to train and evaluate the CNN model for classifying brain MRIs. In the following subsections, the datasets and the experimental settings are elaborated.

[3] https://github.com/ul-mds/HeteroMRI

3.1 Datasets

In this study, we utilized FLAIR images from multiple brain MRI datasets as introduced below. Incorporating a combination of MRIs with a high diversity of acquisition protocols and scanners ensures a robust evaluation of the presented methodology. All the datasets used in this study are either publicly available or are accessible upon request to the respective dataset providers. All the MRIs were visually checked by the authors to exclude images with large artefacts. As presented in Table 1, a total of 11 MRI datasets are utilized.

Table 1: MRI datasets used in this study. M/F: number of male/female subjects; Age: mean±standard deviation (years).

No.	Dataset name/alias	Images[1]		Demographics		Protocols[2]	Availability	Reference
		+	−	M/F	Age			
1	ISBI	19	0	4/15	40.4±9.2	1	Public	[75]
2	UMCL	30	0	7/23	median 39 (25–64)	1	Public	[76]
3	MSSEG	52	0	15/37	45.3±10.3	4	AoR[3]	[77]
4	MSSEG-2	40	0	N/A	N/A	10	AoR[3]	[78]
5	BTH	9	0	1/8	29.4±8.9	2	Public	[79]
6	ICBM	0	5	2/3	28.2±7.5	1	AoR[3]	[80]
7	OASIS-3	14	90	50/54	68.4±9.5	4	AoR[3]	[81]
8	ADNI 3	58	0	28/30	76.5±8.6	8	AoR[3]	[82]
9	CERMEP	0	27	14/13	34.9±9.3	1	AoR[3]	[83]
10	WMH	10	0	N/A**	N/A**	5	Public	[84]
11	PPMI	0	10	3/7	52.7±13.2	4	AoR[3]	[85]
	Sum	232	132	-	-	40*	-	-

[1] Number of FLAIR images, with (+) and without (−) WM abnormality
[2] Number of MRI protocols in the used data
[3] Accessible on Request (to the respective dataset provider)
* Sum of unique protocols in the data
** Not available on individual level

The details of each dataset are outlined below:

1. **ISBI**: The International Symposium on Biomedical Imaging (ISBI) in 2015 [75] conducted an MS lesion segmentation challenge using longitudinal MRI data. The dataset comprises imaging data from MS patients, acquired using the same scanner and protocol. We utilize 19 FLAIR images from this dataset. For each patient, the MRI taken at the latest time point is used.

2. **UMCL**: A cohort of MS patients was imaged at the University Medical Center Ljubljana (UMCL) [76]. The images were acquired using the same scanner and protocol. We use 30 3D FLAIR images from this dataset.

3. **MSSEG**: The MSSEG dataset [77] was presented for the MS lesion segmentation challenge during the MICCAI 2016 conference. The dataset contains MRIs of MS patients from four different sites. Each site used different MRI scanners and protocols. We utilize 52 FLAIR images[4] from this dataset.

4. **MSSEG-2**[5]: MSSEG-2 [78] is a challenge for the segmentation of new MS lesions in the brain conducted in the MICCAI 2021 conference. At the time of the current research, only the training

[4] The MSSEG dataset originally contains 53 FLAIR images, but one of them was inadvertently excluded from our analysis.

[5] Data were generated by participating neurologists in the framework of Observatoire Français de la Sclérose en Plaques (OFSEP), the French MS registry [86]. They collect clinical data prospectively in the European Database for MS (EDMUS) software [87]. MRI of patients were provided as part of a care protocol. Nominative data are deleted from MRI before transfer and storage on the Shanoir platform (Sharing NeurOImagingResources, shanoir.org).

data of the dataset is accessible. The images of the training set were acquired at 12 different sites and using 10 different scanners. All the images were acquired at two different time points from each patient. From this dataset, we utilize 40 3D FLAIR images from the second time point.

5. **BTH**: The brain MRI dataset of MS patients from Baghdad Teaching Hospital (BTH) [79] includes MRIs taken at 20 centers with different protocols. We used nine[6] FLAIR images from this dataset, which were taken using two different protocols.

6. **ICBM**[7]: The International Consortium for Brain Mapping (ICBM) [80] has developed a probabilistic atlas and reference system for the human brain for normal adults. The dataset includes 20 3D FLAIR images; however, only five were selected for this study, as our neurology specialist confirmed these to be the only ones free of WM abnormalities. The images were acquired using the same scanner and protocol.

7. **OASIS-3**: The Open Access Series of Imaging Studies (OASIS) project provides publicly available neuroimaging datasets. Among its releases, only OASIS-3 [81] includes FLAIR images, encompassing both cognitively normal individuals and those at various stages of cognitive decline. Approximately 850 subjects in OASIS-3 have FLAIR scans. For this study, we used 104 FLAIR and T2-FLAIR images acquired under four different MRI protocols. From a random subset of 600 images, 90 were selected by two neurology specialists based on the absence of WM abnormalities. The remaining 14 images were selected from the same pool to ensure representation of diverse WM lesion patterns, as confirmed through visual inspection.

8. **ADNI 3**: The Alzheimer's Disease Neuroimaging Initiative[8] (ADNI) provides a rich repository of neuroimaging and clinical data [82]. From ADNI-3, which includes FLAIR scans for over 1,100 subjects, we selected 58 3D FLAIR images specifically based on the diversity of their WM lesion patterns. A neurology specialist verified that these images include a range of lesion types, including multifocal, confluent, and brainstem lesions.

9. **CERMEP**: The CERMEP-IDB-MRXFDG dataset [83] comprises MRI, CT, and [^{18}F]FDG PET image data with BIDS standard of healthy subjects. The dataset has 37 FLAIR images obtained using the same scanner and protocol. As reported in the original study, these images underwent visual review by two neurologists to confirm the absence of any apparent brain abnormalities. However, due to our strict criteria for even minor lesions, our neurologists confirmed only 27 MRIs as free of WM abnormalities for use as control data in our model.

[6]The NIfTI files in this dataset lack orientation information (qform and sform), making it impossible for the registration algorithm to identify the correct orientation of the brain. Additionally, the MRIs are 2D, resulting in around 10 times fewer slices than the pixels in the first and second dimensions, and they all share the same pixel thickness across all three dimensions. Consequently, the brain appears unrealistically short in 3D view. Due to these dataset-specific conditions, we applied two additional preprocessing steps at the beginning for this dataset: 1) added correct orientation information to each file, and 2) edited slice thicknesses in the header of NIfTI files based on slice thickness information provided in the dataset's metadata. However, the height of many images still does not appear realistic and may cause problems for the registration. Therefore, only nine images were used

[7]The ICBM project (Principal Investigator John Mazziotta, M.D., University of California, Los Angeles) is supported by the National Institute of Biomedical Imaging and BioEngineering. ICBM is the result of efforts of co-investigators from UCLA, Montreal Neurologic Institute, University of Texas at San Antonio, and the Institute of Medicine, Juelich/Heinrich Heine University - Germany.

[8]The ADNI was launched in 2003 as a public-private partnership, led by Principal Investigator Michael W. Weiner, MD. The primary goal of ADNI (`https://adni.loni.usc.edu`) has been to test whether serial MRI, positron emission tomography (PET), other biological markers, and clinical and neuropsychological assessment can be combined to measure the progression of Mild Cognitive Impairment (MCI) and early Alzheimer's Disease (AD).

10. **WMH**: The White Matter Hyperintensity (WMH) segmentation challenge dataset [84], introduced at the MICCAI 2017 conference, includes MRI scans from 170 subjects acquired using five different scanners. The MRIs have different loads of WM lesion. In this paper, we used 10 FLAIR images from this dataset. From each scanner, two MRIs were taken (the first training and the first test MRI, and the first two test MRIs from the scanners that were used only as test data).

11. **PPMI**: The Parkinson's Progression Markers Initiative (PPMI) [85] is a large-scale, longitudinal study that offers comprehensive imaging, clinical, and biospecimen data from individuals with Parkinson's disease and healthy controls. For this study, we selected 10 FLAIR images without WM abnormalities, identified by a neurology specialist from approximately 220 healthy control subjects with available FLAIR scans.

From the MRI data explained above, the datasets one to nine (344 images) are used for training and testing the model in the experimental settings explained below, and the datasets 10 and 11 (20 images) are used as holdout sets. Around 36% of these MRIs are 2D, based on our definition that MRIs with 70 or fewer slices are considered 2D. A comprehensive list of the MRI files is available in the GitHub repository of HeteroMRI, providing details for each image, including the subject ID from the original dataset and the acquisition protocol.

3.2 Experimental settings

Various experimental settings have been designed for a robust evaluation of the presented classification approach. An experimental setting means the specification of the data used for training, validating, and testing the CNN model. By employing the datasets explained in Sec. 3.1, FLAIR images from different scanners and acquisition protocols are intentionally combined and used for training and testing the model. The images necessary for each setting are selected randomly from the MRIs available. The number of MRIs with and without WM abnormality is balanced in the training, validation, and test data of all the settings. There are four setting groups, namely A, B, C, and D. In setting A, the data are selected based on datasets, while in the settings B, C, and D, the data are incorporated based on their acquisition protocol. We assigned a protocol name to each of the MRIs based on the scanner name and model, magnetic field strength, and acquisition parameters. The protocol naming convention is explained in the HeteroMRI Github repository. The experimental settings are introduced below:

- **Setting *A***: In setting A, the goal is to evaluate HeteroMRI on a combination of MRIs from different datasets beginning from a relatively large number of data and then decreasing the data gradually. In setting A, there are 19 settings that are run independently. In $A00$, 244 MRIs from nine datasets are used. The data of *each* dataset is split into training (70%), validation (10%), and test (20%) sets. In $A01$, the training data is downsized by approximately 10% while the test set remains the same images as in $A00$. The downsizing process continues up to $A18$, where the training and validation sets together include only four MRIs. The downsizing is performed by removing random MRIs while keeping the maximum possible number of protocols among the data. Across all settings from $A00$ to $A18$, the test set remains identical. Supplementary Table S1 shows the number of MRIs used for training, validation, and test sets from each dataset in the settings $A00$ to $A18$.

- **Setting B**: In setting B, the goal is to choose the MRIs with the most diversity of protocols while having an equal number of MRIs from each protocol; therefore, the model sees the same number of MRIs per protocol. MRIs from 10 different protocols are incorporated. The test data is selected from all protocols. In $B00$, from each protocol, five MRIs for training, one MRI for validation, and one MRI for the test are used. In the subsequent settings, the training data is reduced. By $B04$, only one MRI per protocol is used for the training set. In Supplementary Table S2, the list of selected protocols and the number of MRIs used for training, validation, and test sets for $B00$ to $B04$ is reported.

- **Setting C**: In setting C, the goal is to assess the generalizability of HeteroMRI to unseen MRI protocols. In this setting, MRIs from eight different protocols are included. Only data from 3D MRIs are included in this setting due to the reason later explained in Sec. 4.3. MRIs from six protocols are used *only* in training and validation sets, while the other two protocols are *only* used in the test set. In fact, the trained model does not see any data from the protocols used in the test set during training. We consider 10 cases, in each case considering two different protocols for testing the model. The setting C begins with $C00$, which uses 64 MRIs for training and validation. This continues up to $C06$ with only eight MRIs for training and validation. In Table S3, the list of selected protocols and the number of MRIs used for training, validation, and test sets for $C00$ to $C06$ is reported.

- **Setting D**: In setting D, the goal is to see the effect of the number of MRI protocols on the performance of the model. Beginning from $D00$ and going toward $D03$, more protocols are included in the data used for training and testing the model. At the same time, the total number of data and test set size are kept the same among the settings $D00$ to $D03$ (82 MRIs for training including validation data), therefore it is possible to compare the results of the settings to see the effect of having more protocols in the data. In $D00$, there are MRIs from four protocols. In $D01$ to $D03$, there are MRIs from, respectively, six, eight, and 10 protocols. The test data is selected from all protocols. In Table S4, the list of selected protocols and the number of MRIs used for training, validation, and test sets for $D00$ to $D03$ is reported.

3.3 Model execution

All the MRIs used in this study, introduced in Sec. 3.1, are preprocessed following the procedure elaborated in Sec. 2.1. Next, the intensity clustering procedure is applied to each preprocessed MRI, following the procedure introduced in Sec. 2.2. As a result, a single intensity cluster per MRI is used for training or testing the model. Notably, the 3D intensity clusters obtained from the MRIs serve as the exclusive training data for the model. The model has no exposure to the original MRIs or any form of WM lesion annotation file. For the preprocessing and intensity clustering tasks, we used a machine with Intel(R) Xeon(R) Gold 6240R CPU @ 2.40GHz and 128 GB of RAM. The preprocessing pipeline (introduced in Sec. 2.1) employs a parallelization approach in some of the preprocessing steps to make the procedure faster. The computation time required for preprocessing each MRI depends on multiple factors; nevertheless, the total number of voxels in the 3D MRI plays a more significant role. More specifically, based on our assessments, the number of slices of the MR image highly affects the required computation time. The Supplementary Fig. S2 shows the average time required for preprocessing and intensity clustering of five

sample MRI dimensions.

The CNN model explained in Sec.2.3 is trained and tested on each experimental setting independently. In settings A, B, C, and D, the required number of data is selected (and split into training, validation, and test sets) from all available data 50 independent times (referred to below as "data shuffle"). For each data shuffle, the model is trained and tested 10 times. For training and testing the CNN model, we used a computational server with an AMD Epyc 7352 CPU, 1 TB of CPU RAM, and an NVIDIA A100 GPU (40 GB GPU RAM). The average required time for training the model of each setting is shown in Supplementary Fig. S3. The inference time of the model on a single test data is a few seconds. The inference process can be efficiently performed without the necessity of a GPU.

3.4 Evaluation metrics

To assess the performance of the classification model, we employ five common metrics: accuracy, sensitivity, specificity, F1 score, and precision. Moreover, the area under the receiver operating characteristic curve (AUROC) is reported for selected settings. Additionally, we calculate a cumulative metric called Machine Learning cumulative performance score (*MLcps*) [88]. The *MLcps* combines the pre-computed performance metrics into a single metric that encapsulates the core aspects of all the metrics. The value of *MLcps* is equal to the area of the polygon created by the metrics in a radar plot. We used the *MLcps Python* package version 0.0.6. The *MLcps* metric was originally designed for comparing and identifying the best-performing ML algorithm. However, herein, we utilize *MLcps* to compare the performance of the same model for the different amounts of training data. As we have a fixed number of pre-calculated metrics (accuracy, sensitivity, specificity, F1 score, and precision), we define *MLcps%* as

$$MLcps\% = \frac{MLcps}{MLcps_{max}} \times 100, \quad (1)$$

where *MLcps* is the area of the pentagon in the radar plot and $MLcps_{max}$ is the area of the pentagon when all the five metrics are 100%.

Furthermore, a permutation test was conducted to assess the statistical significance of the model's performance. By randomly shuffling the class labels 1,000 times and retraining the model on each shuffled set of data, a distribution of accuracy values under chance conditions was obtained for all the experimental settings.

4 Results

In this section, we present and analyze the results of the experiments to evaluate the performance of the HeteroMRI method. The results are reported separately for the experimental settings A, B, C, and D. Furthermore, the insights into limited data scenarios gained through the experiments are discussed afterwards. The classification metrics are provided in box plot and radar plot formats in Figs. 2, 5, 6, and 7. In all box plots, the triangle marker indicates the mean value, and the whiskers represent $1.5 \times IQR$, where IQR is the interquartile range. In settings A, B, and D, the box plots show the distribution of 500 values

for each setting # corresponding to 50 data shuffles that each has been run 10 times. By "setting #", we mean for example $A00, A01, \ldots, A18$. In setting C, the box plot shows the distribution of 5,000 accuracy values for each setting # since there are 10 cases with different protocols chosen as the test set, as explained in Sec. 3.2. The results of permutation tests are plotted as jittered points in the accuracy plots in Figs. 2, 5, 6, and 7 in part (a). For settings A, B, and D, there are 1,000 permutations, while for setting C, there are 10,000 permutations since there are 10 cases with different protocols chosen as the test set.

The radar plots illustrate the metric values for all the setting #s simultaneously, allowing us to perceive the effect of reducing training data size on each metric. In addition, the radar plots are utilized for calculating the *MLcps* values using Eq. (1). The *MLcps%* values for the settings A, B, and C are reported in the Figs. 2(e), 5(e), and 6(c). The radar plots are plotted using the mean metric values; therefore, no standard deviation is reported for the *MLcps%* values. Evaluation of the presented approach across various experimental settings provides several key insights, offering a detailed understanding of its performance and challenges. The results of each experimental setting are presented below.

4.1 Setting *A*

As shown in Fig. 2, setting $A00$, in which the highest number of MRIs (174 training+26 validation+44 test) were included, demonstrates an average accuracy of 93.2±4.3% in the classification of MRIs. The training and testing data in this setting include, in total, 32 different MRI protocols. This underscores the model's adaptability and robustness in handling a diverse range of imaging protocols. Notably, the effect of reducing the training data on the model's performance is inspected here. In $A07$, where the training data (including validation data) is reduced to 36% of $A00$, the accuracy is 89.2±6.4%. Beginning from setting $A08$, where the training data is 25% of $A00$, the average accuracy and sensitivity have a sharp decrease. With much further decreasing the training data (e.g., $A18$ with only two MRIs as training), the accuracy and sensitivity gradually decrease to low values as expected, while specificity tends to remain relatively high. In relatively limited data settings (particularly from $A11$ to $A18$), the boxplots indicate that accuracy values have significant fluctuations across the range.

Sample raw MRIs, the registered MRIs, the obtained WM intensity clusters, and the model's label predictions are provided here for a better insight into the data used for testing the model. Figs. 3 and 4 depict samples of MRIs without and with WM abnormality, respectively. Below each raw MRI, the three-times registered MRI, and the obtained WM intensity cluster (thresholded) are illustrated. Additionally, the model's prediction for the presence of WM abnormalities in setting $A00$ is reported. Each figure includes three true predictions and three false predictions. It is important to note that the shown WM intensity cluster and the registered MRI slice show the same location of the brain (the middle slice). However, these two slices do not directly correspond to the raw MRI slice. This is because the non-linear registration process results in the deformation of the brain. As a result, finding the exact corresponding slices in the raw MRI and the registered one is impractical. Here, the middle slice of the registered MRI is illustrated. The raw MRI slices shown here are selected based on their visual similarity to the corresponding registered

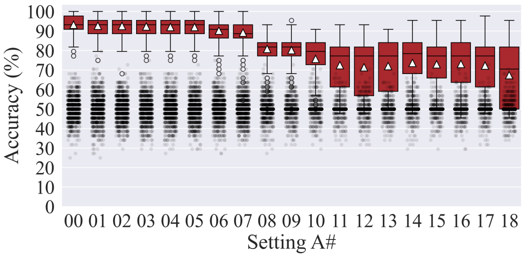

(a)

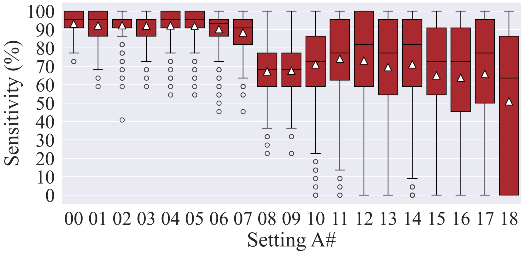

(b)

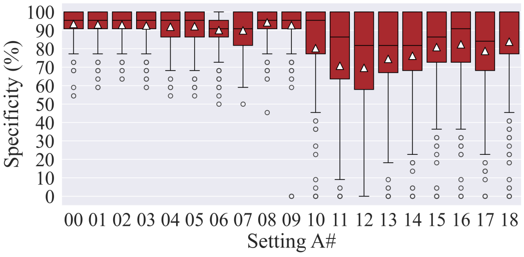

(c)

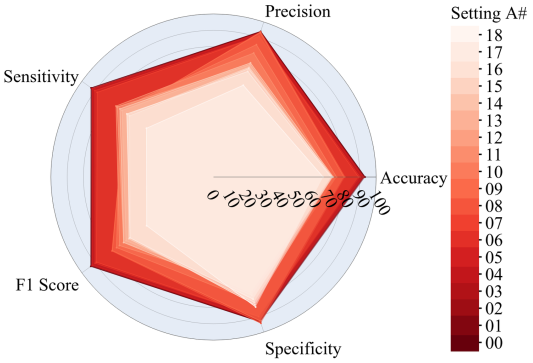

(d)

**Setting**	$A00$	$A01$	$A02$	$A03$	$A04$
MLcps%	85.2	84.0	83.9	83.2	82.6
**Setting**	$A05$	$A06$	$A07$	$A08$	$A09$
MLcps%	82.8	78.8	76.9	63.0	62.0
**Setting**	$A10$	$A11$	$A12$	$A13$	$A14$
MLcps%	53.8	48.3	44.6	44.9	47.3
**Setting**	$A15$	$A16$	$A17$	$A18$	
MLcps%	44.9	44.7	43.4	33.9	

(e)

Figure 2: Classification results of settings $A00$ to $A18$: (a) accuracy, (b) sensitivity, (c) specificity, (d) radar plot of five classification metrics for different setting #s, and (e) *MLcps%* (a cumulative performance score) in % for each setting #. The triangle marker indicates the mean value, and the whiskers represent $1.5 \times IQR$. In each setting # (i.e. $A00$, $A01$, ..., $A18$), the training set size is sequentially reduced by approximately 10% relative to the previous setting, as detailed in Supplementary Table S1. In (a), the jittered points represent the results of 1,000 permutation tests for each setting #. Average accuracy starts at 93.2±4.3% for $A00$, where training includes 200 MRIs, and decreases to 89.2±6.4% in $A07$, with 72 training data, after which it drops with further reductions in training data. A similar trend is observed in the *MLcps%*.

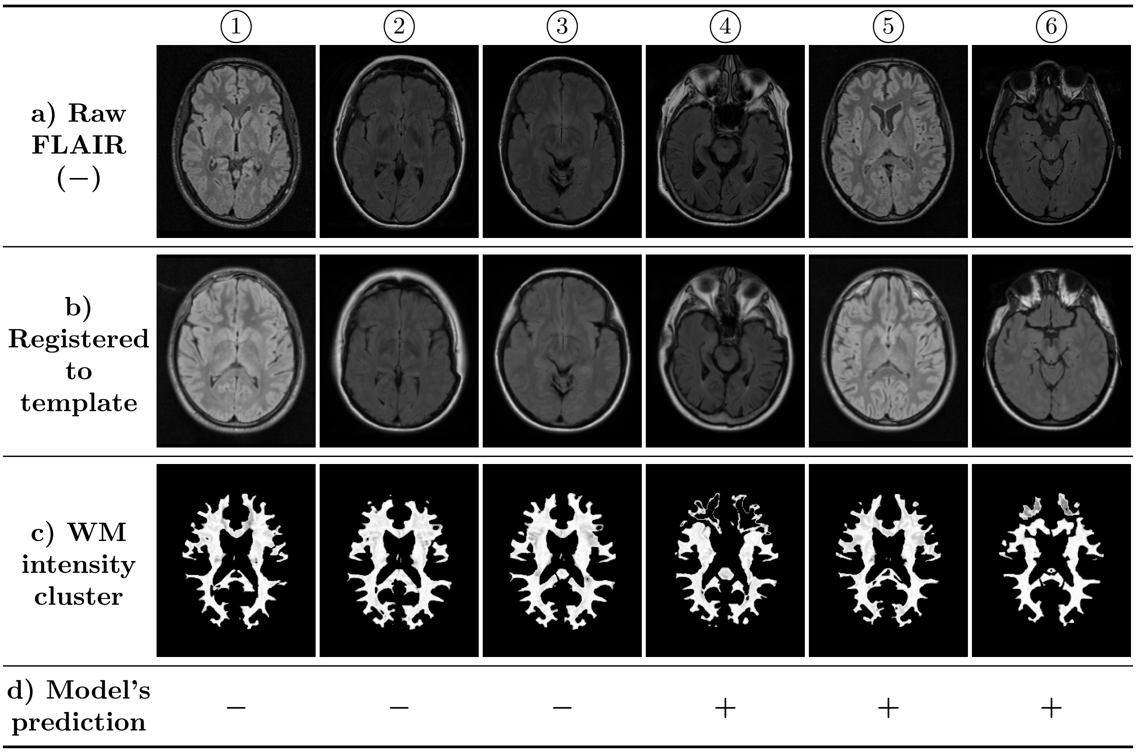

Figure 3: Example of six MRIs (① to ⑥) *without* WM abnormality. a) Raw FLAIR images, b) the MRI three times registered to the MNI template (the middle slice shown), c) the obtained intensity clusters (thresholded) used for testing the model, and d) the prediction of the model in setting $A00$ for the presence of WM abnormalities (+: with WM abnormality, −: without WM abnormality). This figure shows three cases of true negative and three cases of false positive. The WM intensity cluster slice corresponds to the registered MRI slice, but not exactly to the raw MRI slice due to deformations from non-linear registrations. MRIs source: ①, ⑤:[83], ②, ③, ④:[81], ⑥:[80].

slices. The slices of the thresholded WM cluster are presented here solely to illustrate the input provided to the DL model for label prediction.

By investigating the possible reasons for the false predictions in setting $A00$, certain aspects became apparent. In some MRIs, the registration process has not been successful in correctly aligning the brain to the brain template. In such cases, the brain regions are not located in the correct locations after the three-times registration in the MRI preprocessing phase. Therefore, in the WM extraction step, wrong parts of the brain are extracted as WM. This mostly results in a false prediction by the model, especially if the MRI is, in fact, free of WM abnormalities. Examples of incorrect registration are MRIs ④, ⑤, ⑥, ⑩, and ⑫ shown in Figs. 3 and 4. These registration errors are identified by comparing the slices of the registered MRIs with the corresponding slice from the MNI template slice shown in Fig. 1(b). In both cases, the middle slice of the MRI is displayed. In an accurate registration, the brain regions in the registered slice align approximately with those in the template. We noticed that some of the MRIs with incorrect registration are 2D MRIs (such as MRIs ④ and ⑫). The low number of slices in 2D MRIs appears to be a contributing factor to registration problems in some cases. Considering that MRI registration is usually a challenging problem, other factors are also likely to contribute to incorrect registrations; however, they have not been investigated in this study. We refrained from excluding the MRIs with erroneous registration from the study, as our goal was to evaluate HeteroMRI as a fully automatic

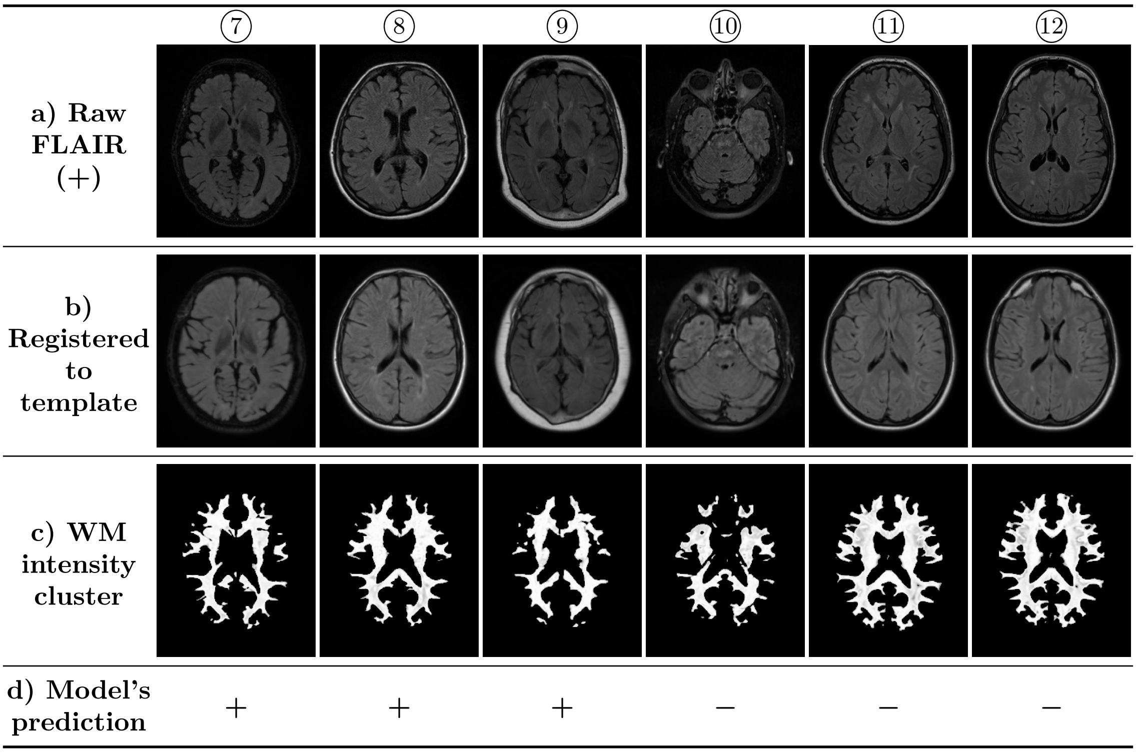

Figure 4: Example of six MRIs (⑦ to ⑫) *with* WM abnormality. a) raw FLAIR images, b) the MRI three times registered to the MNI template (the middle slice shown), c) the obtained intensity clusters (thresholded) used for testing the model, and d) the prediction of the model in setting $A00$ for the presence of WM abnormalities (+: with WM abnormality, −: without WM abnormality). This figure shows three cases of true positive and three cases of false negative. The WM intensity cluster slice corresponds to the registered MRI slice, but not exactly to the raw MRI slice due to deformations from non-linear registrations. MRIs source: ⑦,⑩:[82], ⑧:[79], ⑨:[81], ⑪,⑫:[75].

method without user interference.

4.2 Setting *B*

The setting B, which uses an equal number of MRIs from each MRI protocol, is designed to make the prediction task more challenging for the model. Setting $B00$ has an average accuracy of 89.3±9.1%, as shown in Fig. 5. In terms of the amount of training data, setting $A07$ is the closest match to $B00$. While $B00$ includes 50 training and 10 validation data, $A07$ has a comparable setup with 52 training and 20 validation data. However, setting B is a more challenging scenario than setting A because the model sees an equal number of MRIs from each MRI protocol without being biased by a higher number of images from some protocols. Despite this challenge, the accuracy of $B00$ is almost equal to that of $A07$, which is 89.2±6.4%. This shows the high independence of the presented MRI classification approach on the acquisition protocol of the FLAIR images. With further decrease in the training data, in settings $B01$ to $B04$, all metrics show a gradual decrease in value.

4.3 Setting *C*

The evaluation of setting C is of more importance since it is very close to the real-world use of such a model, as it evaluates the generalizability of HeteroMRI to MRIs from unseen protocols. In this setting, the MRI protocols of the test set are not present in the training data. It resembles a situation in which a clinical center has heterogeneous MRI data and wants to train a classification model with them. Then the model is supposed to classify new MRIs brought by new patients from other centers, acquired most probably with MRI protocols different from those in the training data.

It is noteworthy that in setting C, only 3D MRIs (with 192 or more slices) are included in training and test data, as discussed in the next paragraph. Setting $C00$, as reported in Fig. 6, shows an average accuracy of 92.6±10.6% with 64 MRIs used for training (including validation), which proves the generalizability of the trained model to unseen MRI scanners and protocols. By reducing the data to 46, in $C02$, the model shows an accuracy of 85.7±16.8%. By further decreasing the data to 28 or less, the model's accuracy drops to the chance level (around 50%) in $C04$ to $C06$.

Initially, we used both 3D and 2D MRIs for setting C. However, the model showed a lack of robustness when its generalizability to different protocols was evaluated (i.e., by varying the protocols considered as test data). As we suspected the 2D MRIs (with 70 or fewer slices) as a source of the model's poor performance, we redesigned the setting C to include *only* 3D MRIs. As a result, the performance significantly improved, as reported in the results for setting C. It is important to emphasize that the test data were not fixed for the two versions of setting C, as the model was tested with multiple data shuffles for each setting, with the data being split again in each shuffle. However, from a certain perspective, the decision to exclude 2D MRIs could be interpreted as a form of overfitting to the data characteristics. Additionally, to further investigate the role of 2D MRIs, we also designed a separate setting with *only* 2D MRIs. In this

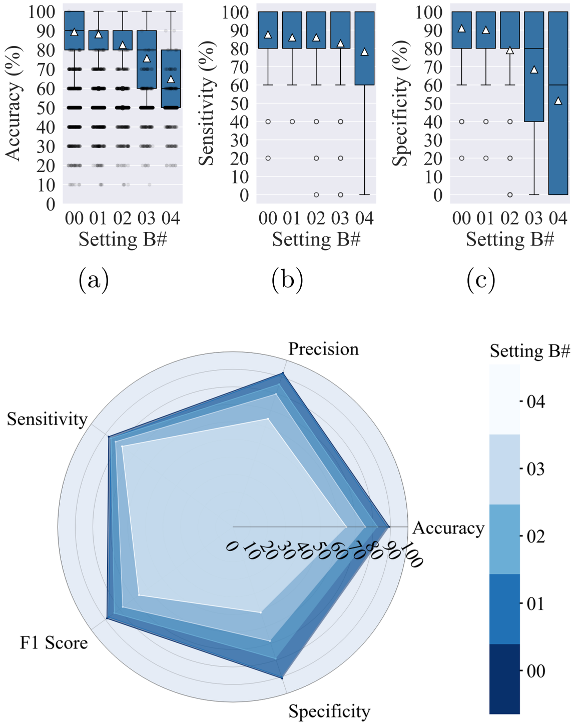

(a) (b) (c)

(d)

**Setting**	$B00$	$B01$	$B02$	$B03$	$B04$
MLcps%	77.9	75.5	65.1	53.4	36.0

(e)

Figure 5: Classification results of settings $B00$ to $B04$: (a) accuracy, (b) sensitivity, (c) specificity, (d) radar plot of five classification metrics for different setting #s, and (e) *MLcps%* (a cumulative performance score) in % for each setting #. The triangle marker indicates the mean value, and the whiskers represent $1.5 \times IQR$. In each setting # (i.e. $B00$, $B01$, ..., $B04$), the training set size is sequentially reduced relative to the previous setting, as detailed in Supplementary Table S2. In (a), the jittered points represent the results of 1,000 permutation tests for each setting #. In setting B, an equal number from 10 different MRI protocols is used for the model. Average accuracy starts at 89.3±9.1% for $B00$, where training includes 60 MRIs, and decreases to 82.5±14.2% in $B02$, with 40 training data, after which it decreases gradually with further reductions in training data. A similar trend is observed in the *MLcps%*.

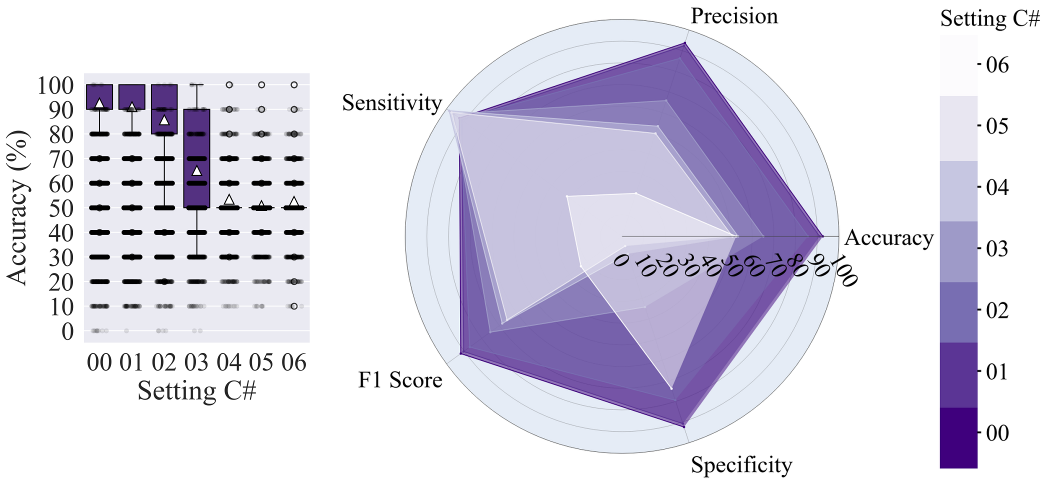

(a) (b)

Setting	Accuracy	Sensitivity	Specificity	***MLcps%***
$C00$	92.58±10.57	92.73±16.94	92.43±15.25	84.02
$C01$	91.09±11.54	91.40±18.54	90.77±17.27	80.93
$C02$	85.74±16.81	92.07±18.46	79.41±32.43	70.49
$C03$	65.14±20.96	96.05±13.51	34.24±45.42	39.07
$C04$	53.50±11.65	98.55±9.38	8.45±26.54	26.42
$C05$	50.93±5.98	97.18±15.83	4.67±20.50	23.55
$C06$	52.61±9.70	31.40±45.12	73.81±42.90	11.53

(c)

Figure 6: Classification results of settings $C00$ to $C06$: (a) accuracy, (b) radar plot of five classification metrics for different setting #s, and (c) four metric values for each setting # (in %, reported as mean±standard deviation). The triangle marker indicates the mean value, and the whiskers represent $1.5 \times IQR$. In each setting # (i.e. $C00$, $C01$, ..., $C06$), the training set size is sequentially reduced relative to the previous setting, as detailed in Supplementary Table S3. In (a), the jittered points represent the results of 1,0000 permutation tests for each setting #. In setting C, the MRI protocols of the test set are unseen by the model during training. Average accuracy starts at 92.6±10.6% for $C00$, where training includes 64 MRIs, and reduces to 85.7±16.8% in $C02$, with 46 training data, after which it drops sharply with further reductions in training data. A similar trend is observed in the *MLcps%*.

case, the model showed notably poor robustness.

4.4 Setting D

Setting D, in which the number of MRI protocols among the data was increased in each setting # while maintaining the same data size (82 MRIs for training), shows the slight negative effect of having higher numbers of protocols, as observed by the overall decrease in accuracy, sensitivity, and specificity (Fig.7). In $D00$ with 4 protocols, the accuracy is 94.80±6.79% while in $D03$ with 10 protocols, the model classifies the MRIs with an accuracy of 90.31±6.36%.

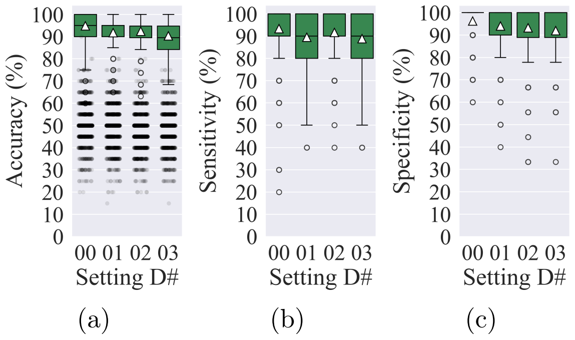

Figure 7: Classification results of settings $D00$ to $D03$: (a) accuracy, (b) sensitivity, and (c) specificity. The triangle marker indicates the mean value, and the whiskers represent $1.5 \times IQR$. In each setting # (i.e. $D00$, $D01$, . . ., $D03$), the number of MRI protocols is sequentially increased relative to the previous setting while maintaining equal training set size (82 MRIs), as detailed in Supplementary Table S4. In (a), the jittered points represent the results of 1,000 permutation tests for each setting #. Average accuracy starts at 94.8±6.8% for $D00$, where training data includes four different MRI protocols, and ends at 90.3±6.3% for $D03$, with 10 MRI protocols in training data.

4.5 Limited data scenarios

A more detailed examination of the impact of reducing training data is presented in Table 2. By comparing settings A, B, and C, a rough correlation can be concluded between the number of MRIs in the training data and the performance of the model for the classification task of this study, regardless of the experimental setting. When the training data (including validation) consists of 60 to 72 MRIs (as in $A07$ and $B00$), an accuracy of 89% is expected. By having 40 to 46 MRIs in the training set (as in $A09, B02$, and $C02$), the accuracy falls roughly within the range of 80% to 86%. Further reducing the training data to the 20 to 36 range (as in $A12, B03$, and $C03$) is associated with a rough accuracy of 76% or less and an F1 score of 78% or less. In this last scenario, the model's performance cannot be considered fully reliable, as the results show a high degree of variability across different runs.

4.6 Comparison with related methods

To further evaluate the performance of HeteroMRI, we applied it on a hold-out set of data and compared the performance with three lesion segmentation methods: DeepWMH [53], WHITE-Net [54], and the Lesion Prediction Algorithm from the Lesion Segmentation Tool (LST-LPA) [55]. These methods are not designed for direct binary classification of MRIs. To enable a comparison, we post-processed their segmentation outputs by binarizing the masks through 3D connected components analysis (using the `ConnectedComponentImageFilter` class from `SimpleITK` [65] *Python* library): if at least one WM lesion consisting of *five* or more 3D-connected voxels was detected, the corresponding MRI scan was labeled as positive. The comparison was conducted using datasets 10 and 11, introduced in Sec. 3.1. The test set includes 20 MRIs in total, with balanced labels and images acquired from nine different MRI protocols. These datasets serve as holdout sets for HeteroMRI and were not used during training or testing in any of the experimental settings. For HeteroMRI, we employed the 500 models trained in setting $A00$. The final prediction label for each test scan was determined by majority vote across these models. The DeepWMH,

Table 2: Average performance results of selected experimental settings (in %, reported as mean±standard deviation). The table shows the effect of reducing training data on the model's performance in settings A, B, and C. For these settings, three cases are reported respectively: 1) with the highest number of training data, 2) with the borderline number of training data after which the performance drops, and 3) with the number of training data that results in relatively low performance. Setting D shows the effect of increasing the number of MRI protocols for the same number of MRI data. AUROC: the area under the receiver operating characteristic curve.

**Setting**	**Data size[1]**		**Protocols[2]**		**Accuracy**	**Sensitivity**	**Specificity**	**F1 score**	**AUROC**
	**Train†**	**Test**	**Train†**	**Test**					
$A00$	200	44	31	14	93.24±4.29	93.05±6.34	93.44±7.38	93.24±4.25	96.76±3.09
$A07$	72	44	17±1	14	89.16±6.39	88.41±11.17	89.91±9.48	88.87±7.00	94.89±4.18
$A09$	42	44	9±1	14	80.17±6.69	67.59±13.29	92.74±14.78	76.83±8.55	91.18±4.30
$A12$	20	44	6±1	14	71.41±13.76	73.14±30.01	69.67±34.80	68.35±23.32	91.91±3.71
$B00$	60	10	10	10	89.32±9.14	87.64±14.31	91.00±14.25	88.88±9.75	95.11±7.24
$B02$	40	10	10	10	82.54±14.17	86.00±15.46	79.08±30.20	83.65±12.09	91.98±8.68
$B03$	30	10	10	10	75.64±16.36	82.76±19.25	68.52±38.97	77.71±14.63	90.26±8.69
$C00$	64	10	8	2	92.58±10.57	92.73±16.94	92.43±15.25	91.91±13.25	99.07±1.06
$C02$	46	10	8	2	85.74±16.81	92.07±18.46	79.41±32.43	86.80±16.27	97.43±2.12
$C03$	36	10	8	2	65.14±20.96	96.05±13.51	34.24±45.42	75.09±14.84	93.34±3.33
$D00$	82	20	4	4	94.80±6.79	93.38±11.91	96.22±7.69	94.38±8.27	99.38±1.86
$D03$	82	20	10	10	90.30±6.33	88.82±11.42	91.95±10.28	90.36±6.85	96.11±5.03

[1] Number of MRIs used in the training and test sets
[2] Number of MRI protocols present in the training and test data
† Including the validation data

WHITE-Net, and LST-LPA are pre-trained, ready-to-use models that do not require retraining. All of the methods require only a FLAIR image per subject. Table 3 reports the accuracy, sensitivity, and specificity for all methods. Based on the results, HeteroMRI outperforms DeepWMH, WHITE-Net, and LST-LPA in accuracy and specificity, while the other methods have a higher sensitivity.

Table 3: Performance of HeteroMRI (setting $A00$) as a binary classifier for detecting brain MRIs with WM abnormalities in a hold-out set, in comparison to three other related methods.

**Method**	**Accuracy**	**Sensitivity**	**Specificity**
**HeteroMRI** (This work)	**70%**	90%	**50%**
**DeepWMH** [53]	55%	**100%**	10%
**WHITE-Net** [54]	55%	**100%**	10%
**LST-LPA** [55]	60%	**100%**	20%

5 Discussion

This study presented HeteroMRI, a DL framework designed for robust binary classification of WM abnormalities across heterogeneous FLAIR MRI datasets. The results show that HeteroMRI is capable of performing the classification while being highly robust to scanner and protocol variabilities. The method

is also adaptable to standardized MRI datasets acquired using a uniform scanner and protocol. The experimental results across settings A–D and the limited-data scenarios offer several insights into the method's robustness, generalizability, and its limitations under varying data constraints.

In Setting A, where training data included all available protocols, HeteroMRI achieved high classification performance. Even by reducing the training data to 72 MRIs, the accuracy remained at 89±6%. This suggests that the model learns effectively from the heterogeneous data, with only a gradual decline in performance as data scarcity increases. In Setting B, where each protocol was equally represented, HeteroMRI maintained comparable accuracy, confirming that the model is highly independent of protocols. In Setting C, which tested generalization to unseen MRI protocols, HeteroMRI continued to perform robustly, provided the training data included a sufficient number of high-resolution (3D) MRIs. This emphasizes the importance of data quality in achieving protocol-invariant performance. Finally, setting D showed the impact of increasing protocol diversity while keeping data volume fixed. A gradual decrease in accuracy was observed with a growing number of MRI protocols, decreasing from 94.8% in D00 (4 protocols) to 90.3% in D03 (10 protocols).

In comparison of the performance on a hold-out set with three segmentation-based methods: DeepWMH [53], WHITE-Net [54], and LST-LPA [55], HeteroMRI outperformed all three methods in terms of both accuracy and specificity, showing a more reliable ability to avoid false positives despite the fact that DeepWMH and WHITE-Net claim to be robust to scanner and protocol variability. This comparison was conducted on a highly heterogeneous hold-out set comprising 20 MRIs from nine different acquisition protocols. Moreover, this comparison result is obtained in a condition in favor of the segmentation methods since we applied a threshold requiring a minimum of five 3D-connected voxels for determining the presence of a lesion in the segmentation outputs. This conservative criterion helps filter out small, potentially false predictions and helps segmentation-based models by reducing false positives. Nevertheless, the segmentation-based models demonstrated higher sensitivity, which can be attributed to their design: these methods are tailored to detect even subtle or small lesion patterns, making them more prone to false positives in borderline cases. These findings generally support HeteroMRI as a method that is highly independent of scanner and protocol variability, generalizes relatively well to unseen protocols, and retains performance under data-limited conditions to some extent.

Despite the promising results of the presented method, it has several limitations to be considered. HeteroMRI faces a challenge with the registration problem with certain MRIs, leading to a false prediction. Registration problems are a well-known issue in MRI analysis. For addressing this challenge with HeteroMRI, a more elaborate registration strategy may reduce the misalignments, or an automatic method may be developed to warn about significant deformations during registration, which can be a sign of wrong registration. These improvements are future work directions for further improving the method. Notably, 2D MRIs were identified as one of the factors that can contribute to registration issues, strongly suggesting the use of 3D MRIs with HeteroMRI. Further factors that cause an incorrect registration were not investigated in this study. Another limitation of HeteroMRI is the high GPU memory requirement, which

is not easily available in every computing server. For each specific use case, one can evaluate the method's performance by downscaling the dimension of input images (and therefore reducing the dimensionality of the CNN) to decrease the required GPU memory. Additionally, the design of HeteroMRI can be upgraded to a multi-channel format, enabling the integration of multiple MRI sequences per subject for enhanced analysis. Moreover, the performance of the method could be studied in case of unbalanced training data. The current classification task of HeteroMRI may be less interesting to apply in clinical practice compared to, for example, segmentation or volumetric measurement methods. However, this paper is supposed to present the HeteroMRI method and evaluate its performance on heterogeneous data. In future work, the methodology is planned to be employed for the disease-specific classification of MRIs based on WM abnormalities by learning from subtle lesion patterns.

6 Conclusion

In this study, we introduced HeteroMRI, a novel approach for robust classification of brain MRIs based on WM abnormalities, specifically designed to handle heterogeneous MRI data acquired from diverse scanners and acquisition protocols. HeteroMRI achieved high accuracy in detecting MRIs with WM abnormalities, even in scenarios with relatively limited data. Furthermore, the method proved to be relatively more generalizable to unseen MRI protocols compared to segmentation-based methods. There is room for enhancing HeteroMRI's performance by improving the registration accuracy or preventing wrong predictions by detecting the erroneous registrations automatically. Our future research will focus on applying this approach to differentiate between unspecific and disease-associated WM lesions, as well as to classify rare demyelinating diseases against their differential diagnoses.

7 Availability of Supporting Source Code and Requirements

Project name: HeteroMRI v1.0

Project home page: https://github.com/ul-mds/HeteroMRI

Operating system(s): Linux-based OS (Ubuntu recommended)

Programming language: Python

Other requirements: TensorFlow v2.x, NVIDIA GPU with CUDA support

License: GNU GPL version 3

8 Data Availability

All the datasets used in this study are either publicly available or are accessible upon request to the respective dataset providers as referenced in Table 1. Snapshots of our code and other data further supporting this work are archived in Software Heritage [89]. In addition, machine learning algorithms have been deposited in the DOME Registry [90].

9 Abbreviations

AI: Artificial Intelligence; ANTs: Advanced Normalization Tools; AUROC: Area Under the Receiver Operating Characteristic Curve; BTH: Baghdad Teaching Hospital; CSF: CerebroSpinal Fluid; CT: Computed Tomography; CNN: Convolutional Neural Network; DL: Deep Learning; TE: Echo Time; FLAIR: Fluid-Attenuated Inversion Recovery; FCM: Fuzzy C-Means; GM: Gray Matter; ICBM: International Consortium for Brain Mapping; TI: Inversion Time; ML: Machine Learning; $MLcps$: Machine Learning Cumulative Performance Score; MRI: Magnetic Resonance Imaging; MNI: Montreal Neurological Institute; MS: Multiple Sclerosis; NifTI: Neuroimaging Informatics Technology Initiative; ReLU: Rectified Linear Unit; TR: Repetition Time; RFCM: Robust Fuzzy C-Means; 3D: three-dimensional; WM: White Matter.

10 Competing Interests

The authors declare that they have no competing interests

11 Authors' Contributions

M.A. (Methodology, Formal Analysis, Software), N.SH. (Conceptualization, Methodology, Writing – Original Draft), P.L.B. (Methodology), N.SC. (Supervision), J.L. (Supervision, Data Curation), C.C.B. (Supervision), W.K. (Supervision, Data Curation, Project Investigation, Funding Acquisition), T.K. (Supervision, Project Investigation, Funding Acquisition). All authors contributed to reviewing and editing the manuscript.

12 Funding

The authors acknowledge the financial support by the Federal Ministry of Health of Germany in project LeukoExpert (grant no. ZMVI1-2520DAT94), the State Ministry for Education and Research of Germany in the project Tag-White (grant no. 100602109), and the Federal Ministry of Research, Technology and Space of Germany and by Sächsische Staatsministerium für Wissenschaft, Kultur und Tourismus in the programme Center of Excellence for AI-research "Center for Scalable Data Analytics and Artificial Intelligence Dresden/Leipzig", project identification number: ScaDS.AI. Supported by the Open Access Publication Fund of Leipzig University.

13 Acknowledgments

The authors sincerely thank Dr. Sina Sadeghi for his insightful comments on the manuscript. The authors are grateful to the Center for Information Services and High-Performance Computing [Zentrum für Informationsdienste und Hochleistungsrechnen (ZIH)] at TU Dresden for providing its facilities for high-throughput calculations. Data were provided in part by OASIS Longitudinal Multimodal Neuroimaging: Principal Investigators: T. Benzinger, D. Marcus, J. Morris; NIH P30 AG066444, P50 AG00561, P30

NS09857781, P01 AG026276, P01 AG003991, R01 AG043434, UL1 TR000448, R01 EB009352. AV-45 doses were provided by Avid Radiopharmaceuticals, a wholly owned subsidiary of Eli Lilly. Part of the Data collection and sharing for the Alzheimer's Disease Neuroimaging Initiative (ADNI) is funded by the National Institute on Aging (National Institutes of Health Grant U19 AG024904). The grantee organization is the Northern California Institute for Research and Education. In the past, ADNI has also received funding from the National Institute of Biomedical Imaging and Bioengineering, the Canadian Institutes of Health Research, and private sector contributions through the Foundation for the National Institutes of Health (FNIH) including generous contributions from the following: AbbVie, Alzheimer's Association; Alzheimer's Drug Discovery Foundation; Araclon Biotech; BioClinica, Inc.; Biogen; Bristol-Myers Squibb Company; CereSpir, Inc.; Cogstate; Eisai Inc.; Elan Pharmaceuticals, Inc.; Eli Lilly and Company; EuroImmun; F. Hoffmann-La Roche Ltd and its affiliated company Genentech, Inc.; Fujirebio; GE Healthcare; IXICO Ltd.; Janssen Alzheimer Immunotherapy Research & Development, LLC.; Johnson & Johnson Pharmaceutical Research &Development LLC.; Lumosity; Lundbeck; Merck & Co., Inc.; Meso Scale Diagnostics, LLC.; NeuroRx Research; Neurotrack Technologies; Novartis Pharmaceuticals Corporation; Pfizer Inc.; Piramal Imaging; Servier; Takeda Pharmaceutical Company; and Transition Therapeutics. Part of the data collection and sharing for this project was provided by the International Consortium for Brain Mapping (ICBM; Principal Investigator: John Mazziotta, MD, PhD). ICBM funding was provided by the National Institute of Biomedical Imaging and BioEngineering. ICBM data are disseminated by the Laboratory of Neuro Imaging at the University of Southern California. Part of the data was provided in collaboration with The Observatoire Français de la Sclérose en Plaques (OFSEP), which is supported by a grant provided by the French State and handled by the "Agence Nationale de la Recherche," within the framework of the "Investments for the Future" program, under the reference ANR-10-COHO-002, by the Eugène Devic EDMUS Foundation against multiple sclerosis and by the ARSEP Foundation. Part of the data used in the preparation of this article was obtained on 2025-04-05 from the Parkinson's Progression Markers Initiative (PPMI) database (www.ppmi-info.org/access-dataspecimens/download-data), RRID:SCR_006431. For up-to-date information on the study, visit www.ppmi-info.org. PPMI—a public-private partnership—is funded by the Michael J. Fox Foundation for Parkinson's Research, and funding partners, including 4D Pharma, Abbvie, AcureX, Allergan, Amathus Therapeutics, Aligning Science Across Parkinson's, AskBio, Avid Radiopharmaceuticals, BIAL, BioArctic, Biogen, Biohaven, BioLegend, BlueRock Therapeutics, Bristol-Myers Squibb, Calico Labs, Capsida Biotherapeutics, Celgene, Cerevel Therapeutics, Coave Therapeutics, DaCapo Brainscience, Denali, Edmond J. Safra Foundation, Eli Lilly, Gain Therapeutics, GE HealthCare, Genentech, GSK, Golub Capital, Handl Therapeutics, Insitro, Jazz Pharmaceuticals, Johnson & Johnson Innovative Medicine, Lundbeck, Merck, Meso Scale Discovery, Mission Therapeutics, Neurocrine Biosciences, Neuron23, Neuropore, Pfizer, Piramal, Prevail Therapeutics, Roche, Sanofi, Servier, Sun Pharma Advanced Research Company, Takeda, Teva, UCB, Vanqua Bio, Verily, Voyager Therapeutics, the Weston Family Foundation and Yumanity Therapeutics.

14 Additional Files

Supplementary Table S1. The experimental setting A and details of the MRI data used.

Supplementary Table S2. The experimental setting B and details of the MRI data used.

Supplementary Table S3. The experimental setting C and details of the MRI data used.

Supplementary Table S4. The experimental setting D and details of the MRI data used.

Supplementary Fig. S1. Mutual information metric between each MRI and the MNI template after each registration.

Supplementary Fig. S2. Average required time for preprocessing and intensity clustering of five sample MRI dimensions.

Supplementary Fig. S3. Average required time for training the CNN model of each experimental setting.

Supplementary Fig. S4. The WM intensity cluster of a sample MRI (a) before and (b) after applying a threshold value of 0.5. The normalized histograms of their 99% upper percentile are shown in (c) and (d), respectively.

References

[1] T. Yousaf, G. Dervenoulas, & M. Politis (2018) *Chapter two - advances in MRI methodology*, M. Politis (Ed.) *Imaging in Movement Disorders: Imaging Methodology and Applications in Parkinson's Disease*, vol. 141 of *International Review of Neurobiology*, pp. 31–76, Academic Press

[2] F. Agosta, S. Galantucci, & M. Filippi (2017) *Advanced magnetic resonance imaging of neurodegenerative diseases*, Neurological Sciences, **38(1)**:pp. 41–51

[3] M. Filippi, P. Preziosa, B. L. Banwell, F. Barkhof, O. Ciccarelli, N. De Stefano, J. J. G. Geurts, F. Paul, D. S. Reich, A. T. Toosy, A. Traboulsee, M. P. Wattjes, T. A. Yousry, A. Gass, C. Lubetzki, B. G. Weinshenker, & M. A. Rocca (2019) *Assessment of lesions on magnetic resonance imaging in multiple sclerosis: practical guidelines*, Brain, **142(7)**:pp. 1858–1875

[4] C. Domínguez-Fernández, J. Egiguren-Ortiz, J. Razquin, M. Gómez-Galán, L. De las Heras-García, E. Paredes-Rodríguez, E. Astigarraga, C. Miguélez, & G. Barreda-Gómez (2023) *Review of technological challenges in personalised medicine and early diagnosis of neurodegenerative disorders*, International Journal of Molecular Sciences, **24(4)**

[5] A. S. Panayides, A. Amini, N. D. Filipovic, A. Sharma, S. A. Tsaftaris, A. Young, D. Foran, N. Do, S. Golemati, T. Kurc, K. Huang, K. S. Nikita, B. P. Veasey, M. Zervakis, J. H. Saltz, & C. S. Pattichis (2020) *AI in medical imaging informatics: Current challenges and future directions*, IEEE Journal of Biomedical and Health Informatics, **24(7)**:pp. 1837–1857

[6] M. L. Giger (2018) *Machine learning in medical imaging*, Journal of the American College of Radiology, **15(3, Part B)**:pp. 512–520, data Science: Big Data Machine Learning and Artificial Intelligence

[7] N. Garg, M. S. Choudhry, & R. M. Bodade (2023) *A review on Alzheimer's disease classification from normal controls and mild cognitive impairment using structural MR images*, Journal of Neuroscience Methods, **384**:p. 109745

[8] J. Wen, E. Thibeau-Sutre, M. Diaz-Melo, J. Samper-González, A. Routier, S. Bottani, D. Dormont, S. Durrleman, N. Burgos, & O. Colliot (2020) *Convolutional neural networks for classification of Alzheimer's disease: Overview and reproducible evaluation*, Medical Image Analysis, **63**:p. 101694

[9] A. Kursad Poyraz, S. Dogan, E. Akbal, & T. Tuncer (2022) *Automated brain disease classification using exemplar deep features*, Biomedical Signal Processing and Control, **73**:p. 103448

[10] D. García-Lorenzo, S. Francis, S. Narayanan, D. L. Arnold, & D. Louis Collins (2013) *Review of automatic segmentation methods of multiple sclerosis white matter lesions on conventional magnetic resonance imaging*, Medical Image Analysis, **17(1)**:pp. 1–18

[11] O. Cetin, V. Seymen, & U. Sakoglu (2020) *Multiple sclerosis lesion detection in multimodal MRI using simple clustering-based segmentation and classification*, Informatics in Medicine Unlocked, **20**:p. 100409

[12] J. Amin, M. Sharif, M. Yasmin, & S. L. Fernandes (2020) *A distinctive approach in brain tumor detection and classification using MRI*, Pattern Recognition Letters, **139**:pp. 118–127

[13] M. A. Naser & M. J. Deen (2020) *Brain tumor segmentation and grading of lower-grade glioma using deep learning in MRI images*, Computers in Biology and Medicine, **121**:p. 103758

[14] S. Zhang, S. Xu, L. Tan, H. Wang, & J. Meng (2021) *Stroke lesion detection and analysis in MRI images based on deep learning*, Journal of Healthcare Engineering, **2021**:p. 5524769

[15] Y. Kabir, M. Dojat, B. Scherrer, F. Forbes, & C. Garbay (2007) *Multimodal MRI segmentation of ischemic stroke lesions*, *2007 29th Annual International Conference of the IEEE Engineering in Medicine and Biology Society*, pp. 1595–1598

[16] H. Peng, W. Gong, C. F. Beckmann, A. Vedaldi, & S. M. Smith (2021) *Accurate brain age prediction with lightweight deep neural networks*, Medical Image Analysis, **68**:p. 101871

[17] H. Sajedi & N. Pardakhti (2019) *Age prediction based on brain MRI image: A survey*, Journal of Medical Systems, **43(8)**:p. 279

[18] J. West, J. B. M. Warntjes, & P. Lundberg (2012) *Novel whole brain segmentation and volume estimation using quantitative MRI*, European Radiology, **22(5)**:pp. 998–1007

[19] S. Valverde, A. Oliver, E. Roura, S. González-Villà, D. Pareto, J. C. Vilanova, L. Ramió-Torrentà, Àlex Rovira, & X. Lladó (2017) *Automated tissue segmentation of MR brain images in the presence of white matter lesions*, Medical Image Analysis, **35**:pp. 446–457

[20] N. Andrade, F. A. Faria, & F. A. M. Cappabianco (2018) *A practical review on medical image registration: From rigid to deep learning based approaches*, *2018 31st SIBGRAPI Conference on Graphics, Patterns and Images (SIBGRAPI)*, pp. 463–470

[21] J. Kleesiek, G. Urban, A. Hubert, D. Schwarz, K. Maier-Hein, M. Bendszus, & A. Biller (2016) *Deep MRI brain extraction: A 3D convolutional neural network for skull stripping*, NeuroImage, **129**:pp. 460–469

[22] P. Kalavathi & V. B. S. Prasath (2016) *Methods on skull stripping of MRI head scan images—a review*, Journal of Digital Imaging, **29(3)**:pp. 365–379

[23] A. Kaur & G. Dong (2023) *A complete review on image denoising techniques for medical images*, Neural Processing Letters, **55(6)**:pp. 7807–7850

[24] J. Mohan, V. Krishnaveni, & Y. Guo (2014) *A survey on the magnetic resonance image denoising methods*, Biomedical Signal Processing and Control, **9**:pp. 56–69

[25] J. V. Manjón, J. Carbonell-Caballero, J. J. Lull, G. García-Martí, L. Martí-Bonmatí, & M. Robles (2008) *MRI denoising using non-local means*, Medical Image Analysis, **12(4)**:pp. 514–523

[26] M. Shah, Y. Xiao, N. Subbanna, S. Francis, D. L. Arnold, D. L. Collins, & T. Arbel (2011) *Evaluating intensity normalization on MRIs of human brain with multiple sclerosis*, Medical Image Analysis, **15(2)**:pp. 267–282

[27] C. Loizou, M. Pantziaris, I. Seimenis, & C. Pattichis (2009) *Brain MR image normalization in texture analysis of multiple sclerosis*, *2009 9th International Conference on Information Technology and Applications in Biomedicine*, pp. 1–5

[28] N. J. Tustison, B. B. Avants, P. A. Cook, Y. Zheng, A. Egan, P. A. Yushkevich, & J. C. Gee (2010) *N4ITK: Improved N3 bias correction*, IEEE Transactions on Medical Imaging, **29(6)**:pp. 1310–1320

[29] M. Joliot & B. Mazoyer (1993) *Three-dimensional segmentation and interpolation of magnetic resonance brain images*, IEEE Transactions on Medical Imaging, **12(2)**:pp. 269–277

[30] R. Shinohara, J. Oh, G. Nair, P. Calabresi, C. Davatzikos, J. Doshi, R. Henry, G. Kim, K. Linn, N. Papinutto, D. Pelletier, D. Pham, D. Reich, W. Rooney, S. Roy, W. Stern, S. Tummala, F. Yousuf, A. Zhu, N. Sicotte, R. Bakshi, & the NAIMS Cooperative (2017) *Volumetric analysis from a harmonized multisite brain MRI study of a single subject with multiple sclerosis*, American Journal of Neuroradiology, **38(8)**:pp. 1501–1509

[31] J.-P. Fortin, N. Cullen, Y. I. Sheline, W. D. Taylor, I. Aselcioglu, P. A. Cook, P. Adams, C. Cooper, M. Fava, P. J. McGrath, M. McInnis, M. L. Phillips, M. H. Trivedi, M. M. Weissman, & R. T. Shinohara (2018) *Harmonization of cortical thickness measurements across scanners and sites*, NeuroImage, **167**:pp. 104–120

[32] J.-P. Fortin, D. Parker, B. Tunç, T. Watanabe, M. A. Elliott, K. Ruparel, D. R. Roalf, T. D. Satterthwaite, R. C. Gur, R. E. Gur, R. T. Schultz, R. Verma, & R. T. Shinohara (2017) *Harmonization of multi-site diffusion tensor imaging data*, NeuroImage, **161**:pp. 149–170

[33] J.-P. Fortin, E. M. Sweeney, J. Muschelli, C. M. Crainiceanu, & R. T. Shinohara (2016) *Removing inter-subject technical variability in magnetic resonance imaging studies*, NeuroImage, **132**:pp. 198–212

[34] C. Marzi, M. Giannelli, A. Barucci, C. Tessa, M. Mascalchi, & S. Diciotti (2024) *Efficacy of MRI data harmonization in the age of machine learning: a multicenter study across 36 datasets*, Scientific Data, **11(1)**:p. 115

[35] R. Pomponio, G. Erus, M. Habes, J. Doshi, D. Srinivasan, E. Mamourian, V. Bashyam, I. M. Nasrallah, T. D. Satterthwaite, Y. Fan, L. J. Launer, C. L. Masters, P. Maruff, C. Zhuo, H. Völzke, S. C.

Johnson, J. Fripp, N. Koutsouleris, D. H. Wolf, R. Gur, R. Gur, J. Morris, M. S. Albert, H. J. Grabe, S. M. Resnick, R. N. Bryan, D. A. Wolk, R. T. Shinohara, H. Shou, & C. Davatzikos (2020) *Harmonization of large MRI datasets for the analysis of brain imaging patterns throughout the lifespan*, NeuroImage, **208**:p. 116450

[36] J. Radua, E. Vieta, R. Shinohara, P. Kochunov, Y. Quidé, M. J. Green, C. S. Weickert, T. Weickert, J. Bruggemann, T. Kircher, I. Nenadić, M. J. Cairns, M. Seal, U. Schall, F. Henskens, J. M. Fullerton, B. Mowry, C. Pantelis, R. Lenroot, V. Cropley, C. Loughland, R. Scott, D. Wolf, T. D. Satterthwaite, Y. Tan, K. Sim, F. Piras, G. Spalletta, N. Banaj, E. Pomarol-Clotet, A. Solanes, A. Albajes-Eizagirre, E. J. Canales-Rodríguez, S. Sarro, A. Di Giorgio, A. Bertolino, M. Stäblein, V. Oertel, C. Knöchel, S. Borgwardt, S. du Plessis, J.-Y. Yun, J. S. Kwon, U. Dannlowski, T. Hahn, D. Grotegerd, C. Alloza, C. Arango, J. Janssen, C. Díaz-Caneja, W. Jiang, V. Calhoun, S. Ehrlich, K. Yang, N. G. Cascella, Y. Takayanagi, A. Sawa, A. Tomyshev, I. Lebedeva, V. Kaleda, M. Kirschner, C. Hoschl, D. Tomecek, A. Skoch, T. van Amelsvoort, G. Bakker, A. James, A. Preda, A. Weideman, D. J. Stein, F. Howells, A. Uhlmann, H. Temmingh, C. López-Jaramillo, A. Díaz-Zuluaga, L. Fortea, E. Martinez-Heras, E. Solana, S. Llufriu, N. Jahanshad, P. Thompson, J. Turner, T. van Erp, D. Glahn, G. Pearlson, E. Hong, A. Krug, V. Carr, P. Tooney, G. Cooper, P. Rasser, P. Michie, S. Catts, R. Gur, R. Gur, F. Yang, F. Fan, J. Chen, H. Guo, S. Tan, Z. Wang, H. Xiang, F. Piras, F. Assogna, R. Salvador, P. McKenna, A. Bonvino, M. King, S. Kaiser, D. Nguyen, & J. Pineda-Zapata (2020) *Increased power by harmonizing structural MRI site differences with the ComBat batch adjustment method in ENIGMA*, NeuroImage, **218**:p. 116956

[37] R. Kushol, P. Parnianpour, A. H. Wilman, S. Kalra, & Y.-H. Yang (2023) *Effects of MRI scanner manufacturers in classification tasks with deep learning models*, Scientific Reports, **13(1)**:p. 16791

[38] A. P. De Rosa, M. Benedetto, S. Tagliaferri, F. Bardozzo, A. D'Ambrosio, A. Bisecco, A. Gallo, M. Cirillo, R. Tagliaferri, & F. Esposito (2024) *Consensus of algorithms for lesion segmentation in brain MRI studies of multiple sclerosis*, Scientific Reports, **14(1)**:p. 21348

[39] T. J. Littlejohns, J. Holliday, L. M. Gibson, S. Garratt, N. Oesingmann, F. Alfaro-Almagro, J. D. Bell, C. Boultwood, R. Collins, M. C. Conroy, N. Crabtree, N. Doherty, A. F. Frangi, N. C. Harvey, P. Leeson, K. L. Miller, S. Neubauer, S. E. Petersen, J. Sellors, S. Sheard, S. M. Smith, C. L. M. Sudlow, P. M. Matthews, & N. E. Allen (2020) *The UK Biobank imaging enhancement of 100,000 participants: rationale, data collection, management and future directions*, Nature Communications, **11(1)**:p. 2624

[40] G. Mårtensson, D. Ferreira, T. Granberg, L. Cavallin, K. Oppedal, A. Padovani, I. Rektorova, L. Bonanni, M. Pardini, M. G. Kramberger, J.-P. Taylor, J. Hort, J. Snædal, J. Kulisevsky, F. Blanc, A. Antonini, P. Mecocci, B. Vellas, M. Tsolaki, I. Kłoszewska, H. Soininen, S. Lovestone, A. Simmons, D. Aarsland, & E. Westman (2020) *The reliability of a deep learning model in clinical out-of-distribution MRI data: A multicohort study*, Med Image Anal, **66**:p. 101714

[41] L. G. Nyúl & J. K. Udupa (1999) *On standardizing the MR image intensity scale*, Magnetic Resonance in Medicine, **42(6)**:pp. 1072–1081

[42] M. E. Torbati, D. S. Minhas, C. M. Laymon, P. Maillard, J. D. Wilson, C.-L. Chen, C. M. Crainiceanu, C. S. DeCarli, S. J. Hwang, & D. L. Tudorascu (2023) *MISPEL: A supervised deep learning harmonization method for multi-scanner neuroimaging data*, Medical Image Analysis, **89**:p. 102926

[43] B. E. Dewey, L. Zuo, A. Carass, Y. He, Y. Liu, E. M. Mowry, S. Newsome, J. Oh, P. A. Calabresi, & J. L. Prince (2020) *A disentangled latent space for cross-site MRI harmonization*, A. L. Martel, P. Abolmaesumi, D. Stoyanov, D. Mateus, M. A. Zuluaga, S. K. Zhou, D. Racoceanu, & L. Joskowicz (Eds.) *Medical Image Computing and Computer Assisted Intervention – MICCAI 2020*, pp. 720–729, Springer International Publishing, Cham

[44] S. Liu & P.-T. Yap (2024) *Learning multi-site harmonization of magnetic resonance images without traveling human phantoms*, Communications Engineering, **3(1)**:p. 6

[45] L. Zuo, B. E. Dewey, Y. Liu, Y. He, S. D. Newsome, E. M. Mowry, S. M. Resnick, J. L. Prince, & A. Carass (2021) *Unsupervised MR harmonization by learning disentangled representations using information bottleneck theory*, NeuroImage, **243**:p. 118569

[46] F. Hu, A. Lucas, A. A. Chen, K. Coleman, H. Horng, R. W. Ng, N. J. Tustison, K. A. Davis, H. Shou, M. Li, R. T. Shinohara, & T. A. D. N. Initiative (2023) *Deepcombat: A statistically motivated, hyperparameter-robust, deep learning approach to harmonization of neuroimaging data*, bioRxiv

[47] F. Hu, A. A. Chen, H. Horng, V. Bashyam, C. Davatzikos, A. Alexander-Bloch, M. Li, H. Shou, T. D. Satterthwaite, M. Yu, & R. T. Shinohara (2023) *Image harmonization: A review of statistical and deep learning methods for removing batch effects and evaluation metrics for effective harmonization*, NeuroImage, **274**:p. 120125

[48] L. L. Resende, A. R. B. de Paiva, F. Kok, C. da Costa Leite, & L. T. Lucato (2019) *Adult leukodystrophies: a step-by-step diagnostic approach*, Radiographics, **39(1)**:pp. 153–168

[49] W. Zhu, H. Huang, Y. Zhou, F. Shi, H. Shen, R. Chen, R. Hua, W. Wang, S. Xu, & X. Luo (2022) *Automatic segmentation of white matter hyperintensities in routine clinical brain mri by 2D VB-Net: A large-scale study*, Frontiers in Aging Neuroscience, **Volume 14 - 2022**

[50] S. Mu, W. Lu, G. Yu, L. Zheng, & J. Qiu (2024) *Deep learning-based grading of white matter hyperintensities enables identification of potential markers in multi-sequence mri data*, Computer Methods and Programs in Biomedicine, **243**:p. 107904

[51] M. Kuwabara, F. Ikawa, S. Nakazawa, S. Koshino, D. Ishii, H. Kondo, T. Hara, Y. Maeda, R. Sato, T. Kaneko, S. Maeyama, Y. Shimahara, & N. Horie (2024) *Artificial intelligence for volumetric measurement of cerebral white matter hyperintensities on thick-slice fluid-attenuated inversion recovery (FLAIR) magnetic resonance images from multiple centers*, Scientific Reports, **14(1)**:p. 10104

[52] R. Guerrero, C. Qin, O. Oktay, C. Bowles, L. Chen, R. Joules, R. Wolz, M. Valdés-Hernández, D. Dickie, J. Wardlaw, & D. Rueckert (2018) *White matter hyperintensity and stroke lesion segmentation and differentiation using convolutional neural networks*, NeuroImage: Clinical, **17**:pp. 918–934

[53] C. Liu, Z. Zhuo, L. Qu, Y. Jin, T. Hua, J. Xu, G. Tan, Y. Li, Y. Duan, T. Wang, Z. Zhang, Y. Zhang, R. Chen, P. Yu, P. Zhang, Y. Shi, J. Zhang, D. Tian, R. Li, X. Zhang, F. Shi, Y. Wang, J. Jiang, A. Carass, Y. Liu, & C. Ye (2024) *DeepWMH: A deep learning tool for accurate white matter hyper-*

intensity segmentation without requiring manual annotations for training, Science Bulletin, **69(7)**:pp. 872–875

[54] C. Cathala, F. Kherif, J.-P. Thiran, A. Bussy, & B. Draganski (2025) *WHITE-Net : White matter hyperintensities tissue extraction using deep learning network*, medRxiv

[55] P. Schmidt (2017) *Bayesian inference for structured additive regression models for large-scale problems with applications to medical imaging*, Ph.D. thesis

[56] P. Tran, U. Thoprakarn, E. Gourieux, C. L. dos Santos, E. Cavedo, N. Guizard, F. Cotton, P. Krolak-Salmon, C. Delmaire, D. Heidelberg, N. Pyatigorskaya, S. Ströer, D. Dormont, J.-B. Martini, & M. Chupin (2022) *Automatic segmentation of white matter hyperintensities: validation and comparison with state-of-the-art methods on both multiple sclerosis and elderly subjects*, NeuroImage: Clinical, **33**:p. 102940

[57] X. Tu, J. Gao, C. Zhu, J.-Z. Cheng, Z. Ma, X. Dai, & M. Xie (2016) *MR image segmentation and bias field estimation based on coherent local intensity clustering with total variation regularization*, Medical & Biological Engineering & Computing, **54(12)**:pp. 1807–1818

[58] D. Kumar, R. K. Agrawal, & P. Kumar (2022) *Bias-corrected intuitionistic fuzzy c-means with spatial neighborhood information approach for human brain MRI image segmentation*, IEEE Transactions on Fuzzy Systems, **30(3)**:pp. 687–700

[59] A. Khosravanian, M. Rahmanimanesh, P. Keshavarzi, & S. Mozaffari (2021) *Fast level set method for glioma brain tumor segmentation based on superpixel fuzzy clustering and lattice boltzmann method*, Computer Methods and Programs in Biomedicine, **198**:p. 105809

[60] L. Szilágyi, S. M. Szilágyi, B. Benyó, & Z. Benyó (2011) *Intensity inhomogeneity compensation and segmentation of MR brain images using hybrid c-means clustering models*, Biomedical Signal Processing and Control, **6(1)**:pp. 3–12, biomedical signal processing(Extended selected papers from the 7th IFAC Symposium on Modelling and Control in Biomedical Systems(MCBMS'09))

[61] V. Fonov, A. Evans, R. McKinstry, C. Almli, & D. Collins (2009) *Unbiased nonlinear average age-appropriate brain templates from birth to adulthood*, NeuroImage, **47**:p. S102, organization for Human Brain Mapping 2009 Annual Meeting

[62] V. Fonov, A. C. Evans, K. Botteron, C. R. Almli, R. C. McKinstry, & D. L. Collins (2011) *Unbiased average age-appropriate atlases for pediatric studies*, NeuroImage, **54(1)**:pp. 313–327

[63] D. L. Pham (2001) *Spatial models for fuzzy clustering*, Computer Vision and Image Understanding, **84(2)**:pp. 285–297

[64] X. Li, P. S. Morgan, J. Ashburner, J. Smith, & C. Rorden (2016) *The first step for neuroimaging data analysis: DICOM to NIfTI conversion*, Journal of Neuroscience Methods, **264**:pp. 47–56

[65] B. Lowekamp, D. Chen, L. Ibanez, & D. Blezek (2013) *The design of SimpleITK*, Frontiers in Neuroinformatics, **7**:p. 45

[66] J. C. Mazziotta, A. W. Toga, A. Evans, P. Fox, & J. Lancaster (1995) *A probabilistic atlas of the human brain: Theory and rationale for its development: The International Consortium for Brain Mapping (ICBM)*, NeuroImage, **2(2, Part A)**:pp. 89–101

[67] B. B. Avants, N. Tustison, G. Song, et al. (2009) *Advanced normalization tools (ANTs)*, Insight j, **2(365)**:pp. 1–35

[68] S. Yang, Z. Yang, K. Fischer, K. Zhong, J. Stadler, F. Godenschweger, J. Steiner, H.-J. Heinze, H.-G. Bernstein, B. Bogerts, C. Mawrin, D. Reutens, O. Speck, & M. Walter (2013) *Integration of ultra-high field MRI and histology for connectome based research of brain disorders*, Frontiers in Neuroanatomy, **7**

[69] J. M. Huntenburg, C. J. Steele, & P.-L. Bazin (2018) *Nighres: processing tools for high-resolution neuroimaging*, GigaScience, **7(7)**:p. giy082

[70] L. R. Dice (1945) *Measures of the amount of ecologic association between species*, Ecology, **26(3)**:pp. 297–302

[71] H. Zunair, A. Rahman, N. Mohammed, & J. P. Cohen (2020) *Uniformizing techniques to process CT scans with 3D CNNs for tuberculosis prediction*, I. Rekik, E. Adeli, S. H. Park, & M. d. C. Valdés Hernández (Eds.) *Predictive Intelligence in Medicine*, pp. 156–168, Springer International Publishing, Cham

[72] D. Maturana & S. Scherer (2015) *VoxNet: A 3D convolutional neural network for real-time object recognition*, *2015 IEEE/RSJ International Conference on Intelligent Robots and Systems (IROS)*, pp. 922–928

[73] S. Ioffe & C. Szegedy (2015) *Batch normalization: Accelerating deep network training by reducing internal covariate shift*, F. Bach & D. Blei (Eds.) *Proceedings of the 32nd International Conference on Machine Learning*, vol. 37 of *Proceedings of Machine Learning Research*, pp. 448–456, PMLR, Lille, France

[74] D. P. Kingma & J. Ba (2017) *Adam: A method for stochastic optimization*, arXiv preprint arXiv:1412.6980

[75] A. Carass, S. Roy, A. Jog, J. L. Cuzzocreo, E. Magrath, A. Gherman, J. Button, J. Nguyen, F. Prados, C. H. Sudre, M. Jorge Cardoso, N. Cawley, O. Ciccarelli, C. A. Wheeler-Kingshott, S. Ourselin, L. Catanese, H. Deshpande, P. Maurel, O. Commowick, C. Barillot, X. Tomas-Fernandez, S. K. Warfield, S. Vaidya, A. Chunduru, R. Muthuganapathy, G. Krishnamurthi, A. Jesson, T. Arbel, O. Maier, H. Handels, L. O. Iheme, D. Unay, S. Jain, D. M. Sima, D. Smeets, M. Ghafoorian, B. Platel, A. Birenbaum, H. Greenspan, P.-L. Bazin, P. A. Calabresi, C. M. Crainiceanu, L. M. Ellingsen, D. S. Reich, J. L. Prince, & D. L. Pham (2017) *Longitudinal multiple sclerosis lesion segmentation: Resource and challenge*, NeuroImage, **148**:pp. 77–102

[76] Ž. Lesjak, A. Galimzianova, A. Koren, M. Lukin, F. Pernuš, B. Likar, & Ž. Špiclin (2018) *A novel public MR image dataset of multiple sclerosis patients with lesion segmentations based on multi-rater consensus*, Neuroinformatics, **16**:pp. 51–63

[77] O. Commowick, A. Istace, M. Kain, B. Laurent, F. Leray, M. Simon, S. C. Pop, P. Girard, R. Améli, J.-C. Ferré, A. Kerbrat, T. Tourdias, F. Cervenansky, T. Glatard, J. Beaumont, S. Doyle, F. Forbes, J. Knight, A. Khademi, A. Mahbod, C. Wang, R. McKinley, F. Wagner, J. Muschelli, E. Sweeney, E. Roura, X. Lladó, M. M. Santos, W. P. Santos, A. G. Silva-Filho, X. Tomas-Fernandez, H. Urien,

I. Bloch, S. Valverde, M. Cabezas, F. J. Vera-Olmos, N. Malpica, C. Guttmann, S. Vukusic, G. Edan, M. Dojat, M. Styner, S. K. Warfield, F. Cotton, & C. Barillot (2018) *Objective evaluation of multiple sclerosis lesion segmentation using a data management and processing infrastructure*, Scientific Reports, **8(1)**:p. 13650

[78] O. Commowick, F. Cervenansky, F. Cotton, & M. Dojat (Eds.) (2021) *MSSEG-2 challenge proceedings: Multiple sclerosis new lesions segmentation challenge using a data management and processing infrastructure*, Strasbourg, France

[79] A. M. Muslim, S. Mashohor, G. A. Gawwam, R. Mahmud, M. binti Hanafi, O. Alnuaimi, R. Josephine, & A. D. Almutairi (2022) *Brain MRI dataset of multiple sclerosis with consensus manual lesion segmentation and patient meta information*, Data in Brief, **42**:p. 108139

[80] R. Kötter, J. Mazziotta, A. Toga, A. Evans, P. Fox, J. Lancaster, K. Zilles, R. Woods, T. Paus, G. Simpson, B. Pike, C. Holmes, L. Collins, P. Thompson, D. MacDonald, M. Iacoboni, T. Schormann, K. Amunts, N. Palomero-Gallagher, S. Geyer, L. Parsons, K. Narr, N. Kabani, G. L. Goualher, D. Boomsma, T. Cannon, R. Kawashima, & B. Mazoyer (2001) *A probabilistic atlas and reference system for the human brain: International Consortium for Brain Mapping (ICBM)*, Philosophical Transactions of the Royal Society of London. Series B: Biological Sciences, **356(1412)**:pp. 1293–1322

[81] P. J. LaMontagne, T. L. Benzinger, J. C. Morris, S. Keefe, R. Hornbeck, C. Xiong, E. Grant, J. Hassenstab, K. Moulder, A. G. Vlassenko, M. E. Raichle, C. Cruchaga, & D. Marcus (2019) *OASIS-3: Longitudinal neuroimaging, clinical, and cognitive dataset for normal aging and alzheimer disease*, medRxiv

[82] C. R. Jack Jr., M. A. Bernstein, N. C. Fox, P. Thompson, G. Alexander, D. Harvey, B. Borowski, P. J. Britson, J. L. Whitwell, C. Ward, A. M. Dale, J. P. Felmlee, J. L. Gunter, D. L. Hill, R. Killiany, N. Schuff, S. Fox-Bosetti, C. Lin, C. Studholme, C. S. DeCarli, G. Krueger, H. A. Ward, G. J. Metzger, K. T. Scott, R. Mallozzi, D. Blezek, J. Levy, J. P. Debbins, A. S. Fleisher, M. Albert, R. Green, G. Bartzokis, G. Glover, J. Mugler, & M. W. Weiner (2008) *The Alzheimer's disease neuroimaging initiative (ADNI): MRI methods*, Journal of Magnetic Resonance Imaging, **27(4)**:pp. 685–691

[83] I. Mérida, J. Jung, S. Bouvard, D. Le Bars, S. Lancelot, F. Lavenne, C. Bouillot, J. Redouté, A. Hammers, & N. Costes (2021) *CERMEP-IDB-MRXFDG: a database of 37 normal adult human brain [^{18}F]FDG PET, T1 and FLAIR MRI, and CT images available for research*, EJNMMI Research, **11(1)**:p. 91

[84] H. J. Kuijf, J. M. Biesbroek, J. De Bresser, R. Heinen, S. Andermatt, M. Bento, M. Berseth, M. Belyaev, M. J. Cardoso, A. Casamitjana, D. L. Collins, M. Dadar, A. Georgiou, M. Ghafoorian, D. Jin, A. Khademi, J. Knight, H. Li, X. Lladó, M. Luna, Q. Mahmood, R. McKinley, A. Mehrtash, S. Ourselin, B.-Y. Park, H. Park, S. H. Park, S. Pezold, E. Puybareau, L. Rittner, C. H. Sudre, S. Valverde, V. Vilaplana, R. Wiest, Y. Xu, Z. Xu, G. Zeng, J. Zhang, G. Zheng, C. Chen, W. van der Flier, F. Barkhof, M. A. Viergever, & G. J. Biessels (2019) *Standardized assessment of automatic segmentation of white matter hyperintensities and results of the wmh segmentation challenge*, IEEE Transactions on Medical Imaging, **38(11)**:pp. 2556–2568

[85] K. Marek, D. Jennings, S. Lasch, A. Siderowf, C. Tanner, T. Simuni, C. Coffey, K. Kieburtz, E. Flagg, S. Chowdhury, W. Poewe, B. Mollenhauer, P.-E. Klinik, T. Sherer, M. Frasier, C. Meunier, A. Rudolph, C. Casaceli, J. Seibyl, S. Mendick, N. Schuff, Y. Zhang, A. Toga, K. Crawford, A. Ansbach, P. De Blasio, M. Piovella, J. Trojanowski, L. Shaw, A. Singleton, K. Hawkins, J. Eberling, D. Brooks, D. Russell, L. Leary, S. Factor, B. Sommerfeld, P. Hogarth, E. Pighetti, K. Williams, D. Standaert, S. Guthrie, R. Hauser, H. Delgado, J. Jankovic, C. Hunter, M. Stern, B. Tran, J. Leverenz, M. Baca, S. Frank, C.-A. Thomas, I. Richard, C. Deeley, L. Rees, F. Sprenger, E. Lang, H. Shill, S. Obradov, H. Fernandez, A. Winters, D. Berg, K. Gauss, D. Galasko, D. Fontaine, Z. Mari, M. Gerstenhaber, D. Brooks, S. Malloy, P. Barone, K. Longo, T. Comery, B. Ravina, I. Grachev, K. Gallagher, M. Collins, K. L. Widnell, S. Ostrowizki, P. Fontoura, T. Ho, J. Luthman, M. van der Brug, A. D. Reith, & P. Taylor (2011) *The parkinson progression marker initiative (ppmi)*, Progress in Neurobiology, **95(4)**:pp. 629–635, biological Markers for Neurodegenerative Diseases

[86] S. Vukusic, R. Casey, F. Rollot, B. Brochet, J. Pelletier, D.-A. Laplaud, J. D. Sèze, F. Cotton, T. Moreau, B. Stankoff, B. Fontaine, F. Guillemin, M. Debouverie, & M. Clanet (2020) *Observatoire Français de la Sclérose en Plaques (OFSEP): A unique multimodal nationwide MS registry in France*, Multiple Sclerosis Journal, **26(1)**:pp. 118–122, pMID: 30541380

[87] C. Confavreux, D. A. Compston, O. R. Hommes, W. I. McDonald, & A. J. Thompson (1992) *EDMUS, a European database for multiple sclerosis.*, Journal of Neurology, Neurosurgery & Psychiatry, **55(8)**:pp. 671–676

[88] A. Akshay, M. Abedi, N. Shekarchizadeh, F. C. Burkhard, M. Katoch, A. Bigger-Allen, R. M. Adam, K. Monastyrskaya, & A. Hashemi Gheinani (2023) *MLcps: machine learning cumulative performance score for classification problems*, GigaScience, **12**:p. giad108

[89] M. Abedi, N. Shekarchizadeh, P.-L. Bazin, N. Scherf, J. Lier, C.-C. Bergner, W. Köhler, & T. Kirsten (2025), *HeteroMRI: Robust white matter abnormality classification across multi-scanner mri data (version 1)*, [Computer software], Software Heritage, https://archive.softwareheritage.org/browse/snapshot/defd0e13d67d336ca96031dd434ea4779bc1ec2a/directory/

[90] M. Abedi, N. Shekarchizadeh, P.-L. Bazin, N. Scherf, J. Lier, C.-C. Bergner, W. Köhler, & T. Kirsten (2025), *HeteroMRI: Robust white matter abnormality classification across multi-scanner mri data*, [DOME-ML Annotations], DOME-ML Registry, https://registry.dome-ml.org/review/zj8thzbsa7

Supplementary Table S1: Number of MRIs used for training (Tr), validation (V), and test (T) sets from each *dataset* in experimental settings $A00$ to $A18$

**Dataset**		**Set**	**Setting $A\#$**																		
**Label***	**Name**		**00**	**01**	**02**	**03**	**04**	**05**	**06**	**07**	**08**	**09**	**10**	**11**	**12**	**13**	**14**	**15**	**16**	**17**	**18**
+	ISBI	Tr	8	7	6	5	4	3	2	1	0	0	0	0	0	0	0	0	0	0	0
		V	1	1	1	1	1	1	1	1	0	0	0	0	0	0	0	0	0	0	0
		T	2	2	2	2	2	2	2	2	2	2	2	2	2	2	2	2	2	2	2
	UMCL	Tr	8	7	6	5	4	3	2	1	0	0	0	0	0	0	0	0	0	0	0
		V	1	1	1	1	1	1	1	1	0	0	0	0	0	0	0	0	0	0	0
		T	2	2	2	2	2	2	2	2	2	2	2	2	2	2	2	2	2	2	2
	MSSEG	Tr	8	7	6	5	4	3	2	1	0	0	0	0	0	0	0	0	0	0	0
		V	1	1	1	1	1	1	1	1	0	0	0	0	0	0	0	0	0	0	0
		T	2	2	2	2	2	2	2	2	2	2	2	2	2	2	2	2	2	2	2
	MSSEG-2	Tr	8	7	6	5	4	3	2	1	0	0	0	0	0	0	0	0	0	0	0
		V	1	1	1	1	1	1	1	1	0	0	0	0	0	0	0	0	0	0	0
		T	2	2	2	2	2	2	2	2	2	2	2	2	2	2	2	2	2	2	2
	BTH	Tr	6	5	4	3	2	1	0	0	0	0	0	0	0	0	0	0	0	0	0
		V	1	1	1	1	1	1	0	0	0	0	0	0	0	0	0	0	0	0	0
		T	2	2	2	2	2	2	2	2	2	2	2	2	2	2	2	2	2	2	2
	OASIS-3	Tr	10	9	8	7	6	5	4	3	2	1	0	0	0	0	0	0	0	0	0
		V	2	2	2	1	1	1	1	1	1	1	0	0	0	0	0	0	0	0	0
		T	2	2	2	2	2	2	2	2	2	2	2	2	2	2	2	2	2	2	2
	ADNI 3	Tr	39	35	31	28	26	23	21	19	17	14	12	9	7	6	5	4	3	2	1
		V	6	6	6	6	6	6	5	5	5	5	4	4	3	3	3	2	2	1	1
		T	10	10	10	10	10	10	10	10	10	10	10	10	10	10	10	10	10	10	10
−	ICBM	Tr	3	2	1	0	0	0	0	0	0	0	0	0	0	0	0	0	0	0	0
		V	1	1	1	0	0	0	0	0	0	0	0	0	0	0	0	0	0	0	0
		T	1	1	1	1	1	1	1	1	1	1	1	1	1	1	1	1	1	1	1
	OASIS-3	Tr	65	58	51	45	39	32	26	21	15	12	10	8	7	6	5	4	3	2	1
		V	9	9	9	9	9	9	8	8	5	5	3	3	3	3	3	2	2	1	1
		T	16	16	16	16	16	16	16	16	16	16	16	16	16	16	16	16	16	16	16
	CERMEP	Tr	19	17	15	13	11	9	7	5	4	3	2	1	0	0	0	0	0	0	0
		V	3	3	3	3	3	3	2	2	1	1	1	1	0	0	0	0	0	0	0
		T	5	5	5	5	5	5	5	5	5	5	5	5	5	5	5	5	5	5	5
+	Total	Tr	87	77	67	58	50	41	33	26	19	15	12	9	7	6	5	4	3	2	1
		V	13	13	13	12	12	12	10	10	6	6	4	4	3	3	3	2	2	1	1
		T	22	22	22	22	22	22	22	22	22	22	22	22	22	22	22	22	22	22	22
−	Total	Tr	87	77	67	58	50	41	33	26	19	15	12	9	7	6	5	4	3	2	1
		V	13	13	13	12	12	12	10	10	6	6	4	4	3	3	3	2	2	1	1
		T	22	22	22	22	22	22	22	22	22	22	22	22	22	22	22	22	22	22	22
**Total**			244	224	204	184	168	150	130	116	94	86	76	70	64	62	60	56	54	50	48

* With (+) and without (−) WM abnormality

Supplementary Table S2: Number of MRIs used for training (Tr), validation (V), and test (T) sets from each MRI acquisition *protocol* in experimental settings $B00$ to $B04$

Protocol		Set	Setting				
**Label***	**Name**		$B00$	$B01$	$B02$	$B03$	$B04$
+	Sie_Tri_30_Prot1	Tr	5	4	3	2	1
		V	1	1	1	1	1
		T	1	1	1	1	1
	Phi_Ing_30_NA	Tr	5	4	3	2	1
		V	1	1	1	1	1
		T	1	1	1	1	1
	Phi_NA_30_Prot1	Tr	5	4	3	2	1
		V	1	1	1	1	1
		T	1	1	1	1	1
	Sie_Aer_15_Prot1	Tr	5	4	3	2	1
		V	1	1	1	1	1
		T	1	1	1	1	1
	GeE_Dis_30_Prot3	Tr	5	4	3	2	1
		V	1	1	1	1	1
		T	1	1	1	1	1
−	Sie_Bio_30_Prot1	Tr	5	4	3	2	1
		V	1	1	1	1	1
		T	1	1	1	1	1
	Sie_Son_15_Prot1	Tr	5	4	3	2	1
		V	1	1	1	1	1
		T	1	1	1	1	1
	Sie_MaV_30_Prot2	Tr	5	4	3	2	1
		V	1	1	1	1	1
		T	1	1	1	1	1
	Sie_MaV_30_Prot1	Tr	5	4	3	2	1
		V	1	1	1	1	1
		T	1	1	1	1	1
	Sie_TrT_30_Prot2	Tr	5	4	3	2	1
		V	1	1	1	1	1
		T	1	1	1	1	1
+	Total	Tr	25	20	15	10	5
		V	5	5	5	5	5
		T	5	5	5	5	5
−	Total	Tr	25	20	15	10	5
		V	5	5	5	5	5
		T	5	5	5	5	5
**Total**			70	60	50	40	30

* With (+) and without (−) WM abnormality

Supplementary Table S3: Number of MRIs used for training (Tr), validation (V), and test (T) sets from each MRI acquisition *protocol* in experimental settings $C00$ to $C06$. The two protocols in boldface are used only as test data. These two protocols are changed in 10 different cases.

Protocol		Set	Setting						
**Label***	**Name**		$C00$	$C01$	$C02$	$C03$	$C04$	$C05$	$C06$
+	Phi_Ing_30_NA	Tr	7	6	5	4	3	2	1
		V	1	1	1	1	1	1	1
		T	0	0	0	0	0	0	0
	GeE_Dis_30_Prot3	Tr	7	6	5	4	3	2	1
		V	1	1	1	1	1	1	1
		T	0	0	0	0	0	0	0
	Sie_Pri_30_Prot1	Tr	6	5	4	3	2	1	0
		V	2	2	2	1	1	1	0
		T	0	0	0	0	0	0	0
	Sie_Ver_30_Prot1	Tr	6	5	4	3	2	1	0
		V	2	1	1	1	1	1	0
		T	0	0	0	0	0	0	0
	**Phi_Ing_30_Prot2**	Tr	0	0	0	0	0	0	0
		V	0	0	0	0	0	0	0
		T	5	5	5	5	5	5	5
−	Sie_MaV_30_Prot1	Tr	6	5	4	3	2	1	0
		V	1	1	1	1	1	1	0
		T	0	0	0	0	0	0	0
	Sie_Son_15_Prot1	Tr	20	17	14	11	8	5	2
		V	5	4	4	3	3	3	2
		T	0	0	0	0	0	0	0
	**Sie_TrT_30_Prot1**	Tr	0	0	0	0	0	0	0
		V	0	0	0	0	0	0	0
		T	5	5	5	5	5	5	5
+	Total	Tr	26	22	18	14	10	6	2
		V	6	5	5	4	4	4	2
		T	5	5	5	5	5	5	5
−	Total	Tr	26	22	18	14	10	6	2
		V	6	5	5	4	4	4	2
		T	5	5	5	5	5	5	5
**Total**			74	64	56	46	38	30	18

* With (+) and without (−) WM abnormality

Supplementary Table S4: Number of MRIs used for training (Tr), validation (V), and test (T) sets from each MRI acquisition *protocol* in experimental settings $D00$ to $D03$

**Protocol**		**Set**	**Setting**			
**Label***	**Name**		$D00$	$D01$	$D02$	$D03$
+	Sie_Tri_30_Prot1	Tr	21	12	9	8
		V	3	2	2	1
		T	6	3	2	2
	Phi_Ing_30_NA	Tr	15	12	9	8
		V	2	2	1	1
		T	4	3	3	2
	Phi_NA_30_Prot1	Tr	0	12	9	7
		V	0	1	1	1
		T	0	4	3	2
	Sie_Aer_15_Prot1	Tr	0	0	9	7
		V	0	0	1	1
		T	0	0	2	2
	GeE_Dis_30_Prot3	Tr	0	0	0	6
		V	0	0	0	1
		T	0	0	0	2
−	Sie_Bio_30_Prot1	Tr	21	124	11	9
		V	3	2	2	1
		T	6	3	2	3
	Sie_Son_15_Prot1	Tr	15	12	10	8
		V	2	2	1	1
		T	4	3	4	3
	Sie_MaV_30_Prot2	Tr	0	12	10	9
		V	0	1	1	1
		T	0	4	3	2
	Sie_MaV_30_Prot1	Tr	0	0	5	5
		V	0	0	1	1
		T	0	0	1	1
	Sie_TrT_30_Prot2	Tr	0	0	0	5
		V	0	0	0	1
		T	0	0	0	1
+	Total	Tr	36	36	36	36
		V	5	5	5	5
		T	10	10	10	10
−	Total	Tr	36	36	36	36
		V	5	5	5	5
		T	10	10	10	10
**Total**			102	102	102	102

[*] With (+) and without (−) WM abnormality

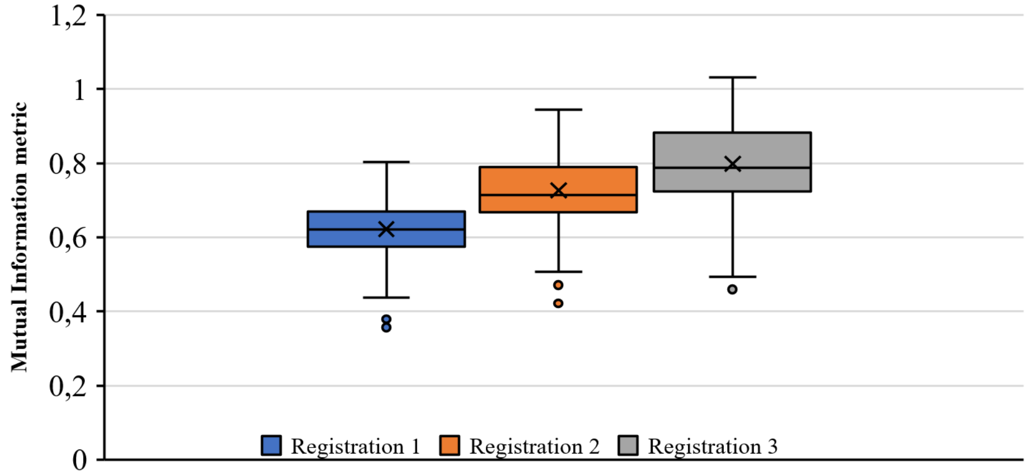

Supplementary Figure S1: Mutual information metric between each MRI and the MNI template after each registration. The $\times$ marker indicates the mean value, and the whiskers represent $1.5 \times IQR$.

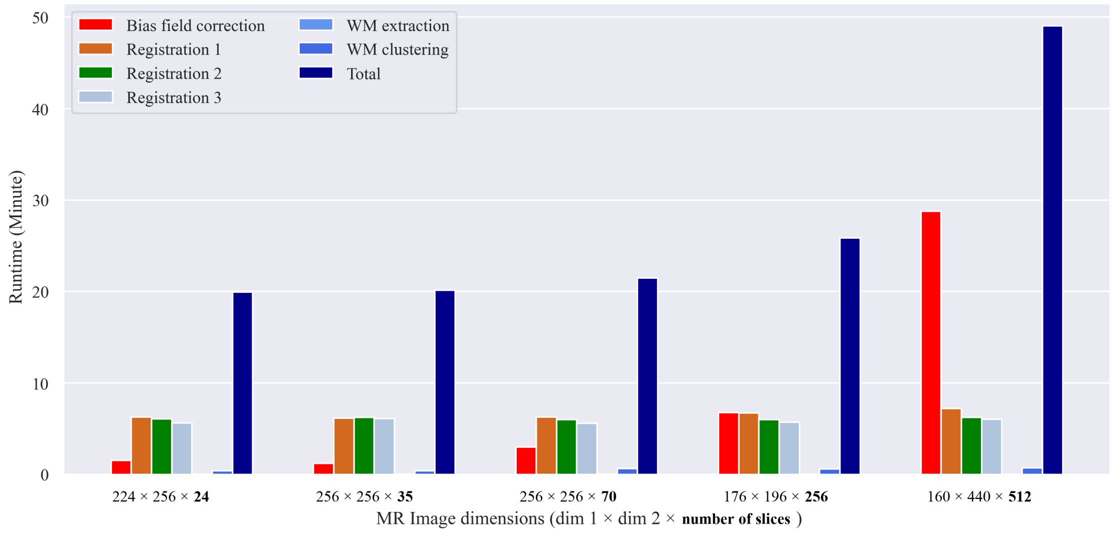

Supplementary Figure S2: Average required time for preprocessing and intensity clustering of five sample MRI dimensions.

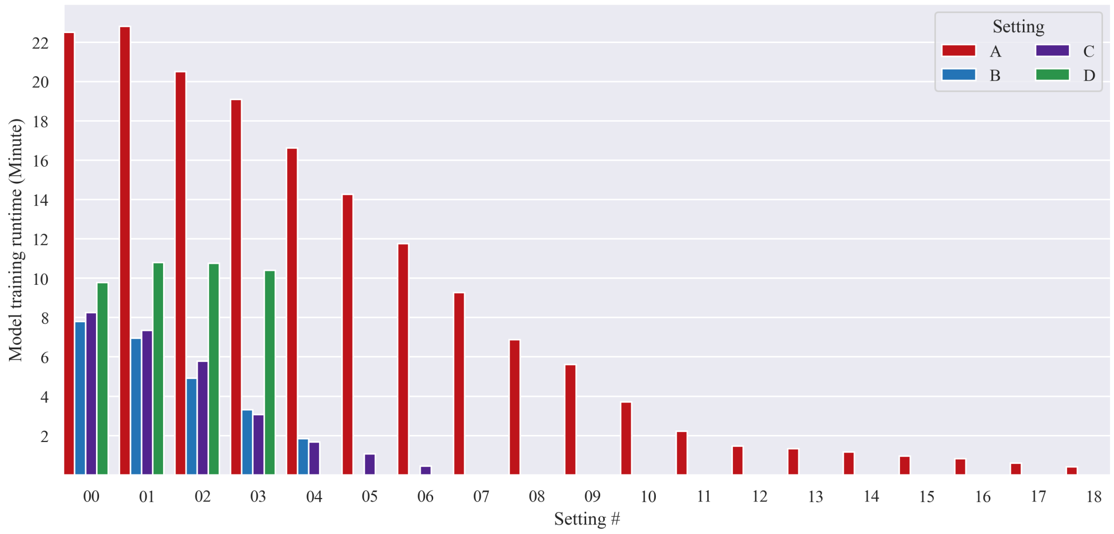

Supplementary Figure S3: Average required time for training the CNN model of each experimental setting.

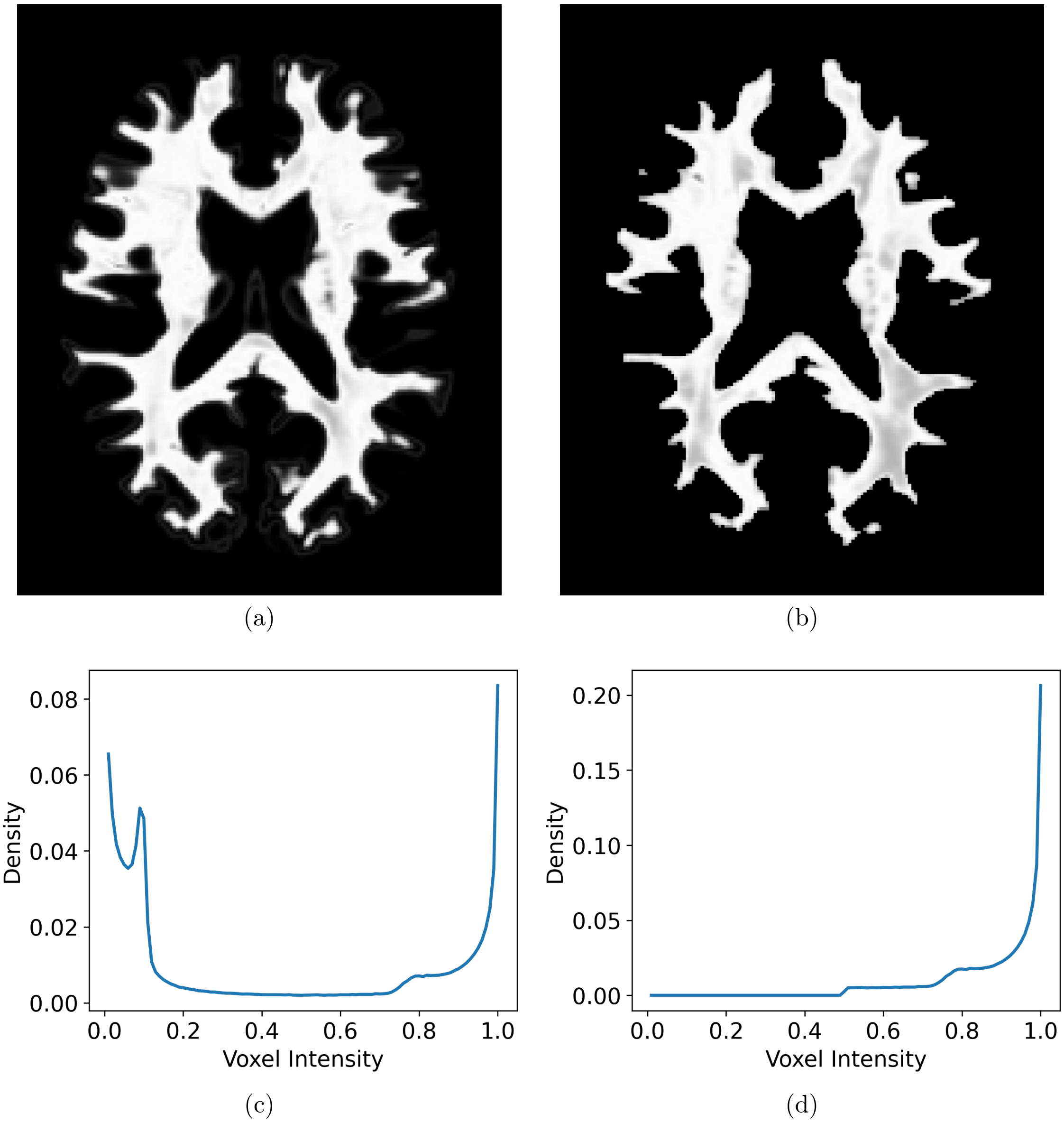

Supplementary Figure S4: The WM intensity cluster of a sample MRI (a) before and (b) after applying a threshold value of 0.5. The normalized histograms of their 99% upper percentile are shown in (c) and (d), respectively.

rev3_Changes marked

Click here to access/download
Supplementary Material
HeteroMRI_rev3_changes.pdf
