# Supplementary material for: HeteroMRI: Robust white matter abnormality classification across multi-scanner MRI data
Source: Gigascience. 2025 Aug 21;14:giaf092. doi: 10.1093/gigascience/giaf092 (PMC12371411; doi:10.1093/gigascience/giaf092)
Supplement: giaf092_Authors_Response_To_Reviewer_Comments_Original_Submission [file giaf092_authors_response_to_reviewer_comments_original_submission.pdf]

Author's Response To Reviewer Comments

Dear Dr. Nicole Nogoy,

We are pleased to have the opportunity to revise our manuscript now entitled “HeteroMRI: Robust white matter abnormality classification across multi-scanner MRI data”. In the revised manuscript, we have carefully considered reviewers’ comments and suggestions. The reviewers’ comments were very helpful overall, and we are appreciative of such constructive feedback on our original submission. We reply to each comment in point-by-point fashion. Please find the responses to the concerns raised by both reviewers below. In addition to the revised manuscript, we also provide a version of the manuscript with all the changes marked with respect to the previous version.

At the end, we would like to thank you and the reviewers for their time and thoughtful comments and efforts towards improving our manuscript.

Sincerely,
Navid Shekarchizadeh
On behalf of the authors

##
Reviewer 1
##
Reviewer #1: The manuscript by Abedi et al have proposed a new deep learning method to predict if a given FLAIR image has white matter abnormalities. The CNN-based method looks interesting and seemingly has quite high performance (up to 96%). However, I have a few concerns: there is no comparison analysis presented, the study design prevents any independent assessment, there is no permutation testing, and very limited clinical utility if the method merely gives a binary decision.

Dear Dr. Pravesh Parekh,
We would like to highly thank you for your detailed assessment of our work and your constructive comments. Thanks to your comments and suggestions, the paper has highly improved through the changes that we made. Please find below a point-to-point answer to your comments. Apart from the changes made based on the comments from the reviewers, we also added the following paragraph (and the relevant Supp. Fig.) as we have missed to include it in the previous version of the paper:

“A thresholding is applied on the selected WM intensity cluster of each MRI. All the intensity values below 0.5 are ignored in order to remove uncertain, low-confidence assignments and retain only the core voxels that are strongly associated with the cluster. The value 0.5 is chosen experimentally in the design phase of HeteroMRI by using datasets different from those used in this study for a task of classifying two WM diseases. The value 0.5 resulted in the highest improvement in the classification accuracy compared to other tested thresholds. The histogram for most MRIs follows the same overall pattern: approximately 50∓5% of values are below 0.2, and around 33∓5% are above 0.8. Supplementary Fig. S4 shows the WM cluster of a sample MRI before and after thresholding along with their normalized histograms (for the 99% upper percentile). Finally, the thresholded clusters from the MRIs (one intensity cluster per MRI) are used as training data for the DL model, as described in the following section.”

Detailed notes below:
Title
1. The title of the paper mentions "classification of multi-scanner and multi-protocol brain magnetic resonance images with deep learning" - however, from this title, it appears that the paper is about differentiating between

multiple scanners and protocols. Put another way, the title doesn't specify the classification task at all. It eithers ends up giving an incorrect impression or fails to answer the question "classification of what"? Suggest updating the title to reflect the content better.

Response: Thank you for your insightful comment regarding the title. The title is changed to “HeteroMRI: Robust white matter abnormality classification across multi-scanner MRI data”. Hopefully, it addresses the points that you mentioned.

Abstract
2. "This study introduces HeteroMRI, a deep-learning method for analyzing MRI datasets incorporating various MRI protocols and scanners" - this seems a bit misleading as HeteroMRI is neither a harmonization method nor is it a general purpose analyses method; this should be rephrased to clarify that HeteroMRI only gives binary predictions of WM abnormalities only using FLAIR images.

Response: The sentence is modified as “This study introduces \textit{HeteroMRI}, a deep-learning method for classifying brain MRIs based on white matter abnormalities. Most importantly, HeteroMRI mitigates the effects of data heterogeneity on classification performance. Herein, the presented method is employed to develop a binary classifier that identifies brain MRIs with white matter abnormalities.”

Introduction
3. Trivial point: page 2, line 52, for consistency, better to introduce the abbreviation FLAIR in the bracket, rather than the other way around.

Response: Thank you for the suggestion. It is changed accordingly.

4. Page 3, line 79, the authors mention that "Before analysing brain MRIs, certain preprocessing steps are commonly performed to optimize the image data for further analysis and interpretation"; at the end of the paragraph, the authors mention defacing which is incongruous to the rest of the paragraph - protecting the privacy of individuals using defacing methods is not a step performed for optimizing the images for any analyses or interpretation - it is a separate issue. I suggest removing that line as it doesn't connect with the rest of the text. It is also not something that the authors themselves re-visit at any point in the manuscript (or something they address).

Response: The sentence about defacing is deleted.

5. Page 3, lines 79-83, the authors mention a range of methods that are commonly employed in (pre-)processing of MR data - however, they do not mention image normalization (i.e., transformation from native space to standard space; although one could argue that it is part of the registration step; note that this is different from intensity normalization), image segmentation, and smoothing (although one could argue that it is related to interpolation and denoising).

Response: Thank you for reminding the point. That is right. There might be further MRI preprocessing procedures that we have not included herein. For the sake of avoiding a long introduction, no change is made in the text in this regard.

6. Page 3, line 86, reference 38: perhaps I missed it while looking into the reference, but I did not see a recommendation that images should be acquired "with the same acquisition protocol, with the same magnetic field strength, and preferably with the same MR scanner model" (or "standardized") - in fact, the reference proposes a harmonization method.

Response: Thank you for your question. In fact the recommendation is not directly mentioned in reference 38 however the following statement is mentioned as a limitation of their work: “There are limitations to this study. In addition to needing all three tissue types (GM, WM, CSF) in each slice for the fit, this study was also performed on a single 3T Philips Ingenia scanner with no changes to protocol (altering TR or TE, slice thickness, etc.).”
We decided to remove this reference from our paper to avoid confusion for the reader.

7. Page 3, line 86, references 37 - 39: I fail to see how the generalization that "it is highly recommended that all the images are standardized" follows from these, where the authors mention that standardized implies same acquisition protocol, same magnetic field strength, and preferably the same MR scanner model.

Response: Please see the response to comment 11

8. Generally, it is true that if the training data and the test data are very different (where differences could originate from different scanner make/models, field strengths, acquisition protocols, etc.), a ML/DL model may fail to generalize. However, that does not imply that "standardization" is the way to go. In other words, while creating a standardized dataset may improve performance in a test scenario, it would never work in a clinical setting (which is what the goal of the paper is).

Response: Yes, completely agreed. As you mentioned correctly, it is the goal of the current paper to perform the analysis with the available heterogenous data since creating a standardized dataset in clinical setting is impractical and even by doing it, one should still make sure if the model is generalizable to the new data in the clinic. We added an essence of your comment to the introduction.

9. "Using a standardized dataset ensures that the images have similar image quality, similar intensity range for each tissue, and similar spatial characteristics." - this is merely a theoretical guarantee; it doesn't really work in practice.

Response: Thank you for spotting the inaccuracy with the sentence. It is revised as follows:
“Using a standardized dataset results in consistent image quality, comparable intensity range for each tissue type, and uniform spatial characteristics, although some variability may still exist.”

10. "Moreover, the contrast among the brain tissues would be uniform across the dataset [40, 37]" - this is not necessarily true; also, doesn't seem to be implied from these citations.

Response: The sentence was removed.

11. The overall issue with this paragraph (page 3, lines 85-94) is that the authors are contextualizing everything as generally true while picking very specific nuanced notes from the field of multiple sclerosis.

Response: Following your comments regarding this paragraph, we rephrased the whole paragraph by changing the way the problem is explained for the reader. As different changes have been made in the introduction, please see the introduction of the revision of the paper for the changes.

12. Page 4, line 114, "The methods based on statistics alter the intensity distribution in the image…" is not entirely accurate - methods like ComBat (references 46-48) are generally meant to be used on features derived from images and not directly the image intensity itself.

Response: Thank you for the correction. The related sentences are revised as follows:
“The proposed MRI harmonization methods in the literature are generally divided into statistical approaches or ML/DL techniques. One group of statistical methods adjusts the intensity distribution, by normalizing the intensity~\cite{SHAH2011267, Nyul1999, SHINOHARA20149, WROBEL2020117242} or by harmonizing values measured from the images (such as cortical thickness) by adjusting the so-called batch effect across different scanners~\cite{FORTIN2016198, FORTIN2017149, FORTIN2018104, Marzi2024, Pomponio2020, RADUA2020116956}”

13. "In this paper, to circumvent the complex MRI harmonization, we present HeteroMRI, an approach for analyzing multi-protocol and multi-scanner MRI data." - well, HeteroMRI has 1,795,905 parameters and therefore is not simple in anyway. Additionally, this doesn't circumvent harmonization in anyway - perhaps I am missing what the authors really wanted to say here.

Response: Thank you for your detecting the ambiguity in this sentence. What we meant here is that HeteroMRI does not try to harmonize the images itself by modifying them for example through a trained AI model.

However, it is true that it reduces the scanner effect through the intensity clustering. We modified the sentence as follows:
“Herein, we present HeteroMRI, an approach for the classification of brain MRIs based on WM abnormalities while mitigating the heterogeneity effects of the images acquired from multiple scanners and acquisition protocols. In this paper, HeteroMRI is utilized to detect brains with WM abnormalities in FLAIR images through binary classification. In future work, the method is intended to be applied to the task of classifying two WM diseases based on their distinct WM abnormality patterns.”

14. Overall, I found the introduction section to be very hard to follow. The introduction starts with a brief remark about WM abnormalities and then goes on to talk extensively about other topics (MR preprocessing, use of ML/DL methods, and an extensive discussion about harmonization). However, it is only towards the end of the introduction that the authors return to WM abnormalities and even then, the goals are a bit unclear - is HeteroMRI a way to harmonize the data or is it a way to detect WM abnormalities which is robust to scanner effects? Throughout the introduction, the authors heavily cite literature from MS and other very specific neurological applications but continue to phrase the introduction very generically. In fact, even towards the end, when the authors state "we present HeteroMRI, an approach for analyzing multi-protocol and multi-scanner MRI data" - even at this point it is unclear what this analysis method is supposed to do - it is only a few sentences
later, "Herein, the presented approach is evaluated for detecting the brains with WM abnormalities through binary classification." that it becomes clear that the method is supposed to identify WM abnormalities. This ambiguity is also applicable to the title where the title states the goal being classification but doesn't specify classification of what. I strongly encourage the authors to edit the introduction to have a stronger focus.

Response: Thank you for your comprehensive comment regarding the introduction. We modified the introduction accordingly in order to improve the flow of the text and remove the ambiguities by adding details on the goal of the paper. Please see the introduction for all the changes.

15. Page 5, line 171: "The presented method opens new avenues for analyzing heterogeneous MRI datasets and the large amount of MRI data generated daily in medical centers" - I don't quite see how. HeteroMRI can classify WM abnormalities as yes and no; it is not a general-purpose analyses tool nor is it a harmonization method. Therefore, "the presented method opens new avenues for detecting WM abnormalities in heterogeneous MRI datasets".
Methodology

Response: We appreciate your thoughtful review. It is true that in this paper HeteroMRI is used for detecting the MRIs that have white matter abnormality, however, this paper is meant to serve as publishing the method which is then to be used for other clinical classification tasks in future work, for example for classifying two different white matter-related diseases which have more or less different white matter abnormality patterns in the brain. We have addressed your comment by modifying the sentence as follows:
“The presented method opens new avenues for performing WM abnormality-related analyses on heterogeneous MRI datasets and the large amount of MRI data generated daily in medical centers.”
Also, another sentence is added in this regard to clarify our future work and the potential applications of HeteroMRI. Please see the response to your comment 13 for the added sentence.

16. "The WM abnormality detection approach presented in this paper uses heterogeneous brain MRI data with various acquisition protocols (multi-protocol) as the data for a Convolutional Neural Network (CNN). The model is a binary classifier trained to detect patients with WM abnormalities in their brain MRI." - this is the first place where the authors have very clearly stated what they do and their goal! This clarity, if added back to introduction, will be very useful to the readers.

Response: Thank you for bringing this to our attention. We modify the introduction accordingly to make the goal of the paper more clear.

17. Page 7, line 189, what about the number of voxels in the third dimension?

Response: The information is added to the text as follows:
“All selected images have a minimum of 128, 192, and 22 voxels in their first, second, and third dimensions,

respectively.”

18. Page 7, line 192: "Detailed information on the parameters used in each step is reported in the GitHub repository of HeteroMRI" - on the GitHub page [https://github.com/ul-mds/HeteroMRI/tree/main], I do not see any details of what FlexiMRIprep settings / configuration was used for which dataset.

Response: Thank you very much for spotting the missing information also on the GitHub page. The info is added to the repository as follows:
“The following parameters are used in the antsRegistration tool:
--dimensionality 3, --interpolation Linear, --transform SyN[gradientStep=0.1], --metric MI[metricWeight=1, numberOfBins=32] (Mutual Information), --initial-moving-transform [initializationFeature=1] --convergence 500x400x70x30, --shrink-factors 8x4x2x1, --smoothing-sigmas 3x2x1x0vox, --use-histogram-matching 0, --winsorize-image-intensities [0.005,0.995]
and for the rest of the parameters the default values are used.

In fuzzy_cmeans function, the parameter values of `clusters=3, max_iterations=200`, and the default values for all the other parameters are used.
”

19. Does this imply that different processing strategies / settings were used for each dataset? If yes, why? What was differently done for which datasets? Please include these details in the manuscript.

Response: Thank you for the insightful question. All the MRIs undergo the same preprocessing steps. We modified the sentence in the paper as follows:
“The preprocessing steps described below are applied identically to all MRIs from different datasets.”

20. Page 7, line 202: "This template is created using the data from the ICBM (International Consortium for Brain Mapping) project" - does this mean you created your own template? I think not, but please clarify / edit the sentence.

Response: Following your advice, we have changed the text as follow:
“Among the available brain templates, we choose the ICBM 2009c Nonlinear Asymmetric template~\cite{FONOV2009S102, FONOV2011313} (referred to below as the MNI template), which the developers created using the data from the International Consortium for Brain Mapping (ICBM) project~\cite{MAZZIOTTA199589}. This template was selected due to its high accuracy and the availability of the WM probability map required for our analysis approach.”

21. Why was the registration step performed three times - the authors claim that this leads to "most precise alignment" - how was this established/judged?

Response: Thank you for your point regarding the registration. We modified the text accordingly to mention how the number 3 is determined and I agree that it the word “most” is not the best word to be used here. The modified text is as follows:

“A nonlinear registration is applied three times (with identical parameters) on each image consecutively. This repeated registration aims to achieve a high level of alignment of the MRIs with the template. The selection of three iterations was determined experimentally to maintain high alignment accuracy while avoiding the higher computational cost of additional nonlinear registrations. Multi-pass registration has also been used by others, for example, to address large differences in the initial positions of image pairs~\cite{Yang2013}.”

22. At the end of registration + normalization step, what was the final voxel size and image size?

Response: Thank you for bringing this to our attention. We added the following sentence:
“The registered MRIs all have a size of 193 × 229 × 193 voxels with a voxel size of 1 × 1 × 1 mm.”

23. Since some of the dataset had 2D images (many in OASIS, ISBI, and BTH), were they all normalized to

some fixed voxel size and overall size?

Response: Yes that is right. All the MRIs were registered to the same template therefore they have an identical image size and voxel size at the end. This is due to the dimension of the input layer of the CNN network which requires all the training data to have a fixed size.

24. From Fig. 1, it appears that all input images were 193 229 193 - that seems like a massive up-sampling for images which only had 22 or so slices. How did the authors arrive at this resolution?

Response: Thank you for the question. That is right. While the 2D MRIs were upscaled to match the fixed 193 229 193 size, many other 3D MRIs were downscaled to this size. This image size is the size of the chosen MNI template. In fact, we did not make any changes to the template, and by registering all the MRIs to the template, they will have the size of the template. Please also see the response to comment 23 in this regard.

25. The WM extraction step is not clear at all - once the FLAIR images are aligned with the MNI template, the voxels still represent some (bias corrected and) interpolated version of the arbitrary MR signal from the raw data. The MNI WM probability map, on the other hand, has values between 0 and 1 showing the probability of that voxel being white matter. A simple multiplication of these two doesn't make sense, given that there will be arbitrary differences between subjects simply because of the scanner intensity scaling differences. The actual voxel intensity per se doesn't have a lot of meaning to it.

Response: Thank you for your question in this regard. What you explained about the mathematics done on the MRI is completely correct. We simply multiply the original WM probability map by the registered MRI. The goal is solely to keep any voxel that “might” be white matter and ignore the rest of the voxels. Of course, the MRIs coming from different scanners still have different orders of voxel values but this is not of importance in this step since the normalization of these numbers is done in the next preprocessing step which is the white matter intensity clustering. We explain further on this step in the response to the comment 27.

Of course, this is true that the brain of each person has a different shape and even a 3-time registration does not make all the brains completely similar. Moreover, multiplying the WM map to it tries to keep the probable WM voxels while ignoring the rest of the voxels. For sure, this approach has some extent of error in extracting the WM which can be considered as a drawback. However, this sacrification of accuracy is with the goal of making use of heterogeneous MRI data, especially in cases of data scarcity such as rare diseases where providing a standardized dataset is impractical.

26. Was the WM probability map binarized?

Response: No, we did not make any changes to the WM probability map.

27. Since there doesn't seem to be any intensity normalization / correction / rescaling, how does the method account for extremely different intensities between different MRI vendors? For example, for data that comes from a Philips scanner, the intensity values would be very high (provided Philips precise scaling is turned on when converting images from DICOM to NIfTI). This would imply intensity values of tens of thousands while the ones from Siemens would be in a few hundreds (typically). Bias correction would not (completely) account for this (since it is not performed across images but within images).

Response: In fact, the intensity normalization is performed in the intensity clustering section. In this step, the extracted WM is divided into three clusters. The resultant 3 intensity clusters have normalized values between 0 and 1. The clusters are membership files (like probability maps) therefore the sum of values of a voxel in the 3 clusters is equal to 1.
In other words, the intensity clustering step not only calculates the 3 clusters but also normalizes the values. Although this normalization is not performed across images, it serves as a last resort in cases of data scarcity where enough data for training an AI model for harmonization of MRI across all the images is not available.

28. Page 7, line 215: "Calculating and obtaining a …", it is just one operation being performed.

Response: Thank you for your attention. The first verb is removed.

29. Visually, it appears that cluster 3 is merely an eroded version of WM segmentation - did the authors try comparing cluster 3 with an eroded version of WM segmentation from standard neuroimaging tools?

Response: Very interesting question. Following your comment, we checked if cluster 3 is merely similar to the eroded version of the WM segmentation. We performed the following steps on one FLAIR MRI: 1-Skullstripping, 2-FAST segmentation, 3-Eroding the segmented WM
And compared it with cluster 3. It came out that they are only similar to a limited extent but are generally different.

30. "We tested various cluster numbers on different MRIs…" - were these the same dataset that were eventually used for training and test?

Response: Thank you for your question. We tested it on a different dataset (a clinical dataset of a different disease) than the ones used for training and testing since this was done in the initial method design stage in which we were not using the final data of the current paper yet. However, it is noteworthy that coming up with the number of clusters does not really depend on the dataset used since after the intensity clustering of the MRIs of the current paper (with 3 clusters) we noticed merely similar-looking clusters in nearly all data. This shows the independency of the selection of the number of clusters from the dataset.

In the light of your question, we clarified the text in the paper as follows:
"The decision to use three clusters was based on our empirical observations from a dataset different from those used in this study. Through testing various cluster numbers on different MRIs, we found that three clusters yielded consistently comparable patterns in nearly all MRIs. In other words, the shapes of the clusters in one MRI were generally consistent with those in another MRI. This was also later observed in the data of the current study. Therefore, the choice of three clusters is robust and does not depend on a specific dataset."

31. "For this study, we manually identified the intended cluster for each MRI by choosing the cluster that looks visually similar to Cluster 3 in Fig. 1." - that doesn't sound like a robust strategy and requires manual intervention. This needs to be addressed.

Response: Thank you for pointing out this point. Please see the response to the next comment.

32. I don't think a DL model to identify the right cluster is necessary - at least a visual examination of the three clusters in Fig. 1 indicates that some combination of standard image processing tools and the right evaluation metrics (like Dice coefficient) might solve the problem. Since the authors have already done the manual identification, it should be easy to evaluate any solution for robustly identifying the correct cluster image.

Response: We thank you very much for this brilliant suggestion. Following your suggestion, we used Dice coefficient and tested it on all the 344 MRIs of this study and Dice score could find the correct WM cluster with 100% accuracy. As the reference image for calculating Dice, we used the average of the intended intensity cluster of four MRIs from our clinal dataset which are not among the data of this study. We updated the code and readme on GitHub and the text in paper accordingly. In fact, we change the manual cluster selection to an automatic one using the Dice score. The related paragraph in the paper is changed as following:

"Upon examining the three WM intensity clusters in MRIs with WM abnormalities, we noted that one of the clusters (Cluster 3 in Fig. 1) within each MRI consistently exhibited significant lesion-related features. Therefore, from each MRI, we should take the cluster that looks visually similar to Cluster 3 in Fig.~\ref{fig:steps} but it is not always the cluster number 3. For this purpose, we use the Dice similarity coefficient\cite{Dice} to compare the three clusters of each MRI with a fixed reference cluster to detect the most similar one. The reference image (available in the GitHub repository of HeteroMRI) is generated by averaging the intended intensity cluster of four MRIs from a clinical dataset. This method detected the right cluster for all the MRIs of this study correctly (i.e. with 100\% accuracy) as checked manually."

33. For the entire DL design - what was the rationale for implementing this specific configuration? In other

words, how did the authors arrive at this precise configuration? Some aspects seem standard while others represent an arbitrary choice - was any of this optimized? If yes, using which dataset?

Response: Good question! We began with a CNN architecture recommended on the Keras website(https://keras.io/examples/vision/3D_image_classification/). The code is an example of 3D image classification of CT scans. We used one of our clinical MRI datasets (Leukodystrophy) and we trained the CNN to classify the MRIs (Leukodystrophy versus its differential diagnoses) with the HeteroMRI approach (this will be another publication based on HeterMRI). As the accuracy was not high enough, we tried adding more layers to the network, which helped increase the accuracy. The added layers are the following:

x = layers.Conv3D(filters=128, kernel_size=3, activation="relu")(x)
x = layers.MaxPool3D(pool_size=2)(x)
x = layers.BatchNormalization()(x)

Considering the GPU memory limitation, which prevents testing the effect of adding more layers, we used this CNN network (with the three new layers added) for the current paper. We observed an even higher accuracy as the classification task of the current paper is easier than differentiating two white-matter diseases. No further optimization of the network was performed due to the costly computations.

34. Table 1: would be additionally helpful (to the readers) to include a hyperlink to the datasets or the page where the dataset can be requested.

Response: Thank you for the suggestion. It is a nice idea however we have noticed that sometimes the webpage address of the datasets changes over time so we refer only to the publication which the reader can use to find out the data access procedure.

35. The MSSEG paper mentions 53 images - why was one image dropped in the current study?

Response: Thank you for noticing this point. Actually, we made an error during the data preparation and creation of the MRI list and one image of the MSSEG dataset is missing from the list. The missing image is "Patient_10_Center_08_test" which is the last image of the dataset (by sorting the images alphabetically) which has probably been deleted from our MRI list inadvertently. Therefore it is not included in the experiments. We noticed this issue after all the costly computations were finished so we could not help it.

36. ICBM dataset - only five out of 20 did not have WM abnormalities; can't the remaining 15 be used as positive cases (just like in OASIS-3)? Why were they removed?

Response: Good question! Yes, that is right, we could have added them as cases. However, there was already more than enough cases available with a high diversity of scanners, protocols, and WM lesion patterns. Furthermore, if we wanted to increase the number of cases in an experiment (e.g. A00), we did not have more controls to add to keep the labels balanced. The number of controls used in A00 setting was the most number of controls that we were able to provide considering our resources and time limits.

37. For OASIS-3, from a pool of 600 images, only 90 were identified without WM abnormalities; then, how are there only 14 cases with WM abnormalities? Either some detail is missing here or one of the numbers is incorrect - from the document on GitHub, it looks like 90 and 14 are correct - that means there was a massive pool of images with WM abnormalities, but they were not used in the study? Why not?

Response: Thank you for the question. We added the 14 images from OASIS-3 during the design of Setting A since in A00 only 14 more MRIs were necessary to complete this setting by considering the balanced number of cases and controls. However, we simply did not put more images of this dataset in the MRI list in favor of data diversity as the scanner and protocol diversity was already so high among the data. In summary, we had no specific intention or goal for not including more images (cases) of this dataset.

38. ADNI: First, please specify if you examined across ADNI1, ADNI2, ADNI-GO, and ADNI-3? Second, what was the total number of available images? Third, if only 58 images with WM abnormalities were identified,

shouldn't there be a good number of control images from ADNI? Why were they not used?

Response: Thank you for noticing the missing information and the question regarding ADNI dataset. The MRIs used in this study are taken randomly from the downloaded pool of FLAIR images from ADNI 3. We changed ADNI to ADNI 3 throughout the paper text. Regarding the second question: By filtering ADNI 3 data by the %FLAIR% in the " Image Description" field, we would get 2401 images from 1147 unique patients. We got the 58 cases from the 235 first patients of the patients' list (by sorting Subject ID ascendingly) by trying to include different WM lesion patterns. Regarding the third question: As the neurologists checked the ADNI images, very limited number of brains were free of WM abnormalities while in OASIS dataset, there was around 15 MRIs per 100 patients. Therefore, it was decided to consider only OASIS (among the two) for finding controls. This decision was made considering the time-consuming task of checking all the slices of hundreds of MRIs. This difference between ADNI and OASIS might be due to the age of the subjects, which may be higher in ADNI, causing most of the brains to have WM abnormalities. However, we did not check this fact specifically.

39. CERMAP: The remaining 10 subjects who were identified as having minor lesions - why were they not used as cases?

Response: Please see the answer to comment 36. The situation is similar.

40. Coming back to BTH: the dataset has 60 2D FLAIR images - however, only 9 were used in the present study. The authors mention a problem with the 2D images; however, many images in the OASIS dataset had similar number of slices: specifically, 8 images with 24 slices and 86 with 35 slices. What was the strategy used for processing the OASIS images and couldn't the same be used for BTH?

Response: Thank you for noticing this point. This is true that the 2D MRIs of BTH and OASIS datasets have merely a similar number of slices. The difference is about the slice thickness. In OASIS the slices have realistic thicknesses so when you open the raw file, it looks like a normal brain but with low resolution in the coronal and sagittal planes. However, in BTH, all slices have a 1 mm thickness. Therefore, in the raw file, the brain has a much lower height than a normal brain in the coronal and sagittal planes. So it is not possible to directly register the BTH images to the MNI template, unlike the OASIS images. Even increasing the thickness of the slices of BTH images based on the provided metadata file does not result in normal-looking brains for all patients. That is why we chose only images of 9 patients that looked "quite" normal after increasing the slice thickness. Despite the problems with BTH dataset, we preferred to keep it in the study in favor of having higher heterogeneity (protocol diversity) in the study data.

41. Overall comments for the data section: the rationale for what was included and not, is not specified / is unclear. Please provide more details. There seems to be a large number of cases/controls that could have been included but were seemingly not.

Response: We hope the current version of the paper and the answers to your comments addresses your concerns.

42. What about the age and sex distribution of the samples from each of the site? These are important variables to consider when evaluating whether there are systematic differences in sites or between cases and controls within a site.

Response: Thank you for your question. We used only public datasets and not all datasets share the demographic data of the subjects. Therefore we cannot consider such variables in the study, unfortunately.

43. There is no mention of quality checks performed on the images prior to their inclusion in the analysis - this is critical, especially given that the authors later remark about registration / segmentation issues - please provide documentation on what were the QA/QC procedures employed. If these were not done, there needs to be a very good reason why not.

Response: In this regard, our criteria was to include FLAIR images that do not have severe noise or artifacts. Since all the MRIs of this study are from public datasets, apparently the MRIs have been checked once by the dataset providers, therefore we expect no MRI with severe noise or artifact to be present among the data.

Furthermore, we created a list (“MRIs_list.csv” on GitHub) of all MRIs with their 3D dimension and voxel size to make sure that there are no images with dimensions less than the minimum values reported in the paper. Regarding registration, It is not completely clear to us which factors result in the registration problem. Otherwise, we would have excluded the MRIs with such factors in the data quality control phase. The only factor we found that sometimes led to incorrect registration was the low number of slices in the 2D MRI. However, one might argue that we could have removed the MRIs with the wrong registration from the study. We refrained from removing such cases since we prefer HeteroMRI to be working without human interference. The goal is to develop a tool (especially for the future applications of HeteroMRI, i.e. other white-matter-related classification tasks) to be used by clinicians therefore we avoid the necessity of technical interference.

44. For each of the settings: as a first sentence, can you please state the goal of the experiment/setting? It would be helpful for the readers to get oriented in the first sentence itself of the goal of the experiment.

Response: Thank you for your nice suggestion. Following your comment, we added the first sentence for the settings that missed such a sentence, as following:
Setting A:
“In setting A, the goal is to evaluate HeteroMRI on a combination of MRIs from different datasets beginning from a relatively large number of data and then decreasing the data gradually.”
Setting B:
“In setting B, the goal is to choose the MRIs with the most diversity of protocols while having an equal number of MRIs from each protocol therefore the model sees the same number of MRIs per protocol.”
Setting C:
“In setting C, the goal is to assess the generalizability of HeteroMRI to unseen MRI protocols.”

45. I found supplementary table S1 hard to follow; it was easier to use the Excel sheet from the GitHub repository of the authors - perhaps the first part of the table (where the dataset breakdown is not present) could be additionally included in the supplementary materials?

Response: We agree with you that understanding the Excel sheet is easier. However, fitting more parts of the table in the supplementary table is challenging as the space is limited in an A4 page. We would expect the interested reader to see the GitHub repository.

46. Page 11, line 229: "The number of MRIs with and without WM abnormality is balanced in the training data of all the settings." - wasn't this the case for validation and test sets as well (and not just for training)?

Response: That is right. Thank you for spotting the missing point. The mentioned sentence is combined with the next sentence and it is modified as follows:
“The number of MRIs with and without WM abnormality is balanced in the training, validation, and test data of all the settings.”

47. What was the rationale for enforcing equal number of cases and controls in training, validation, and test sets? Was it merely convenience to ensure that training was not biased by class imbalance?

Response: Thank you for the question. Yes, we always balanced the cases and controls to avoid any bias due to class imbalance, so that the model learned equally from both classes. It was also important because we wanted to test the ability of the model to handle the heterogeneity of the MRI data coming from different scanners and protocols without the additional complexities introduced by class imbalance.

48. The authors had 222 positive cases and 122 negative cases; in setting A, 100 positive cases were removed - on what basis? And which ones?

Response: Good question! In each setting and in each shuffle, the code (https://github.com/ul-mds/HeteroMRI/blob/main/select_id_settings_class.py) selects the necessary number of MRIs from the MRI list randomly. The selection is either based on name of dataset or name of the protocol depending on the experimental setting design. For example, in setting A00, 244 MRIs are selected randomly (in this case all 122 controls are taken and 122 random MRIs from the 222 pool of cases are taken). In setting A01 (and further),

as the data needs to be decreased, the necessary number of MRIs is removed from the data used for A00 randomly, only with the condition that the highest number of protocols is maintained among the data used in the setting.
We add the following sentences to the paper to clarify the point that you mentioned:
“The number of images necessary for each setting is selected randomly from the MRIs available.”
and
“ The downsizing is performed by removing random MRIs while keeping the maximum possible number of protocols among the data.”

49. Even if the authors insisted on balanced training and validation split, couldn't these 100 be used for external holdout testing?

Response: Thank you for your suggestion. The 100 MRIs are all from cases and considering them as a test set does not allow calculating and evaluating all performance metrics. Due to limited number of available controls, we did not have extra control MRIs to combine them with the 100 cases to consider as a holdout test set.

50. Setting B: there are total 32 protocols; it is unclear how only 10 protocols were sampled. What was the goal of setting B?

Response: Good questions regarding the setting B.
Selection of 10 protocols:
In the setting B, the goal was to include the data with the following conditions: 1) to include as many protocols as possible, 2) to include an equal number of images (as many as possible) from each protocol, and 3) to include an equal number of protocols for both cases and controls. Based on these conditions, we selected a total of 10 protocols (5 for cases and 5 for controls), each with at least 7 MRI scans available in our MRI list. This resulted in having 70 MRIs (10*7) in setting B00. It is noteworthy that, in each data shuffle, the 7 MRIs per protocol are selected randomly among the available data of that protcol. If we chose more than 10 protocols, e.g. 12, then we could take only 5 MRIs per protocol (since not all of the 12 protocols had more than 5 images in the MRI list). In this case, B00 would have 60 MRIs (12*5), however, we prefer to see the effect of data size on the performance of the model beginning from a higher number and then reduce to smaller numbers in B01, B02, etc.
The goal of setting B:
We designed setting B for having the highest diversity of protocols and at the same time trying to remove any other factor that might affect the model’s learning. In setting A, the number of MRIs per protocol is not the same and this may have an effect as the model sees different number of images from each protocol. In setting B, it is the other way around.

51. Overall, the experimental design section was hard to follow; perhaps responding to and adding clarification based on my previous comments might improve the readability of this section.

Response: We hope the current version of the paper and the answers to your comments addresses your concerns and has improved the readability of this section.

52. Generally speaking, the equal number of cases and controls situation is merely an idealised scenario and does not reflect practical clinical setting.

Response: We appreciate your close evaluation of our proposed method. It is true that in clinical settings the cases and controls are not equal. However, the positive point about the proposed approach is that it can ignore the scanner effect to a high degree therefore it is possible to collect data (e.g. from different scanners of the hospital) to provide the necessary training data for the model. Moreover, as we are evaluating the approach in this paper, we are staying with the balanced scenario to avoid other sources of complexity in the model and we may test the model for unbalanced scenarios in future work.

Results and Discussion
53. For Figures 3 and 4, please include a slice from the registered MRI that corresponds to the slice presented in the WM intensity cluster - that will make it easier to compare the image used for learning/prediction vs. the

image from which the cluster was derived.

Response: Thank you for your insightful suggestion. Following your comment, we added the middle slice of the registered MRI in Figs. 3 and 4. Also, the WM cluster is now showing the middle slice so it matches the registered MRI. The WM cluster shown is now thresholded as it is the input to the CNN model. As explained in the beginning of this letter, we previously missed to include the thresholding step in the paper.

54. It is a bit hard to follow how the authors are concluding that MRIs 4, 5, 6, and 10 have incorrect registration - the cluster images still capture white matter. Perhaps including additional images on what went wrong would help underscore this point?

Response: Your helpful suggestion in the previous comment also helps solving this issue! Since we are displaying the middle slice of the registered MRI, it is possible to compare these slices to the middle slice of the MNI template shown in Fig. 1. If one sees different sections of the brain compared to the template, the registration has been incorrect. For example, in MRIs 4 and 6 in Fig. 3, we are seeing the eyes in the middle slice of the registered MRI while in the MNI template we do not see the eyes in this slice number. This is an apparent sign of erroneous registration. The following text is added to the paper in this regard:
" These registration errors are identified by comparing the slices of the registered MRIs with the corresponding slice from the MNI template slice shown in Fig. 1. In both cases, the middle slice of the MRI is displayed. In an accurate registration, the brain regions in the registered slice align approximately with those in the template."

55. It seems a bit remarkable that even in A18 or B04, the average accuracy is ~67% and 65% - since the datasets have been (artificially) balanced between cases and control images, in extreme paucity of data (such as 1 + 1 in A18), one wouldn't expect any meaningful learning to happen - and therefore, the accuracy should fall to chance level: 50%.

Response: Thank you for your interesting question. We would also expect the chance level to happen for such settings. We think a much higher number of shuffles would result in an average accuracy of ~50%, however, due to the high computational cost we refrained from doing that. Moreover, the more important for us was to find the mere border (data size) after which the model's performance falls drastically (e.g. below 70%). Regarding the reason for the 67% average accuracy in A18, it is noteworthy that the test set contained 44 MRIs (a rather large number) and we see in Fig. 2(a) how widely the values fluctuated among the 200 runs (20 shuffles*10 runs each). This shows that the average 67% is not a completely reliable number and more shuffles are necessary which is not the focus of the current study. On the other hand, the accumulative score (MLcps%) for A18 is only 34% which shows the low learning of this setting. The same discussion holds true for B04.

56. In fact, in B04, the accuracy looks to be ~65% which is ~222/(222+122) i.e., the original cases and control proportion, considering majority class. Can the authors comment on this?

Response: Thank you for noticing this interesting point. However, the number of data used in setting B is different from 222+122, as we are taking MRIs only from certain protocols. Therefore, we believe this is a coincidence rather than a result of the original class proportion.

57. Page 18, lines 487 - 491, the authors have inadvertently ended up overfitting to the test set - fitting a model and examining the performance on the test, followed by a decision to change the model, refit, and report performance on the same test set. This is problematic; at the bare minimum, the authors should include (in the supplement), the results from the original situation of using 2D images and acknowledge the fact that these results are obtained after examining the prediction performance on the test set and then re-doing the modelling (overfitted to test).

Response: We understand and appreciate your concern regarding possible refitting of the model on the same test data. The point is that the test set of the two versions of the setting C were not the same data. By making the decision to exclude 2D MRIs, we completely redesigned setting C. Not only the test data is not the same, but also the number of data in the training, validation, and test were changed. Moreover, each setting is run multiple shuffles, each time splitting the data to train and test. Therefore, we believe that it is not a refitting since we have not learnt any lessons from the first test. We redesigned the setting based on our suspicion that

2D MRIs might be source of error. Following your comment we modified the related paragraph in the paper as following, in order to prevent the concern of the reader for refitting:

“Initially, we used both 3D and 2D MRIs for setting C. However, the model showed a lack of robustness when its generalizability to different protocols was evaluated (i.e. by varying the protocols considered as test data). As we suspected the 2D MRIs (with 70 or fewer slices) as a source of the model's poor performance, we redesigned the setting C to include only 3D MRIs. As a result, the performance significantly improved, as reported in the results for setting C. It is important to emphasize that the test data were not fixed for the two versions of setting C, as the model was tested with multiple data shuffles for each setting, with the data being split again in each shuffle. This ensures that this was not a case of refitting on the same test data. Additionally, to verify the adverse effect of 2D MRIs, we designed a separate setting with only 2D MRIs. In this case, the model showed very poor robustness. ”

58. Why does the average performance of C06 look a little better than C05? As mentioned in my previous comment, since there is barely any meaningful learning happening at such a reduced sample size, we expect accuracy to hit 50% (balanced cases and controls) - and we see that in C05; but C06 performance, surprisingly, goes up a little bit - doesn't seem to be noisy variance around 50%.

Response: The same discussion mentioned in response to comment 55 for settings A and B holds true for setting C. In case of having higher number shuffles, the average accuracy of C06 should be less than C05. Nevertheless, even in the current results, the MLcps% score shows a slightly lower value for C06 compared to C05.

59. A general critique of all the box plots - can you make sure they have the same ticks and tick spacing to ensure easy comparability across accuracy, sensitivity, and specificity? Also, please mention that the triangle marker indicates the mean - doesn't seem to be specified anywhere.

Response: Thank you for your comment regarding the box plots. Although having similar ticks for the three plots helps the comparability, it can make the plots hard to read in some cases such as Fig. 6. Putting the ticks range from 0 to 100 for all plots causes the box plots in some plots to be compressed tightly and therefore hard to read and compare. Therefore, we would keep the current tick format. Thank you for reminding the triangle marker meaning. We added the following sentence to all the plots’ titles:
“The triangle marker indicates the mean value.”

Overall feedback
60. Altogether, it is important to assess how these models perform compared to chance - the authors have not performed any permutation testing. Since the image preprocessing and cluster detection is ready, it should be relatively easy to perform permutation testing and include these results.

Response: Thank you for the suggestion. Yes it is easy to run the permutation test except the very high computational cost of the CNN model. However, following your suggestion, we performed the permutation test for the setting A00. We permuted the data labels 7000 times, for the training, validation, and test data and run each model two times (i.e. 7000 data shuffles and permutation, and in total 14000 runs). We averaged over the 2 runs ending up with 7000 accuracy values. None of the 7000 accuracy values was equal or larger than the original performance of setting A00 (even by considering the lowest accuracy among the 10 shuffles of setting A00). This results in a p-value of 1/7000, indicating a highly significant distinction between the two classes in the data. We add the information about the permutation test in the Resutls section of the paper as following:
“We conducted a permutation test to assess the statistical significance of the model’s performance. By randomly shuffling the class labels 7,000 times and retraining the model (for setting $A00$) on each shuffled dataset, a distribution of accuracy values under chance conditions was obtained. None of the 7,000 permuted accuracy values was equal to or greater than the model's original accuracy for setting $A00$. This results in a p-value of 0.0001, showing a statistically significant and meaningful distinction between the two classes, confirming that the model captures real patterns in the data. Supplementary Fig. S3 shows the histogram of the permutations compared to the model's original accuracy.”

Also, Supp. Fig. S3 is added to show the histogram of the permutations.

61. Additionally, the authors have not performed any comparison with any other method - for example, a trivial comparison would be to take any of the commonly used methods for detecting WM abnormality using FLAIR (for example, FSL's BIANCA), pass the images through it, and convert the WM hyperintensity segmentation into yes/no, which can then be compared with HeteroMRI performance.

Response: We thank you for your point regarding the absence of comparison with other methods. The reason for this is that we do not know of any conventional or ML/DL method that classifies brain MRI as with/without WM abnormality. On the other hand, converting WM hyperintensity segmentation into Yes/No brings up the question about the threshold. How much would be the maximum volume of segmented lesions to ignore and count as a “No”? If we take any voxel segmented as hyperintensity into account, probably most or nearly all MRIs would be a “Yes”.
Moreover, the lesion segmentation methods (including FSL’s BIANCA) mostly require lesion annotation for the training data so they are not candidates to be compared with HeteroMRI which only requires a binary label for each MRI.
The focus and goal of HeteroMRI is mainly on mitigating the heterogeneity effects coming from scanners so we do not see it directly comparable with lesion segmentation methods.

62. Similarly, there are several other ML/DL based methods for detecting WM hyperintensities (including using convolutional neural network). The authors have not mentioned any prior work done in this regard nor have they attempted to compare their method with any of the recent work in the area; this is important to assess.

Response: Please see the response to comment 61.

63. One of the biggest drawbacks of the study design is that there is no holdout set - given the propensity for deep learning methods to overfit, it would have been more convincing to see an external evaluation (outside of the train-validation-test framework) - preferably even a site that was never used. It may perhaps still be possible, given large number of additional FLAIR datasets available.

Response: Thank you for your question and suggestions regarding a holdout set. The preferred case of having a test set from a site that was never used in training is in fact implemented in setting C, where the model is trained on 8 MRI protocols and tested on 2 other protocols that were not used in training. In setting C, for all the data shuffles the test set stays the same. Here each protocol can be considered as a site since in our definition, two different protocols have either different scanners or different acquisition parameters.

Moreover, in other settings, we believe that for every data shuffle, we have a holdout test set since the test data is completely held out from the training process. Moreover, the setting #s (i.e. A00, A01, …, with decreasing sizes of training and validation) can be compared with each other while the same holdout test set is used for all of them in every shuffle.

However, if a holdout set means a fixed test set for all the data shuffles (same as in setting C), this situation can be interpreted/addressed in three ways:
1-If we take the holdout set from a combination of the current datasets/sites, the first question that comes to mind would be: What would be the rationale for choosing the holdout data? If we choose the holdout data randomly only one time, the measured performance will not be reliable as the test result is sensitive to the selected holdout data.

2-If, at the current stage of this work, we want to take the holdout data from a new site (from other public datasets) that was never used before in this paper, one might ask the reason for choosing this site from all the available public data?

3-If, at the current stage of this work, we want to take one of the sites, which are currently used in the paper, as the holdout data (and completely exclude it from training), a few concerns/issues will raise, as mentioned below:
3a-We do not have a site among our data that has both cases and controls (except OASIS-3) so we should take one site per cases and one site per controls as a holdout set. In this case, one might ask why you chose

these two specific sites as holdout?
3b-There are numerous possibilities for choosing different sites as holdout. Assume we have data from n sites in the cases and data from m sites in the controls. Testing the combination of choosing all these sites (m*n) has a tremendously high computation cost. Considering the current number of shuffles (20), the number of runs (10), and the number of setting #s (e.g. A00, A01, …), it results in a very high number of runs (probably from 10^4 order). In practice, this is impractical for our CNN model.
3c-The number of data from each site is not equal. Therefore leaving out one site from case and one from control will make the data labels unbalanced which is not preferred by us.

Considering the above-mentioned discussion, we believe the current experimental setup is the best possible design that we could come up with (by considering the conditions of the current data and CNN architecture). Therefore we do not make changes to the experimental setup. Moreover, it is not currently possible to test the already-trained models on a holdout set because we have only extra case MRIs from different datasets but not controls. In fact, we have used all the control MRIs (confirmed by our neurologists) in the experiments.

64. Related to previous comment, except for OASIS-3, all datasets used in the study have either cases or controls but never both - since we do not really see the identified lesions/WM changes (except for cluster3 image), could some of the performance be attributable to merely learning some image characteristics of the site (which correlates to case status)?

Response: Thank you for your thoughtful comment. We believe that the likelihood of this phenomenon occurring is extremely low. This is due to the following reasons:

1-Setting C shows that the approach is highly independent of the MRI protocol or site.
2-The model sees only the WM intensity cluster (not the whole MRI), in which we believe the scanner effects are largely removed.
3-It is noteworthy that a “dataset” does not always mean a “site”. Five out of nine datasets are not from the same site or scanner, and they include heterogeneous MRIs. Therefore, even if we included data (e.g., from ADNI) in both classes, it does not necessarily mean that we have MRIs from a similar site in both classes.
4-We included as many MRI protocols as we could in the dataset to ensure it is as heterogeneous as possible and to minimize the likelihood of learning from site-specific features.

65. Another thing to consider is that HeteroMRI only gives a binary classification output - it does not highlight or mark the suspected regions of WM abnormalities/changes; this makes it hard to evaluate the method from other approaches that attempt to label the changes. It also reduces the applicability of the work in a clinical setting where a radiologist might be interested in having an AI tool to help locate WM changes (rather than decision making, which anyway requires a physician/radiologist to look at the image before signing off a report).

Response: Thank you for your comment on the applicability of HeteroMRI. The main point to consider (also mentioned in response to comment 15) is that this paper is intended to present the HeteroMRI method which is capable of classifying heterogeneous MRIs based on their white matter differences. In future work, HeteroMRI will be used for classifying MRIs based on white matter diseases. In this case, the model will be much more applicable in clinical settings and would be an interesting tool for neurologists.
Following the above-mentioned plan, segmenting the white matter lesions is not the goal of HeterMRI and therefore it does not depend on lesion manual annotation mask for the training phase. In the case of unsupervised lesion segmentation (i.e. without having the lesion annotation), a very high number of MRIs might be necessary for training the model.
We think that the current classification task of HeteroMRI can be useful in some occasions that a non-neurologist person (such as a medical student or an MRI operator) needs to get an idea if the MRI has any white matter abnormalities.
As mentioned correctly, evaluating this model by comparing it with other approaches is not easy since almost all other models are concerning the segmentation of lesions.

66. On a related note, is it possible to apply a reverse (or inverse) deformation field that warps the WM cluster 3 images back to native space? That would improve the utility of the present work and allow examination of what exactly went wrong for false positives and false negatives. This is a solved problem - most non-linear registration tools should be able to generate forward and reverse warps.

Response: Thank you for your interesting suggestion. Technically it should be possible to apply the reverse warp (which is generated by AntsRegistration). We would consider this suggestion in the future work for improving HeteroMRI in the next versions.

##
Reviewer 2
##
Reviewer #2: The authors aim to develop a deep-learning method, HeteroMRI, that minimizes the effects of MRI scanner and protocol heterogeneity in the analysis of white matter abnormalities. Their method achieves high accuracy in binary classification of brain MRIs, even with limited data, thus improving the potential for machine learning integration into clinical MRI practices regardless of scanning conditions.

Dear Dr. Chiara Marzi,
We would like to thank you for your comments and assessment of our work. We hope the current version of the paper addresses your concerns and questions. We believe the paper has highly improved thanks to the comments and suggestions of the reviewers. Please find below a point-to-point answer to your comments. Apart from the changes made based on the comments from the reviewers, we also added the following paragraph (and the relevant Supp. Fig.) as we have missed to include it in the previous version of the paper:

“A thresholding is applied on the selected WM intensity cluster of each MRI. All the intensity values below 0.5 are ignored in order to help the CNN model focus on the parts of the WM cluster indicative of WM abnormalities. The value 0.5 is chosen experimentally in the design phase of HeteroMRI by using datasets different from those used in this study for a task of classifying two WM diseases. The value 0.5 resulted in the highest improvement in the classification accuracy compared to other tested thresholds. The histogram for most MRIs follows the same overall pattern: approximately 50∓5% of values are below 0.2, and around 33∓5% are above 0.8. Supplementary Fig. S4 shows the WM cluster of a sample MRI before and after thresholding along with their normalized histograms (for the 99% upper percentile). Finally, the thresholded clusters from the MRIs (one intensity cluster per MRI) are used as training data for the DL model, as described in the following section.”

Major comments in order of appearance within the text
1. It is unclear how the variability due to different MR scanners and acquisition protocols is identified and attenuated. Is it done through WM clustering, or is it directly addressed within the Deep Learning model designed for the classification task?

Response: Thank you for your question regarding the methodology. In fact, it is the WM clustering that reduces the scanner effects. The intensity clustering step not only calculates the 3 clusters but also normalizes the values. Although this normalization is not performed across images for reducing the scanner effects, it serves as a last resort in cases of data scarcity where enough data for training an AI model for harmonization of MRI across all the images is not available. Therefore, the scanner effects and data heterogeneity is reduced before training the Deep Learning model and the model is solely trained to find patterns and classify the MRIs based on WM changes.

2. In Figure 1, do all the images, except MNI, refer to the same subject? I would also suggest being as consistent as possible in selecting the position along the axial direction, as it does not appear to be consistent in the "MRI preprocessing."

Response: Thank you for noticing this point in the figure. We updated all the screenshots in Fig. 1 so that a more consistent section of the brain is shown.

3. The authors state, "A non-linear registration is applied three times (with identical parameters)." It is unclear why the registration procedure is repeated three times in series. I recommend that the authors provide references regarding the utility of performing multiple registrations and include all parameters used for co-registration (e.g., degrees of freedom, type of interpolation, etc.).

Response: Thank you for your comment and reminding about missing parameters of registration. We modified the text accordingly to mention why the registration is repeated three times. Moreover, a related reference is added about multi-pass registration. The text is modified as follows:
“A nonlinear registration is applied three times (with identical parameters) on each image consecutively. This repeated registration aims to achieve a high level of alignment of the MRIs with the template. The selection of three iterations was determined experimentally to maintain high alignment accuracy while avoiding the higher computational cost of additional nonlinear registrations. Multi-pass registration has also been used by others, for example, to address large differences in the initial positions of image pairs~\cite{Yang2013}.”

The info about parameters of registration is added to the GitHub repository as follows:
“The following parameters are used in the antsRegistration tool:
--dimensionality 3, --interpolation Linear, --transform SyN[gradientStep=0.1], --metric MI[metricWeight=1, numberOfBins=32] (Mutual Information), --initial-moving-transform [initializationFeature=1] --convergence 500x400x70x30, --shrink-factors 8x4x2x1, --smoothing-sigmas 3x2x1x0vox, --use-histogram-matching 0, --winsorize-image-intensities [0.005,0.995]
and for the rest of the parameters the default values are used.

4. In the "WM clustering" paragraph, the authors state that they chose to use three clusters based on empirical observations. What data were these observations derived from? If these empirical tests were performed on the same data used for WM clustering and classification, this constitutes data leakage, potentially leading to overestimated classification performance. Similarly, the authors write: "Finally, all the selected clusters from all the MRIs (one intensity cluster per MRI) serve as the training data for the DL model, as described in the following section." This is another case of significant data leakage, which should be avoided, preferably by design in the processing workflow, by performing WM clustering only on the training data and applying it solely to the test data.

Response:
Regarding the first question:
We updated the text to mention how we chose to use three clusters. The updated paragraph is as following:
“A clustering algorithm is used to estimate three intensity clusters from the WM volumes obtained in the previous step. The algorithm groups the WM volumes into three subgroups that share a relatively similar intensity range. We expect that one of the clusters shows more indications of WM abnormalities (if present in the brain) since the abnormalities have higher intensity values in FLAIR images. This specific cluster will be used as the training data for the classifier model. The cluster is a membership function with float values in the range of [0,1] for each voxel. As a result, the impact of the heterogeneous nature of the multi-protocol MRIs is minimized. The decision to use three clusters was based on our empirical observations from a dataset different from those used in this study. Through testing various cluster numbers on different MRIs, we found that three clusters yielded consistently comparable patterns in nearly all MRIs. In other words, the shapes of the clusters in one MRI were generally consistent with those in another MRI. This was also later observed in the data of the current study. Therefore, the choice of three clusters is robust and does not depend on a specific dataset.”

Regarding the second point:
We would like to clarify that WM clustering here is a preprocessing step, and it is applied to all data, including the test set. The purpose of clustering is to divide the white matter (WM) into 3 clusters and use one cluster as input for the CNN model. This approach helps minimize scanner effects and therefore supports the model in focusing on WM changes. We do not consider this process to involve data leakage since: 1) the number of clusters is fixed for all the data and 2) the selected cluster has a similar visual pattern (and differentiable from the other two clusters) for all the data.

In the updated version of the paper, we have automated the cluster selection process using the Dice

coefficient. This approach selected the same clusters as the ones previously selected manually. This assures that the previous manual selection, based on visual criteria, did not introduce data leakage. In fact, both the previous and current cluster selection methods follow a systematic and reproducible criterion rather than human decisions. We believe this addresses the concerns about potential data leakage. The following text is added to the paper regarding the automatization of selection of WM cluster:
“Upon examining the three WM intensity clusters in MRIs with WM abnormalities, we noted that one of the clusters (Cluster 3 in Fig. 1) within each MRI consistently exhibited significant lesion-related features. Therefore, from each MRI, we should take the cluster that looks visually similar to Cluster 3 in Fig. 1 but it is not always the cluster number 3. For this purpose, we use the Dice similarity coefficient\cite{Dice} to compare the three clusters of each MRI with a fixed reference cluster to detect the most similar one. The reference image (available in the GitHub repository of HeteroMRI) is generated by averaging the intended intensity cluster of four MRIs from a clinical dataset. This method detected the right cluster for all the MRIs of this study correctly (i.e. with 100\% accuracy) as checked manually.”

5. In the description of the various experimental settings, there are cases where the training is done with very few images (4, 10, 8, ...). How is it possible to train a deep architecture with so many parameters using such a small number of examples?

Response: Thank you for your question. It is true that we do not expect a deep model to learn a lot from only a few images therefore we would expect the chance level to happen for such settings (for example the settings A12 to A18). We think a much higher number of shuffles would result in an average accuracy of ~50%, however, due to the high computational cost we refrained from doing that. Moreover, the more important for us was to find the mere border (data size) after which the model’s performance falls drastically (e.g. below 70%).
Regarding the reason for the 67% average accuracy in A18, it is noteworthy that the test set contained 44 MRIs (a rather large number) and we see in Fig. 2(a) how widely the values fluctuated among the 200 runs (20 shuffles*10 runs each). This shows that the average 67% is not a completely reliable number and more shuffles are necessary which is not the focus of the current study. On the other hand, the accumulative score (MLcps%) for A18 is only 34% which shows the low learning of this setting. The same discussion holds true for B04.

6. In the "3.3 Model Execution" section, was the data split stratified to maintain the same proportion of subjects with and without WM abnormalities, as in the entire multicenter cohort? If not, accuracy is not an appropriate metric.

Response: Yes. As mentioned in the paper, in all the experimental settings, the training, validation, and test data are always balanced for the two classes of data.

7. The authors state that they trained and tested the various models 10 times. Was an average performance reported?

Response: Yes, the metric values are average values over all data shuffles and run. For example in setting A, the data is shuffled and splitted 20 times and the model is trained 10 times using each of the 20 data shuffles. The box plots show the resulting 200 values from all the runs (20*10). Moreover, now we added to the figure captions that the triangle marker indicates the mean value of the box plot.

8. Section 4 is titled "Results and Discussion," but the discussion is completely absent. A critical integration of the current work with previous literature is a crucial missing component.

Response:
Thank you for your comment in this regard. A new section is added to the paper to discuss the results and highlighting the limitations and introducing future directions. Please see the section in the revised manuscript.

Regarding the absence of comparison with other methods. The reason for this is that we do not know of any conventional or ML/DL method that classifies brain MRI as with/without WM abnormality. Of course, there are numerous models for WM lesion segmentation but converting WM hyperintensity segmentation into Yes/No

brings up the question about the threshold. How much would be the maximum volume of segmented lesions to ignore and count as a “No”? If we take any voxel segmented as hyperintensity into account, probably most or nearly all MRIs would be a “Yes”.
Moreover, the lesion segmentation methods mostly require lesion annotation for the training data so they are not candidates to be compared with HeteroMRI which only requires a binary label for each MRI.
The focus and goal of HeteroMRI is mainly on mitigating the heterogeneity effects coming from scanners so we do not see it directly comparable with lesion segmentation methods. Moreover, this paper is meant to serve as publishing the method which is then to be used for other clinical classification tasks in future work, for example for classifying two different white matter-related diseases which have more or less different white matter abnormality patterns in the brain.

9. The text between lines 403 and 415 should be included in the methods section rather than int the results section.

Response: Thank you for your suggestion. The mentioned text is moved to a new subsection (3.4 Evaluation metrics) in the Experiments section since the MLcps cumulative score is not the focus of our Methodology or is not innovated by us in this paper.

10. The percentage of 2D MRI scans should be clearly reported in the data description section.

Response: Following your suggestion, we added the following sentence to the Datasets subsection:
“Around 35% of these MRIs are 2D, based on our definition that MRIs with 70 or fewer slices are considered 2D.”

11. After registration, it is mandatory to check the images and exclude those with poor registration output.

Response: We refrained from removing such cases since we prefer HeteroMRI to be working without human interference. The goal is to develop a tool (especially for the future applications of HeteroMRI, i.e. other white-matter-related classification tasks) to be used by clinicians therefore we avoid the necessity of technical interference.
Furthermore, removing cases with poor registration from the dataset could be seen as introducing bias, potentially overfitting the model to a subset of images that may not reflect the real-world variability encountered in clinical settings.

12. In section "4.2 Setting B", the authors state that "Setting B, which uses an equal number of MRIs from each MRI protocol, is designed to make the prediction task more challenging for the model." Why would the task be more complex if the situation is perfectly balanced?

Response: Thank you for your thoughtful question regarding setting B. We designed setting B for having the highest diversity of protocols and at the same time trying to remove any other factor that might affect the model’s learning. In setting A, the number of MRIs per protocol is not the same and this may have an effect as the model sees different number of images from each protocol. In setting B, it is the other way around. The challenge for the model in setting B is to learn the underlying patterns from the available images, without being biased by protocols with a higher number of images.

13. The conclusions are too lengthy and serve as a summary of the work and results rather than true conclusions.

Response: The conclusion is completely revised and made shorter. Please see the conclusion in the revised manuscript.

Minor comments in order of appearance within the text
1. Line 49: "white matter" and "gray matter" do not need to be capitalized.

Response: Changed accordingly.

2. Line 52: the acronym "FLAIR" should be introduced after, not before, its explanation.

Response: Changed accordingly.

3. Lines 85-94: add that the variability due to different scanners and acquisition protocols is often greater than the biological variability (see, e.g., references (Fortin et al., 2018, 2017, 2016; Marzi et al., 2024; Pomponio et al., 2020; Radua et al., 2020)

Response: References added as suggested

4. Line 113: the phrase "are either based on statistics or ??? learning (AI???)" needs clarification.

Response: The sentence is modified as follows:
“The proposed MRI harmonization methods in the literature are generally divided into statistical approaches or ML/DL techniques.”

5. Line 115: add (Marzi et al., 2024; Pomponio et al., 2020; Radua et al., 2020) to the references for "Adjusting for batch effects."

Response: References added as suggested

6. Lines 153-160 should be moved earlier in the text.

Response: Following your suggestion, the paragraph is moved earlier in the introduction

7. Line 177: correct "Secs. 4" to "Sec. 4."

Response: Changed accordingly.

8. Line 181: revise to "as the data input for a CNN."

Response: We changed as “as the input data for a Convolutional Neural Network (CNN)”. We keep the full form of CNN as here is the first time we are using the abbreviation CNN in the paper.

9. In the MSSEG-2 dataset, why did you only consider 10 scanners instead of 12? A few lines earlier, the authors themselves state, "This variability persisted even among the sites that had scanners from the same manufacturer and model."

Response: As mentioned in the paragraph of MSSEG-2, the images of the training set were acquired at “12 different sites” and using “10 different scanners”. In our protocol naming (explained in the GitHub repository of HeterMRI), we do not consider it as a different protocol if only the site is different. If the scanner manufacturer and model, and the acquisition parameters of two MRIs are the same, we consider them as having the same protocol. Following this assumption, we have 10 protocols in MSSEG-2. However, one might be more strict in defining a protocol and consider the site also as a factor.

10. Lines 479 and 489: what are the layers referring to?

Response: Here, by “layers” we mean the slices of an MRI. For higher clarity, we changed the word “layers” to “slices” everywhere in the paper, following your comment.

11. Line 483: the sentence "In a nutshell, by training the model with only 46 3D MRIs, it is able to classify MRIs acquired with protocols unseen during training with 88.1% accuracy." is redundant, as it was already mentioned in the previous line.

Response: Thank you for noticing this point. The sentence is removed.

12. Line 494: what does the "#" symbol signify?

Response: As explained in the first paragraph of Sec. 4, “setting #'' refers to each of the settings in a setting group, for example A00, A01, …, and A18 are the setting #s in setting A.

References

Fortin, J.P., Cullen, N., Sheline, Y.I., Taylor, W.D., Aselcioglu, I., Cook, P.A., Adams, P., Cooper, C., Fava, M., McGrath, P.J., McInnis, M., Phillips, M.L., Trivedi, M.H., Weissman, M.M., Shinohara, R.T., 2018. Harmonization of cortical thickness measurements across scanners and sites. Neuroimage 167, 104-120. https://doi.org/10.1016/j.neuroimage.2017.11.024

Fortin, J.P., Parker, D., Tunç, B., Watanabe, T., Elliott, M.A., Ruparel, K., Roalf, D.R., Satterthwaite, T.D., Gur, R.C., Gur, R.E., Schultz, R.T., Verma, R., Shinohara, R.T., 2017. Harmonization of multi-site diffusion tensor imaging data. Neuroimage 161, 149-170. https://doi.org/10.1016/j.neuroimage.2017.08.047

Fortin, J.-P., Sweeney, E.M., Muschelli, J., Crainiceanu, C.M., Shinohara, R.T., 2016. Removing inter-subject technical variability in magnetic resonance imaging studies. NeuroImage 132, 198-212. https://doi.org/10.1016/j.neuroimage.2016.02.036

Marzi, C., Giannelli, M., Barucci, A., Tessa, C., Mascalchi, M., Diciotti, S., 2024. Efficacy of MRI data harmonization in the age of machine learning: a multicenter study across 36 datasets. Sci Data 11, 115. https://doi.org/10.1038/s41597-023-02421-7

Pomponio, R., Erus, G., Habes, M., Doshi, J., Srinivasan, D., Mamourian, E., Bashyam, V., Nasrallah, I.M., Satterthwaite, T.D., Fan, Y., Launer, L.J., Masters, C.L., Maruff, P., Zhuo, C., Völzke, H., Johnson, S.C., Fripp, J., Koutsouleris, N., Wolf, D.H., Gur, Raquel, Gur, Ruben, Morris, J., Albert, M.S., Grabe, H.J., Resnick, S.M., Bryan, R.N., Wolk, D.A., Shinohara, R.T., Shou, H., Davatzikos, C., 2020. Harmonization of large MRI datasets for the analysis of brain imaging patterns throughout the lifespan. NeuroImage 208, 116450. https://doi.org/10.1016/j.neuroimage.2019.116450

Radua, J., Vieta, E., Shinohara, R., Kochunov, P., Quidé, Y., Green, M.J., Weickert, C.S., Weickert, T., Bruggemann, J., Kircher, T., Nenadić, I., Cairns, M.J., Seal, M., Schall, U., Henskens, F., Fullerton, J.M., Mowry, B., Pantelis, C., Lenroot, R., Cropley, V., Loughland, C., Scott, R., Wolf, D., Satterthwaite, T.D., Tan, Y., Sim, K., Piras, Fabrizio, Spalletta, G., Banaj, N., Pomarol-Clotet, E., Solanes, A., Albajes-Eizagirre, A., Canales-Rodríguez, E.J., Sarro, S., Di Giorgio, A., Bertolino, A., Stäblein, M., Oertel, V., Knöchel, C., Borgwardt, S., du Plessis, S., Yun, J.-Y., Kwon, J.S., Dannlowski, U., Hahn, T., Grotegerd, D., Alloza, C., Arango, C., Janssen, J., Díaz-Caneja, C., Jiang, W., Calhoun, V., Ehrlich, S., Yang, K., Cascella, N.G., Takayanagi, Y., Sawa, A., Tomyshev, A., Lebedeva, I., Kaleda, V., Kirschner, M., Hoschl, C., Tomecek, D., Skoch, A., van Amelsvoort, T., Bakker, G., James, A., Preda, A., Weideman, A., Stein, D.J., Howells, F., Uhlmann, A.,
Temmingh, H., López-Jaramillo, C., Díaz-Zuluaga, A., Fortea, L., Martinez-Heras, E., Solana, E., Llufriu, S., Jahanshad, N., Thompson, P., Turner, J., van Erp, T., Glahn, D., Pearlson, G., Hong, E., Krug, A., Carr, V., Tooney, P., Cooper, G., Rasser, P., Michie, P., Catts, S., Gur, Raquel, Gur, Ruben, Yang, F., Fan, F., Chen, J., Guo, H., Tan, S., Wang, Z., Xiang, H., Piras, Federica, Assogna, F., Salvador, R., McKenna, P., Bonvino, A., King, M., Kaiser, S., Nguyen, D., Pineda-Zapata, J., 2020. Increased power by harmonizing structural MRI site differences with the ComBat batch adjustment method in ENIGMA. NeuroImage 218, 116956. https://doi.org/10.1016/j.neuroimage.2020.116956

Editorial Manager®

Cookie Preference Center

We use cookies which are necessary to make our site work. We may also use additional cookies to analyze, improve and personalize our content and your digital experience. For more information, see our Cookie Policy.

You may choose not to allow some types of cookies. However, blocking some types may impact your experience of our site and the services we are able to offer. See the different category headings below to find out more or change your settings.

Allow all

Manage Consent Preferences

Strictly Necessary Cookies

Always active

These cookies are necessary for the website to function and cannot be switched off in our systems. They are usually only set in response to actions made by you which amount to a request for services, such as setting your privacy preferences, logging in or filling in forms. You can set your browser to block or alert you about these cookies, but some parts of the site will not then work.

Functional Cookies

These cookies enable the website to provide enhanced functionality and personalisation. They may be set by us or by third party providers whose services we have added to our pages. If you do not allow these cookies then some or all of these services may not function properly.

Performance Cookies

These cookies allow us to count visits and traffic sources so we can measure and improve the performance of our site. They help us to know which pages are the most and least popular and see how visitors move around the site.

Targeting Cookies

These cookies may be set through our site by our advertising partners. They may be used by those companies to build a profile of your interests and show you relevant adverts on other sites. If you do not allow these cookies, you will experience less targeted advertising.

Cookie List

Clear

Apply Cancel

Consent Leg.Interest

Confirm my choices

Powered by onetrus
