# Supplementary material for: HeteroMRI: Robust white matter abnormality classification across multi-scanner MRI data
Source: Gigascience. 2025 Aug 21;14:giaf092. doi: 10.1093/gigascience/giaf092 (PMC12371411; doi:10.1093/gigascience/giaf092)
Supplement: giaf092_Authors_Response_To_Reviewer_Comments_Revision_1 [file giaf092_authors_response_to_reviewer_comments_revision_1.pdf]

Author's Response To Reviewer Comments

Dear Dr. Nicole Nogoy,

We are pleased to have the opportunity to revise our manuscript now entitled “HeteroMRI: Robust white matter abnormality classification across multi-scanner MRI data”. In the revised manuscript, we have carefully considered the reviewer’s comments and concerns by addressing them adequately. We reply to each comment in a point-by-point fashion. Please find the responses to the concerns raised by the reviewer below.

At the end, we would like to thank you and the reviewer for their time and thoughtful comments and efforts towards improving our manuscript.

Sincerely,
Navid Shekarchizadeh
On behalf of the authors

##
Reviewer 1
##
Reviewer #1: The authors have revised their previous submission and have adequately responded to several of the comments. Nonetheless, there are still several concerns that remain with the study, including but not limited to lack of comparisons with other methods, lack of integration with the existing literature, incomplete permutation testing framework, and a reluctance to perform any kind of external validation. Detailed comments follow.

Dear Dr. Pravesh Parekh,
Thank you for your 2nd round of detailed comments on our manuscript. We have addressed your comments and concerns in a point-by-point fashion. Please find below the responses to your comments. In addition to the changes mentioned in the following, we have updated all the performance plots and values since they have had slight changes due to increasing the number of data shuffles.

Sincerely,
Navid Shekarchizadeh
On behalf of the authors

Introduction
1. The introduction is still quite long and wordy with needless diversions and forays into areas which generally do not lead to anything. There is plenty of avenue to cut down and make the introduction focused/sharp - for example, most of the first paragraph is superfluous (except for T2 FLAIR), the second and the third paragraphs can easily be slashed altogether, elaboration on the standardized datasets can be compressed into two or so sharp sentences, the harmonization section and all the citations therein do not directly bring much to the table except for the point that it is important, and the elaborate paragraph on the limitations of ML/DL methods is not necessary.

Our response: We thank the reviewer for this recommendation. Indeed, we realize the introduction needs better focus, and removed superfluous text pointed out here. Please see the shortened introduction in the revised paper.

2. Page 4, line 111-112, "Literature concerning harmonization techniques is relatively limited" - not sure where the authors get that impression from. Over the past decade or so, extensive work has been undertaken in the field to not only understand the sources of MR signal variability but also in quantifying the scanner effects as well as developing ways to deal with these. Same holds for the subsequent statement about there being an

"absence of standard criteria for assessing scanner effects and evaluation harmonization process" - there have been a few proposals in the field on quantifying the scanner effect as well as evaluating harmonization process. While this area is nascent, work in the area is not absent.

Our response: Following your comment, we have removed the mentioned sentence.

3. Page 4, line 125, the authors imply that supervised ML harmonization techniques commonly use "manually selected features" - the authors have cited references 51 and 52. In 51, Garcia-Dias et al never performed feature selection but used all 68 IQMs + PCA; in 52, Jog et al compute various features to create synthetic MRI datasets - it is not primarily designed as a "harmonization" method (even though it can be used to achieve intensity standardization which is not equivalent). The point is, the authors are exaggerating the differences between ML/DL approaches which is not well backed up by the literature.

Our response: Thank you for the suggested correction. In the revised version of introduction mentioned in the response to comment 1, this has been removed.

Methods

4. "All selected images have a minimum of 128, 192, and 22 voxels in their first, second, and third dimensions, respectively" - do you mean 22 voxels or 22 slices in the third dimension?

Our response: Thank you for your meticulous point! The information about the number of voxels in the third dimension was added in this sentence following your comment No. 17 in the first review round where you asked the "number of voxels" in the third dimension. Here, voxel and slice have the same meaning for the third dimension, as we are talking about the raw MRIs.

5. The authors have used T2 template for registering FLAIR images - did they run any tests prior to this step to judge whether this was an appropriate decision? For example, ADNI has a large number of T1 weighted images. One possible test could be to see if the T1 images registered to T1 template and then the registration applied to the FLAIR images gives comparable results as FLAIR images registered to T2 template.

Our response: We agree that using T1 contrast should indeed work well when those images are available, and the processing pipeline could easily be adapted to include it. However, we did not have T1 images for all subjects, and thus preferred working directly with the FLAIR data in all cases. Therefore, we have not performed the mentioned test.

6. "This repeated registration aims to achieve a high level of alignment of the MRIs with the template." - the authors have not really answered my question. How was the high level of alignment judged / quantified? This is really critical because the authors are using a T2 template with a different contrast property than the FLAIR images as well as the fact that there are several 2D images in the dataset. How was the accuracy of alignment established for 2D and 3D images?

Our response: Thank you for your question on the registration quality. In this regard, we have performed two evaluations. Firstly, in order to quantify the registration, we have measured the mutual information of the registrations with the MNI template after each registration. The plot is added to the paper as a supplementary figure X. The initial pre-registration images were excluded from the MI plot due to inconsistent voxel resolutions and orientations, which make the MI values incomparable with those after registration. The following text is added to the registration section of the paper:

"

Mutual information (MI) was calculated between each registered MRI and the MNI template after each of three registration steps. As shown in Supplementary Figure X, the MI increased after each nonlinear registration step, with mean values improving from 0.62 (1st registration), to 0.73 (2nd registration) and 0.8 (3rd registration). However, the improvement between the second and third steps was relatively small ($\Delta MI \approx 0.072$), indicating diminishing returns. This suggests that three registration steps are sufficient to achieve consistent and reliable alignment. Performing additional iterations may not justify the computational cost and could even introduce unnecessary anatomical distortions, particularly due to the nonlinear nature of the

transformations.
”

For sure, there will be MRIs with wrong registration that is also affecting the average accuracy of the current results. We have mentioned this as a limitation of the method. However, in the next version of the method, we can add a metric for automatically warning about or removing the wrong registrations, for example by calculating the jacobian of the displacement field of registration and deciding based on a threshold value.

7. So, the images were indeed massively upsampled to 193 229 193 - this is remarkable, especially for the 2D images that had 22 voxels (slices?) to start with. Can the authors include snapshots of a few 2D images with the raw and normalized images put next to each other? I find the decision to upsample so much quite baffling - the authors could have easily chosen a lower resolution to upsample to (for example, 2 or 3 mm isotropic) without modifying the template.

Our response: Given the heterogeneity and sometimes very low quality of the FLAIR images, the high resolution resampling performed during alignment to the template may be unnecessary, and a lower resolution might offer a better compromise. However, with a high resolution we could ensure that most details of the FLAIR data, when present, would be preserved. Even with the current resolution, some 3D FLAIR data have been downsampled as they have had a higher resolution originally. This is because all images have to have the same dimension as the input layer of the CNN model. Actually, we could not follow how could it be possible to “choose a lower resolution to upsample to without modifying the template” since the registered image will have the resolution of the template. At your request, we have provided the snapshots of three 2D MRIs before and after each of the three registrations as a PDF file attached to this letter.

8. For the WM extraction step - I still don't see the rationale of multiplying arbitrary MR intensity values with the probability values. It would be straightforward to take the probability values, apply a minimal threshold to convert it into a binary mask, and then do the multiplication - it still gives you the WM images and you retain the distribution of the MR intensity values (albeit only the white matter) rather than converting them to some sort of probability weighted distribution. If the latter is useful, that needs to be shown; if not, this step is really a strange image processing decision that is not commonplace and warrants some justification. The authors argue that this was done to "keep any voxel that "might" be white matter and ignore the rest of the voxels" - well, that would be easily achieved by a binary mask. In fact, a binary mask is going to result in a more accurate map ignoring of the rest of the voxels because they would be set to zero rather than some arbitrary (relatively small) number.
Our response:
Thank you for your question about the reason for using the WM probability maps. Our goal has been simply to prioritize regions defined as inside the WM for the clustering step, so using the probability values rather than a hard threshold allows to downweight the influence of regions with some partial voluming of other tissues. In fact we stick to the WM probability map that is determined by MNI and avoid changing it to a binary mask since it is hard/expensive to choose/study the threshold value. Of course one could study your suggested method to see the result.

9. In the WM clustering step, the authors state "As a result, the impact of the heterogeneous nature of the multi-protocol MRIs is minimized" - how? The logic of this is not clear to me. If the reasoning is that the images are converted to three separate probability map images with values between 0 and 1 and this somehow leads to "minimization" of the impact of different MRI protocols, then the same could have been achieved with any processing stream that results in probability maps. At best, all this results in are (intensity-wise) comparable images. It does not minimize the impact of different MRI protocols or at least it does it in the same way that any standard processing stream would do (which is to say barely).

Our response: Thank you for pointing out this matter. It is true that other approaches to generate probability maps would also provide such type of effect, which is to reduce the variability coming from different protocols. Following your comment, we change the mentioned sentence in the paper as the following:
“As a result, the impact of the heterogeneous nature of the multi-protocol MRIs is reduced, in a way that the proposed method is robust to the many MRI protocols we used in this work.”

10. Table 1: while it is true that hyperlinks could change over time, that doesn't prevent the authors from including the hyperlink they used to access / request access to the dataset. It also makes it easier to reproduce the analyses from the paper since we know exactly where to go to get the datasets that you used, rather than attempting to trace it through guess-estimates.

Our response: Following your suggestion to include the links for helping the reader to find the datasets more easily, we include the links in the GitHub repository of HeteroMRI.

11. MSSEG - you can include a footnote to indicate that one subject was inadvertently omitted from the analysis.
Our response: Thank you for your suggestion. The footnote is added as following:
"The MSSEG dataset has orignially 53 FLAIR images but one of them was omitted inadvertently by us from our study."

12. For the datasets where a subsample is used in the paper, please include a note stating that this was done (i.e., in the description mention the total images in the dataset, the number of images that were selected, and how the selection was done (e.g., randomly selected).
Our response: The information is now added for the missing ones.

13. The authors have stated that not all the datasets available had demographics information - they can still report the age and sex distribution for the datasets that provided demographics information. My point was not to use this for any kind of prediction of WM but to report the demographics so that we understand the dataset (and any implicit biases therein) better. This is especially true in light of the selection of subset of images.

Our response: We thank you for your suggestion to add the demographic information. We have added the demographic information to Table 1 for the datasets which the demographic information was available.

14. Regarding QA/QC, the authors have responded with "since all the MRIs of this study are from public datasets, apparently the MRIs have been checked once by the dataset providers" - this is not true. There is no obligation on the part of the dataset providers to check the data prior to their release; this responsibility lies with the analysts. For example, ADNI provides QC ratings for different scans and clearly states that "users can employ scan-level QC information as filters in preparing image collections.". For OASIS-3, there is post processing QC on Freesurfer output but that does not imply that the raw dataset (which the present study uses) is QCed. Further the authors state "…therefore we expect no MRI with severe noise or artifact to be present among the data." - this is, again, an incorrect presumption. Several of these datasets are known to have problematic images. I repeat my comment from the first round: "There is no mention of quality checks performed on the
images prior to their inclusion in the analysis - this is critical … - please provide documentation on what were the QA/QC procedures employed. If these were not done, there needs to be a very good reason why not." Filtering scans based on the number of slices is not really quality check.

Our response: Thank you for your comment regarding the QA/QC procedure. In addition, to the previously mentioned assumption about public datasets and to make sure about the quality of images, all the MRIs were indeed checked visually by the authors to exclude images with large artifacts. A number of MRIs were removed based on this criteria from the study. An example is the BTH dataset which had low quality data in it. Moreover, the MRIs without WM abnormality were selected by neurology specialists to assure the absence of even small WM abnormalities in the MRIs considered for the negative label. In this regard, the following sentence was added to the Datasets section:
"All the MRIs were visually checked by the authors to exclude images with large artefacts."

15. For OASIS-3, how were scans selected between repeated measurements?

Our response: Thank you for your question. For each subject, the latest session in date (largest number of days from the first visit shown by d###) was selected. In case of multiple FLAIR images in the same session,

always the image with the suffix “run-1” is selected.

Results
16. Generally speaking, please indicate the standard deviation along with all the reported mean performance measures in the text.

Our response: We appreciate your suggestion. The standard deviation was added to all the mean values reported in the paper.

17. Setting A00: there is at least one instance where the specificity drops close to 50% - what could have happened there? Since the datasets are balanced, it can't be because of class imbalance. Similarly, there are at least two scenarios where the accuracy is <= 80%. Could you look into this and find out what could have caused this variability? Seems to indicate that there are a few specific random splits where the model does not perform quite well - is this related to the 3D/2D scenario?

Our response: Thank you for the fine point you mentioned. We checked the cases you mentioned. Now by increasing the shuffles to 50, such cases increased from 2 to 4. We did not notice a clear reason for such phenomena. As you mentioned correctly, the random data split (train/validation/test data) has caused the model not to be trained well or have a difficult test.

18. In all A* scenarios, the specificity is always on the higher side. Why? Is it that during training, the model is overfitting to the positive class (perhaps incorrectly learning most data points as positive class) and failing to generalize?

Our response: Thank you for your question in this regard. In fact, the values of specificity and sensitivity are very close to each other from A00 up to A07, and sometimes, specificity is even slightly higher by comparing the mean metric values. In A08 to A10, the mean specificity is on the higher side, while in A11 and A12, the sensitivity is higher. Then, from A13 to A18, the specificity is once more on the higher side. Therefore, a continuous pattern is not seen in this aspect. Moreover, from A09 onward, the standard deviations of sensitivity and specificity tend to values higher than 14%. Therefore, high fluctuations are happening in different shuffles/runs. When the training data is scarce (roughly from A09 onward), the training of the model is not fully reliable, and as you also guessed, it could happen that the model tends toward one of the labels in its predictions.

19. The authors speculate that with more shuffles A11-A18 will likely converge to chance - this doesn't seem likely. You are already plotting/examining across 200 shuffles + repeats - there would have been reasonable indication that results are converging to chance. On the contrary, the specificity is still averaging around 80% (A18) and sensitivity around 50%. The authors say that looking into this is beyond the scope of the study - well, isn't it central to your model? If the model is, in the absence of any reasonable data (only four images), still predicting quite a bit above chance, what does it indicate? Seems like a suspect case of some sort of data leakage happening such that the model is still able to predict ~65% of the cases correctly.

Our response: Following your comment and to check our guess, we increased the shuffles to 50 (each shuffle run 10 times, therefore 500 in total) but the average accuracy remained the same in the mentioned settings, so our initial guess did not appear true. However, it is not necessarily true that the model should converge to the chance level. Even with 4 training images (in A18, 2 training+2 validation used in training), the model may learn to some extent. This especially makes sense in our setting. In A18, we have training data of only ADNI and OASIS, and around 60% of the test set is from these two datasets. In ADNI, 79% and in OASIS3, 92% of MRIs are from Siemens scanners. This could be the reason of accuracy of A18 being 67%. Also, when there are more protocols in training set but a small number from each of them, it is harder for the model to learn something compared to when there is less data with less protocols (as in higher A# numbers).
Moreover, it is not impossible to train a CNN even with only one MRI. See for example https://doi.org/10.1109/ACCESS.2019.2900198 which is published by the group of Prof. Xavier Llado. The have a scenario of training a CNN model for lesion segmentation with only one training MRI. The results are shown in their Table 6 in ORG cases.

20. To investigate the above, I had suggested permutation testing - however, the authors have only done that for A00; that does not tell us whether the tail end of the settings are still above chance or is 65% accuracy in the chance limits. If the latter, why is the chance performance not centred at 50%?

Our response: We thank you for your comment regarding the permutations results. Following your comment, we performed the permutation test for all the settings (1000 permutations per setting). The chance level appears to be 50%, however as mentioned in comment 19, we do not necessarily expect limited-data settings to converge to the chance level as the model maystill learn to some extent, at least in some shuffles/runs which will increase the average accuracy value.

21. In fact, for the accuracy plots, it would be meaningful to additionally include the permutation dots for each box plot (i.e., keep the box plots as such but additionally plot the jittered points from permutation testing for each of the scenarios). This establishes a reasonable distribution of how much performance can be expected by chance and the true distribution. The authors have argued that there is high computational cost - in settings like A18, isn't there only four MRI scans to learn from? Model training is the most cost intensive step, shouldn't the computational cost be minimal for such extreme settings?

Our response: Thank you for your suggestion of including the permutation results as jittered points in the accuracy plots. We added the permutation results for all settings as jittered points in the accuracy plots of the settings A, B, C, and D. Therefore, we removed the Supplementary Fig. S3 (added in the 1st revision) from the paper.

22. In setting B, we see the opposite effect of setting A - the specificity drops consistently while the sensitivity remains quite high - why does this happen? These results need to be examined and discussed.

Our response: In the same way as explained in response to comment 18, it is not straightforward to find out what really happened in the model training since it is not an explainable model. Again, as shown in Table 2, the training data size of B02 is the borderline of reliability of the model. The phenomenon mentioned in this comment is only happening beginning from B02. After B02, the models are not considered fully reliable.

23. Again, the authors have claimed that the accuracy will converge to chance with more shuffles and repeats - but these are already adequate repeats (200).

Our response: We increased the shuffles to 50 (also mentioned in response to comment 19 with more details) however our guess didn’t come true and the accuracy did not converge to chance level. Of course, talking about setting B, in B04 there are 20 MRIs used in training (10 training+ 10 validation). Therefore, it is not strange if B04 is not showing chance level and is in fact, learning something.

24. I do not see permutation testing results for settings B, C, or D.

Our response: Following your comment, we performed the permutation test for all the settings (1000 permutations per setting) despite the very high computational cost and added the result to the accuracy plots.

25. For setting C, between C02 and C03, the sample sizes are not that different: 46 and 36. However, the specificity of your models plummet from 85% to 30%. Similar to setting B, we see quite high sensitivity throughout (even in C06; >85%) while the specificity is the only one which takes a hit. Seems to be a scenario where the majority of the times the prediction is a positive class?

Our response: Thank you for your question in this regard. Based on Table 2 of the paper, C02 has the borderline training size for the reliability of the model. After C02, the model fails to be trained well and tends toward the chance level. In C04 to C06, we see the convergence to the chance level in accuracy. However, in C03, still some of the models have been trained to some extent and have shown higher accuracy than 50% but the average specificity has dropped drastically. In general, we are not expecting a linear behavior in the performance of the model by decreasing the training size, especially when passing from the borderline training size when the model performance drops.

26. Regarding the possibility of overfitting to the test, the authors argue that they have "not learnt any lesson from the first test". The biggest lesson learnt from the first test was the fact that 2D images were a source of poor performance. Not only was this lesson learnt, but a decision was made from this lesson - the decision to eliminate 2D scans. While it is true that the authors have recreated the models and run the tests in multiple shuffles, it does not avoid the problem of overfitting to the test set. You have looked at the entire data, made a decision, and then re-run the pipeline which results in better performance. This is problematic and needs to be adequately mentioned with the results considerably downplayed, with an acknowledgement that this could be overfitted to the test.

Our response: Thank you for your concern regarding overfitting of the result of setting C. In this regard, we added the following sentence to the results section for setting C:
"
However, from a certain perspective, the decision to exclude 2D MRIs could be interpreted as a form of overfitting to the data characteristics.
"

27. Usually, one begins to see convergence to chance in 50-100 trials; in your experiments, there are 200 trials. It is a bit suspect that none of the extreme scenarios have converged to chance.

Our response: Thank you for your comment. Regarding settings A and B, please see the responses to comments 19 and 23, respectively. In setting C, the last three models (C04 to C06) are already showing convergence to the chance level by having an accuracy of less than 53.5%.

28. It is hard to visually compare plots given the authors decision to not make the axes equal across; if they are concerned about some of the values not being very visible, they can always include zoomed in versions of the plot.

Our response: Following your suggestion, and after adding the permutation results as jittered points in the accuracy plots, we made the axes of all plots equal (from 0 to 100%). Please see the new plots in the paper.

Overall comments
29. Please include relevant permutation testing and the distribution of values for all scenarios. They are essential more for evaluating how much performance is expected by chance rather than for calculating p values (which can be trivially small).

Our response: Following your suggestion, we added permutation results for all settings and removed the previously reported p-value from the paper.

30. Regarding citing prior literature: as mentioned in the first round, the authors have completely glossed over any direct prior work done in the area. There is a lot of work that has happened in the area: if these methods/papers produce results which are slightly different than the authors' result (i.e., labelling of white matter hyperintensity vs. binary output or volumetric assessment vs. binary output), it does not mean that prior work can be glossed over. I strongly suggest that the authors bring in (in the introduction section at least) the prior work in the field and highlight why and how their approach is different.

Our response: Thank you for your comment regarding the missing literature in the introduction. In this regard, we added the following paragraph to the introduction:
"
Several existing methods address WM abnormality analysis, such as lesion segmentation or volumetric assessments, typically using supervised deep learning models. Many of these approaches require large amounts of voxel-wise, manually annotated data for training~\cite{Zhu2022, MU2024107904, Kuwabara2024}. Some methods specifically target the segmentation of lesions within the brain~\cite{GUERRERO2018918, CETIN2020100409, DeepWMH, WHITE-Net, LPA}, whereas this study focuses on classifying MRIs based on the WM abnormalities. Additionally, several segmentation models require the availability of multiple MRI sequences (e.g., FLAIR, T1, T2)~\cite{TRAN2022102940, DeRosa2024, VALVERDE2017446}, while the approach presented in this study is designed to operate solely on FLAIR images, enhancing its practicality and ease of deployment across heterogeneous clinical datasets.

"

31. On just doing a quick Google search on the topic, I found a series of relevant papers which have used ML/DL approaches for WM lesion detection / labelling. Examples of these include: Zhu et al (2022, Frontiers in Aging Neuroscience), Guerrero et al (2018, NeuroImage Clinical), Tran et al (2022, NeuroImage Clinical), Mu et al (2024, Computer Methods and Programs in Biomedicine), Kuwabara et al (2024, Scientific Reports), Zhang et al (2022, Neuroradiology), to name just a few of the ones I found. In fact, a few of these (and the comparators cited therein) are extremely relevant papers since they use similar approaches as the present work. For example, Zhu et al have done a series of comparisons in their work and they cite to other state of the art methods (back then) such as uResNet, 3D V-Net, and VGGNet. Guerrero et al point to DeepMedic. Beyond these DL methods, there are other series of methods that exist such as lesion prediction algorithm, lesion segmentation tool, lesion growth algorithm, BIANCA, and probably many more. And these are based on a quick search, not a deep dive into the literature; now some of these may not have the code available (to be used as a comparator) or may not be directly relevant due to whatever reason, but to completely miss the entire field in the introduction/discussion and state that there is no comparison possible at all is incorrect.

Our response: Thank you for your points regarding related papers. Preferably, the models that are to be compared need to be trained with the same data as well, so the models can be fairly comparable. However, this was not possible due to the differences between HeteroMRI with other methods. For example, most models either need lesion annotations or a very large amount of data, or require multiple MR sequences from each subject. Finally, for comparison, we could find and implement three WM lesion segmentation methods that require only a FLAIR image per subject in the same way as HeteroMRI but every method is trained/developed on different data. We added the new subsection "4.6 Comparison with related methods" with the following text and Table 3.
"
To further evaluate the performance of HeteroMRI, we applied it on a hold-out set of data and compared the performance with three lesion segmentation methods: DeepWMH~\cite{DeepWMH}, WHITE-Net~\cite{WHITE-Net}, and the Lesion Prediction Algorithm from the Lesion Segmentation Tool (LST-LPA)~\cite{LPA}. These methods are not designed for direct binary classification of MRIs. To enable a fair comparison, we post-processed their segmentation outputs by binarizing the masks through 3D connected components analysis (using the \texttt{ConnectedComponentImageFilter} class from \texttt{SimpleITK}~\cite{SimpleITK} \textit{Python} library): if at least one WM lesion consisting of \textit{five} or more 3D-connected voxels was detected, the corresponding MRI scan was labeled as positive. The comparison was conducted using datasets 10 and 11, introduced in Sec.~\ref{sec:datasets}. The test set includes 20 MRIs in total, with balanced labels and images acquired from nine different MRI protocols. These datasets serve as holdout sets for HeteroMRI and were not used during training or testing in any of the experimental settings. For HeteroMRI, we employed the 500 models trained in setting $A00$. The final prediction label for each test scan was determined by majority vote across these models. The DeepWMH, WHITE-Net, and LST-LPA are pre-trained, ready-to-use models that do not require retraining. All of the methods require only a FLAIR image per subject. Table~\ref{tab:compare} reports the accuracy, sensitivity, and specificity for all methods Based on the results, HeteroMRI outperforms DeepWMH, WHITE-Net, and LST-LPA in accuracy and specificity, while the other methods have a higher sensitivity.
"

32. The authors argue that the value of an appropriate threshold needs to be set for using many of these methods as a comparator. Fair point. However, it is not that difficult to do so. For example, Zhu et al used a 3D connected component to binarize the output - if you have even one connected component standing, treat it as WMH true. Guerrero et al discuss thresholds for some of the methods in their section of comparison to gold standard.

Our response: We thank you for your suggestion of using 3D connected components. We employed this method for binarizing the output mask of the three segmentation methods which we compared our results with. Further details are explained in response to comment 31.

33. Similarly, the need for a training set in BIANCA is well known - there is publicly available training set; see, for example, https://git.fmrib.ox.ac.uk/open-science/analysis/wmh_harmonisation/-

/tree/master/BIANCA_training_datasets

Our response: Thank you for proving the link to training files for BIANCA for facilitating our analysis. Unfortunately, we couldn't include BIANCA among the methods since the training sets presented in the link are only for the cases that there is both FLAIR and T1 image from each subject. As we are considering models that use only a FLAIR image per subject, we did not include BIANCA method.

34. The whole point of the comparison is to gain a perspective on how well HeteroMRI is doing compared to what is already done in the field. This is especially true since several of the DL based methods (some of which I cited above) make the exact claim that their method is robust to varying protocols.

Our response: Please see the response to comment 31 regarding the comparison with other methods.

35. Regarding out of sample validation: while it is true that the authors have attempted to show the performance of HeteroMRI in leave-a-site-out kind of design, it would be more convincing to see the results in a truly out of sample situation. The ideal setting would be when you mix a few images from a range of studies and treat them as truly out of sample (i.e., not include them in the train-validation-test design of your experiments). There is little computational cost to this because your model is already trained. You are taking the pre-trained "final" model that you have created and are merely applying it to out of sample images which may come from different scanners/protocols/sites. In this setting, the held out sample is not biased because it contains diverse protocols and ideally has data independent of any of the protocols used in the study. Again, this is not a combinatorial problem and does not have any significant computational cost since the model is being applied to the test set. Class imbalance does not picture in this setting because you can compute sensitivity, specificity, and balanced accuracy irrespective of class imbalance. If the authors cannot identify any scenario at all where they have external dataset to work with (that would be hard to believe given the extensive work that has already happened in the field with several papers sharing manual annotation and raw images), one could still take all the unused FLAIR images from ADNI, OASIS, and other datasets (irrespective of case status) and do an out of sample application. Once again, class imbalance is not an issue here. The overall point of these comments is that in the absence of any comparators and any out of sample application, it is hard to truly evaluate the performance of any DL model.

Our response: Thank you for your comment regarding out-of-sample testing. We added two new datasets to the study as hold-out sets that are also used for comparison with other methods. The following changes are made in the paper in this regard:
-Table 1 is modified to include the two new datasets
-In subsection "3.1 Datasets", the introduction of the two new datasets (WMH, PPMI) are added as follows:
"
WMH: The White Matter Hyperintensity (WMH) segmentation challenge~\cite{WMH} includes in total MRIs from 170 subjects from five different scanners. The MRIs have different loads of WM lesion. In this paper, we used 10 FLAIR images from this dataset. From each scanner, two MRIs were taken (the first training and the first test MRI, or the first two test MRIs).

PPMI: The Parkinson's Progression Markers Initiative (PPMI)~\cite{PPMI} is a large-scale, longitudinal study that offers comprehensive imaging, clinical, and biospecimen data from individuals with Parkinson's disease and healthy controls. For this study, we selected 10 FLAIR images without WM abnormalities---identified by a neurology specialist---from approximately 220 healthy control subjects with available FLAIR scans.
"
-The hold-out test concept was also mentioned in subsection 4.6 (please see the response to comment 31)

36. One of the reasons for why the authors are finding it hard to find a comparator which gives binary response to an input FLAIR image is the question "how useful is a binary classifier over a WM abnormality labelling method?". The authors mention that their goal is to use the method (in the future) for classifying MRIs based on white matter diseases. A similar evaluation was performed in Guerrero et al (WMH vs. stroke lesion) and probably in other work too. Even in that scenario, would it not be reasonable to get annotation maps out of HeteroMRI? You can always convert labelled images into a binary decision but not the other way round. We need ML models to assist clinical decision making by providing reasonable probabilities and (in case of images) labelled data so that the clinician can use the data for informed decision making; not make the decisions for

the clinician. If the use case audience are medical trainees and MRI operators, then those should have been identified rather than broad clinical practice in the introduction. Overall, these aspects need to be discussed and also listed as limitation of the proposed method.

Our response: Thank you for your concern. We added the following sentence to the limitations in this regard:
"
The current classification task of HeteroMRI may be less interesting to apply in clinical practice compared to, for example, segmentation or volumetric measurement methods. However, this paper is supposed to present HeteroMRI method and evaluate its performance on heterogeneous data. In future work, the methodology is planned to be employed for the disease-specific classification of MRIs based on WM abnormalities by learning from subtle lesion patterns.
"

37. To come back to the point of the WM clustering step making the method robust to multiple MR acquisition protocols, here is a possible test scenario: if you took white matter segmentation probability maps from a standard package (SPM, CAT, FSL, etc.) and repeated the leave-one-protocol-out type of test, would you see comparable performance across the folds? In this scenario, the actual performance of the model does not matter much - it is the stability of the performance (even if it is say 65%). If it is the WM clustering step which brings in the robustness to protocol differences and not the DL method, one could also flip it around and use any other ML/DL method/architecture with the segmentation maps and they ought to result in robust performance.

Our response: Thank you for suggesting this interesting test scenario. We may carry out this test in future studies. For the moment, as mentioned in the response to comment 9, we changed the sentence related to this point in the paper accordingly.

38. Please include a discussion about the limitation of the current work since all the evaluated scenarios are idealised balanced case which are far removed from the clinical setting.

Our response: Thank you for your concern. We added the mentioned limitation to the discussion section. Also about the balanced case, we added the following sentence to the Discussion in this regard:
"
Moreover, the performance of the method could be studied in case of unbalanced training data.
"

##
Reviewer 2
##

Reviewer #2: The manuscript has been revised in accordance with the majority of the comments raised during the first round of review.

Cookie Preference Center

We use cookies which are necessary to make our site work. We may also use additional cookies to analyze, improve and personalize our content and your digital experience. For more information, see our Cookie Policy.

You may choose not to allow some types of cookies. However, blocking some types may impact your experience of our site and the services we are able to offer. See the different category headings below to find out more or change your settings.

Allow all

Manage Consent Preferences

Strictly Necessary Cookies

Always active

These cookies are necessary for the website to function and cannot be switched off in our systems. They are usually only set in response to actions made by you which amount to a request for services, such as setting your privacy preferences, logging in or filling in forms. You can set your browser to block or alert you about these cookies, but some parts of the site will not then work.

Functional Cookies

These cookies enable the website to provide enhanced functionality and personalisation. They may be set by us or by third party providers whose services we have added to our pages. If you do not allow these cookies then some or all of these services may not function properly.

Performance Cookies

These cookies allow us to count visits and traffic sources so we can measure and improve the performance of our site. They help us to know which pages are the most and least popular and see how visitors move around the site.

Targeting Cookies

These cookies may be set through our site by our advertising partners. They may be used by those companies to build a profile of your interests and show you relevant adverts on other sites. If you do not allow these cookies, you will experience less targeted advertising.

Cookie List

Clear

Apply Cancel
Consent Leg.Interest

Confirm my choices

Powered by onetrust
