# Supplementary material for: HeteroMRI: Robust white matter abnormality classification across multi-scanner MRI data
Source: Gigascience. 2025 Aug 21;14:giaf092. doi: 10.1093/gigascience/giaf092 (PMC12371411; doi:10.1093/gigascience/giaf092)
Supplement: giaf092_Reviewer_1_Report_Original_Submission — Pravesh Parekh -- 8/17/2024 [file giaf092_reviewer_1_report_original_submission.pdf]

Reviewer Report

Title: HeteroMRI: Robust white matter abnormality classification across multi-scanner MRI data

Version: Original Submission **Date:** 8/17/2024

Reviewer name: Pravesh Parekh

Reviewer Comments to Author:

The manuscript by Abedi et al have proposed a new deep learning method to predict if a given FLAIR image has white matter abnormalities. The CNN-based method looks interesting and seemingly has quite high performance (up to 96%). However, I have a few concerns: there is no comparison analysis presented, the study design prevents any independent assessment, there is no permutation testing, and very limited clinical utility if the method merely gives a binary decision.

Detailed notes below:

Title

1. The title of the paper mentions "classification of multi-scanner and multi-protocol brain magnetic resonance images with deep learning" - however, from this title, it appears that the paper is about differentiating between multiple scanners and protocols. Put another way, the title doesn't specify the classification task at all. It eithers ends up giving an incorrect impression or fails to answer the question "classification of what"? Suggest updating the title to reflect the content better.

Abstract

2. "This study introduces HeteroMRI, a deep-learning method for analyzing MRI datasets incorporating various MRI protocols and scanners" - this seems a bit misleading as HeteroMRI is neither a harmonization method nor is it a general purpose analyses method; this should be rephrased to clarify that HeteroMRI only gives binary predictions of WM abnormalities only using FLAIR images.

Introduction

3. Trivial point: page 2, line 52, for consistency, better to introduce the abbreviation FLAIR in the bracket, rather than the other way around.

4. Page 3, line 79, the authors mention that "Before analysing brain MRIs, certain preprocessing steps are commonly performed to optimize the image data for further analysis and interpretation"; at the end of the paragraph, the authors mention defacing which is incongruous to the rest of the paragraph - protecting the privacy of individuals using defacing methods is not a step performed for optimizing the images for any analyses or interpretation - it is a separate issue. I suggest removing that line as it doesn't connect with the rest of the text. It is also not something that the authors themselves re-visit at any point in the manuscript (or something they address).

5. Page 3, lines 79-83, the authors mention a range of methods that are commonly employed in (pre-)processing of MR data - however, they do not mention image normalization (i.e., transformation from native space to standard space; although one could argue that it is part of the registration step; note that this is different from intensity normalization), image segmentation, and smoothing (although one could argue that it is related to interpolation and denoising).

6. Page 3, line 86, reference 38: perhaps I missed it while looking into the reference, but I did not see a

recommendation that images should be acquired "with the same acquisition protocol, with the same magnetic field strength, and preferably with the same MR scanner model" (or "standardized") - in fact, the reference proposes a harmonization method.
7. Page 3, line 86, references 37 - 39: I fail to see how the generalization that "it is highly recommended that all the images are standardized" follows from these, where the authors mention that standardized implies same acquisition protocol, same magnetic field strength, and preferably the same MR scanner model.
8. Generally, it is true that if the training data and the test data are very different (where differences could originate from different scanner make/models, field strengths, acquisition protocols, etc.), a ML/DL model may fail to generalize. However, that does not imply that "standardization" is the way to go. In other words, while creating a standardized dataset may improve performance in a test scenario, it would never work in a clinical setting (which is what the goal of the paper is).
9. "Using a standardized dataset ensures that the images have similar image quality, similar intensity range for each tissue, and similar spatial characteristics." - this is merely a theoretical guarantee; it doesn't really work in practice.
10. "Moreover, the contrast among the brain tissues would be uniform across the dataset [40, 37]" - this is not necessarily true; also, doesn't seem to be implied from these citations.
11. The overall issue with this paragraph (page 3, lines 85-94) is that the authors are contextualizing everything as generally true while picking very specific nuanced notes from the field of multiple sclerosis.
12. Page 4, line 114, "The methods based on statistics alter the intensity distribution in the image…" is not entirely accurate - methods like ComBat (references 46-48) are generally meant to be used on features derived from images and not directly the image intensity itself.
13. "In this paper, to circumvent the complex MRI harmonization, we present HeteroMRI, an approach for analyzing multi-protocol and multi-scanner MRI data." - well, HeteroMRI has 1,795,905 parameters and therefore is not simple in anyway. Additionally, this doesn't circumvent harmonization in anyway - perhaps I am missing what the authors really wanted to say here.
14. Overall, I found the introduction section to be very hard to follow. The introduction starts with a brief remark about WM abnormalities and then goes on to talk extensively about other topics (MR preprocessing, use of ML/DL methods, and an extensive discussion about harmonization). However, it is only towards the end of the introduction that the authors return to WM abnormalities and even then, the goals are a bit unclear - is HeteroMRI a way to harmonize the data or is it a way to detect WM abnormalities which is robust to scanner effects? Throughout the introduction, the authors heavily cite literature from MS and other very specific neurological applications but continue to phrase the introduction very generically. In fact, even towards the end, when the authors state "we present HeteroMRI, an approach for analyzing multi-protocol and multi-scanner MRI data" - even at this point it is unclear what this analysis method is supposed to do - it is only a few sentences later, "Herein, the presented approach is evaluated for detecting the brains with WM abnormalities through binary classification." that it becomes clear that the method is supposed to identify WM abnormalities. This ambiguity is also applicable to the title where the title states the goal being classification but doesn't specify classification of what. I strongly encourage the authors to edit the introduction to have a stronger focus.

15. Page 5, line 171: "The presented method opens new avenues for analyzing heterogeneous MRI datasets and the large amount of MRI data generated daily in medical centers" - I don't quite see how. HeteroMRI can classify WM abnormalities as yes and no; it is not a general-purpose analyses tool nor is it a harmonization method. Therefore, "the presented method opens new avenues for detecting WM abnormalities in heterogeneous MRI datasets".
Methodology
16. "The WM abnormality detection approach presented in this paper uses heterogeneous brain MRI data with various acquisition protocols (multi-protocol) as the data for a Convolutional Neural Network (CNN). The model is a binary classifier trained to detect patients with WM abnormalities in their brain MRI." - this is the first place where the authors have very clearly stated what they do and their goal! This clarity, if added back to introduction, will be very useful to the readers.
17. Page 7, line 189, what about the number of voxels in the third dimension?
18. Page 7, line 192: "Detailed information on the parameters used in each step is reported in the GitHub repository of HeteroMRI" - on the GitHub page [https://github.com/ul-mds/HeteroMRI/tree/main], I do not see any details of what FlexiMRIprep settings / configuration was used for which dataset.
19. Does this imply that different processing strategies / settings were used for each dataset? If yes, why? What was differently done for which datasets? Please include these details in the manuscript.
20. Page 7, line 202: "This template is created using the data from the ICBM (International Consortium for Brain Mapping) project" - does this mean you created your own template? I think not, but please clarify / edit the sentence.
21. Why was the registration step performed three times - the authors claim that this leads to "most precise alignment" - how was this established/judged?
22. At the end of registration + normalization step, what was the final voxel size and image size?
23. Since some of the dataset had 2D images (many in OASIS, ISBI, and BTH), were they all normalized to some fixed voxel size and overall size?
24. From Fig. 1, it appears that all input images were 193 ï‚´ 229 ï‚´ 193 - that seems like a massive up-sampling for images which only had 22 or so slices. How did the authors arrive at this resolution?
25. The WM extraction step is not clear at all - once the FLAIR images are aligned with the MNI template, the voxels still represent some (bias corrected and) interpolated version of the arbitrary MR signal from the raw data. The MNI WM probability map, on the other hand, has values between 0 and 1 showing the probability of that voxel being white matter. A simple multiplication of these two doesn't make sense, given that there will be arbitrary differences between subjects simply because of the scanner intensity scaling differences. The actual voxel intensity per se doesn't have a lot of meaning to it.
26. Was the WM probability map binarized?
27. Since there doesn't seem to be any intensity normalization / correction / rescaling, how does the method account for extremely different intensities between different MRI vendors? For example, for data that comes from a Philips scanner, the intensity values would be very high (provided Philips precise scaling is turned on when converting images from DICOM to NIfTI). This would imply intensity values of tens of thousands while the ones from Siemens would be in a few hundreds (typically). Bias correction would not (completely) account for this (since it is not performed across images but within images).

28. Page 7, line 215: "Calculating and obtaining a …", it is just one operation being performed.
29. Visually, it appears that cluster 3 is merely an eroded version of WM segmentation - did the authors try comparing cluster 3 with an eroded version of WM segmentation from standard neuroimaging tools?
30. "We tested various cluster numbers on different MRIs…" - were these the same dataset that were eventually used for training and test?
31. "For this study, we manually identified the intended cluster for each MRI by choosing the cluster that looks visually similar to Cluster 3 in Fig. 1." - that doesn't sound like a robust strategy and requires manual intervention. This needs to be addressed.
32. I don't think a DL model to identify the right cluster is necessary - at least a visual examination of the three clusters in Fig. 1 indicates that some combination of standard image processing tools and the right evaluation metrics (like Dice coefficient) might solve the problem. Since the authors have already done the manual identification, it should be easy to evaluate any solution for robustly identifying the correct cluster image.
33. For the entire DL design - what was the rationale for implementing this specific configuration? In other words, how did the authors arrive at this precise configuration? Some aspects seem standard while others represent an arbitrary choice - was any of this optimized? If yes, using which dataset?
34. Table 1: would be additionally helpful (to the readers) to include a hyperlink to the datasets or the page where the dataset can be requested.
35. The MSSEG paper mentions 53 images - why was one image dropped in the current study?
36. ICBM dataset - only five out of 20 did not have WM abnormalities; can't the remaining 15 be used as positive cases (just like in OASIS-3)? Why were they removed?
37. For OASIS-3, from a pool of 600 images, only 90 were identified without WM abnormalities; then, how are there only 14 cases with WM abnormalities? Either some detail is missing here or one of the numbers is incorrect - from the document on GitHub, it looks like 90 and 14 are correct - that means there was a massive pool of images with WM abnormalities, but they were not used in the study? Why not?
38. ADNI: First, please specify if you examined across ADNI1, ADNI2, ADNI-GO, and ADNI-3? Second, what was the total number of available images? Third, if only 58 images with WM abnormalities were identified, shouldn't there be a good number of control images from ADNI? Why were they not used?
39. CERMAP: The remaining 10 subjects who were identified as having minor lesions - why were they not used as cases?
40. Coming back to BTH: the dataset has 60 2D FLAIR images - however, only 9 were used in the present study. The authors mention a problem with the 2D images; however, many images in the OASIS dataset had similar number of slices: specifically, 8 images with 24 slices and 86 with 35 slices. What was the strategy used for processing the OASIS images and couldn't the same be used for BTH?
41. Overall comments for the data section: the rationale for what was included and not, is not specified / is unclear. Please provide more details. There seems to be a large number of cases/controls that could have been included but were seemingly not.
42. What about the age and sex distribution of the samples from each of the site? These are important variables to consider when evaluating whether there are systematic differences in sites or between cases and controls within a site.
43. There is no mention of quality checks performed on the images prior to their inclusion in the analysis

- this is critical, especially given that the authors later remark about registration / segmentation issues - please provide documentation on what were the QA/QC procedures employed. If these were not done, there needs to be a very good reason why not.
44. For each of the settings: as a first sentence, can you please state the goal of the experiment/setting? It would be helpful for the readers to get oriented in the first sentence itself of the goal of the experiment.
45. I found supplementary table S1 hard to follow; it was easier to use the Excel sheet from the GitHub repository of the authors - perhaps the first part of the table (where the dataset breakdown is not present) could be additionally included in the supplementary materials?
46. Page 11, line 229: "The number of MRIs with and without WM abnormality is balanced in the training data of all the settings." - wasn't this the case for validation and test sets as well (and not just for training)?
47. What was the rationale for enforcing equal number of cases and controls in training, validation, and test sets? Was it merely convenience to ensure that training was not biased by class imbalance?
48. The authors had 222 positive cases and 122 negative cases; in setting A, 100 positive cases were removed - on what basis? And which ones?
49. Even if the authors insisted on balanced training and validation split, couldn't these 100 be used for external holdout testing?
50. Setting B: there are total 32 protocols; it is unclear how only 10 protocols were sampled. What was the goal of setting B?
51. Overall, the experimental design section was hard to follow; perhaps responding to and adding clarification based on my previous comments might improve the readability of this section.
52. Generally speaking, the equal number of cases and controls situation is merely an idealised scenario and does not reflect practical clinical setting.
Results and Discussion
53. For Figures 3 and 4, please include a slice from the registered MRI that corresponds to the slice presented in the WM intensity cluster - that will make it easier to compare the image used for learning/prediction vs. the image from which the cluster was derived.
54. It is a bit hard to follow how the authors are concluding that MRIs 4, 5, 6, and 10 have incorrect registration - the cluster images still capture white matter. Perhaps including additional images on what went wrong would help underscore this point?
55. It seems a bit remarkable that even in A18 or B04, the average accuracy is ~67% and 65% - since the datasets have been (artificially) balanced between cases and control images, in extreme paucity of data (such as 1 + 1 in A18), one wouldn't expect any meaningful learning to happen - and therefore, the accuracy should fall to chance level: 50%.
56. In fact, in B04, the accuracy looks to be ~65% which is ~222/(222+122) i.e., the original cases and control proportion, considering majority class. Can the authors comment on this?
57. Page 18, lines 487 - 491, the authors have inadvertently ended up overfitting to the test set - fitting a model and examining the performance on the test, followed by a decision to change the model, refit, and report performance on the same test set. This is problematic; at the bare minimum, the authors should include (in the supplement), the results from the original situation of using 2D images and acknowledge the fact that these results are obtained after examining the prediction performance on the

test set and then re-doing the modelling (overfitted to test).

58. Why does the average performance of C06 look a little better than C05? As mentioned in my previous comment, since there is barely any meaningful learning happening at such a reduced sample size, we expect accuracy to hit 50% (balanced cases and controls) - and we see that in C05; but C06 performance, surprisingly, goes up a little bit - doesn't seem to be noisy variance around 50%.

59. A general critique of all the box plots - can you make sure they have the same ticks and tick spacing to ensure easy comparability across accuracy, sensitivity, and specificity? Also, please mention that the triangle marker indicates the mean - doesn't seem to be specified anywhere.

Overall feedback

60. Altogether, it is important to assess how these models perform compared to chance - the authors have not performed any permutation testing. Since the image preprocessing and cluster detection is ready, it should be relatively easy to perform permutation testing and include these results.

61. Additionally, the authors have not performed any comparison with any other method - for example, a trivial comparison would be to take any of the commonly used methods for detecting WM abnormality using FLAIR (for example, FSL's BIANCA), pass the images through it, and convert the WM hyperintensity segmentation into yes/no, which can then be compared with HeteroMRI performance.

62. Similarly, there are several other ML/DL based methods for detecting WM hyperintensities (including using convolutional neural network). The authors have not mentioned any prior work done in this regard nor have they attempted to compare their method with any of the recent work in the area; this is important to assess.

63. One of the biggest drawbacks of the study design is that there is no holdout set - given the propensity for deep learning methods to overfit, it would have been more convincing to see an external evaluation (outside of the train-validation-test framework) - preferably even a site that was never used. It may perhaps still be possible, given large number of additional FLAIR datasets available.

64. Related to previous comment, except for OASIS-3, all datasets used in the study have either cases or controls but never both - since we do not really see the identified lesions/WM changes (except for cluster3 image), could some of the performance be attributable to merely learning some image characteristics of the site (which correlates to case status)?

65. Another thing to consider is that HeteroMRI only gives a binary classification output - it does not highlight or mark the suspected regions of WM abnormalities/changes; this makes it hard to evaluate the method from other approaches that attempt to label the changes. It also reduces the applicability of the work in a clinical setting where a radiologist might be interested in having an AI tool to help locate WM changes (rather than decision making, which anyway requires a physician/radiologist to look at the image before signing off a report).

66. On a related note, is it possible to apply a reverse (or inverse) deformation field that warps the WM cluster 3 images back to native space? That would improve the utility of the present work and allow examination of what exactly went wrong for false positives and false negatives. This is a solved problem - most non-linear registration tools should be able to generate forward and reverse warps.

Methods

Are the methods appropriate to the aims of the study, are they well described, and are necessary controls included? Choose an item.

Conclusions

Are the conclusions adequately supported by the data shown? Choose an item.

Reporting Standards

Does the manuscript adhere to the journal's guidelines on minimum standards of reporting? Choose an item.

Choose an item.

Statistics

Are you able to assess all statistics in the manuscript, including the appropriateness of statistical tests used? Choose an item.

Quality of Written English

Please indicate the quality of language in the manuscript: Choose an item.

Declaration of Competing Interests

Please complete a declaration of competing interests, considering the following questions:

- Have you in the past five years received reimbursements, fees, funding, or salary from an organisation that may in any way gain or lose financially from the publication of this manuscript, either now or in the future?
- Do you hold any stocks or shares in an organisation that may in any way gain or lose financially from the publication of this manuscript, either now or in the future?
- Do you hold or are you currently applying for any patents relating to the content of the manuscript?
- Have you received reimbursements, fees, funding, or salary from an organization that holds or has applied for patents relating to the content of the manuscript?
- Do you have any other financial competing interests?
- Do you have any non-financial competing interests in relation to this paper?

If you can answer no to all of the above, write 'I declare that I have no competing interests' below. If your reply is yes to any, please give details below.

I declare that I have no competing interests

I agree to the open peer review policy of the journal. I understand that my name will be included on my report to the authors and, if the manuscript is accepted for publication, my named report including any attachments I upload will be posted on the website along with the authors' responses. I agree for my

report to be made available under an Open Access Creative Commons CC-BY license (http://creativecommons.org/licenses/by/4.0/). I understand that any comments which I do not wish to be included in my named report can be included as confidential comments to the editors, which will not be published.

Choose an item.

To further support our reviewers, we have joined with Publons, where you can gain additional credit to further highlight your hard work (see: https://publons.com/journal/530/gigascience). On publication of this paper, your review will be automatically added to Publons, you can then choose whether or not to claim your Publons credit. I understand this statement.

Yes Choose an item.
