# Supplementary material for: HeteroMRI: Robust white matter abnormality classification across multi-scanner MRI data
Source: Gigascience. 2025 Aug 21;14:giaf092. doi: 10.1093/gigascience/giaf092 (PMC12371411; doi:10.1093/gigascience/giaf092)
Supplement: giaf092_Reviewer_1_Report_Revision_1 — Pravesh Parekh -- 12/24/2024 [file giaf092_reviewer_1_report_revision_1.pdf]

Reviewer Report

Title: HeteroMRI: Robust white matter abnormality classification across multi-scanner MRI data

Version: Revision 1 **Date:** 12/24/2024

Reviewer name: Pravesh Parekh

Reviewer Comments to Author:

The authors have revised their previous submission and have adequately responded to several of the comments. Nonetheless, there are still several concerns that remain with the study, including but not limited to lack of comparisons with other methods, lack of integration with the existing literature, incomplete permutation testing framework, and a reluctance to perform any kind of external validation. Detailed comments follow.

Introduction

1. The introduction is still quite long and wordy with needless diversions and forays into areas which generally do not lead to anything. There is plenty of avenue to cut down and make the introduction focused/sharp - for example, most of the first paragraph is superfluous (except for T2 FLAIR), the second and the third paragraphs can easily be slashed altogether, elaboration on the standardized datasets can be compressed into two or so sharp sentences, the harmonization section and all the citations therein do not directly bring much to the table except for the point that it is important, and the elaborate paragraph on the limitations of ML/DL methods is not necessary.

2. Page 4, line 111-112, "Literature concerning harmonization techniques is relatively limited" - not sure where the authors get that impression from. Over the past decade or so, extensive work has been undertaken in the field to not only understand the sources of MR signal variability but also in quantifying the scanner effects as well as developing ways to deal with these. Same holds for the subsequent statement about there being an "absence of standard criteria for assessing scanner effects and evaluation harmonization process" - there have been a few proposals in the field on quantifying the scanner effect as well as evaluating harmonization process. While this area is nascent, work in the area is not absent.

3. Page 4, line 125, the authors imply that supervised ML harmonization techniques commonly use "manually selected features" - the authors have cited references 51 and 52. In 51, Garcia-Dias et al never performed feature selection but used all 68 IQMs + PCA; in 52, Jog et al compute various features to create synthetic MRI datasets - it is not primarily designed as a "harmonization" method (even though it can be used to achieve intensity standardization which is not equivalent). The point is, the authors are exaggerating the differences between ML/DL approaches which is not well backed up by the literature.

Methods

4. "All selected images have a minimum of 128, 192, and 22 voxels in their first, second, and third dimensions, respectively" - do you mean 22 voxels or 22 slices in the third dimension?

5. The authors have used T2 template for registering FLAIR images - did they run any tests prior to this step to judge whether this was an appropriate decision? For example, ADNI has a large number of T1 weighted images. One possible test could be to see if the T1 images registered to T1 template and then

the registration applied to the FLAIR images gives comparable results as FLAIR images registered to T2 template.

6. "This repeated registration aims to achieve a high level of alignment of the MRIs with the template." - the authors have not really answered my question. How was the high level of alignment judged / quantified? This is really critical because the authors are using a T2 template with a different contrast property than the FLAIR images as well as the fact that there are several 2D images in the dataset. How was the accuracy of alignment established for 2D and 3D images?

7. So, the images were indeed massively upsampled to 193 229 193 - this is remarkable, especially for the 2D images that had 22 voxels (slices?) to start with. Can the authors include snapshots of a few 2D images with the raw and normalized images put next to each other? I find the decision to upsample so much quite baffling - the authors could have easily chosen a lower resolution to upsample to (for example, 2 or 3 mm isotropic) without modifying the template.

8. For the WM extraction step - I still don't see the rationale of multiplying arbitrary MR intensity values with the probability values. It would be straightforward to take the probability values, apply a minimal threshold to convert it into a binary mask, and then do the multiplication - it still gives you the WM images and you retain the distribution of the MR intensity values (albeit only the white matter) rather than converting them to some sort of probability weighted distribution. If the latter is useful, that needs to be shown; if not, this step is really a strange image processing decision that is not commonplace and warrants some justification. The authors argue that this was done to "keep any voxel that "might" be white matter and ignore the rest of the voxels" - well, that would be easily achieved by a binary mask. In fact, a binary mask is going to result in a more accurate map ignoring of the rest of the voxels because they would be set to zero rather than some arbitrary (relatively small) number.

9. In the WM clustering step, the authors state "As a result, the impact of the heterogeneous nature of the multi-protocol MRIs is minimized" - how? The logic of this is not clear to me. If the reasoning is that the images are converted to three separate probability map images with values between 0 and 1 and this somehow leads to "minimization" of the impact of different MRI protocols, then the same could have been achieved with any processing stream that results in probability maps. At best, all this results in are (intensity-wise) comparable images. It does not minimize the impact of different MRI protocols or at least it does it in the same way that any standard processing stream would do (which is to say barely).

10. Table 1: while it is true that hyperlinks could change over time, that doesn't prevent the authors from including the hyperlink they used to access / request access to the dataset. It also makes it easier to reproduce the analyses from the paper since we know exactly where to go to get the datasets that you used, rather than attempting to trace it through guess-estimates.

11. MSSEG - you can include a footnote to indicate that one subject was inadvertently omitted from the analysis.

12. For the datasets where a subsample is used in the paper, please include a note stating that this was done (i.e., in the description mention the total images in the dataset, the number of images that were selected, and how the selection was done (e.g., randomly selected).

13. The authors have stated that not all the datasets available had demographics information - they can still report the age and sex distribution for the datasets that provided demographics information. My point was not to use this for any kind of prediction of WM but to report the demographics so that we understand the dataset (and any implicit biases therein) better. This is especially true in light of the

selection of subset of images.
14. Regarding QA/QC, the authors have responded with "since all the MRIs of this study are from public datasets, apparently the MRIs have been checked once by the dataset providers" - this is not true. There is no obligation on the part of the dataset providers to check the data prior to their release; this responsibility lies with the analysts. For example, ADNI provides QC ratings for different scans and clearly states that "users can employ scan-level QC information as filters in preparing image collections.". For OASIS-3, there is post processing QC on Freesurfer output but that does not imply that the raw dataset (which the present study uses) is QCed. Further the authors state "…therefore we expect no MRI with severe noise or artifact to be present among the data." - this is, again, an incorrect presumption. Several of these datasets are known to have problematic images. I repeat my comment from the first round: "There is no mention of quality checks performed on the images prior to their inclusion in the analysis - this is critical … - please provide documentation on what were the QA/QC procedures employed. If these were not done, there needs to be a very good reason why not." Filtering scans based on the number of slices is not really quality check.
15. For OASIS-3, how were scans selected between repeated measurements?
Results
16. Generally speaking, please indicate the standard deviation along with all the reported mean performance measures in the text.
17. Setting A00: there is at least one instance where the specificity drops close to 50% - what could have happened there? Since the datasets are balanced, it can't be because of class imbalance. Similarly, there are at least two scenarios where the accuracy is <= 80%. Could you look into this and find out what could have caused this variability? Seems to indicate that there are a few specific random splits where the model does not perform quite well - is this related to the 3D/2D scenario?
18. In all A* scenarios, the specificity is always on the higher side. Why? Is it that during training, the model is overfitting to the positive class (perhaps incorrectly learning most data points as positive class) and failing to generalize?
19. The authors speculate that with more shuffles A11-A18 will likely converge to chance - this doesn't seem likely. You are already plotting/examining across 200 shuffles + repeats - there would have been reasonable indication that results are converging to chance. On the contrary, the specificity is still averaging around 80% (A18) and sensitivity around 50%. The authors say that looking into this is beyond the scope of the study - well, isn't it central to your model? If the model is, in the absence of any reasonable data (only four images), still predicting quite a bit above chance, what does it indicate? Seems like a suspect case of some sort of data leakage happening such that the model is still able to predict ~65% of the cases correctly.
20. To investigate the above, I had suggested permutation testing - however, the authors have only done that for A00; that does not tell us whether the tail end of the settings are still above chance or is 65% accuracy in the chance limits. If the latter, why is the chance performance not centred at 50%?
21. In fact, for the accuracy plots, it would be meaningful to additionally include the permutation dots for each box plot (i.e., keep the box plots as such but additionally plot the jittered points from permutation testing for each of the scenarios). This establishes a reasonable distribution of how much performance can be expected by chance and the true distribution. The authors have argued that there is high computational cost - in settings like A18, isn't there only four MRI scans to learn from? Model

training is the most cost intensive step, shouldn't the computational cost be minimal for such extreme settings?
22. In setting B, we see the opposite effect of setting A - the specificity drops consistently while the sensitivity remains quite high - why does this happen? These results need to be examined and discussed.
23. Again, the authors have claimed that the accuracy will converge to chance with more shuffles and repeats - but these are already adequate repeats (200).
24. I do not see permutation testing results for settings B, C, or D.
25. For setting C, between C02 and C03, the sample sizes are not that different: 46 and 36. However, the specificity of your models plummet from 85% to 30%. Similar to setting B, we see quite high sensitivity throughout (even in C06; >85%) while the specificity is the only one which takes a hit. Seems to be a scenario where the majority of the times the prediction is a positive class?
26. Regarding the possibility of overfitting to the test, the authors argue that they have "not learnt any lesson from the first test". The biggest lesson learnt from the first test was the fact that 2D images were a source of poor performance. Not only was this lesson learnt, but a decision was made from this lesson - the decision to eliminate 2D scans. While it is true that the authors have recreated the models and run the tests in multiple shuffles, it does not avoid the problem of overfitting to the test set. You have looked at the entire data, made a decision, and then re-run the pipeline which results in better performance. This is problematic and needs to be adequately mentioned with the results considerably downplayed, with an acknowledgement that this could be overfitted to the test.
27. Usually, one begins to see convergence to chance in 50-100 trials; in your experiments, there are 200 trials. It is a bit suspect that none of the extreme scenarios have converged to chance.
28. It is hard to visually compare plots given the authors decision to not make the axes equal across; if they are concerned about some of the values not being very visible, they can always include zoomed in versions of the plot.
Overall comments
29. Please include relevant permutation testing and the distribution of values for all scenarios. They are essential more for evaluating how much performance is expected by chance rather than for calculating p values (which can be trivially small).
30. Regarding citing prior literature: as mentioned in the first round, the authors have completely glossed over any direct prior work done in the area. There is a lot of work that has happened in the area: if these methods/papers produce results which are slightly different than the authors' result (i.e., labelling of white matter hyperintensity vs. binary output or volumetric assessment vs. binary output), it does not mean that prior work can be glossed over. I strongly suggest that the authors bring in (in the introduction section at least) the prior work in the field and highlight why and how their approach is different.
31. On just doing a quick Google search on the topic, I found a series of relevant papers which have used ML/DL approaches for WM lesion detection / labelling. Examples of these include: Zhu et al (2022, Frontiers in Aging Neuroscience), Guerrero et al (2018, NeuroImage Clinical), Tran et al (2022, NeuroImage Clinical), Mu et al (2024, Computer Methods and Programs in Biomedicine), Kuwabara et al (2024, Scientific Reports), Zhang et al (2022, Neuroradiology), to name just a few of the ones I found. In fact, a few of these (and the comparators cited therein) are extremely relevant papers since they use similar approaches as the present work. For example, Zhu et al have done a series of comparisons in

their work and they cite to other state of the art methods (back then) such as uResNet, 3D V-Net, and VGGNet. Guerrero et al point to DeepMedic. Beyond these DL methods, there are other series of methods that exist such as lesion prediction algorithm, lesion segmentation tool, lesion growth algorithm, BIANCA, and probably many more. And these are based on a quick search, not a deep dive into the literature; now some of these may not have the code available (to be used as a comparator) or may not be directly relevant due to whatever reason, but to completely miss the entire field in the introduction/discussion and state that there is no comparison possible at all is incorrect.
32. The authors argue that the value of an appropriate threshold needs to be set for using many of these methods as a comparator. Fair point. However, it is not that difficult to do so. For example, Zhu et al used a 3D connected component to binarize the output - if you have even one connected component standing, treat it as WMH true. Guerrero et al discuss thresholds for some of the methods in their section of comparison to gold standard.
33. Similarly, the need for a training set in BIANCA is well known - there is publicly available training set; see, for example, https://git.fmrib.ox.ac.uk/open-science/analysis/wmh_harmonisation/-/tree/master/BIANCA_training_datasets
34. The whole point of the comparison is to gain a perspective on how well HeteroMRI is doing compared to what is already done in the field. This is especially true since several of the DL based methods (some of which I cited above) make the exact claim that their method is robust to varying protocols.
35. Regarding out of sample validation: while it is true that the authors have attempted to show the performance of HeteroMRI in leave-a-site-out kind of design, it would be more convincing to see the results in a truly out of sample situation. The ideal setting would be when you mix a few images from a range of studies and treat them as truly out of sample (i.e., not include them in the train-validation-test design of your experiments). There is little computational cost to this because your model is already trained. You are taking the pre-trained "final" model that you have created and are merely applying it to out of sample images which may come from different scanners/protocols/sites. In this setting, the held out sample is not biased because it contains diverse protocols and ideally has data independent of any of the protocols used in the study. Again, this is not a combinatorial problem and does not have any significant computational cost since the model is being applied to the test set. Class imbalance does not picture in this setting because you can compute sensitivity, specificity, and balanced accuracy irrespective of class imbalance. If the authors cannot identify any scenario at all where they have external dataset to work with (that would be hard to believe given the extensive work that has already happened in the field with several papers sharing manual annotation and raw images), one could still take all the unused FLAIR images from ADNI, OASIS, and other datasets (irrespective of case status) and do an out of sample application. Once again, class imbalance is not an issue here. The overall point of these comments is that in the absence of any comparators and any out of sample application, it is hard to truly evaluate the performance of any DL model.
36. One of the reasons for why the authors are finding it hard to find a comparator which gives binary response to an input FLAIR image is the question "how useful is a binary classifier over a WM abnormality labelling method?". The authors mention that their goal is to use the method (in the future) for classifying MRIs based on white matter diseases. A similar evaluation was performed in Guerrero et al (WMH vs. stroke lesion) and probably in other work too. Even in that scenario, would it not be

reasonable to get annotation maps out of HeteroMRI? You can always convert labelled images into a binary decision but not the other way round. We need ML models to assist clinical decision making by providing reasonable probabilities and (in case of images) labelled data so that the clinician can use the data for informed decision making; not make the decisions for the clinician. If the use case audience are medical trainees and MRI operators, then those should have been identified rather than broad clinical practice in the introduction. Overall, these aspects need to be discussed and also listed as limitation of the proposed method.

37. To come back to the point of the WM clustering step making the method robust to multiple MR acquisition protocols, here is a possible test scenario: if you took white matter segmentation probability maps from a standard package (SPM, CAT, FSL, etc.) and repeated the leave-one-protocol-out type of test, would you see comparable performance across the folds? In this scenario, the actual performance of the model does not matter much - it is the stability of the performance (even if it is say 65%). If it is the WM clustering step which brings in the robustness to protocol differences and not the DL method, one could also flip it around and use any other ML/DL method/architecture with the segmentation maps and they ought to result in robust performance.

38. Please include a discussion about the limitation of the current work since all the evaluated scenarios are idealised balanced case which are far removed from the clinical setting.

Methods

Are the methods appropriate to the aims of the study, are they well described, and are necessary controls included? Choose an item.

Conclusions

Are the conclusions adequately supported by the data shown? Choose an item.

Reporting Standards

Does the manuscript adhere to the journal's guidelines on [minimum standards of reporting?]() Choose an item.

Choose an item.

Statistics

Are you able to assess all statistics in the manuscript, including the appropriateness of statistical tests used? Choose an item.

Quality of Written English

Please indicate the quality of language in the manuscript: Choose an item.

Declaration of Competing Interests

Please complete a declaration of competing interests, considering the following questions:

- Have you in the past five years received reimbursements, fees, funding, or salary from an organisation that may in any way gain or lose financially from the publication of this manuscript, either now or in the future?
- Do you hold any stocks or shares in an organisation that may in any way gain or lose financially from the publication of this manuscript, either now or in the future?
- Do you hold or are you currently applying for any patents relating to the content of the manuscript?
- Have you received reimbursements, fees, funding, or salary from an organization that holds or has applied for patents relating to the content of the manuscript?
- Do you have any other financial competing interests?
- Do you have any non-financial competing interests in relation to this paper?

If you can answer no to all of the above, write 'I declare that I have no competing interests' below. If your reply is yes to any, please give details below.

I declare that I have no competing interests

I agree to the open peer review policy of the journal. I understand that my name will be included on my report to the authors and, if the manuscript is accepted for publication, my named report including any attachments I upload will be posted on the website along with the authors' responses. I agree for my report to be made available under an Open Access Creative Commons CC-BY license (http://creativecommons.org/licenses/by/4.0/). I understand that any comments which I do not wish to be included in my named report can be included as confidential comments to the editors, which will not be published.

Choose an item.

To further support our reviewers, we have joined with Publons, where you can gain additional credit to further highlight your hard work (see: https://publons.com/journal/530/gigascience). On publication of this paper, your review will be automatically added to Publons, you can then choose whether or not to claim your Publons credit. I understand this statement.

Yes Choose an item.
