# Supplementary material for: HeteroMRI: Robust white matter abnormality classification across multi-scanner MRI data
Source: Gigascience. 2025 Aug 21;14:giaf092. doi: 10.1093/gigascience/giaf092 (PMC12371411; doi:10.1093/gigascience/giaf092)
Supplement: giaf092_Reviewer_2_Report_Original_Submission — Chiara Marzi -- 8/30/2024 [file giaf092_reviewer_2_report_original_submission.pdf]

Reviewer Report

Title: HeteroMRI: Robust white matter abnormality classification across multi-scanner MRI data

Version: Original Submission **Date:** 8/30/2024

Reviewer name: Chiara Marzi

Reviewer Comments to Author:

The authors aim to develop a deep-learning method, HeteroMRI, that minimizes the effects of MRI scanner and protocol heterogeneity in the analysis of white matter abnormalities. Their method achieves high accuracy in binary classification of brain MRIs, even with limited data, thus improving the potential for machine learning integration into clinical MRI practices regardless of scanning conditions.
Major comments in order of appearance within the text

1. It is unclear how the variability due to different MR scanners and acquisition protocols is identified and attenuated. Is it done through WM clustering, or is it directly addressed within the Deep Learning model designed for the classification task?
2. In Figure 1, do all the images, except MNI, refer to the same subject? I would also suggest being as consistent as possible in selecting the position along the axial direction, as it does not appear to be consistent in the "MRI preprocessing."
3. The authors state, "A non-linear registration is applied three times (with identical parameters)." It is unclear why the registration procedure is repeated three times in series. I recommend that the authors provide references regarding the utility of performing multiple registrations and include all parameters used for co-registration (e.g., degrees of freedom, type of interpolation, etc.).
4. In the "WM clustering" paragraph, the authors state that they chose to use three clusters based on empirical observations. What data were these observations derived from? If these empirical tests were performed on the same data used for WM clustering and classification, this constitutes data leakage, potentially leading to overestimated classification performance. Similarly, the authors write: "Finally, all the selected clusters from all the MRIs (one intensity cluster per MRI) serve as the training data for the DL model, as described in the following section." This is another case of significant data leakage, which should be avoided, preferably by design in the processing workflow, by performing WM clustering only on the training data and applying it solely to the test data.
5. In the description of the various experimental settings, there are cases where the training is done with very few images (4, 10, 8, ...). How is it possible to train a deep architecture with so many parameters using such a small number of examples?
6. In the "3.3 Model Execution" section, was the data split stratified to maintain the same proportion of subjects with and without WM abnormalities, as in the entire multicenter cohort? If not, accuracy is not an appropriate metric.
7. The authors state that they trained and tested the various models 10 times. Was an average performance reported?
8. Section 4 is titled "Results and Discussion," but the discussion is completely absent. A critical integration of the current work with previous literature is a crucial missing component.

9. The text between lines 403 and 415 should be included in the methods section rather than int the results section.
10. The percentage of 2D MRI scans should be clearly reported in the data description section.
11. After registration, it is mandatory to check the images and exclude those with poor registration output.
12. In section "4.2 Setting B", the authors state that "Setting B, which uses an equal number of MRIs from each MRI protocol, is designed to make the prediction task more challenging for the model." Why would the task be more complex if the situation is perfectly balanced?
13. The conclusions are too lengthy and serve as a summary of the work and results rather than true conclusions.

Minor comments in order of appearance within the text

1. Line 49: "white matter" and "gray matter" do not need to be capitalized.
2. Line 52: the acronym "FLAIR" should be introduced after, not before, its explanation.
3. Lines 85-94: add that the variability due to different scanners and acquisition protocols is often greater than the biological variability (see, e.g., references (Fortin et al., 2018, 2017, 2016; Marzi et al., 2024; Pomponio et al., 2020; Radua et al., 2020)
4. Line 113: the phrase "are either based on statistics or ??? learning (AI???)" needs clarification.
5. Line 115: add (Marzi et al., 2024; Pomponio et al., 2020; Radua et al., 2020) to the references for "Adjusting for batch effects."
6. Lines 153-160 should be moved earlier in the text.
7. Line 177: correct "Secs. 4" to "Sec. 4."
8. Line 181: revise to "as the data input for a CNN."
9. In the MSSEG-2 dataset, why did you only consider 10 scanners instead of 12? A few lines earlier, the authors themselves state, "This variability persisted even among the sites that had scanners from the same manufacturer and model."
10. Lines 479 and 489: what are the layers referring to?
11. Line 483: the sentence "In a nutshell, by training the model with only 46 3D MRIs, it is able to classify MRIs acquired with protocols unseen during training with 88.1% accuracy." is redundant, as it was already mentioned in the previous line.
12. Line 494: what does the "#" symbol signify?

References

Fortin, J.P., Cullen, N., Sheline, Y.I., Taylor, W.D., Aselcioglu, I., Cook, P.A., Adams, P., Cooper, C., Fava, M., McGrath, P.J., McInnis, M., Phillips, M.L., Trivedi, M.H., Weissman, M.M., Shinohara, R.T., 2018. Harmonization of cortical thickness measurements across scanners and sites. Neuroimage 167, 104-120. https://doi.org/10.1016/j.neuroimage.2017.11.024

Fortin, J.P., Parker, D., TunÃ§, B., Watanabe, T., Elliott, M.A., Ruparel, K., Roalf, D.R., Satterthwaite, T.D., Gur, R.C., Gur, R.E., Schultz, R.T., Verma, R., Shinohara, R.T., 2017. Harmonization of multi-site diffusion tensor imaging data. Neuroimage 161, 149-170. https://doi.org/10.1016/j.neuroimage.2017.08.047

Fortin, J.-P., Sweeney, E.M., Muschelli, J., Crainiceanu, C.M., Shinohara, R.T., 2016. Removing inter-subject technical variability in magnetic resonance imaging studies. NeuroImage 132, 198-212. https://doi.org/10.1016/j.neuroimage.2016.02.036

Marzi, C., Giannelli, M., Barucci, A., Tessa, C., Mascalchi, M., Diciotti, S., 2024. Efficacy of MRI data

harmonization in the age of machine learning: a multicenter study across 36 datasets. Sci Data 11, 115. https://doi.org/10.1038/s41597-023-02421-7
Pomponio, R., Erus, G., Habes, M., Doshi, J., Srinivasan, D., Mamourian, E., Bashyam, V., Nasrallah, I.M., Satterthwaite, T.D., Fan, Y., Launer, L.J., Masters, C.L., Maruff, P., Zhuo, C., VÃ¶lzke, H., Johnson, S.C., Fripp, J., Koutsouleris, N., Wolf, D.H., Gur, Raquel, Gur, Ruben, Morris, J., Albert, M.S., Grabe, H.J., Resnick, S.M., Bryan, R.N., Wolk, D.A., Shinohara, R.T., Shou, H., Davatzikos, C., 2020. Harmonization of large MRI datasets for the analysis of brain imaging patterns throughout the lifespan. NeuroImage 208, 116450. https://doi.org/10.1016/j.neuroimage.2019.116450
Radua, J., Vieta, E., Shinohara, R., Kochunov, P., QuidÃ©, Y., Green, M.J., Weickert, C.S., Weickert, T., Bruggemann, J., Kircher, T., NenadiÄ‡, I., Cairns, M.J., Seal, M., Schall, U., Henskens, F., Fullerton, J.M., Mowry, B., Pantelis, C., Lenroot, R., Cropley, V., Loughland, C., Scott, R., Wolf, D., Satterthwaite, T.D., Tan, Y., Sim, K., Piras, Fabrizio, Spalletta, G., Banaj, N., Pomarol-Clotet, E., Solanes, A., Albajes-Eizagirre, A., Canales-RodrÃguez, E.J., Sarro, S., Di Giorgio, A., Bertolino, A., StÃ¤blein, M., Oertel, V., KnÃ¶chel, C., Borgwardt, S., du Plessis, S., Yun, J.-Y., Kwon, J.S., Dannlowski, U., Hahn, T., Grotegerd, D., Alloza, C., Arango, C., Janssen, J., DÃaz-Caneja, C., Jiang, W., Calhoun, V., Ehrlich, S., Yang, K., Cascella, N.G., Takayanagi, Y., Sawa, A., Tomyshev, A., Lebedeva, I., Kaleda, V., Kirschner, M., Hoschl, C., Tomecek, D., Skoch, A., van Amelsvoort, T., Bakker, G., James, A., Preda, A., Weideman, A., Stein, D.J., Howells, F., Uhlmann, A., Temmingh, H., LÃ³pez-Jaramillo, C., DÃaz-Zuluaga, A., Fortea, L., Martinez-Heras, E., Solana, E., Llufriu, S., Jahanshad, N., Thompson, P., Turner, J., van Erp, T., Glahn, D., Pearlson, G., Hong, E., Krug, A., Carr, V., Tooney, P., Cooper, G., Rasser, P., Michie, P., Catts, S., Gur, Raquel, Gur, Ruben, Yang, F., Fan, F., Chen, J., Guo, H., Tan, S., Wang, Z., Xiang, H., Piras, Federica, Assogna, F., Salvador, R., McKenna, P., Bonvino, A., King, M., Kaiser, S., Nguyen, D., Pineda-Zapata, J., 2020. Increased power by harmonizing structural MRI site differences with the ComBat batch adjustment method in ENIGMA. NeuroImage 218, 116956. https://doi.org/10.1016/j.neuroimage.2020.116956

Methods

Are the methods appropriate to the aims of the study, are they well described, and are necessary controls included? Choose an item.

Conclusions

Are the conclusions adequately supported by the data shown? Choose an item.

Reporting Standards

Does the manuscript adhere to the journal’s guidelines on [minimum standards of reporting?](#) Choose an item.

Choose an item.

Statistics

Are you able to assess all statistics in the manuscript, including the appropriateness of statistical tests used? Choose an item.

Quality of Written English

Please indicate the quality of language in the manuscript: Choose an item.

Declaration of Competing Interests

Please complete a declaration of competing interests, considering the following questions:

- Have you in the past five years received reimbursements, fees, funding, or salary from an organisation that may in any way gain or lose financially from the publication of this manuscript, either now or in the future?
- Do you hold any stocks or shares in an organisation that may in any way gain or lose financially from the publication of this manuscript, either now or in the future?
- Do you hold or are you currently applying for any patents relating to the content of the manuscript?
- Have you received reimbursements, fees, funding, or salary from an organization that holds or has applied for patents relating to the content of the manuscript?
- Do you have any other financial competing interests?
- Do you have any non-financial competing interests in relation to this paper?

If you can answer no to all of the above, write 'I declare that I have no competing interests' below. If your reply is yes to any, please give details below.

I declare that I have no competing interests.

I agree to the open peer review policy of the journal. I understand that my name will be included on my report to the authors and, if the manuscript is accepted for publication, my named report including any attachments I upload will be posted on the website along with the authors' responses. I agree for my report to be made available under an Open Access Creative Commons CC-BY license (http://creativecommons.org/licenses/by/4.0/). I understand that any comments which I do not wish to be included in my named report can be included as confidential comments to the editors, which will not be published.

Choose an item.

To further support our reviewers, we have joined with Publons, where you can gain additional credit to further highlight your hard work (see: https://publons.com/journal/530/gigascience). On publication of this paper, your review will be automatically added to Publons, you can then choose whether or not to claim your Publons credit. I understand this statement.

Yes Choose an item.
